# Unique proteome signature of post-chemotherapy ovarian cancer ascites-derived tumor cells

**DOI:** 10.1038/srep30061

**Published:** 2016-07-29

**Authors:** Nuzhat Ahmed, David Greening, Chantel Samardzija, Ruth M. Escalona, Maoshan Chen, Jock K. Findlay, George Kannourakis

**Affiliations:** 1 Fiona Elsey Cancer Research Institute, Ballarat, Victoria 3353, Australia; 2 Federation University Australia, Ballarat, Victoria 3010, Australia; 3 Department of Obstetrics and Gynaecology, University of Melbourne, Victoria 3052, Australia; 4 The Hudson Institute of Medical Research, Victoria 3168, Australia; 5 Department of Biochemistry and Genetics, La Trobe Institute for Molecular Science, La Trobe University, Bundoora, Victoria 3086, Australia

## Abstract

Eighty % of ovarian cancer patients diagnosed at an advanced-stage have complete remission after initial surgery and chemotherapy. However, most patients die within <5 years due to episodes of recurrences resulting from the growth of residual chemoresistant cells. In an effort to identify mechanisms associated with chemoresistance and recurrence, we compared the expression of proteins in ascites-derived tumor cells isolated from advanced-stage ovarian cancer patients obtained at diagnosis (chemonaive, CN) and after chemotherapy treatments (chemoresistant/at recurrence, CR) by using in**-**depth, high-resolution label-free quantitative proteomic profiling. A total of 2,999 proteins were identified. Using a stringent selection criterion to define only significantly differentially expressed proteins, we report identification of 353 proteins. There were significant differences in proteins encoding for immune surveillance, DNA repair mechanisms, cytoskeleton rearrangement, cell-cell adhesion, cell cycle pathways, cellular transport, and proteins involved with glycine/proline/arginine synthesis in tumor cells isolated from CR relative to CN patients. Pathway analyses revealed enrichment of metabolic pathways, DNA repair mechanisms and energy metabolism pathways in CR tumor cells. In conclusion, this is the first proteomics study to comprehensively analyze ascites-derived tumor cells from CN and CR ovarian cancer patients.

Ovarian cancer is usually not detected until late in the advanced-stages (stage III–IV), when it has spread beyond ovaries to the adjacent abdominal organs^1^. Initially, most (~80%) advanced-stage patients respond to surgical debulking and chemotherapy treatment, but almost all relapse within a few months due to drug-resistant residual disease^2^. The five year survival period of advanced-stage ovarian cancer patients has remained unchanged and disappointingly low at ~30% for the last thirty years^3^. Hence, there is an urgent need to understand the mechanisms of recurrence and chemoresistance in order to design future treatment strategies which will provide long-term disease-free and overall survival periods for ovarian cancer patients.

The progression of ovarian cancer is characterized by rapid growth and spread of peritoneal tumors, and in most cases is accompanied by accumulation of ascites within the peritoneum^1^, which either in the chemonaive or chemoresistant phase carries a bad prognosis^3^,^4^. Malignant ascites constitutes a dynamic reservoir of survival factors, including cytokines, chemokines, growth factors, and extracellular matrix (ECM) fragments which individually and in a combined fashion affect tumor cell growth and progression through different cellular mechanisms^4^. Ascites also contains a complex mixture of ‘resident cells’ such as tumor and cancer associated fibroblasts or stromal cells and ‘non-resident cells’ such as infiltrating immune cells and bone-marrow derived mesenchymal stem cells^4^. Each population of cells has a defined role and is connected with each other through signaling by the ‘in house’ soluble factors^4^. Tumor cells within the ascites of ovarian cancer patients are present either as single cells or more commonly as aggregates of non-adherent cells commonly known as spheroids^5^,^6^. In this scenario, multiple (a few hundred) tumor aggregates can be seen either floating or embedded in the peritoneal cavity during primary debulking surgery^1^,^5^. Hence, there is a considerable heterogeneity in the ascites tumor population of ovarian cancer patients.

In the past several years, studies have reported proteomic analyses of ascites fluid in an attempt to identify biomarkers for ovarian cancer^7^,^8^,^9^. However, to date no study has been done on ascites-derived tumor cells isolated from chemonaive and chemoresistant patients to understand the mechanisms of chemoresistance and associated recurrence. A limited number of studies have identified differentially expressed proteins between parental and *in vitro* drug-induced chemoresistant cell lines^10^,^11^,^12^ which have showed some relevance to the potential mechanisms of chemoresistance in clinical samples. However, isolated tumor cells in the ascites of cancer patients that have survived chemotherapy treatments and re-emerged as recurrent tumors are likely to experience *in vivo* proteome changes that would allow them to withstand the cytotoxic pressure of chemotherapy. We hypothesize that these ‘therapy resistant distinct’ tumor cells are likely to display a proteomic signature associated with chemoresistance which is different from that of the proteome of isolated tumor cells of chemonaive patients.

In the present study we have used our recently described novel separation technique to isolate tumor cells from the ascites of advanced-stage chemonaive and recurrent serous ovarian cancer patients^5^. Ascites were collected from patients at the time of surgery prior to chemotherapy treatment (CN) and at recurrence (CR). In this preliminary study, label-free quantitative proteomics was used to identify and monitor protein expression changes associated with chemoresistance and recurrence, utilising a set of unmatched CN (n = 4) and CR (n = 4) patient-derived samples. The overall aim was to identify a ‘unique proteome signature’ distinct to the ascites-derived tumor cells of CR patients so that novel information about specific proteins and pathways associated with the chemoresistant/recurrent trait of tumor cells in the ascites can be obtained. Our data identified novel and differentially expressed proteins and associated pathways which will provide better understanding of the intraperitoneal spread of recurrent ovarian cancer. To our knowledge, this is the first study using comparative proteomic analysis to describe the unique protein signature between ascites-derived tumor cells from CN and CR patients.

## Methods and Materials

### Patient Recruitment

Ascites were collected from patients diagnosed with advanced-stage serous ovarian carcinoma after obtaining written informed consent under protocols approved by the Research and Human Ethics Committee (HEC # 09/09) of The Royal Women’s Hospital, Melbourne, Australia. The histopathological diagnosis, tumor grades and stages were determined by independent staff pathologists as part of the clinical diagnosis. All patients recruited were diagnosed with high grade serous carcinomas (Supplementary Table S1). Ascites (As) samples (500 ml-2 L) were obtained before surgery from patients with primary carcinoma (n = 4), and at the time of recurrence following chemotherapy treatment (n = 4). Ascites collected prior to treatment were termed chemonaive (CN), while those collected from patients diagnosed with recurrent disease within 6–20 months after their original diagnosis and which has been treated with chemotherapy were termed chemoresistant/recurrent (CR). The chemotherapy agents and number of chemotherapy cycles administered to patients varied from patient to patient and are indicated in Supplementary Table S1.

### Harvest of tumor cells from ascites of ovarian cancer patients

Tumor cells from fresh ascites samples were isolated as described previously^5^. Following isolation, ascites tumor cells (usually 10^5^–10^6^ cells) were cultured on low attachment plates (Corning Incorporated, NY) in MCDB: DMEM growth medium (50:50) supplemented with heat inactivated fetal bovine serum (10%), glutamine (2 mM) and penicillin/streptomycin (2 mM) (Life Technologies, CA, USA). Cells were maintained at 37 °C in the presence of 5% CO_2_ and tumor cells floating as non-adherent spheroid populations were collected after 2–3 days. Non-adherent cells were screened for CA125, EpCAM, cytokeratin 7 (CK7) and fibroblast surface protein (FSP) by Flow Cytometry to assess their purity before being stored at −80°C until required for analysis.

### Primary cell lysate preparation

Tumor cells were washed (ice cold PBS) and lysed on ice with SDS sample buffer (4% (w/v) SDS, 20% (v/v) glycerol, 0.01% (v/v) bromophenol blue, 0.125 M Tris-HCl, pH 6.8). Lysates were subjected to ultracentrifugation for 30 min at 4 °C (386,000 × *g*, TLA-100 rotor, Beckman Coulter), and soluble supernatants aspirated for downstream use, or frozen at −80 °C.

### Proteomic analysis

Protein content was estimated by 1D-SDS-PAGE/SYPRO^®^ Ruby protein staining-based densitometry, as previously described^13^. Proteomic experiments were performed on primary cell lysates (n = 4) in technical duplicate as described previously^14^. Briefly, lysates from CN or CR tumor cells (10 μg) were separated by short-range SDS-PAGE (10 mm), and visualized by Imperial Protein Stain (Invitrogen, Thermo Fisher Scientific, Australia). Individual samples (n = 2, equal gel slices of 7 × 5 mm) were excised, destained (50 mM ammonium bicarbonate/acetonitrile), reduced [10 mM DTT (Calbiochem, Merck, Australia) for 30 min], alkylated [50 mM iodoacetic acid for 30 min] and trypsinized [0.2 μg trypsin (Promega Sequencing Grade, Australia) for 16 h at 37 °C]. A nanoflow UPLC instrument (Ultimate 3000 RSLCnano, Thermo Fisher Scientific) was coupled on-line to an Orbitrap Elite mass spectrometer (Thermo Fisher Scientific, Australia) with a nanoelectrospray ion source (Thermo Fisher Scientific). Peptides were loaded (Acclaim PepMap100, 5 mm × 300 μm i.d., μ-Precolumn packed with 5 μm C18 beads, Thermo Fisher Scientific, Australia) and separated (Acquity UPLC M-Class Peptide BEH130, C18, 1.7 μm, 75 μm × 250 mm, Waters, Australia). Data was acquired using Xcalibur software v2.1 (Thermo Fisher Scientific). Details of the operation of the mass spectrometer are described previously^14^.

### Database searching and protein identification

Raw data was processed using Proteome Discoverer (v1.4.0.288) and searched with Mascot (Matrix Science, London, UK; v 1.4.0.288), Sequest (Thermo Fisher Scientific, San Jose, CA, v 1.4.0.288), and X! Tandem (v 2010,12.01.1) against a database of 125,803 ORFs (Uniprot Human, Jul-2015) and common contaminants. Data was searched as described previously^15^,^16^ with a parent tolerance of 10 ppm, fragment tolerance of 0.5 Da and minimum peptide length 7, with FDR 1% at the peptide and protein levels. Scaffold (Proteome Software Inc., Portland, OR, v 4.3.4) was employed to validate MS/MS-based peptide and protein identifications from database searching. Data was further examined using semi-quantitative label-free spectral counting^13^,^16^,^17^. Ratio of spectral count [Rsc (CR/CN)] values were normalised by the total number of significant MS/MS spectra identified in each sample and fold change ratios calculated. Total number of spectra was only counted for significant peptides identified (Ion score ≥ Homology score). When Rsc was less than 1, the negative inverse value was used. The number of significant assigned spectra for each protein was used to determine protein expression differences. For each protein the Fisher’s exact test was applied to significant assigned spectra. The resulting p-values were corrected for multiple testing using the Benjamini-Hochberg procedure and statistics performed as previously described^18^. Contaminants and reverse database identifications were excluded from data analysis.

Protein identifications were accepted, if they reached greater than 99% probability and contained at least 2 identified unique peptides. These identification criteria typically established <1% false discovery rate based on a decoy database search strategy at the protein level. Proteins that contained similar peptides and could not be differentiated based on MS/MS analysis alone were grouped to satisfy the principles of parsimony. Contaminants, and reverse identification were excluded from further data analysis. UniProt was used for protein annotation (molecular function/subcellular localisation) and KEGG (http://www.genome.jp/kegg/pathway.html) and DAVID (http://david.abcc.ncifcrf.gov/) for pathway enrichment analyses. Corrplot (R package, https://cran.r-project.org/web/packages/corrplot/) and Cluster (v3.0)^19^ were used to evaluate the correlation across all the samples and combined samples. MEGA (v6.06)^20^ was used to visualize the phylogeny tree. Raw data set of proteins identified in CN and CR tumor cells is described in Supplementary Table 2.

### SDS-PAGE and Western blot analysis

SDS-PAGE and Western blot was performed on tumor cell lysates by the methods described previously^21^. For immunoblotting, cell lysates (n = 4) (10 μg) resolved on 10% SDS-PAGE and transferred to nitrocellulose membranes were probed with primary antibodies [mouse anti-human (1:1000)], TOP2A (Cell Signaling Technology, Danvers, MA, USA), PYCR2 and PPL (Santa Cruz Biotechnology, Dallas, USA) for 1 h in TTBS [50 mM Tris, 150 mM NaCl, 0.05% (v/v) Tween 20] followed by incubation with corresponding secondary antibodies; IRDye 800 goat anti-mouse IgG or IRDye 700 goat anti-rabbit IgG (1:15000, LI-COR Biosciences), for 1 h at room temperature in TTBS. Immunoblots were imaged using the CLx Odyssey Infrared Imaging System, (v3.0, LI-COR Biosciences, Nebraska USA). Loading controls were obtained by staining the membrane with Deep Purple Total Protein stain as previously described^15^,^16^. Semi-quantitative densitometric analysis was performed on all blots (3 biological replicates) to determine the level of protein expression using ImageStudio v5, with mean pixel intensity of the protein of interest normalised to the background.

### Statistical analysis

Student’s t-test was used for statistical analysis which involved comparison between two groups. Unless otherwise stated, data are presented as mean ± SEM (n = 4 biological replicates), with *p < 0.05 considered statistically significant. Data was analysed using the GraphPad Prism Software v5. All experiments were repeated three times unless otherwise indicated.

## Results

### Isolation and characterisation of ascites-derived tumor cells

**Figure 1:**
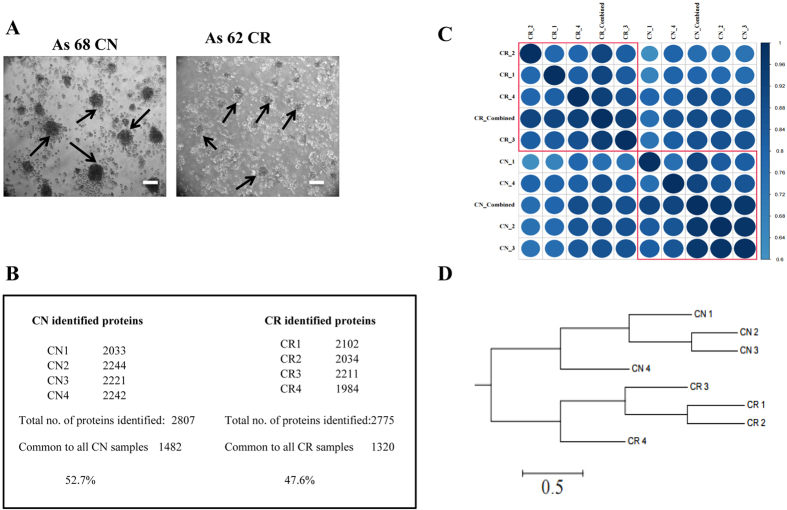
Morphological features of ascites-derived spheroids. **(A)** Representative light microscopy images of non-adherent spheroid tumor populations grown on low-attachment plates derived from CN and CR serous ovarian cancer patient ascites samples. Images are representative of n = 4 samples/group. Arrows indicate non-adherent spheroid ascites tumour populations. Magnification is set at 100x, scale bar = 100μM. **(B)** CN and CR non-adherent spheroid cellular samples were profiled using discovery-based proteomics. Samples were analyzed as technical replicates (n = 2), stringent peptide and protein identification criteria were implemented (1% FDR protein, 5% PEP), with proteins requiring at least two significant peptides for identification. For CN samples (2807 proteins identified, 1482 proteins in common across CN1-4), while for CR samples (2775 proteins identified, 1320 proteins in common across CR1–4). **(C)** Correlation matrix of CN (1–4 replicates and combined) and CR (1–4 replicates and combined) samples showing that each individual sample represents high similarity with other sample replicates of the same cohort. **(D)** Cluster analysis of CN and CR replicates showing clear distribution between sample groups.

Ascites-derived tumor cells from both CN (n = 4) and CR patients (n = 4) were assessed by phase contrast microscopy after seeding on low attachment plates for 24 h (Fig. 1A). As previously described^6^, two distinct populations of cells were observed; (i) non-adherent cellular aggregates (spheroids) that floated as three-dimensional structures in the growth medium without attachment to the plates (Fig. 1A), and (ii) spindle shaped fibroblast-like single cells that adhered to the low attachment plates^5^. Based on our previous studies which identified non-adherent population to be tumorigenic^5^, only non-adherent spheroid populations were assessed by Flow Cytometry in this study (Fig. 1A). As described previously^5^, high expression of cancer antigen 125 (CA125) and cytokeratin 7 (CK7) was observed in the cells dispersed from non-adherent spheroids, while no significant expression of fibroblast surface protein (FSP) was evident.

### Proteome analysis of ascites-derived tumor cells before and after chemotherapy treatments

To identify possible mechanisms responsible for chemoresistance and associated recurrence in serous ovarian cancer patients, we compared the expression of proteins in non-adherent tumor cells isolated from the ascites of untreated (CN) and chemotherapy treated (CR) serous ovarian cancer patients. Using high-resolution mass spectrometry analysis, the ascites-derived CN and CR tumor cell populations showed a high degree of similarity in relation to protein content portraying an average of 2807 and 2775 proteins (Fig. 1B–D, Supplementary Table S2). There was a high degree of correlation between the samples of the same cohort, with the 4 CN samples showing correlation of 52.7% while 47.6% correlation was noted between the 4 CR samples (Fig. 1B). A correlation plot between the samples is presented in Fig. 1C, while Fig. 1D represents a relationship tree portraying significant differences between CN and CR samples.

**Figure 2:**
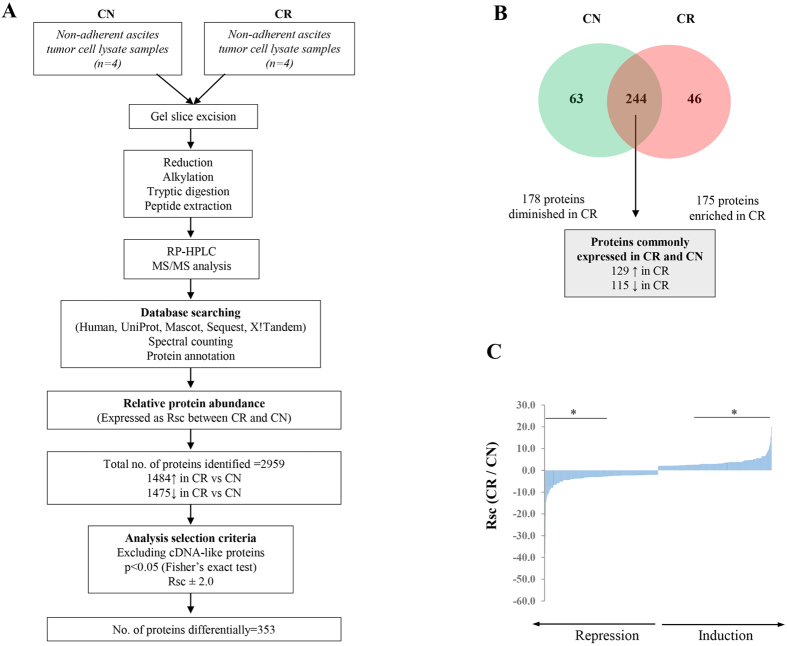
Work flow of proteomic profiling of ascites-derived CN and CR tumor cells and summary of differentially expressed proteins. (**A**) Workflow for the proteomic profiling ovarian cancer ascites-derived tumor cells. Biological replicates of CN (n = 4) and CR (n = 4) patient ascites-derived tumor cell lysates were separated by 1D-SDS-PAGE (10 μg). Individual gel slices were excised and subjected to in-gel reduction, alkylation, and tryptic digestion. Extracted peptides were separated by reverse phase–high performance liquid chromatography (RP-HPLC), followed by mass spectrometry analysis (technical duplicates), database searching and protein annotation. Normalized differential protein expression (Rsc, ratio of spectral counts) ranked as maximal difference between CR and CN samples. Proteins which met the selection criteria of p < 0.05 (Fisher’s exact test) and Rsc > ± 2 are listed and included for further analysis. (**B**) A two-way Venn diagram summarizing the number of proteins in CN and CR cell lysates which meet the selection criteria (p < 0.05, Rsc ± 2), with contaminants and proteins identified as ‘cDNA-like’ excluded from analysis. In total, 353 proteins were found to meet the selection criteria. Two-hundred and forty-four proteins were found to be commonly identified in both CN and CR samples. Of these, 129 proteins were enriched in CR samples compared to CN, while 115 were diminished in CR compared to CN samples. In all, 178 proteins were found to be diminished in CR samples (enriched in CN) and 175 proteins were enriched in CR samples. (**C**) Differential expression (Rsc) ranked as maximal difference between CR and CN. Proteins induced/repressed in expression are displayed (Rsc ± 2), while proteins with p-values <0.05 are shown (*).

For the purpose of analyzing the significant differences between CN and CR samples, a selection criteria was applied and only proteins which were differentially expressed at p < 0.05, and which exhibited a fold change of >2 were only included in the study. In addition, proteins identified as ‘cDNA-like proteins’ were excluded from the analysis. An outline of the protein selection criteria is described in (Fig. 2A). On this basis, a combined total of 353 proteins were identified to be differentially expressed between CN and CR samples (Fig. 2B,C) (Supplementary Table S3). Of these, 175 proteins were enriched in CR compared to CN samples, and 178 were diminished in CR compared to CN ascites-derived tumor cells. Of the 175 enriched proteins in CR compared to CN samples, 40% of the proteins were expressed in all 4 CR samples (number of peptides for each protein ranged between 222–5), 32% of the proteins in 3 CR samples (number of peptides for each protein ranged between 41–4), 20% in 2 CR samples (number of peptides for each protein ranged between 19–4) and 8% in 1 CR sample (number of peptides for each protein ranged between 20–4). On the other hand, among the 178 diminished proteins in CR samples compared to CN samples 12% of the proteins were expressed in 4 CR samples (number of peptides for each protein ranged between 246–4), 18% in 3 CR samples (number of peptides for each protein ranged between 48–3), 12% in 2 CR samples (number of peptides for each protein ranged between 22–2) and 25% in 1 CR sample (number of peptides for each protein ranged between 26–1). The remaining 37% of proteins were unique to CN samples and were not expressed in CR samples.

The top 10 proteins unique to CN samples (Fig. 2B, Supplementary Table S4) were MHC class 1 antigen (HLA-B) [Rsc = −54.6], EVPL protein [Rsc = −38.1], MHC class 1 antigen fragment (HLA-C) [Rsc = −30.9], interferon induced GTP binding protein (MX2) [Rsc = −20.9], Hematopoietic Cell kinase (HCK) [Rsc = −14.5], Erlin-1(ERLIN1) [Rsc = −14.5], spectrin alpha chain (SPTA1) [Rsc = −12.3], protein AHNAK2 [Rsc = −11.6], Lymphocyte antigen 75 (Ly-75) [Rsc = −10.9] and unconventional myosin Vb (MYO5B) [Rsc = −10.9] (Table 1), Other CN unique proteins of interest commonly expressed in carcinomas were Indolamine 2,3 dioxygenase (IDO1) [Rsc = −10.2], Protein arginine deiminase (PADI2) [Rsc = −8.8], Prostaglandin G/H synthase 2 (PTGS2/ COX-2) [Rsc = −8.8], Kynureninase [Rsc = −8.2] and Urokinase-type plasminogen activator chain B (PLAU) [Rsc = −6.6], Mucin-4 beta chain (MUC4) [Rsc = −5.9] (Supplementary Table S4).

The top ten proteins unique to CR tumor cells (Fig. 2B, Supplementary Table S5) were glycine decarboxylase (GLDC) [Rsc = 19.9], glutathione S-transferase Mu 3 (GST-M3) [Rsc = 19.9], MHC class II antigen (HLA-DRB1) [Rsc = 15.5] , acetyl-CoA carboxylase 1 (ACACA) [Rsc = 12.8], Histidyl-tRNA synthetase (HRS) [Rsc = 11.9], Rab3 GTPase-activating protein catalytic subunit (RAB3GAP1) [Rsc = 11.0], cyclin-dependent kinase inhibitor 2 A (CDKN2A) [Rsc = 9.2], DNA mismatch repair protein (MSH6) [Rsc = 9.2], condensin complex subunit 1 (NACAPD2) {Rsc = 9.2], ubiquitin carboxyl-terminal hydrolase isozyme L1 (UCHL1) [Rsc = 8.3] (Table 2). Other CR unique proteins which commonly are reported to be associated with the progression and chemoresistance of ovarian carcinomas were Aurora kinase A and B (AURKA and AURKB) [Rsc = 5.6], metastasis associated protein-1 (MTA-1) [Rsc = 5.6], etc. In addition, C-reactive protein (CRP) [Rsc = 4.7] which is commonly elevated in the serum of ovarian cancer patients was also unique to CR tumor population and was not expressed in CN ascites-derived tumor cells (Supplementary Table S5).

Of the proteins identified, 244 were seen to be commonly expressed in both CN and CR ascites-derived tumor populations (Fig. 2B, Supplementary Table S6). One hundred and twenty nine common proteins were seen to be enriched in CR samples compared to CN samples, while 115 proteins were identified to be diminished in CR versus CN samples. Of the top 10 common proteins identified to be most enriched in CR samples included collagen alpha-1(XII) (COL12A) [Rsc = 12.1] which topped the list (Table 3). This was followed by Asparagine synthetase (ASNS) [Rsc = 10.6], Squalene synthase (FDFT1) [Rsc = 6.6], UDP-glucose 6-dehydrogenase (UGDH) [Rsc = 6.4], Pyrroline-5-carboxylate reductase 2 (PYCR2) [Rsc = 5.8], Cleavage and polyadenylation specific factor subunit 3 (CPSF3) [Rsc = 5.6], Phosphoserine aminotransferase, FAD synthase (FLAD1) [Rsc = 5.3], laminin subunit alpha-5 (LAMA5) [Rsc = 5.1], Methyl transferase-like protein 7B (METTL7B) [Rsc = 5.1]. Other proteins significantly enriched in CR included DNA Topoisomerase 2-alpha (TOP2A) [Rsc = 3.7], Agrin (AGRN) [Rsc = 3.1], Propionyl CoA carboxylase (PCCB), Protein DJ-1 {Rsc = 3.0]{Cao, 2015 #78}, Perilipin [Rsc = 3.0], Elongation factor 1-alpha 1 (EEF1A1), Macrophage migration inhibitory factor (MIF) [Rsc = 2.5], Signal transducer and activation of transcription (STAT3)[Rsc = 2.2], Transferrin (TF) [Rsc = 2.0], Proliferating cell nuclear antigen (PCNA) [Rsc = 2.0], A disintegrin and metalloproteinase with thrombospondin motifs 1 (ADAM-TS 1) [Rsc = 2.1], Multidrug resistance associated protein 4 (ATP binding cassette sub family C member 4) (ABCC4) and others (Supplementary Table S6). In addition, several proteins which previously have been shown to be elevated in the serum of ovarian cancer patients such as Chitinase-3-like protein 1(CHI3L1) [Rsc = 4.5], Haptoglobin (HP) [Rsc = 4.0] and Alpha-2-macroglobulin (A2M) [Rsc = 2.5] were elevated in CR.

The common protein most diminished in CR compared to CN samples was Dehydrogenase/reductase SDR family member 9 (DHRS9) [Rsc = −13.0] (Table 4, Supplementary Table S6). This was followed by Prostacyclin synthase (PTGIS) [Rsc = −8.1], endothelin-converting enzyme 1(ECE1) [Rsc = −6.7], Periplakin (PPL) [Rsc = −6.4], high mobility group protein B2 (HMG-B2) [Rsc = −5.7], interferon induced protein (IFIT2) [Rsc = −5.7], laminin subunit gamma-2 (LAMC2) [Rsc = 5.3], hepatoma derived growth factor (HDGF) [Rsc = −5.1], carnitine O-acetyltransferase (CRAT) [rsc = −4.9] and melanotransferrin (melanoma associated antigen p97, CD228, MFI2) [Rsc = −4.9]. Other common diminished proteins of interest in CR were Placental Alkaline phosphatase (ALPP) [Rsc = −4.3], Calcium and integrin binding protein −1 (CIB-1) [Rsc = −4.2], Plectin (PLEC) [Rsc = −4.1], Ankyrin 1(ANK1) [Rsc = −4.2], Annexin-9 (ANXA9) [Rsc = −3.9], Transaldolase (TAlDO1) [Rsc = −3.7], integrin beta 8 (ITGB8) [Rsc = −3.3], Collagen alpha 1 (COL6A1) [Rsc = −3.0], and Epiplakin (EPPK1) [Rsc = −3.0] and others (Supplementary Table S6).

### Validation of candidate proteins by Western blot

**Figure 3:**
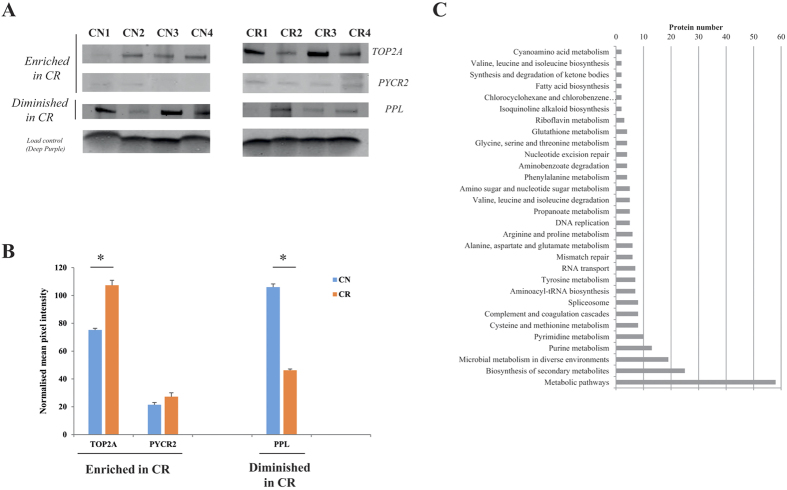
Validation of differentially regulated proteins between CN and CR non-adherent cellular-derived patient samples. **(A)** Representative Western blots of cellular proteins derived from non-adherent spheroid ascites tumor populations from CN (4 individual patient replicates) and CR (4 individual patient replicates) samples. Proteins examined are TOP2A, PYCR2 and PPL. Total protein load was obtained by staining the membrane with Deep Purple. **(B)** Densitometry analysis was performed using ImageStudio v5, with mean pixel intensity normalized to background. Data is expressed as mean ± SEM of n = 4 samples/group performed in triplicate. Significance was determined using Student’s t-test with *p < 0.05 considered statistically significant **(C)** KEGG pathway enrichment analysis of proteins identified enriched in CR. Proteins identified enriched in CR vs CN (based on label-free spectral counting, Rsc > 2, Fisher’s exact test <0.05) were functionally annotated to identify enriched biological pathways using the KEGG database. Only significantly enriched KEGG functional categories (p < 0.05) are depicted according to their protein number. The analysis yielded a strong significance for proteins associated with ‘energy metabolism’ and ‘DNA mismatch repair mechanisms’ pathways.

To confirm expression of selected proteins from proteomic profiling between the CN and CR populations, validation of a subset of proteins was carried out using immunoblotting (Fig. 3A,B). The proteins selected have been shown previously to be expressed in high-grade ovarian tumors and/or to have a role in drug-resistance. For Western blot analysis we examined the expression (in three independent experiments) of TOP2A, PYCR2 and PPL on the CN and CR samples used for proteomic analysis. Based on these results, the expression of TOP2A was significantly greater in CR than CN tumor cells (approx 1.5 fold) (Fig. 3A,B). This elevated expression was consistent with the proteomic profiling analysis, in which the Rsc (CR/CN) value of TOP2A was 3.7 fold higher in CR compared to CN tumor cells (Supplementary Tables S2 and S6). Although proteomic profiling analysis identified a significant enrichment of PYCR2 in CR samples compared to CN samples, the increase in protein expression was not deemed significant by Western blot (1.2 fold change). This may be due to weak signal of the PYCR2 antibody used against the target protein (PYCR2) in Western blot analysis which did not exactly correlate with the more sensitive mass spectrometry-based proteomic profiling. PPL which was found to be diminished in CR (Rsc = −6.4), (Supplementary Tables S2 and 6) by proteomic profiling and showed a similar pattern by Western blot (−2.2- fold change) (Fig. 3A,B).

### Proteome data enrichment and pathway analysis

We subjected the proteome profiles of proteins from CN and CR population to analyses by KEGG, DAVID, and Gene Ontology (Fig. 3C). Several clusters of interacting proteins were significantly (p < 0.05) enriched in CR compared to CN tumor cells. Among these, pathways associated with energy metabolism such as glycine/serine/proline and arginine as well as DNA mismatch repair mechanisms were enriched in CR compared to the CN population. In contrast, certain components of proteins involved with cell-cell adhesion and junction formation were significantly diminished in CR compared to CN tumor cells.

## Discussion

The successful clinical outcomes in terms of long-term recurrence free survival and >5 year overall survival in ovarian cancer patients are rare. Hence, there is an urgent need for investigations into the traits of chemoresistant cells which are associated with recurrence with the aim of designing therapies which would specifically target the identified ‘chemoresistant traits’. We hypothesize that recurrence in ovarian cancer patients is largely dictated by the tumor cells that survive chemotherapy treatments, and a comparative study at the protein level of populations of cells isolated from the ascites of CN and CR ovarian cancer patients represents an important missing link to the recurrent disease. With this goal in mind, label-free quantitative proteome profiling was performed for the first time on a set of isolated tumor cells obtained from the ascites of advanced-stage CN and CR serous ovarian cancer patients in order to identify and understand the CR-associated proteins and pathways that could be involved in recurrence and subsequent progression of the disease.

Among the proteins identified, approximately 20% and 15% of the proteins were unique to CN and CR samples, respectively. Among the unique proteins, there was a distinct difference in the expression of host immune associated MHC class antigens which has not previously been reported. While MHC class II antigen (HLA-DRB1) expression was unique for the CR population, the CN population on the other hand, expressed MHC class I antigen (HLA-C). This distinct difference in the expression of MHC class I and II molecules in the CN and CR populations may suggest distinct mechanisms of tumor antigen processing by the host immune system before and after cancer treatment. While the MHC class I molecules initiate natural adaptive immunity and binds and presents peptide fragments to cytotoxic CD8^+^ T cells, the adaptive humoral and cell-mediated immune responses stimulated by MHC class II molecules is orchestrated by CD4^+^ T helper cells. This MHC-dependent shift in the adaptive immune response after chemotherapy treatment may occur paradoxically as a consequence of genotoxic stress following DNA damage response^22^. This would result in the change of a whole range of cytokine/chemokine expression and signalling proteins/pathways and needs detailed further investigation. A recent global gene expression study on ovarian serous carcinomas representing low and high infiltration of cytotoxic T cells has identified cytotoxic T lymphocyte associated genes and pathways which showed strong immunohistochemical correlation with both MHC class I and II membrane expression, parts of the antigen processing and presentation pathways and cytotoxic T lymphocyte (CTL) recruitment^23^.

Consistent with the unique expression of MHC class I antigens in the CN population, we report unique expression of the IFNγ induced protein, Mx2, which previously has been associated with cytokine/chemokine associated immune responses in viral transfected cells^24^. We also report the unique expression of HCK, a member of the SRC family of cytoplasmic tyrosine kinases (SFKs) involved with immune cell migration/invasion and macrophage polarization to activated phenotype^25^ in CN samples. In addition, IDO1, a rate limiting enzyme which regulates the metabolism of essential amino acid tryptophan was unique to the CN population. IDO1 has previously been associated with the suppression of CD8^+^ and CD4^+^ T cells^26^ and has been shown to be involved with the peritoneal dissemination of ovarian cancer through the inhibition of natural killer cell function^27^. IDO1 has also been identified as a marker for poor prognosis in serous ovarian cancer patients^28^. The expression of IDO1 by CN cells was consistent with the unique expression of kynureninase which acts on the accumulated tryptophan metabolite kynurenine and has been shown to be essential for stem cell biology and inflammation associated cancers^29^.

Contrary to the above findings, an autocrine cytokine/JAK/STAT signalling has been shown to induce kynurenine synthesis in multidrug resistant human cancer cells^30^. In this context, we have recently reported significantly enhanced activation of the JAK2/STAT3 pathway in response to chemotherapy treatment in ovarian cancer cells *in vitro* and in *in vivo* mouse xenograft models^31^,^32^. We have also reported enhanced activation of STAT3 in isolated ascites-derived CR tumor cells compared to CN tumor cells^33^ and enhanced gene expression of STAT3 in CR tumor cells compared to CN tumors^34^. This is consistent with the significantly enhanced expression of STAT3 in CR tumors reported in this study.

Recent studies have led to an appreciation of the importance of metabolic reprogramming in cancer cells^35^. Cancer cells (including those from ovarian tumors) possess a fundamentally altered metabolism that enables tumorigenicity^36^. Besides studies on tumor-associated glycolysis, research into glycine^37^, proline^38^, asparagine^39^, tryptophan^30^, citrulline^40^, lipid^41^ and cholesterol metabolism are gaining momentum in cancer biology. Alterations in the metabolism of amino acids and lipids have been shown to contribute to drug-resistance in ovarian cancer cells^36^. The expression of GLDC which is unique to the CR population was shown to induce dramatic changes in glycolysis, glycine/serine and pyrimidine metabolism, regulate cancer cell proliferation and was shown to be essential for the sustenance of tumor initiating non-small cell lung cancer^37^. Even though the expression of this enzyme has not been previously demonstrated in ovarian cancer, aberrant up regulation of GLDC expression was significantly associated with the poor mortality in lung cancer and has been observed in multiple cancer types^37^. In addition, the expression of lipogenic enzyme ACC which previously has been shown to be highly expressed in some primary tumors^41^ was also unique to CR population. FDFT1 is another up regulated protein in the CR cohort which plays an important role in the cholesterol biosynthesis pathway^42^. Compared to non-cancerous tissues, prostate cancer specimens were shown to have enhanced expression of FDFT1^43^ where it mediated the synthesis of the cholesterol content of membrane lipid rafts^44^. In addition, FDFT1 induced tumor necrosis factor receptor 1 enrichment in lipid rafts promoted lung cancer metastasis^45^. These findings underscore the importance of *de novo* cholesterol biosynthesis in ascites-derived CR tumor cells and suggest that FDFT1 may be a potential target for overcoming chemoresistance.

Besides cholesterol biosynthesis, elevated production of the glycosaminoglycan, hyaluronic acid has been strongly implicated in tumorigenesis and drug resistant ovarian cancer^46^. Hence, it is not surprising that UGDH, which is involved in the synthesis of hyaluronan precursor UDP-glucuronic acid, is elevated several-fold in CR tumor cells. In addition, we report a significantly increased expression of PYCR2, a cytosolic enzyme crucial for the metabolism of proline under conditions of nutrient stress^38^. Metabolism of proline which is found abundantly in collagens associated with the connective tissues is an energy source which provides carbon for tricarboxylic cycle and also participates in proline cycle^38^. In addition, ASNS, up regulated in CR, catalyses the conversion of aspartate and glutamine to asparagine and glutamate in an ATP-dependent manner. ASNS shown to be up regulated in several solid tumors and acute lymphoblastic leukaemia is a stress-regulated enzyme enhanced as a result of tumor adaptation to nutrient deprivation and/or hypoxia^47^. ASNS has recently been identified as a new predictive biomarker for ovarian cancer treatment which can be used for L-Asparaginase therapy in a low-ASNS subset of ovarian cancer^40^.

We also report the expression of GST-M3 unique to CR population. GSTs comprise a family of Phase II drug metabolising enzymes which catalyse the conjugation of reduced glutathione to reactive electrophile as part of cellular adaptive response to chemicals and oxidative stress^48^. Hence, the unique expression of GST-M3 in CR ascites-derived cells may imitate the chemoresistant phenotype, which is also consistent with the unique expression of MSH6, mutations of which have been reported in hereditary ovarian cancer^49^. Even though the expression of UCHL1 and CDKN2A were observed in CR population it is not clear if these proteins were epigenetically silent as both these proteins have been shown to act as putative tumor suppressors in ovarian cancer cells^50^,^51^.

In addition, CR tumors were also enriched in ABCC4 proteins^52^ and TOP2α, which is associated with drug resistance [Rsc = 3.7]^53^. Other proteins of interest were Protein DJ-1, shown to be crucial for the peritoneal metastasis of gastric cancer^54^, Agrin, a proteoglycan, which has been shown to play an oncogenic role in hepatocellular carcinoma^55^ and previously identified in ascites of ovarian cancer patients^56^, and the inflammatory cytokine MIF, shown to contribute to immune escape of ovarian cancer patients by down regulating NK cell receptor NKG2D^57^. Enhanced expression of MIF has previously been reported in the serum of ovarian cancer patients and correlated with poor patient prognosis^58^. In addition, enhanced expression of haptoglobin, transferrin and Chitinase-3-like protein 1 (CHI3L1) in CR ascites tumor cells have been reported previously in malignant ascites^59^.

Other proteins of specific interest down regulated in CR tumor cells were members of plakin family such as PPL, EVPL and Plectin which function as ‘molecular bridges’ linking the intracellular cytoskeleton and cell-cell junctions^60^. PPL, EVPL and Plectin interact with each other and participate in the assembly of intermediate filament organisation (such as keratins and vimentins) in epithelial cells^60^,^61^. Loss of PPL expression has been documented in urethelial and esophageal carcinomas^62^ and aberrant DNA hypermethylation of PPL and interacting EVPL have been reported to be responsible for this down regulation in squamous cell carcinomas^63^. PPL knockdown has been associated with reduced cellular movement and ECM adhesion in malignant pharyngeal cells^64^. On the other hand, significantly increased expression of Plectin compared to non-cancerous tissues has been reported in invasive head and neck squamous cell carcinomas and that has been correlated with poor prognosis of the patients^65^. This is consistent with the role of Plectin in association with vimentin in providing a scaffold for invadopodia, facilitating cancer cell invasion and metastasis^66^. In addition, in pancreatic cancer unexpected extracellular surface expression of Plectin has recently been reported and this was shown to be necessary for exosome production in conjunction with proteins Rab27a and −b and required for sustaining tumor growth in immunocompetent mouse models^67^. Our study also identified significantly high expression of Rab27a and –b in CN samples compared to CR samples (Rsc = −3.5 and −4.1 respectively).

**Figure 4:**
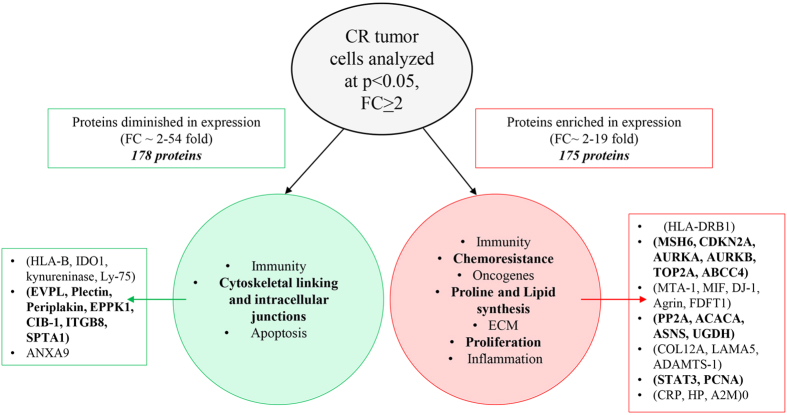
Differential distribution of proteins in the isolated CR compared to CN tumor cells obtained from the ascites of ovarian cancer patients. Cellular functions were assigned to proteins which were evaluated at p < 0.05 and Rsc > ± 2.

In summary, we demonstrate for the first time a novel pattern of protein expression in cells isolated from CR tumors compared to CN tumors obtained from the ascites of ovarian cancer patients. This preliminary profiling-based study utilised a systematic approach of studying only the ascites-derived tumor cells before and after chemotherapy treatments without any background influence from other interacting cells in the tumor microenvironment. An underlying dependence of CR tumor cells for energy metabolism pathways such as enhanced expression of GLDC, ACC, ASNS, FDFT1, UGDH, PYCR2, etc was noted. This may suggest a shift of glucose-dependent mitochondrial function for energy generation for the biosynthesis of secondary metabolites such as glycine/serine, fatty acid, aspartate, cholesterol, hyaluronic acid, proline, which may be involved with the survival of anchorage independent free-floating, genotoxic-stressed, chemoresistant tumor cells. In that setting, concomitant enrichment of proteins involved with DNA repair (MSH6, TOP2A, CDKN2A, AURKA, AURKB) and a member of the ATP-binding cassette transporters (ABCC4) is consistent with the chemoresistant phenotype of CR tumors. Moreover, novel expression of oncogenic Protein DJ-1 previously implicated with the peritoneal dissemination of gastric cancer, makes these findings worthy of future investigation into the role of these proteins in CR biology. Importantly, this study identified a distinct host immune surveillance processes operated by two distinct adaptive immune responses MCH-Class 1 and Class 11 molecules in CN and CR patients respectively. In addition, down regulation of proteins associated with hemidesmosomes and cell cytoskeletal adhesion, such as proteins of the plakin family such as PPL, EVPL, Epiplakin, Plectin etc defines the true free-floating characteristics of CR tumors in the ascites microenvironment. This preliminary study builds the framework for future studies, which should focus on particular proteins and pathways of interest that may have therapeutic potential in reducing ascites-associated chemoresistance and recurrences in ovarian cancer patients. Differential distribution of proteins in the isolated CR compared to CN tumor cells obtained from the ascites of ovarian cancer patients are depicted in Fig. 4.

## Additional Information

**How to cite this article**: Ahmed, N. *et al.* Unique proteome signature of post-chemotherapy ovarian cancer ascites-derived tumor cells. *Sci. Rep.* **6**, 30061; doi: 10.1038/srep30061 (2016).

## Supplementary Material

Supplementary Information

## Figures and Tables

**Table 1:** Top 10 proteins unique to CN ascites tumor cells.

Protein Accession	Gene symbol	Protein Description	CN	CR	R_SC_	Gene ontology (GO)
Q860I7	HLA-B	MHC class I antigen	75	0	−54.5	MHC class I protein complex; antigen processing and presentation of peptide antigen via MHC class I; immune response; integral component of membrane; peptide antigen binding; positive regulation of T cell mediated cytotoxicity
B7ZLH8	EVPL	EVPL protein	52	0	−38.1	cytoskeleton
I6NXG5	HLA-C	MHC class I antigen	42	0	−30.9	MHC class I protein complex; antigen processing and presentation of peptide antigen via MHC class I; immune response; peptide antigen binding; positive regulation of T cell mediated cytotoxicity
P20592	MX2	Interferon-induced GTP-binding protein Mx2	28	0	−20.9	GTP binding; GTPase activity; cytosol; defense response to virus; mRNA transport; nuclear pore; protein transport; regulation of cell cycle; regulation of nucleocytoplasmic transport; response to interferon-alpha; type I interferon signaling pathway
H0Y3C5	HCK	Tyrosine-protein kinase HCK	19	0	−14.5	NA
B0QZ43	ERLIN1	Erlin-1 (Fragment)	19	0	−14.5	ATP binding; peptidyl-tyrosine phosphorylation; protein tyrosine kinase activity
P02549	SPTA1	Spectrin alpha chain, erythrocytic 1 (Erythroid alpha-spectrin)	16	0	−12.3	actin filament binding; actin filament capping; actin filament organization; axon guidance; calcium ion binding; cuticular plate; cytosol; hemopoiesis; intrinsic component of the cytoplasmic side of the plasma membrane; lymphocyte homeostasis; plasma membrane organization; porphyrin-containing compound biosynthetic process; positive regulation of T cell proliferation; positive regulation of protein binding; regulation of cell shape; spectrin; spectrin-associated cytoskeleton; structural constituent of cytoskeleton
Q8IVF2	AHNAK2	Protein AHNAK2	15	0	−11.6	nucleus
O60449	LY75	Lymphocyte antigen 75 (CD antigen CD205)	14	0	−10.9	carbohydrate binding; endocytosis; immune response; inflammatory response; integral component of plasma membrane; receptor activity
Q9ULV0	MYO5B	Unconventional myosin-Vb	14	0	−10.9	ATP binding; cytoplasmic vesicle membrane; microfilament motor activity; myosin complex; protein complex; protein transport; transmembrane transport; vesicle-mediated transport; water transport

Rsc: Spectral count ratio (CR /CN), NA: Not available.

**Table 2:** Top 10 proteins unique to CR ascites tumor cells.

Protein Accession	Gene symbol	Protein Description	CN	CR	R_SC_	Gene ontology (GO)
P23378	GLDC	Glycine dehydrogenase [decarboxylating], mitochondrial (EC 1.4.4.2)	0	21	19.9	electron carrier activity; glycine catabolic process; glycine dehydrogenase (decarboxylating) activity; lyase activity; mitochondrion; pyridoxal phosphate binding
P21266	GSTM3	Glutathione S-transferase Mu 3 (EC 2.5.1.18)	0	21	19.9	cellular detoxification of nitrogen compound; cytosol; establishment of blood-nerve barrier; glutathione binding; glutathione derivative biosynthetic process; glutathione metabolic process; glutathione transferase activity; nitrobenzene metabolic process; protein homodimerization activity; response to estrogen; xenobiotic catabolic process
Q56FN6	HLA-DRB1	MHC class II antigen	0	16	15.5	MHC class II protein complex; antigen processing and presentation of peptide or polysaccharide antigen via MHC class II; immune response; integral component of membrane
Q13085	ACACA	Acetyl-CoA carboxylase 1 (EC 6.4.1.2)	0	13	12.8	ATP binding; acetyl-CoA carboxylase activity; acetyl-CoA metabolic process; biotin carboxylase activity; biotin metabolic process; carnitine shuttle; cytosol; energy reserve metabolic process; fatty acid biosynthetic process; lipid homeostasis; long-chain fatty-acyl-CoA biosynthetic process; malonyl-CoA biosynthetic process; metal ion binding; mitochondrion; multicellular organismal protein metabolic process; positive regulation of cellular metabolic process; protein homotetramerization; tissue homeostasis; triglyceride biosynthetic process
Q52NV4	HRS	Histidyl-tRNA synthetase	0	12	11.9	ATP binding; cytoplasm; histidine-tRNA ligase activity; histidyl-tRNA aminoacylation
C9J837	RAB3GAP1	Rab3 GTPase-activating protein catalytic subunit	0	11	11.0	Rab GTPase activator activity; centrosome; cytoplasm; nucleus; positive regulation of Rab GTPase activity
P42771	CDKN2A	Cyclin-dependent kinase inhibitor 2A, isoforms 1/2/3	0	9	9.2	G1/S transition of mitotic cell cycle; NF-kappaB binding; Ras protein signal transduction; cell cycle arrest; cell cycle checkpoint; cyclin-dependent protein serine/threonine kinase inhibitor activity; cytosol; negative regulation of NF-kappaB transcription factor activity; negative regulation of cell growth; negative regulation of cell proliferation; negative regulation of cell-matrix adhesion; negative regulation of cyclin-dependent protein serine/threonine kinase activity; negative regulation of transcription, positive regulation of cellular senescence; positive regulation of macrophage apoptotic process; positive regulation of smooth muscle cell apoptotic process; replicative senescence; senescence-associated heterochromatin focus; senescence-associated heterochromatin focus assembly
B4DF41	MSH6	DNA mismatch repair protein Msh6	0	9	9.2	ATP binding; mismatch repair; mismatched DNA binding
B3KMS0	NACAPD2	Condensin complex subunit 1	0	9	9.2	mitotic chromosome condensation; nucleus
D6RE83	UCHL1	Ubiquitin carboxyl-terminal hydrolase isozyme L1	0	8	8.3	cytoplasm; nucleus; ubiquitin thiolesterase activity; ubiquitin-dependent protein catabolic process

Rsc: Spectral count ratio (CR /CN).

**Table 3:** Top 10 enriched proteins identified in CR compared to CN ascites tumor cells.

Protein Accession	Gene symbol	Protein Description	CN	CR	R_SC_	Gene ontology (GO)
Q99715	COL12A1	Collagen alpha-1 (XII) chain	1	23	12.1	cell adhesion; collagen catabolic process; collagen fibril organization; collagen type XII; endoplasmic reticulum lumen; extracellular matrix disassembly; extracellular matrix structural constituent conferring tensile strength; extracellular space; extracellular vesicular exosome; skeletal system development
P08243	ASNS	Asparagine synthetase [glutamine-hydrolyzing]	1	20	10.6	ATP binding; L-asparagine biosynthetic process; activation of signaling protein activity involved in unfolded protein response; asparagine biosynthetic process; asparagine synthase (glutamine-hydrolyzing) activity; cellular protein metabolic process; cellular response to glucose starvation; cellular response to hormone stimulus; cofactor binding; cytosol; glutamine metabolic process; liver development; negative regulation of apoptotic process; positive regulation of mitotic cell cycle; response to amino acid; response to follicle-stimulating hormone; response to light stimulus; response to mechanical stimulus; response to methotrexate; response to toxic substance
P37268	FDFT1	Squalene synthase (Farnesyl-diphosphate farnesyltransferase)	1	12	6.6	cellular lipid metabolic process; cholesterol biosynthetic process; endoplasmic reticulum membrane; farnesyl diphosphate metabolic process; farnesyl-diphosphate farnesyltransferase activity; integral component of membrane; isoprenoid biosynthetic process; oxidoreductase activity; squalene synthase activity
O60701	UGDH	UDP-glucose 6-dehydrogenase	3	23	6.4	NAD binding; UDP-glucose 6-dehydrogenase activity; UDP-glucose metabolic process; UDP-glucuronate biosynthetic process; cellular glucuronidation; cytosol; electron carrier activity; gastrulation with mouth forming second; glycosaminoglycan biosynthetic process; xenobiotic metabolic process
Q96C36	PYCR2	Pyrroline-5-carboxylate reductase 2	4	26	5.8	L-proline biosynthetic process; cytoplasm; pyrroline-5-carboxylate reductase activity
Q9UKF6	CPSF3	Cleavage and polyadenylation specificity factor subunit 3	1	10	5.6	5′-3′ exonuclease activity; RNA binding; endoribonuclease activity; histone mRNA 3’-end processing; mRNA cleavage; mRNA cleavage and polyadenylation specificity factor complex; mRNA export from nucleus; mRNA polyadenylation; mRNA splicing, via spliceosome; metal ion binding; ribonucleoprotein complex; termination of RNA polymerase II transcription
B4DHQ3	PSAT	Phosphoserine aminotransferase	1	10	5.6	L-serine biosynthetic process; O-phospho-L-serine:2-oxoglutarate aminotransferase activity; pyridoxal phosphate binding
Q8NFF5	FLAD1	FAD synthase	3	19	5.3	ATP binding; FAD biosynthetic process; FMN adenylyltransferase activity; Mo-molybdopterin cofactor biosynthetic process; cytosol; mitochondrial matrix; riboflavin metabolic process
O15230	LAMA5	Laminin subunit alpha-5	1	9	5.1	angiogenesis; cell migration; cell proliferation; cell recognition; cilium assembly; cytoskeleton organization; embryo development; endothelial cell differentiation; establishment of protein localization to plasma membrane; extracellular matrix organization; extracellular space; focal adhesion assembly; integrin binding; integrin-mediated signaling pathway; laminin-1 complex; laminin-10 complex; laminin-11 complex; laminin-5 complex; lung development; morphogenesis of a polarized epithelium; morphogenesis of embryonic epithelium; muscle organ development; neural crest cell migration; odontogenesis of dentin-containing tooth; regulation of cell adhesion; regulation of cell migration; regulation of cell proliferation; regulation of embryonic development; structural molecule activity; substrate adhesion-dependent cell spreading
Q6UX53	METTL7B	Methyltransferase-like protein 7B	1	9	5.1	methyltransferase activity

Rsc: Spectral count ratio (CR/CN).

**Table 4:** Top 10 diminished proteins identified in CR compared to CN ascites tumor cells.

Protein Accession	Gene symbol	Protein Description	CN	CR	R_SC_	Gene ontology (GO)
B7Z416	DHRS9	Dehydrogenase/reductase SDR family member 9	48	46	−13.0	oxidation-reduction process; oxidoreductase activity
Q16647	PTGIS	Prostacyclin synthase	43	40	−8.7	apoptotic signaling pathway; cellular response to hypoxia; cellular response to interleukin-1; cellular response to interleukin-6; cyclooxygenase pathway; endoplasmic reticulum membrane; extracellular space; heme binding; integral component of membrane; iron ion binding; monooxygenase activity; negative regulation of NF-kappaB transcription factor activity; negative regulation of inflammatory response; negative regulation of nitric oxide biosynthetic process; nucleus; oxidoreductase activity, acting on paired donors, with incorporation or reduction of molecular oxygen; positive regulation of angiogenesis; positive regulation of execution phase of apoptosis; positive regulation of peroxisome proliferator activated receptor signaling pathway; prostaglandin-I synthase activity; xenobiotic metabolic process
B4DKB2	ECE1	Endothelin-converting enzyme 1	42	38	−6.7	metalloendopeptidase activity; proteolysis
K7EKI8	PPL	Periplakin	211	187	−6.4	cytoskeleton
P26583	HMGB2	High mobility group protein B2	14	13	−5.7	DNA binding, bending; DNA topological change; RAGE receptor binding; apoptotic DNA fragmentation; base-excision repair, DNA ligation; cell chemotaxis; cellular response to lipopolysaccharide; chemoattractant activity; damaged DNA binding; double-stranded DNA binding; extracellular space; male gonad development; negative regulation of extrinsic apoptotic signaling pathway via death domain receptors; negative regulation of transcription, DNA-templated; nucleosome assembly; phosphatidylinositol-mediated signaling; positive regulation of DNA binding; positive regulation of endothelial cell proliferation; positive regulation of erythrocyte differentiation; positive regulation of megakaryocyte differentiation; positive regulation of nuclease activity; positive regulation of transcription from RNA polymerase II promoter; protein complex; response to steroid hormone; sequence-specific DNA binding transcription factor activity; single-stranded DNA binding; transcription regulatory region DNA binding
P09913	IFIT2	Interferon-induced protein with tetratricopeptide repeats 2	14	13	−5.7	RNA binding; apoptotic mitochondrial changes; cellular response to interferon-alpha; cytosol; defense response to virus; endoplasmic reticulum; negative regulation of protein binding; positive regulation of apoptotic process; response to virus; type I interferon signaling pathway
Q13753	LAMC2	Laminin subunit gamma-2	34	30	−5.3	cell adhesion; cell cortex; epidermis development; extracellular matrix organization; extracellular region; extracellular space; hemidesmosome assembly; heparin binding; laminin-2 complex; laminin-5 complex; membrane; perinuclear region of cytoplasm
A8K8G0	HDGF	Hepatoma-derived growth factor	33	29	−5.1	NA
P43155	CRAT	Carnitine O-acetyltransferase	12	11	−4.9	carnitine O-acetyltransferase activity; carnitine metabolic process, CoA-linked; endoplasmic reticulum; fatty acid beta-oxidation using acyl-CoA oxidase; mitochondrial inner membrane; mitochondrion; peroxisomal matrix; transport
P08582	MFI2	Melanotransferrin (Melanoma-associated antigen p97) (CD228)	12	11	−4.9	anchored component of membrane; cellular iron ion homeostasis; extracellular region; ferric iron binding; integral component of plasma membrane; iron ion binding; iron ion transport

Rsc: Spectral count ratio (CR /CN), NA: Not available.
