# Supplementary material for: Unique proteome signature of post-chemotherapy ovarian cancer ascites-derived tumor cells
Source: Sci Rep. 2016 Jul 29;6:30061. doi: 10.1038/srep30061 (PMC4965858; doi:10.1038/srep30061)
Supplement: Supplementary Information [file srep30061-s1.pdf]

Supplementary Information

Unique proteome signature of post-chemotherapy ovarian cancer ascites-derived tumor cells

Nuzhat Ahmed[1,2,3,4], David Greening[5*], Chantel Samardzija[3], Ruth M Escalona[3,4], Maoshan Chen[5], Jock K Findlay[3,4] and George Kannourakis[1,2]*

[1]Fiona Elsey Cancer Research Institute, Ballarat, Victoria 3353, Australia,

[2]Federation University, Ballarat, Victoria 3010, Australia

[3]Department of Obstetrics and Gynaecology, University of Melbourne, Victoria 3052

[4]The Hudson Institute of Medical Research, Victoria 3168, Australia

[5]Department of Biochemistry and Genetics, La Trobe Institute for Molecular Science, La Trobe University, Bundoora, Victoria, 3086, Australia.

Running title: Proteomic profiling of ascites-derived tumor cells

Key words: ovarian carcinoma, tumor cells, ascites, chemoresistance, chemotherapy

*Both authors contributed equally to the study

Corresponding author:

Nuzhat Ahmed, PhD

Fiona Elsey Cancer Research Institute

Suites 23-26, 106-110 Lydiard Street South

Ballarat Technology Central Park

Ballarat 3353, Australia

Email: nuzhata@unimelb.edu.au

Figure S1. Immuno-blot analysis of TOP2A, PYCR2, and PPL expression in tumour cell lysate samples

A

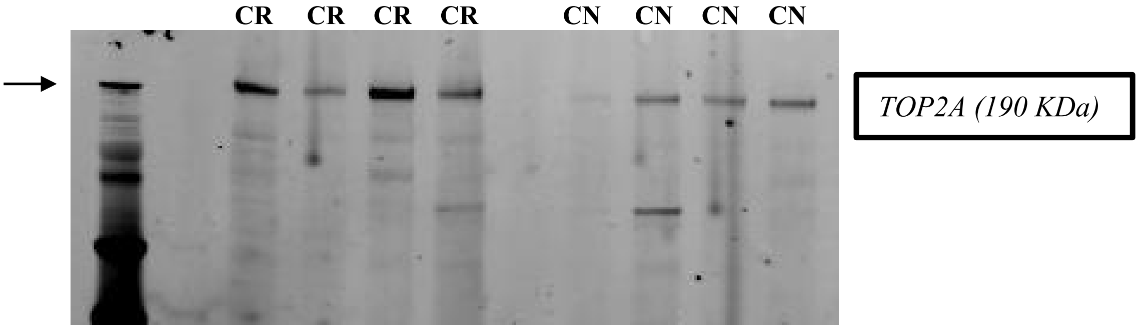

B

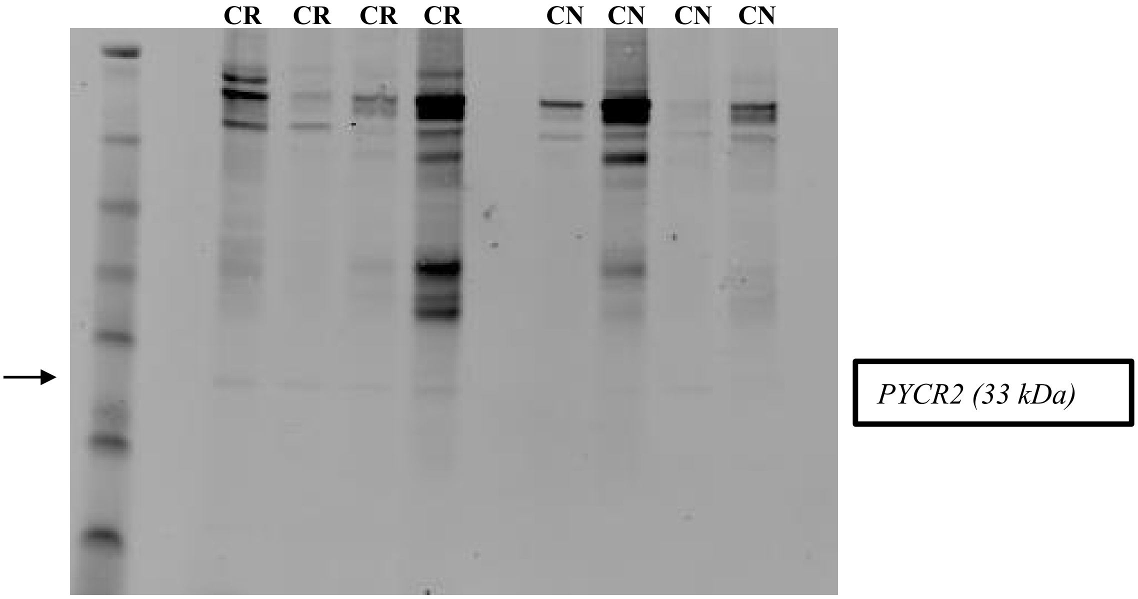

C

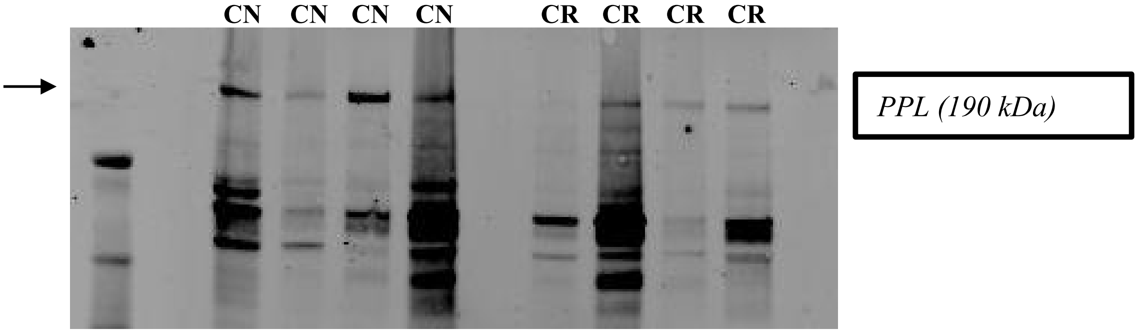

Table S1. Description of patients recruited in the study.

	Patient ID	Sample type	Diagnosis	Silverberg Grade	FIGO Stage	Treatment	Age at diagnosis	Survival	Surivival Status
**Chemonaive**	As59	CN1	Serous Cystadenocarcinoma NOS	G3	IIc	None	64	5 months	Deceased
	As68	CN2	Serous Cystadenoma/ Early Serous Borderline	NA	NA	None	64	NA	NA
	As71	CN3	Serous Cystadenocarcinoma (Genital tract)	NA	III	None	65	2 years, 10 months	Alive
	As77	CN4	Carcinosarcoma NOS	G3	NA	None	53	1 year, 5 months	Deceased
**Chemoreistant (Recurrent)**	As62	CR1	Adenocarcinoma NOS	Not graded	IV	Carboplatin and Paclitaxel 6 cycles	67	2 years, 8 months	Deceased
**Chemoreistant (Recurrent)**	As69	CR2	Serous Cystadenocarcinoma NOS	G3	IIIc	Carboplatin/Paclitaxel/Bevacizumab-VGEF Inhibitor (ICON7 Trial) 18 cycles	55	5 years, 6 months	Deceased
	As72	CR3	Papillary Serous Cystadenocarcinoma	G3	IIIc	Carboplatin and Paclitaxel 6 cycles Gemicitabine and Cisplatin 1 cycle	59	3 years, 2 months	Deceased
	As73	CR4	Serous Cystadenocarcinoma NOS	G3	IIIc	Carboplatin and Paclitaxel 6, AMG-386 182 Trial (Doxorubicin and AMG-386) 9 cycles, Paclitaxel 6 cycles, Cyclophosphamide 2 cycles, Topotecan 2 cycles, Doxorubicin Pegylated Liposomal 2 cycles	53	3years, 1 month	Deceased

*__NOS__: Not otherwise stated
*__NA__: Not available

Table S2. Raw dataset of proteins identified in CN and CR tumor cells.

Gene symbol	UniProt Entry[A]	Gene symbol[A]	Protein Accession[A]	Protein Description[A]	Molecular Weight	%	Combined_ALL[B]	CN_Combined[C]	CR_Combined[D]	CN_1	CN_2	CN_3	CN_4	CR_1	CR_2	CR_3	CR_4	RSC (CR / CN)[E]	p value[F]
PLEC_HUMAN	GCSP_HUMAN	GLDC	P23378	Glycine dehydrogenase [decarboxylating], mitochondrial (EC 1.4.4.2) (Glycine cleavage system P protein) (Glycine decarboxylase)	113 kDa	0.48	21	0	21	0	0	0	0	20	0	0	2	19.9350293	95% (< 0.00010)
GRP78_HUMAN	GSTM3_HUMAN	GSTM3	P21266	Glutathione S-transferase Mu 3 (EC 2.5.1.18) (GST class-mu 3) (GSTM3-3) (hGSTM3-3)	27 kDa	0.59	20	0	21	0	0	0	0	3	14	4	0	19.9350293	95% (< 0.00010)
H4_HUMAN	B4DKU3_HUMAN		B4DKU3	cDNA FLJ53449, highly similar to rRNA methyltransferase 3 (EC 2.1.1.-)	83 kDa	0.59	16	0	16	0	0	0	0	8	2	4	3	15.4541356	95% (< 0.00010)
H2B1D_HUMAN	Q56FN6_HUMAN	HLA-DRB1	Q56FN6	MHC class II antigen	30 kDa	0.61	29	0	16	0	0	0	0	0	9	0	0	15.4541356	95% (< 0.00010)
ANXA2_HUMAN	ACACA_HUMAN	ACACA	Q13085	Acetyl-CoA carboxylase 1 (ACC1) (EC 6.4.1.2) (ACC-alpha) [Includes: Biotin carboxylase (EC 6.3.4.14)]	266 kDa	0.72	12	0	13	0	0	0	0	12	0	0	0	12.76590882	95% (< 0.00010)
CH60_HUMAN	COCA1_HUMAN	COL12A1	Q99715	Collagen alpha-1(XII) chain	333 kDa	0.48	24	1	23	0	0	1	0	12	1	6	4	12.07071565	95% (< 0.00010)
Q53G99_HUMAN	Q52NV4_HUMAN	HRS	Q52NV4	Histidyl-tRNA synthetase	57 kDa	0.52	13	0	12	0	0	0	0	6	0	2	0	11.8698848	95% (0.00012)
AHNK_HUMAN	C9J837_HUMAN	RAB3GAP1	C9J837	Rab3 GTPase-activating protein catalytic subunit	111 kDa	0.28	11	0	11	0	0	0	0	0	9	2	0	10.97388655	95% (0.00025)
ATPB_HUMAN	ASNS_HUMAN	ASNS	P08243	Asparagine synthetase [glutamine-hydrolyzing] (EC 6.3.5.4) (Cell cycle control protein TS11) (Glutamine-dependent asparagine synthetase)	64 kDa	0.42	21	1	20	0	0	1	0	9	10	1	0	10.57697465	95% (< 0.00010)
ENPL_HUMAN (+1)	B2RCG8_HUMAN		B2RCG8	cDNA, FLJ96063, highly similar to Homo sapiens rhophilin, Rho GTPase binding protein 2 (RHPN2), mRNA	77 kDa	0.42	10	0	10	0	0	0	0	2	5	0	3	10.07791409	95% (0.00053)
CLH1_HUMAN	B4DF41_HUMAN	MSH6	B4DF41	DNA mismatch repair protein Msh6 (cDNA FLJ55677, highly similar to DNA mismatch repair protein MSH6)	138 kDa	0.39	9	0	9	0	0	0	0	8	0	1	0	9.181967408	95% (0.0011)
PDIA1_HUMAN	A8K2Q7_HUMAN		A8K2Q7	cDNA FLJ77928, highly similar to Homo sapiens acyl-CoA synthetase medium-chain family member 3 (ACSM3), transcript variant 1, mRNA	66 kDa	0.56	9	0	9	0	0	0	0	9	0	0	0	9.181967408	95% (0.0011)
TBB5_HUMAN	B3KMS0_HUMAN		B3KMS0	Condensin complex subunit 1	157 kDa	0.49	9	0	9	0	0	0	0	8	0	1	0	9.181967408	95% (0.0011)
B7Z4V2_HUMAN (+1)	CD2A1_HUMAN	CDKN2A	P42771	Cyclin-dependent kinase inhibitor 2A, isoforms 1/2/3 (Cyclin-dependent kinase 4 inhibitor A) (CDK4I) (Multiple tumor suppressor 1) (MTS-1) (p16-INK4a) (p16-INK4) (p16INK4A)	17 kDa	0.51	9	0	9	0	0	0	0	3	6	0	0	9.181967408	95% (0.0011)
ATPA_HUMAN	Q86YH3_HUMAN	EARS2	Q86YH3	EARS2 protein	60 kDa	0.47	7	0	8	0	0	0	0	1	0	2	4	8.286046503	95% (0.0024)
B2R5B3_HUMAN (+4)	A8K9U6_HUMAN		A8K9U6	cDNA FLJ76121, highly similar to Homo sapiens zinc finger CCCH-type, antiviral 1 (ZC3HAV1), transcript variant 1, mRNA	101 kDa	0.46	8	0	8	0	0	0	0	2	1	4	0	8.286046503	95% (0.0024)
MYH9_HUMAN	D6RE83_HUMAN	UCHL1	D6RE83	Ubiquitin carboxyl-terminal hydrolase isozyme L1	23 kDa	0.38	8	0	8	0	0	0	0	0	5	3	0	8.286046503	95% (0.0024)

LMNA_HUMAN	B3KUH3_HUMAN		B3KUH3	cDNA FLJ39896 fis, clone SPLEN2016972	90 kDa	0.59	7	0	7	0	0	0	0	0	0	1	6	7.390151376	95% (0.0051)
C8C504_HUMAN (+1)	CADH6_HUMAN	CDH6	P55285	Cadherin-6 (Kidney cadherin) (K-cadherin)	88 kDa	0.82	7	0	7	0	0	0	0	4	0	1	2	7.390151376	95% (0.0051)
IDHP_HUMAN	AK1C1_HUMAN	AKR1C1	Q04828	Aldo-keto reductase family 1 member C1 (EC 1.1.1.-) (20-alpha-hydroxysteroid dehydrogenase) (20-alpha-HSD) (EC 1.1.1.149) (Chlordecone reductase homolog HAKRC) (Dihydrodiol dehydrogenase 1/2) (DD1/DD2) (High-affinity hepatic bile acid-binding protein) (HBAB) (Indanol dehydrogenase) (EC 1.1.1.112) (Trans-1,2-dihydrobenzene-1,2-diol dehydrogenase) (EC 1.3.1.20)	37 kDa	0.56	7	0	7	0	0	0	0	0	6	1	0	7.390151376	95% (0.0051)
LPPRC_HUMAN	A8K5Y7_HUMAN		A8K5Y7	cDNA FLJ78655, highly similar to Homo sapiens exportin 5 (XPO5), mRNA	136 kDa	0.5	22	2	20	0	0	0	2	3	11	6	0	7.322426833	95% (< 0.00010)
B3KQT9_HUMAN (+2)	FDFT_HUMAN	FDFT1	P37268	Squalene synthase (SQS) (SS) (EC 2.5.1.21) (FPP:FPP farnesyltransferase) (Farnesyl-diphosphate farnesyltransferase)	48 kDa	0.53	13	1	12	1	0	0	0	9	1	2	0	6.594295719	95% (0.00087)
HSP7C_HUMAN	A8KAQ3_HUMAN		A8KAQ3	cDNA FLJ76489, highly similar to Homo sapiens sortilin 1 (SORT1), mRNA	92 kDa	0.45	13	1	12	0	0	0	1	3	3	1	5	6.594295719	95% (0.00087)
MYOF_HUMAN	TRIPC_HUMAN	TRIP12	Q14669	E3 ubiquitin-protein ligase TRIP12 (EC 6.3.2.-) (E3 ubiquitin-protein ligase for Arf) (ULF) (Thyroid receptor-interacting protein 12) (TR-interacting protein 12) (TRIP-12)	220 kDa	0.43	6	0	6	0	0	0	0	2	0	4	0	6.494282025	95% (0.011)
KPYM_HUMAN	A8K984_HUMAN		A8K984	Structural maintenance of chromosomes protein	136 kDa	0.57	6	0	6	0	0	0	0	4	0	1	0	6.494282025	95% (0.011)
PRKDC_HUMAN	A8K5B6_HUMAN		A8K5B6	cDNA FLJ76830, highly similar to Homo sapiens SWI/SNF related, matrix associated, actin dependent regulator of chromatin, subfamily a, member 3 (SMARCA3), transcript variant 1, mRNA	114 kDa	0.26	6	0	6	0	0	0	0	6	0	0	0	6.494282025	95% (0.011)
HS90B_HUMAN	D3DNT9_HUMAN	EPHB3	D3DNT9	EPH receptor B3, isoform CRA_a	106 kDa	0.45	6	0	6	0	0	0	0	4	0	2	0	6.494282025	95% (0.011)
B4DMF5_HUMAN (+2)	B4DKC8_HUMAN		B4DKC8	cDNA FLJ56098, highly similar to Sodium bicarbonate transporter-like protein11	103 kDa	0.47	6	0	6	0	0	0	0	2	0	0	4	6.494282025	95% (0.011)
ADT2_HUMAN	A8K245_HUMAN		A8K245	cDNA FLJ75441, highly similar to Homo sapiens vaccinia related kinase 1 (VRK1), mRNA	45 kDa	0.62	6	0	6	0	0	0	0	2	0	4	0	6.494282025	95% (0.011)
FLNA_HUMAN (+1)	ARL3_HUMAN	ARL3	P36405	ADP-ribosylation factor-like protein 3	20 kDa	0.32	6	0	6	0	0	0	0	1	4	1	0	6.494282025	95% (0.011)
MDHM_HUMAN (+2)	A8K6A6_HUMAN		A8K6A6	cDNA FLJ78619, highly similar to Homo sapiens melanoma cell adhesion molecule (MCAM), mRNA	72 kDa	0.63	6	0	6	0	0	0	0	5	1	0	0	6.494282025	95% (0.011)
ECHA_HUMAN	AOFA_HUMAN	MAOA	P21397	Amine oxidase [flavin-containing] A (EC 1.4.3.4) (Monoamine oxidase type A) (MAO-A)	60 kDa	0.39	6	0	6	0	0	0	0	0	3	0	3	6.494282025	95% (0.011)
F5H5D3_HUMAN (+2)	B4DIH5_HUMAN	COPS2	B4DIH5	COP9 signalosome complex subunit 2 (cDNA FLJ52928, highly similar to COP9 signalosome complex subunit 2)	44 kDa	0.37	5	0	6	0	0	0	0	3	2	1	0	6.494282025	95% (0.011)
ANXA1_HUMAN	B4DVV7_HUMAN		B4DVV7	cDNA FLJ51339, highly similar to Methionyl-tRNA synthetase, mitochondrial (EC 6.1.1.10)	58 kDa	0.59	5	0	6	0	0	0	0	2	1	0	2	6.494282025	95% (0.011)

ACTN4_HUMAN	UGDH_HUMAN	UGDH	O60701	UDP-glucose 6-dehydrogenase (UDP-Glc dehydrogenase) (UDP-GlcDH) (UDPGDH) (EC 1.1.1.22)	55 kDa	0.59	26	3	23	1	1	1	0	2	15	6	0	6.390214665	95% (< 0.00010)
B4DJ30_HUMAN (+1)	P5CR2_HUMAN	PYCR2	Q96C36	Pyrroline-5-carboxylate reductase 2 (P5C reductase 2) (P5CR 2) (EC 1.5.1.2)	34 kDa	0.33	29	4	26	0	0	0	3	12	12	12	12	5.813169761	95% (< 0.00010)
ROA2_HUMAN	CPSF3_HUMAN	CPSF3	Q9UKF6	Cleavage and polyadenylation specificity factor subunit 3 (EC 3.1.27.-) (Cleavage and polyadenylation specificity factor 73 kDa subunit) (CPSF 73 kDa subunit) (mRNA 3'-end-processing endonuclease CPSF-73)	77 kDa	0.5	11	1	10	0	0	1	0	1	0	7	2	5.598769229	95% (0.0034)
DYHC1_HUMAN	B4DHQ3_HUMAN		B4DHQ3	Phosphoserine aminotransferase (EC 2.6.1.52)	45 kDa	0.2	11	1	10	0	0	0	1	5	4	1	0	5.598769229	95% (0.0034)
ENOA_HUMAN	K0020_HUMAN	KIAA0020	Q15397	Pumilio domain-containing protein KIAA0020 (HBV X-transactivated gene 5 protein) (HBV XAg-transactivated protein 5) (Minor histocompatibility antigen HA-8) (HLA-HA8)	74 kDa	0.46	5	0	5	0	0	0	0	2	2	1	0	5.59843845	95% (0.023)
B4DGP8_HUMAN (+1)	MAAI_HUMAN	GSTZ1	O43708	Maleylacetoacetate isomerase (MAAI) (EC 5.2.1.2) (GSTZ1-1) (Glutathione S-transferase zeta 1) (EC 2.5.1.18)	24 kDa	0.31	5	0	5	0	0	0	0	2	1	2	0	5.59843845	95% (0.023)
HSP71_HUMAN	UBE2O_HUMAN	UBE2O	Q9C0C9	Ubiquitin-conjugating enzyme E2 O (EC 6.3.2.19) (Ubiquitin carrier protein O) (Ubiquitin-conjugating enzyme E2 of 230 kDa) (Ubiquitin-conjugating enzyme E2-230K) (Ubiquitin-protein ligase O)	141 kDa	0.37	5	0	5	0	0	0	0	0	4	1	0	5.59843845	95% (0.023)
VIME_HUMAN	INT3_HUMAN	INTS3	Q68E01	Integrator complex subunit 3 (Int3) (SOSS complex subunit A) (Sensor of single-strand DNA complex subunit A) (SOSS-A) (Sensor of ssDNA subunit A)	118 kDa	0.56	5	0	5	0	0	0	0	1	1	2	1	5.59843845	95% (0.023)
CATD_HUMAN	AURKB_HUMAN	AURKB	Q96GD4	Aurora kinase B (EC 2.7.11.1) (Aurora 1) (Aurora- and IPL1-like midbody-associated protein 1) (AIM-1) (Aurora/IPL1-related kinase 2) (ARK-2) (Aurora-related kinase 2) (STK-1) (Serine/threonine-protein kinase 12) (Serine/threonine-protein kinase 5) (Serine/threonine-protein kinase aurora-B)	39 kDa	0.41	5	0	5	0	0	0	0	1	0	4	0	5.59843845	95% (0.023)
DHX9_HUMAN	Q59GW6_HUMAN		Q59GW6	Acetyl-CoA acetyltransferase, cytosolic variant (Fragment)	42 kDa	0.29	5	0	5	0	0	0	0	5	0	0	0	5.59843845	95% (0.023)
EF1A1_HUMAN (+5)	B2R6H7_HUMAN		B2R6H7	cDNA, FLJ92955, highly similar to Homo sapiens transportin-SR (TRN-SR), mRNA	104 kDa	0.32	5	0	5	0	0	0	0	3	1	1	0	5.59843845	95% (0.023)
ANXA5_HUMAN	E7ESY4_HUMAN	MTA1	E7ESY4	Metastasis-associated protein MTA1	79 kDa	0.6	8	0	5	0	0	0	0	1	0	3	0	5.59843845	95% (0.023)
A4QPB0_HUMAN (+1)	B7ZMD7_HUMAN	AMY1A	B7ZMD7	Amylase, alpha 1A (Salivary)	58 kDa	0.29	5	0	5	0	0	0	0	0	5	0	0	5.59843845	95% (0.023)
AT1A1_HUMAN	A8K4T2_HUMAN		A8K4T2	cDNA FLJ76281, highly similar to Homo sapiens DnaJ (Hsp40) homolog, subfamily C, member 7 (DNAJC7), mRNA	55 kDa	0.37	5	0	5	0	0	0	0	3	1	1	0	5.59843845	95% (0.023)
TFR1_HUMAN	E9PKF6_HUMAN	PPP6R3	E9PKF6	Serine/threonine-protein phosphatase 6 regulatory subunit 3	94 kDa	0.41	5	0	5	0	0	0	0	0	4	1	0	5.59843845	95% (0.023)
PGK1_HUMAN	A8K559_HUMAN		A8K559	cDNA FLJ76187	51 kDa	0.62	5	0	5	0	0	0	0	0	0	2	3	5.59843845	95% (0.023)
G3P_HUMAN (+1)	PLAP_HUMAN	PLAA	Q9Y263	Phospholipase A-2-activating protein (PLA2P) (PLAP)	87 kDa	0.35	5	0	5	0	0	0	0	2	3	0	0	5.59843845	95% (0.023)
PDIA4_HUMAN	ADHX_HUMAN	ADH5	P11766	Alcohol dehydrogenase class-3 (EC 1.1.1.1) (Alcohol dehydrogenase 5) (Alcohol	40 kDa	0.38	5	0	5	0	0	0	0	2	3	0	0	5.59843845	95% (0.023)

				dehydrogenase class chi chain) (Alcohol dehydrogenase class-III) (Glutathione-dependent formaldehyde dehydrogenase) (FALDH) (FDH) (GSH-FDH) (EC 1.1.1.-) (S-(hydroxymethyl)glutathione dehydrogenase) (EC 1.1.1.284)															
DHB4_HUMAN	AURKA_HUMAN	AURKA	O14965	Aurora kinase A (EC 2.7.11.1) (Aurora 2) (Aurora/IPL1-related kinase 1) (ARK-1) (Aurora-related kinase 1) (hARK1) (Breast tumor-amplified kinase) (Serine/threonine-protein kinase 15) (Serine/threonine-protein kinase 6) (Serine/threonine-protein kinase aurora-A)	46 kDa	0.34	5	0	5	0	0	0	0	0	3	1	0	5.59843845	95% (0.023)
TERA_HUMAN	HEAT2_HUMAN	HEATR2	Q86Y56	HEAT repeat-containing protein 2	94 kDa	0.39	5	0	5	0	0	0	0	1	2	1	0	5.59843845	95% (0.023)
SERPH_HUMAN	FAD1_HUMAN	FLAD1	Q8NFF5	FAD synthase (EC 2.7.7.2) (FAD pyrophosphorylase) (FMN adenylyltransferase) (Flavin adenine dinucleotide synthase) [Includes: Molybdenum cofactor biosynthesis protein-like region; FAD synthase region]	65 kDa	0.41	22	3	19	0	0	0	3	11	2	3	3	5.335851521	95% (0.00016)
H2AY_HUMAN	LAMA5_HUMAN	LAMA5	O15230	Laminin subunit alpha-5 (Laminin-10 subunit alpha) (Laminin-11 subunit alpha) (Laminin-15 subunit alpha)	400 kDa	0.41	10	1	9	1	0	0	0	3	1	3	2	5.101027466	95% (0.0065)
A8K9A4_HUMAN (+7)	MET7B_HUMAN	METTL7B	Q6UX53	Methyltransferase-like protein 7B (EC 2.1.1.-)	28 kDa	0.37	10	1	9	1	0	0	0	6	1	1	1	5.101027466	95% (0.0065)
Q53EP4_HUMAN (+2)	B4DL39_HUMAN		B4DL39	cDNA FLJ57316, highly similar to DNA mismatch repair protein Msh2	77 kDa	0.37	10	1	9	0	0	0	1	2	1	6	0	5.101027466	95% (0.0065)
ACADV_HUMAN	A8K139_HUMAN		A8K139	cDNA FLJ76744, highly similar to Homo sapiens L1 cell adhesion molecule (L1CAM), transcript variant 1, mRNA	140 kDa	0.44	10	1	9	1	0	0	0	7	0	1	1	5.101027466	95% (0.0065)
EFTU_HUMAN	TF3C3_HUMAN	GTF3C3	Q9Y5Q9	General transcription factor 3C polypeptide 3 (Transcription factor IIIC 102 kDa subunit) (TFIIIC 102 kDa subunit) (TFIIIC102) (Transcription factor IIIC subunit gamma) (TF3C-gamma)	101 kDa	0.52	10	1	9	0	0	0	1	4	0	3	2	5.101027466	95% (0.0065)
CALR_HUMAN (+1)	B4DSK8_HUMAN		B4DSK8	cDNA FLJ53515, highly similar to Pogo transposable element with ZNF domain	86 kDa	0.32	10	1	9	0	0	0	1	2	0	7	0	5.101027466	95% (0.0065)
F6KPG5_HUMAN	J3KR97_HUMAN	TBCD	J3KR97	Tubulin-specific chaperone D	137 kDa	0.37	10	1	9	0	1	0	0	1	7	1	0	5.101027466	95% (0.0065)
HBA_HUMAN (+1)	B4DMB5_HUMAN		B4DMB5	CTP synthase (EC 6.3.4.2) (UTP--ammonia ligase)	56 kDa	0.51	10	1	9	0	0	0	1	5	5	0	0	5.101027466	95% (0.0065)
PPIB_HUMAN	CTR2_HUMAN	SLC7A2	P52569	Low affinity cationic amino acid transporter 2 (CAT-2) (CAT2) (Solute carrier family 7 member 2)	72 kDa	0.62	10	1	9	1	0	0	0	1	3	2	3	5.101027466	95% (0.0065)
E9PL22_HUMAN (+1)	LRBA_HUMAN	LRBA	P50851	Lipopolysaccharide-responsive and beige-like anchor protein (Beige-like protein) (CDC4-like protein)	319 kDa	0.31	21	3	18	3	0	0	0	5	9	3	2	5.072279699	95% (0.00029)
U520_HUMAN	A8K3R2_HUMAN		A8K3R2	cDNA FLJ78061, highly similar to Homo sapiens block of proliferation 1, mRNA	84 kDa	0.27	4	0	4	0	0	0	0	2	0	0	2	4.702620648	95% (0.049)
SQRD_HUMAN	J3KNH7_HUMAN	SENP3	J3KNH7	Sentrin-specific protease 3	65 kDa	0.52	4	0	4	0	0	0	0	2	0	2	0	4.702620648	95% (0.049)
Q7Z7M4_HUMAN (+1)	NUP50_HUMAN	NUP50	Q9UKX7	Nuclear pore complex protein Nup50 (50 kDa nucleoporin) (Nuclear pore-associated protein 60 kDa-like) (Nucleoporin Nup50)	50 kDa	0.42	4	0	4	0	0	0	0	2	0	2	0	4.702620648	95% (0.049)

B3KX72_HUMAN (+1)	B4DDT6_HUMAN		B4DDT6	cDNA FLJ60416	18 kDa	0.26	4	0	4	0	0	0	0	0	3	1	0	4.702620648	95% (0.049)
MPCP_HUMAN (+1)	E7EU94_HUMAN	PRPF31	E7EU94	U4/U6 small nuclear ribonucleoprotein Prp31 (Fragment)	29 kDa	0.36	4	0	4	0	0	0	0	2	2	0	0	4.702620648	95% (0.049)
GPDM_HUMAN (+1)	A8K070_HUMAN		A8K070	cDNA FLJ75157, highly similar to Homo sapiens G protein pathway suppressor 1, transcript variant 1, mRNA	59 kDa	0.39	4	0	4	0	0	0	0	1	2	1	0	4.702620648	95% (0.049)
B4E1G2_HUMAN (+3)	A8KAM9_HUMAN		A8KAM9	Peptidyl-prolyl cis-trans isomerase E (PPIase E) (EC 5.2.1.8)	33 kDa	0.41	4	0	4	0	0	0	0	2	0	2	0	4.702620648	95% (0.049)
PHB2_HUMAN	RAB7B_HUMAN	RAB7B	Q96AH8	Ras-related protein Rab-7b	23 kDa	0.63	4	0	4	0	0	0	0	0	0	0	4	4.702620648	95% (0.049)
PHB_HUMAN (+2)	RABP1_HUMAN	CRABP1	P29762	Cellular retinoic acid-binding protein 1 (Cellular retinoic acid-binding protein I) (CRABP-I)	16 kDa	0.56	3	0	4	0	0	0	0	0	4	0	0	4.702620648	95% (0.049)
VDAC1_HUMAN	CRP_HUMAN	CRP	P02741	C-reactive protein [Cleaved into: C-reactive protein(1-205)]	25 kDa	0.43	4	0	4	0	0	0	0	0	0	0	4	4.702620648	95% (0.049)
ESYT1_HUMAN	COQ3_HUMAN	COQ3	Q9NZJ6	Hexaprenyldihydroxybenzoate methyltransferase, mitochondrial (EC 2.1.1.114) (2-polyprenyl-6-hydroxyphenol methylase) (EC 2.1.1.222) (3,4-dihydroxy-5-hexaprenylbenzoate methyltransferase) (DHHB methyltransferase) (DHHB-MT) (DHHB-MTase) (3-demethylubiquinone-10 3-methyltransferase) (EC 2.1.1.64) (Dihydroxyhexaprenylbenzoate methyltransferase)	41 kDa	0.28	4	0	4	0	0	0	0	4	0	0	0	4.702620648	95% (0.049)
B3KVX6_HUMAN (+1)	RM20_HUMAN	MRPL20	Q9BYC9	39S ribosomal protein L20, mitochondrial (L20mt) (MRP-L20)	17 kDa	0.53	4	0	4	0	0	0	0	1	1	0	2	4.702620648	95% (0.049)
F8W6I7_HUMAN (+1)	PTX3_HUMAN	PTX3	P26022	Pentraxin-related protein PTX3 (Pentaxin-related protein PTX3) (Tumor necrosis factor alpha-induced protein 5) (TNF alpha-induced protein 5) (Tumor necrosis factor-inducible gene 14 protein) (TSG-14)	42 kDa	0.44	4	0	4	0	0	0	0	1	0	3	0	4.702620648	95% (0.049)
B4DJV2_HUMAN (+1)	NHRF2_HUMAN	SLC9A3R2	Q15599	Na(+)/H(+) exchange regulatory cofactor NHE-RF2 (NHERF-2) (NHE3 kinase A regulatory protein E3KARP) (SRY-interacting protein 1) (SIP-1) (Sodium-hydrogen exchanger regulatory factor 2) (Solute carrier family 9 isoform A3 regulatory factor 2) (Tyrosine kinase activator protein 1) (TKA-1)	37 kDa	0.32	4	0	4	0	0	0	0	2	0	1	1	4.702620648	95% (0.049)
A6NG51_HUMAN (+1)	GSH0_HUMAN	GCLM	P48507	Glutamate--cysteine ligase regulatory subunit (GCS light chain) (Gamma-ECS regulatory subunit) (Gamma-glutamylcysteine synthetase regulatory subunit) (Glutamate--cysteine ligase modifier subunit)	31 kDa	0.22	4	0	4	0	0	0	0	0	4	0	0	4.702620648	95% (0.049)
HS90A_HUMAN	S35E1_HUMAN	SLC35E1	Q96K37	Solute carrier family 35 member E1	45 kDa	0.41	4	0	4	0	0	0	0	0	2	0	2	4.702620648	95% (0.049)
RAB7A_HUMAN	GMPPA_HUMAN	GMPPA	Q96IJ6	Mannose-1-phosphate guanyltransferase alpha (EC 2.7.7.13) (GDP-mannose pyrophosphorylase A) (GTP-mannose-1-phosphate guanylyltransferase alpha)	46 kDa	0.61	4	0	4	0	0	0	0	0	2	1	1	4.702620648	95% (0.049)
B3KY95_HUMAN (+1)	A8K005_HUMAN		A8K005	cDNA FLJ77896, highly similar to Homo sapiens Ras homolog enriched in brain (RHEB), mRNA	20 kDa	0.3	9	1	8	0	1	0	0	1	4	3	0	4.603300025	95% (0.013)

AT2A2_HUMAN	A0JP11_HUMAN	PIK3R4	A0JP11	Phosphoinositide-3-kinase, regulatory subunit 4	153 kDa	0.32	9	1	8	0	1	0	0	5	2	1	0	4.603300025	95% (0.013)
XRCC6_HUMAN	SAHH3_HUMAN	AHCYL2	Q96HN2	Putative adenosylhomocysteinase 3 (AdoHcyase 3) (EC 3.3.1.1) (S-adenosyl-L-homocysteine hydrolase 3) (S-adenosylhomocysteine hydrolase-like protein 2)	67 kDa	0.38	9	1	8	0	0	0	1	2	2	4	0	4.603300025	95% (0.013)
ODPB_HUMAN	2ABA_HUMAN	PPP2R2A	P63151	Serine/threonine-protein phosphatase 2A 55 kDa regulatory subunit B alpha isoform (PP2A subunit B isoform B55-alpha) (PP2A subunit B isoform PR55-alpha) (PP2A subunit B isoform R2-alpha) (PP2A subunit B isoform alpha)	52 kDa	0.39	9	1	8	1	0	0	0	3	4	2	0	4.603300025	95% (0.013)
LMNB1_HUMAN	RBM10_HUMAN	RBM10	P98175	RNA-binding protein 10 (G patch domain-containing protein 9) (RNA-binding motif protein 10) (RNA-binding protein S1-1) (S1-1)	104 kDa	0.45	9	1	8	0	0	0	1	1	0	4	3	4.603300025	95% (0.013)
FLNB_HUMAN	B3GA3_HUMAN	B3GAT3	O94766	Galactosylgalactosylxylosylprotein 3-beta-glucuronosyltransferase 3 (EC 2.4.1.135) (Beta-1,3-glucuronyltransferase 3) (Glucuronosyltransferase I) (GlcAT-I) (UDP-GlcUA:Gal beta-1,3-Gal-R glucuronyltransferase) (GlcUAT-I)	37 kDa	0.26	9	1	8	0	0	0	1	2	1	2	3	4.603300025	95% (0.013)
Q6FIG4_HUMAN (+1)	COQ9_HUMAN	COQ9	O75208	Ubiquinone biosynthesis protein COQ9, mitochondrial	36 kDa	0.51	9	1	8	1	0	0	0	6	0	2	0	4.603300025	95% (0.013)
B4E2W0_HUMAN (+1)	B4E1G6_HUMAN	GALK1	B4E1G6	Galactokinase (Galactokinase 1) (cDNA FLJ56840, highly similar to Galactokinase (EC 2.7.1.6))	45 kDa	0.39	9	1	8	0	1	0	0	0	6	2	0	4.603300025	95% (0.013)
UGGG1_HUMAN	FPPS_HUMAN	FDPS	P14324	Farnesyl pyrophosphate synthase (FPP synthase) (FPS) (EC 2.5.1.10) ((2E,6E)-farnesyl diphosphate synthase) (Dimethylallyltranstransferase) (EC 2.5.1.1) (Farnesyl diphosphate synthase) (Geranyltranstransferase)	48 kDa	0.24	9	1	8	0	1	0	0	7	0	1	0	4.603300025	95% (0.013)
A8K486_HUMAN (+1)	E1NZA1_HUMAN	PRIC295	E1NZA1	Peroxisome proliferator activated receptor interacting complex protein	293 kDa	0.58	110	21	90	4	2	2	13	40	38	12	2	4.596358964	95% (<0.00010)
CMC2_HUMAN (+1)	WDR75_HUMAN	WDR75	Q8IWA0	WD repeat-containing protein 75	95 kDa	0.4	14	2	12	0	2	0	0	9	0	3	0	4.565222997	95% (0.0035)
MAOM_HUMAN	CH3L1_HUMAN	CHI3L1	P36222	Chitinase-3-like protein 1 (39 kDa synovial protein) (Cartilage glycoprotein 39) (CGP-39) (GP-39) (hCGP-39) (YKL-40)	43 kDa	0.44	29	5	24	3	1	0	1	2	1	3	18	4.524484335	95% (<0.00010)
A2A274_HUMAN (+3)	ALAT2_HUMAN	GPT2	Q8TD30	Alanine aminotransferase 2 (ALT2) (EC 2.6.1.2) (Glutamate pyruvate transaminase 2) (GPT 2) (Glutamic--alanine transaminase 2) (Glutamic--pyruvic transaminase 2)	58 kDa	0.36	29	5	24	0	1	1	3	14	9	1	0	4.524484335	95% (<0.00010)
B4DIT7_HUMAN (+1)	PXDN_HUMAN	PXDN	Q92626	Peroxidasin homolog (EC 1.11.1.7) (Melanoma-associated antigen MG50) (Vascular peroxidase 1) (p53-responsive gene 2 protein)	165 kDa	0.37	23	4	19	4	0	0	0	10	1	2	6	4.319443352	95% (0.00051)
RAB2A_HUMAN	ABCE1_HUMAN	ABCE1	P61221	ATP-binding cassette sub-family E member 1 (2'-5'-oligoadenylate-binding protein) (HuHP68) (RNase L inhibitor) (Ribonuclease 4 inhibitor) (RNS4I)	67 kDa	0.68	17	3	15	0	0	0	3	7	4	4	0	4.281609737	95% (0.0018)
K7EKI8_HUMAN (+1)	RDH10_HUMAN	RDH10	Q8IZV5	Retinol dehydrogenase 10 (EC 1.1.1.300)	38 kDa	0.35	13	2	11	1	0	1	0	0	9	1	1	4.220617143	95% (0.0064)

FAS_HUMAN	PGLT1_HUMAN	POGLUT1	Q8NBL1	Protein O-glucosyltransferase 1 (EC 2.4.1.-) (CAP10-like 46 kDa protein) (hCLP46) (KTEL motif-containing protein 1) (Myelodysplastic syndromes relative protein)	46 kDa	0.25	20	4	18	0	2	1	1	8	4	2	5	4.106078428	95% (0.00090)
NUCL_HUMAN	CSN5_HUMAN	COPS5	Q92905	COP9 signalosome complex subunit 5 (SGN5) (Signalosome subunit 5) (EC 3.4.-.-) (Jun activation domain-binding protein 1)	38 kDa	0.21	8	1	7	0	0	0	1	4	1	2	0	4.105586905	95% (0.024)
HNRPM_HUMAN	B7Z5H3_HUMAN	MAOB	B7Z5H3	Amine oxidase [flavin-containing] B (cDNA FLJ52418, highly similar to Amine oxidase (flavin-containing) B (EC 1.4.3.4))	47 kDa	0.33	8	1	7	0	0	0	1	0	7	0	0	4.105586905	95% (0.024)
LDHA_HUMAN	NUDC_HUMAN	NUDC	Q9Y266	Nuclear migration protein nudC (Nuclear distribution protein C homolog)	38 kDa	0.47	8	1	7	0	0	0	1	5	0	2	0	4.105586905	95% (0.024)
Q59GL1_HUMAN	HPT_HUMAN	HP	P00738	Haptoglobin (Zonulin) [Cleaved into: Haptoglobin alpha chain; Haptoglobin beta chain]	45 kDa	0.31	16	3	14	0	2	0	1	0	2	5	6	4.01806825	95% (0.0032)
AMPL_HUMAN	A8KA19_HUMAN		A8KA19	cDNA FLJ75831, highly similar to Homo sapiens exportin, tRNA (nuclear export receptor for tRNAs) (XPOT), mRNA	110 kDa	0.47	12	2	10	0	1	0	1	4	4	2	0	3.876021205	95% (0.012)
MVP_HUMAN	NTPCR_HUMAN	NTPCR	Q9BSD7	Cancer-related nucleoside-triphosphatase (NTPase) (EC 3.6.1.15) (Nucleoside triphosphate phosphohydrolase)	21 kDa	0.36	12	2	10	2	0	0	0	1	2	0	7	3.876021205	95% (0.012)
B4DY09_HUMAN (+2)	SAFB2_HUMAN	SAFB2	Q14151	Scaffold attachment factor B2 (SAF-B2)	107 kDa	0.29	12	2	10	2	0	0	0	6	0	2	2	3.876021205	95% (0.012)
A6NN80_HUMAN (+2)	CO9_HUMAN	C9	P02748	Complement component C9 [Cleaved into: Complement component C9a; Complement component C9b]	63 kDa	0.39	12	2	10	0	0	0	2	0	0	0	10	3.876021205	95% (0.012)
PABP1_HUMAN	ACPM_HUMAN	NDUFAB1	O14561	Acyl carrier protein, mitochondrial (ACP) (CI-SDAP) (NADH-ubiquinone oxidoreductase 9.6 kDa subunit)	17 kDa	0.28	3	0	3	0	0	0	1	3	0	0	0	3.806828619	0% (0.10)
DLDH_HUMAN	E9PPN3_HUMAN	SCYL1	E9PPN3	N-terminal kinase-like protein	74 kDa	0.32	3	0	3	0	0	0	0	0	3	0	0	3.806828619	0% (0.10)
ITPR3_HUMAN	GLE1_HUMAN	GLE1	Q53GS7	Nucleoporin GLE1 (hGLE1) (GLE1-like protein)	80 kDa	0.21	3	0	3	0	0	0	0	0	0	3	0	3.806828619	0% (0.10)
PRP8_HUMAN	MDC1_HUMAN	MDC1	Q14676	Mediator of DNA damage checkpoint protein 1 (Nuclear factor with BRCT domains 1)	227 kDa	0.17	3	0	3	0	0	0	0	0	0	3	0	3.806828619	0% (0.10)
ALDH2_HUMAN (+1)	B7Z7F0_HUMAN		B7Z7F0	cDNA FLJ56420, highly similar to Aspartyl aminopeptidase (EC 3.4.11.21)	54 kDa	0.35	3	0	3	0	0	0	0	0	3	0	0	3.806828619	0% (0.10)
DESP_HUMAN	IPO8_HUMAN	IPO8	O15397	Importin-8 (Imp8) (Ran-binding protein 8) (RanBP8)	120 kDa	0.12	3	0	3	0	0	0	0	2	0	1	0	3.806828619	0% (0.10)
K7EJE8_HUMAN (+1)	ABCF2_HUMAN	ABCF2	Q9UG63	ATP-binding cassette sub-family F member 2 (Iron-inhibited ABC transporter 2)	71 kDa	0.38	3	0	3	0	0	0	0	0	2	1	0	3.806828619	0% (0.10)
XRCC5_HUMAN	A4D212_HUMAN	DKFZP586J0619	A4D212	DKFZP586J0619 protein (Integrator complex subunit 1)	266 kDa	0.28	3	0	3	0	0	0	0	0	0	3	0	3.806828619	0% (0.10)
AL7A1_HUMAN	B3KM36_HUMAN		B3KM36	cDNA FLJ10153 fis, clone HEMBA1003417, highly similar to BAG family molecular chaperone regulator 2	24 kDa	0.39	3	0	3	0	0	0	0	0	3	0	0	3.806828619	0% (0.10)
ANX11_HUMAN (+1)	B3KQV7_HUMAN	SLC29A1	B3KQV7	Equilibrative nucleoside transporter 1 (Solute carrier family 29 (Nucleoside transporters), member 1, isoform CRA_b) (cDNA FLJ33172 fis, clone ADRGL2002029, highly similar to Equilibrative nucleoside transporter 1)	59 kDa	0.37	3	0	3	0	0	0	0	1	1	1	0	3.806828619	0% (0.10)
B2R6E5_HUMAN (+2)	DDX54_HUMAN	DDX54	Q8TDD1	ATP-dependent RNA helicase DDX54 (EC 3.6.4.13) (ATP-dependent RNA helicase DP97) (DEAD box RNA	99 kDa	0.24	3	0	3	0	0	0	0	0	0	3	0	3.806828619	0% (0.10)

				helicase 97 kDa) (DEAD box protein 54)															
EF2_HUMAN	E7ESL0_HUMAN	MRPL22	E7ESL0	39S ribosomal protein L22, mitochondrial	24 kDa	0.3	3	0	3	0	0	0	0	2	0	0	1	3.806828619	0% (0.10)
A8K590_HUMAN (+3)	B4DZK0_HUMAN		B4DZK0	cDNA FLJ56391, highly similar to Cysteine protease ATG4B (EC 3.4.22.-)	57 kDa	0.34	3	0	3	0	0	0	0	1	2	0	0	3.806828619	0% (0.10)
B7Z992_HUMAN	A8K8C2_HUMAN		A8K8C2	cDNA FLJ76932, highly similar to Homo sapiens tubulin-specific chaperone e (TBCE), mRNA	59 kDa	0.25	3	0	3	0	0	0	0	0	3	0	0	3.806828619	0% (0.10)
MOES_HUMAN	Q658W7_HUMAN	DKFZp666L2010	Q658W7	Putative uncharacterized protein DKFZp666L2010 (Fragment)	92 kDa	0.29	3	0	3	0	0	0	0	0	3	0	0	3.806828619	0% (0.10)
QCR2_HUMAN	B7Z7N6_HUMAN	PDK1	B7Z7N6	[Pyruvate dehydrogenase (acetyl-transferring)] kinase isozyme 1, mitochondrial (cDNA FLJ51565, highly similar to Pyruvate dehydrogenase (lipoamide) kinase isozyme 1 (EC 2.7.11.2))	41 kDa	0.37	3	0	3	0	0	0	0	2	0	0	1	3.806828619	0% (0.10)
GSTK1_HUMAN	DDX56_HUMAN	DDX56	Q9NY93	Probable ATP-dependent RNA helicase DDX56 (EC 3.6.4.13) (ATP-dependent 61 kDa nucleolar RNA helicase) (DEAD box protein 21) (DEAD box protein 56)	62 kDa	0.43	3	0	3	0	0	0	0	1	0	1	0	3.806828619	0% (0.10)
B9A067_HUMAN (+1)	MRRP3_HUMAN	KIAA0391	O15091	Mitochondrial ribonuclease P protein 3 (Mitochondrial RNase P protein 3)	67 kDa	0.34	3	0	3	0	0	0	0	3	0	0	0	3.806828619	0% (0.10)
COPA_HUMAN	H0YEE1_HUMAN	VPS51	H0YEE1	Vacuolar protein sorting-associated protein 51 homolog (Fragment)	22 kDa	0.27	3	0	3	0	0	0	0	1	1	1	0	3.806828619	0% (0.10)
ECHM_HUMAN	BRCC3_HUMAN	BRCC3	P46736	Lys-63-specific deubiquitinase BRCC36 (EC 3.4.19.-) (BRCA1-A complex subunit BRCC36) (BRCA1/BRCA2-containing complex subunit 3) (BRCA1/BRCA2-containing complex subunit 36) (BRISC complex subunit BRCC36)	36 kDa	0.54	3	0	3	0	0	0	0	0	3	0	0	3.806828619	0% (0.10)
ERO1A_HUMAN	Q7Z524_HUMAN		Q7Z524	HUMEEP	51 kDa	0.27	3	0	3	0	0	0	0	1	1	1	0	3.806828619	0% (0.10)
HSPB1_HUMAN	A8K6N3_HUMAN		A8K6N3	cDNA FLJ76886, highly similar to Homo sapiens loss of heterozygosity, 11, chromosomal region 2, gene A (LOH11CR2A), transcript variant 1, mRNA	87 kDa	0.54	3	0	3	0	0	0	0	0	3	0	0	3.806828619	0% (0.10)
B4E380_HUMAN	B7SBB1_HUMAN	AIP	B7SBB1	Aryl hydrocarbon receptor interacting protein	38 kDa	0.39	3	0	3	0	0	0	0	0	3	0	0	3.806828619	0% (0.10)
OST48_HUMAN	UBA7_HUMAN	UBA7	P41226	Ubiquitin-like modifier-activating enzyme 7 (Ubiquitin-activating enzyme 7) (D8) (Ubiquitin-activating enzyme E1 homolog)	112 kDa	0.27	3	0	3	0	0	0	0	0	3	0	0	3.806828619	0% (0.10)
F5H897_HUMAN (+2)	A2IDC6_HUMAN	MRPL28	A2IDC6	39S ribosomal protein L28, mitochondrial (Fragment)	29 kDa	0.38	3	0	3	0	0	0	0	1	0	0	2	3.806828619	0% (0.10)
ODO1_HUMAN	B3KY51_HUMAN		B3KY51	cDNA FLJ46863 fis, clone UTERU3011558	78 kDa	0.27	3	0	3	0	0	0	0	3	0	0	0	3.806828619	0% (0.10)
PRDX1_HUMAN	H7C189_HUMAN	USP4	H7C189	Ubiquitin carboxyl-terminal hydrolase (EC 3.4.19.12) (Fragment)	70 kDa	0.45	3	0	3	0	0	0	0	0	4	0	0	3.806828619	0% (0.10)
B4DWS6_HUMAN (+1)	INT5_HUMAN	INTS5	Q6P9B9	Integrator complex subunit 5 (Int5)	108 kDa	0.39	3	0	3	0	0	0	0	0	0	2	1	3.806828619	0% (0.10)
E7ENR4_HUMAN	PHS_HUMAN	PCBD1	P61457	Pterin-4-alpha-carbinolamine dehydratase (PHS) (EC 4.2.1.96) (4-alpha-hydroxy-tetrahydropterin dehydratase) (Dimerization cofactor of hepatocyte nuclear factor 1-alpha) (DCoH) (Dimerization cofactor of HNF1) (Phenylalanine hydroxylase-stimulating protein) (Pterin carbinolamine dehydratase) (PCD)	12 kDa	0.3	2	0	3	0	0	0	0	0	0	2	0	3.806828619	0% (0.10)

UBA1_HUMAN	B7Z2F7_HUMAN		B7Z2F7	cDNA FLJ54655, highly similar to Heat shock 70 kDa protein 12A	66 kDa	0.25	2	0	3	0	0	0	0	0	2	0	0	3.806828619	0% (0.10)
A8K525_HUMAN (+1)	CASP6_HUMAN	CASP6	P55212	Caspase-6 (CASP-6) (EC 3.4.22.59) (Apoptotic protease Mch-2) [Cleaved into: Caspase-6 subunit p18; Caspase-6 subunit p11]	33 kDa	0.27	3	0	3	0	0	0	0	0	3	0	0	3.806828619	0% (0.10)
PROF1_HUMAN	GATC_HUMAN	GATC	O43716	Glutamyl-tRNA(Gln) amidotransferase subunit C, mitochondrial (Glu-AdT subunit C) (EC 6.3.5.-) (Protein 15E1.2)	15 kDa	0.73	3	0	3	0	0	0	0	2	0	0	1	3.806828619	0% (0.10)
A8KAP3_HUMAN (+5)	HID1_HUMAN	HID1	Q8IV36	Protein HID1 (Down-regulated in multiple cancers 1) (HID1 domain-containing protein) (Protein hid-1 homolog)	89 kDa	0.31	3	0	3	0	0	0	0	0	2	1	0	3.806828619	0% (0.10)
AMPN_HUMAN (+1)	F8VWL0_HUMAN	TFCP2	F8VWL0	Alpha-globin transcription factor CP2 (Fragment)	44 kDa	0.28	3	0	3	0	0	0	0	1	1	1	0	3.806828619	0% (0.10)
HNRPL_HUMAN (+1)	A6ND99_HUMAN	HS2ST1	A6ND99	Heparan sulfate 2-O-sulfotransferase 1	37 kDa	0.25	3	0	3	0	0	0	0	0	2	0	1	3.806828619	0% (0.10)
ROA3_HUMAN	ELP1_HUMAN	IKBKAP	O95163	Elongator complex protein 1 (ELP1) (IkappaB kinase complex-associated protein) (IKK complex-associated protein) (p150)	150 kDa	0.36	3	0	3	0	0	0	0	3	0	0	0	3.806828619	0% (0.10)
SF3B3_HUMAN	B2R533_HUMAN		B2R533	cDNA, FLJ92320, highly similar to Homo sapiens glutathione S-transferase theta 2 (GSTT2), mRNA	28 kDa	0.27	3	0	3	0	0	0	0	3	0	0	0	3.806828619	0% (0.10)
C9J9K3_HUMAN (+1)	B3KRF9_HUMAN		B3KRF9	cDNA FLJ34156 fis, clone FCBBF3013266, highly similar to Tsukushi (Leucine-richrepeat-containing protein 54)	34 kDa	0.45	3	0	3	0	0	0	0	0	3	0	0	3.806828619	0% (0.10)
I3L1P8_HUMAN	G3V4W4_HUMAN	GNPNAT1	G3V4W4	Glucosamine 6-phosphate N-acetyltransferase (Fragment)	18 kDa	0.46	3	0	3	0	0	0	0	0	3	0	0	3.806828619	0% (0.10)
Q59GY2_HUMAN	PCH2_HUMAN	TRIP13	Q15645	Pachytene checkpoint protein 2 homolog (Human papillomavirus type 16 E1 protein-binding protein) (16E1-BP) (HPV16 E1 protein-binding protein) (Thyroid hormone receptor interactor 13) (Thyroid receptor-interacting protein 13) (TR-interacting protein 13) (TRIP-13)	49 kDa	0.28	3	0	3	0	0	0	0	3	0	0	0	3.806828619	0% (0.10)
AATM_HUMAN (+1)	A8K329_HUMAN		A8K329	cDNA FLJ76656, highly similar to Homo sapiens scaffold attachment factor B (SAFB), mRNA	103 kDa	0.37	16	3	13	1	1	0	1	7	0	3	3	3.754534347	95% (0.0056)
STT3A_HUMAN	A8K454_HUMAN		A8K454	cDNA FLJ78187, highly similar to Homo sapiens calmegin, mRNA	70 kDa	0.17	37	8	29	4	0	0	0	9	9	6	6	3.66256952	95% (< 0.00010)
E9PCY7_HUMAN (+2)	TOP2A_HUMAN	TOP2A	P11388	DNA topoisomerase 2-alpha (EC 5.99.1.3) (DNA topoisomerase II, alpha isozyme)	174 kDa	0.26	88	20	68	0	9	9	2	26	18	14	10	3.651236066	95% (< 0.00010)
RAB14_HUMAN	B4DQ14_HUMAN	EIF2A	B4DQ14	Eukaryotic translation initiation factor 2A (cDNA FLJ58035, highly similar to Homo sapiens eukaryotic translation initiation factor (eIF) 2A (eIF2A), mRNA)	62 kDa	0.63	7	1	6	0	0	0	1	3	3	1	0	3.607888104	95% (0.045)
B2ZDQ1_HUMAN (+1)	RT05_HUMAN	MRPS5	P82675	28S ribosomal protein S5, mitochondrial (MRP-S5) (S5mt)	48 kDa	0.49	7	1	6	1	0	0	0	1	0	1	4	3.607888104	95% (0.045)
B4DUQ1_HUMAN	B4DMJ9_HUMAN		B4DMJ9	cDNA FLJ60022, highly similar to DnaJ homolog subfamily B member 6	24 kDa	0.4	7	1	6	1	0	0	0	2	1	1	2	3.607888104	95% (0.045)
EPIPL_HUMAN	EI2BA_HUMAN	EIF2B1	Q14232	Translation initiation factor eIF-2B subunit alpha (eIF-2B GDP-GTP exchange factor subunit alpha)	34 kDa	0.081	7	1	6	0	0	0	1	3	3	0	0	3.607888104	95% (0.045)
ANXA4_HUMAN (+1)	SYAM_HUMAN	AARS2	Q5JTZ9	Alanine--tRNA ligase, mitochondrial (EC 6.1.1.7) (Alanyl-tRNA synthetase) (AlaRS)	107 kDa	0.5	7	1	6	1	0	0	0	5	0	0	1	3.607888104	95% (0.045)

SYIM_HUMAN	F210A_HUMAN	FAM210A	Q96ND0	Protein FAM210A	31 kDa	0.29	7	1	6	0	0	1	0	1	2	2	1	3.607888104	95% (0.045)
H3BLZ8_HUMAN (+1)	B4DU97_HUMAN	SLC25A17	B4DU97	Peroxisomal membrane protein PMP34 (cDNA FLJ57596, highly similar to Peroxisomal membrane protein PMP34)	31 kDa	0.28	7	1	6	0	1	0	0	2	4	0	0	3.607888104	95% (0.045)
A8K7F6_HUMAN (+1)	VIR_HUMAN	KIAA1429	Q69YN4	Protein virilizer homolog	202 kDa	0.39	11	2	9	0	1	1	0	2	0	7	0	3.531435181	95% (0.021)
AIFM1_HUMAN	PCCA_HUMAN	PCCA	P05165	Propionyl-CoA carboxylase alpha chain, mitochondrial (PCCase subunit alpha) (EC 6.4.1.3) (Propanoyl-CoA:carbon dioxide ligase subunit alpha)	80 kDa	0.33	11	2	9	0	0	1	1	3	0	1	5	3.531435181	95% (0.021)
B2RBR9_HUMAN (+1)	MMS19_HUMAN	MMS19	Q96T76	MMS19 nucleotide excision repair protein homolog (hMMS19) (MET18 homolog) (MMS19-like protein)	113 kDa	0.27	10	2	9	0	0	0	2	2	5	1	0	3.531435181	95% (0.021)
B4DE36_HUMAN (+1)	FA98A_HUMAN	FAM98A	Q8NCA5	Protein FAM98A	55 kDa	0.3	11	2	9	0	0	0	2	3	3	1	2	3.531435181	95% (0.021)
B7Z8A2_HUMAN (+1)	SYAC_HUMAN	AARS	P49588	Alanine--tRNA ligase, cytoplasmic (EC 6.1.1.7) (Alanyl-tRNA synthetase) (AlaRS) (Renal carcinoma antigen NY-REN-42)	107 kDa	0.39	49	11	37	0	2	4	5	15	18	6	0	3.497283516	95% (< 0.00010)
PLSL_HUMAN (+1)	Q59FH1_HUMAN		Q59FH1	Transformation/transcription domain-associated protein variant (Fragment)	406 kDa	0.47	15	3	12	1	0	0	2	2	0	9	1	3.491008026	95% (0.0098)
GNAI2_HUMAN	AAPK1_HUMAN	PRKAA1	Q13131	5'-AMP-activated protein kinase catalytic subunit alpha-1 (AMPK subunit alpha-1) (EC 2.7.11.1) (Acetyl-CoA carboxylase kinase) (ACACA kinase) (EC 2.7.11.27) (Hydroxymethylglutaryl-CoA reductase kinase) (HMGCR kinase) (EC 2.7.11.31) (Tau-protein kinase PRKAA1) (EC 2.7.11.26)	64 kDa	0.4	15	3	12	0	2	1	0	5	5	2	0	3.491008026	95% (0.0098)
P4HA1_HUMAN	B9A018_HUMAN	USP39	B9A018	U4/U6.U5 tri-snRNP-associated protein 2	62 kDa	0.43	15	3	12	1	0	2	0	0	0	11	1	3.491008026	95% (0.0098)
3HIDH_HUMAN	UN45A_HUMAN	UNC45A	Q9H3U1	Protein unc-45 homolog A (Unc-45A) (GCUNC-45) (Smooth muscle cell-associated protein 1) (SMAP-1)	103 kDa	0.33	19	4	15	0	0	0	4	2	8	4	0	3.466020492	95% (0.0047)
B4DJB4_HUMAN (+2)	B2R769_HUMAN		B2R769	cDNA, FLJ93308, highly similar to Homo sapiens leukocyte-derived arginine aminopeptidase (LRAP), mRNA	110 kDa	0.35	64	15	49	0	4	5	6	14	1	18	16	3.46394414	95% (< 0.00010)
B7Z5J7_HUMAN (+2)	E7EUY3_HUMAN	PCCB	E7EUY3	Propionyl-CoA carboxylase beta chain, mitochondrial	56 kDa	0.32	38	9	29	1	3	2	3	17	1	3	9	3.305203195	95% (0.0002 3)
A7E2Y5_HUMAN (+1)	B2R5W3_HUMAN		B2R5W3	cDNA, FLJ92658, highly similar to Homo sapiens poly (ADP-ribose) polymerase family, member 1 (PARP1), mRNA	113 kDa	0.17	104	26	78	3	7	8	8	33	23	15	8	3.258673749	95% (< 0.00010)
PCKGM_HUMAN	M1V485_HUMAN	SLC34A2-ROS1	M1V485	Tyrosine-protein kinase receptor (EC 2.7.10.1)	141 kDa	0.32	54	13	40	4	4	2	3	8	1	14	18	3.242292332	95% (< 0.00010)
HNRL2_HUMAN	A7J992_HUMAN	PHIP	A7J992	Pleckstrin homology domain-interacting protein variant 1	207 kDa	0.33	11	2	8	0	1	0	1	4	0	3	1	3.186859072	95% (0.037)
SPTB2_HUMAN	B4E0B1_HUMAN		B4E0B1	cDNA FLJ52100	101 kDa	0.21	10	2	8	0	1	0	1	6	1	1	0	3.186859072	95% (0.037)
B5BUB1_HUMAN (+1)	CLP1L_HUMAN	CLPTM1L	Q96KA5	Cleft lip and palate transmembrane protein 1-like protein (CLPTM1-like protein) (Cisplatin resistance-related protein 9) (CRR9p)	62 kDa	0.44	10	2	8	0	0	2	0	2	0	4	2	3.186859072	95% (0.037)
CTNA1_HUMAN	RUFY1_HUMAN	RUFY1	Q96T51	RUN and FYVE domain-containing protein 1 (FYVE-finger protein EIP1) (La-binding protein 1) (Rab4-interacting protein) (Zinc	80 kDa	0.34	10	2	8	1	0	0	1	3	2	3	0	3.186859072	95% (0.037)

				finger FYVE domain-containing protein 12)															
CO3_HUMAN	B4DHJ3_HUMAN		B4DHJ3	DNA-directed RNA polymerase (EC 2.7.7.6)	133 kDa	0.24	24	6	19	0	2	1	3	2	2	12	3	3.127792396	95% (0.0031)
HCD2_HUMAN	NOL9_HUMAN	NOL9	Q5SY16	Polynucleotide 5'-hydroxyl-kinase NOL9 (EC 2.7.1.-) (Nucleolar protein 9)	79 kDa	0.52	6	1	5	0	0	0	1	2	2	1	1	3.110203623	0% (0.084)
TLN1_HUMAN	Q5J8M4_HUMAN		Q5J8M4	Migration-inducing gene 14	39 kDa	0.17	6	1	5	0	0	0	1	0	1	3	1	3.110203623	0% (0.084)
B7ZB41_HUMAN (+1)	E9PHY5_HUMAN	EPB41L2	E9PHY5	Band 4.1-like protein 2	104 kDa	0.3	6	1	5	0	1	0	0	0	5	0	0	3.110203623	0% (0.084)
ERP44_HUMAN	NFKB2_HUMAN	NFKB2	Q00653	Nuclear factor NF-kappa-B p100 subunit (DNA-binding factor KBF2) (H2TF1) (Lymphocyte translocation chromosome 10 protein) (Nuclear factor of kappa light polypeptide gene enhancer in B-cells 2) (Oncogene Lyt-10) (Lyt10) [Cleaved into: Nuclear factor NF-kappa-B p52 subunit]	97 kDa	0.34	6	1	5	0	1	0	0	0	5	0	0	3.110203623	0% (0.084)
E9PH29_HUMAN (+1)	GATA_HUMAN	QRSL1	Q9H0R6	Glutamyl-tRNA(Gln) amidotransferase subunit A, mitochondrial (Glu-AdT subunit A) (EC 6.3.5.-) (Glutaminyl-tRNA synthase-like protein 1)	57 kDa	0.34	6	1	5	0	0	0	0	4	0	0	1	3.110203623	0% (0.084)
QCR1_HUMAN	ECSIT_HUMAN	ECSIT	Q9BQ95	Evolutionarily conserved signaling intermediate in Toll pathway, mitochondrial (Protein SITPEC)	49 kDa	0.26	5	1	5	1	0	0	0	3	0	0	2	3.110203623	0% (0.084)
A0PJ92_HUMAN (+2)	E9PIN3_HUMAN	NXF1	E9PIN3	Nuclear RNA export factor 1 (Fragment)	68 kDa	0.31	6	1	5	0	0	0	1	2	0	3	0	3.110203623	0% (0.084)
ERP29_HUMAN	NOG2_HUMAN	GNL2	Q13823	Nucleolar GTP-binding protein 2 (Autoantigen NGP-1)	84 kDa	0.34	6	1	5	0	0	0	1	3	0	2	0	3.110203623	0% (0.084)
VATB2_HUMAN	ENOPH_HUMAN	ENOPH1	Q9UHY7	Enolase-phosphatase E1 (EC 3.1.3.77) (2,3-diketo-5-methylthio-1-phosphopentane phosphatase) (MASA homolog)	29 kDa	0.32	6	1	5	0	0	0	1	2	0	2	1	3.110203623	0% (0.084)
ECH1_HUMAN	M0QYR6_HUMAN	PLAUR	M0QYR6	Urokinase plasminogen activator surface receptor (Fragment)	25 kDa	0.46	6	1	5	0	1	0	0	0	0	2	3	3.110203623	0% (0.084)
VDAC2_HUMAN	B2R5R5_HUMAN		B2R5R5	cDNA, FLJ92583, highly similar to Homo sapiens glycogenin (GYG), mRNA	39 kDa	0.34	6	1	5	0	0	1	0	4	1	0	0	3.110203623	0% (0.084)
A8K766_HUMAN (+1)	B3KUJ0_HUMAN		B3KUJ0	cDNA FLJ39996 fis, clone STOMA2002166, highly similar to Splicing factor 3B subunit 4	44 kDa	0.47	6	1	5	0	0	1	0	1	0	3	1	3.110203623	0% (0.084)
LRC59_HUMAN	K7ELW0_HUMAN	PARK7	K7ELW0	Protein DJ-1	18 kDa	0.33	21	5	16	1	1	2	1	5	5	3	3	3.090628568	95% (0.0065)
Q53HV1_HUMAN (+2)	BCAM_HUMAN	BCAM	P50895	Basal cell adhesion molecule (Auberger B antigen) (B-CAM cell surface glycoprotein) (F8/G253 antigen) (Lutheran antigen) (Lutheran blood group glycoprotein) (CD antigen CD239)	67 kDa	0.43	42	11	32	0	2	1	8	14	0	16	2	3.039903518	95% (0.00025)
B2R8R5_HUMAN (+1)	Q6IAX9_HUMAN	SRPR	Q6IAX9	SRPR protein	70 kDa	0.17	65	17	48	0	5	6	6	18	12	15	4	3.022832841	95% (< 0.00010)
RUVB2_HUMAN	F8VPD4_HUMAN	CAD	F8VPD4	CAD protein	236 kDa	0.39	39	10	29	3	2	2	3	17	7	4	1	3.011368661	95% (0.00049)
DHX15_HUMAN	AGRIN_HUMAN	AGRN	O00468	Agrin [Cleaved into: Agrin N-terminal 110 kDa subunit; Agrin C-terminal 110 kDa subunit; Agrin C-terminal 90 kDa fragment (C90); Agrin C-terminal 22 kDa fragment (C22)]	217 kDa	0.31	13	3	10	0	2	0	1	8	0	2	0	2.963978133	95% (0.029)
NDUS3_HUMAN	F5GZS0_HUMAN	DHX36	F5GZS0	Probable ATP-dependent RNA helicase DHX36	113 kDa	0.42	13	3	10	0	1	2	0	5	2	2	1	2.963978133	95% (0.029)

NB5R3_HUMAN	B4DY16_HUMAN		B4DY16	cDNA FLJ51385, highly similar to Ran GTPase-activating protein 1	58 kDa	0.31	13	3	10	0	1	0	2	6	2	2	0	2.963978133	95% (0.029)
ARP2_HUMAN	B4DJK9_HUMAN		B4DJK9	Perilipin	45 kDa	0.35	13	3	10	1	0	1	1	0	1	3	6	2.963978133	95% (0.029)
B2R888_HUMAN (+2)	Q6NVW7_HUMAN	KPNA2	Q6NVW7	Importin subunit alpha	58 kDa	0.29	49	13	36	0	3	8	2	5	8	18	5	2.927719674	95% (0.00016)
STAT1_HUMAN	SON_HUMAN	SON	P18583	Protein SON (Bax antagonist selected in saccharomyces 1) (BASS1) (Negative regulatory element-binding protein) (NRE-binding protein) (Protein DBP-5) (SON3)	264 kDa	0.35	20	5	15	0	1	3	1	6	0	6	3	2.911419805	95% (0.011)
A8K4W2_HUMAN (+1)	E7D7X9_HUMAN		E7D7X9	Pyrroline-5-carboxylate reductase (EC 1.5.1.2)	33 kDa	0.44	20	5	15	0	2	2	1	5	6	4	0	2.911419805	95% (0.011)
B2R8N0_HUMAN (+1)	G8JLD3_HUMAN	ERC1	G8JLD3	ELKS/Rab6-interacting/CAST family member 1	125 kDa	0.29	2	0	2	0	0	0	0	0	2	0	0	2.911062362	0% (0.22)
A8K4Z4_HUMAN (+1)	B3KXX5_HUMAN		B3KXX5	Structural maintenance of chromosomes protein	144 kDa	0.31	2	0	2	0	0	0	0	2	0	0	0	2.911062362	0% (0.22)
Q96MH4_HUMAN	CENPF_HUMAN	CENPF	P49454	Centromere protein F (CENP-F) (AH antigen) (Kinetochore protein CENPF) (Mitosin)	368 kDa	0.12	2	0	2	0	0	0	0	0	0	2	0	2.911062362	0% (0.22)
Q6PJ75_HUMAN	CO6A3_HUMAN	COL6A3	P12111	Collagen alpha-3(VI) chain	344 kDa	0.27	2	0	2	0	0	0	0	0	0	2	0	2.911062362	0% (0.22)
RS3_HUMAN	C2D1A_HUMAN	CC2D1A	Q6P1N0	Coiled-coil and C2 domain-containing protein 1A (Akt kinase-interacting protein 1) (Five prime repressor element under dual repression-binding protein 1) (FRE under dual repression-binding protein 1) (Freud-1) (Putative NF-kappa-B-activating protein 023N)	104 kDa	0.52	2	0	2	0	0	0	0	2	0	0	0	2.911062362	0% (0.22)
ETFA_HUMAN	ARHG2_HUMAN	ARHGEF2	Q92974	Rho guanine nucleotide exchange factor 2 (Guanine nucleotide exchange factor H1) (GEF-H1) (Microtubule-regulated Rho-GEF) (Proliferating cell nucleolar antigen p40)	112 kDa	0.33	2	0	2	0	0	0	0	1	1	0	0	2.911062362	0% (0.22)
ODO2_HUMAN	B2RCJ6_HUMAN		B2RCJ6	cDNA, FLJ96114, highly similar to Homo sapiens bromodomain and WD repeat domain containing 2 (BRWD2), mRNA	137 kDa	0.21	2	0	2	0	0	0	0	1	1	0	0	2.911062362	0% (0.22)
B2RAQ8_HUMAN (+1)	B2R6G5_HUMAN		B2R6G5	cDNA, FLJ92942, highly similar to Homo sapiens T-box, brain, 1 (TBR1), mRNA	74 kDa	0.33	2	0	2	0	0	0	0	1	0	0	1	2.911062362	0% (0.22)
NU205_HUMAN	ADNP_HUMAN	ADNP	Q9H2P0	Activity-dependent neuroprotector homeobox protein (Activity-dependent neuroprotective protein)	124 kDa	0.15	2	0	2	0	0	0	0	1	0	1	0	2.911062362	0% (0.22)
MTCH2_HUMAN	NEK9_HUMAN	NEK9	Q8TD19	Serine/threonine-protein kinase Nek9 (EC 2.7.11.1) (Nercc1 kinase) (Never in mitosis A-related kinase 9) (NimA-related protein kinase 9) (NimA-related kinase 8) (Nek8)	107 kDa	0.35	2	0	2	0	0	0	0	1	1	0	0	2.911062362	0% (0.22)
PYC_HUMAN	A7E2E5_HUMAN	PDE3B	A7E2E5	Phosphodiesterase 3B, cGMP-inhibited	124 kDa	0.25	2	0	2	0	0	0	0	0	1	1	0	2.911062362	0% (0.22)
TPIS_HUMAN	A8K5G0_HUMAN		A8K5G0	cDNA FLJ76836, highly similar to Homo sapiens splicing factor, arginine/serine-rich 14 (SFRS14), transcript variant 2, mRNA	120 kDa	0.56	2	0	2	0	0	0	0	1	0	0	1	2.911062362	0% (0.22)
B7ZLE5_HUMAN	Q59ET0_HUMAN		Q59ET0	Glucan , branching enzyme 1 variant (Fragment)	86 kDa	0.2	2	0	2	0	0	0	0	0	2	0	0	2.911062362	0% (0.22)
ALDOA_HUMAN (+1)	PTPRK_HUMAN	PTPRK	Q15262	Receptor-type tyrosine-protein phosphatase kappa (Protein-tyrosine phosphatase kappa) (R-PTP-kappa) (EC 3.1.3.48)	162 kDa	0.38	2	0	2	0	0	0	0	3	0	0	0	2.911062362	0% (0.22)
FACE1_HUMAN	FN3K_HUMAN	FN3K	Q9H479	Fructosamine-3-kinase (EC 2.7.1.-)	35 kDa	0.24	2	0	2	0	0	0	0	0	2	0	0	2.911062362	0% (0.22)
H12_HUMAN	B4DZX1_HUMAN		B4DZX1	cDNA FLJ53892, highly similar to Pantothenate kinase 4 (EC 2.7.1.33)	81 kDa	0.36	1	0	2	0	0	0	0	0	2	0	0	2.911062362	0% (0.22)

Q59GW8_HUMAN	DPOD1_HUMAN	POLD1	P28340	DNA polymerase delta catalytic subunit (EC 2.7.7.7) (DNA polymerase subunit delta p125)	124 kDa	0.3	2	0	2	0	0	0	0	2	0	0	0	2.911062362	0% (0.22)
A8K2H4_HUMAN (+2)	KIF1A_HUMAN	KIF1A	Q12756	Kinesin-like protein KIF1A (Axonal transporter of synaptic vesicles) (Microtubule-based motor KIF1A) (Unc-104- and KIF1A-related protein) (hUnc-104)	191 kDa	0.3	2	0	2	0	0	0	0	0	2	0	0	2.911062362	0% (0.22)
D3DSQ1_HUMAN	TM38B_HUMAN	TMEM38B	Q9NVV0	Trimeric intracellular cation channel type B (TRIC-B) (TRICB) (Transmembrane protein 38B)	33 kDa	0.21	3	0	2	0	0	0	0	0	0	0	2	2.911062362	0% (0.22)
B7Z3E5_HUMAN (+2)	B3KUH0_HUMAN		B3KUH0	cDNA FLJ39883 fis, clone SPLEN2016268, highly similar to Protein CHMP7	51 kDa	0.23	2	0	2	0	0	0	0	0	2	0	0	2.911062362	0% (0.22)
NB5R1_HUMAN	VPS39_HUMAN	VPS39	Q96JC1	Vam6/Vps39-like protein (TRAP1-like protein) (hVam6p)	102 kDa	0.37	2	0	2	0	0	0	0	2	0	0	0	2.911062362	0% (0.22)
ATPG_HUMAN (+1)	B4DL80_HUMAN	CDC27	B4DL80	Cell division cycle protein 27 homolog (cDNA FLJ57525, highly similar to Cell division cycle protein 27 homolog)	85 kDa	0.34	2	0	2	0	0	0	0	0	1	1	0	2.911062362	0% (0.22)
Q58F09_HUMAN	A8K7A1_HUMAN		A8K7A1	cDNA FLJ77346, highly similar to Homo sapiens DEAD (Asp-Glu-Ala-Asp) box polypeptide 49 (DDX49), mRNA	54 kDa	0.26	2	0	2	0	0	0	0	0	1	1	0	2.911062362	0% (0.22)
HNRPD_HUMAN	SPEE_HUMAN	SRM	P19623	Spermidine synthase (SPDSY) (EC 2.5.1.16) (Putrescine aminopropyltransferase)	34 kDa	0.25	2	0	2	0	0	0	0	2	0	0	0	2.911062362	0% (0.22)
B2R7W4_HUMAN (+1)	A8K9V7_HUMAN		A8K9V7	cDNA FLJ77207	102 kDa	0.26	2	0	2	0	0	0	0	0	0	2	0	2.911062362	0% (0.22)
F8W930_HUMAN (+1)	J3QT22_HUMAN	PPME1	J3QT22	Protein phosphatase methylesterase 1 (PME-1) (EC 3.1.1.-)	44 kDa	0.3	2	0	2	0	0	0	0	0	1	1	0	2.911062362	0% (0.22)
B2RA56_HUMAN (+1)	HEAT3_HUMAN	HEATR3	Q7Z4Q2	HEAT repeat-containing protein 3	75 kDa	0.34	2	0	2	0	0	0	0	1	1	0	0	2.911062362	0% (0.22)
RL7_HUMAN	NENF_HUMAN	NENF	Q9UMX5	Neudesin (Cell immortalization-related protein 2) (Neuron-derived neurotrophic factor) (Secreted protein of unknown function) (SPUF protein)	19 kDa	0.38	2	0	2	0	0	0	0	0	1	0	1	2.911062362	0% (0.22)
IPYR2_HUMAN	HMCS1_HUMAN	HMGCS1	Q01581	Hydroxymethylglutaryl-CoA synthase, cytoplasmic (HMG-CoA synthase) (EC 2.3.3.10) (3-hydroxy-3-methylglutaryl coenzyme A synthase)	57 kDa	0.4	2	0	2	0	0	0	0	2	0	0	0	2.911062362	0% (0.22)
B3KTM6_HUMAN (+1)	A8K0C9_HUMAN		A8K0C9	cDNA FLJ78382, highly similar to Homo sapiens C-terminal modulator protein, transcript variant 2,mRNA	12 kDa	0.41	2	0	2	0	0	0	0	2	0	0	0	2.911062362	0% (0.22)
ABCD3_HUMAN	B4DMK2_HUMAN		B4DMK2	cDNA FLJ57398	36 kDa	0.26	2	0	2	0	0	0	0	2	0	0	0	2.911062362	0% (0.22)
PML_HUMAN	A8K097_HUMAN		A8K097	cDNA FLJ78373, highly similar to Homo sapiens PCI domain containing 2, mRNA	46 kDa	0.25	2	0	2	0	0	0	0	1	0	1	0	2.911062362	0% (0.22)
J3KN69_HUMAN (+1)	MGME1_HUMAN	MGME1	Q9BQP7	Mitochondrial genome maintenance exonuclease 1 (EC 3.1.-.-)	39 kDa	0.28	2	0	2	0	0	0	0	2	0	0	1	2.911062362	0% (0.22)
CY24B_HUMAN	XCT_HUMAN	SLC7A11	Q9UPY5	Cystine/glutamate transporter (Amino acid transport system xc-) (Calcium channel blocker resistance protein CCBR1) (Solute carrier family 7 member 11) (xCT)	55 kDa	0.27	2	0	2	0	0	0	0	0	0	0	2	2.911062362	0% (0.22)
ACTN1_HUMAN	PPA6_HUMAN	ACP6	Q9NPH0	Lysophosphatidic acid phosphatase type 6 (EC 3.1.3.2) (Acid phosphatase 6, lysophosphatidic) (Acid phosphatase-like protein 1) (PACPL1)	49 kDa	0.48	2	0	2	0	0	0	0	2	0	0	0	2.911062362	0% (0.22)
C9JZR2_HUMAN (+1)	MP2K3_HUMAN	MAP2K3	P46734	Dual specificity mitogen-activated protein kinase kinase 3 (MAP kinase kinase 3) (MAPKK 3) (EC 2.7.12.2) (MAPK/ERK kinase 3) (MEK	39 kDa	0.2	2	0	2	0	0	0	0	0	2	0	0	2.911062362	0% (0.22)

				3) (Stress-activated protein kinase kinase 2) (SAPK kinase 2) (SAPKK-2) (SAPKK2)															
E5KLL9_HUMAN (+1)	ARMC9_HUMAN	ARMC9	Q7Z3E5	LisH domain-containing protein ARMC9 (Melanoma/melanocyte-specific tumor antigen KU-MEL-1) (NS21)	92 kDa	0.26	2	0	2	0	0	0	0	0	2	0	0	2.911062362	0% (0.22)
KAD4_HUMAN	AAK1_HUMAN	AAK1	Q2M2I8	AP2-associated protein kinase 1 (EC 2.7.11.1) (Adaptor-associated kinase 1)	104 kDa	0.38	2	0	2	0	0	0	0	1	1	0	0	2.911062362	0% (0.22)
Q8TBK5_HUMAN (+2)	F127A_HUMAN	FAM127A	A6ZKI3	Protein FAM127A (Mammalian retrotransposon derived protein 8C)	13 kDa	0.37	2	0	2	0	0	0	0	0	2	0	0	2.911062362	0% (0.22)
B2RWP9_HUMAN	PYRG2_HUMAN	CTPS2	Q9NRF8	CTP synthase 2 (EC 6.3.4.2) (CTP synthetase 2) (UTP--ammonia ligase 2)	66 kDa	0.23	2	0	2	0	0	0	0	0	2	0	0	2.911062362	0% (0.22)
GT251_HUMAN	GRPE2_HUMAN	GRPEL2	Q8TAA5	GrpE protein homolog 2, mitochondrial (Mt-GrpE#2)	25 kDa	0.28	2	0	2	0	0	0	0	2	0	0	0	2.911062362	0% (0.22)
RTN4_HUMAN	B4DRC8_HUMAN		B4DRC8	cDNA FLJ50047	38 kDa	0.065	2	0	2	0	0	0	0	0	0	2	0	2.911062362	0% (0.22)
COF1_HUMAN	OSBL2_HUMAN	OSBPL2	Q9H1P3	Oxysterol-binding protein-related protein 2 (ORP-2) (OSBP-related protein 2)	55 kDa	0.52	2	0	2	0	0	0	0	0	2	0	0	2.911062362	0% (0.22)
VPS35_HUMAN	A5D8W8_HUMAN	GGT7	A5D8W8	Gamma-glutamyltransferase 7	70 kDa	0.33	2	0	2	0	0	0	0	2	0	0	0	2.911062362	0% (0.22)
K6PP_HUMAN	PR38A_HUMAN	PRPF38A	Q8NAV1	Pre-mRNA-splicing factor 38A	37 kDa	0.22	2	0	2	0	0	0	0	1	0	1	0	2.911062362	0% (0.22)
RN213_HUMAN	B3KTN5_HUMAN		B3KTN5	cDNA FLJ38538 fis, clone HCHON2001407, highly similar to LanC-like protein 2	51 kDa	0.088	2	0	2	0	0	0	0	0	2	0	0	2.911062362	0% (0.22)
Q06AH7_HUMAN (+2)	ACDSB_HUMAN	ACADSB	P45954	Short/branched chain specific acyl-CoA dehydrogenase, mitochondrial (SBCAD) (EC 1.3.8.5) (2-methyl branched chain acyl-CoA dehydrogenase) (2-MEBCAD) (2-methylbutyryl-coenzyme A dehydrogenase) (2-methylbutyryl-CoA dehydrogenase)	47 kDa	0.38	9	2	7	2	0	0	0	2	4	0	1	2.842292878	0% (0.064)
B2R5W3_HUMAN	Q53GS0_HUMAN		Q53GS0	Nucleolar GTP-binding protein 1 (Fragment)	74 kDa	0.31	16	4	12	1	0	0	2	6	2	4	0	2.826017807	95% (0.023)
XPO1_HUMAN	PUR2_HUMAN	GART	P22102	Trifunctional purine biosynthetic protein adenosine-3 [Includes: Phosphoribosylamine--glycine ligase (EC 6.3.4.13) (Glycinamide ribonucleotide synthetase) (GARS) (Phosphoribosylglycinamide synthetase); Phosphoribosylformylglycinamidine cyclo-ligase (EC 6.3.3.1) (AIR synthase) (AIRS) (Phosphoribosyl-aminoimidazole synthetase); Phosphoribosylglycinamide formyltransferase (EC 2.1.2.2) (5'-phosphoribosylglycinamide transformylase) (GAR transformylase) (GART)]	108 kDa	0.24	30	8	22	0	1	1	6	9	9	4	0	2.814749232	95% (0.0032)
B4DVE1_HUMAN (+1)	Q6IBU0_HUMAN	EIF5	Q6IBU0	EIF5 protein	49 kDa	0.18	19	5	14	0	2	2	1	3	7	4	0	2.732216199	95% (0.017)
1A69_HUMAN	B4DHL7_HUMAN		B4DHL7	cDNA FLJ59515	85 kDa	0.37	19	5	14	0	1	3	1	4	6	4	0	2.732216199	95% (0.017)
SSRP1_HUMAN	A2RTY6_HUMAN	ITIH2	A2RTY6	Inter-alpha (Globulin) inhibitor H2 (Inter-alpha (Globulin) inhibitor H2, isoform CRA_b) (cDNA FLJ75038, highly similar to Homo sapiens inter-alpha (globulin) inhibitor H2 (ITIH2), mRNA)	106 kDa	0.25	12	3	9	0	1	1	1	2	4	1	2	2.70047456	95% (0.049)
ADT3_HUMAN	B4E1K0_HUMAN	KIF23	B4E1K0	Kinesin-like protein KIF23 (cDNA FLJ58416, highly similar to Kinesin-like protein KIF23)	78 kDa	0.6	12	3	9	0	2	1	0	2	1	4	2	2.70047456	95% (0.049)

NOP58_HUMAN	B4DLE7_HUMAN		B4DLE7	cDNA FLJ50983, highly similar to Homo sapiens lysocardiolipin acyltransferase (LYCAT), transcript variant 1, mRNA	39 kDa	0.33	12	3	9	1	1	0	1	3	3	1	2	2.70047456	95% (0.049)
Q53GF9_HUMAN	Q06AH7_HUMAN	TF	Q06AH7	Transferrin	77 kDa	0.28	117	34	83	1	11	10	12	27	13	30	14	2.67796886	95% (< 0.00010)
HSP74_HUMAN (+1)	B3KQ33_HUMAN		B3KQ33	cDNA FLJ32715 fis, clone TESTI2000784, highly similar to Importin-4	119 kDa	0.27	22	6	16	0	0	2	4	9	7	0	0	2.664300739	95% (0.013)
NDUS1_HUMAN	J3KN16_HUMAN	KIAA0368	J3KN16	Proteasome-associated protein ECM29 homolog	224 kDa	0.3	15	4	11	0	0	0	4	3	4	4	0	2.612695855	95% (0.037)
TOP2B_HUMAN	E9PF74_HUMAN	SLC25A19	E9PF74	Mitochondrial thiamine pyrophosphate carrier	29 kDa	0.16	15	4	11	0	0	1	3	1	2	1	7	2.612695855	95% (0.037)
FRIL_HUMAN	FADS2_HUMAN	FADS2	O95864	Fatty acid desaturase 2 (EC 1.14.19.-) (Delta(6) fatty acid desaturase) (D6D) (Delta(6) desaturase) (Delta-6 desaturase)	52 kDa	0.51	15	4	11	0	2	2	0	2	0	9	0	2.612695855	95% (0.037)
CP27A_HUMAN	COASY_HUMAN	COASY	Q13057	Bifunctional coenzyme A synthase (CoA synthase) (NBP) (POV-2) [Includes: Phosphopantetheine adenylyltransferase (EC 2.7.7.3) (Dephospho-CoA pyrophosphorylase) (Pantetheine-phosphate adenylyltransferase) (PPAT); Dephospho-CoA kinase (DPCK) (EC 2.7.1.24) (Dephosphocoenzyme A kinase) (DPCOAK)]	62 kDa	0.28	15	4	11	0	0	1	3	5	3	2	1	2.612695855	95% (0.037)
RL7A_HUMAN	GBF1_HUMAN	GBF1	Q92538	Golgi-specific brefeldin A-resistance guanine nucleotide exchange factor 1 (BFA-resistant GEF 1)	206 kDa	0.26	5	1	4	0	0	0	1	0	4	0	0	2.612533461	0% (0.15)
4F2_HUMAN (+2)	TEX2_HUMAN	TEX2	Q8IWB9	Testis-expressed sequence 2 protein (Transmembrane protein 96)	125 kDa	0.31	5	1	4	0	0	0	1	1	1	1	1	2.612533461	0% (0.15)
ERAP1_HUMAN	PARN_HUMAN	PARN	O95453	Poly(A)-specific ribonuclease PARN (EC 3.1.13.4) (Deadenylating nuclease) (Deadenylation nuclease) (Polyadenylate-specific ribonuclease)	73 kDa	0.26	5	1	4	0	0	0	1	0	1	3	0	2.612533461	0% (0.15)
PP1A_HUMAN	NPA1P_HUMAN	URB1	O60287	Nucleolar pre-ribosomal-associated protein 1 (Nucleolar protein 254 kDa) (URB1 ribosome biogenesis 1 homolog)	254 kDa	0.28	5	1	4	0	1	0	0	2	1	1	0	2.612533461	0% (0.15)
B2R4M6_HUMAN (+1)	D3DNQ1_HUMAN	TLOC1	D3DNQ1	Translocation protein 1, isoform CRA_b	41 kDa	0.68	5	1	4	1	0	0	0	0	2	0	2	2.612533461	0% (0.15)
A5YM50_HUMAN (+3)	GDE_HUMAN	AGL	P35573	Glycogen debranching enzyme (Glycogen debrancher) [Includes: 4-alpha-glucanotransferase (EC 2.4.1.25) (Oligo-1,4-1,4-glucantransferase); Amylo-alpha-1,6-glucosidase (Amylo-1,6-glucosidase) (EC 3.2.1.33) (Dextrin 6-alpha-D-glucosidase)]	175 kDa	0.44	5	1	4	0	0	0	1	2	2	0	0	2.612533461	0% (0.15)
PRDX5_HUMAN	B4DG11_HUMAN		B4DG11	cDNA FLJ55382, highly similar to Hsp70-binding protein 1	44 kDa	0.45	5	1	4	0	0	0	1	3	1	0	0	2.612533461	0% (0.15)
1433Z_HUMAN (+1)	RRF2M_HUMAN	GFM2	Q969S9	Ribosome-releasing factor 2, mitochondrial (RRF2mt) (Elongation factor G 2, mitochondrial) (EF-G2mt) (mEF-G 2) (Elongation factor G2) (hEFG2)	87 kDa	0.48	5	1	4	0	1	0	0	2	0	2	0	2.612533461	0% (0.15)
NAMPT_HUMAN	SYRM_HUMAN	RARS2	Q5T160	Probable arginine--tRNA ligase, mitochondrial (EC 6.1.1.19) (Arginyl-tRNA synthetase) (ArgRS)	66 kDa	0.29	5	1	4	0	0	1	0	2	0	0	2	2.612533461	0% (0.15)
B7Z1R5_HUMAN (+1)	Q5LJA9_HUMAN	UCHL5	Q5LJA9	Ubiquitin carboxyl-terminal hydrolase isozyme L5 (Fragment)	42 kDa	0.34	5	1	4	0	0	1	0	1	4	0	0	2.612533461	0% (0.15)

E1NZA1_HUMAN	PPIG_HUMAN	PPIG	Q13427	Peptidyl-prolyl cis-trans isomerase G (PPIase G) (Peptidyl-prolyl isomerase G) (EC 5.2.1.8) (CASP10) (Clk-associating RS-cyclophilin) (CARS-Cyp) (CARS-cyclophilin) (SR-cyclophilin) (SR-cyp) (SRcyp) (Cyclophilin G) (Rotamase G)	89 kDa	0.21	5	1	4	0	0	0	1	0	0	4	0	2.612533461	0% (0.15)
ANXA3_HUMAN	B2RDR4_HUMAN		B2RDR4	cDNA, FLJ96732, highly similar to Homo sapiens testis derived transcript (3 LIM domains) (TES), transcript variant 1, mRNA	48 kDa	0.51	5	1	4	1	0	0	0	0	4	0	0	2.612533461	0% (0.15)
PRDX6_HUMAN	B9A062_HUMAN	MTHFD2	B9A062	Bifunctional methylenetetrahydrofolate dehydrogenase/cyclohydrolase, mitochondrial	24 kDa	0.56	5	1	4	0	1	0	0	2	1	0	1	2.612533461	0% (0.15)
ACADM_HUMAN (+2)	B3KS61_HUMAN		B3KS61	cDNA FLJ35571 fis, clone SPLEN2005806, highly similar to Protein-S-isoprenylcysteineO-methyltransferase (EC 2.1.1.100)	22 kDa	0.27	5	1	4	1	0	0	0	3	0	0	1	2.612533461	0% (0.15)
DDB1_HUMAN	SFXN2_HUMAN	SFXN2	Q96NB2	Sideroflexin-2	36 kDa	0.2	5	1	4	1	0	0	0	1	2	0	1	2.612533461	0% (0.15)
DRA_HUMAN	B7Z4A7_HUMAN	EVI2B	B7Z4A7	Protein EVI2B (cDNA FLJ55224, highly similar to EVI2B protein)	51 kDa	0.26	5	1	4	0	0	1	0	0	0	3	1	2.612533461	0% (0.15)
Q8TA92_HUMAN	FA10_HUMAN	F10	P00742	Coagulation factor X (EC 3.4.21.6) (Stuart factor) (Stuart-Prower factor) [Cleaved into: Factor X light chain; Factor X heavy chain; Activated factor Xa heavy chain]	55 kDa	0.15	5	1	4	0	0	0	1	0	4	0	0	2.612533461	0% (0.15)
A8K8D9_HUMAN (+3)	TIGAR_HUMAN	TIGAR	Q9NQ88	Fructose-2,6-bisphosphatase TIGAR (EC 3.1.3.46) (TP53-induced glycolysis and apoptosis regulator)	30 kDa	0.35	5	1	4	0	0	1	0	0	4	0	0	2.612533461	0% (0.15)
RL10A_HUMAN	DDX46_HUMAN	DDX46	Q7L014	Probable ATP-dependent RNA helicase DDX46 (EC 3.6.4.13) (DEAD box protein 46) (PRP5 homolog)	117 kDa	0.41	38	11	27	0	2	2	6	5	4	14	4	2.582589337	95% (0.0024)
J3KN36_HUMAN (+3)	EF1A1_HUMAN	EEF1A1	P68104	Elongation factor 1-alpha 1 (EF-1-alpha-1) (Elongation factor Tu) (EF-Tu) (Eukaryotic elongation factor 1 A-1) (eEF1A-1) (Leukocyte receptor cluster member 7)	50 kDa	0.19	317	96	222	13	28	24	32	26	137	40	22	2.575257241	95% (< 0.00010)
B4DZC0_HUMAN (+2)	TFB2M_HUMAN	TFB2M	Q9H5Q4	Dimethyladenosine transferase 2, mitochondrial (EC 2.1.1.-) (Hepatitis C virus NS5A-transactivated protein 5) (HCV NS5A-transactivated protein 5) (Mitochondrial 12S rRNA dimethylase 2) (Mitochondrial transcription factor B2) (h-mtTFB) (h-mtTFB2) (hTFB2M) (mtTFB2) (S-adenosylmethionine-6-N', N'-adenosyl(rRNA) dimethyltransferase 2)	45 kDa	0.25	18	5	13	1	1	2	1	6	2	1	4	2.55301775	95% (0.028)
SF3B1_HUMAN	Q0VGA5_HUMAN	SARS	Q0VGA5	SARS protein	58 kDa	0.15	31	9	22	0	0	1	8	7	9	6	0	2.540106912	95% (0.0062)
ACOX1_HUMAN	A2MG_HUMAN	A2M	P01023	Alpha-2-macroglobulin (Alpha-2-M) (C3 and PZP-like alpha-2-macroglobulin domain-containing protein 5)	163 kDa	0.31	31	9	22	0	1	7	1	2	1	6	12	2.540106912	95% (0.0062)
RS9_HUMAN	B4DIA6_HUMAN		B4DIA6	cDNA FLJ57064, highly similar to Alpha crystallin B chain (Alpha(B)-crystallin)	18 kDa	0.36	34	10	24	4	0	0	7	6	9	0	9	2.513440927	95% (0.0048)
TOM40_HUMAN	ATPF1_HUMAN	ATPAF1	Q5TC12	ATP synthase mitochondrial F1 complex assembly factor 1 (ATP11 homolog)	36 kDa	0.27	21	6	15	1	2	2	1	10	2	3	0	2.509812412	95% (0.021)
TCPB_HUMAN	GNL3_HUMAN	GNL3	Q9BVP2	Guanine nucleotide-binding protein-like 3 (E2-induced gene 3 protein) (Novel	62 kDa	0.34	8	2	6	0	0	1	1	2	3	1	0	2.497736596	0% (0.11)

				nucleolar protein 47) (NNP47) (Nucleolar GTP-binding protein 3) (Nucleostemin)															
ARL8B_HUMAN (+1)	TXD17_HUMAN	TXNDC17	Q9BRA2	Thioredoxin domain-containing protein 17 (14 kDa thioredoxin-related protein) (TRP14) (Protein 42-9-9) (Thioredoxin-like protein 5)	14 kDa	0.46	8	2	6	0	0	1	1	0	6	0	0	2.497736596	0% (0.11)
B2R5U1_HUMAN (+2)	D6RGZ2_HUMAN	THOC3	D6RGZ2	THO complex subunit 3	11 kDa	0.3	8	2	6	0	0	0	2	3	0	2	1	2.497736596	0% (0.11)
B4DH07_HUMAN	ATG3_HUMAN	ATG3	Q9NT62	Ubiquitin-like-conjugating enzyme ATG3 (EC 6.3.2.-) (Autophagy-related protein 3) (APG3-like) (hApg3) (Protein PC3-96)	36 kDa	0.24	8	2	6	0	0	1	1	5	1	0	0	2.497736596	0% (0.11)
B2R5M9_HUMAN (+2)	J3KN55_HUMAN	MMAB	J3KN55	Cob(I)yrinic acid a,c-diamide adenosyltransferase, mitochondrial	18 kDa	0.3	8	2	6	1	0	1	0	2	0	2	2	2.497736596	0% (0.11)
RS2_HUMAN	EMD_HUMAN	EMD	P50402	Emerin	29 kDa	0.33	8	2	6	1	0	0	1	2	0	2	2	2.497736596	0% (0.11)
A8MXP9_HUMAN	TIM22_HUMAN	TIMM22	Q9Y584	Mitochondrial import inner membrane translocase subunit Tim22 (Testis-expressed sequence 4)	20 kDa	0.2	8	2	6	0	0	0	2	1	3	1	1	2.497736596	0% (0.11)
SRPRB_HUMAN	B4DEX8_HUMAN	MAT2A	B4DEX8	S-adenosylmethionine synthase (EC 2.5.1.6)	40 kDa	0.49	8	2	6	0	0	0	2	2	0	4	0	2.497736596	0% (0.11)
IF4A3_HUMAN	MIF_HUMAN	MIF	P14174	Macrophage migration inhibitory factor (MIF) (EC 5.3.2.1) (Glycosylation-inhibiting factor) (GIF) (L-dopachrome isomerase) (L-dopachrome tautomerase) (EC 5.3.3.12) (Phenylpyruvate tautomerase)	12 kDa	0.37	27	8	19	1	3	4	0	4	7	5	3	2.451449961	95% (0.012)
LETM1_HUMAN	PDPR_HUMAN	PDPR	Q8NCN5	Pyruvate dehydrogenase phosphatase regulatory subunit, mitochondrial (PDPr)	99 kDa	0.25	47	14	32	2	5	6	1	21	2	1	9	2.441795579	95% (0.0017)
B2RE46_HUMAN (+1)	APT_HUMAN	APRT	P07741	Adenine phosphoribosyltransferase (APRT) (EC 2.4.2.7)	20 kDa	0.21	46	14	32	1	3	5	5	9	15	4	4	2.441795579	95% (0.0017)
PSMD2_HUMAN (+1)	RRS1_HUMAN	RRS1	Q15050	Ribosome biogenesis regulatory protein homolog	41 kDa	0.2	11	3	8	1	0	0	2	4	0	2	2	2.436978568	0% (0.080)
TPR_HUMAN	TR112_HUMAN	TRMT112	Q9UI30	tRNA methyltransferase 112 homolog (TRM112-like protein)	14 kDa	0.13	11	3	8	0	0	0	3	5	2	1	0	2.436978568	0% (0.080)
B5BTY4_HUMAN (+2)	COIL_HUMAN	COIL	P38432	Coilin (p80)	63 kDa	0.26	11	3	8	0	1	1	1	4	1	2	1	2.436978568	0% (0.080)
ACSL3_HUMAN (+2)	DDX47_HUMAN	DDX47	Q9H0S4	Probable ATP-dependent RNA helicase DDX47 (EC 3.6.4.13) (DEAD box protein 47)	51 kDa	0.23	30	9	21	0	1	5	3	4	7	6	4	2.430820025	95% (0.0095)
B2R5M8_HUMAN (+1)	B3KN29_HUMAN		B3KN29	cDNA FLJ13371 fis, clone PLACE1000656, highly similar to PRA1 family protein 2	19 kDa	0.37	30	9	21	4	0	0	5	1	12	5	3	2.430820025	95% (0.0095)
EFGM_HUMAN	UBP5_HUMAN	USP5	P45974	Ubiquitin carboxyl-terminal hydrolase 5 (EC 3.4.19.12) (Deubiquitinating enzyme 5) (Isopeptidase T) (Ubiquitin thioesterase 5) (Ubiquitin-specific-processing protease 5)	96 kDa	0.26	34	10	23	0	0	3	7	6	15	3	0	2.413863977	95% (0.0073)
NDUA9_HUMAN (+1)	THOC2_HUMAN	THOC2	Q8NI27	THO complex subunit 2 (Tho2) (hTREX120)	183 kDa	0.42	36	11	25	1	2	4	3	7	1	12	5	2.399682091	95% (0.0056)
P5CS_HUMAN	Q5W0H4_HUMAN	TPT1	Q5W0H4	Translationally-controlled tumor protein	22 kDa	0.29	35	11	25	2	4	3	1	8	8	6	3	2.399682091	95% (0.0056)
ACAD9_HUMAN	IF2P_HUMAN	EIF5B	O60841	Eukaryotic translation initiation factor 5B (eIF-5B) (Translation initiation factor IF-2)	139 kDa	0.25	14	4	10	0	2	1	1	3	2	4	1	2.399380041	0% (0.059)
BAP31_HUMAN (+1)	D7PBN3_HUMAN		D7PBN3	ESRP1/RAF1 fusion protein	119 kDa	0.37	14	4	10	0	3	1	0	4	3	3	0	2.399380041	0% (0.059)
SUCB2_HUMAN	GAK_HUMAN	GAK	O14976	Cyclin-G-associated kinase (EC 2.7.11.1)	143 kDa	0.28	17	5	12	0	1	2	3	3	6	3	0	2.373824457	95% (0.044)
HM13_HUMAN	LAS1L_HUMAN	LAS1L	Q9Y4W2	Ribosomal biogenesis protein LAS1L (Protein LAS1 homolog)	83 kDa	0.23	17	5	12	2	0	0	3	6	2	0	5	2.373824457	95% (0.044)
FKBP4_HUMAN	B7Z6C2_HUMAN		B7Z6C2	cDNA FLJ50663, highly similar to	64 kDa	0.35	17	5	12	0	3	2	0	1	10	1	0	2.373824457	95% (0.044)

				Phosphoglucomutase-1 (EC 5.4.2.2)															
RS8_HUMAN	A5PLK7_HUMAN	RCC2	A5PLK7	RCC2 protein (Fragment)	50 kDa	0.46	20	6	14	0	1	1	4	8	4	2	0	2.35532853	95% (0.033)
A8K4W0_HUMAN (+3)	Q6URC4_HUMAN		Q6URC4	Diaphanous 1	141 kDa	0.35	22	7	16	0	3	3	1	7	2	5	1	2.341325111	95% (0.025)
LDHB_HUMAN	A8K607_HUMAN		A8K607	cDNA FLJ76855, highly similar to Homo sapiens exportin 7 (XPO7), mRNA	124 kDa	0.42	23	7	16	0	2	3	2	1	9	6	0	2.341325111	95% (0.025)
B4DLV7_HUMAN (+2)	Q53GX7_HUMAN		Q53GX7	Threonyl-tRNA synthetase variant (Fragment)	83 kDa	0.32	60	19	41	0	6	5	8	15	13	9	4	2.336778412	95% (0.00071)
RAB21_HUMAN	BAZ1B_HUMAN	BAZ1B	Q9UIG0	Tyrosine-protein kinase BAZ1B (EC 2.7.10.2) (Bromodomain adjacent to zinc finger domain protein 1B) (Williams syndrome transcription factor) (Williams-Beuren syndrome chromosomal region 10 protein) (Williams-Beuren syndrome chromosomal region 9 protein) (hWALp2)	171 kDa	0.4	27	8	18	2	2	0	5	14	0	3	1	2.330357173	95% (0.019)
EF1G_HUMAN	PSME3_HUMAN	PSME3	P61289	Proteasome activator complex subunit 3 (11S regulator complex subunit gamma) (REG-gamma) (Activator of multicatalytic protease subunit 3) (Ki nuclear autoantigen) (Proteasome activator 28 subunit gamma) (PA28g) (PA28gamma)	30 kDa	0.27	26	8	18	0	1	1	6	4	6	8	1	2.330357173	95% (0.019)
VDAC3_HUMAN	B2R7G6_HUMAN		B2R7G6	cDNA, FLJ93437, highly similar to Homo sapiens histidyl-tRNA synthetase-like (HARSL), mRNA	57 kDa	0.45	26	8	18	4	3	1	1	7	0	3	8	2.330357173	95% (0.019)
COPB_HUMAN	ACOX3_HUMAN	ACOX3	O15254	Peroxisomal acyl-coenzyme A oxidase 3 (EC 1.3.3.6) (Branched-chain acyl-CoA oxidase) (BRCACox) (Pristanoyl-CoA oxidase)	78 kDa	0.27	25	8	18	7	0	1	0	1	8	3	6	2.330357173	95% (0.019)
FBRL_HUMAN	ERGI1_HUMAN	ERGIC1	Q969X5	Endoplasmic reticulum-Golgi intermediate compartment protein 1 (ER-Golgi intermediate compartment 32 kDa protein) (ERGIC-32)	33 kDa	0.3	29	9	20	7	0	0	2	2	6	7	5	2.321536283	95% (0.014)
APMAP_HUMAN	LBP_HUMAN	LBP	P18428	Lipopolysaccharide-binding protein (LBP)	53 kDa	0.3	28	9	20	0	2	1	6	0	0	4	15	2.321536283	95% (0.014)
S10AB_HUMAN	CLUS_HUMAN	CLU	P10909	Clusterin (Aging-associated gene 4 protein) (Apolipoprotein J) (Apo-J) (Complement cytolysis inhibitor) (CLI) (Complement-associated protein SP-40,40) (Ku70-binding protein 1) (NA1/NA2) (Testosterone-repressed prostate message 2) (TRPM-2) [Cleaved into: Clusterin beta chain (ApoJalpha) (Complement cytolysis inhibitor a chain); Clusterin alpha chain (ApoJbeta) (Complement cytolysis inhibitor b chain)]	52 kDa	0.41	28	9	20	3	1	4	1	1	8	3	7	2.321536283	95% (0.014)
B2RB23_HUMAN (+2)	Q4LE36_HUMAN	ACLY	Q4LE36	ACLY variant protein (Fragment)	125 kDa	0.27	96	31	64	0	12	9	10	28	24	11	2	2.266435984	95% (< 0.00010)
Q53HQ0_HUMAN	EFGM_HUMAN	GFM1	Q96RP9	Elongation factor G, mitochondrial (EF-Gmt) (Elongation factor G 1, mitochondrial) (mEF-G 1) (Elongation factor G1) (hEFG1)	83 kDa	0.42	102	33	68	4	12	10	8	37	6	10	15	2.26498698	95% (< 0.00010)
MX1_HUMAN	B4DH07_HUMAN		B4DH07	cDNA FLJ53321, highly similar to Homo sapiens pitrilysin metallopeptidase 1 (PITRM1), mRNA	116 kDa	0.31	111	37	75	12	7	7	11	28	5	19	23	2.233244959	95% (< 0.00010)

AP2A2_HUMAN	B5BTZ6_HUMA N	STAT3	B5BTZ6	Signal transducer and activator of transcription	88 kDa	0.22	60	20	41	3	7	3	7	12	21	9	0	2.2267837 52	95% (0.0012)
B2R6J2_HUMA N (+2)	SYYC_HUMAN	YARS	P54577	Tyrosine--tRNA ligase, cytoplasmic (EC 6.1.1.1) (Tyrosyl-tRNA synthetase) (TyrRS) [Cleaved into: Tyrosine--tRNA ligase, cytoplasmic, N-terminally processed]	59 kDa	0.24	28	9	19	0	3	3	3	7	8	3	1	2.2122556 85	95% (0.022)
ATLA3_HUMA N (+1)	UMPS_HUMAN	UMPS	P11172	Uridine 5'-monophosphate synthase (UMP synthase) [Includes: Orotate phosphoribosyltransferase (OPRT) (OPRTase) (EC 2.4.2.10); Orotidine 5'-phosphate decarboxylase (ODC) (EC 4.1.1.23) (OMPdecase)]	52 kDa	0.22	25	8	17	0	2	5	1	12	2	3	1	2.2092678 7	95% (0.029)
F8W1R7_HUMA N (+2)	Q53FW8_HUMA N		Q53FW8	TAP binding protein-like variant (Fragment)	50 kDa	0.61	22	7	15	2	0	0	6	0	11	0	5	2.2055643 86	95% (0.038)
B4DJX1_HUMA N (+1)	Q53G19_HUMA N		Q53G19	Mitochondrial ribosomal protein L11 isoform a variant (Fragment)	21 kDa	0.22	22	7	15	3	3	0	1	4	0	5	6	2.2055643 86	95% (0.038)
NUMA1_HUMA N (+1)	DHTK1_HUMA N	DHTKD1	Q96HY7	Probable 2-oxoglutarate dehydrogenase E1 component DHKTD1, mitochondrial (EC 1.2.4.2) (Dehydrogenase E1 and transketolase domain-containing protein 1)	103 kDa	0.16	16	5	11	2	2	1	0	7	1	1	2	2.1946363 2	0% (0.068)
Q32Q12_HUMA N	B5MCF9_HUM AN	PES1	B5MCF9	Pescadillo homolog	66 kDa	0.32	16	5	11	0	2	2	1	5	0	4	2	2.1946363 2	0% (0.068)
NNTM_HUMAN (+1)	RFA1_HUMAN	RPA1	P27694	Replication protein A 70 kDa DNA-binding subunit (RP-A p70) (Replication factor A protein 1) (RF-A protein 1) (Single-stranded DNA-binding protein) [Cleaved into: Replication protein A 70 kDa DNA-binding subunit, N-terminally processed]	68 kDa	0.18	16	5	11	0	1	2	2	6	3	2	0	2.1946363 2	0% (0.068)
DHC24_HUMA N	ANT3_HUMAN	SERPINC1	P01008	Antithrombin-III (ATIII) (Serpin C1)	53 kDa	0.23	13	4	9	0	1	1	2	1	4	1	3	2.1860703 65	0% (0.093)
A7BI36_HUMA N	DAZP1_HUMA N	DAZAP1	Q96EP5	DAZ-associated protein 1 (Deleted in azoospermia-associated protein 1)	43 kDa	0.19	13	4	9	0	0	3	2	2	0	3	4	2.1860703 65	0% (0.093)
SC22B_HUMAN	RTN1_HUMAN	RTN1	Q16799	Reticulon-1 (Neuroendocrine-specific protein)	84 kDa	0.34	13	4	9	0	2	1	0	0	2	1	5	2.1860703 65	0% (0.093)
Q53HU0_HUMA N (+1)	B3KRC6_HUMA N		B3KRC6	cDNA FLJ34004 fis, clone FCBBF1000232, highly similar to Cytochrome P450 51A1 (EC 1.14.13.70)	51 kDa	0.37	13	4	9	4	0	0	0	4	1	1	3	2.1860703 65	0% (0.093)
B4DLW8_HUM AN (+4)	Q59E99_HUMA N		Q59E99	Thrombospondin 1 variant (Fragment)	135 kDa	0.31	10	3	7	0	2	1	0	3	2	1	1	2.1734901 57	0% (0.13)
MCU_HUMAN	J3KMX1_HUMA N	NUP88	J3KMX1	Nuclear pore complex protein Nup88 (Fragment)	79 kDa	0.28	10	3	7	0	1	0	2	1	1	5	0	2.1734901 57	0% (0.13)
CAN1_HUMAN	B4DQJ1_HUMA N	SNRNP40	B4DQJ1	U5 small nuclear ribonucleoprotein 40 kDa protein (cDNA FLJ56825, highly similar to WD repeat protein 57)	45 kDa	0.24	10	3	7	2	1	0	0	1	0	3	3	2.1734901 57	0% (0.13)
MCCB_HUMAN	MSLN_HUMAN	MSLN	Q13421	Mesothelin (CAK1 antigen) (Pre-pro-megakaryocyte-potentiating factor) [Cleaved into: Megakaryocyte-potentiating factor (MPF); Mesothelin, cleaved form]	69 kDa	0.25	10	3	7	2	0	0	1	4	0	2	1	2.1734901 57	0% (0.13)
Q4LE36_HUMA N	PURA2_HUMA N	ADSS	P30520	Adenylosuccinate synthetase isozyme 2 (AMPSase 2) (AdSS 2) (EC 6.3.4.4) (Adenylosuccinate synthetase, acidic isozyme) (Adenylosuccinate synthetase, liver isozyme) (L-type adenylosuccinate synthetase) (IMP--aspartate ligase 2)	50 kDa	0.25	10	3	7	0	0	0	3	2	4	1	0	2.1734901 57	0% (0.13)
A8K916_HUMA N (+2)	B4DTS6_HUMA N		B4DTS6	cDNA FLJ54117, highly similar to CD97 antigen	84 kDa	0.19	10	3	7	0	2	1	0	0	0	2	5	2.1734901 57	0% (0.13)

GPX1_HUMAN	E9PGN7_HUMAN	SERPING1	E9PGN7	Plasma protease C1 inhibitor	59 kDa	0.41	10	3	7	0	0	0	3	1	0	2	4	2.173490157	0% (0.13)
GBLP_HUMAN	B3KY30_HUMAN		B3KY30	cDNA FLJ46699 fis, clone TRACH3013700, highly similar to Stress 70 protein chaperonemicrosome-associated 60 kDa protein	29 kDa	0.44	10	3	7	1	1	1	0	1	0	2	4	2.173490157	0% (0.13)
PNPT1_HUMAN	PORED_HUMAN	SRD5A3	Q9H8P0	Polyprenol reductase (EC 1.3.1.94) (3-oxo-5-alpha-steroid 4-dehydrogenase 3) (EC 1.3.1.22) (Steroid 5-alpha-reductase 2-like) (Steroid 5-alpha-reductase 3) (S5AR 3) (SR type 3)	37 kDa	0.26	10	3	7	3	0	0	0	0	4	1	2	2.173490157	0% (0.13)
Q8IWP6_HUMAN	RSF1_HUMAN	RSF1	Q96T23	Remodeling and spacing factor 1 (Rsf-1) (HBV pX-associated protein 8) (Hepatitis B virus X-associated protein) (p325 subunit of RSF chromatin-remodeling complex)	164 kDa	0.49	7	2	5	1	0	1	0	3	0	0	2	2.153190229	0% (0.18)
OAT_HUMAN	RFC1_HUMAN	RFC1	P35251	Replication factor C subunit 1 (Activator 1 140 kDa subunit) (A1 140 kDa subunit) (Activator 1 large subunit) (Activator 1 subunit 1) (DNA-binding protein PO-GA) (Replication factor C 140 kDa subunit) (RF-C 140 kDa subunit) (RFC140) (Replication factor C large subunit)	128 kDa	0.29	7	2	5	0	0	1	1	1	1	3	0	2.153190229	0% (0.18)
VPP3_HUMAN	E41L5_HUMAN	EPB41L5	Q9HCM4	Band 4.1-like protein 5	82 kDa	0.25	7	2	5	1	0	0	1	0	5	0	0	2.153190229	0% (0.18)
DHB11_HUMAN	B7Z787_HUMAN		B7Z787	cDNA FLJ54765	48 kDa	0.44	7	2	5	0	0	0	2	2	1	1	1	2.153190229	0% (0.18)
B1AZV3_HUMAN (+1)	RBM28_HUMAN	RBM28	Q9NW13	RNA-binding protein 28 (RNA-binding motif protein 28)	86 kDa	0.19	7	2	5	0	0	0	2	0	1	4	0	2.153190229	0% (0.18)
TACD2_HUMAN	RT15_HUMAN	MRPS15	P82914	28S ribosomal protein S15, mitochondrial (MRP-S15) (S15mt)	30 kDa	0.23	7	2	5	1	0	0	1	2	1	1	1	2.153190229	0% (0.18)
A5YM53_HUMAN (+2)	D6W4Z6_HUMAN	hCG_23833	D6W4Z6	HCG23833, isoform CRA_b	51 kDa	0.2	7	2	5	0	1	0	1	3	0	2	0	2.153190229	0% (0.18)
HS105_HUMAN	B2R7C2_HUMAN		B2R7C2	cDNA, FLJ93375, highly similar to Homo sapiens ZW10, kinetochore associated, homolog (Drosophila) (ZW10), mRNA	89 kDa	0.25	7	2	5	0	0	2	0	2	0	2	1	2.153190229	0% (0.18)
B3KX11_HUMAN (+1)	ASGL1_HUMAN	ASRGL1	Q7L266	Isoaspartyl peptidase/L-asparaginase (EC 3.4.19.5) (EC 3.5.1.1) (Asparaginase-like protein 1) (Beta-aspartyl-peptidase) (Isoaspartyl dipeptidase) (L-asparagine amidohydrolase) [Cleaved into: Isoaspartyl peptidase/L-asparaginase alpha chain; Isoaspartyl peptidase/L-asparaginase beta chain]	32 kDa	0.31	7	2	5	0	0	0	2	1	3	1	0	2.153190229	0% (0.18)
PLOD2_HUMAN	C9JLU1_HUMAN	POLR2H	C9JLU1	DNA-directed RNA polymerases I, II, and III subunit RPABC3 (Fragment)	17 kDa	0.28	7	2	5	0	0	0	2	1	2	2	0	2.153190229	0% (0.18)
F2Z2U8_HUMAN (+2)	CO3_HUMAN	C3	P01024	Complement C3 (C3 and PZP-like alpha-2-macroglobulin domain-containing protein 1) [Cleaved into: Complement C3 beta chain; Complement C3 alpha chain; C3a anaphylatoxin; Acylation stimulating protein (ASP) (C3adesArg); Complement C3b alpha' chain; Complement C3c alpha' chain fragment 1; Complement C3dg fragment; Complement C3g fragment; Complement C3d fragment; Complement C3f fragment; Complement C3c alpha' chain fragment 2]	187 kDa	0.18	156	54	104	18	11	11	15	5	14	38	47	2.134539838	95% (< 0.00010)

PLAK_HUMAN	SYTM_HUMAN	TARS2	Q9BW92	Threonine--tRNA ligase, mitochondrial (EC 6.1.1.3) (Threonyl-tRNA synthetase) (ThrRS) (Threonyl-tRNA synthetase-like 1)	81 kDa	0.34	33	11	22	4	3	2	2	11	1	3	7	2.12534096	95% (0.018)
ABHDA_HUMAN	EIF3M_HUMAN	EIF3M	Q7L2H7	Eukaryotic translation initiation factor 3 subunit M (eIF3m) (Fetal lung protein B5) (hFL-B5) (PCI domain-containing protein 1)	43 kDa	0.31	33	11	22	0	5	1	5	6	10	4	2	2.12534096	95% (0.018)
ABHDB_HUMAN	MTOR_HUMAN	MTOR	P42345	Serine/threonine-protein kinase mTOR (EC 2.7.11.1) (FK506-binding protein 12-rapamycin complex-associated protein 1) (FKBP12-rapamycin complex-associated protein) (Mammalian target of rapamycin) (mTOR) (Mechanistic target of rapamycin) (Rapamycin and FKBP12 target 1) (Rapamycin target protein 1)	289 kDa	0.23	4	1	3	1	0	0	0	1	0	2	0	2.114877617	0% (0.27)
ANXA7_HUMAN (+6)	B3KMQ6_HUMAN		B3KMQ6	cDNA FLJ12172 fis, clone MAMMA1000684, highly similar to Opioid growth factor receptor	73 kDa	0.23	4	1	3	0	0	0	1	0	3	0	0	2.114877617	0% (0.27)
B4DV28_HUMAN (+1)	ATS1_HUMAN	ADAMTS1	Q9UHI8	A disintegrin and metalloproteinase with thrombospondin motifs 1 (ADAM-TS 1) (ADAM-TS1) (ADAMTS-1) (EC 3.4.24.-) (METH-1)	105 kDa	0.27	4	1	3	0	0	0	1	3	0	0	0	2.114877617	0% (0.27)
CY1_HUMAN (+1)	MTER1_HUMAN	MTERFD1	Q96E29	mTERF domain-containing protein 1, mitochondrial (Mitochondrial transcription termination factor 3) (mTERF3)	48 kDa	0.28	4	1	3	0	1	0	0	2	0	1	0	2.114877617	0% (0.27)
GSTP1_HUMAN	COQ6_HUMAN	COQ6	Q9Y2Z9	Ubiquinone biosynthesis monooxygenase COQ6 (EC 1.14.13.-) (Coenzyme Q10 monooxygenase 6)	51 kDa	0.49	4	1	3	0	0	0	1	3	0	0	0	2.114877617	0% (0.27)
B3KPS3_HUMAN (+2)	CSDE1_HUMAN	CSDE1	O75534	Cold shock domain-containing protein E1 (N-ras upstream gene protein) (Protein UNR)	89 kDa	0.47	4	1	3	0	0	0	1	0	2	1	0	2.114877617	0% (0.27)
H0Y8C6_HUMAN (+1)	A8KAJ7_HUMAN		A8KAJ7	cDNA FLJ77222, highly similar to Homo sapiens UDP-N-acetyl-alpha-D-galactosamine:polypeptide N-acetylgalactosaminyltransferase 1 (GalNAc-T1) (GALNT1), mRNA	64 kDa	0.2	4	1	3	0	0	0	1	0	3	0	0	2.114877617	0% (0.27)
PAI2_HUMAN	AATC_HUMAN	GOT1	P17174	Aspartate aminotransferase, cytoplasmic (cAspAT) (EC 2.6.1.1) (EC 2.6.1.3) (Cysteine aminotransferase, cytoplasmic) (Cysteine transaminase, cytoplasmic) (cCAT) (Glutamate oxaloacetate transaminase 1) (Transaminase A)	46 kDa	0.26	4	1	3	0	0	0	1	3	1	0	0	2.114877617	0% (0.27)
PO210_HUMAN	Q6NUR1_HUMAN	NCAPG	Q6NUR1	Non-SMC condensin I complex, subunit G	114 kDa	0.12	4	1	3	0	0	1	0	2	1	0	0	2.114877617	0% (0.27)
SGPL1_HUMAN	B4DM85_HUMAN		B4DM85	cDNA FLJ56002, highly similar to Kinesin-like protein KIF2	84 kDa	0.22	4	1	3	0	1	0	0	1	2	0	0	2.114877617	0% (0.27)
A8K8U1_HUMAN (+1)	RENT2_HUMAN	UPF2	Q9HAU5	Regulator of nonsense transcripts 2 (Nonsense mRNA reducing factor 2) (Up-frameshift suppressor 2 homolog) (hUpf2)	148 kDa	0.18	4	1	3	0	0	1	0	3	0	0	0	2.114877617	0% (0.27)
F213A_HUMAN	RCL1_HUMAN	RCL1	Q9Y2P8	RNA 3'-terminal phosphate cyclase-like protein	41 kDa	0.36	4	1	3	0	0	0	1	0	0	1	2	2.114877617	0% (0.27)
A8KAP9_HUMAN (+1)	FA50A_HUMAN	FAM50A	Q14320	Protein FAM50A (Protein HXC-26) (Protein XAP-5)	40 kDa	0.25	4	1	3	0	0	0	1	0	3	0	0	2.114877617	0% (0.27)
B7Z6B8_HUMAN (+1)	OSBP1_HUMAN	OSBP	P22059	Oxysterol-binding protein 1	89 kDa	0.31	4	1	3	1	1	0	0	0	2	1	0	2.114877617	0% (0.27)
COX41_HUMAN	PSB5_HUMAN	PSMB5	P28074	Proteasome subunit beta type-5 (EC 3.4.25.1) (Macropain epsilon chain) (Multicatalytic	28 kDa	0.49	3	1	3	0	0	0	1	1	1	0	0	2.114877617	0% (0.27)

				endopeptidase complex epsilon chain) (Proteasome chain 6) (Proteasome epsilon chain) (Proteasome subunit MB1) (Proteasome subunit X)															
DSRAD_HUMAN (+1)	A8MVT4_HUMAN	MRPL23	A8MVT4	39S ribosomal protein L23, mitochondrial	19 kDa	0.12	4	1	3	0	0	0	1	1	0	1	1	2.114877617	0% (0.27)
ILVBL_HUMAN	B7Z377_HUMAN		B7Z377	cDNA FLJ57937, highly similar to DNA-directed RNA polymerase II 33 kDa polypeptide (EC 2.7.7.6)	17 kDa	0.19	4	1	3	0	0	0	1	0	0	2	1	2.114877617	0% (0.27)
MBB1A_HUMAN	RM01_HUMAN	MRPL1	Q9BYD6	39S ribosomal protein L1, mitochondrial (L1mt) (MRP-L1)	37 kDa	0.13	27	9	18	5	1	3	0	8	3	3	4	2.102978232	95% (0.032)
PLOD3_HUMAN	B2R739_HUMAN		B2R739	cDNA, FLJ93269, highly similar to Homo sapiens mitochondrial ribosomal protein L15 (MRPL15), nuclear gene encoding mitochondrial protein, mRNA	33 kDa	0.26	27	9	18	4	1	2	2	8	2	3	5	2.102978232	95% (0.032)
RL10_HUMAN	J3KQ32_HUMAN	OLA1	J3KQ32	Obg-like ATPase 1	47 kDa	0.15	27	9	18	0	2	2	5	3	6	4	5	2.102978232	95% (0.032)
SP16H_HUMAN	RBP2_HUMAN	RANBP2	P49792	E3 SUMO-protein ligase RanBP2 (EC 6.3.2.-) (358 kDa nucleoporin) (Nuclear pore complex protein Nup358) (Nucleoporin Nup358) (Ran-binding protein 2) (RanBP2) (p270)	358 kDa	0.19	50	17	33	2	4	4	7	7	6	15	5	2.101719296	95% (0.0054)
CPT2_HUMAN	BDH_HUMAN	BDH1	Q02338	D-beta-hydroxybutyrate dehydrogenase, mitochondrial (BDH) (EC 1.1.1.30) (3-hydroxybutyrate dehydrogenase)	38 kDa	0.19	49	17	33	7	1	0	8	13	12	7	1	2.101719296	95% (0.0054)
XPO2_HUMAN	PLPL6_HUMAN	PNPLA6	Q8IY17	Neuropathy target esterase (EC 3.1.1.5) (Patatin-like phospholipase domain-containing protein 6)	150 kDa	0.28	24	8	16	6	0	1	1	4	1	5	6	2.088182052	95% (0.042)
P4K2A_HUMAN	NUP98_HUMAN	NUP98	P52948	Nuclear pore complex protein Nup98-Nup96 [Cleaved into: Nuclear pore complex protein Nup98 (98 kDa nucleoporin) (Nucleoporin Nup98) (Nup98); Nuclear pore complex protein Nup96 (96 kDa nucleoporin) (Nucleoporin Nup96) (Nup96)]	198 kDa	0.35	44	15	29	4	2	2	7	4	3	15	7	2.084659746	95% (0.0091)
B2R6D7_HUMAN (+4)	XPP1_HUMAN	XPNPEP1	Q9NQW7	Xaa-Pro aminopeptidase 1 (EC 3.4.11.9) (Aminoacylproline aminopeptidase) (Cytosolic aminopeptidase P) (Soluble aminopeptidase P) (sAmp) (X-Pro aminopeptidase 1) (X-prolyl aminopeptidase 1, soluble)	70 kDa	0.26	21	7	14	1	1	1	4	3	6	2	3	2.069807567	0% (0.057)
RAB5C_HUMAN	B3KQF0_HUMAN		B3KQF0	cDNA FLJ90354 fis, clone NT2RP2003390, highly similar to Translocation protein SEC63 homolog	88 kDa	0.39	69	24	45	11	3	5	6	5	5	7	28	2.051461156	95% (0.0018)
STT3B_HUMAN	SAHH_HUMAN	AHCY	P23526	Adenosylhomocysteinase (AdoHcyase) (EC 3.3.1.1) (S-adenosyl-L-homocysteine hydrolase)	48 kDa	0.14	32	11	21	0	3	2	8	6	8	4	2	2.03389918	95% (0.027)
FUMH_HUMAN	A8K6A7_HUMAN		A8K6A7	cDNA FLJ76867, highly similar to Homo sapiens mannosidase, alpha, class 2B, member 1 (MAN2B1), mRNA	114 kDa	0.22	32	11	21	0	6	6	0	0	0	10	11	2.03389918	95% (0.027)
IDH3B_HUMAN	A8K3C3_HUMAN		A8K3C3	T-complex protein 1 subunit delta	58 kDa	0.24	76	27	50	6	8	6	7	23	12	11	4	2.031902541	95% (0.0012)
F8W1A4_HUMAN (+1)	RMD3_HUMAN	RMDN3	Q96TC7	Regulator of microtubule dynamics protein 3 (RMD-3) (hRMD-3) (Cerebral protein 10) (Protein FAM82A2) (Protein FAM82C) (Protein tyrosine phosphatase-interacting protein 51)	52 kDa	0.37	29	10	19	5	1	0	4	0	0	12	7	2.015584836	95% (0.035)

				(TCPTP-interacting protein 51)														
ABD12_HUMAN	MTX1_HUMAN	MTX1	Q13505	Metaxin-1 (Mitochondrial outer membrane import complex protein 1)	51 kDa	0.19	29	10	19	3	0	0	7	6	4	5	4	2.015584836	95% (0.035)
LRP1_HUMAN	B4DZ67_HUMAN	NUP107	B4DZ67	Nuclear pore complex protein Nup107 (cDNA FLJ58739, highly similar to Nuclear pore complex protein Nup107)	103 kDa	0.075	29	10	19	3	3	1	3	3	2	10	4	2.015584836	95% (0.035)
H7BXI1_HUMAN	B4DT03_HUMAN		B4DT03	cDNA FLJ61382, highly similar to Protein GPR89A	51 kDa	0.16	15	5	10	3	0	1	1	2	4	2	2	2.015453339	0% (0.10)
SYEP_HUMAN	NMT1_HUMAN	NMT1	P30419	Glycylpeptide N-tetradecanoyltransferase 1 (EC 2.3.1.97) (Myristoyl-CoA:protein N-myristoyltransferase 1) (NMT 1) (Type I N-myristoyltransferase) (Peptide N-myristoyltransferase 1)	57 kDa	0.15	15	5	10	0	1	0	4	4	4	2	0	2.015453339	0% (0.10)
VINC_HUMAN	B3KUY2_HUMAN	PTGES3	B3KUY2	Prostaglandin E synthase 3 (Cytosolic), isoform CRA_c (cDNA FLJ40895 fis, clone UTERU2002294, highly similar to Prostaglandin E synthase 3 (EC 5.3.99.3))	19 kDa	0.18	15	5	10	0	1	1	3	3	5	2	0	2.015453339	0% (0.10)
MGST1_HUMAN (+1)	B4DZ28_HUMAN		B4DZ28	cDNA FLJ53577, highly similar to pre-mRNA-splicing factor ATP-dependentRNA helicase DHX16 (EC 3.6.1.-)	112 kDa	0.27	15	5	10	0	0	2	0	2	0	6	0	2.015453339	0% (0.10)
SFXN1_HUMAN	SYNE3_HUMAN	SYNE3	Q6ZMZ3	Nesprin-3 (Nuclear envelope spectrin repeat protein 3)	112 kDa	0.37	2	0	1	0	0	0	1	0	0	1	0	2.015321876	0% (0.47)
HUWE1_HUMAN	A8KA74_HUMAN		A8KA74	cDNA FLJ76065	98 kDa	0.066	1	0	1	0	0	0	0	0	0	2	0	2.015321876	0% (0.47)
EIF3A_HUMAN (+2)	F208A_HUMAN	FAM208A	Q9UK61	Protein FAM208A (CTCL tumor antigen se89-1) (Retinoblastoma-associated protein RAP140)	189 kDa	0.092	1	0	1	0	0	0	0	2	0	0	0	2.015321876	0% (0.47)
A8K9T3_HUMAN (+2)	SR140_HUMAN	U2SURP	O15042	U2 snRNP-associated SURP motif-containing protein (140 kDa Ser/Arg-rich domain protein) (U2-associated protein SR140)	118 kDa	0.29	51	18	33	2	5	4	7	8	4	12	9	1.992513468	95% (0.0085)
ACOT9_HUMAN	F1T0J2_HUMAN	GOLGB1	F1T0J2	Golgin subfamily B member 1	367 kDa	0.29	48	17	31	5	5	6	1	13	1	12	5	1.978934203	95% (0.011)
B9EJA8_HUMAN (+1)	PCNA_HUMAN	PCNA	P12004	Proliferating cell nuclear antigen (PCNA) (Cyclin)	29 kDa	0.2	12	4	8	0	1	1	2	2	3	2	1	1.972766827	0% (0.14)
ARP3_HUMAN	INO1_HUMAN	ISYNA1	Q9NPH2	Inositol-3-phosphate synthase 1 (IPS 1) (EC 5.5.1.4) (Myo-inositol 1-phosphate synthase) (MI-1-P synthase) (MIP synthase) (hIPS) (Myo-inositol 1-phosphate synthase A1) (hINO1)	61 kDa	0.22	12	4	8	0	0	0	4	2	4	2	0	1.972766827	0% (0.14)
SYK_HUMAN	5NTC_HUMAN	NT5C2	P49902	Cytosolic purine 5'-nucleotidase (EC 3.1.3.5) (Cytosolic 5'-nucleotidase II)	65 kDa	0.26	12	4	8	2	0	0	2	0	7	0	1	1.972766827	0% (0.14)
AL3A2_HUMAN	CO4B_HUMAN	C4B	P0C0L5	Complement C4-B (Basic complement C4) (C3 and PZP-like alpha-2-macroglobulin domain-containing protein 3) [Cleaved into: Complement C4 beta chain; Complement C4-B alpha chain; C4a anaphylatoxin; C4b-B; C4d-B; Complement C4 gamma chain]	193 kDa	0.23	34	12	22	0	2	2	8	3	0	17	3	1.96491262	95% (0.029)
UFL1_HUMAN	TFR1_HUMAN	TFRC	P02786	Transferrin receptor protein 1 (TR) (TfR) (TfR1) (Trfr) (T9) (p90) (CD antigen CD71) [Cleaved into: Transferrin receptor protein 1, serum form (sTfR)]	85 kDa	0.17	322	117	205	29	34	37	20	59	36	47	65	1.955634563	95% (< 0.00010)
E7EX73_HUMAN	A8K6I4_HUMAN		A8K6I4	cDNA FLJ76877, highly similar to Homo sapiens superkiller viralicidic activity 2-like 2 (SKIV2L2), mRNA	118 kDa	0.14	42	15	27	4	4	4	3	4	1	12	10	1.946774972	95% (0.019)
B4DKN9_HUMAN (+1)	PCKGC_HUMAN	PCK1	P35558	Phosphoenolpyruvate carboxykinase, cytosolic [GTP] (PEPCK-C) (EC 4.1.1.32)	69 kDa	0.34	31	11	20	0	0	0	0	2	12	4	3	1.942460031	95% (0.038)

				(Phosphoenolpyruvate carboxylase)														
HNRPF_HUMAN	A8K9W7_HUMAN		A8K9W7	cDNA FLJ77440, highly similar to Homo sapiens general transcription factor II, i (GTF2I), transcript variant 2, mRNA	110 kDa	0.27	60	22	39	3	7	4	7	20	3	8	7	1.938784453	95% (0.0059)
B4DRA0_HUMAN (+1)	A8K6Q8_HUMAN		A8K6Q8	cDNA FLJ75881, highly similar to Homo sapiens transferrin receptor (p90, CD71) (TFRC), mRNA	85 kDa	0.26	325	119	206	32	0	0	0	59	37	48	64	1.932410728	95% (< 0.00010)
ATPO_HUMAN	FAS_HUMAN	FASN	P49327	Fatty acid synthase (EC 2.3.1.85) [Includes: [Acyl-carrier-protein] S-acetyltransferase (EC 2.3.1.38); [Acyl-carrier-protein] S-malonyltransferase (EC 2.3.1.39); 3-oxoacyl-[acyl-carrier-protein] synthase (EC 2.3.1.41); 3-oxoacyl-[acyl-carrier-protein] reductase (EC 1.1.1.100); 3-hydroxyacyl-[acyl-carrier-protein] dehydratase (EC 4.2.1.59); Enoyl-[acyl-carrier-protein] reductase (EC 1.3.1.39); Oleoyl-[acyl-carrier-protein] hydrolase (EC 3.1.2.14)]	273 kDa	0.46	205	75	130	6	28	25	16	43	52	26	7	1.92894273	95% (< 0.00010)
ASPH_HUMAN	XPO2_HUMAN	CSE1L	P55060	Exportin-2 (Exp2) (Cellular apoptosis susceptibility protein) (Chromosome segregation 1-like protein) (Importin-alpha re-exporter)	110 kDa	0.18	90	32	56	3	8	10	11	26	17	12	5	1.928505577	95% (0.0013)
Q53GW1_HUMAN (+1)	B3KNN9_HUMAN		B3KNN9	cDNA FLJ30070 fis, clone ASTRO2000046, highly similar to CD166 ANTIGEN	62 kDa	0.21	29	10	18	6	1	3	0	6	0	7	6	1.916022214	0% (0.051)
Q2TU34_HUMAN	K7ES61_HUMAN	MRPL4	K7ES61	39S ribosomal protein L4, mitochondrial (Fragment)	34 kDa	0.33	28	10	18	5	4	0	1	8	2	4	4	1.916022214	0% (0.051)
A8K781_HUMAN (+2)	CKAP5_HUMAN	CKAP5	Q14008	Cytoskeleton-associated protein 5 (Colonic and hepatic tumor overexpressed gene protein) (Ch-TOG)	226 kDa	0.29	9	3	6	0	1	2	0	0	4	1	1	1.910009327	0% (0.20)
SYRC_HUMAN	CBPM_HUMAN	CPM	P14384	Carboxypeptidase M (CPM) (EC 3.4.17.12)	51 kDa	0.32	9	3	6	0	1	1	1	3	2	0	1	1.910009327	0% (0.20)
CBX3_HUMAN	MRP4_HUMAN	ABCC4	O15439	Multidrug resistance-associated protein 4 (ATP-binding cassette sub-family C member 4) (MRP/cMOAT-related ABC transporter) (Multi-specific organic anion transporter B) (MOAT-B)	150 kDa	0.3	9	3	6	0	1	2	0	1	1	4	0	1.910009327	0% (0.20)
D9HTE9_HUMAN (+2)	ANM5_HUMAN	PRMT5	O14744	Protein arginine N-methyltransferase 5 (EC 2.1.1.-) (72 kDa ICln-binding protein) (Histone-arginine N-methyltransferase PRMT5) (EC 2.1.1.125) (Jak-binding protein 1) (Shk1 kinase-binding protein 1 homolog) (SKB1 homolog) (SKB1Hs) [Cleaved into: Protein arginine N-methyltransferase 5, N-terminally processed]	73 kDa	0.28	9	3	6	1	0	0	2	2	3	1	0	1.910009327	0% (0.20)
A8K132_HUMAN (+1)	NAGAB_HUMAN	NAGA	P17050	Alpha-N-acetylgalactosaminidase (EC 3.2.1.49) (Alpha-galactosidase B)	47 kDa	0.25	9	3	6	0	1	2	0	1	0	5	0	1.910009327	0% (0.20)
DJB11_HUMAN	EI2BE_HUMAN	EIF2B5	Q13144	Translation initiation factor eIF-2B subunit epsilon (eIF-2B GDP-GTP exchange factor subunit epsilon)	80 kDa	0.2	9	3	6	0	1	1	1	4	2	0	0	1.910009327	0% (0.20)
B4DJQ5_HUMAN (+2)	B7ZLZ2_HUMAN	EDEM3	B7ZLZ2	ER degradation-enhancing alpha-mannosidase-like protein 3 (Uncharacterized protein)	102 kDa	0.2	9	3	6	0	3	0	0	1	1	3	1	1.910009327	0% (0.20)
GBB2_HUMAN	EF1B_HUMAN	EEF1B2	P24534	Elongation factor 1-beta (EF-1-beta)	25 kDa	0.28	9	3	6	0	1	1	1	1	2	2	1	1.910009327	0% (0.20)

SNAA_HUMAN	E9PHV4_HUMA N	POLR2D	E9PHV4	DNA-directed RNA polymerase II subunit RPB4	13 kDa	0.39	9	3	6	0	2	1	0	0	0	4	2	1.9100093 27	0% (0.20)
PSD13_HUMAN (+1)	IDI1_HUMAN	IDI1	Q13907	Isopentenyl-diphosphate Delta-isomerase 1 (EC 5.3.3.2) (Isopentenyl pyrophosphate isomerase 1) (IPP isomerase 1) (IPPI1)	26 kDa	0.31	17	6	11	1	3	1	1	4	3	1	3	1.8919035 53	0% (0.11)
MLEC_HUMAN	E9PDC5_HUMA N	PTPRS	E9PDC5	Receptor-type tyrosine-protein phosphatase S	217 kDa	0.33	17	6	11	0	0	0	0	6	0	0	2	1.8919035 53	0% (0.11)
NPM_HUMAN	D3DP46_HUMA N	SPCS3	D3DP46	Signal peptidase complex subunit 3 homolog (S. cerevisiae), isoform CRA_a	20 kDa	0.2	25	9	16	3	1	2	3	2	6	5	3	1.8844327 6	0% (0.067)
Q567R6_HUMA N (+1)	Q4LE48_HUMA N	STAG1	Q4LE48	STAG1 variant protein (Fragment)	146 kDa	0.61	25	9	16	0	3	0	0	6	0	7	3	1.8844327 6	0% (0.067)
OXLA_HUMAN	A8MXP9_HUM AN	MATR3	A8MXP9	Matrin-3	100 kDa	0.21	107	40	68	4	11	10	15	14	8	33	13	1.8804563 48	95% (0.0006 4)
ITAM_HUMAN	B4DDT3_HUMA N		B4DDT3	cDNA FLJ54622, highly similar to Prothrombin (EC 3.4.21.5)	53 kDa	0.2	41	15	26	0	6	4	6	4	11	3	8	1.8778355 62	95% (0.026)
Q5VU59_HUMA N	H0Y4R1_HUMA N	IMPDH2	H0Y4R1	Inosine-5'-monophosphate dehydrogenase 2 (Fragment)	51 kDa	0.46	49	18	31	0	1	0	17	7	21	1	1	1.8761083 17	95% (0.017)
HEAT1_HUMA N	A8K492_HUMA N		A8K492	cDNA FLJ76789, highly similar to Homo sapiens methionine-tRNA synthetase (MARS), mRNA	101 kDa	0.13	65	24	41	5	8	4	7	14	17	7	3	1.8739295 97	95% (0.0068)
COPG1_HUMA N	B2RBR9_HUMA N		B2RBR9	cDNA, FLJ95650, highly similar to Homo sapiens karyopherin (importin) beta 1 (KPNB1), mRNA	97 kDa	0.27	177	66	111	8	16	23	19	26	24	41	21	1.8701881 15	95% (< 0.00010)
E9PLK3_HUMA N (+1)	MBB1A_HUMA N	MYBBP1A	Q9BQG0	Myb-binding protein 1A	149 kDa	0.23	94	35	59	2	8	8	17	17	14	22	6	1.8616074 51	95% (0.0016)
B3AT_HUMAN (+3)	HS90A_HUMAN	HSP90AA1	P07900	Heat shock protein HSP 90-alpha (Heat shock 86 kDa) (HSP 86) (HSP86) (Renal carcinoma antigen NY-REN-38)	85 kDa	0.16	437	164	273	19	47	40	59	64	94	73	43	1.8615024 48	95% (< 0.00010)
CH10_HUMAN	PSIP1_HUMAN	PSIP1	O75475	PC4 and SFRS1-interacting protein (CLL-associated antigen KW-7) (Dense fine speckles 70 kDa protein) (DFS 70) (Lens epithelium-derived growth factor) (Transcriptional coactivator p75/p52)	60 kDa	0.7	46	17	29	1	6	6	4	11	2	10	6	1.8561561 78	95% (0.021)
J3KRX5_HUMA N (+2)	ERG7_HUMAN	LSS	P48449	Lanosterol synthase (EC 5.4.99.7) (2,3-epoxysqualene--lanosterol cyclase) (Oxidosqualene--lanosterol cyclase) (OSC) (hOSC)	83 kDa	0.29	39	14	24	2	4	7	1	19	0	2	4	1.8540824 32	95% (0.034)
RS13_HUMAN	CADH1_HUMA N	CDH1	P12830	Cadherin-1 (CAM 120/80) (Epithelial cadherin) (E-cadherin) (Uvomorulin) (CD antigen CD324) [Cleaved into: E-Cad/CTF1; E-Cad/CTF2; E-Cad/CTF3]	97 kDa	0.46	30	11	19	5	2	2	2	7	2	3	7	1.8510235 13	0% (0.054)
A0S3T1_HUMA N (+113)	DHC24_HUMA N	DHCR24	Q15392	Delta(24)-sterol reductase (EC 1.3.1.72) (24-dehydrocholesterol reductase) (3-beta-hydroxysterol delta-24-reductase) (Diminuto/dwarf1 homolog) (Seladin-1)	60 kDa	0.19	79	30	50	4	9	3	14	15	11	16	8	1.8367690 74	95% (0.0039)
B4DLT2_HUMA N (+1)	DNJA1_HUMA N	DNAJA1	P31689	DnaJ homolog subfamily A member 1 (DnaJ protein homolog 2) (HSDJ) (Heat shock 40 kDa protein 4) (Heat shock protein J2) (HSJ-2) (Human DnaJ protein 2) (hDj-2)	45 kDa	0.13	15	5	9	0	1	2	2	4	2	0	3	1.8362755 13	0% (0.15)
EMC1_HUMAN	B4DEL5_HUMA N		B4DEL5	cDNA FLJ57874, highly similar to Homo sapiens succinate dehydrogenase complex, subunit C, transcript variant 3, mRNA	15 kDa	0.16	14	5	9	1	0	0	5	4	2	2	1	1.8362755 13	0% (0.15)
J3KTL2_HUMA N (+1)	B4E259_HUMA N		B4E259	cDNA FLJ59607, moderately similar to D-dopachrome decarboxylase (EC 4.1.1.84) (cDNA, FLJ78842, moderately	25 kDa	0.39	14	5	9	0	2	3	0	4	3	2	0	1.8362755 13	0% (0.15)

				similar to D-dopachrome decarboxylase (EC 4.1.1.84))														
PDC6I_HUMAN (+1)	WDR43_HUMAN	WDR43	Q15061	WD repeat-containing protein 43	75 kDa	0.23	14	5	9	1	1	2	2	5	1	2	1	1.836275513	0% (0.15)
S10A8_HUMAN	B2R9L0_HUMAN		B2R9L0	cDNA, FLJ94443, highly similar to Homo sapiens NADH dehydrogenase (ubiquinone) 1 beta subcomplex, 5, 16kDa (NDUFB5), mRNA	22 kDa	0.69	14	5	9	3	0	1	1	3	2	4	0	1.836275513	0% (0.15)
RL15_HUMAN	ARPC5_HUMAN	ARPC5	O15511	Actin-related protein 2/3 complex subunit 5 (Arp2/3 complex 16 kDa subunit) (p16-ARC)	16 kDa	0.38	14	5	9	1	1	2	1	2	3	2	2	1.836275513	0% (0.15)
B3KNN7_HUMAN (+3)	Q6FHJ5_HUMAN	SCAMP3	Q6FHJ5	SCAMP3 protein (Fragment)	38 kDa	0.27	14	5	9	2	0	3	0	1	2	2	4	1.836275513	0% (0.15)
RAB18_HUMAN	B2R6P1_HUMAN		B2R6P1	cDNA, FLJ93045, highly similar to Homo sapiens galactosamine (N-acetyl)-6-sulfate sulfatase (Morquiosyndrome, mucopolysaccharidosis type IVA) (GALNS), mRNA	58 kDa	0.49	14	5	9	5	0	0	0	2	0	4	3	1.836275513	0% (0.15)
RAB1A_HUMAN	OSTC_HUMAN	OSTC	Q9NRP0	Oligosaccharyltransferase complex subunit OSTC (Hydrophobic protein HSF-28)	17 kDa	0.5	14	5	9	1	1	1	2	1	4	2	2	1.836275513	0% (0.15)
B3KMV8_HUMAN (+1)	Q86X36_HUMAN	DHX8	Q86X36	DEAH (Asp-Glu-Ala-His) box polypeptide 8	139 kDa	0.26	14	5	9	0	2	0	0	2	0	4	3	1.836275513	0% (0.15)
J9JID7_HUMAN (+1)	KCRB_HUMAN	CKB	P12277	Creatine kinase B-type (EC 2.7.3.2) (B-CK) (Creatine kinase B chain)	43 kDa	0.31	43	16	27	2	2	1	11	14	6	6	2	1.833894886	95% (0.028)
B2RB06_HUMAN (+3)	PRS7_HUMAN	PSMC2	P35998	26S protease regulatory subunit 7 (26S proteasome AAA-ATPase subunit RPT1) (Proteasome 26S subunit ATPase 2) (Protein MSS1)	49 kDa	0.16	57	21	35	1	6	5	9	7	19	8	1	1.824505497	95% (0.015)
BGAL_HUMAN (+2)	F5H897_HUMAN	TRAP1	F5H897	Heat shock protein 75 kDa, mitochondrial	74 kDa	0.19	209	79	129	17	27	20	15	64	16	25	25	1.818712119	95% (< 0.00010)
D3DPU2_HUMAN	NOP2_HUMAN	NOP2	P46087	Putative ribosomal RNA methyltransferase NOP2 (EC 2.1.1.-) (Nucleolar protein 1) (Nucleolar protein 2 homolog) (Proliferating-cell nucleolar antigen p120) (Proliferation-associated nucleolar protein p120)	89 kDa	0.21	28	10	17	1	3	3	3	4	6	5	3	1.816462457	0% (0.072)
KAD3_HUMAN (+1)	LARP7_HUMAN	LARP7	Q4G0J3	La-related protein 7 (La ribonucleoprotein domain family member 7) (P-TEFb-interaction protein for 7SK stability) (PIP7S)	67 kDa	0.55	6	2	4	0	0	2	0	2	0	1	1	1.808653774	0% (0.29)
H3BPE1_HUMAN (+1)	WDR3_HUMAN	WDR3	Q9UNX4	WD repeat-containing protein 3	106 kDa	0.035	4	2	4	0	0	1	1	3	0	0	0	1.808653774	0% (0.29)
B4DLN1_HUMAN	A8K8E1_HUMAN	TBC1D15	A8K8E1	TBC1 domain family member 15 (TBC1 domain family, member 15, isoform CRA_d) (cDNA FLJ77374)	52 kDa	0.33	6	2	4	0	1	1	0	2	2	0	0	1.808653774	0% (0.29)
B4DRS6_HUMAN	B4DS83_HUMAN		B4DS83	cDNA FLJ53179, highly similar to Nucleolar protein 10	74 kDa	0.38	6	2	4	0	1	1	0	2	1	1	0	1.808653774	0% (0.29)
A8K651_HUMAN (+1)	FA5_HUMAN	F5	P12259	Coagulation factor V (Activated protein C cofactor) (Proaccelerin, labile factor) [Cleaved into: Coagulation factor V heavy chain; Coagulation factor V light chain]	252 kDa	0.15	6	2	4	0	1	0	1	0	4	0	0	1.808653774	0% (0.29)
Q9BTQ7_HUMAN (+1)	PEX16_HUMAN	PEX16	Q9Y5Y5	Peroxisomal membrane protein PEX16 (Peroxin-16) (Peroxisomal biogenesis factor 16)	39 kDa	0.4	6	2	4	0	0	0	2	0	2	1	1	1.808653774	0% (0.29)
B4DSE2_HUMAN (+4)	RPRD2_HUMAN	RPRD2	Q5VT52	Regulation of nuclear pre-mRNA domain-containing protein 2	156 kDa	0.16	6	2	4	0	0	1	1	0	0	4	0	1.808653774	0% (0.29)
ARM10_HUMAN	ACSA_HUMAN	ACSS2	Q9NR19	Acetyl-coenzyme A synthetase, cytoplasmic (EC 6.2.1.1) (Acetate--CoA ligase) (Acetyl-CoA synthetase) (ACS) (AceCS) (Acyl-CoA	79 kDa	0.33	6	2	4	0	1	1	0	2	1	1	0	1.808653774	0% (0.29)

				synthetase short-chain family member 2) (Acyl-activating enzyme)															
Q59H06_HUMAN (+1)	A8K6U0_HUMAN		A8K6U0	cDNA FLJ77572, highly similar to Homo sapiens intracellular membrane-associated calcium-independent phospholipase A2 gamma (IPLA2(GAMMA)), mRNA	89 kDa	0.12	6	2	4	0	0	1	1	0	0	1	3	1.808653774	0% (0.29)
B2R4C0_HUMAN (+2)	B4DP38_HUMAN	WDR77	B4DP38	Methylosome protein 50 (cDNA FLJ55120, moderately similar to Methylosome protein 50)	30 kDa	0.38	6	2	4	0	1	0	1	0	2	0	2	1.808653774	0% (0.29)
CBR1_HUMAN	B2RAH7_HUMAN		B2RAH7	cDNA, FLJ94921, highly similar to Homo sapiens prolyl endopeptidase (PREP), mRNA	81 kDa	0.54	6	2	4	0	0	2	0	0	3	1	0	1.808653774	0% (0.29)
ODPA_HUMAN	VANG1_HUMAN	VANGL1	Q8TAA9	Vang-like protein 1 (Loop-tail protein 2 homolog) (LPP2) (Strabismus 2) (Van Gogh-like protein 1)	60 kDa	0.25	6	2	4	0	0	0	2	0	2	2	0	1.808653774	0% (0.29)
SAM50_HUMAN	AIFM2_HUMAN	AIFM2	Q9BRQ8	Apoptosis-inducing factor 2 (EC 1.-.-.-) (Apoptosis-inducing factor homologous mitochondrion-associated inducer of death) (Apoptosis-inducing factor-like mitochondrion-associated inducer of death) (p53-responsive gene 3 protein)	41 kDa	0.2	6	2	4	1	1	0	0	0	2	1	1	1.808653774	0% (0.29)
RENT1_HUMAN	B4DXB6_HUMAN		B4DXB6	cDNA FLJ58335, highly similar to GPI transamidase component PIG-U	48 kDa	0.2	6	2	4	0	1	0	1	1	0	0	3	1.808653774	0% (0.29)
M1VPF6_HUMAN	VSIG4_HUMAN	VSIG4	Q9Y279	V-set and immunoglobulin domain-containing protein 4 (Protein Z39Ig)	44 kDa	0.084	5	2	4	0	1	1	0	0	0	1	3	1.808653774	0% (0.29)
TOIP1_HUMAN	B2R9X3_HUMAN		B2R9X3	cDNA, FLJ94599, highly similar to Homo sapiens GDP-mannose 4,6-dehydratase (GMDS), mRNA	42 kDa	0.14	6	2	4	0	0	0	2	0	4	0	0	1.808653774	0% (0.29)
TMM43_HUMAN	SRPK2_HUMAN	SRPK2	P78362	SRSF protein kinase 2 (EC 2.7.11.1) (SFRS protein kinase 2) (Serine/arginine-rich protein-specific kinase 2) (SR-protein-specific kinase 2) [Cleaved into: SRSF protein kinase 2 N-terminal; SRSF protein kinase 2 C-terminal]	78 kDa	0.34	6	2	4	0	1	0	1	0	3	1	0	1.808653774	0% (0.29)
B3KQF0_HUMAN (+1)	B4E2I4_HUMAN		B4E2I4	cDNA FLJ58227, highly similar to Glutamate--cysteine ligase catalytic subunit (EC 6.3.2.2)	66 kDa	0.24	6	2	4	0	0	2	0	1	0	3	0	1.808653774	0% (0.29)
ERLN2_HUMAN	ADCK3_HUMAN	ADCK3	Q8NI60	Chaperone activity of bc1 complex-like, mitochondrial (Chaperone-ABC1-like) (EC 2.7.11.-) (aarF domain-containing protein kinase 3)	72 kDa	0.4	6	2	4	1	1	0	0	4	0	0	0	1.808653774	0% (0.29)
A8K5N5_HUMAN (+2)	D6RDJ3_HUMAN	POLR1C	D6RDJ3	DNA-directed RNA polymerases I and III subunit RPAC1 (Fragment)	14 kDa	0.22	5	2	4	0	1	0	1	1	2	1	0	1.808653774	0% (0.29)
CLIC1_HUMAN (+1)	FA98B_HUMAN	FAM98B	Q52LJ0	Protein FAM98B	37 kDa	0.4	6	2	4	0	0	0	2	1	0	0	0	1.808653774	0% (0.29)
DX39A_HUMAN	FKBP4_HUMAN	FKBP4	Q02790	Peptidyl-prolyl cis-trans isomerase FKBP4 (PPIase FKBP4) (EC 5.2.1.8) (51 kDa FK506-binding protein) (FKBP51) (52 kDa FK506-binding protein) (52 kDa FKBP) (FKBP-52) (59 kDa immunophilin) (p59) (FK506-binding protein 4) (FKBP-4) (FKBP59) (HSP-binding immunophilin) (HBI) (Immunophilin FKBP52) (Rotamase) [Cleaved into: Peptidyl-prolyl cis-trans isomerase FKBP4, N-terminally processed]	52 kDa	0.29	112	43	70	1	16	11	15	12	41	13	4	1.803577396	95% (0.0010)

AT131_HUMAN	B3KY60_HUMAN		B3KY60	cDNA FLJ16777 fis, clone BRHIP2029567, highly similar to Cell division cycle 5-like protein	92 kDa	0.15	19	7	12	1	2	2	2	5	0	4	3	1.798305649	0% (0.12)
Q53G69_HUMAN (+2)	WDR36_HUMAN	WDR36	Q8NI36	WD repeat-containing protein 36 (T-cell activation WD repeat-containing protein) (TA-WDRP)	105 kDa	0.31	19	7	12	2	2	1	2	8	0	4	0	1.798305649	0% (0.12)
RAP1A_HUMAN	XPO1_HUMAN	XPO1	O14980	Exportin-1 (Exp1) (Chromosome region maintenance 1 protein homolog)	123 kDa	0.42	119	45	73	4	9	7	26	27	28	17	1	1.798272512	95% (0.00087)
ARPC2_HUMAN	Q53GD7_HUMAN		Q53GD7	FUS interacting protein (Serine-arginine rich) 1 isoform 2 variant (Fragment)	31 kDa	0.32	36	14	23	4	2	2	6	5	5	4	9	1.780627802	95% (0.047)
A8K3C3_HUMAN (+1)	CBR1_HUMAN	CBR1	P16152	Carbonyl reductase [NADPH] 1 (EC 1.1.1.184) (15-hydroxyprostaglandin dehydrogenase [NADP(+)]) (EC 1.1.1.197) (NADPH-dependent carbonyl reductase 1) (Prostaglandin 9-ketoreductase) (Prostaglandin-E(2) 9-reductase) (EC 1.1.1.189)	30 kDa	0.27	81	31	50	3	7	8	13	12	24	11	3	1.779792119	95% (0.0056)
FLOT2_HUMAN	CUL4A_HUMAN	CUL4A	Q13619	Cullin-4A (CUL-4A)	88 kDa	0.35	24	9	15	1	3	4	1	7	1	5	2	1.77516474	0% (0.095)
B2R7M1_HUMAN (+4)	RM37_HUMAN	MRPL37	Q9BZE1	39S ribosomal protein L37, mitochondrial (L37mt) (MRP-L37) (39S ribosomal protein L2, mitochondrial) (L2mt) (MRP-L2)	48 kDa	0.28	24	9	15	7	1	0	1	5	0	5	5	1.77516474	0% (0.095)
TOM70_HUMAN	VPP2_HUMAN	ATP6V0A2	Q9Y487	V-type proton ATPase 116 kDa subunit a isoform 2 (V-ATPase 116 kDa isoform a2) (Lysosomal H(+)-transporting ATPase V0 subunit a2) (TJ6) (Vacuolar proton translocating ATPase 116 kDa subunit a isoform 2)	98 kDa	0.11	24	9	15	4	3	1	1	4	4	2	5	1.77516474	0% (0.095)
Q86VG2_HUMAN	A1JUI8_HUMAN	CCT6A	A1JUI8	Chaperonin subunit 6A (Fragment)	54 kDa	0.16	42	16	26	1	4	6	5	5	13	5	2	1.768952797	95% (0.038)
D6R9P3_HUMAN	VWA8_HUMAN	VWA8	A3KMH1	von Willebrand factor A domain-containing protein 8	215 kDa	0.27	11	4	7	2	1	1	0	6	0	0	1	1.759469425	0% (0.21)
A8K7T4_HUMAN (+2)	ABCBA_HUMAN	ABCB10	Q9NRK6	ATP-binding cassette sub-family B member 10, mitochondrial (ATP-binding cassette transporter 10) (ABC transporter 10 protein) (Mitochondrial ATP-binding cassette 2) (M-ABC2)	79 kDa	0.27	12	4	7	1	1	1	0	2	3	0	3	1.759469425	0% (0.21)
VAT1_HUMAN	F8VY86_HUMAN	TP53BP1	F8VY86	Tumor suppressor p53-binding protein 1	214 kDa	0.22	11	4	7	1	0	0	2	2	0	2	2	1.759469425	0% (0.21)
Q59GM9_HUMAN	ES1_HUMAN	C21orf33	P30042	ES1 protein homolog, mitochondrial (Protein GT335) (Protein KNP-I)	28 kDa	0.25	11	4	7	0	2	2	1	5	0	0	2	1.759469425	0% (0.21)
LEG1_HUMAN	FUCO2_HUMAN	FUCA2	Q9BTY2	Plasma alpha-L-fucosidase (EC 3.2.1.51) (Alpha-L-fucoside fucohydrolase 2) (Alpha-L-fucosidase 2)	54 kDa	0.44	11	4	7	2	1	1	0	3	0	3	1	1.759469425	0% (0.21)
Q53EM5_HUMAN	THOC1_HUMAN	THOC1	Q96FV9	THO complex subunit 1 (Tho1) (Nuclear matrix protein p84) (p84N5) (hTREX84)	76 kDa	0.19	11	4	7	0	1	3	0	3	0	4	0	1.759469425	0% (0.21)
M0R210_HUMAN (+1)	SRP68_HUMAN	SRP68	Q9UHB9	Signal recognition particle subunit SRP68 (SRP68) (Signal recognition particle 68 kDa protein)	71 kDa	0.52	11	4	7	0	0	3	1	2	3	2	0	1.759469425	0% (0.21)
K6PL_HUMAN	B4DXJ1_HUMAN	SEC13	B4DXJ1	Protein SEC13 homolog (cDNA FLJ56334, highly similar to SEC13-related protein)	41 kDa	0.22	11	4	7	1	1	1	1	1	2	3	2	1.759469425	0% (0.21)
TCPA_HUMAN	DUT_HUMAN	DUT	P33316	Deoxyuridine 5'-triphosphate nucleotidohydrolase, mitochondrial (dUTPase) (EC 3.6.1.23) (dUTP pyrophosphatase)	27 kDa	0.25	10	4	7	0	2	0	2	4	0	3	0	1.759469425	0% (0.21)
D3DV26_HUMAN	ECI2_HUMAN	ECI2	O75521	Enoyl-CoA delta isomerase 2, mitochondrial (EC 5.3.3.8) (DRS-1) (Delta(3),delta(2)-	44 kDa	0.13	34	13	21	3	3	1	6	6	10	2	3	1.748394712	0% (0.061)

				enoyl-CoA isomerase) (D3,D2-enoyl-CoA isomerase) (Diazepam-binding inhibitor-related protein 1) (DBI-related protein 1) (Dodecenoyl-CoA isomerase) (Hepatocellular carcinoma-associated antigen 88) (Peroxisomal 3,2-trans-enoyl-CoA isomerase) (pECI) (Renal carcinoma antigen NY-REN-1)															
A8K4I8_HUMAN (+1)	B3KME2_HUMAN		B3KME2	cDNA FLJ10772 fis, clone NT2RP4000243, highly similar to Cartilage-associated protein	47 kDa	0.22	16	6	10	0	2	3	0	2	3	2	4	1.737437451	0% (0.16)
A8K897_HUMAN (+1)	RT07_HUMAN	MRPS7	Q9Y2R9	28S ribosomal protein S7, mitochondrial (MRP-S7) (S7mt) (bMRP-27a) (bMRP27a)	28 kDa	0.18	16	6	10	4	2	0	0	1	0	2	7	1.737437451	0% (0.16)
TAGL2_HUMAN	C9JL85_HUMAN	MTPN	C9JL85	Myotrophin	6 kDa	0.4	17	6	10	0	3	2	1	4	2	1	3	1.737437451	0% (0.16)
Q6ZS74_HUMAN	RBM14_HUMAN	RBM14	Q96PK6	RNA-binding protein 14 (Paraspeckle protein 2) (PSP2) (RNA-binding motif protein 14) (RRM-containing coactivator activator/modulator) (Synaptotagmin-interacting protein) (SYT-interacting protein)	69 kDa	0.24	16	6	10	2	0	1	3	4	2	2	2	1.737437451	0% (0.16)
A8KA83_HUMAN (+1)	G3P_HUMAN	GAPDH	P04406	Glyceraldehyde-3-phosphate dehydrogenase (GAPDH) (EC 1.2.1.12) (Peptidyl-cysteine S-nitrosylase GAPDH) (EC 2.6.99.-)	36 kDa	0.18	293	115	178	23	22	28	43	27	84	41	27	1.728236358	95% (< 0.00010)
SYDC_HUMAN	B4DU42_HUMAN		B4DU42	cDNA FLJ56153, highly similar to Homo sapiens transforming growth factor beta regulator 4 (TBRG4), transcript variant 1, mRNA	72 kDa	0.3	21	8	13	0	2	4	1	5	1	2	6	1.724945501	0% (0.13)
B3KM97_HUMAN (+1)	H0YKD8_HUMAN	RPL28	H0YKD8	60S ribosomal protein L28	19 kDa	0.25	24	8	13	1	1	1	4	5	6	2	2	1.724945501	0% (0.13)
M0R0F0_HUMAN (+3)	VPS45_HUMAN	VPS45	Q9NRW7	Vacuolar protein sorting-associated protein 45 (h-VPS45) (hlVps45)	65 kDa	0.32	26	10	16	3	4	3	0	5	3	5	3	1.716905564	0% (0.10)
AT1B3_HUMAN	CRNL1_HUMAN	CRNKL1	Q9BZJ0	Crooked neck-like protein 1 (Crooked neck homolog) (hCrn)	100 kDa	0.18	25	10	16	2	3	3	2	10	0	4	1	1.716905564	0% (0.10)
MGN2_HUMAN	B7Z6F7_HUMAN		B7Z6F7	cDNA FLJ61705, highly similar to Symplekin	141 kDa	0.48	26	10	16	1	5	2	2	5	5	5	1	1.716905564	0% (0.10)
THIK_HUMAN	B4DRT2_HUMAN	MRPS27	B4DRT2	28S ribosomal protein S27, mitochondrial (cDNA FLJ54536, highly similar to Mitochondrial 28S ribosomal protein S27)	49 kDa	0.23	26	10	16	3	3	3	1	7	1	2	6	1.716905564	0% (0.10)
Q59GA1_HUMAN (+2)	A6NJA2_HUMAN	USP14	A6NJA2	Ubiquitin carboxyl-terminal hydrolase (EC 3.4.19.12)	51 kDa	0.17	30	12	19	2	2	5	2	7	4	7	1	1.711301636	0% (0.080)
A8K4H1_HUMAN (+3)	A8K690_HUMAN		A8K690	cDNA FLJ76863, highly similar to Homo sapiens stress-induced-phosphoprotein 1 (Hsp70/Hsp90-organizing protein) (STIP1), mRNA	63 kDa	0.12	31	12	19	0	4	4	4	4	5	6	3	1.711301636	0% (0.080)
B0I1T1_HUMAN (+3)	CATZ_HUMAN	CTSZ	Q9UBR2	Cathepsin Z (EC 3.4.18.1) (Cathepsin P) (Cathepsin X)	34 kDa	0.13	31	12	19	1	7	2	2	3	2	7	7	1.711301636	0% (0.080)
Q6IAX9_HUMAN (+1)	B5BUB1_HUMAN	RUVBL1	B5BUB1	RuvB-like 1 (Fragment)	50 kDa	0.19	152	60	92	7	16	19	19	39	26	16	11	1.705487796	95% (0.00059)
ATD3A_HUMAN	B4DZQ5_HUMAN		B4DZQ5	cDNA FLJ51417, highly similar to Serine/threonine-protein kinase PRP4 homolog (EC 2.7.11.1)	115 kDa	0.18	46	18	28	5	3	6	4	6	1	14	7	1.701513154	95% (0.042)
B3KY63_HUMAN	B7Z747_HUMAN		B7Z747	cDNA FLJ51120, highly similar to Matrix metalloproteinase-9 (EC 3.4.24.35)	66 kDa	0.087	51	20	31	0	10	9	0	0	0	7	22	1.699489736	95% (0.034)
ADAS_HUMAN (+1)	SYRC_HUMAN	RARS	P54136	Arginine--tRNA ligase, cytoplasmic (EC 6.1.1.19) (Arginyl-tRNA synthetase) (ArgRS)	75 kDa	0.24	80	32	49	5	9	7	11	17	15	14	3	1.692535207	95% (0.010)

B4DW81_HUMAN (+1)	KPYM_HUMAN	PKM	P14618	Pyruvate kinase PKM (EC 2.7.1.40) (Cytosolic thyroid hormone-binding protein) (CTHBP) (Opa-interacting protein 3) (OIP-3) (Pyruvate kinase 2/3) (Pyruvate kinase muscle isozyme) (Thyroid hormone-binding protein 1) (THBP1) (Tumor M2-PK) (p58)	58 kDa	0.43	518	209	313	48	54	48	59	68	108	92	43	1.676474595	95% (< 0.00010)
Q59G75_HUMAN (+1)	B2R8A2_HUMAN		B2R8A2	cDNA, FLJ93804, highly similar to Homo sapiens gp25L2 protein (HSGP25L2G), mRNA	25 kDa	0.14	43	17	26	5	4	3	5	6	7	3	11	1.672002391	0% (0.054)
B4E0H8_HUMAN (+1)	B3KSI3_HUMAN	BCAT2	B3KSI3	Branched-chain-amino-acid aminotransferase (EC 2.6.1.42)	40 kDa	0.13	38	15	23	4	4	3	4	13	2	4	4	1.671029234	0% (0.067)
B3KVN0_HUMAN (+3)	B0UX83_HUMAN	BAG6	B0UX83	HLA-B associated transcript 3, isoform CRA_a (Large proline-rich protein BAG6)	119 kDa	0.14	28	11	17	1	4	3	3	5	3	7	2	1.668158372	0% (0.10)
THIL_HUMAN	B3KMR5_HUMAN		B3KMR5	cDNA FLJ12434 fis, clone NT2RM1000037, highly similar to Homo sapiens KIAA0690 protein	144 kDa	0.39	24	9	14	1	2	2	4	4	5	4	3	1.665899865	0% (0.13)
D3DUZ3_HUMAN (+2)	TM214_HUMAN	TMEM214	Q6NUQ4	Transmembrane protein 214	77 kDa	0.21	23	9	14	4	2	3	1	5	1	2	6	1.665899865	0% (0.13)
B7Z7A8_HUMAN (+4)	B1AKV3_HUMAN	UQCC1	B1AKV3	Protein UQCC1 (Fragment)	29 kDa	0.22	23	9	14	4	3	0	2	5	1	5	3	1.665899865	0% (0.13)
RHG01_HUMAN	K7EJE8_HUMAN	LONP1	K7EJE8	Lon protease homolog, mitochondrial (EC 3.4.21.-) (Lon protease-like protein) (Mitochondrial ATP-dependent protease Lon) (Serine protease 15)	93 kDa	0.2	192	77	115	25	20	14	19	50	12	16	38	1.664421981	95% (0.00024)
B5MDF5_HUMAN (+1)	GSTP1_HUMAN	GSTP1	P09211	Glutathione S-transferase P (EC 2.5.1.18) (GST class-pi) (GSTP1-1)	23 kDa	0.27	100	40	60	7	4	5	24	15	22	13	10	1.663027952	95% (0.0064)
A8K492_HUMAN (+1)	ZN326_HUMAN	ZNF326	Q5BKZ1	DBIRD complex subunit ZNF326 (Zinc finger protein 326) (Zinc finger protein interacting with mRNPs and DBC1)	66 kDa	0.17	18	7	11	2	2	1	2	2	2	4	3	1.662560548	0% (0.17)
G3V0I5_HUMAN (+1)	Q53G69_HUMAN		Q53G69	Translocase of inner mitochondrial membrane 44 homolog (Fragment)	51 kDa	0.18	79	32	48	10	7	4	11	25	9	5	8	1.658829036	95% (0.014)
A7E2D8_HUMAN	LGUL_HUMAN	GLO1	Q04760	Lactoylglutathione lyase (EC 4.4.1.5) (Aldoketomutase) (Glyoxalase I) (Glx I) (Ketone-aldehyde mutase) (Methylglyoxalase) (S-D-lactoylglutathione methylglyoxal lyase)	21 kDa	0.14	14	5	8	0	0	0	5	3	4	2	0	1.657102843	0% (0.22)
PTBP1_HUMAN	SE1L3_HUMAN	SEL1L3	Q68CR1	Protein sel-1 homolog 3 (Suppressor of lin-12-like protein 3) (Sel-1L3)	129 kDa	0.12	13	5	8	5	0	0	0	1	0	1	6	1.657102843	0% (0.22)
B3KWV6_HUMAN (+1)	B3KYA7_HUMAN	STK3	B3KYA7	Serine/threonine-protein kinase 3 (cDNA FLJ16404 fis, clone UTERU2008019, highly similar to Serine/threonine-protein kinase 3 (EC 2.7.11.1))	59 kDa	0.18	13	5	8	1	0	2	2	3	2	2	1	1.657102843	0% (0.22)
AL1B1_HUMAN	UK114_HUMAN	HRSP12	P52758	Ribonuclease UK114 (EC 3.1.-.-) (14.5 kDa translational inhibitor protein) (p14.5) (Heat-responsive protein 12) (UK114 antigen homolog)	14 kDa	0.24	13	5	8	0	2	2	1	3	2	2	1	1.657102843	0% (0.22)
CATA_HUMAN	SPHM_HUMAN	SGSH	P51688	N-sulphoglucosamine sulphohydrolase (EC 3.10.1.1) (Sulfoglucosamine sulfamidase) (Sulphamidase)	57 kDa	0.29	13	5	8	2	0	1	3	1	0	2	5	1.657102843	0% (0.22)
A8K3A8_HUMAN	NCOA5_HUMAN	NCOA5	Q9HCD5	Nuclear receptor coactivator 5 (NCoA-5) (Coactivator independent of AF-2) (CIA)	66 kDa	0.24	13	5	8	4	0	0	1	2	0	3	3	1.657102843	0% (0.22)
F1T0J2_HUMAN	RM41_HUMAN	MRPL41	Q8IXM3	39S ribosomal protein L41, mitochondrial (L41mt) (MRP-L41) (39S ribosomal protein L27 homolog) (Bcl-2-interacting mitochondrial ribosomal protein L41) (Cell proliferation-inducing gene 3 protein) (MRP-L27 homolog)	15 kDa	0.056	13	5	8	1	2	1	1	2	2	1	3	1.657102843	0% (0.22)

SAMH1_HUMAN	ARSA_HUMAN	ARSA	P15289	Arylsulfatase A (ASA) (EC 3.1.6.8) (Cerebroside-sulfatase) [Cleaved into: Arylsulfatase A component B; Arylsulfatase A component C]	54 kDa	0.31	13	5	8	4	1	1	0	1	1	4	2	1.657102843	0% (0.22)
B5BUB5_HUMAN (+1)	BGH3_HUMAN	TGFBI	Q15582	Transforming growth factor-beta-induced protein ig-h3 (Beta ig-h3) (Kerato-epithelin) (RGD-containing collagen-associated protein) (RGD-CAP)	75 kDa	0.23	13	5	8	0	1	1	3	0	3	1	4	1.657102843	0% (0.22)
FAM3C_HUMAN	B5BU32_HUMAN	TK1	B5BU32	Thymidine kinase (EC 2.7.1.21)	25 kDa	0.35	13	5	8	0	4	1	0	1	4	1	2	1.657102843	0% (0.22)
HNRH3_HUMAN (+1)	DSRAD_HUMAN	ADAR	P55265	Double-stranded RNA-specific adenosine deaminase (DRADA) (EC 3.5.4.37) (136 kDa double-stranded RNA-binding protein) (p136) (Interferon-inducible protein 4) (IFI-4) (K88DSRBP)	136 kDa	0.15	69	28	42	4	4	5	14	10	5	18	9	1.655892372	95% (0.020)
F8VXC8_HUMAN (+2)	B7Z507_HUMAN		B7Z507	cDNA FLJ51036, highly similar to Matrix metalloproteinase-9 (EC3.4.24.35)	72 kDa	0.14	55	22	33	3	11	9	0	0	2	8	23	1.649630651	95% (0.037)
B3KU62_HUMAN (+2)	MGST1_HUMAN	MGST1	P10620	Microsomal glutathione S-transferase 1 (Microsomal GST-1) (EC 2.5.1.18) (Microsomal GST-I)	18 kDa	0.22	97	39	58	14	9	10	6	15	19	10	14	1.648667042	95% (0.0080)
ARF4_HUMAN	Q86UA3_HUMAN	C12orf10	Q86UA3	Chromosome 12 open reading frame 10 (Chromosome 12 open reading frame 10, isoform CRA_b)	43 kDa	0.42	8	3	5	1	2	0	0	2	2	1	0	1.646536078	0% (0.30)
A8K9W7_HUMAN (+3)	J3KN32_HUMAN	RCOR1	J3KN32	REST corepressor 1 (REST corepressor 1, isoform CRA_a)	53 kDa	0.19	8	3	5	0	1	2	0	2	0	2	1	1.646536078	0% (0.30)
PSMD6_HUMAN	RMND1_HUMAN	RMND1	Q9NWS8	Required for meiotic nuclear division protein 1 homolog	52 kDa	0.51	8	3	5	1	1	0	1	3	0	0	2	1.646536078	0% (0.30)
SMC1A_HUMAN	GSTM2_HUMAN	GSTM2	P28161	Glutathione S-transferase Mu 2 (EC 2.5.1.18) (GST class-mu 2) (GSTM2-2)	26 kDa	0.14	8	3	5	0	0	0	3	1	5	0	0	1.646536078	0% (0.30)
ANM1_HUMAN (+2)	B2R6A3_HUMAN		B2R6A3	cDNA, FLJ92860, highly similar to Homo sapiens solute carrier family 9 (sodium/hydrogen exchanger), member 3 regulator 1 (SLC9A3R1), mRNA	39 kDa	0.33	8	3	5	0	1	1	1	1	0	0	4	1.646536078	0% (0.30)
HIBCH_HUMAN	CLPX_HUMAN	CLPX	O76031	ATP-dependent Clp protease ATP-binding subunit clpX-like, mitochondrial	69 kDa	0.21	8	3	5	1	0	1	1	3	0	2	0	1.646536078	0% (0.30)
PTN1_HUMAN	B2R514_HUMAN		B2R514	cDNA, FLJ92300, Homo sapiens COP9 subunit 6 (MOV34 homolog, 34 kD) (COPS6), mRNA	34 kDa	0.28	8	3	5	0	0	0	3	1	4	0	0	1.646536078	0% (0.30)
MPRI_HUMAN (+1)	A8K9D2_HUMAN	MRPL18	A8K9D2	Mitochondrial ribosomal protein L18, isoform CRA_b (cDNA FLJ76688, highly similar to Homo sapiens HSPC071 mRNA)	20 kDa	0.077	8	3	5	1	1	1	1	1	1	1	2	1.646536078	0% (0.30)
ODB2_HUMAN	B2R7N6_HUMAN		B2R7N6	cDNA, FLJ93528, highly similar to Homo sapiens solute carrier family 1 (glutamate/neutral amino acid transporter), member 4 (SLC1A4), mRNA	56 kDa	0.17	8	3	5	0	1	2	0	0	1	3	1	1.646536078	0% (0.30)
H0YHS6_HUMAN (+1)	B4DPS5_HUMAN	BPNT1	B4DPS5	3'(2'),5'-bisphosphate nucleotidase 1 (cDNA FLJ52203, highly similar to 3'(2'),5'-bisphosphate nucleotidase 1 (EC 3.1.3.7))	28 kDa	0.33	8	3	5	0	1	0	2	0	3	2	0	1.646536078	0% (0.30)
B0UXB6_HUMAN	DYL2_HUMAN	DYNLL2	Q96FJ2	Dynein light chain 2, cytoplasmic (8 kDa dynein light chain b) (DLC8b) (Dynein light chain LC8-type 2)	10 kDa	0.24	8	3	5	0	0	2	1	1	2	1	0	1.646536078	0% (0.30)
RS10_HUMAN	PDS5B_HUMAN	PDS5B	Q9NTI5	Sister chromatid cohesion protein PDS5 homolog B (Androgen-induced proliferation inhibitor) (Androgen-induced prostate	165 kDa	0.27	44	18	27	2	8	4	4	14	0	11	2	1.643318116	0% (0.056)

				proliferative shutoff-associated protein AS3)															
LOX5_HUMAN	EST1_HUMAN	CES1	P23141	Liver carboxylesterase 1 (Acyl-coenzyme A:cholesterol acyltransferase) (ACAT) (Brain carboxylesterase hBr1) (Carboxylesterase 1) (CE-1) (hCE-1) (EC 3.1.1.1) (Cocaine carboxylesterase) (Egasyn) (HMSE) (Methylumbelliferyl-acetate deacetylase 1) (EC 3.1.1.56) (Monocyte/macrophage serine esterase) (Retinyl ester hydrolase) (REH) (Serine esterase 1) (Triacylglycerol hydrolase) (TGH)	63 kDa	0.26	45	18	27	0	10	8	0	0	3	4	21	1.6433181 16	0% (0.056)
SOAT1_HUMA N	TCPB_HUMAN	CCT2	P78371	T-complex protein 1 subunit beta (TCP-1-beta) (CCT-beta)	57 kDa	0.12	116	46	68	1	12	19	14	23	26	14	4	1.6415416 24	95% (0.0047)
PRS7_HUMAN	HS90B_HUMAN	HSP90AB1	P08238	Heat shock protein HSP 90-beta (HSP 90) (Heat shock 84 kDa) (HSP 84) (HSP84)	83 kDa	0.35	512	208	305	30	61	56	62	72	106	78	49	1.6414351 15	95% (< 0.00010)
ACSS3_HUMAN	A4FTY4_HUMA N	TXNRD2	A4FTY4	TXNRD2 protein	36 kDa	0.17	35	14	21	0	5	4	0	10	4	0	4	1.6337248 85	0% (0.086)
B4DF70_HUMA N (+1)	GFPT1_HUMAN	GFPT1	Q06210	Glutamine--fructose-6-phosphate aminotransferase [isomerizing] 1 (EC 2.6.1.16) (D-fructose-6-phosphate amidotransferase 1) (Glutamine:fructose-6-phosphate amidotransferase 1) (GFAT 1) (GFAT1) (Hexosephosphate aminotransferase 1)	79 kDa	0.26	56	23	34	4	4	9	6	6	20	8	0	1.6277853 9	95% (0.038)
2AAA_HUMAN (+2)	Q53HU0_HUMA N		Q53HU0	Chaperonin containing TCP1, subunit 8 (Theta) variant (Fragment)	60 kDa	0.27	101	41	60	0	18	15	8	22	15	14	9	1.6236454 72	95% (0.0085)
RCC1_HUMAN	SYWC_HUMAN	WARS	P23381	Tryptophan--tRNA ligase, cytoplasmic (EC 6.1.1.2) (Interferon-induced protein 53) (IFP53) (Tryptophanyl-tRNA synthetase) (TrpRS) (hWRS) [Cleaved into: T1-TrpRS; T2-TrpRS]	53 kDa	0.28	52	21	31	1	6	3	11	2	20	6	4	1.6230873 16	95% (0.047)
B4DTA2_HUMA N	ATD3A_HUMA N	ATAD3A	Q9NVI7	ATPase family AAA domain-containing protein 3A	71 kDa	0.26	70	28	41	10	4	6	8	13	6	8	14	1.6175825 61	95% (0.027)
MGST3_HUMA N (+2)	DKC1_HUMAN	DKC1	O60832	H/ACA ribonucleoprotein complex subunit 4 (EC 5.4.99.-) (CBF5 homolog) (Dyskerin) (Nopp140-associated protein of 57 kDa) (Nucleolar protein NAP57) (Nucleolar protein family A member 4) (snoRNP protein DKC1)	58 kDa	0.24	25	10	15	3	2	4	2	5	2	4	4	1.6173515 37	0% (0.14)
ELAV1_HUMA N	B4E1Z4_HUMA N	CFB	B4E1Z4	Complement factor B (Uncharacterized protein) (cDNA FLJ55673, highly similar to Complement factor B (EC 3.4.21.47))	141 kDa	0.28	24	10	15	1	3	5	1	3	4	2	6	1.6173515 37	0% (0.14)
PGH1_HUMAN (+2)	IF2M_HUMAN	MTIF2	P46199	Translation initiation factor IF-2, mitochondrial (IF-2(Mt)) (IF-2Mt) (IF2(mt))	81 kDa	0.22	25	10	15	2	0	3	5	10	0	4	1	1.6173515 37	0% (0.14)
SERA_HUMAN	ARP10_HUMAN	ACTR10	Q9NZ32	Actin-related protein 10 (Actin-related protein 11) (hARP11)	46 kDa	0.18	3	1	2	0	1	0	0	0	1	0	1	1.6172360 9	0% (0.46)
B5BTZ6_HUMA N (+2)	H0Y2S9_HUMA N	MPRIP	H0Y2S9	Myosin phosphatase Rho-interacting protein (Fragment)	203 kDa	0.26	3	1	2	1	0	0	0	0	0	1	1	1.6172360 9	0% (0.46)
H0YK72_HUMA N (+4)	NIPBL_HUMAN	NIPBL	Q6KC79	Nipped-B-like protein (Delangin) (SCC2 homolog)	316 kDa	0.24	3	1	2	0	0	0	1	2	0	0	0	1.6172360 9	0% (0.46)
DDX21_HUMA N	RRP1B_HUMA N	RRP1B	Q14684	Ribosomal RNA processing protein 1 homolog B (RRP1-like protein B)	84 kDa	0.2	3	1	2	0	1	0	0	0	0	2	0	1.6172360 9	0% (0.46)
RS11_HUMAN	Q6AHZ7_HUM AN	DKFZp686A11 1	Q6AHZ7	Putative uncharacterized protein DKFZp686A111	239 kDa	0.36	3	1	2	0	1	0	0	0	0	1	1	1.6172360 9	0% (0.46)
B1AVU8_HUM AN (+4)	ABCA1_HUMA N	ABCA1	O95477	ATP-binding cassette sub-family A member 1 (ATP-binding cassette transporter 1)	254 kDa	0.18	3	1	2	0	1	0	0	0	0	1	1	1.6172360 9	0% (0.46)

				(ABC-1) (ATP-binding cassette 1) (Cholesterol efflux regulatory protein)															
Q86VX4_HUMAN (+1)	CHERP_HUMAN	CHERP	Q8IWX8	Calcium homeostasis endoplasmic reticulum protein (ERPROT 213-21) (SR-related CTD-associated factor 6)	104 kDa	0.19	3	1	2	0	0	1	0	1	0	0	1	1.61723609	0% (0.46)
ATP5H_HUMAN	LONP2_HUMAN	LONP2	Q86WA8	Lon protease homolog 2, peroxisomal (EC 3.4.21.-) (Lon protease-like protein 2) (Lon protease 2) (Peroxisomal Lon protease)	95 kDa	0.4	3	1	2	1	0	0	0	0	1	1	0	1.61723609	0% (0.46)
DHSB_HUMAN	GLRX3_HUMAN	GLRX3	O76003	Glutaredoxin-3 (PKC-interacting cousin of thioredoxin) (PICOT) (PKC-theta-interacting protein) (PKCq-interacting protein) (Thioredoxin-like protein 2)	37 kDa	0.3	3	1	2	0	1	0	0	0	2	0	0	1.61723609	0% (0.46)
IPO7_HUMAN	A0AV58_HUMAN	STRN3	A0AV58	Striatin, calmodulin binding protein 3 (cDNA FLJ77970, highly similar to Homo sapiens nuclear autoantigen GS2NA mRNA)	78 kDa	0.13	3	1	2	0	0	1	0	2	0	0	0	1.61723609	0% (0.46)
SYNE2_HUMAN	GBRL2_HUMAN	GABARAPL2	P60520	Gamma-aminobutyric acid receptor-associated protein-like 2 (GABA(A) receptor-associated protein-like 2) (Ganglioside expression factor 2) (GEF-2) (General protein transport factor p16) (Golgi-associated ATPase enhancer of 16 kDa) (GATE-16) (MAP1 light chain 3-related protein)	14 kDa	0.04	3	1	2	0	1	0	0	1	0	1	0	1.61723609	0% (0.46)
B3KM81_HUMAN (+2)	APOBR_HUMAN	APOBR	Q0VD83	Apolipoprotein B receptor (Apolipoprotein B-100 receptor) (Apolipoprotein B-48 receptor) (Apolipoprotein B48 receptor) (apoB-48R)	115 kDa	0.14	3	1	2	0	0	1	0	0	0	0	2	1.61723609	0% (0.46)
Q5NKV8_HUMAN	MSPD2_HUMAN	MOSPD2	Q8NHP6	Motile sperm domain-containing protein 2	60 kDa	0.25	3	1	2	0	0	1	0	0	0	2	0	1.61723609	0% (0.46)
H3BT71_HUMAN (+1)	QSOX2_HUMAN	QSOX2	Q6ZRP7	Sulfhydryl oxidase 2 (EC 1.8.3.2) (Neuroblastoma-derived sulfhydryl oxidase) (Quiescin Q6-like protein 1)	78 kDa	0.2	3	1	2	0	0	0	1	1	0	0	1	1.61723609	0% (0.46)
B3KXC3_HUMAN (+2)	B4DGF8_HUMAN		B4DGF8	cDNA FLJ57877, highly similar to Cleavage and polyadenylation specificity factor 7	51 kDa	0.44	3	1	2	0	0	0	1	0	1	1	0	1.61723609	0% (0.46)
PRDX4_HUMAN	WDR12_HUMAN	WDR12	Q9GZL7	Ribosome biogenesis protein WDR12 (WD repeat-containing protein 12)	48 kDa	0.23	3	1	2	0	1	0	0	2	0	0	0	1.61723609	0% (0.46)
PSMD1_HUMAN	G9JXB8_HUMAN		G9JXB8	RON variant E2E3	141 kDa	0.17	3	1	2	0	0	1	0	0	0	2	0	1.61723609	0% (0.46)
B0YIW6_HUMAN (+2)	GMPPB_HUMAN	GMPPB	Q9Y5P6	Mannose-1-phosphate guanyltransferase beta (EC 2.7.7.13) (GDP-mannose pyrophosphorylase B) (GTP-mannose-1-phosphate guanylyltransferase beta)	40 kDa	0.14	3	1	2	0	0	0	1	0	2	0	0	1.61723609	0% (0.46)
Q6FHK9_HUMAN	E9PJ81_HUMAN	UBXN1	E9PJ81	UBX domain-containing protein 1 (Fragment)	33 kDa	0.38	3	1	2	0	1	0	0	1	1	0	0	1.61723609	0% (0.46)
Q6GMX0_HUMAN	NO66_HUMAN	NO66	Q9H6W3	Bifunctional lysine-specific demethylase and histidyl-hydroxylase NO66 (EC 1.14.11.-) (EC 1.14.11.27) (60S ribosomal protein L8 histidine hydroxylase) (Histone lysine demethylase NO66) (Myc-associated protein with JmjC domain) (Nucleolar protein 66) (hsNO66) (Ribosomal oxygenase NO66) (ROX)	71 kDa	0.28	3	1	2	0	0	1	0	0	2	0	0	1.61723609	0% (0.46)
B7ZLH8_HUMAN (+2)	B4DFL3_HUMAN		B4DFL3	cDNA FLJ56661, highly similar to Proteasome subunit beta type 4 (EC 3.4.25.1)	18 kDa	0.16	3	1	2	0	0	0	1	0	0	1	1	1.61723609	0% (0.46)
B4DYX8_HUMAN (+1)	A8MY43_HUMAN	BCKDK	A8MY43	Branched chain ketoacid dehydrogenase kinase, isoform CRA_c ([3-methyl-2-	41 kDa	0.15	3	1	2	1	0	0	0	0	1	0	1	1.61723609	0% (0.46)

				oxobutanoate dehydrogenase [lipoamide]] kinase, mitochondrial)															
MPPA_HUMAN	HSP7E_HUMAN	HSPA14	Q0VDF9	Heat shock 70 kDa protein 14 (HSP70-like protein 1) (Heat shock protein HSP60)	55 kDa	0.18	3	1	2	0	0	1	0	0	1	1	0	1.6172360 9	0% (0.46)
Q53G61_HUMA N (+1)	TPPC5_HUMAN	TRAPPC5	Q8IUR0	Trafficking protein particle complex subunit 5	21 kDa	0.28	3	1	2	0	0	1	0	0	1	1	0	1.6172360 9	0% (0.46)
B4DZI8_HUMA N (+1)	B5BUI8_HUMA N	DUSP3	B5BUI8	Dual specificity phosphatase 3 (Fragment)	21 kDa	0.24	3	1	2	0	0	0	1	0	2	0	0	1.6172360 9	0% (0.46)
H2AZ_HUMAN	A8K3S0_HUMA N		A8K3S0	cDNA FLJ76127, highly similar to Homo sapiens replication factor C (activator 1) 5, 36.5kDa (RFC5), transcript variant 1, mRNA	38 kDa	0.31	3	1	2	0	0	1	0	2	0	0	0	1.6172360 9	0% (0.46)
FXR1_HUMAN	TRMB_HUMAN	METTL1	Q9UBP6	tRNA (guanine-N(7)-)-methyltransferase (EC 2.1.1.33) (Methyltransferase-like protein 1) (tRNA (guanine(46)-N(7))-methyltransferase) (tRNA(m7G46)-methyltransferase)	31 kDa	0.12	3	1	2	0	0	0	1	2	0	0	0	1.6172360 9	0% (0.46)
A8K7N0_HUMA N	Q58F09_HUMA N	GCS1	Q58F09	Glucosidase I	92 kDa	0.23	125	51	74	14	9	15	13	13	22	14	26	1.6131091 29	95% (0.0043)
Q53HV2_HUMA N (+2)	TR150_HUMAN	THRAP3	Q9Y2W1	Thyroid hormone receptor-associated protein 3 (Thyroid hormone receptor-associated protein complex 150 kDa component) (Trap150)	109 kDa	0.29	20	8	12	1	2	2	3	6	0	3	3	1.6038736 19	0% (0.18)
IF2B3_HUMAN	TBL3_HUMAN	TBL3	Q12788	Transducin beta-like protein 3 (WD repeat-containing protein SAZD)	89 kDa	0.25	20	8	12	1	2	2	3	6	2	4	0	1.6038736 19	0% (0.18)
RAB10_HUMA N	B3KRI2_HUMA N	NDUFS7	B3KRI2	NADH dehydrogenase [ubiquinone] iron-sulfur protein 7, mitochondrial (cDNA FLJ34304 fis, clone FEBRA2007880, highly similar to NADH-ubiquinone oxidoreductase 20 kDa subunit, mitochondrial (EC 1.6.5.3))	22 kDa	0.5	20	8	12	4	2	1	1	4	2	2	4	1.6038736 19	0% (0.18)
B7ZM99_HUMA N (+1)	AAKG1_HUMA N	PRKAG1	P54619	5'-AMP-activated protein kinase subunit gamma-1 (AMPK gamma1) (AMPK subunit gamma-1) (AMPKg)	38 kDa	0.15	20	8	12	1	3	2	2	3	7	2	0	1.6038736 19	0% (0.18)
1433E_HUMAN	PSMD6_HUMA N	PSMD6	Q15008	26S proteasome non-ATPase regulatory subunit 6 (26S proteasome regulatory subunit RPN7) (26S proteasome regulatory subunit S10) (Breast cancer-associated protein SGA-113M) (Phosphonoformate immuno-associated protein 4) (Proteasome regulatory particle subunit p44S10) (p42A)	46 kDa	0.53	54	22	32	1	6	5	11	3	19	6	4	1.6014432 06	95% (0.049)
IPYR_HUMAN	PYC_HUMAN	PC	P11498	Pyruvate carboxylase, mitochondrial (EC 6.4.1.1) (Pyruvic carboxylase) (PCB)	130 kDa	0.38	116	48	69	19	15	14	0	28	10	9	22	1.5976039 12	95% (0.0064)
SYDM_HUMAN	B4E0U6_HUMA N		B4E0U6	cDNA FLJ53687, highly similar to Hsc70-interacting protein	40 kDa	0.13	15	6	9	0	2	3	1	5	1	3	0	1.5829757 93	0% (0.23)
G6PE_HUMAN (+1)	A8K521_HUMA N		A8K521	cDNA FLJ78580, highly similar to Homo sapiens MCM5 minichromosome maintenance deficient 5, cell division cycle 46 (MCM5), mRNA	82 kDa	0.19	15	6	9	0	3	2	1	5	1	3	0	1.5829757 93	0% (0.23)
Q9BWC7_HUM AN	E9PH70_HUMA N	KIDINS220	E9PH70	Kinase D-interacting substrate of 220 kDa (Fragment)	145 kDa	0.4	15	6	9	2	2	1	1	2	0	3	4	1.5829757 93	0% (0.23)
B4E3A8_HUMA N (+1)	TM9S4_HUMA N	TM9SF4	Q92544	Transmembrane 9 superfamily member 4	75 kDa	0.28	15	6	9	2	1	3	0	2	3	1	3	1.5829757 93	0% (0.23)
TOP2A_HUMA N	FABP5_HUMAN	FABP5	Q01469	Fatty acid-binding protein, epidermal (Epidermal-type fatty acid-binding protein) (E-FABP) (Fatty acid-binding	15 kDa	0.14	18	6	9	1	3	0	2	2	4	1	2	1.5829757 93	0% (0.23)

				protein 5) (Psoriasis-associated fatty acid-binding protein homolog) (PA-FABP)															
CCD47_HUMAN	B2R9T9_HUMAN		B2R9T9	cDNA, FLJ94551	26 kDa	0.17	15	6	9	2	1	1	2	1	4	2	2	1.582975793	0% (0.23)
E7EW20_HUMAN (+1)	S10AE_HUMAN	S100A14	Q9HCY8	Protein S100-A14 (S100 calcium-binding protein A14) (S114)	12 kDa	0.18	15	6	9	3	2	1	0	3	0	2	4	1.582975793	0% (0.23)
M1V485_HUMAN	C9J2G3_HUMAN	OXSM	C9J2G3	3-oxoacyl-[acyl-carrier-protein] synthase, mitochondrial (Fragment)	24 kDa	0.076	15	6	9	1	1	1	3	8	1	0	0	1.582975793	0% (0.23)
PLXB2_HUMAN	SERA_HUMAN	PHGDH	O43175	D-3-phosphoglycerate dehydrogenase (3-PGDH) (EC 1.1.1.95)	57 kDa	0.11	67	28	40	2	8	8	10	12	14	10	4	1.579273853	95% (0.034)
DSG2_HUMAN	B3KMV8_HUMAN		B3KMV8	cDNA FLJ12766 fis, clone NT2RP2001520, highly similar to Calcium-binding mitochondrial carrier protein Aralar1	75 kDa	0.072	147	60	85	22	9	8	22	25	15	22	25	1.577302871	95% (0.0034)
B2R769_HUMAN (+1)	PBIP1_HUMAN	PBXIP1	Q96AQ6	Pre-B-cell leukemia transcription factor-interacting protein 1 (Hematopoietic PBX-interacting protein)	81 kDa	0.19	40	16	23	4	5	6	2	5	1	5	12	1.574137745	0% (0.092)
RBP2_HUMAN	B4DDU6_HUMAN		B4DDU6	cDNA FLJ50442, highly similar to T-complex protein 1 subunit epsilon	55 kDa	0.056	51	21	30	0	8	8	5	7	14	8	2	1.572736394	0% (0.062)
HEM6_HUMAN	ABHDB_HUMAN	ABHD11	Q8NFV4	Alpha/beta hydrolase domain-containing protein 11 (Abhydrolase domain-containing protein 11) (EC 3.-.-.-) (Williams-Beuren syndrome chromosomal region 21 protein)	35 kDa	0.24	99	41	58	10	6	7	18	20	18	6	14	1.570583254	95% (0.014)
Q96CP5_HUMAN	UBA1_HUMAN	UBA1	P22314	Ubiquitin-like modifier-activating enzyme 1 (Protein A1S9) (Ubiquitin-activating enzyme E1)	118 kDa	0.18	178	74	104	10	11	24	29	23	36	28	19	1.566817293	95% (0.0015)
Q53GX7_HUMAN (+1)	Q5TB53_HUMAN	TM9SF3	Q5TB53	Transmembrane 9 superfamily member 3 (Fragment)	30 kDa	0.25	35	14	20	6	3	3	3	4	7	5	4	1.560276597	0% (0.11)
E9PC28_HUMAN (+1)	ARGI2_HUMAN	ARG2	P78540	Arginase-2, mitochondrial (EC 3.5.3.1) (Kidney-type arginase) (Non-hepatic arginase) (Type II arginase)	39 kDa	0.13	21	9	13	7	0	2	0	7	5	0	1	1.556638134	0% (0.18)
CNPY2_HUMAN	D3DTC2_HUMAN	AYTL2	D3DTC2	Acyltransferase like 2, isoform CRA_a	54 kDa	0.52	22	9	13	7	2	0	0	2	1	5	5	1.556638134	0% (0.18)
MRP1_HUMAN	A8K5A4_HUMAN		A8K5A4	cDNA FLJ76826, highly similar to Homo sapiens ceruloplasmin (ferroxidase) (CP), mRNA	122 kDa	0.11	53	22	31	3	8	7	5	12	2	12	4	1.553257147	0% (0.063)
TNPO1_HUMAN	FUMH_HUMAN	FH	P07954	Fumarate hydratase, mitochondrial (Fumarase) (EC 4.2.1.2)	55 kDa	0.18	84	35	49	9	12	8	6	21	8	7	13	1.552403466	95% (0.025)
AL9A1_HUMAN (+1)	TXD12_HUMAN	TXNDC12	O95881	Thioredoxin domain-containing protein 12 (EC 1.8.4.2) (Endoplasmic reticulum resident protein 18) (ER protein 18) (ERp18) (Endoplasmic reticulum resident protein 19) (ER protein 19) (ERp19) (Thioredoxin-like protein p19) (hTLP19)	19 kDa	0.21	40	17	24	4	5	5	3	6	5	4	9	1.549242032	0% (0.094)
B4DJ38_HUMAN (+1)	J9JIE6_HUMAN	TMCO1	J9JIE6	Transmembrane and coiled-coil domain-containing protein 1 (Transmembrane and coiled-coil domains 1, isoform CRA_b)	27 kDa	0.063	41	17	24	3	4	2	8	3	10	5	6	1.549242032	0% (0.094)
B4DDU6_HUMAN (+1)	B7Z4V2_HUMAN		B7Z4V2	cDNA FLJ51907, highly similar to Stress-70 protein, mitochondrial	72 kDa	0.28	659	277	383	74	88	73	44	160	51	80	94	1.549156471	95% (< 0.00010)
B7Z507_HUMAN	B7Z6B8_HUMAN	DECR1	B7Z6B8	2,4-dienoyl-CoA reductase, mitochondrial (cDNA FLJ50204, highly similar to 2,4-dienoyl-CoA reductase, mitochondrial (EC 1.3.1.34))	35 kDa	0.2	92	38	53	9	8	9	12	16	16	10	12	1.547907091	95% (0.021)
B4E1U9_HUMAN	QPCTL_HUMAN	QPCTL	Q9NXS2	Glutaminyl-peptide cyclotransferase-like protein (EC 2.3.2.5) (Golgi-resident glutaminyl-peptide	43 kDa	0.22	10	4	6	0	1	1	2	0	4	1	1	1.546178161	0% (0.31)

				cyclotransferase) (isoQC) (gQC)														
Q53H34_HUMAN (+2)	KPCA_HUMAN	PRKCA	P17252	Protein kinase C alpha type (PKC-A) (PKC-alpha) (EC 2.7.11.13)	77 kDa	0.19	10	4	6	0	2	1	1	0	1	1	4	1.546178161	0% (0.31)
SYAC_HUMAN	ADA17_HUMAN	ADAM17	P78536	Disintegrin and metalloproteinase domain-containing protein 17 (ADAM 17) (EC 3.4.24.86) (Snake venom-like protease) (TNF-alpha convertase) (TNF-alpha-converting enzyme) (CD antigen CD156b)	93 kDa	0.19	10	4	6	2	0	2	0	4	0	1	1	1.546178161	0% (0.31)
B2R8J0_HUMAN (+1)	PCP_HUMAN	PRCP	P42785	Lysosomal Pro-X carboxypeptidase (EC 3.4.16.2) (Angiotensinase C) (Lysosomal carboxypeptidase C) (Proline carboxypeptidase) (Prolylcarboxypeptidase) (PRCP)	56 kDa	0.15	10	4	6	0	1	3	0	0	0	5	1	1.546178161	0% (0.31)
GRHPR_HUMAN (+1)	MGST2_HUMAN	MGST2	Q99735	Microsomal glutathione S-transferase 2 (Microsomal GST-2) (EC 2.5.1.18) (Microsomal GST-II)	17 kDa	0.27	10	4	6	1	1	0	2	2	2	1	1	1.546178161	0% (0.31)
TEX10_HUMAN	ARL2_HUMAN	ARL2	P36404	ADP-ribosylation factor-like protein 2	21 kDa	0.14	10	4	6	0	0	1	3	2	3	1	0	1.546178161	0% (0.31)
AP2M1_HUMAN (+1)	B7WP27_HUMAN	CWC22	B7WP27	Pre-mRNA-splicing factor CWC22 homolog	106 kDa	0.25	10	4	6	1	1	1	1	2	0	2	2	1.546178161	0% (0.31)
LEMD2_HUMAN	GCP60_HUMAN	ACBD3	Q9H3P7	Golgi resident protein GCP60 (Acyl-CoA-binding domain-containing protein 3) (Golgi complex-associated protein 1) (GOCAP1) (Golgi phosphoprotein 1) (GOLPH1) (PBR- and PKA-associated protein 7) (Peripheral benzodiazepine receptor-associated protein PAP7)	61 kDa	0.21	9	4	6	1	0	0	3	3	3	0	0	1.546178161	0% (0.31)
Q6N097_HUMAN	UBP10_HUMAN	USP10	Q14694	Ubiquitin carboxyl-terminal hydrolase 10 (EC 3.4.19.12) (Deubiquitinating enzyme 10) (Ubiquitin thioesterase 10) (Ubiquitin-specific-processing protease 10)	87 kDa	0.19	10	4	6	0	2	2	0	2	0	4	0	1.546178161	0% (0.31)
B2R761_HUMAN (+1)	HTRA2_HUMAN	HTRA2	O43464	Serine protease HTRA2, mitochondrial (EC 3.4.21.108) (High temperature requirement protein A2) (HtrA2) (Omi stress-regulated endoprotease) (Serine protease 25) (Serine proteinase OMI)	49 kDa	0.22	10	4	6	1	1	1	1	1	1	3	1	1.546178161	0% (0.31)
ACSF2_HUMAN (+2)	SELT_HUMAN	SELT	P62341	Selenoprotein T (SelT)	22 kDa	0.19	10	4	6	1	1	1	1	1	2	1	2	1.546178161	0% (0.31)
APOE_HUMAN	SHLB1_HUMAN	SH3GLB1	Q9Y371	Endophilin-B1 (Bax-interacting factor 1) (Bif-1) (SH3 domain-containing GRB2-like protein B1)	41 kDa	0.35	10	4	6	0	1	1	2	0	4	2	0	1.546178161	0% (0.31)
B4DPK2_HUMAN (+2)	B4E2X9_HUMAN		B4E2X9	cDNA FLJ58081, highly similar to Cleft lip and palate transmembrane protein 1	75 kDa	0.24	30	12	17	6	3	1	2	1	5	6	6	1.542239809	0% (0.14)
H0Y368_HUMAN	I1SRC5_HUMAN		I1SRC5	UBE2L3/KRAS fusion protein	34 kDa	0.28	29	12	17	1	1	3	7	2	7	6	3	1.542239809	0% (0.14)
Q658S9_HUMAN (+3)	DRG1_HUMAN	DRG1	Q9Y295	Developmentally-regulated GTP-binding protein 1 (DRG-1) (Neural precursor cell expressed developmentally down-regulated protein 3) (NEDD-3)	41 kDa	0.18	29	12	17	0	1	4	7	4	8	5	0	1.542239809	0% (0.14)
OCAD1_HUMAN	B2RCM2_HUMAN		B2RCM2	cDNA, FLJ96156, highly similar to Homo sapiens leucyl-tRNA synthetase (LARS), mRNA	134 kDa	0.3	48	20	28	2	6	7	5	16	5	6	1	1.541331124	0% (0.078)
B4DGU4_HUMAN (+4)	DYHC1_HUMAN	DYNC1H1	Q14204	Cytoplasmic dynein 1 heavy chain 1 (Cytoplasmic dynein heavy chain 1) (Dynein heavy chain, cytosolic)	532 kDa	0.24	347	146	201	49	32	40	27	40	96	45	26	1.539362758	95% (< 0.00010)
P4HA2_HUMAN (+2)	TCPA_HUMAN	TCP1	P17987	T-complex protein 1 subunit alpha (TCP-1-alpha) (CCT-alpha)	60 kDa	0.16	75	31	43	3	11	10	8	15	15	9	5	1.536543733	95% (0.037)

Q53EW8_HUMAN (+1)	GALT2_HUMAN	GALNT2	Q10471	Polypeptide N-acetylgalactosaminyltransferase 2 (EC 2.4.1.41) (Polypeptide GalNAc transferase 2) (GalNAc-T2) (pp-GaNTase 2) (Protein-UDP acetylgalactosaminyltransferase 2) (UDP-GalNAc:polypeptide N-acetylgalactosaminyltransferase 2) [Cleaved into: Polypeptide N-acetylgalactosaminyltransferase 2 soluble form]	65 kDa	0.24	36	15	21	4	6	2	4	4	7	4	6	1.533168267	0% (0.12)
B2R7U4_HUMAN (+1)	J3KNU8_HUMAN	ALDH6A1	J3KNU8	Methylmalonate-semialdehyde dehydrogenase [acylating], mitochondrial	56 kDa	0.36	42	18	25	6	5	4	3	12	3	6	4	1.526933065	0% (0.097)
M0QWZ7_HUMAN (+1)	RPB1_HUMAN	POLR2A	P24928	DNA-directed RNA polymerase II subunit RPB1 (RNA polymerase II subunit B1) (EC 2.7.7.6) (DNA-directed RNA polymerase II subunit A) (DNA-directed RNA polymerase III largest subunit) (RNA-directed RNA polymerase II subunit RPB1) (EC 2.7.7.48)	217 kDa	0.24	17	7	10	0	2	3	2	0	1	6	3	1.526819354	0% (0.23)
B4DZK8_HUMAN (+2)	B4DNJ6_HUMAN	STRAP	B4DNJ6	Serine-threonine kinase receptor-associated protein (cDNA FLJ51909, highly similar to Serine-threonine kinase receptor-associatedprotein)	40 kDa	0.33	17	7	10	1	5	1	0	2	5	3	0	1.526819354	0% (0.23)
GNAS1_HUMAN	E7ER89_HUMAN	PAPSS2	E7ER89	Sulfate adenylyltransferase	70 kDa	0.083	17	7	10	0	3	3	1	0	8	2	0	1.526819354	0% (0.23)
LACTB_HUMAN	B4DEI4_HUMAN		B4DEI4	cDNA FLJ54595, highly similar to Golgi reassembly-stacking protein 2	42 kDa	0.2	17	7	10	2	1	1	3	2	2	3	3	1.526819354	0% (0.23)
Q6IQ30_HUMAN	GOGA2_HUMAN	GOLGA2	Q08379	Golgin subfamily A member 2 (130 kDa cis-Golgi matrix protein) (GM130) (GM130 autoantigen) (Golgin-95)	113 kDa	0.22	17	7	10	0	1	2	3	2	2	6	0	1.526819354	0% (0.23)
E7ETK0_HUMAN (+1)	A7YA96_HUMAN	GGCX	A7YA96	Gamma-glutamyl carboxylase (Gamma-glutymyl carboxylase) (cDNA, FLJ93613, highly similar to Homo sapiens gamma-glutamyl carboxylase (GGCX), mRNA)	88 kDa	0.36	17	7	10	4	1	1	1	4	0	3	3	1.526819354	0% (0.23)
B4DZQ5_HUMAN	CN166_HUMAN	C14orf166	Q9Y224	UPF0568 protein C14orf166 (CLE7 homolog) (CLE)	28 kDa	0.1	50	21	29	4	3	5	9	7	9	7	7	1.522386921	0% (0.080)
B7Z809_HUMAN (+3)	B4DJQ5_HUMAN		B4DJQ5	cDNA FLJ59211, highly similar to Glucosidase 2 subunit beta	60 kDa	0.18	83	35	48	7	8	11	10	13	5	17	13	1.521487964	95% (0.032)
PDCD6_HUMAN	B7Z809_HUMAN		B7Z809	cDNA FLJ56016, highly similar to C-1-tetrahydrofolate synthase, cytoplasmic	110 kDa	0.28	57	24	33	1	7	8	7	10	13	9	1	1.518927796	0% (0.066)
PLD3_HUMAN	B2R679_HUMAN		B2R679	cDNA, FLJ92825, highly similar to Homo sapiens SAR1a gene homolog 1 (S. cerevisiae) (SARA1), mRNA	22 kDa	0.13	24	10	14	1	2	2	5	2	9	2	1	1.517800374	0% (0.18)
H3BN98_HUMAN	AIMP2_HUMAN	AIMP2	Q13155	Aminoacyl tRNA synthase complex-interacting multifunctional protein 2 (Multisynthase complex auxiliary component p38) (Protein JTV-1)	35 kDa	0.27	24	10	14	1	1	3	5	2	9	1	2	1.517800374	0% (0.18)
A8K6Y1_HUMAN	BIEA_HUMAN	BLVRA	P53004	Biliverdin reductase A (BVR A) (EC 1.3.1.24) (Biliverdin-IX alpha-reductase)	33 kDa	0.28	24	10	14	3	2	2	3	3	5	2	4	1.517800374	0% (0.18)
ITB4_HUMAN	A8K335_HUMAN		A8K335	cDNA FLJ76254, highly similar to Homo sapiens gamma-glutamyl hydrolase (GGH), mRNA	36 kDa	0.15	31	13	18	1	5	4	3	3	2	9	4	1.512590806	0% (0.15)
BDH_HUMAN	PNPT1_HUMAN	PNPT1	Q8TCS8	Polyribonucleotide nucleotidyltransferase 1, mitochondrial (EC 2.7.7.8) (3'-5' RNA exonuclease OLD35) (PNPase old-35)	86 kDa	0.23	92	39	53	7	11	11	11	22	11	8	12	1.509430369	95% (0.027)

				(Polynucleotide phosphorylase 1) (PNPase 1) (Polynucleotide phosphorylase-like protein)															
B5BU61_HUMAN (+1)	RM44_HUMAN	MRPL44	Q9H9J2	39S ribosomal protein L44, mitochondrial (L44mt) (MRP-L44) (EC 3.1.26.-)	38 kDa	0.15	38	16	22	6	3	2	5	11	2	3	7	1.509203132	0% (0.12)
B4DZ88_HUMAN (+1)	B3KTT0_HUMAN		B3KTT0	cDNA FLJ38675 fis, clone IMR322000243, highly similar to Nucleoporin Nup43	42 kDa	0.13	38	16	22	0	3	5	8	7	5	6	4	1.509203132	0% (0.12)
A8K032_HUMAN (+3)	A8K2U2_HUMAN		A8K2U2	cDNA FLJ75392, highly similar to Homo sapiens hexokinase II (HKII) mRNA	102 kDa	0.12	38	16	22	3	3	2	8	7	6	6	3	1.509203132	0% (0.12)
TM9S2_HUMAN	Q9H9B7_HUMAN		Q9H9B7	Coatomer subunit gamma	89 kDa	0.1	38	16	22	2	0	3	9	7	8	5	0	1.509203132	0% (0.12)
SYNC_HUMAN	Q8TA92_HUMAN		Q8TA92	Similar to AFG3 ATPase family gene 3-like 2 (Yeast) (Fragment)	90 kDa	0.18	105	45	61	21	8	10	7	20	10	15	16	1.507382142	95% (0.020)
Q7Z3K9_HUMAN	ITB1_HUMAN	ITGB1	P05556	Integrin beta-1 (Fibronectin receptor subunit beta) (Glycoprotein IIa) (GPIIA) (VLA-4 subunit beta) (CD antigen CD29)	88 kDa	0.11	45	19	26	5	4	5	5	3	3	7	13	1.506827624	0% (0.099)
C9JFR7_HUMAN (+1)	F8WCY5_HUMAN	RCN2	F8WCY5	Reticulocalbin-2	39 kDa	0.4	44	19	26	4	4	3	8	4	6	7	9	1.506827624	0% (0.099)
Q53HH4_HUMAN	Q86VX4_HUMAN	SMC3	Q86VX4	Structural maintenance of chromosomes 3	142 kDa	0.19	52	22	30	6	7	7	2	13	1	13	3	1.505072476	0% (0.081)
ADPGK_HUMAN	B3KNT8_HUMAN	NAP1L1	B3KNT8	Nucleosome assembly protein 1-like 1 (cDNA FLJ30458 fis, clone BRACE2009421, highly similar to NUCLEOSOME ASSEMBLY PROTEIN 1-LIKE 1)	43 kDa	0.14	52	22	30	4	4	11	3	8	11	6	5	1.505072476	0% (0.081)
GFPT1_HUMAN	HNRH3_HUMAN	HNRNPH3	P31942	Heterogeneous nuclear ribonucleoprotein H3 (hnRNP H3) (Heterogeneous nuclear ribonucleoprotein 2H9) (hnRNP 2H9)	37 kDa	0.23	66	28	38	4	8	8	7	9	8	14	7	1.502659744	0% (0.056)
SAC1_HUMAN	CCD47_HUMAN	CCDC47	Q96A33	Coiled-coil domain-containing protein 47	56 kDa	0.24	67	28	38	8	6	5	9	7	9	9	13	1.502659744	0% (0.056)
RS14_HUMAN	B4DLN1_HUMAN	SLC25A10	B4DLN1	Mitochondrial dicarboxylate carrier (Uncharacterized protein) (cDNA FLJ60124, highly similar to Mitochondrial dicarboxylate carrier)	48 kDa	0.36	78	34	46	9	9	7	10	10	18	13	4	1.501087706	95% (0.040)
HSDL2_HUMAN	CMC2_HUMAN	SLC25A13	Q9UJS0	Calcium-binding mitochondrial carrier protein Aralar2 (Citrin) (Mitochondrial aspartate glutamate carrier 2) (Solute carrier family 25 member 13)	74 kDa	0.17	244	104	139	41	18	13	32	40	31	32	37	1.492914615	95% (0.00097)
ACSL4_HUMAN (+2)	ECHM_HUMAN	ECHS1	P30084	Enoyl-CoA hydratase, mitochondrial (EC 4.2.1.17) (Enoyl-CoA hydratase 1) (Short-chain enoyl-CoA hydratase) (SCEH)	31 kDa	0.16	194	83	111	28	15	14	25	49	21	17	24	1.492494452	95% (0.0029)
PSD11_HUMAN	B2R761_HUMAN		B2R761	cDNA, FLJ93299, highly similar to Homo sapiens sterol carrier protein 2 (SCP2), mRNA	59 kDa	0.32	53	23	31	5	5	6	8	6	11	6	9	1.489186168	0% (0.083)
H10_HUMAN	FLNA_HUMAN	FLNA	P21333	Filamin-A (FLN-A) (Actin-binding protein 280) (ABP-280) (Alpha-filamin) (Endothelial actin-binding protein) (Filamin-1) (Non-muscle filamin)	281 kDa	0.27	444	192	255	31	65	80	18	57	33	83	81	1.486391961	95% (< 0.00010)
E5RI99_HUMAN (+1)	B7Z6B3_HUMAN		B7Z6B3	cDNA FLJ53094, highly similar to Receptor expression-enhancing protein 5	18 kDa	0.35	26	11	15	4	2	0	5	3	4	2	6	1.485303754	0% (0.19)
B7Z792_HUMAN (+1)	HNRPF_HUMAN	HNRNPF	P52597	Heterogeneous nuclear ribonucleoprotein F (hnRNP F) (Nucleolin-like protein mcs94-1) [Cleaved into: Heterogeneous nuclear ribonucleoprotein F, N-terminally processed]	46 kDa	0.28	116	48	64	9	8	16	15	19	11	21	12	1.483788625	95% (0.021)
MRRP1_HUMAN	F5H3I5_HUMAN	CDK5RAP3	F5H3I5	CDK5 regulatory subunit-associated protein 3	60 kDa	0.21	19	8	11	3	3	1	1	2	1	5	3	1.48280522	0% (0.24)

PCBP1_HUMAN	RT02_HUMAN	MRPS2	Q9Y399	28S ribosomal protein S2, mitochondrial (MRP-S2) (S2mt)	33 kDa	0.23	18	8	11	2	2	1	3	2	3	2	4	1.48280522	0% (0.24)
B7Z4B8_HUMAN	CPSF6_HUMAN	CPSF6	Q16630	Cleavage and polyadenylation specificity factor subunit 6 (Cleavage and polyadenylation specificity factor 68 kDa subunit) (CFIm68) (CPSF 68 kDa subunit) (Pre-mRNA cleavage factor Im 68 kDa subunit) (Protein HPBRII-4/7)	59 kDa	0.14	19	8	11	0	3	1	4	2	0	8	1	1.48280522	0% (0.24)
LMAN1_HUMAN (+1)	A8K813_HUMAN		A8K813	cDNA FLJ77763, highly similar to Homo sapiens prolactin regulatory element binding (PREB), mRNA	45 kDa	0.11	19	8	11	6	0	1	1	1	3	2	5	1.48280522	0% (0.24)
C1PHA2_HUMAN	K7ELC4_HUMAN	RPL22	K7ELC4	60S ribosomal protein L22 (Fragment)	9 kDa	0.085	19	8	11	1	2	2	3	3	4	1	3	1.48280522	0% (0.24)
ACTZ_HUMAN (+1)	TI23B_HUMAN	TIMM23B	Q5SRD1	Putative mitochondrial import inner membrane translocase subunit Tim23B	28 kDa	0.19	19	8	11	2	2	1	3	3	1	1	6	1.48280522	0% (0.24)
Q9BR63_HUMAN (+1)	A8K8D9_HUMAN		A8K8D9	Glucose-6-phosphate 1-dehydrogenase (EC 1.1.1.49)	59 kDa	0.15	99	42	56	3	13	15	11	11	14	21	10	1.482417773	95% (0.029)
LYAG_HUMAN	NPM_HUMAN	NPM1	P06748	Nucleophosmin (NPM) (Nucleolar phosphoprotein B23) (Nucleolar protein NO38) (Numatrin)	33 kDa	0.092	77	33	44	7	6	9	11	10	14	10	10	1.479498279	95% (0.049)
SR140_HUMAN	ADAS_HUMAN	AGPS	O00116	Alkyldihydroxyacetonephosphate synthase, peroxisomal (Alkyl-DHAP synthase) (EC 2.5.1.26) (Aging-associated gene 5 protein) (Alkylglycerone-phosphate synthase)	73 kDa	0.11	70	30	40	16	4	4	7	12	8	8	12	1.478162329	0% (0.058)
PSD12_HUMAN	SQSTM_HUMAN	SQSTM1	Q13501	Sequestosome-1 (EBI3-associated protein of 60 kDa) (EBIAP) (p60) (Phosphotyrosine-independent ligand for the Lck SH2 domain of 62 kDa) (Ubiquitin-binding protein p62)	48 kDa	0.16	12	5	7	2	2	1	0	2	3	1	2	1.477935328	0% (0.31)
RAB6B_HUMAN	PTN6_HUMAN	PTPN6	P29350	Tyrosine-protein phosphatase non-receptor type 6 (EC 3.1.3.48) (Hematopoietic cell protein-tyrosine phosphatase) (Protein-tyrosine phosphatase 1C) (PTP-1C) (Protein-tyrosine phosphatase SHP-1) (SH-PTP1)	68 kDa	0.44	12	5	7	0	1	4	0	0	0	5	3	1.477935328	0% (0.31)
Q53GX6_HUMAN	SPT6H_HUMAN	SUPT6H	Q7KZ85	Transcription elongation factor SPT6 (hSPT6) (Histone chaperone suppressor of Ty6) (Tat-cotransactivator 2 protein) (Tat-CT2 protein)	199 kDa	0.13	12	5	7	0	3	2	0	0	0	7	0	1.477935328	0% (0.31)
ARF1_HUMAN (+1)	IDE_HUMAN	IDE	P14735	Insulin-degrading enzyme (EC 3.4.24.56) (Abeta-degrading protease) (Insulin protease) (Insulinase) (Insulysin)	118 kDa	0.31	12	5	7	1	1	1	2	1	4	1	1	1.477935328	0% (0.31)
ITB1_HUMAN	Q53FV3_HUMAN		Q53FV3	COP9 signalosome subunit 4 variant (Fragment)	46 kDa	0.14	11	5	7	0	1	2	2	3	2	2	0	1.477935328	0% (0.31)
Q53GN4_HUMAN (+3)	ATG9A_HUMAN	ATG9A	Q7Z3C6	Autophagy-related protein 9A (APG9-like 1) (mATG9)	94 kDa	0.1	12	5	7	1	2	1	1	1	4	1	1	1.477935328	0% (0.31)
A6QKW0_HUMAN (+1)	J3K000_HUMAN	PEPD	J3K000	PEPD protein	55 kDa	0.29	12	5	7	1	2	1	1	2	2	1	2	1.477935328	0% (0.31)
Q6NVW7_HUMAN	THOC6_HUMAN	THOC6	Q86W42	THO complex subunit 6 homolog (Functional spliceosome-associated protein 35) (fSAP35) (WD repeat-containing protein 58)	38 kDa	0.25	12	5	7	0	0	2	3	2	1	3	1	1.477935328	0% (0.31)
DHX30_HUMAN (+1)	MCTS1_HUMAN	MCTS1	Q9ULC4	Malignant T-cell-amplified sequence 1 (MCT-1) (Multiple copies T-cell malignancies)	21 kDa	0.14	12	5	7	0	0	0	5	3	3	1	0	1.477935328	0% (0.31)
GRPE1_HUMAN	CAYP1_HUMAN	CAPS	Q13938	Calcyphosin (Calcyphosine)	21 kDa	0.37	12	5	7	1	0	0	4	7	0	0	0	1.477935328	0% (0.31)
ECI1_HUMAN	APOE_HUMAN	APOE	P02649	Apolipoprotein E (Apo-E)	36 kDa	0.18	57	24	32	5	3	3	13	0	16	6	11	1.474558318	0% (0.084)
AAAS_HUMAN	AIFM1_HUMAN	AIFM1	O95831	Apoptosis-inducing factor 1, mitochondrial (EC 1.1.1.-) (Programmed cell death protein 8)	67 kDa	0.2	175	75	99	20	20	14	21	39	17	23	21	1.472686898	95% (0.0059)

USP9X_HUMAN	LPPRC_HUMAN	LRPPRC	P42704	Leucine-rich PPR motif-containing protein, mitochondrial (130 kDa leucine-rich protein) (LRP 130) (GP130)	158 kDa	0.088	539	234	306	56	66	58	53	118	46	66	79	1.464320483	95% (< 0.00010)
DHRS7_HUMAN	NIPS2_HUMAN	GBAS	O75323	Protein NipSnap homolog 2 (NipSnap2) (Glioblastoma-amplified sequence)	34 kDa	0.28	35	15	20	1	3	2	9	4	10	3	3	1.46424076	0% (0.15)
B2RBL3_HUMAN (+1)	INF2_HUMAN	INF2	Q27J81	Inverted formin-2 (HBEBP2-binding protein C)	136 kDa	0.34	34	15	20	3	4	6	2	2	3	15	0	1.46424076	0% (0.15)
B2RD27_HUMAN (+1)	MYEF2_HUMAN	MYEF2	Q9P2K5	Myelin expression factor 2 (MEF-2) (MyEF-2) (MST156)	64 kDa	0.24	5	2	3	0	0	0	2	1	0	2	0	1.464127231	0% (0.45)
ARPC3_HUMAN (+2)	G3XAB3_HUMAN	TTC17	G3XAB3	Tetratricopeptide repeat domain 17, isoform CRA_c (Tetratricopeptide repeat protein 17)	109 kDa	0.25	5	2	3	3	0	0	0	1	0	1	1	1.464127231	0% (0.45)
B2R6N9_HUMAN (+5)	B7Z2D6_HUMAN		B7Z2D6	cDNA FLJ53863, highly similar to Cystathionine beta-synthase (EC 4.2.1.22)	56 kDa	0.12	3	2	3	0	0	1	1	1	1	1	0	1.464127231	0% (0.45)
CD82_HUMAN (+2)	Q5S9Q5_HUMAN	ND2	Q5S9Q5	NADH-ubiquinone oxidoreductase chain 2 (EC 1.6.5.3)	39 kDa	0.17	5	2	3	1	0	0	1	1	1	1	0	1.464127231	0% (0.45)
VATL_HUMAN	HPDL_HUMAN	HPDL	Q96IR7	4-hydroxyphenylpyruvate dioxygenase-like protein (EC 1.13.-.-) (Glyoxalase domain-containing protein 1)	39 kDa	0.16	5	2	3	0	1	1	0	2	1	0	0	1.464127231	0% (0.45)
B4DP27_HUMAN (+1)	GATM_HUMAN	GATM	P50440	Glycine amidinotransferase, mitochondrial (EC 2.1.4.1) (L-arginine:glycine amidinotransferase) (Transamidinase)	48 kDa	0.3	5	2	3	0	2	0	0	2	1	0	0	1.464127231	0% (0.45)
A8K5A4_HUMAN (+4)	A6NJZ9_HUMAN	NOC3L	A6NJZ9	Nucleolar complex protein 3 homolog	93 kDa	0.11	5	2	3	0	0	0	2	1	0	1	1	1.464127231	0% (0.45)
SMU1_HUMAN	NOC4L_HUMAN	NOC4L	Q9BVI4	Nucleolar complex protein 4 homolog (NOC4 protein homolog) (NOC4-like protein) (Nucleolar complex-associated protein 4-like protein)	58 kDa	0.16	5	2	3	0	1	0	1	0	0	3	0	1.464127231	0% (0.45)
NSDHL_HUMAN	CUL2_HUMAN	CUL2	Q13617	Cullin-2 (CUL-2)	87 kDa	0.17	5	2	3	0	0	1	1	1	1	1	0	1.464127231	0% (0.45)
B4E2A6_HUMAN (+1)	C9JZG9_HUMAN	UBE2H	C9JZG9	Ubiquitin-conjugating enzyme E2 H (Fragment)	12 kDa	0.18	5	2	3	0	0	0	2	2	0	1	0	1.464127231	0% (0.45)
B3KTQ2_HUMAN (+2)	DGLB_HUMAN	DAGLB	Q8NCG7	Sn1-specific diacylglycerol lipase beta (DGL-beta) (EC 3.1.1.-) (KCCR13L)	74 kDa	0.23	5	2	3	2	0	0	0	0	1	1	1	1.464127231	0% (0.45)
H0Y4R1_HUMAN (+1)	NADE_HUMAN	NADSYN1	Q6IA69	Glutamine-dependent NAD(+) synthetase (EC 6.3.5.1) (NAD(+) synthase [glutamine-hydrolyzing]) (NAD(+) synthetase)	79 kDa	0.24	5	2	3	0	1	0	1	0	3	0	0	1.464127231	0% (0.45)
Q5JQ44_HUMAN (+1)	A6NFN2_HUMAN	ABI1	A6NFN2	Abl interactor 1	55 kDa	0.34	5	2	3	1	0	1	0	0	2	0	1	1.464127231	0% (0.45)
SYG_HUMAN	G9LJ35_HUMAN	ND5	G9LJ35	NADH-ubiquinone oxidoreductase chain 5 (EC 1.6.5.3)	67 kDa	0.18	5	2	3	2	0	0	0	1	0	2	0	1.464127231	0% (0.45)
6PGD_HUMAN (+3)	BYST_HUMAN	BYSL	Q13895	Bystin	50 kDa	0.22	5	2	3	0	1	0	1	1	0	2	0	1.464127231	0% (0.45)
PDS5B_HUMAN	IKIP_HUMAN	IKBIP	Q70UQ0	Inhibitor of nuclear factor kappa-B kinase-interacting protein (I kappa-B kinase-interacting protein) (IKBKB-interacting protein) (IKK-interacting protein)	39 kDa	0.12	5	2	3	2	0	0	0	0	0	2	1	1.464127231	0% (0.45)
RS18_HUMAN	GHDC_HUMAN	GHDC	Q8N2G8	GH3 domain-containing protein	58 kDa	0.41	5	2	3	2	0	0	0	0	0	0	3	1.464127231	0% (0.45)
G3V0E8_HUMAN	ISOC1_HUMAN	ISOC1	Q96CN7	Isochorismatase domain-containing protein 1	32 kDa	0.21	5	2	3	0	1	1	0	0	0	2	1	1.464127231	0% (0.45)
B2RCM2_HUMAN (+5)	B3KN09_HUMAN		B3KN09	cDNA FLJ13173 fis, clone NT2RP3003831, highly similar to Endonuclease G-like 1 (EC 3.1.30.-)	41 kDa	0.14	5	2	3	2	0	0	0	1	2	0	1	1.464127231	0% (0.45)
LAP2A_HUMAN	MAVS_HUMAN	MAVS	Q7Z434	Mitochondrial antiviral-signaling protein (MAVS) (CARD adapter inducing interferon beta) (Cardif) (Interferon beta promoter stimulator protein 1) (IPS-1) (Putative NF-kappa-B-activating protein 031N)	57 kDa	0.22	5	2	3	1	0	0	1	1	1	1	0	1.464127231	0% (0.45)

				(Virus-induced-signaling adapter) (VISA)														
A8K9T8_HUMAN (+1)	B2R960_HUMAN		B2R960	cDNA, FLJ94230, highly similar to Homo sapiens thioredoxin-like 1 (TXNL1), mRNA	32 kDa	0.11	5	2	3	0	0	0	2	0	2	1	0	1.464127231	0% (0.45)
B7Z416_HUMAN (+1)	A8KAP9_HUMAN		A8KAP9	cDNA FLJ78448, highly similar to Homo sapiens argininosuccinate synthetase (ASS), transcript variant 1, mRNA	47 kDa	0.22	88	38	50	5	8	7	18	13	23	13	1	1.462245393	95% (0.042)
C9JNW5_HUMAN	H7BXI1_HUMAN	ESYT2	H7BXI1	Extended synaptotagmin-2 (Fragment)	98 kDa	0.31	88	38	50	20	6	5	7	9	16	13	14	1.462245393	95% (0.042)
NCBP1_HUMAN	DHSB_HUMAN	SDHB	P21912	Succinate dehydrogenase [ubiquinone] iron-sulfur subunit, mitochondrial (EC 1.3.5.1) (Iron-sulfur subunit of complex II) (Ip)	32 kDa	0.14	51	22	29	7	3	5	7	11	4	7	7	1.456889191	0% (0.10)
B0V043_HUMAN (+1)	B3KX11_HUMAN		B3KX11	cDNA FLJ44436 fis, clone UTERU2019706, highly similar to T-complex protein 1 subunit gamma	58 kDa	0.13	90	39	51	4	14	8	13	17	16	12	6	1.453741312	95% (0.043)
NUP98_HUMAN	ACSL4_HUMAN	ACSL4	O60488	Long-chain-fatty-acid--CoA ligase 4 (EC 6.2.1.3) (Long-chain acyl-CoA synthetase 4) (LACS 4)	79 kDa	0.1	66	29	38	8	9	10	2	9	7	11	10	1.452966367	0% (0.072)
OSBL8_HUMAN (+1)	CTL2_HUMAN	SLC44A2	Q8IWA5	Choline transporter-like protein 2 (Solute carrier family 44 member 2)	80 kDa	0.14	44	19	25	14	1	1	3	12	1	6	7	1.45151031	0% (0.13)
S12A2_HUMAN	A8K9T3_HUMAN		A8K9T3	cDNA FLJ76467, highly similar to Homo sapiens acyl-CoA synthetase long-chain family member 1 (ACSL1), mRNA	78 kDa	0.14	83	36	47	9	8	9	10	1	13	12	21	1.450539314	0% (0.051)
RT29_HUMAN	B4DLT2_HUMAN		B4DLT2	cDNA FLJ56637, highly similar to Nuclear pore complex protein Nup155	151 kDa	0.19	84	36	47	11	9	8	9	11	11	14	11	1.450539314	0% (0.051)
SYWC_HUMAN	DHX15_HUMAN	DHX15	O43143	Putative pre-mRNA-splicing factor ATP-dependent RNA helicase DHX15 (EC 3.6.4.13) (ATP-dependent RNA helicase #46) (DEAH box protein 15)	91 kDa	0.26	137	60	78	7	18	13	22	17	10	31	19	1.449143789	95% (0.016)
H0Y9V9_HUMAN (+1)	J3QQQ9_HUMAN	KRBA2	J3QQQ9	KRAB-A domain-containing protein 2	13 kDa	0.2	22	9	12	2	2	2	3	1	6	2	3	1.447379546	0% (0.24)
RLA2_HUMAN	WDR18_HUMAN	WDR18	Q9BV38	WD repeat-containing protein 18	47 kDa	0.56	21	9	12	5	0	0	4	6	2	1	3	1.447379546	0% (0.24)
Q6FGL0_HUMAN	E9PK47_HUMAN	PYGL	E9PK47	Phosphorylase (EC 2.4.1.1)	94 kDa	0.26	21	9	12	4	0	0	3	1	7	4	0	1.447379546	0% (0.24)
DEK_HUMAN	B4DII5_HUMAN		B4DII5	Importin subunit alpha	60 kDa	0.18	21	9	12	1	3	2	3	5	4	3	1	1.447379546	0% (0.24)
MESD_HUMAN	IF4G3_HUMAN	EIF4G3	O43432	Eukaryotic translation initiation factor 4 gamma 3 (eIF-4-gamma 3) (eIF-4G 3) (eIF4G 3) (eIF-4-gamma II) (eIF4GII)	177 kDa	0.29	21	9	12	1	3	2	0	3	3	4	2	1.447379546	0% (0.24)
Q96DV6_HUMAN (+1)	EF1G_HUMAN	EEF1G	P26641	Elongation factor 1-gamma (EF-1-gamma) (eEF-1B gamma)	50 kDa	0.16	92	40	52	3	9	10	18	10	23	12	6	1.445649634	95% (0.044)
J3KNU8_HUMAN (+1)	PDCD6_HUMAN	PDCD6	O75340	Programmed cell death protein 6 (Apoptosis-linked gene 2 protein) (Probable calcium-binding protein ALG-2)	22 kDa	0.23	37	16	21	3	3	5	6	3	2	5	11	1.444270388	0% (0.15)
PDPR_HUMAN	PR40A_HUMAN	PRPF40A	O75400	Pre-mRNA-processing factor 40 homolog A (Fas ligand-associated factor 1) (Formin-binding protein 11) (Formin-binding protein 3) (Huntingtin yeast partner A) (Huntingtin-interacting protein 10) (HIP-10) (Huntingtin-interacting protein A) (Renal carcinoma antigen NY-REN-6)	109 kDa	0.19	37	16	21	3	6	4	4	5	1	5	10	1.444270388	0% (0.15)
I7GSS2_HUMAN	Q6MZM3_HUMAN	DKFZp686C21148	Q6MZM3	Putative uncharacterized protein DKFZp686C21148	141 kDa	0.42	37	16	21	3	6	3	4	8	0	9	4	1.444270388	0% (0.15)
Q5XPV6_HUMAN	CP1B1_HUMAN	CYP1B1	Q16678	Cytochrome P450 1B1 (EC 1.14.14.1) (CYPIB1)	61 kDa	0.19	37	16	21	5	3	2	6	0	4	8	9	1.444270388	0% (0.15)
B5MCA4_HUMAN (+1)	SMCA1_HUMAN	SMARCA1	P28370	Probable global transcription activator SNF2L1 (EC 3.6.4.-) (ATP-dependent helicase	123 kDa	0.11	37	16	21	4	3	5	0	10	1	8	2	1.444270388	0% (0.15)

				SMARCA1) (Nucleosome-remodeling factor subunit SNF2L) (SWI/SNF-related matrix-associated actin-dependent regulator of chromatin subfamily A member 1)															
PSIP1_HUMAN	B3KSW0_HUMAN	CDIPT	B3KSW0	CDP-diacylglycerol--inositol 3-phosphatidyltransferase (Phosphatidylinositol synthase), isoform CRA_b (cDNA FLJ37149 fis, clone BRACE2025437, highly similar to CDP-diacylglycerol--inositol3-phosphatidyltransferase (EC 2.7.8.11))	20 kDa	0.11	53	23	30	8	4	3	8	7	8	7	8	1.442989087	0% (0.11)
PGAM1_HUMAN (+1)	A8K4W7_HUMAN		A8K4W7	cDNA FLJ76284, highly similar to Homo sapiens succinate-CoA ligase, GDP-forming, alpha subunit (SUCLG1), mRNA	35 kDa	0.31	29	13	17	4	2	2	5	7	3	4	3	1.433994028	0% (0.19)
VATE1_HUMAN	M0QXB4_HUMAN	COPE	M0QXB4	Coatomer protein complex, subunit epsilon, isoform CRA_g (Coatomer subunit epsilon)	37 kDa	0.21	15	6	8	0	1	2	2	2	7	0	0	1.428518579	0% (0.31)
A8K6I4_HUMAN (+1)	B4DE16_HUMAN	CTNNBL1	B4DE16	Beta-catenin-like protein 1 (cDNA FLJ53492, highly similar to Beta-catenin-like protein 1)	62 kDa	0.12	14	6	8	0	4	1	1	1	3	4	0	1.428518579	0% (0.31)
A1JUI8_HUMAN	TIM14_HUMAN	DNAJC19	Q96DA6	Mitochondrial import inner membrane translocase subunit TIM14 (DnaJ homolog subfamily C member 19)	12 kDa	0.22	14	6	8	1	1	1	2	2	2	2	2	1.428518579	0% (0.31)
PRS8_HUMAN	B3KM74_HUMAN		B3KM74	cDNA FLJ10425 fis, clone NT2RP1000326, highly similar to Metaxin-2	30 kDa	0.25	14	6	8	3	1	0	2	3	1	1	3	1.428518579	0% (0.31)
SPON1_HUMAN	D6R952_HUMAN	MSMO1	D6R952	Methylsterol monooxygenase 1	28 kDa	0.13	14	6	8	1	2	1	2	2	3	1	1	1.428518579	0% (0.31)
B3KNT8_HUMAN (+11)	A8K5M4_HUMAN		A8K5M4	cDNA FLJ75088, highly similar to Homo sapiens p21 (CDKN1A)-activated kinase 2 (PAK2), mRNA	58 kDa	0.14	39	17	22	0	4	8	5	5	12	4	1	1.426488739	0% (0.16)
ECHP_HUMAN	B4DPD5_HUMAN		B4DPD5	cDNA FLJ56307, highly similar to Ubiquitin thioesterase protein OTUB1 (EC 3.4.-.-)	35 kDa	0.23	39	17	22	0	5	2	10	8	7	4	3	1.426488739	0% (0.16)
Q7KZ24_HUMAN (+1)	H3BN98_HUMAN		H3BN98	Uncharacterized protein (Fragment)	27 kDa	0.2	48	21	27	4	6	4	7	8	12	4	4	1.421692323	0% (0.13)
B4DKB2_HUMAN (+1)	PSMD2_HUMAN	PSMD2	Q13200	26S proteasome non-ATPase regulatory subunit 2 (26S proteasome regulatory subunit RPN1) (26S proteasome regulatory subunit S2) (26S proteasome subunit p97) (Protein 55.11) (Tumor necrosis factor type 1 receptor-associated protein 2)	100 kDa	0.19	108	47	60	6	14	13	14	14	20	17	9	1.421631716	95% (0.038)
STML2_HUMAN	C9J9I1_HUMAN	SELI	C9J9I1	Selenoprotein I	44 kDa	0.21	30	10	13	2	0	6	2	6	2	3	2	1.418252076	0% (0.24)
B2R8A2_HUMAN (+1)	A0SBM6_HUMAN	ND1	A0SBM6	NADH-ubiquinone oxidoreductase chain 1 (EC 1.6.5.3)	36 kDa	0.22	23	10	13	6	1	2	2	4	4	3	2	1.418252076	0% (0.24)
DNJC3_HUMAN	CDK1_HUMAN	CDK1	P06493	Cyclin-dependent kinase 1 (CDK1) (EC 2.7.11.22) (EC 2.7.11.23) (Cell division control protein 2 homolog) (Cell division protein kinase 1) (p34 protein kinase)	34 kDa	0.18	22	10	13	0	4	4	2	3	4	5	1	1.418252076	0% (0.24)
SCRIB_HUMAN	B0I1T1_HUMAN		B0I1T1	MYO1F variant protein	125 kDa	0.057	66	29	37	6	12	9	2	3	6	8	19	1.415927738	0% (0.090)
MUTA_HUMAN	QCR2_HUMAN	UQCRC2	P22695	Cytochrome b-c1 complex subunit 2, mitochondrial (Complex III subunit 2) (Core protein II) (Ubiquinol-cytochrome-c reductase complex core protein 2)	48 kDa	0.23	194	86	109	32	16	19	19	32	16	27	34	1.41540321	95% (0.0083)
DDX46_HUMAN	B2R7P8_HUMAN		B2R7P8	cDNA, FLJ93545, highly similar to Homo sapiens 5-	65 kDa	0.1	32	14	18	0	6	4	4	5	10	3	0	1.413386361	0% (0.19)

				aminoimidazole-4-carboxamide ribonucleotide formyltransferase/IMP cyclohydrolase (ATIC), mRNA															
NPC1_HUMAN (+1)	J3QRU1_HUMAN	YES1	J3QRU1	Tyrosine-protein kinase Yes	61 kDa	0.07	31	14	18	3	4	5	2	7	4	0	4	1.413386361	0% (0.19)
A8K548_HUMAN (+3)	PO210_HUMAN	NUP210	Q8TEM1	Nuclear pore membrane glycoprotein 210 (Nuclear pore protein gp210) (Nuclear envelope pore membrane protein POM 210) (POM210) (Nucleoporin Nup210) (Pore membrane protein of 210 kDa)	205 kDa	0.069	84	37	47	14	8	8	7	17	14	8	9	1.41259856	0% (0.064)
DDX18_HUMAN	RCN1_HUMAN	RCN1	Q15293	Reticulocalbin-1	39 kDa	0.13	41	18	23	8	3	3	4	2	6	5	10	1.410554713	0% (0.16)
TMX1_HUMAN	PP2AA_HUMAN	PPP2CA	P67775	Serine/threonine-protein phosphatase 2A catalytic subunit alpha isoform (PP2A-alpha) (EC 3.1.3.16) (Replication protein C) (RP-C)	36 kDa	0.13	41	18	23	1	5	2	10	6	6	9	2	1.410554713	0% (0.16)
F5H6U7_HUMAN (+1)	A6QKW0_HUMAN	SHINC3	A6QKW0	SHINC3	28 kDa	0.27	50	22	28	6	4	5	8	3	5	8	12	1.408707293	0% (0.13)
B4DJQ8_HUMAN	B7Z5J7_HUMAN		B7Z5J7	cDNA FLJ58682, highly similar to Vesicle-fusing ATPase (EC 3.6.4.6)	82 kDa	0.13	151	67	84	16	12	24	15	18	15	25	27	1.398970221	95% (0.021)
B4E324_HUMAN (+2)	NNTM_HUMAN	NNT	Q13423	NAD(P) transhydrogenase, mitochondrial (EC 1.6.1.2) (Nicotinamide nucleotide transhydrogenase) (Pyridine nucleotide transhydrogenase)	114 kDa	0.1	97	43	54	17	8	9	9	12	13	17	12	1.398241392	0% (0.055)
ARC1B_HUMAN	B3KRS5_HUMAN	HDAC2	B3KRS5	Histone deacetylase (EC 3.5.1.98)	52 kDa	0.22	52	23	29	0	7	5	6	0	0	11	7	1.396793335	0% (0.13)
CISD2_HUMAN	FAF2_HUMAN	FAF2	Q96CS3	FAS-associated factor 2 (Protein ETEA) (UBX domain-containing protein 3B) (UBX domain-containing protein 8)	53 kDa	0.37	34	15	19	10	1	3	1	7	1	7	4	1.395315235	0% (0.20)
C9JV86_HUMAN (+4)	A8K6A5_HUMAN		A8K6A5	cDNA FLJ77742, highly similar to Homo sapiens integrin, alpha 5 (fibronectin receptor, alpha polypeptide), mRNA	114 kDa	0.085	25	11	14	0	6	5	0	0	1	6	7	1.393880392	0% (0.24)
BZW1_HUMAN (+1)	4F2_HUMAN	SLC3A2	P08195	4F2 cell-surface antigen heavy chain (4F2hc) (4F2 heavy chain antigen) (Lymphocyte activation antigen 4F2 large subunit) (Solute carrier family 3 member 2) (CD antigen CD98)	68 kDa	0.14	117	52	65	9	18	19	6	14	12	19	20	1.393310617	95% (0.040)
B9EGQ8_HUMAN (+2)	MUC16_HUMAN	MUC16	Q8WXI7	Mucin-16 (MUC-16) (Ovarian cancer-related tumor marker CA125) (CA-125) (Ovarian carcinoma antigen CA125)	2353 kDa	0.062	16	7	9	6	1	0	0	7	0	1	1	1.391082065	0% (0.31)
FLII_HUMAN	APOL2_HUMAN	APOL2	Q9BQE5	Apolipoprotein L2 (Apolipoprotein L-II) (ApoL-II)	37 kDa	0.084	17	7	9	5	0	0	2	3	1	1	4	1.391082065	0% (0.31)
B0QY89_HUMAN	E9PFA8_HUMAN	ERGIC3	E9PFA8	Endoplasmic reticulum-Golgi intermediate compartment protein 3	45 kDa	0.22	16	7	9	2	2	2	1	3	3	2	1	1.391082065	0% (0.31)
B4DGM9_HUMAN	BRE_HUMAN	BRE	Q9NXR7	BRCA1-A complex subunit BRE (BRCA1/BRCA2-containing complex subunit 45) (Brain and reproductive organ-expressed protein)	44 kDa	0.38	16	7	9	2	1	2	2	1	4	2	2	1.391082065	0% (0.31)
NDUV2_HUMAN	B2R4Q7_HUMAN		B2R4Q7	cDNA, FLJ92180, highly similar to Homo sapiens brain protein 44 (BRP44), mRNA	14 kDa	0.3	16	7	9	3	1	2	1	1	3	1	4	1.391082065	0% (0.31)
A4D0R2_HUMAN (+1)	GRB7_HUMAN	GRB7	Q14451	Growth factor receptor-bound protein 7 (B47) (Epidermal growth factor receptor GRB-7) (GRB7 adapter protein)	60 kDa	0.15	16	7	9	1	0	2	4	4	4	1	0	1.391082065	0% (0.31)
B2RBG3_HUMAN (+1)	NDUA4_HUMAN	NDUFA4	O00483	NADH dehydrogenase [ubiquinone] 1 alpha subcomplex subunit 4 (Complex I-MLRQ) (CI-MLRQ) (NADH-ubiquinone oxidoreductase MLRQ subunit)	9 kDa	0.085	16	7	9	5	0	0	2	4	2	2	1	1.391082065	0% (0.31)

H15_HUMAN	PHB_HUMAN	PHB	P35232	Prohibitin	30 kDa	0.3	259	116	144	39	19	16	42	28	54	27	34	1.38778357	95% (0.0044)
C163A_HUMAN	BAP31_HUMAN	BCAP31	P51572	B-cell receptor-associated protein 31 (BCR-associated protein 31) (Bap31) (6C6-AG tumor-associated antigen) (Protein CDM) (p28)	28 kDa	0.1	101	45	56	13	7	9	15	6	13	14	24	1.386207484	0% (0.056)
PTPRF_HUMAN	QCR1_HUMAN	UQCRC1	P31930	Cytochrome b-c1 complex subunit 1, mitochondrial (Complex III subunit 1) (Core protein I) (Ubiquinol-cytochrome-c reductase complex core protein 1)	53 kDa	0.077	139	62	77	24	14	12	13	24	11	21	22	1.385557954	95% (0.030)
G3V198_HUMAN (+1)	B9A067_HUMAN	IMMT	B9A067	Mitochondrial inner membrane protein	79 kDa	0.083	175	79	98	34	15	14	16	28	13	28	30	1.385232802	95% (0.016)
ARPC4_HUMAN (+1)	SYEP_HUMAN	EPRS	P07814	Bifunctional glutamate/proline--tRNA ligase (Bifunctional aminoacyl-tRNA synthetase) (Cell proliferation-inducing gene 32 protein) (Glutamatyl-prolyl-tRNA synthetase) [Includes: Glutamate--tRNA ligase (EC 6.1.1.17) (Glutamyl-tRNA synthetase) (GluRS); Proline--tRNA ligase (EC 6.1.1.15) (Prolyl-tRNA synthetase)]	171 kDa	0.35	82	37	46	0	8	14	14	17	14	10	5	1.383301995	0% (0.079)
F8VPD4_HUMAN (+1)	USP9X_HUMAN	USP9X	Q93008	Probable ubiquitin carboxyl-terminal hydrolase FAF-X (EC 3.4.19.12) (Deubiquitinating enzyme FAF-X) (Fat facets in mammals) (hFAM) (Fat facets protein-related, X-linked) (Ubiquitin thioesterase FAF-X) (Ubiquitin-specific protease 9, X chromosome) (Ubiquitin-specific-processing protease FAF-X)	292 kDa	0.097	45	20	25	3	9	5	4	1	17	7	0	1.383186167	0% (0.16)
B2RAK1_HUMAN (+1)	B3KNB4_HUMAN		B3KNB4	cDNA FLJ14168 fis, clone NT2RP2001440, highly similar to 14-3-3 protein gamma	28 kDa	0.11	45	20	25	5	5	5	5	9	5	5	6	1.383186167	0% (0.16)
PRS10_HUMAN	ELYS_HUMAN	AHCTF1	Q8WYP5	Protein ELYS (Embryonic large molecule derived from yolk sac) (Protein MEL-28) (Putative AT-hook-containing transcription factor 1)	253 kDa	0.27	7	3	4	1	1	0	1	3	0	1	0	1.38307041	0% (0.44)
ZNT7_HUMAN	B4DH53_HUMAN	MAP1S	B4DH53	MAP1S light chain (cDNA FLJ55328, highly similar to Homo sapiens BPY2 interacting protein 1 (BPY2IP1), mRNA)	110 kDa	0.12	7	3	4	0	0	0	3	0	4	0	0	1.38307041	0% (0.44)
PPAL_HUMAN	E7EX90_HUMAN	DCTN1	E7EX90	Dynactin subunit 1 (Uncharacterized protein)	139 kDa	0.2	7	3	4	0	0	2	1	0	1	3	0	1.38307041	0% (0.44)
PUR6_HUMAN	EDC4_HUMAN	EDC4	Q6P2E9	Enhancer of mRNA-decapping protein 4 (Autoantigen Ge-1) (Autoantigen RCD-8) (Human enhancer of decapping large subunit) (Hedls)	152 kDa	0.32	7	3	4	1	1	1	0	4	0	0	0	1.38307041	0% (0.44)
B3KXZ4_HUMAN	ODBB_HUMAN	BCKDHB	P21953	2-oxoisovalerate dehydrogenase subunit beta, mitochondrial (EC 1.2.4.4) (Branched-chain alpha-keto acid dehydrogenase E1 component beta chain) (BCKDE1B) (BCKDH E1-beta)	43 kDa	0.17	7	3	4	2	1	0	0	1	2	0	1	1.38307041	0% (0.44)
AK1A1_HUMAN	GTPBA_HUMAN	GTPBP10	A4D1E9	GTP-binding protein 10 (Protein obg homolog 2) (ObgH2)	43 kDa	0.29	7	3	4	3	0	0	0	0	0	2	1	1.38307041	0% (0.44)
HEXB_HUMAN	TMM68_HUMAN	TMEM68	Q96MH6	Transmembrane protein 68	37 kDa	0.17	7	3	4	3	0	0	0	2	0	0	2	1.38307041	0% (0.44)
SCPDL_HUMAN	NDUB3_HUMAN	NDUFB3	O43676	NADH dehydrogenase [ubiquinone] 1 beta subcomplex subunit 3 (Complex I-B12) (CI-B12) (NADH-ubiquinone oxidoreductase B12 subunit)	11 kDa	0.23	7	3	4	0	1	1	1	2	0	1	1	1.38307041	0% (0.44)

EST1_HUMAN	H9NTY4_HUMAN	CYTB	H9NTY4	Cytochrome b	43 kDa	0.27	9	3	4	1	2	0	0	1	1	1	1	1.38307041	0% (0.44)
CN166_HUMAN (+1)	MECR_HUMAN	MECR	Q9BV79	Trans-2-enoyl-CoA reductase, mitochondrial (EC 1.3.1.38) (Nuclear receptor-binding factor 1) (HsNrbf-1) (NRBF-1)	40 kDa	0.39	7	3	4	1	0	0	2	2	0	0	2	1.38307041	0% (0.44)
B3KSW0_HUMAN (+5)	C9JAF7_HUMAN	SNAP29	C9JAF7	Synaptosomal-associated protein (Fragment)	18 kDa	0.26	7	3	4	1	1	1	0	0	0	1	3	1.38307041	0% (0.44)
Q32Q62_HUMAN (+1)	F5GYJ5_HUMAN		F5GYJ5	Uncharacterized protein	18 kDa	0.14	7	3	4	0	1	2	0	3	0	0	1	1.38307041	0% (0.44)
RBBP4_HUMAN	H7C525_HUMAN	CCDC58	H7C525	Coiled-coil domain-containing protein 58 (Fragment)	16 kDa	0.15	7	3	4	0	1	2	0	2	0	2	0	1.38307041	0% (0.44)
AT1B1_HUMAN (+1)	NADC_HUMAN	QPRT	Q15274	Nicotinate-nucleotide pyrophosphorylase [carboxylating] (EC 2.4.2.19) (Quinolinate phosphoribosyltransferase [decarboxylating]) (QAPRTase) (QPRTase)	31 kDa	0.18	7	3	4	1	1	1	0	3	0	1	0	1.38307041	0% (0.44)
SF3A1_HUMAN	RL7L_HUMAN	RPL7L1	Q6DKI1	60S ribosomal protein L7-like 1	29 kDa	0.13	7	3	4	0	0	0	3	0	0	1	3	1.38307041	0% (0.44)
A8K274_HUMAN (+1)	FAAH1_HUMAN	FAAH	O00519	Fatty-acid amide hydrolase 1 (EC 3.5.1.99) (Anandamide amidohydrolase 1) (Oleamide hydrolase 1)	63 kDa	0.17	7	3	4	3	0	0	0	0	4	0	0	1.38307041	0% (0.44)
KCRU_HUMAN	ZNT1_HUMAN	SLC30A1	Q9Y6M5	Zinc transporter 1 (ZnT-1) (Solute carrier family 30 member 1)	55 kDa	0.17	7	3	4	1	1	1	0	1	0	2	1	1.38307041	0% (0.44)
Q6NUK7_HUMAN	CLYBL_HUMAN	CLYBL	Q8N0X4	Citrate lyase subunit beta-like protein, mitochondrial (Citrate lyase beta-like) (EC 4.1.-.-)	37 kDa	0.14	7	3	4	2	1	0	0	3	0	0	1	1.38307041	0% (0.44)
Q6NZ55_HUMAN (+1)	B4DN34_HUMAN	MRPL48	B4DN34	39S ribosomal protein L48, mitochondrial (cDNA FLJ60720, highly similar to Homo sapiens mitochondrial ribosomal protein L48 (MRPL48), transcript variant 1, mRNA)	22 kDa	0.19	7	3	4	1	1	1	0	2	1	0	1	1.38307041	0% (0.44)
B3KRY3_HUMAN (+1)	A8MUB1_HUMAN	TUBA4A	A8MUB1	Tubulin alpha-4A chain (Tubulin, alpha 1 (Testis specific), isoform CRA_a) (cDNA FLJ58687, highly similar to Tubulin alpha-4 chain)	48 kDa	0.14	390	174	215	48	43	44	39	53	69	48	47	1.382728791	95% (0.00071)
DYN2_HUMAN	UCRI_HUMAN	UQCRFS1	P47985	Cytochrome b-c1 complex subunit Rieske, mitochondrial (EC 1.10.2.2) (Complex III subunit 5) (Cytochrome b-c1 complex subunit 5) (Rieske iron-sulfur protein) (RISP) (Ubiquinol-cytochrome c reductase iron-sulfur subunit) [Cleaved into: Cytochrome b-c1 complex subunit 11 (Complex III subunit IX) (Ubiquinol-cytochrome c reductase 8 kDa protein)]	30 kDa	0.16	36	16	20	5	4	4	3	8	2	3	7	1.379339512	0% (0.20)
Q6DEN2_HUMAN	F6S0T5_HUMAN	KDM1A	F6S0T5	Lysine-specific histone demethylase 1A	95 kDa	0.26	36	16	20	2	6	7	1	5	0	12	3	1.379339512	0% (0.20)
Q8WVX7_HUMAN (+1)	RT09_HUMAN	MRPS9	P82933	28S ribosomal protein S9, mitochondrial (MRP-S9) (S9mt)	46 kDa	0.32	36	16	20	5	6	2	3	8	3	2	7	1.379339512	0% (0.20)
RS7_HUMAN	A6NNI4_HUMAN	CD9	A6NNI4	CD9 antigen	18 kDa	0.23	36	16	20	4	5	5	2	5	6	4	5	1.379339512	0% (0.20)
H3BS72_HUMAN (+1)	SAMH1_HUMAN	SAMHD1	Q9Y3Z3	Deoxynucleoside triphosphate triphosphohydrolase SAMHD1 (dNTPase) (EC 3.1.5.-) (Dendritic cell-derived IFNG-induced protein) (DCIP) (Monocyte protein 5) (MOP-5) (SAM domain and HD domain-containing protein 1)	72 kDa	0.16	65	29	36	0	8	11	10	3	16	12	5	1.378890174	0% (0.11)
G3V203_HUMAN (+2)	ACOX1_HUMAN	ACOX1	Q15067	Peroxisomal acyl-coenzyme A oxidase 1 (AOX) (EC 1.3.3.6) (Palmitoyl-CoA oxidase) (Straight-chain acyl-CoA oxidase) (SCOX)	74 kDa	0.35	114	51	63	10	8	10	23	2	32	19	11	1.377087673	95% (0.049)
NDUS8_HUMAN (+1)	AT2B1_HUMAN	ATP2B1	P20020	Plasma membrane calcium-transporting ATPase 1	139 kDa	0.15	56	25	31	8	6	4	7	10	0	8	0	1.375689004	0% (0.13)

				(PMCA1) (EC 3.6.3.8) (Plasma membrane calcium ATPase isoform 1) (Plasma membrane calcium pump isoform 1)													
J3KN00_HUMA N	DX39A_HUMA N	DDX39A	O00148	ATP-dependent RNA helicase DDX39A (EC 3.6.4.13) (DEAD box protein 39) (Nuclear RNA helicase URH49)	49 kDa	0.32	75	34	42	2	8	9	15	12	14	12	4	1.3739323 93	0% (0.094)
APT_HUMAN	B7Z3E3_HUMA N		B7Z3E3	Reticulon	101 kDa	0.34	27	12	15	5	2	2	3	4	3	3	5	1.3731877 13	0% (0.24)
A8K6K8_HUMA N (+1)	Q53HV2_HUMA N		Q53HV2	Chaperonin containing TCP1, subunit 7 (Eta) variant (Fragment)	59 kDa	0.19	48	21	26	1	6	5	9	9	11	5	1	1.3713471 97	0% (0.16)
B4DZF2_HUMA N (+4)	B2R8G6_HUMA N		B2R8G6	cDNA, FLJ93891, Homo sapiens FK506 binding protein 8, 38kDa (FKBP8), mRNA	38 kDa	0.094	47	21	26	4	10	3	4	6	6	9	5	1.3713471 97	0% (0.16)
PTGIS_HUMAN (+2)	HEAT1_HUMA N	HEATR1	Q9H583	HEAT repeat-containing protein 1 (Protein BAP28) [Cleaved into: HEAT repeat-containing protein 1, N-terminally processed]	242 kDa	0.28	67	30	37	5	6	1	18	16	3	11	7	1.3706004 34	0% (0.11)
DCXR_HUMAN	B3KPS3_HUMA N		B3KPS3	cDNA FLJ32131 fis, clone PEBLM2000267, highly similar to Tubulin alpha-ubiquitous chain	46 kDa	0.28	472	212	259	57	55	55	46	65	80	60	56	1.3677410 16	95% (0.0003 4)
SEPT9_HUMAN	LACTB_HUMA N	LACTB	P83111	Serine beta-lactamase-like protein LACTB, mitochondrial (EC 3.4.-.-)	61 kDa	0.19	57	26	32	15	5	3	3	1	3	9	19	1.3662987 35	0% (0.13)
RECQ1_HUMA N	MDHM_HUMA N	MDH2	P40926	Malate dehydrogenase, mitochondrial (EC 1.1.1.37)	36 kDa	0.19	476	214	261	43	56	49	67	109	55	46	52	1.3654501 84	95% (0.0003 4)
D3DR31_HUMA N (+1)	PUR6_HUMAN	PAICS	P22234	Multifunctional protein ADE2 [Includes: Phosphoribosylaminoimidazol e-succinocarboxamide synthase (EC 6.3.2.6) (SAICAR synthetase); Phosphoribosylaminoimidazol e carboxylase (EC 4.1.1.21) (AIR carboxylase) (AIRC)]	47 kDa	0.3	39	17	21	0	6	6	5	11	5	5	1	1.3651147 42	0% (0.20)
RRP5_HUMAN	F162A_HUMAN	FAM162A	Q96A26	Protein FAM162A (E2-induced gene 5 protein) (Growth and transformation-dependent protein) (HGTD-P)	17 kDa	0.051	36	17	21	2	4	3	7	6	3	2	9	1.3651147 42	0% (0.20)
Q0VAB1_HUM AN	B4DE36_HUMA N		B4DE36	Glucose-6-phosphate isomerase (EC 5.3.1.9)	60 kDa	0.12	171	77	94	8	20	24	26	23	28	22	22	1.3633396 91	95% (0.023)
D6RER5_HUMA N (+2)	B7Z4S9_HUMA N	BUD31	B7Z4S9	BUD31 homolog (Yeast), isoform CRA_b (cDNA FLJ53814, highly similar to Protein G10 homolog) (cDNA, FLJ79376, highly similar to Protein G10 homolog)	17 kDa	0.17	18	8	10	2	2	2	2	5	0	4	2	1.3617403 05	0% (0.31)
B4DE59_HUMA N	SBP1_HUMAN	SELENBP1	Q13228	Selenium-binding protein 1 (56 kDa selenium-binding protein) (SBP56) (SP56)	52 kDa	0.44	18	8	10	0	1	1	6	2	4	4	0	1.3617403 05	0% (0.31)
ELOV1_HUMA N (+1)	B5MCA4_HUM AN	EPCAM	B5MCA 4	Epithelial cell adhesion molecule	38 kDa	0.079	49	22	27	6	5	4	7	10	4	8	5	1.3605267 81	0% (0.16)
B5MCX3_HUM AN (+1)	B3KX72_HUMA N		B3KX72	cDNA FLJ44920 fis, clone BRAMY3011501, highly similar to Heterogeneous nuclear ribonucleoprotein U	83 kDa	0.3	235	107	130	22	33	25	28	39	23	40	28	1.3581649 43	95% (0.0098)
G5E972_HUMA N (+1)	B2RAF9_HUMA N		B2RAF9	cDNA, FLJ94888, highly similar to Homo sapiens suppression of tumorigenicity 14 (colon carcinoma, matriptase, epithin) (ST14), mRNA	95 kDa	0.24	28	13	16	2	4	5	2	5	0	5	6	1.3553995 11	0% (0.25)
RTCB_HUMAN	EMC1_HUMAN	EMC1	Q8N766	ER membrane protein complex subunit 1	112 kDa	0.16	82	37	45	15	6	10	8	8	11	10	16	1.3540062 72	0% (0.096)
B4E175_HUMA N	PRDX6_HUMA N	PRDX6	P30041	Peroxiredoxin-6 (EC 1.11.1.15) (1-Cys peroxiredoxin) (1-Cys PRX) (24 kDa protein) (Acidic calcium-independent phospholipase A2) (aiPLA2) (EC 3.1.1.-) (Antioxidant protein 2) (Liver 2D page spot	25 kDa	0.2	93	42	51	5	9	9	23	14	17	14	6	1.3528515 93	0% (0.081)

				40) (Non-selenium glutathione peroxidase) (NSGPx) (EC 1.11.1.9) (Red blood cells page spot 12)															
G3V5Z7_HUMAN (+1)	B3KQF5_HUMAN		B3KQF5	cDNA FLJ90381 fis, clone NT2RP2005035, highly similar to Calumenin	37 kDa	0.33	39	18	22	6	6	4	2	2	2	8	10	1.352368049	0% (0.20)
MDHC_HUMAN	SF3B3_HUMAN	SF3B3	Q15393	Splicing factor 3B subunit 3 (Pre-mRNA-splicing factor SF3b 130 kDa subunit) (SF3b130) (STAF130) (Spliceosome-associated protein 130) (SAP 130)	136 kDa	0.29	169	77	93	18	24	23	12	22	13	31	26	1.349006982	95% (0.028)
HP1B3_HUMAN	D6R9P3_HUMAN	HNRNPAB	D6R9P3	Heterogeneous nuclear ribonucleoprotein A/B	30 kDa	0.13	82	38	46	5	14	11	7	18	5	16	7	1.348041304	0% (0.096)
KCRB_HUMAN	STAT1_HUMAN	STAT1	P42224	Signal transducer and activator of transcription 1-alpha/beta (Transcription factor ISGF-3 components p91/p84)	87 kDa	0.29	138	63	76	1	14	18	31	5	42	16	13	1.346524927	95% (0.044)
B4E1R7_HUMAN (+1)	A8K525_HUMAN		A8K525	cDNA FLJ76817, highly similar to Homo sapiens non-POU domain containing, octamer-binding (NONO), mRNA	54 kDa	0.2	161	73	88	23	16	14	20	21	14	33	22	1.34623233	95% (0.032)
EBP_HUMAN	D9HTE9_HUMAN	SLC25A1	D9HTE9	Plasma membrane citrate carrier	35 kDa	0.17	86	39	47	15	9	6	10	8	18	9	13	1.342372817	0% (0.097)
B3KR70_HUMAN	CBX1_HUMAN	CBX1	P83916	Chromobox protein homolog 1 (HP1Hsbeta) (Heterochromatin protein 1 homolog beta) (HP1 beta) (Heterochromatin protein p25) (M31) (Modifier 1 protein) (p25beta)	21 kDa	0.22	54	24	29	5	0	0	6	6	6	12	0	1.341457547	0% (0.16)
B4DRA5_HUMAN	LKHA4_HUMAN	LTA4H	P09960	Leukotriene A-4 hydrolase (LTA-4 hydrolase) (EC 3.3.2.6) (Leukotriene A(4) hydrolase)	69 kDa	0.14	42	19	23	5	3	8	3	7	1	11	4	1.340880459	0% (0.20)
B0QZ18_HUMAN (+2)	I3L397_HUMAN	EIF5A	I3L397	Eukaryotic translation initiation factor 5A-1 (Fragment)	16 kDa	0.079	31	14	17	2	3	3	6	5	6	2	3	1.339944414	0% (0.25)
A4D210_HUMAN (+2)	F2Z2U8_HUMAN	MYH14	F2Z2U8	Myosin-14	231 kDa	0.1	142	65	78	25	8	8	25	13	26	19	21	1.339688314	95% (0.044)
BCAM_HUMAN (+3)	NUP37_HUMAN	NUP37	Q8NFH4	Nucleoporin Nup37 (p37) (Nup107-160 subcomplex subunit Nup37)	37 kDa	0.22	20	9	11	0	3	4	2	4	0	5	3	1.338124102	0% (0.31)
GALT2_HUMAN	A8K6K7_HUMAN		A8K6K7	cDNA FLJ76881, highly similar to Homo sapiens glycogen synthase 1 (muscle) (GYS1), mRNA	84 kDa	0.19	20	9	11	2	2	4	2	2	8	1	0	1.338124102	0% (0.31)
Q6LEU0_HUMAN (+1)	A6NFX8_HUMAN	NUDT5	A6NFX8	ADP-sugar pyrophosphatase	26 kDa	0.29	20	9	11	0	2	2	6	4	4	3	0	1.338124102	0% (0.31)
B3KSI3_HUMAN (+3)	Q5SYZ4_HUMAN	CRABP2	Q5SYZ4	Cellular retinoic acid-binding protein 2 (Fragment)	9 kDa	0.27	20	9	11	0	2	3	4	2	5	3	1	1.338124102	0% (0.31)
RRAS_HUMAN	H0YN88_HUMAN	RPS17L	H0YN88	40S ribosomal protein S17 (40S ribosomal protein S17-like)	16 kDa	0.34	20	9	11	0	3	2	4	3	6	1	1	1.338124102	0% (0.31)
A8K6V7_HUMAN	B1AKZ3_HUMAN	PEA15	B1AKZ3	Astrocytic phosphoprotein PEA-15 (Phosphoprotein enriched in astrocytes 15, isoform CRA_b) (cDNA FLJ38560 fis, clone HCHON2003642, highly similar to Astrocytic phosphoprotein PEA-15)	17 kDa	0.096	19	9	11	0	3	3	3	4	3	3	1	1.338124102	0% (0.31)
E9PAV3_HUMAN	EF2_HUMAN	EEF2	P13639	Elongation factor 2 (EF-2)	95 kDa	0.035	191	87	104	17	21	20	29	22	38	28	16	1.335787991	95% (0.024)
A8K5M4_HUMAN (+1)	Q6FIG4_HUMAN	RAB1B	Q6FIG4	RAB1B protein	22 kDa	0.19	244	109	130	31	24	32	23	22	39	30	41	1.333492717	95% (0.014)
NEUA_HUMAN (+1)	MAGD2_HUMAN	MAGED2	Q9UNF1	Melanoma-associated antigen D2 (11B6) (Breast cancer-associated gene 1 protein) (BCG-1) (Hepatocellular carcinoma-associated protein JCL-1) (MAGE-D2 antigen)	65 kDa	0.14	9	4	5	0	0	2	2	0	4	1	0	1.332893033	0% (0.43)
AHNK2_HUMAN	SRRT_HUMAN	SRRT	Q9BXP5	Serrate RNA effector molecule homolog (Arsenite-resistance protein 2)	101 kDa	0.032	9	4	5	0	1	2	1	1	0	2	2	1.332893033	0% (0.43)
EMC3_HUMAN	RM17_HUMAN	MRPL17	Q9NRX2	39S ribosomal protein L17, mitochondrial (L17mt) (MRP-	20 kDa	0.35	10	4	5	1	1	2	0	1	0	3	1	1.332893033	0% (0.43)

				L17) (LYST-interacting protein 2)															
PABP2_HUMAN	B7Z700_HUMAN		B7Z700	cDNA FLJ54557, highly similar to helicase MOV-10 (EC 3.6.1.-)	107 kDa	0.13	9	4	5	1	0	0	3	4	0	1	0	1.332893033	0% (0.43)
OFUT1_HUMAN	NPL4_HUMAN	NPLOC4	Q8TAT6	Nuclear protein localization protein 4 homolog (Protein NPL4)	68 kDa	0.11	9	4	5	0	3	1	0	0	5	0	0	1.332893033	0% (0.43)
B4DKM6_HUMAN (+1)	S15A3_HUMAN	SLC15A3	Q8IY34	Solute carrier family 15 member 3 (Osteoclast transporter) (Peptide transporter 3) (Peptide/histidine transporter 2)	64 kDa	0.14	9	4	5	0	2	1	1	0	0	1	4	1.332893033	0% (0.43)
E7ESI6_HUMAN	A1L3A9_HUMAN	TBC1D9B	A1L3A9	TBC1 domain family, member 9B (With GRAM domain)	139 kDa	0.13	9	4	5	0	3	0	1	1	3	1	0	1.332893033	0% (0.43)
CPSF1_HUMAN (+1)	EI2BG_HUMAN	EIF2B3	Q9NR50	Translation initiation factor eIF-2B subunit gamma (eIF-2B GDP-GTP exchange factor subunit gamma)	50 kDa	0.09	9	4	5	0	1	3	0	3	2	0	0	1.332893033	0% (0.43)
B2R7P8_HUMAN (+1)	ABCF3_HUMAN	ABCF3	Q9NUQ8	ATP-binding cassette sub-family F member 3	80 kDa	0.2	9	4	5	0	2	0	2	3	0	2	0	1.332893033	0% (0.43)
H3BV16_HUMAN (+2)	F5H013_HUMAN	SNRPG	F5H013	Small nuclear ribonucleoprotein G	8 kDa	0.29	10	4	5	1	0	2	1	0	1	3	1	1.332893033	0% (0.43)
LKHA4_HUMAN	Q5JWT2_HUMAN	MED23	Q5JWT2	Mediator of RNA polymerase II transcription subunit 23	157 kDa	0.2	9	4	5	1	0	1	2	1	0	4	0	1.332893033	0% (0.43)
PSB3_HUMAN	B3KRM8_HUMAN	TSN	B3KRM8	Translin, isoform CRA_b (cDNA FLJ34596 fis, clone KIDNE2009191, highly similar to TRANSLIN)	21 kDa	0.34	9	4	5	0	0	0	3	2	3	0	0	1.332893033	0% (0.43)
THOC2_HUMAN	B7Z5W1_HUMAN		B7Z5W1	cDNA FLJ54854, highly similar to Junctional adhesion molecule A	33 kDa	0.061	9	4	5	1	1	1	1	1	1	2	1	1.332893033	0% (0.43)
TXD12_HUMAN	E9PS41_HUMAN	WDR74	E9PS41	WD repeat-containing protein 74	36 kDa	0.44	9	4	5	0	3	0	1	2	0	3	0	1.332893033	0% (0.43)
ATP5L_HUMAN (+1)	HSP74_HUMAN	HSPA4	P34932	Heat shock 70 kDa protein 4 (HSP70RY) (Heat shock 70-related protein APG-2)	94 kDa	0.36	125	57	68	3	23	14	17	21	13	25	9	1.331362637	0% (0.060)
FAHD1_HUMAN	FLII_HUMAN	FLII	Q13045	Protein flightless-1 homolog	145 kDa	0.28	45	20	24	6	5	4	5	9	5	7	4	1.330474216	0% (0.20)
A1KYQ7_HUMAN (+2)	PTPRF_HUMAN	PTPRF	P10586	Receptor-type tyrosine-protein phosphatase F (EC 3.1.3.48) (Leukocyte common antigen related) (LAR)	213 kDa	0.091	43	20	24	5	7	5	5	10	0	13	1	1.330474216	0% (0.20)
NSMA3_HUMAN	H15_HUMAN	HIST1H1B	P16401	Histone H1.5 (Histone H1a) (Histone H1b) (Histone H1s-3)	23 kDa	0.12	68	31	37	2	8	9	13	5	11	7	14	1.328084126	0% (0.14)
NCKP1_HUMAN	CPNS1_HUMAN	CAPNS1	P04632	Calpain small subunit 1 (CSS1) (Calcium-activated neutral proteinase small subunit) (CANP small subunit) (Calcium-dependent protease small subunit) (CDPS) (Calcium-dependent protease small subunit 1) (Calpain regulatory subunit)	28 kDa	0.087	33	15	18	4	3	3	5	4	6	3	5	1.326391695	0% (0.25)
TBB6_HUMAN	AUP1_HUMAN	AUP1	Q9Y679	Ancient ubiquitous protein 1	53 kDa	0.37	33	15	18	8	1	2	5	0	2	7	9	1.326391695	0% (0.25)
H2AW_HUMAN	ISOC2_HUMAN	ISOC2	Q96AB3	Isochorismatase domain-containing protein 2, mitochondrial	22 kDa	0.31	33	15	18	3	4	2	6	4	5	4	5	1.326391695	0% (0.25)
Q53GD7_HUMAN (+2)	CECR5_HUMAN	CECR5	Q9BXW7	Cat eye syndrome critical region protein 5	46 kDa	0.23	33	15	18	3	4	5	3	9	2	4	3	1.326391695	0% (0.25)
CP2S1_HUMAN	TBB6_HUMAN	TUBB6	Q9BUF5	Tubulin beta-6 chain (Tubulin beta class V)	50 kDa	0.19	309	142	168	48	32	34	29	42	43	36	46	1.323602115	95% (0.0070)
B2R8G6_HUMAN	Q6NUK7_HUMAN	LYN	Q6NUK7	LYN protein (Fragment)	66 kDa	0.17	44	21	25	4	6	8	3	5	4	11	5	1.32100352	0% (0.20)
B1AK13_HUMAN (+1)	KAT3_HUMAN	CCBL2	Q6YP21	Kynurenine--oxoglutarate transaminase 3 (EC 2.6.1.7) (Cysteine-S-conjugate beta-lyase 2) (EC 4.4.1.13) (Kynurenine aminotransferase III) (KATIII) (Kynurenine--glyoxylate transaminase) (EC 2.6.1.63) (Kynurenine--oxoglutarate transaminase III)	51 kDa	0.22	22	10	12	4	1	4	2	8	0	1	3	1.318706642	0% (0.31)
NIPS2_HUMAN	E7ETK0_HUMAN	RPS24	E7ETK0	40S ribosomal protein S24	15 kDa	0.17	59	27	32	8	6	3	10	8	14	5	5	1.317917239	0% (0.17)

SMHD1_HUMAN	1433T_HUMAN	YWHAQ	P27348	14-3-3 protein theta (14-3-3 protein T-cell) (14-3-3 protein tau) (Protein HS1)	28 kDa	0.071	59	27	32	5	6	7	9	11	6	8	7	1.317917239	0% (0.17)
B2RBA6_HUMAN (+1)	B4DZC0_HUMAN		B4DZC0	cDNA FLJ51771, highly similar to SWI/SNF-related matrix-associatedactin-dependent regulator of chromatin subfamily A member5 (EC 3.6.1.-)	117 kDa	0.23	97	44	52	11	10	12	12	30	3	11	9	1.31778964	0% (0.098)
A8KA84_HUMAN (+1)	B7Z532_HUMAN		B7Z532	cDNA FLJ51028, highly similar to 60 kDa heat shock protein, mitochondrial	27 kDa	0.16	360	166	195	38	50	46	32	48	38	54	56	1.314624976	95% (0.0048)
RCN1_HUMAN	ILVBL_HUMAN	ILVBL	A1L0T0	Acetolactate synthase-like protein (EC 2.2.1.-) (IlvB-like protein)	68 kDa	0.33	99	45	53	23	5	3	14	11	15	14	13	1.313511062	0% (0.099)
F8WCY5_HUMAN (+1)	G3V198_HUMAN	NUP160	G3V198	Nuclear pore complex protein Nup160 (Nucleoporin 160kDa, isoform CRA_e)	140 kDa	0.18	48	22	26	3	7	5	7	5	5	7	9	1.312347656	0% (0.20)
RM44_HUMAN	NU205_HUMAN	NUP205	Q92621	Nuclear pore complex protein Nup205 (205 kDa nucleoporin) (Nucleoporin Nup205)	228 kDa	0.2	137	63	74	14	15	17	18	15	8	35	17	1.311625665	0% (0.062)
B3KQF5_HUMAN (+1)	A7E2D8_HUMAN	ATP2B4	A7E2D8	ATP2B4 protein	129 kDa	0.3	61	28	33	14	4	2	8	11	5	6	11	1.311143768	0% (0.17)
DERL1_HUMAN	RAB6B_HUMAN	RAB6B	Q9NRW1	Ras-related protein Rab-6B	23 kDa	0.18	74	34	40	11	7	6	10	11	14	6	9	1.310360226	0% (0.14)
GSLG1_HUMAN	B4DGP8_HUMAN	CANX	B4DGP8	Calnexin (cDNA FLJ55574, highly similar to Calnexin)	72 kDa	0.07	359	165	193	47	38	39	40	45	52	43	53	1.309033546	95% (0.0055)
CTL2_HUMAN	TBB4A_HUMAN	TUBB4A	P04350	Tubulin beta-4A chain (Tubulin 5 beta) (Tubulin beta-4 chain)	50 kDa	0.12	496	229	267	65	49	56	60	71	80	63	55	1.305561414	95% (0.0015)
SPTN2_HUMAN	ELAV1_HUMAN	ELAVL1	Q15717	ELAV-like protein 1 (Hu-antigen R) (HuR)	36 kDa	0.067	63	29	34	3	9	13	4	10	6	12	6	1.304818244	0% (0.17)
B7Z7G9_HUMAN	TMED7_HUMAN	TMED7	Q9Y3B3	Transmembrane emp24 domain-containing protein 7 (p24 family protein gamma-3) (p24gamma3) (p27)	25 kDa	0.14	50	23	27	7	5	5	6	6	9	7	5	1.30440582	0% (0.20)
A8K3M9_HUMAN (+1)	PRS8_HUMAN	PSMC5	P62195	26S protease regulatory subunit 8 (26S proteasome AAA-ATPase subunit RPT6) (Proteasome 26S subunit ATPase 5) (Proteasome subunit p45) (Thyroid hormone receptor-interacting protein 1) (TRIP1) (p45/SUG)	46 kDa	0.29	37	17	20	1	5	5	6	2	12	4	3	1.303742511	0% (0.25)
B3KTT0_HUMAN (+1)	B1AK13_HUMAN	HMGCL	B1AK13	3-hydroxymethyl-3-methylglutaryl-Coenzyme A lyase (Hydroxymethylglutaricaciduria), isoform CRA_b (Hydroxymethylglutaryl-CoA lyase, mitochondrial) (cDNA FLJ16378 fis, clone TKIDN2016399, highly similar to Hydroxymethylglutaryl-CoA lyase, mitochondrial (EC 4.1.3.4))	32 kDa	0.19	37	17	20	4	2	3	9	9	3	3	5	1.303742511	0% (0.25)
Q59GK9_HUMAN	SMHD1_HUMAN	SMCHD1	A6NHR9	Structural maintenance of chromosomes flexible hinge domain-containing protein 1 (SMC hinge domain-containing protein 1)	226 kDa	0.16	37	17	20	0	7	9	2	5	5	10	0	1.303742511	0% (0.25)
DNJB1_HUMAN (+1)	G5E9V5_HUMAN	MRPS22	G5E9V5	28S ribosomal protein S22, mitochondrial (Mitochondrial ribosomal protein S22, isoform CRA_a)	41 kDa	0.23	37	17	20	6	4	3	4	8	2	3	7	1.303742511	0% (0.25)
SSRD_HUMAN	PGRC2_HUMAN	PGRMC2	O15173	Membrane-associated progesterone receptor component 2 (Progesterone membrane-binding protein) (Steroid receptor protein DG6)	24 kDa	0.27	37	17	20	3	4	5	5	4	6	4	6	1.303742511	0% (0.25)
B2RDW0_HUMAN (+2)	Q860C0_HUMAN	HLA-DRB1	Q860C0	MHC class II antigen	30 kDa	0.48	37	17	20	1	3	3	9	2	4	2	12	1.303742511	0% (0.25)
G5E9V5_HUMAN	MYO1E_HUMAN	MYO1E	Q12965	Unconventional myosin-Ie (Myosin-Ic) (Unconventional myosin 1E)	127 kDa	0.3	37	17	20	6	5	4	2	5	0	6	7	1.303742511	0% (0.25)

MTA2_HUMAN	MCCA_HUMAN	MCCC1	Q96RQ3	Methylcrotonoyl-CoA carboxylase subunit alpha, mitochondrial (MCCase subunit alpha) (EC 6.4.1.4) (3-methylcrotonyl-CoA carboxylase 1) (3-methylcrotonyl-CoA carboxylase biotin-containing subunit) (3-methylcrotonyl-CoA:carbon dioxide ligase subunit alpha)	80 kDa	0.13	24	11	13	6	1	3	1	7	0	3	3	1.30245966	0% (0.31)
B4DRV2_HUMAN (+4)	ALG3_HUMAN	ALG3	Q92685	Dol-P-Man:Man(5)GlcNAc(2)-PP-Dol alpha-1,3-mannosyltransferase (EC 2.4.1.258) (Asparagine-linked glycosylation protein 3 homolog) (Dol-P-Man-dependent alpha(1-3)-mannosyltransferase) (Dolichyl-P-Man:Man(5)GlcNAc(2)-PP-dolichyl mannosyltransferase) (Dolichyl-phosphate-mannose--glycolipid alpha-mannosyltransferase) (Not56-like protein)	50 kDa	0.2	23	11	13	3	2	3	3	3	2	3	4	1.30245966	0% (0.31)
B4DYU3_HUMAN	P3H1_HUMAN	LEPRE1	Q32P28	Prolyl 3-hydroxylase 1 (EC 1.14.11.7) (Growth suppressor 1) (Leucine- and proline-enriched proteoglycan 1) (Leprecan-1)	83 kDa	0.19	25	11	13	1	6	3	1	2	6	3	2	1.30245966	0% (0.31)
A8K4T6_HUMAN (+1)	DHB11_HUMAN	HSD17B11	Q8NBQ5	Estradiol 17-beta-dehydrogenase 11 (EC 1.1.1.62) (17-beta-hydroxysteroid dehydrogenase 11) (17-beta-HSD 11) (17bHSD11) (17betaHSD11) (17-beta-hydroxysteroid dehydrogenase XI) (17-beta-HSD XI) (17betaHSDXI) (Cutaneous T-cell lymphoma-associated antigen HD-CL-03) (CTCL-associated antigen HD-CL-03) (Dehydrogenase/reductase SDR family member 8) (Retinal short-chain dehydrogenase/reductase 2) (retSDR2)	33 kDa	0.24	90	42	49	11	9	6	15	0	20	16	14	1.301030343	0% (0.12)
HMGB1_HUMAN	PDC6I_HUMAN	PDCD6IP	Q8WUM4	Programmed cell death 6-interacting protein (PDCD6-interacting protein) (ALG-2-interacting protein 1) (ALG-2-interacting protein X) (Hp95)	96 kDa	0.23	79	36	42	9	12	8	7	15	8	13	6	1.300130787	0% (0.14)
TPBG_HUMAN	Q59G75_HUMAN		Q59G75	Isoleucyl-tRNA synthetase, cytoplasmic variant (Fragment)	146 kDa	0.16	65	30	35	2	10	12	5	13	14	6	2	1.298897664	0% (0.17)
ERGI1_HUMAN	RAB3D_HUMAN	RAB3D	O95716	Ras-related protein Rab-3D	24 kDa	0.17	65	30	35	14	5	4	7	9	10	5	11	1.298897664	0% (0.17)
B2R6U8_HUMAN (+1)	NBAS_HUMAN	NBAS	A2RRP1	Neuroblastoma-amplified sequence (Neuroblastoma-amplified gene protein)	269 kDa	0.43	11	5	6	1	1	2	1	2	0	2	2	1.298772968	0% (0.42)
UCRI_HUMAN	RFC4_HUMAN	RFC4	P35249	Replication factor C subunit 4 (Activator 1 37 kDa subunit) (A1 37 kDa subunit) (Activator 1 subunit 4) (Replication factor C 37 kDa subunit) (RF-C 37 kDa subunit) (RFC37)	40 kDa	0.14	11	5	6	0	1	2	2	4	1	1	0	1.298772968	0% (0.42)
A8YXX5_HUMAN	S27A3_HUMAN	SLC27A3	Q5K4L6	Long-chain fatty acid transport protein 3 (FATP-3) (Fatty acid transport protein 3) (EC 6.2.1.-) (Solute carrier family 27 member 3) (Very long-chain acyl-CoA synthetase homolog 3) (VLCS-3)	79 kDa	0.16	10	5	6	0	2	2	1	0	2	1	3	1.298772968	0% (0.42)
B5BU25_HUMAN (+2)	E9PKP7_HUMAN	UBTF	E9PKP7	Nucleolar transcription factor 1	87 kDa	0.14	11	5	6	1	1	3	0	6	0	0	0	1.298772968	0% (0.42)

PBIP1_HUMAN	FMNL_HUMAN	FMNL1	O95466	Formin-like protein 1 (CLL-associated antigen KW-13) (Leukocyte formin)	122 kDa	0.13	11	5	6	0	2	0	3	0	3	2	1	1.2987729 68	0% (0.42)
F6S0T5_HUMA N	GUAA_HUMAN	GMPS	P49915	GMP synthase [glutamine-hydrolyzing] (EC 6.3.5.2) (GMP synthetase) (Glutamine amidotransferase)	77 kDa	0.16	11	5	6	0	1	1	3	1	3	2	0	1.2987729 68	0% (0.42)
DKC1_HUMAN	B2RE59_HUMA N		B2RE59	cDNA, FLJ93148, highly similar to Homo sapiens RCD1 required for cell differentiation1 homolog (S. pombe) (RQCD1), mRNA	34 kDa	0.086	11	5	6	1	2	1	1	1	3	1	1	1.2987729 68	0% (0.42)
DDRGK_HUMA N	B4DK32_HUMA N		B4DK32	cDNA FLJ61095, highly similar to Ubiquitin conjugation factor E4 A (Fragment)	120 kDa	0.21	11	5	6	0	4	1	0	1	4	1	0	1.2987729 68	0% (0.42)
B3KSH1_HUMA N (+2)	ATPF2_HUMAN	ATPAF2	Q8N5M1	ATP synthase mitochondrial F1 complex assembly factor 2 (ATP12 homolog)	33 kDa	0.21	11	5	6	2	2	0	1	4	0	0	2	1.2987729 68	0% (0.42)
ALG1_HUMAN	TM245_HUMAN	TMEM245	Q9H330	Transmembrane protein 245 (Protein CG-2)	101 kDa	0.11	11	5	6	1	2	2	0	2	1	1	2	1.2987729 68	0% (0.42)
H0YKW5_HUM AN	B7Z5X7_HUMA N	WARS2	B7Z5X7	Tryptophan--tRNA ligase, mitochondrial (cDNA FLJ50844, highly similar to Tryptophanyl-tRNA synthetase, mitochondrial (EC 6.1.1.2))	30 kDa	0.19	11	5	6	0	1	1	3	0	3	0	3	1.2987729 68	0% (0.42)
A8K529_HUMA N (+1)	B1AJY5_HUMA N	PSMD10	B1AJY5	26S proteasome non-ATPase regulatory subunit 10	20 kDa	0.12	10	5	6	0	3	1	1	1	4	1	0	1.2987729 68	0% (0.42)
B3KMR5_HUM AN (+1)	IF6_HUMAN	EIF6	P56537	Eukaryotic translation initiation factor 6 (eIF-6) (B(2)GCN homolog) (B4 integrin interactor) (CAB) (p27(BBP))	27 kDa	0.066	11	5	6	0	2	1	2	4	0	1	1	1.2987729 68	0% (0.42)
MA2A1_HUMA N	QCR9_HUMAN	UQCR10	Q9UDW 1	Cytochrome b-c1 complex subunit 9 (Complex III subunit 9) (Complex III subunit X) (Cytochrome c1 non-heme 7 kDa protein) (Ubiquinol-cytochrome c reductase complex 7.2 kDa protein)	7 kDa	0.1	11	5	6	1	1	1	2	2	1	1	2	1.2987729 68	0% (0.42)
RMD3_HUMAN	RB22A_HUMA N	RAB22A	Q9UL26	Ras-related protein Rab-22A (Rab-22)	22 kDa	0.24	11	5	6	3	0	0	0	0	0	2	0	1.2987729 68	0% (0.42)
AP2A1_HUMAN	P5CS_HUMAN	ALDH18A1	P54886	Delta-1-pyrroline-5-carboxylate synthase (P5CS) (Aldehyde dehydrogenase family 18 member A1) [Includes: Glutamate 5-kinase (GK) (EC 2.7.2.11) (Gamma-glutamyl kinase); Gamma-glutamyl phosphate reductase (GPR) (EC 1.2.1.41) (Glutamate-5-semialdehyde dehydrogenase) (Glutamyl-gamma-semialdehyde dehydrogenase)]	87 kDa	0.18	106	49	57	15	15	10	8	20	17	8	13	1.2981003 55	0% (0.099)
MIRO2_HUMA N	Q53GC2_HUMA N		Q53GC2	RAB4A, member RAS oncogene family variant (Fragment)	24 kDa	0.14	52	24	28	7	6	0	0	6	6	7	9	1.2970931 77	0% (0.20)
A0JLQ5_HUMA N (+1)	LETM1_HUMA N	LETM1	O95202	LETM1 and EF-hand domain-containing protein 1, mitochondrial (Leucine zipper-EF-hand-containing transmembrane protein 1)	83 kDa	0.11	107	50	58	16	10	16	10	17	6	17	18	1.2946237 18	0% (0.100)
PRS6B_HUMAN	H3BS72_HUMA N	PTPLAD1	H3BS72	Very-long-chain (3R)-3-hydroxyacyl-[acyl-carrier protein] dehydratase 3	47 kDa	0.12	39	18	21	9	3	3	3	3	9	6	3	1.2941830 59	0% (0.25)
CPNS1_HUMAN	CH10_HUMAN	HSPE1	P61604	10 kDa heat shock protein, mitochondrial (Hsp10) (10 kDa chaperonin) (Chaperonin 10) (CPN10) (Early-pregnancy factor) (EPF)	11 kDa	0.18	66	31	36	5	9	10	7	10	9	8	9	1.2933443 58	0% (0.17)
PRP6_HUMAN	NOP58_HUMAN	NOP58	Q9Y2X3	Nucleolar protein 58 (Nucleolar protein 5)	60 kDa	0.073	125	58	67	9	12	17	21	16	17	16	19	1.2899562 49	0% (0.085)
A8K2T7_HUMA N (+4)	RT35_HUMAN	MRPS35	P82673	28S ribosomal protein S35, mitochondrial (MRP-S35) (S35mt) (28S ribosomal protein S28, mitochondrial) (MRP-S28) (S28mt)	37 kDa	0.098	25	12	14	5	2	2	3	5	2	2	5	1.2886653 13	0% (0.31)

G3V2S9_HUMAN	RINI_HUMAN	RNH1	P13489	Ribonuclease inhibitor (Placental ribonuclease inhibitor) (Placental RNase inhibitor) (Ribonuclease/angiogenin inhibitor 1) (RAI)	50 kDa	0.51	26	12	14	1	6	4	1	4	4	4	2	1.288665313	0% (0.31)
A8K787_HUMAN (+1)	Q59GW8_HUMAN		Q59GW8	Succinate dehydrogenase complex, subunit A, flavoprotein variant (Fragment)	73 kDa	0.61	127	59	68	10	18	19	12	35	6	13	14	1.287134928	0% (0.085)
B2R673_HUMAN (+2)	Q32Q12_HUMAN	NME1-NME2	Q32Q12	Nucleoside diphosphate kinase (EC 2.7.4.6)	33 kDa	0.17	85	39	45	9	9	11	11	13	9	13	10	1.286693379	0% (0.14)
PTCD3_HUMAN	MRRP1_HUMAN	TRMT10C	Q7L0Y3	Mitochondrial ribonuclease P protein 1 (Mitochondrial RNase P protein 1) (EC 2.1.1.-) (HBV pre-S2 trans-regulated protein 2) (RNA (guanine-9-)-methyltransferase domain-containing protein 1) (Renal carcinoma antigen NY-REN-49) (tRNA methyltransferase 10 homolog C)	47 kDa	0.18	42	19	22	5	4	4	6	7	3	4	8	1.285567921	0% (0.25)
B3KUZ7_HUMAN (+3)	Q9BR63_HUMAN	FARSB	Q9BR63	FARSB protein (Fragment)	66 kDa	0.15	56	26	30	1	9	8	8	14	9	7	0	1.284078393	0% (0.20)
B3KY04_HUMAN (+1)	DDX21_HUMAN	DDX21	Q9NR30	Nucleolar RNA helicase 2 (EC 3.6.4.13) (DEAD box protein 21) (Gu-alpha) (Nucleolar RNA helicase Gu) (Nucleolar RNA helicase II) (RH II/Gu)	87 kDa	0.19	70	33	38	3	8	12	9	7	9	19	3	1.283210891	0% (0.17)
SPB6_HUMAN	Q567R6_HUMAN	SSBP1	Q567R6	Single-stranded DNA-binding protein	17 kDa	0.34	86	40	46	13	7	6	14	21	5	6	14	1.28264875	0% (0.14)
B2R806_HUMAN (+2)	STT3A_HUMAN	STT3A	P46977	Dolichyl-diphosphooligosaccharide--protein glycosyltransferase subunit STT3A (Oligosaccharyl transferase subunit STT3A) (STT3-A) (EC 2.4.99.18) (B5) (Integral membrane protein 1) (Transmembrane protein TMC)	81 kDa	0.19	145	68	78	25	20	15	7	19	27	12	21	1.281601835	0% (0.073)
B4DSN3_HUMAN (+2)	B2RWP9_HUMAN	MYH10	B2RWP9	MYH10 protein	230 kDa	0.11	193	90	103	29	19	14	29	23	16	48	16	1.27952955	95% (0.046)
BAIP2_HUMAN	EFTU_HUMAN	TUFM	P49411	Elongation factor Tu, mitochondrial (EF-Tu) (P43)	50 kDa	0.18	241	112	128	27	29	29	31	45	11	30	42	1.278300606	95% (0.030)
B4DPD5_HUMAN (+3)	TEX10_HUMAN	TEX10	Q9NXF1	Testis-expressed sequence 10 protein	106 kDa	0.23	58	27	31	11	2	2	12	11	6	4	11	1.278262235	0% (0.20)
J9JIE6_HUMAN	SMU1_HUMAN	SMU1	Q2TAY7	WD40 repeat-containing protein SMU1 (Smu-1 suppressor of mec-8 and unc-52 protein homolog) [Cleaved into: WD40 repeat-containing protein SMU1, N-terminally processed]	58 kDa	0.17	43	20	23	2	7	7	4	6	1	8	8	1.277763781	0% (0.24)
MUC16_HUMAN	MTA2_HUMAN	MTA2	O94776	Metastasis-associated protein MTA2 (Metastasis-associated 1-like 1) (MTA1-L1 protein) (p53 target protein in deacetylase complex)	75 kDa	0.0043	29	13	15	4	5	4	4	4	1	8	4	1.276807256	0% (0.31)
B3KNK5_HUMAN (+1)	B3KMC9_HUMAN		B3KMC9	cDNA FLJ10711 fis, clone NT2RP3000917, highly similar to 5'-3' exoribonuclease 2 (EC 3.1.11.-)	109 kDa	0.12	27	13	15	1	4	6	2	6	0	6	2	1.276807256	0% (0.31)
TOP1_HUMAN	NDUAA_HUMAN	NDUFA10	O95299	NADH dehydrogenase [ubiquinone] 1 alpha subcomplex subunit 10, mitochondrial (Complex I-42kD) (CI-42kD) (NADH-ubiquinone oxidoreductase 42 kDa subunit)	41 kDa	0.14	28	13	15	6	2	2	3	6	1	4	4	1.276807256	0% (0.31)
RPR1B_HUMAN	SPRE_HUMAN	SPR	P35270	Sepiapterin reductase (SPR) (EC 1.1.1.153)	28 kDa	0.23	28	13	15	3	4	5	1	2	8	2	3	1.276807256	0% (0.31)
B2R7T8_HUMAN	RL35A_HUMAN	RPL35A	P18077	60S ribosomal protein L35a (Cell growth-inhibiting gene 33 protein)	13 kDa	0.3	13	6	7	2	1	2	1	1	3	1	2	1.274065809	0% (0.41)
ERG7_HUMAN	NOL11_HUMAN	NOL11	Q9H8H0	Nucleolar protein 11	81 kDa	0.21	13	6	7	1	3	1	1	3	1	2	1	1.274065809	0% (0.41)
RS25_HUMAN	ITPA_HUMAN	ITPA	Q9BY32	Inosine triphosphate pyrophosphatase (ITPase)	21 kDa	0.26	13	6	7	0	0	0	6	6	1	0	0	1.274065809	0% (0.41)

				(Inosine triphosphatase) (EC 3.6.1.19) (Non-canonical purine NTP pyrophosphatase) (Non-standard purine NTP pyrophosphatase) (Nucleoside-triphosphate diphosphatase) (Nucleoside-triphosphate pyrophosphatase) (NTPase) (Putative oncogene protein hlc14-06-p)															
RL36_HUMAN	B3KX82_HUMAN		B3KX82	cDNA FLJ44964 fis, clone BRAWH2016209, highly similar to Homo sapiens synovial apoptosis inhibitor 1, synoviolin (SYVN1), transcript variant 2, mRNA	70 kDa	0.3	13	6	7	1	0	1	4	2	0	2	3	1.274065809	0% (0.41)
S10A4_HUMAN	PFD2_HUMAN	PFDN2	Q9UHV9	Prefoldin subunit 2	17 kDa	0.36	13	6	7	0	2	3	1	3	2	2	0	1.274065809	0% (0.41)
B7ZL00_HUMAN (+2)	G45IP_HUMAN	GADD45GIP1	Q8TAE8	Growth arrest and DNA damage-inducible proteins-interacting protein 1 (39S ribosomal protein L59, mitochondrial) (MRP-L59) (CKII beta-associating protein) (CR6-interacting factor 1) (CRIF1) (Papillomavirus L2-interacting nuclear protein 1) (PLINP) (PLINP-1) (p53-responsive gene 6 protein)	25 kDa	0.11	13	6	7	5	1	0	0	2	0	2	3	1.274065809	0% (0.41)
E7EUY3_HUMAN	RS8_HUMAN	RPS8	P62241	40S ribosomal protein S8	24 kDa	0.23	107	50	57	12	13	14	13	16	18	14	9	1.272755205	0% (0.12)
LAMC1_HUMAN	DHX30_HUMAN	DHX30	Q7L2E3	Putative ATP-dependent RNA helicase DHX30 (EC 3.6.4.13) (DEAH box protein 30)	134 kDa	0.065	43	21	24	10	6	3	3	11	3	5	3	1.270661292	0% (0.24)
RL3_HUMAN	SCPDL_HUMAN	SCCPDH	Q8NBX0	Saccharopine dehydrogenase-like oxidoreductase (EC 1.-.-.-)	47 kDa	0.19	45	21	24	14	1	0	6	2	6	6	10	1.270661292	0% (0.24)
K7ELQ9_HUMAN (+1)	LRP1_HUMAN	LRP1	Q07954	Prolow-density lipoprotein receptor-related protein 1 (LRP-1) (Alpha-2-macroglobulin receptor) (A2MR) (Apolipoprotein E receptor) (APOER) (CD antigen CD91) [Cleaved into: Low-density lipoprotein receptor-related protein 1 85 kDa subunit (LRP-85); Low-density lipoprotein receptor-related protein 1 515 kDa subunit (LRP-515); Low-density lipoprotein receptor-related protein 1 intracellular domain (LRPICD)]	505 kDa	0.31	77	36	41	6	13	15	2	0	3	12	26	1.27005168	0% (0.17)
ARL8A_HUMAN (+1)	LDHB_HUMAN	LDHB	P07195	L-lactate dehydrogenase B chain (LDH-B) (EC 1.1.1.27) (LDH heart subunit) (LDH-H) (Renal carcinoma antigen NY-REN-46)	37 kDa	0.46	107	51	58	6	14	8	22	19	15	14	11	1.269829905	0% (0.12)
PSME1_HUMAN (+1)	ESYT1_HUMAN	ESYT1	Q9BSJ8	Extended synaptotagmin-1 (E-Syt1) (Membrane-bound C2 domain-containing protein)	123 kDa	0.35	252	119	135	45	30	28	17	32	21	40	43	1.269102724	95% (0.030)
B3KMX0_HUMAN (+1)	OAT_HUMAN	OAT	P04181	Ornithine aminotransferase, mitochondrial (EC 2.6.1.13) (Ornithine delta-aminotransferase) (Ornithine--oxo-acid aminotransferase) [Cleaved into: Ornithine aminotransferase, hepatic form; Ornithine aminotransferase, renal form]	49 kDa	0.2	94	44	50	13	10	8	13	12	19	6	13	1.26825868	0% (0.14)
B4DIA6_HUMAN (+4)	Q59GM9_HUMAN		Q59GM9	Phosphorylase (EC 2.4.1.1) (Fragment)	99 kDa	0.43	62	29	33	8	3	5	12	6	23	4	0	1.267783878	0% (0.20)
STXB3_HUMAN	B7ZM99_HUMAN	MTHFD1L	B7ZM99	MTHFD1L protein (Methylenetetrahydrofolate dehydrogenase (NADP+ dependent) 1-like)	106 kDa	0.11	62	29	33	14	4	7	4	12	6	4	11	1.267783878	0% (0.20)
Q5QPL9_HUMAN	TOP2B_HUMAN	TOP2B	Q02880	DNA topoisomerase 2-beta (EC 5.99.1.3) (DNA topoisomerase II, beta isozyme)	183 kDa	0.2	111	52	59	12	15	13	12	24	15	8	12	1.26701454	0% (0.12)

MBOA7_HUMAN	AAAT_HUMAN	SLC1A5	Q15758	Neutral amino acid transporter B(0) (ATB(0)) (Baboon M7 virus receptor) (RD114/simian type D retrovirus receptor) (Sodium-dependent neutral amino acid transporter type 2) (Solute carrier family 1 member 5)	57 kDa	0.13	30	14	16	3	3	5	3	3	4	6	3	1.26650458	0% (0.31)
J3KQU9_HUMAN	CLPP_HUMAN	CLPP	Q16740	Putative ATP-dependent Clp protease proteolytic subunit, mitochondrial (EC 3.4.21.92) (Endopeptidase Clp)	30 kDa	0.082	30	14	16	4	4	2	4	7	3	3	3	1.26650458	0% (0.31)
A4D2P0_HUMAN (+1)	A8K766_HUMAN		A8K766	cDNA FLJ77343, highly similar to Homo sapiens electron-transfer-flavoprotein, beta polypeptide(ETFB), mRNA	28 kDa	0.18	162	76	86	21	21	13	22	27	22	17	19	1.264870115	0% (0.073)
B4DG42_HUMAN (+4)	F8W6I7_HUMAN	HNRNPA1	F8W6I7	Heterogeneous nuclear ribonucleoprotein A1	34 kDa	0.098	298	141	159	11	39	44	48	48	22	58	31	1.261881003	95% (0.023)
B7Z7X8_HUMAN	ABCD3_HUMAN	ABCD3	P28288	ATP-binding cassette sub-family D member 3 (70 kDa peroxisomal membrane protein) (PMP70)	75 kDa	0.076	117	54	61	21	9	9	16	12	24	11	16	1.261689739	0% (0.12)
RAB8A_HUMAN	RUVB2_HUMAN	RUVBL2	Q9Y230	RuvB-like 2 (EC 3.6.4.12) (48 kDa TATA box-binding protein-interacting protein) (48 kDa TBP-interacting protein) (51 kDa erythrocyte cytosolic protein) (ECP-51) (INO80 complex subunit J) (Repressing pontin 52) (Reptin 52) (TIP49b) (TIP60-associated protein 54-beta) (TAP54-beta)	51 kDa	0.36	149	70	79	11	16	22	22	20	30	17	13	1.261330462	0% (0.085)
GCDH_HUMAN	AGK_HUMAN	AGK	Q53H12	Acylglycerol kinase, mitochondrial (hAGK) (EC 2.7.1.107) (EC 2.7.1.94) (Multiple substrate lipid kinase) (HsMuLK) (MuLK) (Multi-substrate lipid kinase)	47 kDa	0.16	32	15	17	4	4	1	6	6	0	1	8	1.257470137	0% (0.30)
F5H569_HUMAN (+2)	H3BN02_HUMAN	ITGAX	H3BN02	Integrin alpha-X	129 kDa	0.14	32	15	17	3	5	7	0	0	0	4	13	1.257470137	0% (0.30)
Q8N7G1_HUMAN	PLOD3_HUMAN	PLOD3	O60568	Procollagen-lysine,2-oxoglutarate 5-dioxygenase 3 (EC 1.14.11.4) (Lysyl hydroxylase 3) (LH3)	85 kDa	0.31	84	40	45	11	14	11	3	15	6	12	15	1.255484673	0% (0.16)
UBP5_HUMAN	B4DKN9_HUMAN		B4DKN9	cDNA FLJ57740, highly similar to Transforming protein RhoA	20 kDa	0.14	85	40	45	7	8	11	14	9	22	10	4	1.255484673	0% (0.16)
F8VQX6_HUMAN (+2)	RISC_HUMAN	SCPEP1	Q9HB40	Retinoid-inducible serine carboxypeptidase (EC 3.4.16.-) (Serine carboxypeptidase 1)	51 kDa	0.23	16	7	8	0	3	4	0	2	0	4	2	1.255348682	0% (0.41)
AMRP_HUMAN (+1)	QCR7_HUMAN	UQCRB	P14927	Cytochrome b-c1 complex subunit 7 (Complex III subunit 7) (Complex III subunit VII) (QP-C) (Ubiquinol-cytochrome c reductase complex 14 kDa protein)	14 kDa	0.18	16	7	8	3	1	1	2	2	1	1	4	1.255348682	0% (0.41)
B4DDT3_HUMAN (+1)	A8K6F0_HUMAN		A8K6F0	cDNA FLJ75449, highly similar to Homo sapiens aquarius homolog (mouse) (AQR), mRNA	171 kDa	0.21	14	7	8	3	1	1	2	2	0	4	1	1.255348682	0% (0.41)
LRBA_HUMAN	AMPB_HUMAN	RNPEP	Q9H4A4	Aminopeptidase B (AP-B) (EC 3.4.11.6) (Arginine aminopeptidase) (Arginyl aminopeptidase)	73 kDa	0.046	15	7	8	0	2	2	3	2	5	0	1	1.255348682	0% (0.41)
PARP9_HUMAN	B3KQF4_HUMAN		B3KQF4	cDNA FLJ90373 fis, clone NT2RP2004606, highly similar to Metalloproteinase inhibitor 1	23 kDa	0.096	15	7	8	0	5	2	0	0	3	2	3	1.255348682	0% (0.41)
ACATN_HUMAN	LBR_HUMAN	LBR	Q14739	Lamin-B receptor (Integral nuclear envelope inner membrane protein) (LMN2R)	71 kDa	0.11	15	7	8	2	1	2	2	0	4	2	2	1.255348682	0% (0.41)
FKB10_HUMAN	A8K2X4_HUMAN		A8K2X4	cDNA FLJ75401, highly similar to Homo sapiens endoglin (Osler-Rendu-Weber syndrome 1), mRNA	71 kDa	0.2	15	7	8	2	1	4	0	0	3	3	2	1.255348682	0% (0.41)
ASNA_HUMAN	D6REA1_HUMAN	SIL1	D6REA1	Nucleotide exchange factor SIL1	53 kDa	0.13	15	7	8	1	3	2	1	1	1	3	3	1.255348682	0% (0.41)

BAZ1B_HUMA N	B2R6P4_HUMA N		B2R6P4	cDNA, FLJ93048, highly similar to Homo sapiens synaptobrevin-like 1 (SYBL1), mRNA	25 kDa	0.11	15	7	8	0	3	2	2	2	0	1	5	1.2553486 82	0% (0.41)
B0YJ32_HUMA N (+1)	NQO1_HUMAN	NQO1	P15559	NAD(P)H dehydrogenase [quinone] 1 (EC 1.6.5.2) (Azoreductase) (DT-diaphorase) (DTD) (Menadione reductase) (NAD(P)H:quinone oxidoreductase 1) (Phylloquinone reductase) (Quinone reductase 1) (QR1)	31 kDa	0.032	15	7	8	0	0	1	6	6	0	2	0	1.2553486 82	0% (0.41)
E7ENA9_HUMA N (+1)	Q53GN4_HUMA N	WDR1	Q53GN4	WD repeat domain 1, isoform CRA_a (WD repeat-containing protein 1 isoform 1 variant) (Fragment)	66 kDa	0.11	51	24	27	4	9	6	5	4	9	9	5	1.2527300 84	0% (0.24)
B4E206_HUMA N (+2)	TBB2B_HUMA N	TUBB2B	Q9BVA1	Tubulin beta-2B chain	50 kDa	0.11	484	230	257	71	58	51	52	67	73	54	61	1.2512598 21	95% (0.0066)
ROA0_HUMAN	B4DLW8_HUM AN	DDX5	B4DLW 8	Probable ATP-dependent RNA helicase DDX5 (cDNA FLJ59339, highly similar to Probable ATP-dependent RNA helicase DDX5 (EC 3.6.1.-))	61 kDa	0.13	161	76	85	17	21	19	20	21	15	32	17	1.2503550 28	0% (0.084)
OCAD2_HUMA N	ANXA7_HUMA N	ANXA7	P20073	Annexin A7 (Annexin VII) (Annexin-7) (Synexin)	53 kDa	0.41	88	42	47	14	8	10	11	7	17	12	11	1.2492120 77	0% (0.16)
B4DTE8_HUMA N (+3)	E9PKU7_HUMA N	GANAB	E9PKU7	Neutral alpha-glucosidase AB	97 kDa	0.1	366	174	194	42	56	45	32	35	43	51	0	1.2480740 57	95% (0.017)
PX11B_HUMAN	B4DJ38_HUMA N		B4DJ38	cDNA FLJ56092, highly similar to Pentatricopeptide repeat protein 1	84 kDa	0.21	53	25	28	8	5	5	9	10	8	5	6	1.2476640 69	0% (0.24)
Q6URC4_HUM AN	LAMC1_HUMA N	LAMC1	P11047	Laminin subunit gamma-1 (Laminin B2 chain) (Laminin-1 subunit gamma) (Laminin-10 subunit gamma) (Laminin-11 subunit gamma) (Laminin-2 subunit gamma) (Laminin-3 subunit gamma) (Laminin-4 subunit gamma) (Laminin-6 subunit gamma) (Laminin-7 subunit gamma) (Laminin-8 subunit gamma) (Laminin-9 subunit gamma) (S-laminin subunit gamma) (S-LAM gamma)	178 kDa	0.086	36	17	19	0	8	8	1	6	2	4	7	1.2423720 46	0% (0.30)
DAD1_HUMAN (+1)	ANXA3_HUMA N	ANXA3	P12429	Annexin A3 (35-alpha calcimedin) (Annexin III) (Annexin-3) (Inositol 1,2-cyclic phosphate 2-phosphohydrolase) (Lipocortin III) (Placental anticoagulant protein III) (PAP-III)	36 kDa	0.28	117	54	60	27	1	4	22	17	8	14	21	1.2414037 58	0% (0.14)
RT09_HUMAN	SRPRB_HUMA N	SRPRB	Q9Y5M8	Signal recognition particle receptor subunit beta (SR-beta) (Protein APMCF1)	30 kDa	0.22	113	54	60	16	12	13	13	19	17	13	12	1.2414037 58	0% (0.14)
MOT4_HUMAN	RM47_HUMAN	MRPL47	Q9HD33	39S ribosomal protein L47, mitochondrial (L47mt) (MRP-L47) (Nasopharyngeal carcinoma metastasis-related protein 1)	29 kDa	0.088	17	8	9	3	2	2	1	3	1	1	4	1.2406788 73	0% (0.40)
B2RAR6_HUMA N (+1)	B2R6X2_HUMA N		B2R6X2	cDNA, FLJ93161, highly similar to Homo sapiens glucuronidase, beta (GUSB), mRNA	75 kDa	0.082	17	8	9	2	2	3	1	1	0	3	5	1.2406788 73	0% (0.40)
ALG5_HUMAN (+1)	SIN3A_HUMAN	SIN3A	Q96ST3	Paired amphipathic helix protein Sin3a (Histone deacetylase complex subunit Sin3a) (Transcriptional corepressor Sin3a)	145 kDa	0.27	17	8	9	1	1	1	5	1	1	5	2	1.2406788 73	0% (0.40)
A8K8G0_HUMA N (+2)	DEST_HUMAN	DSTN	P60981	Destrin (Actin-depolymerizing factor) (ADF)	19 kDa	0.25	19	8	9	1	2	3	2	2	4	2	2	1.2406788 73	0% (0.40)
VIGLN_HUMA N	K6PF_HUMAN	PFKM	P08237	6-phosphofructokinase, muscle type (EC 2.7.1.11) (Phosphofructo-1-kinase isozyme A) (PFK-A) (Phosphofructokinase-M) (Phosphofructokinase 1) (Phosphohexokinase)	85 kDa	0.12	17	8	9	1	5	1	0	2	5	2	0	1.2406788 73	0% (0.40)

PGRC2_HUMAN	RL7_HUMAN	RPL7	P18124	60S ribosomal protein L7	29 kDa	0.22	116	55	61	13	9	14	20	12	19	11	18	1.239243766	0% (0.14)
ERMP1_HUMAN	Q53EP4_HUMAN		Q53EP4	Ribophorin I variant (Fragment)	69 kDa	0.13	297	142	157	48	35	32	28	40	34	39	44	1.237381488	95% (0.034)
Q9BQQ5_HUMAN	K7ELQ9_HUMAN	TMEM205	K7ELQ9	Transmembrane protein 205 (Fragment)	19 kDa	0.43	38	18	20	6	4	0	8	2	7	3	8	1.235999744	0% (0.30)
H2B1B_HUMAN (+3)	B4E206_HUMAN		B4E206	cDNA FLJ55441, highly similar to Nuclear pore complex protein Nup133	127 kDa	0.61	37	18	20	3	5	3	7	7	1	6	6	1.235999744	0% (0.30)
PR40A_HUMAN	ROA0_HUMAN	HNRNPA0	Q13151	Heterogeneous nuclear ribonucleoprotein A0 (hnRNP A0)	31 kDa	0.057	38	18	20	2	6	5	5	7	4	4	5	1.235999744	0% (0.30)
F162A_HUMAN (+1)	Q8IWP6_HUMAN		Q8IWP6	Class IVb beta tubulin	50 kDa	0.31	574	274	302	81	62	69	64	77	86	71	67	1.2345206	95% (0.0056)
Q6FGX3_HUMAN (+1)	B4DJG6_HUMAN		B4DJG6	cDNA FLJ54691, highly similar to Thromboxane-A synthase (EC 5.3.99.5)	66 kDa	0.43	102	48	53	8	14	18	9	0	14	11	28	1.233452665	0% (0.16)
K7EPB2_HUMAN	IF4A3_HUMAN	EIF4A3	P38919	Eukaryotic initiation factor 4A-III (eIF-4A-III) (eIF4A-III) (EC 3.6.4.13) (ATP-dependent RNA helicase DDX48) (ATP-dependent RNA helicase eIF4A-3) (DEAD box protein 48) (Eukaryotic initiation factor 4A-like NUK-34) (Eukaryotic translation initiation factor 4A isoform 3) (Nuclear matrix protein 265) (NMP 265) (hNMP 265) [Cleaved into: Eukaryotic initiation factor 4A-III, N-terminally processed]	47 kDa	0.14	126	58	64	10	12	20	16	27	10	14	14	1.233201608	0% (0.14)
PGRC1_HUMAN	B5BTY4_HUMAN	DDX3X	B5BTY4	ATP-dependent RNA helicase DDX3X	73 kDa	0.16	122	58	64	12	18	13	16	19	8	18	19	1.233201608	0% (0.14)
RAB3D_HUMAN	PHB2_HUMAN	PHB2	Q99623	Prohibitin-2 (B-cell receptor-associated protein BAP37) (D-prohibitin) (Repressor of estrogen receptor activity)	33 kDa	0.3	248	118	130	36	20	19	42	27	38	28	37	1.232709002	0% (0.052)
PP2AA_HUMAN	B4DZI8_HUMAN	COPB2	B4DZI8	Coatomer protein complex, subunit beta 2 (Beta prime), isoform CRA_b (Coatomer subunit beta') (cDNA FLJ56271, highly similar to Coatomer subunit beta)	99 kDa	0.27	61	29	32	6	8	9	6	11	12	6	3	1.230750579	0% (0.24)
AGK_HUMAN (+2)	ARPC4_HUMAN	ARPC4	P59998	Actin-related protein 2/3 complex subunit 4 (Arp2/3 complex 20 kDa subunit) (p20-ARC)	20 kDa	0.2	42	19	21	3	6	5	5	5	7	3	6	1.230256975	0% (0.30)
DDX1_HUMAN	AP3B1_HUMAN	AP3B1	O00203	AP-3 complex subunit beta-1 (Adapter-related protein complex 3 subunit beta-1) (Adaptor protein complex AP-3 subunit beta-1) (Beta-3A-adaptin) (Clathrin assembly protein complex 3 beta-1 large chain)	121 kDa	0.16	19	9	10	1	2	6	0	1	0	4	5	1.228871802	0% (0.40)
Q6MZM3_HUMAN (+1)	AL5AP_HUMAN	ALOX5AP	P20292	Arachidonate 5-lipoxygenase-activating protein (FLAP) (MK-886-binding protein)	18 kDa	0.087	19	9	10	1	3	5	0	0	3	2	5	1.228871802	0% (0.40)
B1AVQ5_HUMAN (+2)	MIPEP_HUMAN	MIPEP	Q99797	Mitochondrial intermediate peptidase (MIP) (EC 3.4.24.59)	81 kDa	0.072	19	9	10	7	0	3	0	6	0	1	3	1.228871802	0% (0.40)
CP1B1_HUMAN	C9JRZ6_HUMAN	CHCHD3	C9JRZ6	Coiled-coil-helix-coiled-coil-helix domain-containing protein 3, mitochondrial	27 kDa	0.13	19	9	10	2	1	1	5	5	1	1	3	1.228871802	0% (0.40)
DPP4_HUMAN	A8K494_HUMAN		A8K494	cDNA FLJ78440, highly similar to Human lactoferrin	78 kDa	0.16	19	9	10	2	2	2	3	3	3	3	1	1.228871802	0% (0.40)
LYRIC_HUMAN	B3KP52_HUMAN		B3KP52	cDNA FLJ31171 fis, clone KIDNE2000046, highly similar to 3-mercaptopyruvate sulfurtransferase	33 kDa	0.11	19	9	10	2	2	2	3	3	4	2	1	1.228871802	0% (0.40)
B5MBZ0_HUMAN (+1)	A8K800_HUMAN		A8K800	cDNA FLJ76924, highly similar to Homo sapiens brix domain containing 1 (BXDC1), mRNA	36 kDa	0.057	19	9	10	1	3	1	4	2	3	3	2	1.228871802	0% (0.40)
A2AB90_HUMAN (+2)	PTPM1_HUMAN	PTPMT1	Q8WUK0	Phosphatidylglycerophosphatase and protein-tyrosine phosphatase 1 (EC 3.1.3.27)	23 kDa	0.14	19	9	10	3	2	2	2	1	4	3	2	1.228871802	0% (0.40)

				(PTEN-like phosphatase) (Phosphoinositide lipid phosphatase) (Protein-tyrosine phosphatase mitochondrial 1) (EC 3.1.3.16) (EC 3.1.3.48)														
CS010_HUMAN	B4DRW3_HUMAN		B4DRW3	cDNA FLJ57180, highly similar to TAR DNA-binding protein 43	35 kDa	0.27	19	9	10	2	2	2	3	2	4	2	2	1.228871802	0% (0.40)
B4DDB9_HUMAN (+1)	B4DED6_HUMAN		B4DED6	cDNA FLJ56545, highly similar to ATP-dependent RNA helicase DDX50 (EC 3.6.1.-)	72 kDa	0.23	19	9	10	0	2	4	2	1	2	6	0	1.228871802	0% (0.40)
B4E2X9_HUMAN	Q58EY4_HUMAN	SMARCC1	Q58EY4	SWI/SNF related, matrix associated, actin dependent regulator of chromatin, subfamily c, member 1	123 kDa	0.1	19	9	10	0	2	2	0	4	0	5	1	1.228871802	0% (0.40)
EFTS_HUMAN	AT1A1_HUMAN	ATP1A1	P05023	Sodium/potassium-transporting ATPase subunit alpha-1 (Na(+)/K(+) ATPase alpha-1 subunit) (EC 3.6.3.9) (Sodium pump subunit alpha-1)	113 kDa	0.16	326	156	171	44	44	44	23	42	25	45	59	1.226965929	95% (0.033)
B4DH58_HUMAN (+1)	1433Z_HUMAN	YWHAZ	P63104	14-3-3 protein zeta/delta (Protein kinase C inhibitor protein 1) (KCIP-1)	28 kDa	0.17	107	51	56	7	13	13	18	20	11	12	13	1.226931121	0% (0.16)
Q860C0_HUMAN	E9PCY7_HUMAN	HNRNPH1	E9PCY7	Heterogeneous nuclear ribonucleoprotein H	47 kDa	0.27	178	84	92	22	22	17	22	23	18	27	24	1.224972344	0% (0.096)
A8K4K9_HUMAN (+2)	KAD4_HUMAN	AK4	P27144	Adenylate kinase 4, mitochondrial (AK 4) (EC 2.7.4.10) (EC 2.7.4.6) (Adenylate kinase 3-like) (GTP:AMP phosphotransferase AK4)	25 kDa	0.23	108	52	57	7	16	21	10	18	15	12	12	1.224920697	0% (0.16)
A6NEM2_HUMAN (+1)	AL7A1_HUMAN	ALDH7A1	P49419	Alpha-aminoadipic semialdehyde dehydrogenase (Alpha-AASA dehydrogenase) (EC 1.2.1.31) (Aldehyde dehydrogenase family 7 member A1) (EC 1.2.1.3) (Antiquitin-1) (Betaine aldehyde dehydrogenase) (EC 1.2.1.8) (Delta1-piperideine-6-carboxylate dehydrogenase) (P6c dehydrogenase)	58 kDa	0.034	198	95	104	29	27	19	21	52	14	13	27	1.224635432	0% (0.081)
Q59GW5_HUMAN (+1)	PRKDC_HUMAN	PRKDC	P78527	DNA-dependent protein kinase catalytic subunit (DNA-PK catalytic subunit) (DNA-PKcs) (EC 2.7.11.1) (DNPK1) (p460)	469 kDa	0.15	470	225	246	21	52	67	86	79	52	93	20	1.22432195	95% (0.014)
F5GYR8_HUMAN (+2)	F5H5D3_HUMAN	TUBA1C	F5H5D3	Tubulin alpha-1C chain	58 kDa	0.11	437	209	228	61	49	50	50	58	65	55	51	1.221512613	95% (0.018)
AUP1_HUMAN	RAB18_HUMAN	RAB18	Q9NP72	Ras-related protein Rab-18	23 kDa	0.16	90	43	47	13	10	11	9	3	18	13	13	1.2209656	0% (0.19)
BASI_HUMAN (+1)	ATPB_HUMAN	ATP5B	P06576	ATP synthase subunit beta, mitochondrial (EC 3.6.3.14)	57 kDa	0.21	793	380	414	96	92	91	101	112	90	99	113	1.220770425	95% (0.0023)
A0S1I7_HUMAN (+277)	A6NEM2_HUMAN	HCFC1	A6NEM2	HCF N-terminal chain 5	213 kDa	0.076	24	10	11	2	3	2	3	2	1	4	4	1.219164072	0% (0.39)
B4DT31_HUMAN (+4)	B3KNC3_HUMAN		B3KNC3	cDNA FLJ14222 fis, clone NT2RP3003992, highly similar to Nucleolar complex protein 2 homolog	85 kDa	0.15	21	10	11	0	3	2	5	4	3	3	1	1.219164072	0% (0.39)
SAHH_HUMAN	RM38_HUMAN	MRPL38	Q96DV4	39S ribosomal protein L38, mitochondrial (L38mt) (MRP-L38)	45 kDa	0.16	21	10	11	5	2	1	2	5	2	2	2	1.219164072	0% (0.39)
B4DWI8_HUMAN	F5H315_HUMAN	XAB2	F5H315	Pre-mRNA-splicing factor SYF1	100 kDa	0.091	21	10	11	4	1	2	3	7	0	3	1	1.219164072	0% (0.39)
THEM6_HUMAN	NDUAC_HUMAN	NDUFA12	Q9UI09	NADH dehydrogenase [ubiquinone] 1 alpha subcomplex subunit 12 (13 kDa differentiation-associated protein) (Complex I-B17.2) (CI-B17.2) (CIB17.2) (NADH-ubiquinone oxidoreductase subunit B17.2)	17 kDa	0.25	21	10	11	5	1	2	2	2	2	3	4	1.219164072	0% (0.39)
J3KT73_HUMAN (+2)	IDH3B_HUMAN	IDH3B	O43837	Isocitrate dehydrogenase [NAD] subunit beta, mitochondrial (EC 1.1.1.41) (Isocitric dehydrogenase	42 kDa	0.39	92	44	48	12	9	7	16	17	8	10	13	1.218730571	0% (0.19)

				subunit beta) (NAD(+)-specific ICDH subunit beta)															
DEGS1_HUMAN	ANM1_HUMAN	PRMT1	Q99873	Protein arginine N-methyltransferase 1 (EC 2.1.1.-) (Histone-arginine N-methyltransferase PRMT1) (EC 2.1.1.125) (Interferon receptor 1-bound protein 4)	42 kDa	0.096	68	33	36	12	7	6	8	13	16	6	1	1.217789295	0% (0.23)
RAD21_HUMAN	IPYR_HUMAN	PPA1	Q15181	Inorganic pyrophosphatase (EC 3.6.1.1) (Pyrophosphate phospho-hydrolase) (PPase)	33 kDa	0.15	69	33	36	2	12	2	17	5	15	12	4	1.217789295	0% (0.23)
KCY_HUMAN	F6KPG5_HUMAN		F6KPG5	Albumin (Fragment)	67 kDa	0.34	263	126	137	17	30	37	42	22	40	24	53	1.216819324	0% (0.058)
B3KTJ9_HUMAN (+1)	M1RL68_HUMAN	HLA-A	M1RL68	MHC class I antigen	41 kDa	0.11	119	57	62	10	15	13	19	0	0	0	32	1.215904874	0% (0.16)
ACO13_HUMAN	NB5R3_HUMAN	CYB5R3	P00387	NADH-cytochrome b5 reductase 3 (B5R) (Cytochrome b5 reductase) (EC 1.6.2.2) (Diaphorase-1) [Cleaved into: NADH-cytochrome b5 reductase 3 membrane-bound form; NADH-cytochrome b5 reductase 3 soluble form]	34 kDa	0.38	145	69	75	25	14	14	16	8	22	17	29	1.215465958	0% (0.13)
A8K4W7_HUMAN (+2)	TPR_HUMAN	TPR	P12270	Nucleoprotein TPR (Megator) (NPC-associated intranuclear protein) (Translocated promoter region protein)	267 kDa	0.12	96	46	50	14	10	14	8	8	2	25	16	1.214544546	0% (0.19)
Q5W0H4_HUMAN (+1)	PDIA4_HUMAN	PDIA4	P13667	Protein disulfide-isomerase A4 (EC 5.3.4.1) (Endoplasmic reticulum resident protein 70) (ER protein 70) (ERp70) (Endoplasmic reticulum resident protein 72) (ER protein 72) (ERp-72) (ERp72)	73 kDa	0.22	293	141	153	27	50	39	25	26	38	41	48	1.214529225	95% (0.030)
RALA_HUMAN	B4DJB4_HUMAN		B4DJB4	cDNA FLJ55931, highly similar to Isocitrate dehydrogenase	35 kDa	0.3	170	83	90	24	17	23	19	30	13	22	24	1.212908025	0% (0.11)
GNA13_HUMAN	TNPO1_HUMAN	TNPO1	Q92973	Transportin-1 (Importin beta-2) (Karyopherin beta-2) (M9 region interaction protein) (MIP)	102 kDa	0.18	48	23	25	3	4	7	9	8	7	10	0	1.212023623	0% (0.29)
B4DRT4_HUMAN (+1)	Q5XPV6_HUMAN		Q5XPV6	Small nuclear ribonucleoprotein-associated protein	24 kDa	0.49	49	23	25	4	6	7	5	6	4	8	7	1.212023623	0% (0.29)
VTNC_HUMAN	A8K7F6_HUMAN		A8K7F6	cDNA FLJ78244, highly similar to Homo sapiens eukaryotic translation initiation factor 4A, isoform 1 (EIF4A1), mRNA	46 kDa	0.17	176	84	91	10	25	23	26	28	30	26	7	1.211818456	0% (0.11)
C9J9I1_HUMAN (+1)	ETFA_HUMAN	ETFA	P13804	Electron transfer flavoprotein subunit alpha, mitochondrial (Alpha-ETF)	35 kDa	0.078	149	72	78	19	19	18	16	23	17	18	19	1.21155443	0% (0.13)
PLPL6_HUMAN	SYFA_HUMAN	FARSA	Q9Y285	Phenylalanine--tRNA ligase alpha subunit (EC 6.1.1.20) (CML33) (Phenylalanyl-tRNA synthetase alpha subunit) (PheRS)	58 kDa	0.075	25	11	12	0	4	4	3	7	5	1	0	1.211041559	0% (0.39)
CO4B_HUMAN (+1)	PRP19_HUMAN	PRPF19	Q9UMS4	Pre-mRNA-processing factor 19 (Nuclear matrix protein 200) (PRP19/PSO4 homolog) (hPso4) (Senescence evasion factor)	55 kDa	0.1	23	11	12	1	1	5	4	3	3	5	1	1.211041559	0% (0.39)
COCA1_HUMAN (+1)	RER1_HUMAN	RER1	O15258	Protein RER1	23 kDa	0.056	23	11	12	2	2	3	4	5	2	1	4	1.211041559	0% (0.39)
ANK1_HUMAN	MK67I_HUMAN	NIFK	Q9BYG3	MKI67 FHA domain-interacting nucleolar phosphoprotein (Nucleolar phosphoprotein Nopp34) (Nucleolar protein interacting with the FHA domain of pKI-67) (hNIFK)	34 kDa	0.087	23	11	12	4	1	1	5	4	3	2	3	1.211041559	0% (0.39)
THIO_HUMAN	XPP3_HUMAN	XPNPEP3	Q9NQH7	Probable Xaa-Pro aminopeptidase 3 (X-Pro aminopeptidase 3) (EC 3.4.11.9) (Aminopeptidase P3) (APP3)	57 kDa	0.49	24	11	12	2	5	0	3	7	0	3	0	1.211041559	0% (0.39)

B3KQB4_HUMAN (+2)	ROA3_HUMAN	HNRNPA3	P51991	Heterogeneous nuclear ribonucleoprotein A3 (hnRNP A3)	40 kDa	0.22	177	85	92	14	22	20	28	20	8	39	25	1.210754193	0% (0.11)
QOR_HUMAN	E7EX73_HUMAN	EIF4G1	E7EX73	Eukaryotic translation initiation factor 4 gamma 1	159 kDa	0.29	75	36	39	3	10	13	10	13	9	14	4	1.209896062	0% (0.23)
A6NJA2_HUMAN (+3)	A8K548_HUMAN		A8K548	cDNA FLJ75008, highly similar to Homo sapiens proline-, glutamic acid-, leucine-rich protein 1 (PELP1), mRNA	120 kDa	0.2	50	24	26	8	6	3	7	7	5	3	11	1.208368268	0% (0.29)
B4DJP7_HUMAN (+1)	DHB4_HUMAN	HSD17B4	P51659	Peroxisomal multifunctional enzyme type 2 (MFE-2) (17-beta-hydroxysteroid dehydrogenase 4) (17-beta-HSD 4) (D-bifunctional protein) (DBP) (Multifunctional protein 2) (MPF-2) [Cleaved into: (3R)-hydroxyacyl-CoA dehydrogenase (EC 1.1.1.n12); Enoyl-CoA hydratase 2 (EC 4.2.1.107) (EC 4.2.1.119) (3-alpha,7-alpha,12-alpha-trihydroxy-5-beta-cholest-24-enoyl-CoA hydratase)]	80 kDa	0.16	295	142	153	48	31	41	22	29	43	31	50	1.206035313	0% (0.056)
B7ZKK7_HUMAN	B4DUX0_HUMAN	ACOT7	B4DUX0	Cytosolic acyl coenzyme A thioester hydrolase (cDNA FLJ60167, highly similar to Cytosolic acyl coenzyme A thioester hydrolase(EC 3.1.2.2))	30 kDa	0.18	25	12	13	3	3	3	3	11	2	0	0	1.204145345	0% (0.38)
BLVRB_HUMAN	HACD2_HUMAN	PTPLB	Q6Y1H2	Very-long-chain (3R)-3-hydroxyacyl-[acyl-carrier protein] dehydratase 2 (EC 4.2.1.134) (3-hydroxyacyl-CoA dehydratase 2) (HACD2) (Protein-tyrosine phosphatase-like member B)	28 kDa	0.31	25	12	13	3	5	3	1	4	3	2	4	1.204145345	0% (0.38)
LAMB3_HUMAN	UBA6_HUMAN	UBA6	A0AVT1	Ubiquitin-like modifier-activating enzyme 6 (Ubiquitin-activating enzyme 6) (Monocyte protein 4) (MOP-4) (Ubiquitin-activating enzyme E1-like protein 2) (E1-L2)	118 kDa	0.11	25	12	13	0	6	3	3	5	6	2	0	1.204145345	0% (0.38)
SYTM_HUMAN	HSP72_HUMAN	HSPA2	P54652	Heat shock-related 70 kDa protein 2 (Heat shock 70 kDa protein 2)	70 kDa	0.14	201	96	103	0	0	0	24	20	28	27	28	1.200494197	0% (0.10)
B4DM33_HUMAN (+1)	HNRPM_HUMAN	HNRNPM	P52272	Heterogeneous nuclear ribonucleoprotein M (hnRNP M)	78 kDa	0.27	199	97	104	11	30	22	33	36	19	25	23	1.199675602	0% (0.10)
B7Z815_HUMAN (+2)	B5BU61_HUMAN	HDAC1	B5BU61	Histone deacetylase (EC 3.5.1.98)	55 kDa	0.084	56	27	29	5	6	8	8	5	8	10	6	1.19895565	0% (0.28)
PUR2_HUMAN (+2)	MTCH2_HUMAN	MTCH2	Q9Y6C9	Mitochondrial carrier homolog 2 (Met-induced mitochondrial protein)	33 kDa	0.12	145	70	75	22	11	15	23	17	20	16	22	1.198391377	0% (0.15)
LAMC2_HUMAN	J3KT10_HUMAN	NUP85	J3KT10	Nuclear pore complex protein Nup85	70 kDa	0.12	27	13	14	2	4	2	5	4	3	4	3	1.198217262	0% (0.38)
ITA2_HUMAN	IN35_HUMAN	IFI35	P80217	Interferon-induced 35 kDa protein (IFP 35) (Ifi-35)	32 kDa	0.11	28	13	14	1	1	2	9	3	3	3	5	1.198217262	0% (0.38)
VATC1_HUMAN	IMA5_HUMAN	KPNA1	P52294	Importin subunit alpha-5 (Karyopherin subunit alpha-1) (Nucleoprotein interactor 1) (NPI-1) (RAG cohort protein 2) (SRP1-beta) [Cleaved into: Importin subunit alpha-5, N-terminally processed]	60 kDa	0.26	27	13	14	3	4	2	4	6	3	5	1	1.198217262	0% (0.38)
RM01_HUMAN	PSA7_HUMAN	PSMA7	O14818	Proteasome subunit alpha type-7 (EC 3.4.25.1) (Proteasome subunit RC6-1) (Proteasome subunit XAPC7)	28 kDa	0.18	27	13	14	2	2	3	6	3	3	3	5	1.198217262	0% (0.38)
Q59FH1_HUMAN	H12_HUMAN	HIST1H1C	P16403	Histone H1.2 (Histone H1c) (Histone H1d) (Histone H1s-1)	21 kDa	0.035	117	57	61	5	20	15	17	12	16	14	18	1.196663853	0% (0.18)
B2RB52_HUMAN (+4)	PSMD1_HUMAN	PSMD1	Q99460	26S proteasome non-ATPase regulatory subunit 1 (26S proteasome regulatory subunit RPN2) (26S proteasome regulatory subunit S1) (26S proteasome subunit p112)	106 kDa	0.15	58	28	30	3	9	8	8	6	13	8	4	1.196247412	0% (0.28)

A8K690_HUMAN (+2)	TBB5_HUMAN	TUBB	P07437	Tubulin beta chain (Tubulin beta-5 chain)	50 kDa	0.17	676	328	350	93	84	80	73	98	96	75	80	1.195402631	95% (0.0097)
Q5TB53_HUMAN (+2)	ECH1_HUMAN	ECH1	Q13011	Delta(3,5)-Delta(2,4)-dienoyl-CoA isomerase, mitochondrial (EC 5.3.3.-)	36 kDa	0.19	150	73	78	16	20	14	24	17	31	10	19	1.195221829	0% (0.15)
A8KAQ5_HUMAN (+1)	CH60_HUMAN	HSPD1	P10809	60 kDa heat shock protein, mitochondrial (60 kDa chaperonin) (Chaperonin 60) (CPN60) (Heat shock protein 60) (HSP-60) (Hsp60) (HuCHA60) (Mitochondrial matrix protein P1) (P60 lymphocyte protein)	61 kDa	0.11	869	422	450	86	133	110	91	118	98	120	115	1.194937522	95% (0.0040)
F8RZC1_HUMAN	RAB1A_HUMAN	RAB1A	P62820	Ras-related protein Rab-1A (YPT1-related protein)	23 kDa	0.12	217	104	111	29	27	30	20	20	32	22	38	1.194381905	0% (0.10)
F120A_HUMAN	MPPA_HUMAN	PMPCA	Q10713	Mitochondrial-processing peptidase subunit alpha (EC 3.4.24.64) (Alpha-MPP) (P-55)	58 kDa	0.063	60	29	31	6	6	5	12	14	6	4	7	1.193718345	0% (0.28)
A1L172_HUMAN (+2)	D3YTB1_HUMAN	RPL32	D3YTB1	60S ribosomal protein L32 (Fragment)	16 kDa	0.19	29	14	15	3	3	3	5	3	10	2	0	1.193066859	0% (0.38)
EFHD2_HUMAN	B4E2Q0_HUMAN	ATP2C1	B4E2Q0	ATPase, Ca++ transporting, type 2C, member 1, isoform CRA_d (cDNA FLJ55630, highly similar to Calcium-transporting ATPase type 2C member 1 (EC 3.6.3.8))	105 kDa	0.2	29	14	15	5	5	4	0	3	1	4	7	1.193066859	0% (0.38)
F5GZY0_HUMAN	A8K566_HUMAN		A8K566	cDNA FLJ78246, highly similar to Homo sapiens splicing factor 3a, subunit 3, 60kDa (SF3A3), mRNA	59 kDa	0.12	29	14	15	0	5	5	4	4	2	5	4	1.193066859	0% (0.38)
SCRB2_HUMAN	SMD1_HUMAN	SNRPD1	P62314	Small nuclear ribonucleoprotein Sm D1 (Sm-D1) (Sm-D autoantigen) (snRNP core protein D1)	13 kDa	0.13	29	14	15	3	2	3	6	3	2	6	4	1.193066859	0% (0.38)
MCM6_HUMAN (+1)	B2R7I0_HUMAN		B2R7I0	cDNA, FLJ93451, highly similar to Homo sapiens thioredoxin domain containing 14 (TXNDC14), mRNA	34 kDa	0.15	29	14	15	3	2	3	6	2	2	5	6	1.193066859	0% (0.38)
A6NNI4_HUMAN (+3)	A8K2D6_HUMAN		A8K2D6	cDNA FLJ76011, highly similar to Homo sapiens lactamase, beta 2 (LACTB2), mRNA	33 kDa	0.21	29	14	15	1	4	2	7	6	3	4	2	1.193066859	0% (0.38)
FUBP2_HUMAN	F5H0M4_HUMAN	LPCAT3	F5H0M4	Lysophospholipid acyltransferase 5	39 kDa	0.12	29	14	15	6	0	2	6	1	7	4	3	1.193066859	0% (0.38)
DRS7B_HUMAN	B4DJ30_HUMAN		B4DJ30	cDNA FLJ61290, highly similar to Neutral alpha-glucosidase AB	113 kDa	0.21	415	202	215	46	65	51	42	38	47	56	73	1.191812311	95% (0.037)
MFN2_HUMAN	ODB2_HUMAN	DBT	P11182	Lipoamide acyltransferase component of branched-chain alpha-keto acid dehydrogenase complex, mitochondrial (EC 2.3.1.168) (Branched-chain alpha-keto acid dehydrogenase complex component E2) (BCKAD-E2) (BCKADE2) (Dihydrolipoamide acetyltransferase component of branched-chain alpha-keto acid dehydrogenase complex) (Dihydrolipoamide branched chain transacylase) (Dihydrolipoyllysine-residue (2-methylpropanoyl)transferase)	53 kDa	0.13	62	30	32	12	4	3	11	12	7	7	6	1.191351248	0% (0.28)
MTX1_HUMAN	IPO7_HUMAN	IPO7	O95373	Importin-7 (Imp7) (Ran-binding protein 7) (RanBP7)	120 kDa	0.14	63	30	32	0	10	13	7	10	8	9	5	1.191351248	0% (0.28)
Q0VGA5_HUMAN (+3)	ATPO_HUMAN	ATP5O	P48047	ATP synthase subunit O, mitochondrial (Oligomycin sensitivity conferral protein) (OSCP)	23 kDa	0.19	95	46	49	13	8	8	17	11	13	13	13	1.190828974	0% (0.22)
B4DN31_HUMAN (+2)	B2R4C0_HUMAN		B2R4C0	60S ribosomal protein L18a	21 kDa	0.18	64	31	33	7	8	6	10	9	12	6	7	1.189131055	0% (0.28)
FKBP2_HUMAN	FKB10_HUMAN	FKBP10	Q96AY3	Peptidyl-prolyl cis-trans isomerase FKBP10 (PPIase FKBP10) (EC 5.2.1.8) (65 kDa FK506-binding protein) (65 kDa FKBP) (FKBP-65)	64 kDa	0.35	30	15	16	1	5	2	7	10	1	3	2	1.188550563	0% (0.37)

				(FK506-binding protein 10) (FKBP-10) (Immunophilin FKBP65) (Rotamase)															
H0YF29_HUMAN	A8K486_HUMAN		A8K486	Peptidyl-prolyl cis-trans isomerase (EC 5.2.1.8)	18 kDa	0.22	206	98	104	15	30	18	37	27	37	20	22	1.187572913	0% (0.12)
VAPB_HUMAN	E9PL22_HUMAN	HYOU1	E9PL22	Hypoxia up-regulated protein 1	105 kDa	0.25	272	132	140	27	49	37	20	32	37	32	39	1.187203676	0% (0.082)
B4DT35_HUMAN (+3)	AMRP_HUMAN	LRPAP1	P30533	Alpha-2-macroglobulin receptor-associated protein (Alpha-2-MRAP) (Low density lipoprotein receptor-related protein-associated protein 1) (RAP)	41 kDa	0.16	33	16	17	1	6	5	4	2	3	7	5	1.184558096	0% (0.37)
B4DVA7_HUMAN (+1)	DPP4_HUMAN	DPP4	P27487	Dipeptidyl peptidase 4 (EC 3.4.14.5) (ADABP) (Adenosine deaminase complexing protein 2) (ADCP-2) (Dipeptidyl peptidase IV) (DPP IV) (T-cell activation antigen CD26) (TP103) (CD antigen CD26) [Cleaved into: Dipeptidyl peptidase 4 membrane form (Dipeptidyl peptidase IV membrane form); Dipeptidyl peptidase 4 soluble form (Dipeptidyl peptidase IV soluble form)]	88 kDa	0.15	33	16	17	16	0	0	0	5	3	2	7	1.184558096	0% (0.37)
RS26_HUMAN	B2RB52_HUMAN		B2RB52	cDNA, FLJ95314, highly similar to Homo sapiens transducin (beta)-like 2 (TBL2), transcript variant 1, mRNA	50 kDa	0.31	32	16	17	3	3	6	4	2	0	8	7	1.184558096	0% (0.37)
CUL4A_HUMAN	PGK1_HUMAN	PGK1	P00558	Phosphoglycerate kinase 1 (EC 2.7.2.3) (Cell migration-inducing gene 10 protein) (Primer recognition protein 2) (PRP 2)	45 kDa	0.15	290	141	149	24	33	40	46	40	45	42	23	1.182965924	0% (0.079)
VPS45_HUMAN	H13_HUMAN	HIST1H1D	P16402	Histone H1.3 (Histone H1c) (Histone H1s-2)	22 kDa	0.11	106	52	55	5	19	14	14	12	13	13	17	1.182829277	0% (0.21)
A2MG_HUMAN	CY1_HUMAN	CYC1	P08574	Cytochrome c1, heme protein, mitochondrial (Complex III subunit 4) (Complex III subunit IV) (Cytochrome b-c1 complex subunit 4) (Ubiquinol-cytochrome-c reductase complex cytochrome c1 subunit) (Cytochrome c-1)	35 kDa	0.095	71	35	37	10	5	8	12	12	4	10	11	1.181476167	0% (0.27)
H3BN02_HUMAN (+1)	Q2TU34_HUMAN	FBP1	Q2TU34	Fructose-1,6-bisphosphatase 1 (Growth-inhibiting protein 17) (cDNA FLJ75786, highly similar to Homo sapiens fructose-1,6-bisphosphatase 1 (FBP1), mRNA)	37 kDa	0.09	72	35	37	8	12	15	0	0	7	13	17	1.181476167	0% (0.27)
B2R5V2_HUMAN (+1)	A1KYQ7_HUMAN	EIF3C	A1KYQ7	Eukaryotic translation initiation factor 3 subunit C (eIF3c) (Eukaryotic translation initiation factor 3 subunit 8) (eIF3 p110)	105 kDa	0.16	35	17	18	0	6	7	4	3	7	2	5	1.181003347	0% (0.36)
B2R4D8_HUMAN (+1)	G3V2S9_HUMAN	SLIRP	G3V2S9	SRA stem-loop-interacting RNA-binding protein, mitochondrial	14 kDa	0.21	35	17	18	3	5	5	4	6	5	4	3	1.181003347	0% (0.36)
J3KT10_HUMAN (+1)	B4DWI8_HUMAN		B4DWI8	cDNA FLJ57805, highly similar to Homo sapiens paraspeckle component 1 (PSPC1), transcript variant alpha, mRNA	53 kDa	0.11	34	17	18	8	2	2	4	1	4	6	7	1.181003347	0% (0.36)
ECI2_HUMAN (+3)	VTNC_HUMAN	VTN	P04004	Vitronectin (VN) (S-protein) (Serum-spreading factor) (V75) [Cleaved into: Vitronectin V65 subunit; Vitronectin V10 subunit; Somatomedin-B]	54 kDa	0.18	35	17	18	2	1	1	13	2	5	2	9	1.181003347	0% (0.36)
FAF2_HUMAN	B2R8R5_HUMAN		B2R8R5	cDNA, FLJ94025, highly similar to Homo sapiens tripartite motif-containing 28 (TRIM28), mRNA	89 kDa	0.17	148	72	76	13	19	19	22	26	11	25	14	1.180944912	0% (0.17)
ETHE1_HUMAN (+1)	DEGS1_HUMAN	DEGS1	O15121	Sphingolipid delta(4)-desaturase DES1 (EC 1.14.-.-) (Cell migration-inducing gene	38 kDa	0.2	37	18	19	5	3	7	3	4	4	4	7	1.177818103	0% (0.36)

				15 protein) (Degenerative spermatocyte homolog 1) (Membrane lipid desaturase)															
IN35_HUMAN	B7ZW51_HUMAN		B7ZW51	Uncharacterized protein	28 kDa	0.29	37	18	19	0	0	0	5	0	4	0	11	1.177818103	0% (0.36)
FAH2A_HUMAN	Q86VG2_HUMAN	SFPQ	Q86VG2	Splicing factor proline/glutamine-rich (Polypyrimidine tract binding protein associated)	76 kDa	0.24	78	38	40	12	9	7	11	10	4	12	15	1.176759826	0% (0.26)
PECA1_HUMAN	COF1_HUMAN	CFL1	P23528	Cofilin-1 (18 kDa phosphoprotein) (p18) (Cofilin, non-muscle isoform)	19 kDa	0.15	120	58	61	8	14	14	22	13	24	13	12	1.176451868	0% (0.20)
B4DLC3_HUMAN (+2)	ACATN_HUMAN	SLC33A1	O00400	Acetyl-coenzyme A transporter 1 (AT-1) (Acetyl-CoA transporter 1) (Solute carrier family 33 member 1)	61 kDa	0.19	39	19	20	8	3	4	4	6	4	6	5	1.17494762	0% (0.36)
FA49B_HUMAN	SPON1_HUMAN	SPON1	Q9HCB6	Spondin-1 (F-spondin) (Vascular smooth muscle cell growth-promoting factor)	91 kDa	0.28	40	20	21	4	8	4	4	7	2	5	7	1.172347463	0% (0.35)
OAS2_HUMAN	HSP7C_HUMAN	HSPA8	P11142	Heat shock cognate 71 kDa protein (Heat shock 70 kDa protein 8)	71 kDa	0.17	597	292	305	73	94	69	55	63	80	86	76	1.169986789	95% (0.027)
RM09_HUMAN	B3KXZ4_HUMAN		B3KXZ4	cDNA FLJ46429 fis, clone THYMU3014372, highly similar to DNA replication licensing factor MCM2	91 kDa	0.21	42	21	22	0	11	8	1	12	4	5	0	1.169981182	0% (0.35)
INF2_HUMAN	NDUS8_HUMAN	NDUFS8	O00217	NADH dehydrogenase [ubiquinone] iron-sulfur protein 8, mitochondrial (EC 1.6.5.3) (EC 1.6.99.3) (Complex I-23kD) (CI-23kD) (NADH-ubiquinone oxidoreductase 23 kDa subunit) (TYKY subunit)	24 kDa	0.13	43	21	22	6	4	5	6	5	4	6	8	1.169981182	0% (0.35)
PRS4_HUMAN	SSRD_HUMAN	SSR4	P51571	Translocon-associated protein subunit delta (TRAP-delta) (Signal sequence receptor subunit delta) (SSR-delta)	19 kDa	0.18	43	21	22	5	3	3	10	3	6	7	6	1.169981182	0% (0.35)
Q53HJ4_HUMAN	A3R0T7_HUMAN		A3R0T7	Liver histone H1e	22 kDa	0.12	134	66	69	0	23	17	20	14	17	16	21	1.169721716	0% (0.20)
A8K335_HUMAN (+1)	STT3B_HUMAN	STT3B	Q8TCJ2	Dolichyl-diphosphooligosaccharide--protein glycosyltransferase subunit STT3B (Oligosaccharyl transferase subunit STT3B) (STT3-B) (EC 2.4.99.18) (Source of immunodominant MHC-associated peptides homolog)	94 kDa	0.24	88	44	46	17	8	10	9	10	13	11	12	1.169205314	0% (0.25)
B3KUF6_HUMAN (+1)	DCXR_HUMAN	DCXR	Q7Z4W1	L-xylulose reductase (XR) (EC 1.1.1.10) (Carbonyl reductase II) (Dicarbonyl/L-xylulose reductase) (Kidney dicarbonyl reductase) (kiDCR) (Sperm surface protein P34H)	26 kDa	0.27	46	22	23	1	8	8	5	5	9	4	6	1.167818601	0% (0.35)
B4DEK2_HUMAN (+2)	Q59GL1_HUMAN		Q59GL1	Synaptotagmin binding, cytoplasmic RNA interacting protein variant (Fragment)	60 kDa	0.19	193	95	99	10	33	34	21	25	4	50	21	1.166373963	0% (0.15)
CHP1_HUMAN	HSPB1_HUMAN	HSPB1	P04792	Heat shock protein beta-1 (HspB1) (28 kDa heat shock protein) (Estrogen-regulated 24 kDa protein) (Heat shock 27 kDa protein) (HSP 27) (Stress-responsive protein 27) (SRP27)	23 kDa	0.46	147	71	74	11	17	22	23	18	32	15	11	1.166273775	0% (0.19)
RAB43_HUMAN (+1)	RAB7A_HUMAN	RAB7A	P51149	Ras-related protein Rab-7a	23 kDa	0.27	250	123	128	36	30	30	30	30	23	34	40	1.164966235	0% (0.12)
J3KTE4_HUMAN (+4)	B4DJX1_HUMAN		B4DJX1	cDNA FLJ50978, highly similar to Dihydrolipoyllysine-residue acetyltransferasecomponent of pyruvate dehydrogenase complex, mitochondrial (EC 2.3.1.12)	63 kDa	0.22	100	49	51	19	11	12	10	16	8	11	16	1.164289878	0% (0.25)
C9JLI6_HUMAN (+2)	PGRC1_HUMAN	PGRMC1	O00264	Membrane-associated progesterone receptor component 1 (mPR)	22 kDa	0.25	49	24	25	6	3	6	9	4	9	7	5	1.164007728	0% (0.34)
YMEL1_HUMAN	F8W1R7_HUMAN	MYL6	F8W1R7	Myosin light polypeptide 6	16 kDa	0.082	101	50	52	13	11	10	17	8	13	17	12	1.163422086	0% (0.24)

A4_HUMAN (+2)	SUCB2_HUMAN	SUCLG2	Q96I99	Succinyl-CoA ligase [GDP-forming] subunit beta, mitochondrial (EC 6.2.1.4) (GTP-specific succinyl-CoA synthetase subunit beta) (Succinyl-CoA synthetase beta-G chain) (SCS-betaG)	47 kDa	0.13	105	51	53	17	12	11	11	21	10	11	11	1.162587577	0% (0.24)
GHC1_HUMAN	Q53HH4_HUMAN		Q53HH4	Ras-GTPase-activating protein SH3-domain-binding protein variant (Fragment)	52 kDa	0.11	53	26	27	3	9	9	5	13	1	12	1	1.160756755	0% (0.33)
R4GN98_HUMAN (+1)	VATE1_HUMAN	ATP6V1E1	P36543	V-type proton ATPase subunit E 1 (V-ATPase subunit E 1) (V-ATPase 31 kDa subunit) (p31) (Vacuolar proton pump subunit E 1)	26 kDa	0.38	52	26	27	8	7	7	5	3	7	8	10	1.160756755	0% (0.33)
HTAI2_HUMAN	ECHA_HUMAN	HADHA	P40939	Trifunctional enzyme subunit alpha, mitochondrial (78 kDa gastrin-binding protein) (TP-alpha) [Includes: Long-chain enoyl-CoA hydratase (EC 4.2.1.17); Long chain 3-hydroxyacyl-CoA dehydrogenase (EC 1.1.1.211)]	83 kDa	0.27	461	227	235	82	56	45	42	48	33	55	98	1.159388012	0% (0.056)
Q53FN0_HUMAN	H0YK72_HUMAN	SEC11A	H0YK72	SEC11-like 1 (S. cerevisiae), isoform CRA_a (Signal peptidase complex catalytic subunit SEC11A)	19 kDa	0.19	55	27	28	6	4	7	10	5	8	6	9	1.15930407	0% (0.33)
TALDO_HUMAN	B0YIW6_HUMAN	ARCN1	B0YIW6	Archain 1, isoform CRA_a (Coatomer subunit delta) (Coatomer subunit delta variant 2)	62 kDa	0.28	55	27	28	0	7	9	11	5	13	7	4	1.15930407	0% (0.33)
B4E1Z4_HUMAN	J3KN36_HUMAN	NOMO3	J3KN36	Nodal modulator 3	139 kDa	0.041	114	56	58	17	14	15	10	13	12	13	21	1.158853233	0% (0.24)
B3KMC9_HUMAN (+3)	GPDM_HUMAN	GPD2	P43304	Glycerol-3-phosphate dehydrogenase, mitochondrial (GPD-M) (GPDH-M) (EC 1.1.5.3) (mtGPD)	81 kDa	0.15	232	115	119	38	30	28	20	22	18	30	50	1.158403043	0% (0.14)
C9JVN9_HUMAN	Q96CP5_HUMAN	PMPCB	Q96CP5	PMPCB protein (Fragment)	54 kDa	0.15	58	28	29	6	6	8	8	20	4	1	6	1.157950831	0% (0.33)
TNAP2_HUMAN	ECI1_HUMAN	ECI1	P42126	Enoyl-CoA delta isomerase 1, mitochondrial (EC 5.3.3.8) (3,2-trans-enoyl-CoA isomerase) (Delta(3),Delta(2)-enoyl-CoA isomerase) (D3,D2-enoyl-CoA isomerase) (Dodecenoyl-CoA isomerase)	33 kDa	0.21	57	28	29	6	7	6	9	9	5	8	7	1.157950831	0% (0.33)
RS12_HUMAN	RLA2_HUMAN	RPLP2	P05387	60S acidic ribosomal protein P2 (Renal carcinoma antigen NY-REN-44)	12 kDa	0.32	57	28	29	6	8	6	8	7	8	7	7	1.157950831	0% (0.33)
EIF3M_HUMAN	B4DJA5_HUMAN	RAB5A	B4DJA5	Ras-related protein Rab-5A (cDNA FLJ51867, highly similar to Ras-related protein Rab-5A)	22 kDa	0.28	57	28	29	7	8	7	6	7	7	6	8	1.157950831	0% (0.33)
A8KAH1_HUMAN (+1)	A8K7H3_HUMAN		A8K7H3	cDNA FLJ77670, highly similar to Homo sapiens ribosomal protein S15a (RPS15A), mRNA	15 kDa	0.11	58	28	29	5	6	7	10	9	14	5	3	1.157950831	0% (0.33)
COR1B_HUMAN	B2R5M9_HUMAN		B2R5M9	cDNA, FLJ92537, highly similar to Homo sapiens procollagen-lysine, 2-oxoglutarate 5-dioxygenase (lysine hydroxylase, Ehlers-Danlos syndrome type VI) (PLOD), mRNA	84 kDa	0.12	120	59	61	11	17	17	14	9	14	17	21	1.156910821	0% (0.23)
TIDC1_HUMAN	XRCC5_HUMAN	XRCC5	P13010	X-ray repair cross-complementing protein 5 (EC 3.6.4.-) (86 kDa subunit of Ku antigen) (ATP-dependent DNA helicase 2 subunit 2) (ATP-dependent DNA helicase II 80 kDa subunit) (CTC box-binding factor 85 kDa subunit) (CTC85) (CTCBF) (DNA repair protein XRCC5) (Ku80) (Ku86) (Lupus Ku autoantigen protein p86) (Nuclear factor IV) (Thyroid-lupus autoantigen) (TLAA) (X-ray repair complementing defective	83 kDa	0.15	182	90	93	11	33	23	24	26	26	22	17	1.156626281	0% (0.17)

				repair in Chinese hamster cells 5 (double-strand-break rejoining))															
RL11_HUMAN	B4DPK2_HUMAN		B4DPK2	cDNA FLJ59729, highly similar to DnaJ homolog subfamily C member 11	59 kDa	0.24	61	30	31	12	4	0	14	10	11	4	6	1.155504506	0% (0.32)
ISOC2_HUMAN (+2)	B3KM97_HUMAN		B3KM97	cDNA FLJ10554 fis, clone NT2RP2002385, highly similar to Synaptic glycoprotein SC2	36 kDa	0.16	70	35	36	13	8	7	8	10	10	8	9	1.150571342	0% (0.31)
SPTC1_HUMAN	HNRL2_HUMAN	HNRNPUL2	Q1KMD3	Heterogeneous nuclear ribonucleoprotein U-like protein 2 (Scaffold-attachment factor A2) (SAF-A2)	85 kDa	0.13	151	75	77	14	23	22	16	16	6	42	12	1.149139564	0% (0.21)
A8MUB1_HUMAN (+1)	B2RB06_HUMAN		B2RB06	cDNA, FLJ95242, highly similar to Homo sapiens L-3-hydroxyacyl-Coenzyme A dehydrogenase, short chain (HADHSC), mRNA	34 kDa	0.43	77	38	39	10	8	8	12	11	9	10	9	1.148215789	0% (0.30)
APOOL_HUMAN	TOM70_HUMAN	TOMM70A	O94826	Mitochondrial import receptor subunit TOM70 (Mitochondrial precursor proteins import receptor) (Translocase of outer membrane 70 kDa subunit)	67 kDa	0.28	76	38	39	13	10	9	5	13	4	10	12	1.148215789	0% (0.30)
RM13_HUMAN	VDAC3_HUMAN	VDAC3	Q9Y277	Voltage-dependent anion-selective channel protein 3 (VDAC-3) (hVDAC3) (Outer mitochondrial membrane protein porin 3)	31 kDa	0.32	159	79	81	29	14	15	21	19	27	20	16	1.147682661	0% (0.21)
MCAT_HUMAN	CBX3_HUMAN	CBX3	Q13185	Chromobox protein homolog 3 (HECH) (Heterochromatin protein 1 homolog gamma) (HP1 gamma) (Modifier 2 protein)	21 kDa	0.31	90	44	45	7	13	11	14	12	10	15	8	1.144443755	0% (0.29)
B4DHJ3_HUMAN (+2)	F213A_HUMAN	FAM213A	Q9BRX8	Redox-regulatory protein FAM213A (Peroxiredoxin-like 2 activated in M-CSF stimulated monocytes) (Protein PAMM)	26 kDa	0.13	97	48	49	20	10	9	9	8	14	9	18	1.142441063	0% (0.28)
AL4A1_HUMAN	XRCC6_HUMAN	XRCC6	P12956	X-ray repair cross-complementing protein 6 (EC 3.6.4.-) (EC 4.2.99.-) (5'-deoxyribose-5-phosphate lyase Ku70) (5'-dRP lyase Ku70) (70 kDa subunit of Ku antigen) (ATP-dependent DNA helicase 2 subunit 1) (ATP-dependent DNA helicase II 70 kDa subunit) (CTC box-binding factor 75 kDa subunit) (CTC75) (CTCBF) (DNA repair protein XRCC6) (Lupus Ku autoantigen protein p70) (Ku70) (Thyroid-lupus autoantigen) (TLAA) (X-ray repair complementing defective repair in Chinese hamster cells 6)	70 kDa	0.11	221	110	112	18	31	34	28	33	26	30	22	1.139960737	0% (0.17)
Q53EY4_HUMAN (+1)	TOM40_HUMAN	TOMM40	O96008	Mitochondrial import receptor subunit TOM40 homolog (Protein Haymaker) (Translocase of outer membrane 40 kDa subunit homolog) (p38.5)	38 kDa	0.43	113	56	57	14	10	8	24	11	16	13	18	1.139278127	0% (0.27)
SYCM_HUMAN	CALR_HUMAN	CALR	P27797	Calreticulin (CRP55) (Calregulin) (Endoplasmic reticulum resident protein 60) (ERp60) (HACBP) (grp60)	48 kDa	0.16	267	134	135	34	43	33	25	27	28	42	39	1.128134174	0% (0.17)
DNM1L_HUMAN	A8K9A4_HUMAN		A8K9A4	cDNA FLJ75154, highly similar to Homo sapiens heterogeneous nuclear ribonucleoprotein C (C1/C2), mRNA	34 kDa	0.15	303	151	152	31	40	36	44	44	26	46	38	1.12723922	0% (0.15)
A8K435_HUMAN (+1)	PRP8_HUMAN	PRPF8	Q6P2Q9	Pre-mRNA-processing-splicing factor 8 (220 kDa U5 snRNP-specific protein) (PRP8 homolog) (Splicing factor Prp8) (p220)	274 kDa	0.097	193	96	96	25	27	23	21	23	16	36	21	1.119772555	0% (0.23)

ADA10_HUMAN	VDAC2_HUMAN	VDAC2	P45880	Voltage-dependent anion-selective channel protein 2 (VDAC-2) (hVDAC2) (Outer mitochondrial membrane protein porin 2)	32 kDa	0.068	143	71	71	19	14	17	20	17	21	18	15	1.119729439	0% (0.27)
SORL_HUMAN	NDUS3_HUMAN	NDUFS3	O75489	NADH dehydrogenase [ubiquinone] iron-sulfur protein 3, mitochondrial (EC 1.6.5.3) (EC 1.6.99.3) (Complex I-30kD) (CI-30kD) (NADH-ubiquinone oxidoreductase 30 kDa subunit)	30 kDa	0.056	142	71	71	27	15	11	20	20	15	18	18	1.119729439	0% (0.27)
NAT10_HUMAN	Q6PJ75_HUMAN	ITGB2	Q6PJ75	Integrin beta (Fragment)	84 kDa	0.11	126	63	63	12	25	19	7	0	12	20	31	1.119715648	0% (0.28)
NAKD2_HUMAN	NB5R1_HUMAN	CYB5R1	Q9UHQ9	NADH-cytochrome b5 reductase 1 (b5R.1) (EC 1.6.2.2) (Humb5R2) (NAD(P)H:quinone oxidoreductase type 3 polypeptide A2)	34 kDa	0.15	125	63	63	28	8	8	20	3	21	14	26	1.119715648	0% (0.28)
DDX47_HUMAN (+1)	VPS35_HUMAN	VPS35	Q96QK1	Vacuolar protein sorting-associated protein 35 (hVPS35) (Maternal-embryonic 3) (Vesicle protein sorting 35)	92 kDa	0.14	120	60	60	10	12	20	18	14	18	16	12	1.119710478	0% (0.29)
B2R6X6_HUMAN (+1)	RS2_HUMAN	RPS2	P15880	40S ribosomal protein S2 (40S ribosomal protein S4) (Protein LLRep3)	31 kDa	0.14	108	55	55	10	15	12	18	15	23	8	8	1.119701861	0% (0.30)
D3DUE6_HUMAN (+1)	NDUA9_HUMAN	NDUFA9	Q16795	NADH dehydrogenase [ubiquinone] 1 alpha subcomplex subunit 9, mitochondrial (Complex I-39kD) (CI-39kD) (NADH-ubiquinone oxidoreductase 39 kDa subunit)	43 kDa	0.11	108	54	54	24	10	8	12	14	6	18	16	1.119700138	0% (0.30)
IF2M_HUMAN	ATLA3_HUMAN	ATL3	Q6DD88	Atlastin-3 (EC 3.6.5.-)	61 kDa	0.13	98	49	49	20	9	5	15	7	11	12	18	1.119691523	0% (0.32)
ARP10_HUMAN (+4)	PLOD2_HUMAN	PLOD2	O00469	Procollagen-lysine,2-oxoglutarate 5-dioxygenase 2 (EC 1.14.11.4) (Lysyl hydroxylase 2) (LH2)	85 kDa	0.058	92	46	46	7	19	18	2	9	6	17	15	1.119686354	0% (0.32)
SEP15_HUMAN	ITAM_HUMAN	ITGAM	P11215	Integrin alpha-M (CD11 antigen-like family member B) (CR-3 alpha chain) (Cell surface glycoprotein MAC-1 subunit alpha) (Leukocyte adhesion receptor MO1) (Neutrophil adherence receptor) (CD antigen CD11b)	127 kDa	0.25	90	45	45	9	15	17	4	0	3	18	24	1.119684631	0% (0.33)
SPRY4_HUMAN	AT1B3_HUMAN	ATP1B3	P54709	Sodium/potassium-transporting ATPase subunit beta-3 (Sodium/potassium-dependent ATPase subunit beta-3) (ATPB-3) (CD antigen CD298)	32 kDa	0.28	72	36	36	7	10	10	10	10	9	7	10	1.119669129	0% (0.35)
B2R7B5_HUMAN	PCBP1_HUMAN	PCBP1	Q15365	Poly(rC)-binding protein 1 (Alpha-CP1) (Heterogeneous nuclear ribonucleoprotein E1) (hnRNP E1) (Nucleic acid-binding protein SUB2.3)	37 kDa	0.032	63	31	31	5	9	7	10	10	9	8	5	1.119660518	0% (0.37)
SYNM_HUMAN	SYNC_HUMAN	NARS	O43776	Asparagine--tRNA ligase, cytoplasmic (EC 6.1.1.22) (Asparaginyl-tRNA synthetase) (AsnRS)	63 kDa	0.14	54	27	27	1	8	6	12	10	12	5	1	1.11965363	0% (0.38)
CQ062_HUMAN (+2)	ACTZ_HUMAN	ACTR1A	P61163	Alpha-centractin (Centractin) (ARP1) (Actin-RPV) (Centrosome-associated actin homolog)	43 kDa	0.25	54	27	27	5	10	6	6	4	11	6	6	1.11965363	0% (0.38)
A8K6A7_HUMAN (+3)	C9JNW5_HUMAN	RPL24	C9JNW5	60S ribosomal protein L24 (Ribosomal protein L24, isoform CRA_e)	18 kDa	0.1	52	26	26	5	7	5	9	8	8	4	6	1.119651909	0% (0.39)
D3YTB1_HUMAN (+2)	Q7KZ24_HUMAN		Q7KZ24	Nuclease sensitive element binding protein-1	35 kDa	0.41	51	26	26	1	11	12	2	9	2	8	7	1.119651909	0% (0.39)
B2RAF9_HUMAN (+1)	Q658S9_HUMAN	DKFZp666I134	Q658S9	Putative uncharacterized protein DKFZp666I134 (Fragment)	44 kDa	0.083	50	25	25	6	7	6	5	4	10	5	6	1.119650187	0% (0.39)
B2RCX0_HUMAN	E5RI99_HUMAN	RPL30	E5RI99	60S ribosomal protein L30 (Fragment)	13 kDa	0.17	50	25	25	6	4	6	9	6	7	7	5	1.119650187	0% (0.39)

PXDN_HUMAN	SYG_HUMAN	GARS	P41250	Glycine--tRNA ligase (EC 6.1.1.14) (Diadenosine tetraphosphate synthetase) (AP-4-A synthetase) (Glycyl-tRNA synthetase) (GlyRS)	83 kDa	0.1	50	25	25	4	7	7	7	5	14	3	3	1.1196501 87	0% (0.39)
CTL1_HUMAN	PRS10_HUMAN	PSMC6	P62333	26S protease regulatory subunit 10B (26S proteasome AAA-ATPase subunit RPT4) (Proteasome 26S subunit ATPase 6) (Proteasome subunit p42)	44 kDa	0.11	50	25	25	3	7	7	8	6	11	5	3	1.1196501 87	0% (0.39)
B2RBB2_HUMA N (+2)	B7Z7X8_HUMA N	ATL2	B7Z7X8	Atlastin-2 (cDNA FLJ51152, highly similar to Homo sapiens ADP-ribosylation factor-like 6 interacting protein 2 (ARL6IP2), mRNA)	65 kDa	0.15	47	24	24	10	4	2	8	5	5	7	7	1.1196484 65	0% (0.40)
B3KNC3_HUM AN	Q0VAB1_HUM AN	TIMM50	Q0VAB1	Translocase of inner mitochondrial membrane 50 homolog (S. cerevisiae)	50 kDa	0.12	46	23	23	8	2	6	7	8	1	7	8	1.1196467 44	0% (0.40)
CBPD_HUMAN	DDX18_HUMA N	DDX18	Q9NVP1	ATP-dependent RNA helicase DDX18 (EC 3.6.4.13) (DEAD box protein 18) (Myc-regulated DEAD box protein) (MrDb)	75 kDa	0.075	44	22	22	3	9	4	8	3	3	9	7	1.1196450 22	0% (0.40)
H0YL69_HUMA N (+2)	B0QZ18_HUMA N	CPNE1	B0QZ18	Copine-1 (HCG38213, isoform CRA_b)	60 kDa	0.24	44	22	22	4	7	7	4	10	3	5	4	1.1196450 22	0% (0.40)
J3KQ48_HUMA N (+2)	1433F_HUMAN	YWHAH	Q04917	14-3-3 protein eta (Protein AS1)	28 kDa	0.21	42	21	21	0	6	6	5	0	4	5	0	1.1196433	0% (0.41)
IMA5_HUMAN (+1)	SF3A1_HUMAN	SF3A1	Q15459	Splicing factor 3A subunit 1 (SF3a120) (Spliceosome-associated protein 114) (SAP 114)	89 kDa	0.16	40	20	20	1	5	11	3	2	0	11	7	1.1196415 79	0% (0.41)
CECR5_HUMA N	NSMA3_HUMA N	SMPD4	Q9NXE4	Sphingomyelin phosphodiesterase 4 (EC 3.1.4.12) (Neutral sphingomyelinase 3) (nSMase-3) (nSMase3) (Neutral sphingomyelinase III)	93 kDa	0.14	41	20	20	6	5	2	7	6	5	3	6	1.1196415 79	0% (0.41)
A8K6V6_HUMA N (+4)	STXB3_HUMA N	STXBP3	O00186	Syntaxin-binding protein 3 (Platelet Sec1 protein) (PSP) (Protein unc-18 homolog 3) (Unc18-3) (Protein unc-18 homolog C) (Unc-18C)	68 kDa	0.12	39	20	20	5	5	5	5	6	2	6	7	1.1196415 79	0% (0.41)
I3L397_HUMAN (+2)	MBOA7_HUMA N	MBOAT7	Q96N66	Lysophospholipid acyltransferase 7 (LPLAT 7) (EC 2.3.1.-) (1-acylglycerophosphatidylinositol O-acyltransferase) (EC 2.3.1.n4) (Bladder and breast carcinoma-overexpressed gene 1 protein) (Leukocyte receptor cluster member 4) (Lysophosphatidylinositol acyltransferase) (LPIAT) (Lyso-PI acyltransferase) (Membrane-bound O-acyltransferase domain-containing protein 7) (O-acyltransferase domain-containing protein 7) (h-mboa-7)	53 kDa	0.17	37	19	19	4	4	4	7	4	5	4	6	1.1196398 57	0% (0.42)
RB12B_HUMAN	B4DRV2_HUM AN		B4DRV2	cDNA FLJ53646, highly similar to Succinyl-CoA ligase (ADP-forming) beta-chain, mitochondrial (EC 6.2.1.5)	44 kDa	0.063	36	18	18	7	3	5	4	10	3	1	4	1.1196381 36	0% (0.43)
I1SRC5_HUMA N	A8K529_HUMA N		A8K529	cDNA FLJ76184, highly similar to Homo sapiens NOL1/NOP2/Sun domain family, member 2 (NSUN2), mRNA	86 kDa	0.14	36	18	18	0	7	9	2	8	3	5	2	1.1196381 36	0% (0.43)
STING_HUMAN	B7ZL00_HUMA N	SEC31A	B7ZL00	Protein transport protein Sec31A (SEC31A protein)	128 kDa	0.14	34	17	17	7	4	2	4	1	8	2	6	1.1196364 15	0% (0.43)
B4DU42_HUMA N (+1)	RL3_HUMAN	RPL3	P39023	60S ribosomal protein L3 (HIV-1 TAR RNA-binding protein B) (TARBP-B)	46 kDa	0.09	34	17	17	0	3	7	7	8	3	3	3	1.1196364 15	0% (0.43)
PGLT1_HUMAN	E7ENA9_HUMA N	NCSTN	E7ENA9	Nicastrin	50 kDa	0.17	34	17	17	7	4	4	3	5	4	3	5	1.1196364 15	0% (0.43)
DHRS3_HUMA N (+1)	KCY_HUMAN	CMPK1	P30085	UMP-CMP kinase (EC 2.7.4.14) (Deoxycytidylate	22 kDa	0.2	34	17	17	0	5	6	6	8	5	4	0	1.1196364 15	0% (0.43)

				kinase) (CK) (dCMP kinase) (Nucleoside-diphosphate kinase) (EC 2.7.4.6) (Uridine monophosphate/cytidine monophosphate kinase) (UMP/CMP kinase) (UMP/CMPK)														
A8K454_HUMA N	B7ZKK7_HUMA N	EIF2AK2	B7ZKK7	EIF2AK2 protein	62 kDa	0.1	34	17	17	0	2	3	12	6	6	3	2	1.1196364 15	0% (0.43)
ACL6A_HUMA N (+1)	PTCD3_HUMA N	PTCD3	Q96EY7	Pentatricopeptide repeat domain-containing protein 3, mitochondrial (28S ribosomal protein S39, mitochondrial) (MRP-S39) (Transformation-related gene 15 protein) (TRG-15)	79 kDa	0.12	32	16	16	8	3	1	4	8	1	2	5	1.1196346 93	0% (0.44)
XPP1_HUMAN	Q53HJ4_HUMA N		Q53HJ4	Minichromosome maintenance protein 3 variant (Fragment)	91 kDa	0.095	30	15	15	0	9	6	0	7	4	4	0	1.1196329 72	0% (0.44)
PSMD8_HUMA N	IMA3_HUMAN	KPNA4	O00629	Importin subunit alpha-3 (Importin alpha Q1) (Qip1) (Karyopherin subunit alpha-4)	58 kDa	0.15	30	15	15	2	4	6	3	5	2	5	3	1.1196329 72	0% (0.44)
MNDA_HUMA N	CAZA2_HUMA N	CAPZA2	P47755	F-actin-capping protein subunit alpha-2 (CapZ alpha-2)	33 kDa	0.23	31	15	15	3	4	5	3	4	2	4	5	1.1196329 72	0% (0.44)
A8K6A5_HUMA N (+2)	Q59GW5_HUM AN		Q59GW5	Tripartite motif-containing 25 variant (Fragment)	72 kDa	0.078	28	14	14	1	4	5	4	2	10	2	0	1.1196312 51	0% (0.45)
MYO1B_HUMA N	CSK22_HUMAN	CSNK2A2	P19784	Casein kinase II subunit alpha' (CK II alpha') (EC 2.7.11.1)	41 kDa	0.12	28	14	14	1	5	4	4	4	1	9	1	1.1196312 51	0% (0.45)
J3QRS3_HUMA N	RM19_HUMAN	MRPL19	P49406	39S ribosomal protein L19, mitochondrial (L19mt) (MRP-L19) (39S ribosomal protein L15, mitochondrial) (L15mt) (MRP-L15)	34 kDa	0.18	26	13	13	6	2	3	2	5	0	3	5	1.1196295 3	0% (0.46)
IMA3_HUMAN	F5H5P2_HUMA N	BCKDHA	F5H5P2	2-oxoisovalerate dehydrogenase subunit alpha, mitochondrial (Uncharacterized protein)	54 kDa	0.12	26	13	13	8	1	2	2	3	4	2	4	1.1196295 3	0% (0.46)
PSDE_HUMAN	G3V2S6_HUMA N	ATP6V1D	G3V2S6	V-type proton ATPase subunit D	22 kDa	0.16	26	13	13	4	3	4	2	0	2	3	8	1.1196295 3	0% (0.46)
B2R739_HUMA N	C9JJ19_HUMAN	MRPS34	C9JJ19	28S ribosomal protein S34, mitochondrial	26 kDa	0.33	24	12	12	3	3	3	3	3	3	2	4	1.1196278 09	0% (0.47)
D0UU70_HUMA N (+4)	AGAL_HUMAN	GLA	P06280	Alpha-galactosidase A (EC 3.2.1.22) (Alpha-D-galactosidase A) (Alpha-D-galactoside galactohydrolase) (Melibiase) (Agalsidase)	49 kDa	0.06	24	12	12	1	6	5	0	1	2	1	8	1.1196278 09	0% (0.47)
MARH5_HUMA N	MK01_HUMAN	MAPK1	P28482	Mitogen-activated protein kinase 1 (MAP kinase 1) (MAPK 1) (EC 2.7.11.24) (ERT1) (Extracellular signal-regulated kinase 2) (ERK-2) (MAP kinase isoform p42) (p42-MAPK) (Mitogen-activated protein kinase 2) (MAP kinase 2) (MAPK 2)	41 kDa	0.29	23	11	11	0	3	1	7	0	7	4	0	1.1196260 88	0% (0.47)
NAGK_HUMAN	HINT2_HUMAN	HINT2	Q9BX68	Histidine triad nucleotide-binding protein 2, mitochondrial (HINT-2) (EC 3.-.-.-) (HINT-3) (HIT-17kDa) (PKCI-1-related HIT protein)	17 kDa	0.3	22	11	11	2	5	2	2	2	2	2	4	1.1196260 88	0% (0.47)
STX7_HUMAN	ALG2_HUMAN	ALG2	Q9H553	Alpha-1,3/1,6-mannosyltransferase ALG2 (EC 2.4.1.132) (EC 2.4.1.257) (Asparagine-linked glycosylation protein 2 homolog) (GDP-Man:Man(1)GlcNAc(2)-PP-Dol alpha-1,3-mannosyltransferase) (GDP-Man:Man(1)GlcNAc(2)-PP-dolichol mannosyltransferase) (GDP-Man:Man(2)GlcNAc(2)-PP-Dol alpha-1,6-mannosyltransferase)	47 kDa	0.16	23	11	11	5	2	3	1	1	0	4	6	1.1196260 88	0% (0.47)
RASL1_HUMA N	DNJA2_HUMA N	DNAJA2	O60884	DnaJ homolog subfamily A member 2 (Cell cycle progression restoration gene 3 protein) (Dnj3) (Dj3) (HIRA-	46 kDa	0.17	19	10	10	2	3	1	4	5	4	1	0	1.1196243 67	0% (0.48)

				interacting protein 4) (Renal carcinoma antigen NY-REN-14)														
NDUAA_HUMAN	ICT1_HUMAN	ICT1	Q14197	Peptidyl-tRNA hydrolase ICT1, mitochondrial (EC 3.1.1.29) (39S ribosomal protein L58, mitochondrial) (MRP-L58) (Digestion substraction 1) (DS-1) (Immature colon carcinoma transcript 1 protein)	24 kDa	0.12	20	10	10	3	5	1	1	4	2	2	2	1.119624367	0% (0.48)
RAB5B_HUMAN	IMA4_HUMAN	KPNA3	O00505	Importin subunit alpha-4 (Importin alpha Q2) (Qip2) (Karyopherin subunit alpha-3) (SRP1-gamma)	58 kDa	0.33	20	10	10	0	3	5	0	4	0	3	2	1.119624367	0% (0.48)
CB047_HUMAN	A8K968_HUMAN		A8K968	cDNA FLJ77757	85 kDa	0.19	18	9	9	0	5	4	0	0	1	2	6	1.119622646	0% (0.49)
RAP1B_HUMAN	ERH_HUMAN	ERH	P84090	Enhancer of rudimentary homolog	12 kDa	0.42	18	9	9	1	1	4	3	2	1	4	2	1.119622646	0% (0.49)
A8K0T9_HUMAN (+1)	LPCT4_HUMAN	LPCAT4	Q643R3	Lysophospholipid acyltransferase LPCAT4 (1-acylglycerol-3-phosphate O-acyltransferase 7) (1-AGP acyltransferase 7) (1-AGPAT 7) (1-acylglycerophosphocholine O-acyltransferase) (EC 2.3.1.23) (1-acylglycerophosphoserine O-acyltransferase) (EC 2.3.1.n6) (1-alkenylglycerophosphoethanolamine O-acyltransferase) (EC 2.3.1.121) (1-alkylglycerophosphocholine O-acetyltransferase) (EC 2.3.1.67) (Acyltransferase-like 3) (Lysophosphatidylcholine acyltransferase 4) (Lysophosphatidylethanolamine acyltransferase 2) (EC 2.3.1.n7) (Plasmalogen synthase)	57 kDa	0.24	18	9	9	5	1	2	1	0	1	3	5	1.119622646	0% (0.49)
OXSR1_HUMAN	NDUA5_HUMAN	NDUFA5	Q16718	NADH dehydrogenase [ubiquinone] 1 alpha subcomplex subunit 5 (Complex I subunit B13) (Complex I-13kD-B) (CI-13kD-B) (NADH-ubiquinone oxidoreductase 13 kDa-B subunit)	13 kDa	0.14	18	9	9	5	1	1	2	3	2	2	2	1.119622646	0% (0.49)
B7Z1V4_HUMAN (+2)	PGAM5_HUMAN	PGAM5	Q96HS1	Serine/threonine-protein phosphatase PGAM5, mitochondrial (EC 3.1.3.16) (Bcl-XL-binding protein v68) (Phosphoglycerate mutase family member 5)	32 kDa	0.12	16	8	8	4	3	1	0	7	0	1	0	1.119620925	0% (0.50)
RUXE_HUMAN	B2RCZ4_HUMAN		B2RCZ4	cDNA, FLJ96384, highly similar to Homo sapiens protein kinase C, iota (PRKCI), mRNA	67 kDa	0.29	16	8	8	0	3	2	3	1	2	1	4	1.119620925	0% (0.50)
RS20_HUMAN	LMF2_HUMAN	LMF2	Q9BU23	Lipase maturation factor 2 (Transmembrane protein 112B) (Transmembrane protein 153)	80 kDa	0.19	16	8	8	4	2	2	0	2	1	4	1	1.119620925	0% (0.50)
B4E2Q0_HUMAN	B7Z7P8_HUMAN	ETF1	B7Z7P8	Eukaryotic peptide chain release factor subunit 1 (cDNA FLJ56175, highly similar to Eukaryotic peptide chain release factor subunit1)	47 kDa	0.082	17	8	8	1	2	0	6	3	2	2	1	1.119620925	0% (0.50)
PSME3_HUMAN	DPP3_HUMAN	DPP3	Q9NY33	Dipeptidyl peptidase 3 (EC 3.4.14.4) (Dipeptidyl aminopeptidase III) (Dipeptidyl arylamidase III) (Dipeptidyl peptidase III) (DPP III) (Enkephalinase B)	83 kDa	0.2	16	8	8	2	0	2	4	2	0	5	1	1.119620925	0% (0.50)
SON_HUMAN	H3BSH7_HUMAN	CIRH1A	H3BSH7	Cirhin (Fragment)	78 kDa	0.035	16	8	8	1	3	2	2	5	0	1	2	1.119620925	0% (0.50)
AAAT_HUMAN (+2)	EMC7_HUMAN	EMC7	Q9NPA0	ER membrane protein complex subunit 7	26 kDa	0.11	15	8	8	2	2	2	1	0	4	3	2	1.119620925	0% (0.50)

Q53GD1_HUMAN	B3KQN7_HUMAN		B3KQN7	cDNA FLJ90789 fis, clone THYRO1001523, highly similar to G-protein coupled receptor 56	77 kDa	0.25	16	8	8	3	4	1	0	3	0	3	2	1.119620925	0% (0.50)
Q53GS0_HUMAN	B3KSE0_HUMAN		B3KSE0	cDNA FLJ36069 fis, clone TESTI2019406, highly similar to HEME OXYGENASE 2 (EC 1.14.99.3)	36 kDa	0.13	16	8	8	6	0	1	1	1	1	2	4	1.119620925	0% (0.50)
HPCL1_HUMAN	ATAD1_HUMAN	ATAD1	Q8NBU5	ATPase family AAA domain-containing protein 1 (EC 3.6.1.3) (Thorase)	41 kDa	0.38	16	8	8	2	1	1	4	2	1	2	3	1.119620925	0% (0.50)
PERM_HUMAN	NFU1_HUMAN	NFU1	Q9UMS0	NFU1 iron-sulfur cluster scaffold homolog, mitochondrial (HIRA-interacting protein 5)	28 kDa	0.14	16	8	8	3	1	2	2	4	1	1	2	1.119620925	0% (0.50)
CLPP_HUMAN	PRAF1_HUMAN	RABAC1	Q9UI14	Prenylated Rab acceptor protein 1 (PRA1 family protein 1)	21 kDa	0.17	15	7	7	1	4	0	2	1	2	2	2	1.119619204	0% (0.52)
B4E2S7_HUMAN	NOP9_HUMAN	NOP9	Q86U38	Nucleolar protein 9	69 kDa	0.08	14	7	7	0	1	2	4	2	2	2	1	1.119619204	0% (0.52)
CH3L1_HUMAN	B4DZJ7_HUMAN		B4DZJ7	Transcription elongation factor SPT5	118 kDa	0.33	14	7	7	0	3	2	2	3	0	1	3	1.119619204	0% (0.52)
B2RBD5_HUMAN (+1)	B3KQ18_HUMAN		B3KQ18	cDNA FLJ32632 fis, clone SYNOV1000190, highly similar to Alpha-1,3-mannosyl-glycoprotein2-beta-N-acetylglucosaminyltransferase (EC 2.4.1.101)	51 kDa	0.41	14	7	7	1	1	1	4	2	0	1	4	1.119619204	0% (0.52)
CD47_HUMAN	SNAG_HUMAN	NAPG	Q99747	Gamma-soluble NSF attachment protein (SNAP-gamma) (N-ethylmaleimide-sensitive factor attachment protein gamma)	35 kDa	0.084	14	7	7	2	2	2	1	3	0	2	2	1.119619204	0% (0.52)
E7EQT4_HUMAN	CSK2B_HUMAN	CSNK2B	P67870	Casein kinase II subunit beta (CK II beta) (Phosvitin) (Protein G5a)	25 kDa	0.048	14	7	7	0	2	2	3	2	3	2	0	1.119619204	0% (0.52)
ARL1_HUMAN (+1)	TMED5_HUMAN	TMED5	Q9Y3A6	Transmembrane emp24 domain-containing protein 5 (p24 family protein gamma-2) (p24gamma2) (p28)	26 kDa	0.28	14	7	7	2	1	0	4	1	4	1	1	1.119619204	0% (0.52)
CATZ_HUMAN (+1)	TMED1_HUMAN	TMED1	Q13445	Transmembrane emp24 domain-containing protein 1 (Interleukin-1 receptor-like 1 ligand) (Putative T1/ST2 receptor-binding protein) (p24 family protein gamma-1) (Tp24) (p24gamma1)	25 kDa	0.13	14	7	7	2	1	1	3	2	2	1	2	1.119619204	0% (0.52)
CSK22_HUMAN	B7ZA00_HUMAN		B7ZA00	cDNA, FLJ79014, highly similar to cAMP-dependent protein kinase, beta-catalytic subunit (EC 2.7.11.11)	36 kDa	0.2	12	6	6	0	1	2	3	1	4	2	0	1.119617483	0% (0.53)
A8K566_HUMAN (+4)	RBM25_HUMAN	RBM25	P49756	RNA-binding protein 25 (Arg/Glu/Asp-rich protein of 120 kDa) (RED120) (Protein S164) (RNA-binding motif protein 25) (RNA-binding region-containing protein 7)	100 kDa	0.11	10	5	5	1	3	1	0	1	0	3	1	1.119615762	0% (0.55)
GNAQ_HUMAN	ACAD8_HUMAN	ACAD8	Q9UKU7	Isobutyryl-CoA dehydrogenase, mitochondrial (EC 1.3.99.-) (Activator-recruited cofactor 42 kDa component) (ARC42) (Acyl-CoA dehydrogenase family member 8) (ACAD-8)	45 kDa	0.18	9	5	5	1	1	2	1	3	1	1	0	1.119615762	0% (0.55)
5NTD_HUMAN (+3)	PSB2_HUMAN	PSMB2	P49721	Proteasome subunit beta type-2 (EC 3.4.25.1) (Macropain subunit C7-I) (Multicatalytic endopeptidase complex subunit C7-I) (Proteasome component C7-I)	23 kDa	0.2	10	5	5	0	2	2	1	1	1	1	2	1.119615762	0% (0.55)
B3KNN9_HUMAN (+1)	CPSF2_HUMAN	CPSF2	Q9P2I0	Cleavage and polyadenylation specificity factor subunit 2 (Cleavage and polyadenylation specificity factor 100 kDa subunit) (CPSF 100 kDa subunit)	88 kDa	0.15	10	5	5	1	1	1	2	0	1	3	1	1.119615762	0% (0.55)
J3KP14_HUMAN (+1)	S35B2_HUMAN	SLC35B2	Q8TB61	Adenosine 3'-phospho 5'-phosphosulfate transporter 1	48 kDa	0.0039	10	5	5	3	1	0	1	0	5	0	0	1.119615762	0% (0.55)

				(PAPS transporter 1) (Putative MAPK-activating protein PM15) (Putative NF-kappa-B-activating protein 48) (Solute carrier family 35 member B2)															
LBP_HUMAN	TMLH_HUMAN	TMLHE	Q9NVH6	Trimethyllysine dioxygenase, mitochondrial (EC 1.14.11.8) (Epsilon-trimethyllysine 2-oxoglutarate dioxygenase) (Epsilon-trimethyllysine hydroxylase) (TML hydroxylase) (TML-alpha-ketoglutarate dioxygenase) (TML dioxygenase) (TMLD)	50 kDa	0.13	10	5	5	0	0	0	5	6	0	0	0	1.1196157 62	0% (0.55)
TM214_HUMAN	B2RAG5_HUM AN		B2RAG5	cDNA, FLJ94903, highly similar to Homo sapiens glyceronephosphate O-acyltransferase (GNPAT), mRNA	77 kDa	0.11	10	5	5	3	0	0	2	0	2	0	3	1.1196157 62	0% (0.55)
B3KMB8_HUM AN (+1)	H0UI80_HUMA N	TH1L	H0UI80	Negative elongation factor C/D (TH1-like (Drosophila), isoform CRA_b)	67 kDa	0.063	10	5	5	0	0	2	2	2	1	2	0	1.1196157 62	0% (0.55)
CRNL1_HUMA N (+1)	B4DZP4_HUMA N	DYNC1LI2	B4DZP4	Cytoplasmic dynein 1 light intermediate chain 2 (cDNA FLJ52924, highly similar to Cytoplasmic dynein 1 light intermediate chain 2)	45 kDa	0.13	10	5	5	5	0	0	0	1	4	0	0	1.1196157 62	0% (0.55)
B7Z6B3_HUMA N (+1)	RM24_HUMAN	MRPL24	Q96A35	39S ribosomal protein L24, mitochondrial (L24mt) (MRP-L24)	25 kDa	0.25	10	5	5	3	1	1	0	3	0	1	1	1.1196157 62	0% (0.55)
D3DQ69_HUMA N (+1)	B4DHE8_HUMA N	MSI2	B4DHE8	RNA-binding protein Musashi homolog 2 (cDNA FLJ56904, highly similar to RNA-binding protein Musashi homolog 2)	35 kDa	0.036	10	5	5	1	2	1	1	0	0	4	1	1.1196157 62	0% (0.55)
CISD1_HUMAN	B5BU81_HUMA N	YKT6	B5BU81	YKT6 v-SNARE protein	22 kDa	0.34	10	5	5	1	1	0	3	4	1	0	0	1.1196157 62	0% (0.55)
FCL_HUMAN	Q8WY67_HUM AN	RAB24	Q8WY67	PP4748 (RAB24, member RAS oncogene family, isoform CRA_d)	21 kDa	0.18	10	5	5	4	0	0	1	0	2	1	2	1.1196157 62	0% (0.55)
PAFA2_HUMA N	CY24A_HUMA N	CYBA	P13498	Cytochrome b-245 light chain (Cytochrome b(558) alpha chain) (Cytochrome b558 subunit alpha) (Neutrophil cytochrome b 22 kDa polypeptide) (Superoxide-generating NADPH oxidase light chain subunit) (p22 phagocyte B-cytochrome) (p22-phox) (p22phox)	21 kDa	0.24	8	4	4	0	2	1	1	0	1	1	2	1.1196140 42	0% (0.57)
DCMC_HUMAN	B0QYK0_HUM AN	EWSR1	B0QYK0	RNA-binding protein EWS	65 kDa	0.19	8	4	4	2	0	1	1	2	0	1	1	1.1196140 42	0% (0.57)
B7Z3E3_HUMA N (+2)	COX5B_HUMA N	COX5B	P10606	Cytochrome c oxidase subunit 5B, mitochondrial (Cytochrome c oxidase polypeptide Vb)	14 kDa	0.045	8	4	4	3	0	0	1	2	2	0	0	1.1196140 42	0% (0.57)
DX39B_HUMA N (+1)	B4DP99_HUMA N		B4DP99	cDNA FLJ51496, highly similar to Tyrosine-protein kinase CSK (EC 2.7.10.2)	45 kDa	0.29	8	4	4	0	0	2	2	0	4	0	0	1.1196140 42	0% (0.57)
RM19_HUMAN	PARP4_HUMAN	PARP4	Q9UKK3	Poly [ADP-ribose] polymerase 4 (PARP-4) (EC 2.4.2.30) (193 kDa vault protein) (ADP-ribosyltransferase diphtheria toxin-like 4) (ARTD4) (PARP-related/IalphaI-related H5/proline-rich) (PH5P) (Vault poly(ADP-ribose) polymerase) (VPARP)	193 kDa	0.15	8	4	4	1	0	2	1	0	0	1	3	1.1196140 42	0% (0.57)
B3KN29_HUMA N (+1)	Q5SWX3_HUM AN	CAMK2G	Q5SWX 3	Calcium/calmodulin-dependent protein kinase (CaM kinase) II gamma, isoform CRA_n (Calcium/calmodulin-dependent protein kinase type II subunit gamma)	58 kDa	0.16	8	4	4	1	1	1	1	2	1	1	0	1.1196140 42	0% (0.57)
ARK72_HUMA N (+1)	SRP72_HUMAN	SRP72	O76094	Signal recognition particle subunit SRP72 (SRP72) (Signal recognition particle 72 kDa protein)	75 kDa	0.19	8	4	4	0	2	1	1	0	4	0	0	1.1196140 42	0% (0.57)

B7Z6F7_HUMAN (+1)	RDH11_HUMAN	RDH11	Q8TC12	Retinol dehydrogenase 11 (EC 1.1.1.300) (Androgen-regulated short-chain dehydrogenase/reductase 1) (HCV core-binding protein HCBP12) (Prostate short-chain dehydrogenase/reductase 1) (Retinal reductase 1) (RalR1)	35 kDa	0.078	8	4	4	4	0	0	0	0	0	1	3	1.119614042	0% (0.57)
IMPA3_HUMAN	NELFB_HUMAN	NELFB	Q8WX92	Negative elongation factor B (NELF-B) (Cofactor of BRCA1)	66 kDa	0.11	8	4	4	0	2	1	1	1	1	2	0	1.119614042	0% (0.57)
SPRE_HUMAN	B4DR61_HUMAN	SEC61A1	B4DR61	Protein transport protein Sec61 subunit alpha isoform 1 (cDNA FLJ59739, highly similar to Protein transport protein Sec61 subunit alpha isoform 1)	53 kDa	0.39	8	4	4	2	1	0	1	1	1	1	1	1.119614042	0% (0.57)
CADH1_HUMAN (+2)	DJB12_HUMAN	DNAJB12	Q9NXW2	DnaJ homolog subfamily B member 12	42 kDa	0.088	8	4	4	1	0	1	2	0	1	1	2	1.119614042	0% (0.57)
PPT1_HUMAN	A6XND5_HUMAN		A6XND5	Ribonuclease T2	36 kDa	0.14	8	4	4	0	1	2	2	0	0	1	3	1.119614042	0% (0.57)
RASN_HUMAN	NIBAN_HUMAN	FAM129A	Q9BZQ8	Protein Niban (Cell growth-inhibiting gene 39 protein) (Protein FAM129A)	103 kDa	0.25	8	4	4	2	0	2	0	0	2	1	1	1.119614042	0% (0.57)
CYTB_HUMAN	CP4FB_HUMAN	CYP4F11	Q9HBI6	Cytochrome P450 4F11 (EC 1.14.14.1) (CYPIVF11)	60 kDa	0.34	8	4	4	4	0	0	0	0	0	0	4	1.119614042	0% (0.57)
HNRH2_HUMAN	C9JEJ2_HUMAN	PCYT1A	C9JEJ2	Choline-phosphate cytidylyltransferase A	43 kDa	0.2	8	4	4	0	1	1	2	2	1	1	0	1.119614042	0% (0.57)
RL23A_HUMAN	DCD_HUMAN	DCD	P81605	Dermcidin (EC 3.4.-.-) (Preproteolysin) [Cleaved into: Survival-promoting peptide; DCD-1]	11 kDa	0.37	8	4	4	0	1	2	1	0	0	0	4	1.119614042	0% (0.57)
B4DZ67_HUMAN (+1)	I3VM54_HUMAN	KDM2A	I3VM54	N-terminus deleted lysine-specific demethylase 2A	131 kDa	0.12	6	3	3	0	2	1	0	0	0	2	1	1.119612321	0% (0.60)
VASP_HUMAN	CLCC1_HUMAN	CLCC1	Q96S66	Chloride channel CLIC-like protein 1 (Mid-1-related chloride channel protein 1)	62 kDa	0.22	5	3	3	0	1	1	2	1	0	1	1	1.119612321	0% (0.60)
PIGT_HUMAN	LSM4_HUMAN	LSM4	Q9Y4Z0	U6 snRNA-associated Sm-like protein LSm4 (Glycine-rich protein) (GRP)	15 kDa	0.13	6	3	3	0	0	1	2	0	0	1	2	1.119612321	0% (0.60)
MCCA_HUMAN	B4DSD7_HUMAN		B4DSD7	cDNA FLJ55863, highly similar to Serine/threonine-protein phosphatase 2A 56 kDa regulatory subunit delta isoform	65 kDa	0.16	6	3	3	0	2	1	0	1	1	1	0	1.119612321	0% (0.60)
K7ES61_HUMAN (+1)	Q53FA5_HUMAN		Q53FA5	Protein translocation complex beta variant (Fragment)	10 kDa	0.25	6	3	3	1	1	0	1	0	1	0	2	1.119612321	0% (0.60)
PPA5_HUMAN	B9EIS5_HUMAN	PCM1	B9EIS5	PCM1 protein	228 kDa	0.16	6	3	3	2	0	1	0	0	2	0	1	1.119612321	0% (0.60)
SPTA1_HUMAN	NTF2_HUMAN	NUTF2	P61970	Nuclear transport factor 2 (NTF-2) (Placental protein 15) (PP15)	14 kDa	0.06	6	3	3	0	0	0	3	2	1	0	0	1.119612321	0% (0.60)
COX6C_HUMAN	PAXB1_HUMAN	PAXBP1	Q9Y5B6	PAX3- and PAX7-binding protein 1 (GC-rich sequence DNA-binding factor 1)	105 kDa	0.37	6	3	3	0	1	0	2	1	0	2	0	1.119612321	0% (0.60)
AP1B1_HUMAN	ATG7_HUMAN	ATG7	O95352	Ubiquitin-like modifier-activating enzyme ATG7 (ATG12-activating enzyme E1 ATG7) (Autophagy-related protein 7) (APG7-like) (hAGP7) (Ubiquitin-activating enzyme E1-like protein)	78 kDa	0.18	6	3	3	0	1	2	0	0	3	0	0	1.119612321	0% (0.60)
A8K2Y2_HUMAN (+1)	GLCE_HUMAN	GLCE	O94923	D-glucuronyl C5-epimerase (EC 5.1.3.17) (Heparan sulfate C5-epimerase) (Hsepi) (Heparin/heparan sulfate:glucuronic acid C5-epimerase) (Heparosan-N-sulfate-glucuronate 5-epimerase)	70 kDa	0.21	6	3	3	3	0	0	1	0	2	0	1	1.119612321	0% (0.60)
CN37_HUMAN	6PGL_HUMAN	PGLS	O95336	6-phosphogluconolactonase (6PGL) (EC 3.1.1.31)	28 kDa	0.16	6	3	3	2	0	0	1	0	3	0	0	1.119612321	0% (0.60)
ANXA9_HUMAN	SNX27_HUMAN	SNX27	Q96L92	Sorting nexin-27	61 kDa	0.44	6	3	3	0	0	3	0	2	0	1	0	1.119612321	0% (0.60)
ALG3_HUMAN	B4DPG9_HUMAN		B4DPG9	cDNA FLJ59630, highly similar to Growth hormone-inducible transmembrane protein	38 kDa	0.046	6	3	3	2	0	0	1	1	1	0	1	1.119612321	0% (0.60)

CLUS_HUMAN	PLCC_HUMAN	AGPAT3	Q9NRZ7	1-acyl-sn-glycerol-3-phosphate acyltransferase gamma (EC 2.3.1.51) (1-acylglycerol-3-phosphate O-acyltransferase 3) (1-AGP acyltransferase 3) (1-AGPAT 3) (Lysophosphatidic acid acyltransferase gamma) (LPAAT-gamma)	43 kDa	0.15	6	3	3	2	0	1	0	1	0	1	1	1.119612321	0% (0.60)
RMD1_HUMAN	ABHD6_HUMAN	ABHD6	Q9BV23	Monoacylglycerol lipase ABHD6 (EC 3.1.1.23) (2-arachidonoylglycerol hydrolase) (Abhydrolase domain-containing protein 6)	38 kDa	0.22	6	3	3	1	1	1	0	0	1	0	2	1.119612321	0% (0.60)
B2R7G6_HUMAN (+1)	E7EQ01_HUMAN	CASP8	E7EQ01	Caspase-8 subunit p10 (Fragment)	14 kDa	0.097	6	3	3	0	1	1	1	0	3	0	0	1.119612321	0% (0.60)
CCAR1_HUMAN	B4DEF8_HUMAN		B4DEF8	cDNA FLJ61100, highly similar to 39S ribosomal protein L45, mitochondrial	38 kDa	0.082	6	3	3	2	1	0	0	1	0	1	1	1.119612321	0% (0.60)
AP3B1_HUMAN (+1)	Q96AD0_HUMAN	TRIM47	Q96AD0	TRIM47 protein (Tripartite motif-containing 47, isoform CRA_c) (Tripartite motif-containing protein 47)	44 kDa	0.052	6	3	3	3	0	0	0	0	1	2	0	1.119612321	0% (0.60)
TMOD1_HUMAN	B4DT01_HUMAN		B4DT01	cDNA FLJ60347, highly similar to Homo sapiens hydroxyacylglutathione hydrolase (HAGH), transcript variant 1, mRNA	33 kDa	0.16	6	3	3	2	0	0	1	0	1	0	2	1.119612321	0% (0.60)
FIS1_HUMAN	B3KUB8_HUMAN		B3KUB8	cDNA FLJ39539 fis, clone PUAEN2008228, highly similar to Platelet glycoprotein 4	53 kDa	0.35	6	3	3	1	1	0	1	0	0	2	1	1.119612321	0% (0.60)
RT35_HUMAN	E7EMW7_HUMAN	UBR5	E7EMW7	E3 ubiquitin-protein ligase UBR5	309 kDa	0.071	4	2	2	0	0	1	1	0	0	2	0	1.1196106	0% (0.64)
A8K2Q6_HUMAN (+1)	B4DG57_HUMAN		B4DG57	cDNA FLJ61230, highly similar to PHD finger protein 14	69 kDa	0.15	4	2	2	1	0	0	1	2	0	0	0	1.1196106	0% (0.64)
Q504R6_HUMAN	PSA1_HUMAN	PSMA1	P25786	Proteasome subunit alpha type-1 (EC 3.4.25.1) (30 kDa prosomal protein) (PROS-30) (Macropain subunit C2) (Multicatalytic endopeptidase complex subunit C2) (Proteasome component C2) (Proteasome nu chain)	30 kDa	0.25	4	2	2	0	0	0	2	0	1	2	0	1.1196106	0% (0.64)
B0UX83_HUMAN (+1)	F5GYG5_HUMAN	REXO2	F5GYG5	Oligoribonuclease, mitochondrial	25 kDa	0.075	4	2	2	0	0	0	1	0	2	0	0	1.1196106	0% (0.64)
LTOR3_HUMAN (+1)	SLTM_HUMAN	SLTM	Q9NWH9	SAFB-like transcription modulator (Modulator of estrogen-induced transcription)	117 kDa	0.21	4	2	2	0	0	1	1	0	0	2	0	1.1196106	0% (0.64)
SMD1_HUMAN	B3KTA3_HUMAN		B3KTA3	Fascin	52 kDa	0.2	4	2	2	0	0	2	0	0	2	0	0	1.1196106	0% (0.64)
B4DRT2_HUMAN (+1)	B7Z3U1_HUMAN		B7Z3U1	cDNA FLJ53798, highly similar to Long-chain fatty acid transport protein 1 (EC 6.2.1.-)	71 kDa	0.19	4	2	2	2	0	0	1	0	0	1	1	1.1196106	0% (0.64)
H0YKD8_HUMAN (+2)	NDUBB_HUMAN	NDUFB11	Q9NX14	NADH dehydrogenase [ubiquinone] 1 beta subcomplex subunit 11, mitochondrial (Complex I-ESSS) (CI-ESSS) (NADH-ubiquinone oxidoreductase ESSS subunit) (Neuronal protein 17.3) (Np17.3) (p17.3)	17 kDa	0.21	4	2	2	2	1	0	1	2	0	0	0	1.1196106	0% (0.64)
B2R7I0_HUMAN (+1)	B4DQQ5_HUMAN		B4DQQ5	cDNA FLJ53159, highly similar to Nucleoporin NDC1	63 kDa	0.17	4	2	2	1	0	0	1	1	0	0	1	1.1196106	0% (0.64)
B2R9S4_HUMAN (+2)	RM16_HUMAN	MRPL16	Q9NX20	39S ribosomal protein L16, mitochondrial (L16mt) (MRP-L16)	28 kDa	0.1	4	2	2	1	0	0	1	1	0	1	0	1.1196106	0% (0.64)
B2R6F5_HUMAN (+1)	B4DJ54_HUMAN		B4DJ54	cDNA FLJ53010, highly similar to Soluble calcium-activated nucleotidase 1 (EC 3.6.1.6)	39 kDa	0.091	4	2	2	0	2	0	0	0	0	2	0	1.1196106	0% (0.64)
TBB4A_HUMAN	STAT2_HUMAN	STAT2	P52630	Signal transducer and activator of transcription 2 (p113)	98 kDa	0.44	4	2	2	0	0	0	2	0	2	0	0	1.1196106	0% (0.64)
ATPF1_HUMAN (+1)	ARP5L_HUMAN	ARPC5L	Q9BPX5	Actin-related protein 2/3 complex subunit 5-like protein (Arp2/3 complex 16 kDa subunit 2) (ARC16-2)	17 kDa	0.27	4	2	2	0	1	0	1	1	0	0	1	1.1196106	0% (0.64)

UMPS_HUMAN	B3KNI8_HUMAN		B3KNI8	cDNA FLJ14670 fis, clone NT2RP2003272, highly similar to Ubiquilin-1	63 kDa	0.13	4	2	2	0	1	1	0	0	0	2	0	1.1196106	0% (0.64)
PUF60_HUMAN	E9PN70_HUMAN	TRAPPC4	E9PN70	Trafficking protein particle complex subunit 4	29 kDa	0.14	4	2	2	0	0	1	1	0	2	0	0	1.1196106	0% (0.64)
ACTBL_HUMAN	B1ALI9_HUMAN	TXNIP	B1ALI9	Thioredoxin interacting protein (Fragment)	34 kDa	0.23	4	2	2	0	0	2	0	0	0	2	0	1.1196106	0% (0.64)
ALAT2_HUMAN	B2R8N1_HUMAN		B2R8N1	cDNA, FLJ93976, highly similar to Homo sapiens COP9 homolog (COP9), mRNA	23 kDa	0.17	4	2	2	0	0	0	2	1	1	0	1	1.1196106	0% (0.64)
CD109_HUMAN	Q53R32_HUMAN	RPIA	Q53R32	Putative uncharacterized protein RPIA (Fragment)	20 kDa	0.065	4	2	2	0	0	0	2	2	0	0	0	1.1196106	0% (0.64)
PPB1_HUMAN	C9J8U2_HUMAN	NAPRT1	C9J8U2	Nicotinate phosphoribosyltransferase (Nicotinate phosphoribosyltransferase domain containing 1, isoform CRA_e)	52 kDa	0.2	4	2	2	0	1	1	0	0	2	0	0	1.1196106	0% (0.64)
A8K5Y7_HUMAN (+1)	B4DDV1_HUMAN	IFIT5	B4DDV1	Interferon-induced protein with tetratricopeptide repeats 5 (cDNA FLJ53857, highly similar to Interferon-induced protein with tetratricopeptide repeats 5)	51 kDa	0.1	4	2	2	0	0	0	2	0	2	0	0	1.1196106	0% (0.64)
A8K2D6_HUMAN (+1)	ZN292_HUMAN	ZNF292	O60281	Zinc finger protein 292	305 kDa	0.2	2	1	1	0	1	0	0	0	1	0	0	1.11960888	0% (0.72)
Q4AEJ3_HUMAN (+1)	EXOSX_HUMAN	EXOSC10	Q01780	Exosome component 10 (EC 3.1.13.-) (Autoantigen PM/Scl 2) (P100 polymyositis-scleroderma overlap syndrome-associated autoantigen) (Polymyositis/scleroderma autoantigen 100 kDa) (PM/Scl-100) (Polymyositis/scleroderma autoantigen 2)	101 kDa	0.096	2	1	1	0	1	0	0	0	0	1	0	1.11960888	0% (0.72)
PSA3_HUMAN (+1)	PB1_HUMAN	PBRM1	Q86U86	Protein polybromo-1 (hPB1) (BRG1-associated factor 180) (BAF180) (Polybromo-1D)	193 kDa	0.25	2	1	1	0	0	0	1	0	0	1	0	1.11960888	0% (0.72)
RM47_HUMAN	CBP_HUMAN	CREBBP	Q92793	CREB-binding protein (EC 2.3.1.48)	265 kDa	0.088	2	1	1	1	0	0	0	0	1	0	0	1.11960888	0% (0.72)
SYYC_HUMAN	NUDT9_HUMAN	NUDT9	Q9BW91	ADP-ribose pyrophosphatase, mitochondrial (EC 3.6.1.13) (ADP-ribose diphosphatase) (ADP-ribose phosphohydrolase) (Adenosine diphosphoribose pyrophosphatase) (ADPR-PPase) (Nucleoside diphosphate-linked moiety X motif 9) (Nudix motif 9)	39 kDa	0.11	3	1	1	1	0	0	0	0	0	1	0	1.11960888	0% (0.72)
F5H0M4_HUMAN (+1)	ATAD5_HUMAN	ATAD5	Q96QE3	ATPase family AAA domain-containing protein 5 (Chromosome fragility-associated gene 1 protein)	208 kDa	0.22	2	1	1	1	0	0	0	0	1	0	0	1.11960888	0% (0.72)
DNJA2_HUMAN	M0QX35_HUMAN	PAF1	M0QX35	Paf1, RNA polymerase II associated factor, homolog (S. cerevisiae), isoform CRA_c (RNA polymerase II-associated factor 1 homolog)	55 kDa	0.14	2	1	1	0	1	0	0	0	0	1	0	1.11960888	0% (0.72)
RPB1_HUMAN	STRP1_HUMAN	STRIP1	Q5VSL9	Striatin-interacting protein 1 (Protein FAM40A)	96 kDa	0.039	2	1	1	0	0	1	0	1	0	0	0	1.11960888	0% (0.72)
NUP37_HUMAN	CORO7_HUMAN	CORO7	P57737	Coronin-7 (Crn7) (70 kDa WD repeat tumor rejection antigen homolog)	101 kDa	0.17	2	1	1	0	0	1	0	0	1	0	0	1.11960888	0% (0.72)
B4DUX0_HUMAN (+1)	CAND2_HUMAN	CAND2	O75155	Cullin-associated NEDD8-dissociated protein 2 (Cullin-associated and neddylation-dissociated protein 2) (Epididymis tissue protein Li 169) (TBP-interacting protein of 120 kDa B) (TBP-interacting protein 120B) (p120 CAND2)	135 kDa	0.31	2	1	1	0	0	1	0	0	1	0	0	1.11960888	0% (0.72)
RM37_HUMAN	DNJC9_HUMAN	DNAJC9	Q8WXX5	DnaJ homolog subfamily C member 9 (DnaJ protein SB73)	30 kDa	0.19	2	1	1	0	1	0	0	0	0	0	1	1.11960888	0% (0.72)

F5GWP8_HUMAN	A8K4P8_HUMAN		A8K4P8	cDNA FLJ75337	171 kDa	0.27	2	1	1	0	0	0	1	0	0	1	0	1.11960888	0% (0.72)
UGDH_HUMAN	LSG1_HUMAN	LSG1	Q9H089	Large subunit GTPase 1 homolog (hLsg1) (EC 3.6.1.-)	75 kDa	0.23	2	1	1	0	1	0	0	0	0	1	0	1.11960888	0% (0.72)
ACOX3_HUMAN (+1)	HD_HUMAN	HTT	P42858	Huntingtin (Huntington disease protein) (HD protein)	348 kDa	0.14	2	1	1	0	1	0	0	0	0	1	0	1.11960888	0% (0.72)
DYSF_HUMAN	GEMI4_HUMAN	GEMIN4	P57678	Gem-associated protein 4 (Gemin-4) (Component of gems 4) (p97)	120 kDa	0.056	2	1	1	0	0	0	1	1	0	0	0	1.11960888	0% (0.72)
MK01_HUMAN	B2R6Q2_HUMAN		B2R6Q2	Alkaline phosphatase (EC 3.1.3.1)	57 kDa	0.19	2	1	1	0	0	0	1	0	1	0	0	1.11960888	0% (0.72)
PSA7_HUMAN	FAKD5_HUMAN	FASTKD5	Q7L8L6	FAST kinase domain-containing protein 5	87 kDa	0.21	2	1	1	0	0	0	1	1	0	0	0	1.11960888	0% (0.72)
B4DP80_HUMAN	CC115_HUMAN	CCDC115	Q96NT0	Coiled-coil domain-containing protein 115	20 kDa	0.26	2	1	1	0	0	1	0	0	0	0	1	1.11960888	0% (0.72)
B7XGC2_HUMAN	A8K5N3_HUMAN		A8K5N3	Palmitoyltransferase (EC 2.3.1.-)	71 kDa	0.46	2	1	1	1	0	0	0	0	0	0	1	1.11960888	0% (0.72)
B3GQE6_HUMAN (+2)	CHM2A_HUMAN	CHMP2A	O43633	Charged multivesicular body protein 2a (Chromatin-modifying protein 2a) (CHMP2a) (Putative breast adenocarcinoma marker BC-2) (Vacuolar protein sorting-associated protein 2-1) (Vps2-1) (hVps2-1)	25 kDa	0.13	2	1	1	0	0	0	1	0	0	1	0	1.11960888	0% (0.72)
B3KUB6_HUMAN	TSR1_HUMAN	TSR1	Q2NL82	Pre-rRNA-processing protein TSR1 homolog	92 kDa	0.075	2	1	1	0	0	0	1	0	1	0	0	1.11960888	0% (0.72)
DRG1_HUMAN	ANPRA_HUMAN	NPR1	P16066	Atrial natriuretic peptide receptor 1 (EC 4.6.1.2) (Atrial natriuretic peptide receptor type A) (ANP-A) (ANPR-A) (NPR-A) (Guanylate cyclase A) (GC-A)	119 kDa	0.23	2	1	1	0	0	1	0	1	0	0	0	1.11960888	0% (0.72)
SYJ2B_HUMAN	DDX60_HUMAN	DDX60	Q8IY21	Probable ATP-dependent RNA helicase DDX60 (EC 3.6.4.13) (DEAD box protein 60)	198 kDa	0.25	2	1	1	0	0	0	1	0	1	0	0	1.11960888	0% (0.72)
NOP2_HUMAN	A8K367_HUMAN		A8K367	cDNA FLJ76104, highly similar to Homo sapiens carboxylesterase 2 (intestine, liver) (CES2), transcript variant 1, mRNA	69 kDa	0.067	2	1	1	1	0	0	0	1	0	0	0	1.11960888	0% (0.72)
P3H1_HUMAN	B2RBP3_HUMAN		B2RBP3	cDNA, FLJ95615, highly similar to Homo sapiens ubiquitin-activating enzyme E1C (UBA3 homolog, yeast)(UBE1C), mRNA	52 kDa	0.077	2	1	1	0	0	0	1	0	1	0	0	1.11960888	0% (0.72)
F5H5P2_HUMAN (+3)	E7ETU7_HUMAN	MRPL3	E7ETU7	39S ribosomal protein L3, mitochondrial	42 kDa	0.11	2	1	1	1	0	0	0	0	0	0	1	1.11960888	0% (0.72)
F5H3I5_HUMAN	ISCA2_HUMAN	ISCA2	Q86U28	Iron-sulfur cluster assembly 2 homolog, mitochondrial (HESB-like domain-containing protein 1)	16 kDa	0.092	2	1	1	0	1	0	0	1	0	0	0	1.11960888	0% (0.72)
CLD3_HUMAN	B4DMD3_HUMAN		B4DMD3	cDNA FLJ58174, highly similar to WW domain-binding protein 11	65 kDa	0.19	2	1	1	0	1	0	0	0	0	0	1	1.11960888	0% (0.72)
B2R6K4_HUMAN (+2)	SLAF8_HUMAN	SLAMF8	Q9P0V8	SLAM family member 8 (B-lymphocyte activator macrophage expressed) (BCM-like membrane protein) (CD antigen CD353)	32 kDa	0.22	2	1	1	1	0	0	0	0	0	0	1	1.11960888	0% (0.72)
J3QLR8_HUMAN (+1)	NDUB1_HUMAN	NDUFB1	O75438	NADH dehydrogenase [ubiquinone] 1 beta subcomplex subunit 1 (Complex I-MNLL) (CI-MNLL) (NADH-ubiquinone oxidoreductase MNLL subunit)	7 kDa	0.38	2	1	1	1	0	0	0	0	0	1	0	1.11960888	0% (0.72)
RAB9A_HUMAN	ARFRP_HUMAN	ARFRP1	Q13795	ADP-ribosylation factor-related protein 1 (ARF-related protein 1) (ARP)	23 kDa	0.21	2	1	1	0	1	0	0	0	0	0	1	1.11960888	0% (0.72)
B3KY60_HUMAN (+1)	C9J712_HUMAN	PFN2	C9J712	Profilin-2	10 kDa	0.086	2	1	1	0	0	0	1	0	1	0	0	1.11960888	0% (0.72)
CLIC5_HUMAN	DCAKD_HUMAN	DCAKD	Q8WVC6	Dephospho-CoA kinase domain-containing protein	27 kDa	0.15	2	1	1	1	0	0	0	0	0	1	0	1.11960888	0% (0.72)
Q53HG7_HUMAN (+1)	WDR82_HUMAN	WDR82	Q6UXN9	WD repeat-containing protein 82 (Protein TMEM113) (Swd2)	35 kDa	0.049	2	1	1	0	0	0	1	1	0	0	0	1.11960888	0% (0.72)
SC61G_HUMAN	B8K1J4_HUMAN		B8K1J4	Signal protein	28 kDa	0.37	2	1	1	1	0	0	0	1	0	0	0	1.11960888	0% (0.72)

RSF1_HUMAN	ALG9_HUMAN	ALG9	Q9H6U8	Alpha-1,2-mannosyltransferase ALG9 (EC 2.4.1.259) (EC 2.4.1.261) (Asparagine-linked glycosylation protein 9 homolog) (Disrupted in bipolar disorder protein 1) (Dol-P-Man:Man(6)GlcNAc(2)-PP-Dol alpha-1,2-mannosyltransferase) (Dol-P-Man:Man(8)GlcNAc(2)-PP-Dol alpha-1,2-mannosyltransferase)	70 kDa	0.035	2	1	1	1	0	0	0	0	0	0	1	1.11960888	0% (0.72)
SYFA_HUMAN	B4E126_HUMAN		B4E126	cDNA FLJ61199, highly similar to UBX domain-containing protein 7	39 kDa	0.11	2	1	1	0	0	1	0	1	0	0	0	1.11960888	0% (0.72)
E7EVQ6_HUMAN (+2)	DUS23_HUMAN	DUSP23	Q9BVJ7	Dual specificity protein phosphatase 23 (EC 3.1.3.16) (EC 3.1.3.48) (Low molecular mass dual specificity phosphatase 3) (LDP-3) (VH1-like phosphatase Z)	17 kDa	0.12	2	1	1	0	0	0	1	0	1	0	0	1.11960888	0% (0.72)
Q53GA0_HUMAN (+1)	F8VX04_HUMAN	SLC38A1	F8VX04	Sodium-coupled neutral amino acid transporter 1	56 kDa	0.25	2	1	1	0	1	0	0	1	0	0	0	1.11960888	0% (0.72)
PRP19_HUMAN	DHX9_HUMAN	DHX9	Q08211	ATP-dependent RNA helicase A (RHA) (EC 3.6.4.13) (DEAH box protein 9) (Leukophysin) (LKP) (Nuclear DNA helicase II) (NDH II)	141 kDa	0.13	346	174	173	23	54	44	53	49	25	62	37	1.113500877	0% (0.16)
A8K0F7_HUMAN (+1)	B4DWS6_HUMAN		B4DWS6	cDNA FLJ61181, highly similar to Homo sapiens hydroxysteroid (17-beta) dehydrogenase 12 (HSD17B12), mRNA	34 kDa	0.17	182	92	91	30	18	17	29	19	26	20	26	1.107741488	0% (0.26)
RAD50_HUMAN	A0PJ92_HUMAN	NOP56	A0PJ92	NOP56 protein (Fragment)	50 kDa	0.051	161	81	80	13	17	21	30	15	21	23	21	1.10611681	0% (0.28)
B4DXK8_HUMAN (+1)	ATPA_HUMAN	ATP5A1	P25705	ATP synthase subunit alpha, mitochondrial	60 kDa	0.082	611	309	304	100	68	63	79	64	80	67	95	1.102009316	0% (0.11)
CNOT1_HUMAN	GT251_HUMAN	COLGALT1	Q8NBJ5	Procollagen galactosyltransferase 1 (EC 2.4.1.50) (Collagen beta(1-O)galactosyltransferase 1) (Glycosyltransferase 25 family member 1) (Hydroxylysine galactosyltransferase 1)	72 kDa	0.042	112	57	56	9	24	18	7	10	10	15	22	1.100467056	0% (0.33)
WFS1_HUMAN	PROF1_HUMAN	PFN1	P07737	Profilin-1 (Epididymis tissue protein Li 184a) (Profilin I)	15 kDa	0.079	188	95	93	11	22	27	35	16	36	26	16	1.096471277	0% (0.28)
A5PLK7_HUMAN (+1)	RL10_HUMAN	RPL10	P27635	60S ribosomal protein L10 (Laminin receptor homolog) (Protein QM) (Tumor suppressor QM)	25 kDa	0.13	83	42	41	10	9	12	11	10	7	13	11	1.093775178	0% (0.38)
P5CR2_HUMAN	E9PLK3_HUMAN	NPEPPS	E9PLK3	Puromycin-sensitive aminopeptidase	103 kDa	0.24	81	42	41	12	7	6	17	15	7	11	7	1.093775178	0% (0.38)
D6RA47_HUMAN (+3)	SFXN1_HUMAN	SFXN1	Q9H9B4	Sideroflexin-1 (Tricarboxylate carrier protein) (TCC)	36 kDa	0.14	81	41	40	15	6	9	11	10	13	8	9	1.093160759	0% (0.38)
NU188_HUMAN	A0S3T1_HUMAN	COX2	A0S3T1	Cytochrome c oxidase subunit 2	26 kDa	0.069	79	41	40	14	10	6	11	10	10	9	11	1.093160759	0% (0.38)
PDIP2_HUMAN	B7Z8A2_HUMAN		B7Z8A2	cDNA FLJ51671, highly similar to Prenylcysteine oxidase (EC 1.8.3.5)	55 kDa	0.23	164	83	81	40	16	12	15	12	38	17	14	1.093137039	0% (0.30)
FCGRN_HUMAN	THIL_HUMAN	ACAT1	P24752	Acetyl-CoA acetyltransferase, mitochondrial (EC 2.3.1.9) (Acetoacetyl-CoA thiolase) (T2)	45 kDa	0.13	77	39	38	9	10	11	9	15	6	6	11	1.091840587	0% (0.39)
B7Z4A1_HUMAN (+1)	A8KA83_HUMAN		A8KA83	cDNA FLJ78586, highly similar to Homo sapiens VAMP (vesicle-associated membrane protein)-associated protein A, 33kDa (VAPA), mRNA (cDNA, FLJ96653, Homo sapiens VAMP (vesicle-associated membrane protein)-associated protein A, 33kDa (VAPA), mRNA)	27 kDa	0.2	73	37	36	12	8	8	9	9	11	7	9	1.090382718	0% (0.39)
A8K883_HUMAN	A8K897_HUMAN		A8K897	cDNA FLJ78686, highly similar to Homo sapiens nucleoporin 93kDa (NUP93), mRNA	93 kDa	0.1	71	36	35	5	10	11	10	10	4	12	9	1.089595216	0% (0.40)

AGR2_HUMAN (+3)	B5MDF5_HUMAN	RAN	B5MDF5	GTP-binding nuclear protein Ran (RAN, member RAS oncogene family, isoform CRA_c)	26 kDa	0.36	71	36	35	7	9	6	14	10	14	7	4	1.089595216	0% (0.40)
B4DNJ6_HUMAN (+1)	Q59GA1_HUMAN		Q59GA1	Splicing factor, arginine/serine-rich 10 (Transformer 2 homolog, Drosophila) variant (Fragment)	32 kDa	0.21	69	35	34	7	8	9	11	11	6	9	8	1.088764361	0% (0.40)
SYLM_HUMAN	CPNE3_HUMAN	CPNE3	O75131	Copine-3 (Copine III)	60 kDa	0.11	69	35	34	19	5	6	8	10	10	8	6	1.088764361	0% (0.40)
IPO9_HUMAN	RAB10_HUMAN	RAB10	P61026	Ras-related protein Rab-10	23 kDa	0.067	128	65	63	20	17	15	13	12	14	17	21	1.085884986	0% (0.34)
VAC14_HUMAN	Q6IQ30_HUMAN	PABPC4	Q6IQ30	PABPC4 protein	72 kDa	0.11	127	65	63	9	19	21	17	20	13	18	13	1.085884986	0% (0.34)
PGAM5_HUMAN	SOAT1_HUMAN	SOAT1	P35610	Sterol O-acyltransferase 1 (EC 2.3.1.26) (Acyl-coenzyme A:cholesterol acyltransferase 1) (ACAT-1) (Cholesterol acyltransferase 1)	65 kDa	0.22	61	31	30	8	9	10	4	1	3	6	20	1.084926749	0% (0.42)
CATS_HUMAN	B2R7W4_HUMAN		B2R7W4	cDNA, FLJ93632, highly similar to Homo sapiens heterogeneous nuclear ribonucleoprotein R (HNRPR), mRNA	71 kDa	0.21	185	95	92	18	33	23	22	33	9	31	20	1.084822003	0% (0.30)
RT02_HUMAN	PPIB_HUMAN	PPIB	P23284	Peptidyl-prolyl cis-trans isomerase B (PPIase B) (EC 5.2.1.8) (CYP-S1) (Cyclophilin B) (Rotamase B) (S-cyclophilin) (SCYLP)	24 kDa	0.061	248	126	122	28	27	27	43	12	37	34	38	1.084561053	0% (0.27)
D3DP46_HUMAN (+1)	A8KAP3_HUMAN		A8KAP3	cDNA FLJ78483, highly similar to Homo sapiens elongation factor Tu GTP binding domain containing 2 (EFTUD2), mRNA	109 kDa	0.18	185	94	91	27	25	24	20	19	13	32	28	1.084453925	0% (0.31)
HACD2_HUMAN	B3KY63_HUMAN		B3KY63	cDNA FLJ16830 fis, clone UTERU3022536, highly similar to Chromodomain helicase-DNA-binding protein 4 (EC 3.6.1.-)	215 kDa	0.094	55	28	27	3	7	8	11	11	2	9	5	1.081360976	0% (0.43)
PSA5_HUMAN (+1)	SMC1A_HUMAN	SMC1A	Q14683	Structural maintenance of chromosomes protein 1A (SMC protein 1A) (SMC-1-alpha) (SMC-1A) (Sb1.8)	143 kDa	0.25	56	28	27	7	7	13	1	12	0	13	4	1.081360976	0% (0.43)
F8W6G1_HUMAN (+1)	PSD12_HUMAN	PSMD12	O00232	26S proteasome non-ATPase regulatory subunit 12 (26S proteasome regulatory subunit RPN5) (26S proteasome regulatory subunit p55)	53 kDa	0.11	55	28	27	7	7	6	8	6	11	7	2	1.081360976	0% (0.43)
LTOR1_HUMAN	F5H6U7_HUMAN	GOLT1B	F5H6U7	Vesicle transport protein GOT1B	14 kDa	0.3	55	28	27	10	7	7	4	4	8	5	10	1.081360976	0% (0.43)
EIF3I_HUMAN	MYH9_HUMAN	MYH9	P35579	Myosin-9 (Cellular myosin heavy chain, type A) (Myosin heavy chain 9) (Myosin heavy chain, non-muscle IIa) (Non-muscle myosin heavy chain A) (NMMHC-A) (Non-muscle myosin heavy chain IIa) (NMMHC II-a) (NMMHC-IIA)	227 kDa	0.21	615	313	302	48	92	94	81	37	75	102	89	1.080766686	0% (0.17)
MIF_HUMAN	ACADM_HUMAN	ACADM	P11310	Medium-chain specific acyl-CoA dehydrogenase, mitochondrial (MCAD) (EC 1.3.8.7)	47 kDa	0.17	109	56	54	7	25	22	3	22	9	10	13	1.08055619	0% (0.37)
ATP5I_HUMAN	B3KTQ2_HUMAN		B3KTQ2	cDNA FLJ38589 fis, clone HCHON2010074, highly similar to LACTADHERIN	42 kDa	0.51	53	27	26	9	4	3	11	8	2	6	10	1.080004332	0% (0.44)
TMX4_HUMAN	OSBL8_HUMAN	OSBPL8	Q9BZF1	Oxysterol-binding protein-related protein 8 (ORP-8) (OSBP-related protein 8)	101 kDa	0.17	49	25	24	9	7	8	1	0	3	14	6	1.076981349	0% (0.45)
A8K309_HUMAN	AT2A3_HUMAN	ATP2A3	Q93084	Sarcoplasmic/endoplasmic reticulum calcium ATPase 3 (SERCA3) (SR Ca(2+)-ATPase 3) (EC 3.6.3.8) (Calcium pump 3)	114 kDa	0.17	50	25	24	0	7	10	4	4	0	0	12	1.076981349	0% (0.45)
B3KWW5_HUMAN (+3)	B4DTA2_HUMAN		B4DTA2	cDNA FLJ60148, highly similar to Homo sapiens heterogeneous nuclear ribonucleoprotein D-like	30 kDa	0.13	97	50	48	7	19	12	12	16	0	22	6	1.075966923	0% (0.39)

				(HNRPDL), transcript variant 2, mRNA															
UBA6_HUMAN	B4DVY2_HUMAN		B4DVY2	cDNA FLJ54184, highly similar to Tropomyosin alpha-4 chain	26 kDa	0.1	46	24	23	0	5	8	8	4	4	7	7	1.0752904 79	0% (0.46)
CKAP5_HUMAN	K6PL_HUMAN	PFKL	P17858	6-phosphofructokinase, liver type (EC 2.7.1.11) (Phosphofructo-1-kinase isozyme B) (PFK-B) (Phosphofructokinase 1) (Phosphohexokinase)	85 kDa	0.043	93	48	46	2	17	15	14	17	15	12	2	1.0741892 38	0% (0.40)
A4D2Q0_HUMAN	MPCP_HUMAN	SLC25A3	Q00325	Phosphate carrier protein, mitochondrial (Phosphate transport protein) (PTP) (Solute carrier family 25 member 3)	40 kDa	0.1	235	120	115	42	27	23	28	28	29	28	32	1.0735587 32	0% (0.31)
J3KN16_HUMAN	ECHP_HUMAN	EHHADH	Q08426	Peroxisomal bifunctional enzyme (PBE) (PBFE) [Includes: Enoyl-CoA hydratase/3,2-trans-enoyl-CoA isomerase (EC 4.2.1.17) (EC 5.3.3.8); 3-hydroxyacyl-CoA dehydrogenase (EC 1.1.1.35)]	79 kDa	0.039	43	22	21	3	8	10	1	5	1	5	10	1.0714728 29	0% (0.47)
J3QQQ9_HUMAN (+4)	B2RAK1_HUMAN		B2RAK1	cDNA, FLJ94965, highly similar to Homo sapiens leucyl/cystinyl aminopeptidase (LNPEP), mRNA	117 kDa	0.46	43	22	21	10	6	4	2	3	1	7	10	1.0714728 29	0% (0.47)
PSB1_HUMAN (+1)	B3KR70_HUMAN		B3KR70	cDNA FLJ33764 fis, clone BRCOC2000360, highly similar to Vacuolar ATP synthase subunit S1 (EC 3.6.3.14)	48 kDa	0.13	43	22	21	5	5	6	6	3	0	9	9	1.0714728 29	0% (0.47)
ALG11_HUMAN	B2RBA6_HUMAN		B2RBA6	cDNA, FLJ95407, highly similar to Homo sapiens MCM7 minichromosome maintenance deficient 7 (S. cerevisiae) (MCM7), mRNA	81 kDa	0.083	43	22	21	0	10	13	0	12	3	5	0	1.0714728 29	0% (0.47)
Q6PK82_HUMAN	B4DGU4_HUMAN	CTNNB1	B4DGU4	Catenin beta-1 (cDNA FLJ57878, highly similar to Beta-catenin)	85 kDa	0.091	89	45	43	14	14	9	8	16	2	16	9	1.0712349 98	0% (0.41)
B2R679_HUMAN (+2)	D3DSQ1_HUMAN	ASAH1	D3DSQ1	N-acylsphingosine amidohydrolase (Acid ceramidase) 1, isoform CRA_c	61 kDa	0.25	131	67	64	19	17	14	17	5	9	19	31	1.0704577 31	0% (0.37)
NBAS_HUMAN	MLEC_HUMAN	MLEC	Q14165	Malectin	32 kDa	0.008 9	86	44	42	16	9	9	10	8	12	8	15	1.0701633 54	0% (0.41)
ITPR2_HUMAN	NSDHL_HUMAN	NSDHL	Q15738	Sterol-4-alpha-carboxylate 3-dehydrogenase, decarboxylating (EC 1.1.1.170) (Protein H105e3)	42 kDa	0.044	41	21	20	8	2	5	6	6	5	3	6	1.0693068 67	0% (0.47)
TR150_HUMAN	DNJB1_HUMAN	DNAJB1	P25685	DnaJ homolog subfamily B member 1 (DnaJ protein homolog 1) (Heat shock 40 kDa protein 1) (HSP40) (Heat shock protein 40) (Human DnaJ protein 1) (hDj-1)	38 kDa	0.058	41	21	20	3	5	4	9	5	8	2	6	1.0693068 67	0% (0.47)
CSTF3_HUMAN	B1AVQ5_HUMAN	MUC1	B1AVQ5	Mucin-1 subunit alpha	49 kDa	0.063	41	21	20	10	2	3	6	8	0	4	8	1.0693068 67	0% (0.47)
SQSTM_HUMAN	SYIM_HUMAN	IARS2	Q9NSE4	Isoleucine--tRNA ligase, mitochondrial (EC 6.1.1.5) (Isoleucyl-tRNA synthetase) (IleRS)	114 kDa	0.14	166	85	81	27	22	16	22	26	12	19	24	1.0677614 67	0% (0.36)
RM39_HUMAN	K6PP_HUMAN	PFKP	Q01813	6-phosphofructokinase type C (EC 2.7.1.11) (6-phosphofructokinase, platelet type) (Phosphofructo-1-kinase isozyme C) (PFK-C) (Phosphofructokinase 1) (Phosphohexokinase)	86 kDa	0.11	123	63	60	11	19	15	17	20	18	16	6	1.0673871 02	0% (0.39)
B4DDP6_HUMAN (+2)	Q53GF9_HUMAN		Q53GF9	Full-length cDNA 5-PRIME end of clone CS0DF013YM24 of Fetal brain of Homo sapiens (Human) variant (Fragment)	26 kDa	0.081	125	63	60	17	13	14	19	10	19	13	19	1.0673871 02	0% (0.39)
E7ES33_HUMAN	B9EGQ8_HUMAN	SMARCA4	B9EGQ8	SMARCA4 protein	189 kDa	0.099	39	20	19	6	6	5	3	4	0	9	6	1.0669372 12	0% (0.48)
APOA1_HUMAN	B4DZF2_HUMAN		B4DZF2	cDNA FLJ59571, highly similar to Eukaryotic translation initiation factor 4gamma 2	110 kDa	0.3	39	20	19	0	6	10	4	7	4	6	1	1.0669372 12	0% (0.48)

GCSP_HUMAN	H0Y8C6_HUMAN	IPO5	H0Y8C6	Importin-5 (Fragment)	124 kDa	0.13	81	41	39	19	11	9	2	12	9	14	5	1.06664454	0% (0.42)
RL35_HUMAN	H3BLZ8_HUMAN	DDX17	H3BLZ8	Probable ATP-dependent RNA helicase DDX17	80 kDa	0.19	157	81	77	17	23	23	20	20	17	21	19	1.065229553	0% (0.37)
K7ELW0_HUMAN (+1)	TMX1_HUMAN	TMX1	Q9H3N1	Thioredoxin-related transmembrane protein 1 (Thioredoxin domain-containing protein 1) (Transmembrane Trx-related protein)	32 kDa	0.42	37	19	18	6	5	4	4	3	5	5	5	1.064333686	0% (0.49)
COX5A_HUMAN (+1)	C9JLI6_HUMAN	RPS27L	C9JLI6	40S ribosomal protein S27	11 kDa	0.15	37	19	18	7	4	5	3	5	2	5	6	1.064333686	0% (0.49)
Q96K24_HUMAN	B4DSQ5_HUMAN		B4DSQ5	cDNA FLJ53608, highly similar to Protein transport protein Sec23A	83 kDa	0.19	37	19	18	5	2	6	6	3	5	4	6	1.064333686	0% (0.49)
A8K6K7_HUMAN (+1)	B7ZB41_HUMAN		B7ZB41	cDNA, FLJ79405, highly similar to Homo sapiens solute carrier family 25, member 24, transcript variant 1, mRNA	53 kDa	0.12	149	77	73	27	14	12	23	13	18	16	26	1.062439491	0% (0.38)
KAT3_HUMAN	GSTK1_HUMAN	GSTK1	Q9Y2Q3	Glutathione S-transferase kappa 1 (EC 2.5.1.18) (GST 13-13) (GST class-kappa) (GSTK1-1) (hGSTK1) (Glutathione S-transferase subunit 13)	25 kDa	0.18	184	95	90	27	20	17	31	26	15	20	30	1.061524464	0% (0.36)
G3V2S6_HUMAN (+2)	Q53EY4_HUMAN		Q53EY4	RAB31, member RAS oncogene family variant (Fragment)	22 kDa	0.21	35	18	17	4	9	4	1	0	5	4	8	1.061459843	0% (0.50)
B3KME2_HUMAN (+3)	ACAD9_HUMAN	ACAD9	Q9H845	Acyl-CoA dehydrogenase family member 9, mitochondrial (ACAD-9) (EC 1.3.99.-)	69 kDa	0.17	106	55	52	23	15	10	7	20	3	14	15	1.059938652	0% (0.41)
Q6IBU0_HUMAN	SC22B_HUMAN	SEC22B	O75396	Vesicle-trafficking protein SEC22b (ER-Golgi SNARE of 24 kDa) (ERS-24) (ERS24) (SEC22 vesicle-trafficking protein homolog B) (SEC22 vesicle-trafficking protein-like 1)	25 kDa	0.084	105	54	51	12	16	17	9	14	7	12	18	1.058856211	0% (0.42)
B4DLN7_HUMAN	G3V0I5_HUMAN	NDUFV1	G3V0I5	NADH dehydrogenase (Ubiquinone) flavoprotein 1, 51kDa, isoform CRA_c (NADH dehydrogenase [ubiquinone] flavoprotein 1, mitochondrial)	50 kDa	0.17	69	35	33	13	8	6	8	11	7	10	6	1.057862204	0% (0.45)
NDUA6_HUMAN	UBR4_HUMAN	UBR4	Q5T4S7	E3 ubiquitin-protein ligase UBR4 (EC 6.3.2.-) (600 kDa retinoblastoma protein-associated factor) (N-recognin-4) (Retinoblastoma-associated factor of 600 kDa) (RBAF600) (p600) (Zinc finger UBR1-type protein 1)	574 kDa	0.32	31	16	15	0	3	11	2	7	4	5	0	1.054713159	0% (0.51)
A8MXB9_HUMAN	B4DJP7_HUMAN	SNRPD3	B4DJP7	Small nuclear ribonucleoprotein Sm D3 (cDNA FLJ51872, highly similar to Small nuclear ribonucleoprotein Sm D3)	13 kDa	0.18	31	16	15	5	4	5	4	2	5	4	4	1.054713159	0% (0.51)
HINT2_HUMAN	SPTC1_HUMAN	SPTLC1	O15269	Serine palmitoyltransferase 1 (EC 2.3.1.50) (Long chain base biosynthesis protein 1) (LCB 1) (Serine-palmitoyl-CoA transferase 1) (SPT 1) (SPT1)	53 kDa	0.29	31	16	15	7	6	2	1	3	4	3	5	1.054713159	0% (0.51)
TRA2A_HUMAN	MPRI_HUMAN	IGF2R	P11717	Cation-independent mannose-6-phosphate receptor (CI Man-6-P receptor) (CI-MPR) (M6PR) (300 kDa mannose 6-phosphate receptor) (MPR 300) (Insulin-like growth factor 2 receptor) (Insulin-like growth factor II receptor) (IGF-II receptor) (M6P/IGF2 receptor) (M6P/IGF2R) (CD antigen CD222)	274 kDa	0.082	65	33	31	11	12	9	1	4	7	10	10	1.054251781	0% (0.46)
RDH14_HUMAN	FBRL_HUMAN	FBL	P22087	rRNA 2'-O-methyltransferase fibrillarin (EC 2.1.1.-) (34 kDa nucleolar scleroderma antigen)	34 kDa	0.14	97	50	47	11	12	8	21	13	10	12	12	1.054104705	0% (0.43)

B4DVD7_HUMAN (+2)	B5BUB5_HUMAN	SSB	B5BUB5	Autoantigen La (Fragment)	47 kDa	0.35	62	32	30	0	9	8	15	12	11	4	3	1.052283848	0% (0.47)
GRB2_HUMAN (+1)	IF4A2_HUMAN	EIF4A2	Q14240	Eukaryotic initiation factor 4A-II (eIF-4A-II) (eIF4A-II) (EC 3.6.4.13) (ATP-dependent RNA helicase eIF4A-2)	46 kDa	0.18	127	65	61	0	17	13	26	17	22	0	0	1.052052825	0% (0.42)
HPRT_HUMAN (+1)	GRP78_HUMAN	HSPA5	P11021	78 kDa glucose-regulated protein (GRP-78) (Endoplasmic reticulum lumenal Ca(2+)-binding protein grp78) (Heat shock 70 kDa protein 5) (Immunoglobulin heavy chain-binding protein) (BiP)	72 kDa	0.27	1108	571	536	135	180	138	119	114	132	147	144	1.051525826	0% (0.19)
PDS5A_HUMAN	DX39B_HUMAN	DDX39B	Q13838	Spliceosome RNA helicase DDX39B (EC 3.6.4.13) (56 kDa U2AF65-associated protein) (ATP-dependent RNA helicase p47) (DEAD box protein UAP56) (HLA-B-associated transcript 1 protein)	49 kDa	0.073	91	48	45	3	12	11	22	13	11	16	5	1.05143994	0% (0.44)
DPYL2_HUMAN (+1)	B7Z815_HUMAN	USP7	B7Z815	Ubiquitin carboxyl-terminal hydrolase (EC 3.4.19.12)	126 kDa	0.25	29	15	14	0	4	4	7	1	2	7	2	1.050717364	0% (0.52)
Q53FW8_HUMAN (+1)	RUXE_HUMAN	SNRPE	P62304	Small nuclear ribonucleoprotein E (snRNP-E) (Sm protein E) (Sm-E) (SmE)	11 kDa	0.18	29	15	14	3	3	5	4	3	4	4	3	1.050717364	0% (0.52)
GNAI3_HUMAN	ARK72_HUMAN	AKR7A2	O43488	Aflatoxin B1 aldehyde reductase member 2 (EC 1.1.1.n11) (AFB1 aldehyde reductase 1) (AFB1-AR 1) (Aldoketoreductase 7) (Succinic semialdehyde reductase) (SSA reductase)	40 kDa	0.26	29	15	14	5	2	3	5	4	3	4	3	1.050717364	0% (0.52)
RM38_HUMAN	B4DRA0_HUMAN		B4DRA0	cDNA FLJ58459, highly similar to RNA-binding region-containing protein 2	56 kDa	0.092	90	46	43	0	14	13	20	11	6	16	10	1.048549878	0% (0.45)
VP26A_HUMAN	RAB14_HUMAN	RAB14	P61106	Ras-related protein Rab-14	24 kDa	0.25	177	91	85	25	21	24	21	16	25	20	24	1.046843286	0% (0.40)
B2R6X2_HUMAN (+1)	DNM1L_HUMAN	DNM1L	O00429	Dynamin-1-like protein (EC 3.6.5.5) (Dnm1p/Vps1p-like protein) (DVLP) (Dynamin family member proline-rich carboxyl-terminal domain less) (Dymple) (Dynamin-like protein) (Dynamin-like protein 4) (Dynamin-like protein IV) (HdynIV) (Dynamin-related protein 1)	82 kDa	0.061	27	14	13	1	5	4	4	2	8	1	2	1.046197756	0% (0.53)
J3KQ32_HUMAN (+1)	SEP15_HUMAN	42248	O60613	15 kDa selenoprotein	18 kDa	0.19	27	14	13	2	4	6	2	2	3	3	5	1.046197756	0% (0.53)
A8K2I7_HUMAN (+1)	AL9A1_HUMAN	ALDH9A1	P49189	4-trimethylaminobutyraldehyde dehydrogenase (TMABADH) (EC 1.2.1.47) (Aldehyde dehydrogenase E3 isozyme) (Aldehyde dehydrogenase family 9 member A1) (EC 1.2.1.3) (Gamma-aminobutyraldehyde dehydrogenase) (EC 1.2.1.19) (R-aminobutyraldehyde dehydrogenase)	54 kDa	0.22	56	29	27	4	9	7	9	12	9	4	2	1.045600067	0% (0.48)
NDUB4_HUMAN	PSD13_HUMAN	PSMD13	Q9UNM6	26S proteasome non-ATPase regulatory subunit 13 (26S proteasome regulatory subunit RPN9) (26S proteasome regulatory subunit S11) (26S proteasome regulatory subunit p40.5)	43 kDa	0.18	84	44	41	3	14	6	21	11	15	12	3	1.045404646	0% (0.46)
ICT1_HUMAN	M1VPF6_HUMAN	CD74-ROS1_C6;R32	M1VPF6	Tyrosine-protein kinase receptor (EC 2.7.10.1)	91 kDa	0.13	85	44	41	11	12	10	11	8	6	10	17	1.045404646	0% (0.46)
Q6LEU8_HUMAN	A2A274_HUMAN	ACO2	A2A274	Aconitate hydratase, mitochondrial	88 kDa	0.12	229	119	111	36	36	34	12	39	13	28	31	1.045192822	0% (0.38)
B7ZKM0_HUMAN (+1)	B2R6J2_HUMAN		B2R6J2	cDNA, FLJ92973, highly similar to Homo sapiens villin 2 (ezrin) (VIL2), mRNA	69 kDa	0.086	169	88	82	33	20	18	19	22	20	21	19	1.044390602	0% (0.41)
LY75_HUMAN	HM13_HUMAN	HM13	Q8TCT9	Minor histocompatibility antigen H13 (EC 3.4.23.-)	41 kDa	0.064	112	58	54	17	18	12	11	9	12	12	21	1.044054872	0% (0.44)

				(Intramembrane protease 1) (IMP-1) (IMPAS-1) (hIMP1) (Presenilin-like protein 3) (Signal peptide peptidase)														
HXK3_HUMAN	GRHPR_HUMAN	GRHPR	Q9UBQ7	Glyoxylate reductase/hydroxypyruvate reductase (EC 1.1.1.79) (EC 1.1.1.81)	36 kDa	0.15	54	28	26	3	11	4	10	12	5	4	4	1.043067701	0% (0.49)
C9JJ19_HUMAN (+1)	RT29_HUMAN	DAP3	P51398	28S ribosomal protein S29, mitochondrial (MRP-S29) (S29mt) (Death-associated protein 3) (DAP-3) (Ionizing radiation resistance conferring protein)	46 kDa	0.1	54	28	26	8	8	5	7	9	4	5	8	1.043067701	0% (0.49)
B4DJA5_HUMAN (+1)	SYK_HUMAN	KARS	Q15046	Lysine--tRNA ligase (EC 6.1.1.6) (Lysyl-tRNA synthetase) (LysRS)	68 kDa	0.36	81	42	39	5	20	14	4	17	7	11	4	1.041968844	0% (0.47)
ABCF1_HUMAN	MNDA_HUMAN	MNDA	P41218	Myeloid cell nuclear differentiation antigen	46 kDa	0.05	25	13	12	0	4	4	5	0	8	4	1	1.041044059	0% (0.54)
Q9HBK7_HUMAN (+1)	A8K2Q6_HUMAN		A8K2Q6	Peptidyl-prolyl cis-trans isomerase (EC 5.2.1.8)	23 kDa	0.074	25	13	12	2	4	3	4	4	1	2	5	1.041044059	0% (0.54)
B2RCZ4_HUMAN (+2)	B4DP80_HUMAN		B4DP80	cDNA FLJ56357, highly similar to Homo sapiens apolipoprotein A-I binding protein (APOA1BP), mRNA	34 kDa	0.11	25	13	12	2	3	1	7	3	4	2	3	1.041044059	0% (0.54)
B2R4G1_HUMAN (+3)	B4DVD7_HUMAN	EIF3K	B4DVD7	Eukaryotic translation initiation factor 3 subunit K (eIF3k) (Eukaryotic translation initiation factor 3 subunit 12) (eIF-3 p25)	25 kDa	0.29	24	13	12	2	4	2	5	2	6	3	1	1.041044059	0% (0.54)
A0SBM6_HUMAN (+202)	ABCB7_HUMAN	ABCB7	O75027	ATP-binding cassette sub-family B member 7, mitochondrial (ATP-binding cassette transporter 7) (ABC transporter 7 protein)	83 kDa	0.12	25	13	12	4	2	2	5	5	3	2	2	1.041044059	0% (0.54)
B5BU01_HUMAN (+1)	ACSF2_HUMAN	ACSF2	Q96CM8	Acyl-CoA synthetase family member 2, mitochondrial (EC 6.2.1.-)	68 kDa	0.14	52	27	25	13	6	4	4	10	3	5	7	1.040356175	0% (0.50)
Q3KQZ2_HUMAN (+1)	ACTBL_HUMAN	ACTBL2	Q562R1	Beta-actin-like protein 2 (Kappa-actin)	42 kDa	0.13	321	168	156	42	46	37	45	33	39	53	33	1.040315045	0% (0.37)
RAC2_HUMAN	B2RBD5_HUMAN		B2RBD5	cDNA, FLJ95457, highly similar to Homo sapiens tubulin, beta, 4 (TUBB4), mRNA	50 kDa	0.2	431	224	208	72	54	50	49	0	57	0	52	1.040198009	0% (0.34)
AL5AP_HUMAN	SAM50_HUMAN	SAMM50	Q9Y512	Sorting and assembly machinery component 50 homolog (Transformation-related gene 3 protein) (TRG-3)	52 kDa	0.27	80	41	38	13	8	3	17	10	15	6	7	1.040129084	0% (0.47)
GAK_HUMAN	AATM_HUMAN	GOT2	P00505	Aspartate aminotransferase, mitochondrial (mAspAT) (EC 2.6.1.1) (EC 2.6.1.7) (Fatty acid-binding protein) (FABP-1) (Glutamate oxaloacetate transaminase 2) (Kynurenine aminotransferase 4) (Kynurenine aminotransferase IV) (Kynurenine--oxoglutarate transaminase 4) (Kynurenine--oxoglutarate transaminase IV) (Plasma membrane-associated fatty acid-binding protein) (FABPpm) (Transaminase A)	48 kDa	0.076	181	95	88	26	24	21	24	41	13	13	20	1.038228267	0% (0.42)
B5BU72_HUMAN	PLD3_HUMAN	PLD3	Q8IV08	Phospholipase D3 (PLD 3) (EC 3.1.4.4) (Choline phosphatase 3) (HindIII K4L homolog) (Hu-K4) (Phosphatidylcholine-hydrolyzing phospholipase D3)	55 kDa	0.15	49	26	24	5	8	5	8	2	5	10	7	1.037445764	0% (0.50)
MIPEP_HUMAN	AAAS_HUMAN	AAAS	Q9NRG9	Aladin (Adracalin)	60 kDa	0.073	50	26	24	11	7	4	4	7	9	3	5	1.037445764	0% (0.50)
TMED4_HUMAN	PGAM1_HUMAN	PGAM1	P18669	Phosphoglycerate mutase 1 (EC 3.1.3.13) (EC 5.4.2.11) (EC 5.4.2.4) (BPG-dependent PGAM 1) (Phosphoglycerate mutase isozyme B) (PGAM-B)	29 kDa	0.2	50	26	24	6	7	4	10	5	9	8	2	1.037445764	0% (0.50)
AIMP2_HUMAN (+1)	STML2_HUMAN	STOML2	Q9UJZ1	Stomatin-like protein 2, mitochondrial (SLP-2)	39 kDa	0.12	50	26	24	5	7	8	6	7	7	5	5	1.037445764	0% (0.50)

				(EPB72-like protein 2) (Paraprotein target 7) (Paratarg-7)														
XPR1_HUMAN	RAB21_HUMAN	RAB21	Q9UL25	Ras-related protein Rab-21	24 kDa	0.07	101	53	49	15	16	13	9	9	12	15	13	1.037080289	0% (0.46)
D3K174_HUMAN (+1)	ADT3_HUMAN	SLC25A6	P12236	ADP/ATP translocase 3 (ADP,ATP carrier protein 3) (ADP,ATP carrier protein, isoform T2) (ANT 2) (Adenine nucleotide translocator 3) (ANT 3) (Solute carrier family 25 member 6)	33 kDa	0.041	337	174	161	53	40	34	51	29	49	38	46	1.036638505	0% (0.38)
A8K878_HUMAN (+1)	TAGL2_HUMAN	TAGLN2	P37802	Transgelin-2 (Epididymis tissue protein Li 7e) (SM22-alpha homolog)	22 kDa	0.37	74	39	36	6	11	12	10	12	9	6	9	1.036175571	0% (0.48)
PTN6_HUMAN (+2)	EIF3I_HUMAN	EIF3I	Q13347	Eukaryotic translation initiation factor 3 subunit I (eIF3i) (Eukaryotic translation initiation factor 3 subunit 2) (TGF-beta receptor-interacting protein 1) (TRIP-1) (eIF-3-beta) (eIF3 p36)	37 kDa	0.1	23	12	11	0	3	4	5	1	6	3	1	1.035112704	0% (0.55)
RT07_HUMAN	RAB25_HUMAN	RAB25	P57735	Ras-related protein Rab-25 (CATX-8)	23 kDa	0.24	23	12	11	4	2	2	4	3	6	2	0	1.035112704	0% (0.55)
LAMB2_HUMAN	EMC4_HUMAN	EMC4	Q5J8M3	ER membrane protein complex subunit 4 (Cell proliferation-inducing gene 17 protein) (Transmembrane protein 85)	20 kDa	0.059	23	12	11	3	3	3	3	2	3	3	3	1.035112704	0% (0.55)
LAS1L_HUMAN	ELOV1_HUMAN	ELOVL1	Q9BW60	Elongation of very long chain fatty acids protein 1 (EC 2.3.1.199) (3-keto acyl-CoA synthase ELOVL1) (ELOVL fatty acid elongase 1) (ELOVL FA elongase 1) (Very-long-chain 3-oxoacyl-CoA synthase 1)	33 kDa	0.13	50	25	23	6	7	7	5	5	4	7	8	1.034313739	0% (0.51)
ABCB7_HUMAN (+1)	B2RE46_HUMAN		B2RE46	cDNA, FLJ96923, highly similar to Homo sapiens ribophorin II (RPN2), mRNA	69 kDa	0.092	122	64	59	18	19	18	9	14	14	17	14	1.03384085	0% (0.46)
BIEA_HUMAN	PDIA1_HUMAN	P4HB	P07237	Protein disulfide-isomerase (PDI) (EC 5.3.4.1) (Cellular thyroid hormone-binding protein) (Prolyl 4-hydroxylase subunit beta) (p55)	57 kDa	0.23	614	321	296	67	102	98	58	37	97	82	82	1.032887207	0% (0.34)
TBL3_HUMAN	PAI2_HUMAN	SERPINB2	P05120	Plasminogen activator inhibitor 2 (PAI-2) (Monocyte Arg-serpin) (Placental plasminogen activator inhibitor) (Serpin B2) (Urokinase inhibitor)	47 kDa	0.14	96	50	46	4	26	19	1	0	2	20	24	1.032243117	0% (0.47)
KDEL2_HUMAN	A8K6Y1_HUMAN		A8K6Y1	cDNA FLJ75526, highly similar to Homo sapiens proliferation-associated 2G4, 38kDa (PA2G4), mRNA (Fragment)	41 kDa	0.11	45	24	22	1	6	8	9	5	11	4	2	1.030933769	0% (0.52)
B7Z6C2_HUMAN	PPAL_HUMAN	ACP2	P11117	Lysosomal acid phosphatase (LAP) (EC 3.1.3.2)	48 kDa	0.13	46	24	22	5	7	8	4	1	2	7	12	1.030933769	0% (0.52)
B3KS98_HUMAN	EMC3_HUMAN	EMC3	Q9P0I2	ER membrane protein complex subunit 3 (Transmembrane protein 111)	30 kDa	0.074	46	24	22	10	5	4	5	4	7	5	6	1.030933769	0% (0.52)
Q0PNF2_HUMAN	RAB8A_HUMAN	RAB8A	P61006	Ras-related protein Rab-8A (Oncogene c-mel)	24 kDa	0.044	95	49	45	13	15	12	9	8	11	14	13	1.030502514	0% (0.48)
MYO5B_HUMAN	ROA2_HUMAN	HNRNPA2B1	P22626	Heterogeneous nuclear ribonucleoproteins A2/B1 (hnRNP A2/B1)	37 kDa	0.07	407	212	195	27	64	53	70	55	22	76	42	1.030437542	0% (0.39)
RINI_HUMAN	ENOA_HUMAN	ENO1	P06733	Alpha-enolase (EC 4.2.1.11) (2-phospho-D-glycerate hydro-lyase) (C-myc promoter-binding protein) (Enolase 1) (MBP-1) (MPB-1) (Non-neural enolase) (NNE) (Phosphopyruvate hydratase) (Plasminogen-binding protein)	47 kDa	0.18	354	184	169	32	38	41	75	56	52	32	30	1.0290198	0% (0.40)
B4E205_HUMAN (+1)	SNAA_HUMAN	NAPA	P54920	Alpha-soluble NSF attachment protein (SNAP-alpha) (N-ethylmaleimide-sensitive factor attachment protein alpha)	33 kDa	0.074	92	48	44	11	11	9	17	8	12	9	15	1.028691297	0% (0.48)

RAB4B_HUMAN	Q6FGX3_HUMAN	RAB6A	Q6FGX3	RAB6A protein	24 kDa	0.35	93	48	44	14	12	10	12	8	14	9	13	1.028691297	0% (0.48)
PDXK_HUMAN	B4DVE1_HUMAN		B4DVE1	cDNA FLJ53478, highly similar to Galectin-3-binding protein	64 kDa	0.11	115	60	55	22	8	11	19	8	21	15	11	1.028231524	0% (0.47)
B4DLB8_HUMAN (+3)	COX5A_HUMAN	COX5A	P20674	Cytochrome c oxidase subunit 5A, mitochondrial (Cytochrome c oxidase polypeptide Va)	17 kDa	0.056	21	11	10	4	2	3	2	2	2	2	4	1.028213247	0% (0.56)
Q86XM2_HUMAN	RAC2_HUMAN	RAC2	P15153	Ras-related C3 botulinum toxin substrate 2 (GX) (Small G protein) (p21-Rac2)	21 kDa	0.074	20	11	10	3	2	4	1	0	2	3	5	1.028213247	0% (0.56)
ELYS_HUMAN	B7Z611_HUMAN		B7Z611	cDNA FLJ50848, highly similar to Homo sapiens suppressor of var1, 3-like 1 (S. cerevisiae) (SUPV3L1), mRNA (Fragment)	71 kDa	0.015	21	11	10	1	3	5	2	5	3	2	0	1.028213247	0% (0.56)
A8K2U2_HUMAN (+3)	RM49_HUMAN	MRPL49	Q13405	39S ribosomal protein L49, mitochondrial (L49mt) (MRP-L49) (Neighbor of FAU) (NOF) (Protein NOF1)	19 kDa	0.18	21	11	10	4	2	3	2	2	1	6	1	1.028213247	0% (0.56)
LRC8A_HUMAN	H7C579_HUMAN	NIT2	H7C579	Omega-amidase NIT2 (Fragment)	29 kDa	0.096	21	11	10	0	3	3	5	2	6	2	0	1.028213247	0% (0.56)
B4E3I3_HUMAN (+2)	MUTA_HUMAN	MUT	P22033	Methylmalonyl-CoA mutase, mitochondrial (MCM) (EC 5.4.99.2) (Methylmalonyl-CoA isomerase)	83 kDa	0.12	43	23	21	7	6	5	4	13	1	4	3	1.027275181	0% (0.52)
ANT3_HUMAN	RS7_HUMAN	RPS7	P62081	40S ribosomal protein S7	22 kDa	0.065	44	23	21	6	4	7	6	7	8	3	3	1.027275181	0% (0.52)
ALG2_HUMAN	Q59GK9_HUMAN		Q59GK9	Ribosomal protein L21 variant (Fragment)	19 kDa	0.094	44	23	21	6	6	5	6	5	6	4	6	1.027275181	0% (0.52)
FABD_HUMAN	B4E1G2_HUMAN		B4E1G2	Serine hydroxymethyltransferase (EC 2.1.2.1)	49 kDa	0.11	252	131	120	31	34	30	38	42	30	24	25	1.026527228	0% (0.43)
E5KSE7_HUMAN	UGGG1_HUMAN	UGGT1	Q9NYU2	UDP-glucose:glycoprotein glucosyltransferase 1 (UGT1) (hUGT1) (EC 2.4.1.-) (UDP--Glc:glycoprotein glucosyltransferase) (UDP-glucose ceramide glucosyltransferase-like 1)	177 kDa	0.16	225	117	107	29	34	31	23	21	22	23	41	1.024962693	0% (0.44)
WDR36_HUMAN	COPG1_HUMAN	COPG1	Q9Y678	Coatomer subunit gamma-1 (Gamma-1-coat protein) (Gamma-1-COP)	98 kDa	0.13	88	46	42	3	15	12	16	10	20	10	2	1.024839085	0% (0.49)
B4DVY2_HUMAN (+1)	F6IQY8_HUMAN	HLA-A	F6IQY8	MHC class I antigen (Fragment)	38 kDa	0.3	88	46	42	0	12	8	16	0	0	0	21	1.024839085	0% (0.49)
BPHL_HUMAN	C1PHA2_HUMAN	KIF5B-ALK	C1PHA2	Tyrosine-protein kinase receptor (EC 2.7.10.1)	168 kDa	0.25	42	22	20	7	7	6	2	2	8	7	3	1.023302023	0% (0.53)
A6NFX8_HUMAN (+1)	NUCL_HUMAN	NCL	P19338	Nucleolin (Protein C23)	77 kDa	0.3	216	113	103	7	33	36	37	31	26	30	16	1.021641399	0% (0.45)
A8K968_HUMAN (+1)	AT2A2_HUMAN	ATP2A2	P16615	Sarcoplasmic/endoplasmic reticulum calcium ATPase 2 (SERCA2) (SR Ca(2+)-ATPase 2) (EC 3.6.3.8) (Calcium pump 2) (Calcium-transporting ATPase sarcoplasmic reticulum type, slow twitch skeletal muscle isoform) (Endoplasmic reticulum class 1/2 Ca(2+) ATPase)	115 kDa	0.041	237	124	113	46	32	30	16	21	14	33	45	1.021311448	0% (0.45)
A7J992_HUMAN (+1)	NUMA1_HUMAN	NUMA1	Q14980	Nuclear mitotic apparatus protein 1 (NuMA protein) (SP-H antigen)	238 kDa	0.035	105	55	50	14	14	22	6	9	2	28	11	1.020099381	0% (0.49)
RISC_HUMAN	NAKD2_HUMAN	NADK2	Q4G0N4	NAD kinase 2, mitochondrial (EC 2.7.1.23) (Mitochondrial NAD kinase) (NAD kinase domain-containing protein 1, mitochondrial)	49 kDa	0.095	19	10	9	3	2	3	2	4	0	1	4	1.020087525	0% (0.58)
ASNS_HUMAN	PDS5A_HUMAN	PDS5A	Q29RF7	Sister chromatid cohesion protein PDS5 homolog A (Cell proliferation-inducing gene 54 protein) (Sister chromatid cohesion protein 112) (SCC-112)	151 kDa	0.22	19	10	9	1	2	2	5	4	0	5	0	1.020087525	0% (0.58)
RER1_HUMAN	TRNT1_HUMAN	TRNT1	Q96Q11	CCA tRNA nucleotidyltransferase 1, mitochondrial (EC 2.7.7.72) (Mitochondrial tRNA	50 kDa	0.15	19	10	9	2	4	0	4	4	3	1	1	1.020087525	0% (0.58)

				nucleotidyl transferase, CCA-adding) (mt CCA-adding enzyme) (mt tRNA CCA-diphosphorylase) (mt tRNA CCA-pyrophosphorylase) (mt tRNA adenylyltransferase)															
B7Z611_HUMAN (+1)	PLBL1_HUMAN	PLBD1	Q6P4A8	Phospholipase B-like 1 (EC 3.1.1.-) (LAMA-like protein 1) (Lamina ancestor homolog 1) (Phospholipase B domain-containing protein 1)	63 kDa	0.15	19	10	9	1	4	4	1	0	3	3	3	1.020087525	0% (0.58)
F5H1S9_HUMAN	A8K6V7_HUMAN		A8K6V7	cDNA FLJ76053, highly similar to Homo sapiens Ras-GTPase activating protein SH3 domain-binding protein 2 (G3BP2), transcript variant 3, mRNA	51 kDa	0.15	40	21	19	2	5	9	5	4	5	9	1	1.018971882	0% (0.54)
PPOX_HUMAN	B3KUZ7_HUMAN		B3KUZ7	cDNA FLJ40986 fis, clone UTERU2014898, highly similar to Vacuolar ATP synthase subunit H (EC 3.6.3.14)	56 kDa	0.065	40	21	19	6	6	5	4	1	4	9	5	1.018971882	0% (0.54)
BIG2_HUMAN	F8VXC8_HUMAN	SMARCC2	F8VXC8	SWI/SNF complex subunit SMARCC2	136 kDa	0.035	60	32	29	9	11	6	6	7	1	13	8	1.018596107	0% (0.52)
UTRO_HUMAN	I3L1P8_HUMAN	SLC25A11	I3L1P8	Mitochondrial 2-oxoglutarate/malate carrier protein (Fragment)	32 kDa	0.021	186	98	89	32	19	16	31	17	29	16	26	1.018102614	0% (0.47)
ARGI2_HUMAN	DLDH_HUMAN	DLD	P09622	Dihydrolipoyl dehydrogenase, mitochondrial (EC 1.8.1.4) (Dihydrolipoamide dehydrogenase) (Glycine cleavage system L protein)	54 kDa	0.22	206	108	98	28	24	24	32	32	23	20	23	1.017148483	0% (0.47)
ISG15_HUMAN	PRDX5_HUMAN	PRDX5	P30044	Peroxiredoxin-5, mitochondrial (EC 1.11.1.15) (Alu corepressor 1) (Antioxidant enzyme B166) (AOEB166) (Liver tissue 2D-page spot 71B) (PLP) (Peroxiredoxin V) (Prx-V) (Peroxisomal antioxidant enzyme) (TPx type VI) (Thioredoxin peroxidase PMP20) (Thioredoxin reductase)	22 kDa	0.37	119	63	57	21	12	8	22	14	12	13	18	1.015063076	0% (0.50)
A7MAP1_HUMAN	A5YM53_HUMAN	ITGAV	A5YM53	ITGAV protein	116 kDa	0.093	98	52	47	19	14	12	7	7	7	16	16	1.014487833	0% (0.51)
MK67I_HUMAN	B4E324_HUMAN		B4E324	cDNA FLJ60397, highly similar to Lysosomal protective protein (EC 3.4.16.5)	54 kDa	0.14	38	20	18	5	6	6	3	3	1	7	7	1.014234361	0% (0.55)
A8K329_HUMAN (+4)	HP1B3_HUMAN	HP1BP3	Q5SSJ5	Heterochromatin protein 1-binding protein 3 (Protein HP1-BP74)	61 kDa	0.082	38	20	18	7	2	6	6	7	2	3	6	1.014234361	0% (0.55)
DNJA1_HUMAN	A4D210_HUMAN	EIF3B	A4D210	Eukaryotic translation initiation factor 3 subunit B (eIF3b) (Eukaryotic translation initiation factor 3 subunit 9) (eIF-3-eta)	89 kDa	0.086	38	20	18	1	9	8	2	1	8	4	6	1.014234361	0% (0.55)
Q53G19_HUMAN (+1)	Q53HV1_HUMAN		Q53HV1	Ribosomal protein S4, X-linked X isoform variant (Fragment)	30 kDa	0.31	139	73	66	12	20	19	23	20	21	13	10	1.014066628	0% (0.49)
ABCD1_HUMAN	ODO2_HUMAN	DLST	P36957	Dihydrolipoyllysine-residue succinyltransferase component of 2-oxoglutarate dehydrogenase complex, mitochondrial (EC 2.3.1.61) (2-oxoglutarate dehydrogenase complex component E2) (OGDC-E2) (Dihydrolipoamide succinyltransferase component of 2-oxoglutarate dehydrogenase complex) (E2K)	49 kDa	0.079	139	73	66	24	14	22	15	18	20	9	18	1.014066628	0% (0.49)
A8K9B9_HUMAN (+1)	HNRPL_HUMAN	HNRNPL	P14866	Heterogeneous nuclear ribonucleoprotein L (hnRNP L)	64 kDa	0.18	178	94	85	11	31	31	22	22	12	37	14	1.01383272	0% (0.48)
ACACA_HUMAN	A8K651_HUMAN		A8K651	cDNA FLJ75700, highly similar to Homo sapiens	31 kDa	0.065	77	41	37	9	7	8	17	11	10	5	11	1.013614392	0% (0.52)

				complement component 1, q subcomponent binding protein (C1QBP), nuclear gene encoding mitochondrial protein, mRNA															
APOO_HUMAN (+2)	B3KY95_HUMAN		B3KY95	cDNA FLJ16143 fis, clone BRAMY2038516, highly similar to Protein disulfide-isomerase A6 (EC 5.3.4.1)	53 kDa	0.22	237	125	113	27	35	33	31	25	34	22	32	1.013208814	0% (0.48)
H3BV80_HUMAN	IPYR2_HUMAN	PPA2	Q9H2U2	Inorganic pyrophosphatase 2, mitochondrial (EC 3.6.1.1) (Pyrophosphatase SID6-306) (Pyrophosphate phospho-hydrolase 2) (PPase 2)	38 kDa	0.15	137	72	65	16	23	18	15	21	15	6	23	1.012624069	0% (0.50)
A8K7H3_HUMAN (+2)	RHG01_HUMAN	ARHGAP1	Q07960	Rho GTPase-activating protein 1 (CDC42 GTPase-activating protein) (GTPase-activating protein rhoOGAP) (Rho-related small GTPase protein activator) (Rho-type GTPase-activating protein 1) (p50-RhoGAP)	50 kDa	0.38	57	30	27	6	5	7	12	2	13	8	4	1.012127856	0% (0.53)
B1AKV3_HUMAN (+1)	1433E_HUMAN	YWHAE	P62258	14-3-3 protein epsilon (14-3-3E)	29 kDa	0.22	95	50	45	10	14	13	13	17	9	11	8	1.010382159	0% (0.52)
VPP2_HUMAN	B4E205_HUMAN		B4E205	cDNA FLJ61651, highly similar to Protein transport protein Sec24A	96 kDa	0.091	18	9	8	1	4	3	1	3	1	3	1	1.010376632	0% (0.59)
G0XQ39_HUMAN (+1)	B4DEK4_HUMAN	SNX2	B4DEK4	Sorting nexin-2 (cDNA FLJ51799, highly similar to Sorting nexin-2)	46 kDa	0.063	17	9	8	1	2	2	4	2	4	0	2	1.010376632	0% (0.59)
ARHL2_HUMAN	B4DMR0_HUMAN		B4DMR0	cDNA FLJ60706, highly similar to Serine/threonine-protein kinase 24 (EC 2.7.11.1)	38 kDa	0.18	17	9	8	0	2	1	6	2	2	3	1	1.010376632	0% (0.59)
URP2_HUMAN	Q4LE60_HUMAN	TNPO2	Q4LE60	TNPO2 variant protein (Fragment)	118 kDa	0.1	17	9	8	0	0	4	5	3	1	4	0	1.010376632	0% (0.59)
COPZ1_HUMAN (+6)	H2B1B_HUMAN	HIST1H2BB	P33778	Histone H2B type 1-B (Histone H2B.1) (Histone H2B.f) (H2B/f)	14 kDa	0.25	846	447	403	111	92	97	148	128	91	64	122	1.009761894	0% (0.43)
A8K607_HUMAN (+2)	CPSF1_HUMAN	CPSF1	Q10570	Cleavage and polyadenylation specificity factor subunit 1 (Cleavage and polyadenylation specificity factor 160 kDa subunit) (CPSF 160 kDa subunit)	161 kDa	0.12	37	19	17	3	5	4	7	3	1	8	4	1.009029106	0% (0.56)
FAD1_HUMAN	1433B_HUMAN	YWHAB	P31946	14-3-3 protein beta/alpha (Protein 1054) (Protein kinase C inhibitor protein 1) (KCIP-1) [Cleaved into: 14-3-3 protein beta/alpha, N-terminally processed]	28 kDa	0.17	55	29	26	5	8	7	9	8	0	6	8	1.008573162	0% (0.54)
B3KQ33_HUMAN (+1)	Q8TBK5_HUMAN	RPL6	Q8TBK5	60S ribosomal protein L6	33 kDa	0.088	127	68	61	15	17	15	21	16	16	15	14	1.006437704	0% (0.51)
GSTM3_HUMAN	P4HA2_HUMAN	P4HA2	O15460	Prolyl 4-hydroxylase subunit alpha-2 (4-PH alpha-2) (EC 1.14.11.2) (Procollagen-proline,2-oxoglutarate-4-dioxygenase subunit alpha-2)	61 kDa	0.36	53	28	25	1	16	11	0	8	1	6	10	1.004775529	0% (0.55)
SAP18_HUMAN	P4K2A_HUMAN	PI4K2A	Q9BTU6	Phosphatidylinositol 4-kinase type 2-alpha (EC 2.7.1.67) (Phosphatidylinositol 4-kinase type II-alpha)	54 kDa	0.2	89	47	42	26	10	9	4	1	10	11	20	1.003585995	0% (0.53)
AGAL_HUMAN (+2)	DRS7B_HUMAN	DHRS7B	Q6IAN0	Dehydrogenase/reductase SDR family member 7B (EC 1.1.-.-)	35 kDa	0.096	34	18	16	7	1	3	7	4	3	4	5	1.003283225	0% (0.57)
B4DEL5_HUMAN	ATPG_HUMAN	ATP5C1	P36542	ATP synthase subunit gamma, mitochondrial (F-ATPase gamma subunit)	33 kDa	0.28	124	66	59	21	10	16	19	13	18	8	19	1.003068857	0% (0.52)
B4DGM3_HUMAN (+4)	HNRH2_HUMAN	HNRNPH2	P55795	Heterogeneous nuclear ribonucleoprotein H2 (hnRNP H2) (FTP-3) (Heterogeneous nuclear ribonucleoprotein H') (hnRNP H') [Cleaved into: Heterogeneous nuclear ribonucleoprotein H2, N-terminally processed]	49 kDa	0.069	122	65	58	14	16	14	21	16	15	17	10	1.001308236	0% (0.53)
TOR1B_HUMAN	H2B1D_HUMAN	HIST1H2BD	P58876	Histone H2B type 1-D (HIRA-interacting protein 2) (Histone H2B.1 B) (Histone H2B.b) (H2B/b)	14 kDa	0.26	855	453	405	112	96	96	150	130	93	64	121	1.001305449	0% (0.48)

C9JRZ6_HUMAN	VDAC1_HUMAN	VDAC1	P21796	Voltage-dependent anion-selective channel protein 1 (VDAC-1) (hVDAC1) (Outer mitochondrial membrane protein porin 1) (Plasmalemmal porin) (Porin 31HL) (Porin 31HM)	31 kDa	0.19	248	131	117	40	26	30	35	23	34	27	34	1.001085341	0% (0.51)
B2L13_HUMAN	G3V0E8_HUMAN	PCBP2	G3V0E8	Poly(RC) binding protein 2, isoform CRA_f (Poly(rC)-binding protein 2)	33 kDa	0.21	51	27	24	3	7	7	10	9	8	6	2	1.000709159	0% (0.56)
RAB25_HUMAN	PLAK_HUMAN	JUP	P14923	Junction plakoglobin (Catenin gamma) (Desmoplakin III) (Desmoplakin-3)	82 kDa	0.22	142	74	66	22	18	17	17	20	7	17	22	1.000577793	0% (0.53)
A8KAH7_HUMAN (+2)	MCCB_HUMAN	MCCC2	Q9HCC0	Methylcrotonoyl-CoA carboxylase beta chain, mitochondrial (MCCase subunit beta) (EC 6.4.1.4) (3-methylcrotonyl-CoA carboxylase 2) (3-methylcrotonyl-CoA carboxylase non-biotin-containing subunit) (3-methylcrotonyl-CoA:carbon dioxide ligase subunit beta)	61 kDa	0.15	105	55	49	21	8	7	19	11	12	10	17	1.000180605	0% (0.53)
B4E2S3_HUMAN	A8K7T4_HUMAN		A8K7T4	cDNA FLJ75774, highly similar to Homo sapiens lectin, mannose-binding 2 (LMAN2), mRNA	40 kDa	0.057	68	36	32	14	10	3	9	4	13	7	8	-1.000621716	0% (0.55)
CPSF6_HUMAN (+1)	SF01_HUMAN	SF1	Q15637	Splicing factor 1 (Mammalian branch point-binding protein) (BBP) (mBBP) (Transcription factor ZFM1) (Zinc finger gene in MEN1 locus) (Zinc finger protein 162)	68 kDa	0.11	15	8	7	2	2	2	2	0	0	2	5	-1.001435599	0% (0.59)
D3DTC2_HUMAN (+1)	PP1R7_HUMAN	PPP1R7	Q15435	Protein phosphatase 1 regulatory subunit 7 (Protein phosphatase 1 regulatory subunit 22)	42 kDa	0.14	15	8	7	0	2	3	3	2	1	1	3	-1.001435599	0% (0.59)
M0QXB4_HUMAN	CKLF6_HUMAN	CMTM6	Q9NX76	CKLF-like MARVEL transmembrane domain-containing protein 6 (Chemokine-like factor superfamily member 6)	20 kDa	0.19	15	8	7	2	2	3	2	1	2	1	3	-1.001435599	0% (0.59)
H13_HUMAN	K7EIN2_HUMAN	NUDT16L1	K7EIN2	Protein syndesmos (Fragment)	22 kDa	0.37	15	8	7	1	0	1	6	1	3	2	0	-1.001435599	0% (0.59)
PADI2_HUMAN	PPP6_HUMAN	PPP6C	O00743	Serine/threonine-protein phosphatase 6 catalytic subunit (PP6C) (EC 3.1.3.16) [Cleaved into: Serine/threonine-protein phosphatase 6 catalytic subunit, N-terminally processed]	35 kDa	0.11	15	8	7	0	3	3	2	2	4	1	0	-1.001435599	0% (0.59)
B2RMN7_HUMAN (+2)	HCD2_HUMAN	HSD17B10	Q99714	3-hydroxyacyl-CoA dehydrogenase type-2 (EC 1.1.1.35) (17-beta-hydroxysteroid dehydrogenase 10) (17-beta-HSD 10) (EC 1.1.1.51) (3-hydroxy-2-methylbutyryl-CoA dehydrogenase) (EC 1.1.1.178) (3-hydroxyacyl-CoA dehydrogenase type II) (Endoplasmic reticulum-associated amyloid beta-peptide-binding protein) (Mitochondrial ribonuclease P protein 2) (Mitochondrial RNase P protein 2) (Short-chain type dehydrogenase/reductase XH98G2) (Type II HADH)	27 kDa	0.059	171	91	81	24	23	19	24	23	21	16	20	-1.00176659	0% (0.53)
A8K494_HUMAN (+7)	B2R4D8_HUMAN		B2R4D8	60S ribosomal protein L27	16 kDa	0.086	32	17	15	4	5	3	5	4	4	3	4	-1.003101745	0% (0.56)
B3KRI2_HUMAN (+1)	A8KAH1_HUMAN		A8KAH1	cDNA FLJ75839, highly similar to Homo sapiens phosphatidylserine synthase 1 (PTDSS1), mRNA	55 kDa	0.11	31	17	15	5	4	4	4	5	2	4	4	-1.003101745	0% (0.56)

NDUAB_HUMAN	A8K435_HUMAN		A8K435	cDNA FLJ78564, highly similar to Homo sapiens aldehyde dehydrogenase 5 family, member A1 (succinate-semialdehyde dehydrogenase) (ALDH5A1), nuclear gene encoding mitochondrial protein, transcript variant 2, mRNA	57 kDa	0.24	32	17	15	2	6	4	5	7	1	5	2	-1.003101745	0% (0.56)
B3KM95_HUMAN (+2)	ADPGK_HUMAN	ADPGK	Q9BRR6	ADP-dependent glucokinase (ADP-GK) (ADPGK) (EC 2.7.1.147) (RbBP-35)	54 kDa	0.081	50	26	23	6	4	9	7	1	8	4	11	-1.003668945	0% (0.55)
LMF2_HUMAN	PTBP1_HUMAN	PTBP1	P26599	Polypyrimidine tract-binding protein 1 (PTB) (57 kDa RNA-binding protein PPTB-1) (Heterogeneous nuclear ribonucleoprotein I) (hnRNP I)	57 kDa	0.078	65	35	31	3	8	14	10	8	5	13	4	-1.003955021	0% (0.54)
GSHR_HUMAN	BGAL_HUMAN	GLB1	P16278	Beta-galactosidase (EC 3.2.1.23) (Acid beta-galactosidase) (Lactase) (Elastin receptor 1)	76 kDa	0.14	84	44	39	16	11	10	7	7	5	16	12	-1.004127599	0% (0.54)
DHTK1_HUMAN	KAD3_HUMAN	AK3	Q9UIJ7	GTP:AMP phosphotransferase AK3, mitochondrial (EC 2.7.4.10) (Adenylate kinase 3) (AK 3) (Adenylate kinase 3 alpha-like 1)	26 kDa	0.047	83	44	39	16	11	10	7	18	5	4	12	-1.004127599	0% (0.54)
J3KRX2_HUMAN	RAB5C_HUMAN	RAB5C	P51148	Ras-related protein Rab-5C (L1880) (RAB5L)	23 kDa	0.17	100	53	47	17	14	13	9	10	14	9	15	-1.004243134	0% (0.53)
WDR18_HUMAN	PML_HUMAN	PML	P29590	Protein PML (Promyelocytic leukemia protein) (RING finger protein 71) (Tripartite motif-containing protein 19)	98 kDa	0.11	133	71	63	38	5	8	20	10	14	22	17	-1.004388307	0% (0.53)
ITA6_HUMAN	CLH1_HUMAN	CLTC	Q00610	Clathrin heavy chain 1 (Clathrin heavy chain on chromosome 17) (CLH-17)	192 kDa	0.071	707	375	333	68	118	126	65	91	81	78	84	-1.005429438	0% (0.51)
COR1A_HUMAN	ODPB_HUMAN	PDHB	P11177	Pyruvate dehydrogenase E1 component subunit beta, mitochondrial (PDHE1-B) (EC 1.2.4.1)	39 kDa	0.091	246	131	116	29	34	35	34	44	25	22	25	-1.007449889	0% (0.51)
B2R5N4_HUMAN (+1)	A8K032_HUMAN		A8K032	cDNA FLJ75031, highly similar to Homo sapiens translocation associated membrane protein 1 (TRAM1), mRNA	43 kDa	0.17	46	25	22	9	5	5	6	4	5	7	6	-1.008422997	0% (0.54)
APOL2_HUMAN	VIME_HUMAN	VIM	P08670	Vimentin	54 kDa	0.11	387	207	183	46	46	56	60	9	42	86	47	-1.009540303	0% (0.50)
B4DHL7_HUMAN (+6)	RS12_HUMAN	RPS12	P25398	40S ribosomal protein S12	15 kDa	0.092	30	16	14	1	5	4	6	7	3	3	1	-1.010311754	0% (0.56)
A8K6H9_HUMAN (+1)	APOOL_HUMAN	APOOL	Q6UXV4	Apolipoprotein O-like (Protein FAM121A)	29 kDa	0.085	30	16	14	7	1	1	7	5	3	2	4	-1.010311754	0% (0.56)
S12A7_HUMAN	J3KQ48_HUMAN	PTRH2	J3KQ48	Peptidyl-tRNA hydrolase 2, mitochondrial	19 kDa	0.077	30	16	14	5	4	4	3	5	2	3	4	-1.010311754	0% (0.56)
LGUL_HUMAN	ACL6A_HUMAN	ACTL6A	O96019	Actin-like protein 6A (53 kDa BRG1-associated factor A) (Actin-related protein Baf53a) (ArpNbeta) (BRG1-associated factor 53A) (BAF53A) (INO80 complex subunit K)	47 kDa	0.32	30	16	14	5	4	5	2	5	1	3	5	-1.010311754	0% (0.56)
M1RL68_HUMAN	A8K0T9_HUMAN		A8K0T9	cDNA FLJ75422, highly similar to Homo sapiens capping protein (actin filament) muscle Z-line, alpha 1, mRNA	33 kDa	0.34	31	16	14	4	4	2	6	5	3	3	3	-1.010311754	0% (0.56)
QCR7_HUMAN	FIS1_HUMAN	FIS1	Q9Y3D6	Mitochondrial fission 1 protein (FIS1 homolog) (hFis1) (Tetratricopeptide repeat protein 11) (TPR repeat protein 11)	17 kDa	0.5	30	16	14	5	2	2	7	2	3	4	5	-1.010311754	0% (0.56)
ZN326_HUMAN	B3KVX6_HUMAN		B3KVX6	cDNA FLJ41699 fis, clone HCHON2004776, highly similar to Homo sapiens cytoskeleton-associated protein 4 (CKAP4), mRNA	58 kDa	0.05	236	126	111	41	40	25	23	5	27	35	44	-1.012547504	0% (0.50)

F5H315_HUMAN (+3)	ADT2_HUMAN	SLC25A5	P05141	ADP/ATP translocase 2 (ADP,ATP carrier protein 2) (ADP,ATP carrier protein, fibroblast isoform) (Adenine nucleotide translocator 2) (ANT 2) (Solute carrier family 25 member 5) [Cleaved into: ADP/ATP translocase 2, N-terminally processed]	33 kDa	0.1	427	227	200	57	54	45	75	48	65	44	46	-1.013039683	0% (0.48)
F8W7U8_HUMAN (+1)	A8K590_HUMAN		A8K590	cDNA FLJ77456, highly similar to Homo sapiens interleukin enhancer binding factor 3, 90kDa (ILF3), transcript variant 2, mRNA	76 kDa	0.059	188	100	88	12	33	29	26	25	4	40	18	-1.013279785	0% (0.50)
E7D7X9_HUMAN (+2)	CISD2_HUMAN	CISD2	Q8N5K1	CDGSH iron-sulfur domain-containing protein 2 (Endoplasmic reticulum intermembrane small protein) (MitoNEET-related 1 protein) (Miner1) (Nutrient-deprivation autophagy factor-1) (NAF-1)	15 kDa	0.18	45	24	21	6	4	6	8	1	4	7	9	-1.013604256	0% (0.54)
APEX1_HUMAN	OFUT1_HUMAN	POFUT1	Q9H488	GDP-fucose protein O-fucosyltransferase 1 (EC 2.4.1.221) (Peptide-O-fucosyltransferase 1) (O-FucT-1)	44 kDa	0.36	45	24	21	6	9	4	5	2	7	7	5	-1.013604256	0% (0.54)
GOGA4_HUMAN (+1)	PABP1_HUMAN	PABPC1	P11940	Polyadenylate-binding protein 1 (PABP-1) (Poly(A)-binding protein 1)	71 kDa	0.0094	219	116	102	17	39	36	25	31	25	24	23	-1.014301769	0% (0.49)
SE1L3_HUMAN	COPA_HUMAN	COPA	P53621	Coatomer subunit alpha (Alpha-coat protein) (Alpha-COP) (HEP-COP) (HEPCOP) [Cleaved into: Xenin (Xenopsin-related peptide); Proxenin]	138 kDa	0.052	185	99	87	24	29	29	19	26	22	16	23	-1.014642166	0% (0.50)
UN45A_HUMAN	ITA6_HUMAN	ITGA6	P23229	Integrin alpha-6 (CD49 antigen-like family member F) (VLA-6) (CD antigen CD49f) [Cleaved into: Integrin alpha-6 heavy chain; Integrin alpha-6 light chain]	127 kDa	0.11	13	7	6	4	1	2	0	1	0	3	2	-1.016370031	0% (0.59)
BCAT1_HUMAN	B4DTY9_HUMAN	ITGB3	B4DTY9	Integrin beta	84 kDa	0.11	12	7	6	6	1	0	0	3	0	2	1	-1.016370031	0% (0.59)
AP1M2_HUMAN (+2)	PWP2_HUMAN	PWP2	Q15269	Periodic tryptophan protein 2 homolog	102 kDa	0.13	13	7	6	1	1	1	4	2	0	3	1	-1.016370031	0% (0.59)
B7Z7P8_HUMAN (+2)	A8K885_HUMAN		A8K885	cDNA FLJ77179, highly similar to Homo sapiens sorting nexin 6 (SNX6) mRNA	47 kDa	0.16	13	7	6	0	2	0	5	1	3	1	1	-1.016370031	0% (0.59)
PRAF1_HUMAN	ANAG_HUMAN	NAGLU	P54802	Alpha-N-acetylglucosaminidase (EC 3.2.1.50) (N-acetyl-alpha-glucosaminidase) (NAG) [Cleaved into: Alpha-N-acetylglucosaminidase 82 kDa form; Alpha-N-acetylglucosaminidase 77 kDa form]	82 kDa	0.16	13	7	6	2	1	4	0	1	0	2	3	-1.016370031	0% (0.59)
ACSF3_HUMAN	PA1B3_HUMAN	PAFAH1B3	Q15102	Platelet-activating factor acetylhydrolase IB subunit gamma (EC 3.1.1.47) (PAF acetylhydrolase 29 kDa subunit) (PAF-AH 29 kDa subunit) (PAF-AH subunit gamma) (PAFAH subunit gamma)	26 kDa	0.1	13	7	6	0	1	3	3	0	3	2	1	-1.016370031	0% (0.59)
IF2A_HUMAN	LTOR2_HUMAN	LAMTOR2	Q9Y2Q5	Ragulator complex protein LAMTOR2 (Endosomal adaptor protein p14) (Late endosomal/lysosomal Mp1-interacting protein) (Late endosomal/lysosomal adaptor and MAPK and MTOR activator 2) (Mitogen-activated protein-binding protein-interacting protein) (MAPBP-interacting protein)	14 kDa	0.37	13	7	6	0	4	1	2	1	1	2	2	-1.016370031	0% (0.59)

				(Roadblock domain-containing protein 3)															
RFC4_HUMAN	AL3B1_HUMAN	ALDH3B1	P43353	Aldehyde dehydrogenase family 3 member B1 (EC 1.2.1.5) (Aldehyde dehydrogenase 7)	52 kDa	0.11	13	7	6	2	0	0	3	0	0	2	0	-1.016370031	0% (0.59)
DPP3_HUMAN (+3)	YMEL1_HUMAN	YME1L1	Q96TA2	ATP-dependent zinc metalloprotease YME1L1 (EC 3.4.24.-) (ATP-dependent metalloprotease FtsH1) (Meg-4) (Presenilin-associated metalloprotease) (PAMP) (YME1-like protein 1)	86 kDa	0.09	28	15	13	5	5	3	2	7	0	3	3	-1.018533501	0% (0.55)
LAMA5_HUMAN	CQ062_HUMAN	C17orf62	Q9BQA9	Uncharacterized protein C17orf62	21 kDa	0.0076	28	15	13	4	3	2	6	0	7	2	4	-1.018533501	0% (0.55)
SPT6H_HUMAN	PPT1_HUMAN	PPT1	P50897	Palmitoyl-protein thioesterase 1 (PPT-1) (EC 3.1.2.22) (Palmitoyl-protein hydrolase 1)	34 kDa	0.052	27	15	13	2	6	4	3	2	1	4	6	-1.018533501	0% (0.55)
EMC4_HUMAN (+1)	RMD1_HUMAN	RMDN1	Q96DB5	Regulator of microtubule dynamics protein 1 (RMD-1) (hRMD-1) (Protein FAM82B)	36 kDa	0.21	28	15	13	6	3	2	4	5	0	6	2	-1.018533501	0% (0.55)
Q05BS6_HUMAN (+2)	NU188_HUMAN	NUP188	Q5SRE5	Nucleoporin NUP188 homolog (hNup188)	196 kDa	0.057	28	15	13	2	2	6	5	1	1	10	1	-1.018533501	0% (0.55)
ERH_HUMAN	A8K309_HUMAN		A8K309	cDNA FLJ76654, highly similar to Homo sapiens CD44 antigen (homing function and Indian blood group system) (CD44), transcript variant 4, mRNA	39 kDa	0.32	28	15	13	2	5	6	2	0	3	7	3	-1.018533501	0% (0.55)
SIN3A_HUMAN	ALG1_HUMAN	ALG1	Q9BT22	Chitobiosyldiphosphodolichol beta-mannosyltransferase (EC 2.4.1.142) (Asparagine-linked glycosylation protein 1 homolog) (Beta-1,4-mannosyltransferase) (GDP-Man:GlcNAc2-PP-dolichol mannosyltransferase) (GDP-mannose-dolichol diphosphochitobiose mannosyltransferase) (Mannosyltransferase-1) (MT-1) (hMat-1)	53 kDa	0.06	43	23	20	9	5	3	6	2	4	5	9	-1.019273033	0% (0.53)
J3QQX2_HUMAN	OXLA_HUMAN	IL4I1	Q96RQ9	L-amino-acid oxidase (LAAO) (LAO) (EC 1.4.3.2) (Interleukin-4-induced protein 1) (IL4-induced protein 1) (Protein Fig-1) (hFIG1)	63 kDa	0.19	73	39	34	13	3	6	17	0	14	7	14	-1.019873499	0% (0.51)
A8K813_HUMAN (+2)	J3KN69_HUMAN	NCEH1	J3KN69	Arylacetamide deacetylase-like 1 (Neutral cholesterol ester hydrolase 1)	49 kDa	0.1	133	71	62	25	17	21	9	3	12	14	33	-1.020282654	0% (0.49)
A8K1D2_HUMAN (+1)	B3KQT9_HUMAN		B3KQT9	cDNA PSEC0175 fis, clone OVARC1000169, highly similar to Protein disulfide-isomerase A3 (EC 5.3.4.1)	54 kDa	0.16	527	284	248	65	80	74	65	35	53	75	84	-1.022257507	0% (0.43)
B4DEK4_HUMAN (+1)	A8K8U1_HUMAN		A8K8U1	cDNA FLJ77762, highly similar to Homo sapiens cullin-associated and neddylation-dissociated 1 (CAND1), mRNA	136 kDa	0.18	86	46	40	7	11	13	16	11	10	13	6	-1.02310211	0% (0.50)
HSDL1_HUMAN (+1)	MOES_HUMAN	MSN	P26038	Moesin (Membrane-organizing extension spike protein)	68 kDa	0.14	172	93	81	16	33	26	19	15	21	23	22	-1.023511437	0% (0.48)
NOP9_HUMAN	ACSS3_HUMAN	ACSS3	Q9H6R3	Acyl-CoA synthetase short-chain family member 3, mitochondrial (EC 6.2.1.1) (Acyl-CoA synthetase short-chain family member 3)	75 kDa	0.072	56	30	26	16	2	2	10	7	0	9	10	-1.024289728	0% (0.52)
TRNT1_HUMAN	A4QPB0_HUMAN	IQGAP1	A4QPB0	IQ motif containing GTPase activating protein 1	189 kDa	0.1	329	177	154	41	39	44	52	23	36	56	40	-1.025552454	0% (0.44)
RETST_HUMAN	Q9BTQ7_HUMAN		Q9BTQ7	Similar to ribosomal protein L23 (Fragment)	14 kDa	0.11	83	45	39	11	16	7	11	12	9	8	10	-1.026331458	0% (0.50)
F8W914_HUMAN (+1)	PTN1_HUMAN	PTPN1	P18031	Tyrosine-protein phosphatase non-receptor type 1 (EC	50 kDa	0.27	69	37	32	6	11	15	5	5	8	11	8	-1.027497251	0% (0.50)

				3.1.3.48) (Protein-tyrosine phosphatase 1B) (PTP-1B)															
RU2B_HUMAN	PSDE_HUMAN	PSMD14	O00487	26S proteasome non-ATPase regulatory subunit 14 (EC 3.4.19.-) (26S proteasome regulatory subunit RPN11) (26S proteasome-associated PAD1 homolog 1)	35 kDa	0.12	26	14	12	1	5	2	6	4	5	2	1	-1.02799606	0% (0.54)
JAGN1_HUMAN	AGR2_HUMAN	AGR2	O95994	Anterior gradient protein 2 homolog (AG-2) (hAG-2) (HPC8) (Secreted cement gland protein XAG-2 homolog)	20 kDa	0.14	26	14	12	10	1	0	4	1	3	6	2	-1.02799606	0% (0.54)
Q53HP9_HUMAN	PSB1_HUMAN	PSMB1	P20618	Proteasome subunit beta type-1 (EC 3.4.25.1) (Macropain subunit C5) (Multicatalytic endopeptidase complex subunit C5) (Proteasome component C5) (Proteasome gamma chain)	26 kDa	0.12	26	14	12	3	4	3	4	3	2	5	2	-1.02799606	0% (0.54)
B4DTY9_HUMAN (+3)	RDH14_HUMAN	RDH14	Q9HBH5	Retinol dehydrogenase 14 (EC 1.1.1.-) (Alcohol dehydrogenase PAN2)	37 kDa	0.071	26	14	12	6	2	1	5	4	1	3	4	-1.02799606	0% (0.54)
B3KX23_HUMAN (+1)	G6PE_HUMAN	H6PD	O95479	GDH/6PGL endoplasmic bifunctional protein [Includes: Glucose 1-dehydrogenase (EC 1.1.1.47) (Hexose-6-phosphate dehydrogenase); 6-phosphogluconolactonase (6PGL) (EC 3.1.1.31)]	89 kDa	0.095	54	29	25	7	5	7	10	7	3	6	10	-1.029285942	0% (0.51)
F8VXY3_HUMAN (+2)	RS14_HUMAN	RPS14	P62263	40S ribosomal protein S14	16 kDa	0.19	53	29	25	4	5	11	9	10	9	3	3	-1.029285942	0% (0.51)
U2AF1_HUMAN	ARM10_HUMAN	ARMC10	Q8N2F6	Armadillo repeat-containing protein 10 (Splicing variant involved in hepatocarcinogenesis protein)	38 kDa	0.12	82	44	38	10	13	12	9	11	5	12	10	-1.029725288	0% (0.49)
SURF1_HUMAN	B4DF70_HUMAN		B4DF70	cDNA FLJ60461, highly similar to Peroxiredoxin-2 (EC 1.11.1.15)	20 kDa	0.13	82	44	38	8	12	12	12	7	9	10	12	-1.029725288	0% (0.49)
A8K6F0_HUMAN (+1)	B4E2W0_HUMAN	HADHB	B4E2W0	3-ketoacyl-CoA thiolase (cDNA FLJ56214, highly similar to Trifunctional enzyme subunit beta, mitochondrial)	49 kDa	0.036	235	126	109	36	29	24	37	35	15	25	36	-1.030945418	0% (0.44)
Q5SYZ4_HUMAN (+1)	AMPL_HUMAN	LAP3	P28838	Cytosol aminopeptidase (EC 3.4.11.1) (Leucine aminopeptidase 3) (LAP-3) (Leucyl aminopeptidase) (Peptidase S) (Proline aminopeptidase) (EC 3.4.11.5) (Prolyl aminopeptidase)	56 kDa	0.23	207	111	96	15	24	23	50	23	27	28	17	-1.030981158	0% (0.45)
B2R5I8_HUMAN (+1)	ALDOA_HUMAN	ALDOA	P04075	Fructose-bisphosphate aldolase A (EC 4.1.2.13) (Lung cancer antigen NY-LU-1) (Muscle-type aldolase)	39 kDa	0.22	122	66	57	8	16	17	29	14	9	20	15	-1.031199941	0% (0.47)
H0YN88_HUMAN	AL3A2_HUMAN	ALDH3A2	P51648	Fatty aldehyde dehydrogenase (EC 1.2.1.3) (Aldehyde dehydrogenase 10) (Aldehyde dehydrogenase family 3 member A2) (Microsomal aldehyde dehydrogenase)	55 kDa	0.16	95	51	44	21	7	7	17	12	8	7	17	-1.031363468	0% (0.48)
TBB2B_HUMAN	2AAA_HUMAN	PPP2R1A	P30153	Serine/threonine-protein phosphatase 2A 65 kDa regulatory subunit A alpha isoform (Medium tumor antigen-associated 61 kDa protein) (PP2A subunit A isoform PR65-alpha) (PP2A subunit A isoform R1-alpha)	65 kDa	0.46	67	36	31	6	12	9	8	8	11	10	2	-1.031663593	0% (0.50)
VWA8_HUMAN	Q9BQQ5_HUMAN	L27a	Q9BQQ5	Ribosomal protein L27a	12 kDa	0.053	39	21	18	5	5	6	5	6	6	4	3	-1.032377052	0% (0.52)
E7EVP7_HUMAN (+1)	CN37_HUMAN	CNP	P09543	2',3'-cyclic-nucleotide 3'-phosphodiesterase (CNP) (CNPase) (EC 3.1.4.37)	48 kDa	0.045	11	6	5	2	0	0	5	0	4	1	0	-1.036083042	0% (0.58)
ABCBA_HUMAN	PPOX_HUMAN	PPOX	P50336	Protoporphyrinogen oxidase (PPO) (EC 1.3.3.4)	51 kDa	0.057	11	6	5	0	1	2	3	3	0	1	1	-1.036083042	0% (0.58)

AAPK1_HUMAN	A8K6G9_HUMAN		A8K6G9	cDNA FLJ76871, highly similar to Homo sapiens DEAH (Asp-Glu-Ala-His) box polypeptide 38 (DHX38), mRNA	140 kDa	0.12	12	6	5	0	3	2	1	2	1	2	0	- 1.0360830 42	0% (0.58)
E7ER89_HUMAN (+1)	TLR2_HUMAN	TLR2	O60603	Toll-like receptor 2 (Toll/interleukin-1 receptor-like protein 4) (CD antigen CD282)	90 kDa	0.14	11	6	5	1	4	1	0	0	1	3	1	- 1.0360830 42	0% (0.58)
HPT_HUMAN (+2)	A8K588_HUMAN		A8K588	cDNA FLJ76823, highly similar to Homo sapiens splicing factor, arginine/serine-rich 6 (SFRS6), mRNA	39 kDa	0.29	11	6	5	0	1	4	1	1	2	1	1	- 1.0360830 42	0% (0.58)
B3KP52_HUMAN (+2)	FND3B_HUMAN	FNDC3B	Q53EP0	Fibronectin type III domain-containing protein 3B (Factor for adipocyte differentiation 104) (HCV NS5A-binding protein 37)	133 kDa	0.098	11	6	5	2	2	2	0	0	0	3	2	- 1.0360830 42	0% (0.58)
K7ELC4_HUMAN (+4)	NU214_HUMAN	NUP214	P35658	Nuclear pore complex protein Nup214 (214 kDa nucleoporin) (Nucleoporin Nup214) (Protein CAN)	214 kDa	0.32	11	6	5	0	3	3	0	1	0	3	1	- 1.0360830 42	0% (0.58)
TFB2M_HUMAN	SNX3_HUMAN	SNX3	O60493	Sorting nexin-3 (Protein SDP3)	19 kDa	0.096	10	6	5	2	2	1	1	0	1	2	2	- 1.0360830 42	0% (0.58)
F8VUJ3_HUMAN	B4DXV9_HUMAN		B4DXV9	cDNA FLJ53678	46 kDa	0.047	11	6	5	2	2	2	0	4	0	0	1	- 1.0360830 42	0% (0.58)
PM14_HUMAN	B2R5U7_HUMAN		B2R5U7	cDNA, FLJ92633, highly similar to Homo sapiens CCAAT-box-binding transcription factor (CBF2), mRNA	114 kDa	0.3	11	6	5	0	2	3	1	5	0	0	0	- 1.0360830 42	0% (0.58)
IF2P_HUMAN (+1)	Q59G24_HUMAN		Q59G24	Activated RNA polymerase II transcription cofactor 4 variant (Fragment)	15 kDa	0.059	11	6	5	2	1	0	3	2	0	2	1	- 1.0360830 42	0% (0.58)
B4DMR0_HUMAN (+3)	PXMP2_HUMAN	PXMP2	Q9NR77	Peroxisomal membrane protein 2 (22 kDa peroxisomal membrane protein)	22 kDa	0.16	11	6	5	1	1	2	2	1	2	1	1	- 1.0360830 42	0% (0.58)
S27A3_HUMAN	B3KNQ2_HUMAN		B3KNQ2	cDNA FLJ30150 fis, clone BRACE2000300, highly similar to Transmembrane protein TPARL	16 kDa	0.047	11	6	5	2	2	1	1	1	0	3	1	- 1.0360830 42	0% (0.58)
AMPB_HUMAN (+1)	A8K7A4_HUMAN		A8K7A4	cDNA FLJ76904, highly similar to Homo sapiens methionine adenosyltransferase II, beta (MAT2B), transcript variant 2, mRNA	36 kDa	0.11	11	6	5	1	0	3	2	2	0	2	1	- 1.0360830 42	0% (0.58)
PCAT2_HUMAN	CELF1_HUMAN	CELF1	Q92879	CUGBP Elav-like family member 1 (CELF-1) (50 kDa nuclear polyadenylated RNA-binding protein) (Bruno-like protein 2) (CUG triplet repeat RNA-binding protein 1) (CUG-BP1) (CUG-BP- and ETR-3-like factor 1) (Deadenylation factor CUG-BP) (Embryo deadenylation element-binding protein homolog) (EDEN-BP homolog) (RNA-binding protein BRUNOL-2)	52 kDa	0.075	11	6	5	0	2	1	3	1	0	4	0	- 1.0360830 42	0% (0.58)
GALE_HUMAN	B4DWC4_HUMAN		B4DWC4	cDNA FLJ61180, highly similar to Chloride intracellular channel protein 4	28 kDa	0.2	11	6	5	1	1	2	2	3	1	0	0	- 1.0360830 42	0% (0.58)
NDUAC_HUMAN (+1)	RS13_HUMAN	RPS13	P62277	40S ribosomal protein S13	17 kDa	0.17	65	35	30	9	9	7	11	7	13	5	5	- 1.0360964 92	0% (0.49)
RM49_HUMAN	HIBCH_HUMAN	HIBCH	Q6NVY1	3-hydroxyisobutyryl-CoA hydrolase, mitochondrial (EC 3.1.2.4) (3-hydroxyisobutyryl-coenzyme A hydrolase) (HIB-CoA hydrolase) (HIBYL-CoA-H)	43 kDa	0.23	65	35	30	7	8	10	11	6	5	6	13	- 1.0360964 92	0% (0.49)
B4DSQ5_HUMAN (+2)	B3KM81_HUMAN		B3KM81	cDNA FLJ10471 fis, clone NT2RP2000045, highly similar to DnaJ homolog	50 kDa	0.12	65	35	30	7	9	7	12	5	11	5	9	- 1.0360964 92	0% (0.49)

				subfamily A member 3, mitochondrial															
DEST_HUMAN (+1)	RB12B_HUMAN	RBM12B	Q8IXT5	RNA-binding protein 12B (RNA-binding motif protein 12B)	118 kDa	0.19	24	13	11	2	3	3	5	3	0	6	2	-1.039003302	0% (0.53)
PEF1_HUMAN	PUF60_HUMAN	PUF60	Q9UHX1	Poly(U)-binding-splicing factor PUF60 (60 kDa poly(U)-binding-splicing factor) (FUSE-binding protein-interacting repressor) (FBP-interacting repressor) (Ro-binding protein 1) (RoBP1) (Siah-binding protein 1) (Siah-BP1)	60 kDa	0.085	25	13	11	0	4	6	3	2	1	7	1	-1.039003302	0% (0.53)
B7Z7N1_HUMAN (+3)	B4DXK8_HUMAN	UTP15	B4DXK8	U3 small nucleolar RNA-associated protein 15 homolog (cDNA FLJ57656, highly similar to U3 small nucleolar RNA-associated protein 15 homolog)	56 kDa	0.26	25	13	11	4	2	2	5	7	0	2	2	-1.039003302	0% (0.53)
B4DEI4_HUMAN (+2)	CATS_HUMAN	CTSS	P25774	Cathepsin S (EC 3.4.22.27)	37 kDa	0.064	24	13	11	0	7	5	0	0	0	3	8	-1.039003302	0% (0.53)
B7Z4S9_HUMAN (+2)	NDUA6_HUMAN	NDUFA6	P56556	NADH dehydrogenase [ubiquinone] 1 alpha subcomplex subunit 6 (Complex I-B14) (CI-B14) (LYR motif-containing protein 6) (NADH-ubiquinone oxidoreductase B14 subunit)	18 kDa	0.12	24	13	11	3	4	4	2	5	1	3	2	-1.039003302	0% (0.53)
A3R0T7_HUMAN (+2)	APOO_HUMAN	APOO	Q9BUR5	Apolipoprotein O (Protein FAM121B)	22 kDa	0.34	24	13	11	4	3	2	4	3	2	3	3	-1.039003302	0% (0.53)
B1AKZ3_HUMAN (+2)	B3KM95_HUMAN		B3KM95	Phosphatidate cytidylyltransferase (EC 2.7.7.41)	51 kDa	0.17	24	13	11	5	3	3	2	1	3	3	4	-1.039003302	0% (0.53)
H3BSH7_HUMAN	B4E175_HUMAN		B4E175	cDNA FLJ54775, highly similar to Syntaxin-binding protein 2	68 kDa	0.039	37	20	17	10	2	5	3	2	3	7	5	-1.040005878	0% (0.51)
PLBL1_HUMAN	A8K4T6_HUMAN		A8K4T6	cDNA FLJ76282, highly similar to Homo sapiens proteasome (prosome, macropain) 26S subunit, non-ATPase, 5 (PSMD5), mRNA	56 kDa	0.12	37	20	17	1	2	2	14	3	10	3	1	-1.040005878	0% (0.51)
AAKG1_HUMAN	ERMP1_HUMAN	ERMP1	Q7Z2K6	Endoplasmic reticulum metallopeptidase 1 (EC 3.4.-.-) (Felix-ina)	100 kDa	0.19	38	20	17	10	5	4	2	5	2	3	7	-1.040005878	0% (0.51)
RBM25_HUMAN	K7EPB2_HUMAN	PRKAR1A	K7EPB2	cAMP-dependent protein kinase type I-alpha regulatory subunit (Fragment)	34 kDa	0.039	37	20	17	5	3	3	9	5	4	7	1	-1.040005878	0% (0.51)
H7C579_HUMAN (+1)	A4D0R2_HUMAN	SYPL	A4D0R2	Synaptophysin-like 1, isoform CRA_b (Synaptophysin-like protein)	26 kDa	0.27	52	27	23	5	7	5	10	6	7	6	4	-1.040514206	0% (0.50)
R4RWU8_HUMAN	AL1B1_HUMAN	ALDH1B1	P30837	Aldehyde dehydrogenase X, mitochondrial (EC 1.2.1.3) (Aldehyde dehydrogenase 5) (Aldehyde dehydrogenase family 1 member B1)	57 kDa	0.42	76	41	35	22	9	5	5	19	3	4	10	-1.041029799	0% (0.48)
B4DKQ5_HUMAN (+1)	RS9_HUMAN	RPS9	P46781	40S ribosomal protein S9	23 kDa	0.18	102	55	47	14	14	13	14	13	13	11	9	-1.041292697	0% (0.46)
AGRIN_HUMAN	B4DUQ1_HUMAN		B4DUQ1	cDNA FLJ54552, highly similar to Heterogeneous nuclear ribonucleoprotein K	49 kDa	0.057	178	96	82	8	27	26	35	20	14	28	20	-1.043429523	0% (0.42)
D3DWV9_HUMAN (+5)	TPIS_HUMAN	TPI1	P60174	Triosephosphate isomerase (TIM) (EC 5.3.1.1) (Triose-phosphate isomerase)	31 kDa	0.1	137	74	63	11	18	13	33	16	29	11	7	-1.04613337	0% (0.43)
A8K7S8_HUMAN (+4)	LMAN1_HUMAN	LMAN1	P49257	Protein ERGIC-53 (ER-Golgi intermediate compartment 53 kDa protein) (Gp58) (Intracellular mannose-specific lectin MR60) (Lectin mannose-binding 1)	58 kDa	0.11	47	26	22	6	7	9	4	6	5	4	7	-1.046852565	0% (0.49)
B7Z954_HUMAN (+1)	B0QY89_HUMAN	EIF3L	B0QY89	Eukaryotic translation initiation factor 3 subunit L (eIF3l) (Eukaryotic translation initiation factor 3 subunit 6-interacting protein) (Eukaryotic translation	71 kDa	0.047	48	26	22	2	9	8	8	6	7	6	3	-1.046852565	0% (0.49)

				initiation factor 3 subunit E-interacting protein)															
STRN_HUMAN	Q8WVX7_HUMAN		Q8WVX7	Ribosomal protein S19 (Fragment)	17 kDa	0.063	48	26	22	6	6	7	7	8	8	3	3	-1.046852565	0% (0.49)
E9PF74_HUMAN (+3)	2B1G_HUMAN	HLA-DRB1	Q29974	HLA class II histocompatibility antigen, DRB1-16 beta chain (MHC class II antigen DRB1*16) (DR-16) (DR16)	30 kDa	0.23	48	26	22	0	7	7	0	0	4	0	10	-1.046852565	0% (0.49)
Q86UA3_HUMAN	AMPN_HUMAN	ANPEP	P15144	Aminopeptidase N (AP-N) (hAPN) (EC 3.4.11.2) (Alanyl aminopeptidase) (Aminopeptidase M) (AP-M) (Microsomal aminopeptidase) (Myeloid plasma membrane glycoprotein CD13) (gp150) (CD antigen CD13)	110 kDa	0.09	161	87	74	43	15	26	6	3	15	28	26	-1.04752819	0% (0.42)
EXOS2_HUMAN	AP2A1_HUMAN	AP2A1	O95782	AP-2 complex subunit alpha-1 (100 kDa coated vesicle protein A) (Adapter-related protein complex 2 subunit alpha-1) (Adaptor protein complex AP-2 subunit alpha-1) (Alpha-adaptin A) (Alpha1-adaptin) (Clathrin assembly protein complex 2 alpha-A large chain) (Plasma membrane adaptor HA2/AP2 adaptin alpha A subunit)	108 kDa	0.21	99	53	45	14	17	12	10	8	11	14	11	-1.047700025	0% (0.45)
A8K3B0_HUMAN (+2)	J3KN00_HUMAN	NDUFA13	J3KN00	NADH dehydrogenase (Ubiquinone) 1 alpha subcomplex, 13 (NADH dehydrogenase [ubiquinone] 1 alpha subcomplex subunit 13)	26 kDa	0.19	35	19	16	10	3	2	5	6	4	4	2	-1.048519047	0% (0.50)
A9CQZ8_HUMAN (+2)	A0JLQ5_HUMAN	BXDC2	A0JLQ5	BXDC2 protein (Fragment)	39 kDa	0.033	35	19	16	3	6	4	5	3	3	4	4	-1.048519047	0% (0.50)
SBP1_HUMAN	GCDH_HUMAN	GCDH	Q92947	Glutaryl-CoA dehydrogenase, mitochondrial (GCD) (EC 1.3.8.6)	48 kDa	0.17	35	19	16	4	3	2	10	9	0	1	6	-1.048519047	0% (0.50)
A8K4Q9_HUMAN (+2)	B4DLC3_HUMAN		B4DLC3	Claudin	18 kDa	0.13	35	19	16	5	5	4	6	7	0	2	7	-1.048519047	0% (0.50)
B3KQF4_HUMAN (+2)	RM09_HUMAN	MRPL9	Q9BYD2	39S ribosomal protein L9, mitochondrial (L9mt) (MRP-L9)	30 kDa	0.19	35	19	16	6	4	3	6	5	2	2	7	-1.048519047	0% (0.50)
F8VY86_HUMAN (+1)	B4DEK2_HUMAN		B4DEK2	cDNA FLJ59182, highly similar to Splicing factor, arginine/serine-rich 7	19 kDa	0.037	35	19	16	4	5	4	6	7	3	3	2	-1.048519047	0% (0.50)
B4E259_HUMAN	SP16H_HUMAN	SUPT16H	Q9Y5B9	FACT complex subunit SPT16 (Chromatin-specific transcription elongation factor 140 kDa subunit) (FACT 140 kDa subunit) (FACTp140) (Facilitates chromatin transcription complex subunit SPT16) (hSPT16)	120 kDa	0.11	85	46	39	6	14	10	16	26	4	7	3	-1.048535887	0% (0.46)
COTL1_HUMAN	U520_HUMAN	SNRNP200	O75643	U5 small nuclear ribonucleoprotein 200 kDa helicase (EC 3.6.4.13) (Activating signal cointegrator 1 complex subunit 3-like 1) (BRR2 homolog) (U5 snRNP-specific 200 kDa protein) (U5-200KD)	245 kDa	0.2	259	140	119	23	43	47	27	29	11	50	30	-1.049241664	0% (0.38)
ABCE1_HUMAN	DDB1_HUMAN	DDB1	Q16531	DNA damage-binding protein 1 (DDB p127 subunit) (DNA damage-binding protein a) (DDBa) (Damage-specific DNA-binding protein 1) (HBV X-associated protein 1) (XAP-1) (UV-damaged DNA-binding factor) (UV-damaged DNA-binding protein 1) (UV-DDB 1) (XPE-binding factor) (XPE-BF) (Xeroderma pigmentosum group E-complementing protein) (XPCe)	127 kDa	0.11	107	59	50	11	16	17	15	19	2	23	4	-1.05006063	0% (0.44)

E7EMW7_HUMAN (+2)	HSP71_HUMAN	HSPA1A	P08107	Heat shock 70 kDa protein 1A/1B (Heat shock 70 kDa protein 1/2) (HSP70-1/HSP70-2) (HSP70.1/HSP70.2)	70 kDa	0.016	415	225	191	40	66	55	64	53	36	64	40	-1.051281424	0% (0.34)
SRRM2_HUMAN	D3DQ69_HUMAN	SERBP1	D3DQ69	SERPINE1 mRNA binding protein 1, isoform CRA_c	49 kDa	0.011	22	12	10	2	2	5	4	4	0	3	3	-1.051967143	0% (0.53)
E9PFA8_HUMAN (+4)	XPR1_HUMAN	XPR1	Q9UBH6	Xenotropic and polytropic retrovirus receptor 1 (Protein SYG1 homolog) (Xenotropic and polytropic murine leukemia virus receptor X3) (X-receptor)	82 kDa	0.073	22	12	10	1	5	4	2	3	2	3	2	-1.051967143	0% (0.53)
CAPR1_HUMAN	O19707_HUMAN	HLA-DQB1	O19707	MHC class II HLA-DQ-beta-1 (Fragment)	26 kDa	0.048	21	12	10	1	4	3	3	0	2	1	7	-1.051967143	0% (0.53)
B4E0U6_HUMAN (+2)	SERPH_HUMAN	SERPINH1	P50454	Serpin H1 (47 kDa heat shock protein) (Arsenic-transactivated protein 3) (AsTP3) (Cell proliferation-inducing gene 14 protein) (Collagen-binding protein) (Colligin) (Rheumatoid arthritis-related antigen RA-A47)	46 kDa	0.11	315	171	145	38	53	43	38	33	35	39	38	-1.052073535	0% (0.36)
A8K521_HUMAN	ASPH_HUMAN	ASPH	Q12797	Aspartyl/asparaginyl beta-hydroxylase (EC 1.14.11.16) (Aspartate beta-hydroxylase) (ASP beta-hydroxylase) (Peptide-aspartate beta-dioxygenase)	86 kDa	0.084	83	45	38	20	12	7	6	9	7	11	11	-1.052495178	0% (0.45)
S2540_HUMAN	B3KRY3_HUMAN		B3KRY3	cDNA FLJ35079 fis, clone PLACE6005283, highly similar to Lysosome-associated membrane glycoprotein 1	43 kDa	0.15	45	25	21	7	6	6	6	5	1	5	10	-1.053760542	0% (0.48)
AGO2_HUMAN	A7E2Y5_HUMAN	DNAJC13	A7E2Y5	DnaJ (Hsp40) homolog, subfamily C, member 13	254 kDa	0.1	129	70	59	25	21	19	5	15	6	21	17	-1.056292441	0% (0.42)
LRC47_HUMAN	ATP5H_HUMAN	ATP5H	O75947	ATP synthase subunit d, mitochondrial (ATPase subunit d)	18 kDa	0.14	57	31	26	6	7	6	12	3	6	5	12	-1.057080585	0% (0.47)
B4DWA0_HUMAN (+1)	SYDM_HUMAN	DARS2	Q6PI48	Aspartate--tRNA ligase, mitochondrial (EC 6.1.1.12) (Aspartyl-tRNA synthetase) (AspRS)	74 kDa	0.076	58	31	26	8	12	5	7	12	1	6	7	-1.057080585	0% (0.47)
B4E1L5_HUMAN (+1)	OCAD1_HUMAN	OCIAD1	Q9NX40	OCIA domain-containing protein 1 (Ovarian carcinoma immunoreactive antigen)	28 kDa	0.14	57	31	26	13	6	3	9	3	10	6	7	-1.057080585	0% (0.47)
USMG5_HUMAN	LYAG_HUMAN	GAA	P10253	Lysosomal alpha-glucosidase (EC 3.2.1.20) (Acid maltase) (Aglucosidase alfa) [Cleaved into: 76 kDa lysosomal alpha-glucosidase; 70 kDa lysosomal alpha-glucosidase]	105 kDa	0.43	57	31	26	4	13	7	7	7	6	8	5	-1.057080585	0% (0.47)
IVD_HUMAN (+2)	MESD_HUMAN	MESDC2	Q14696	LDLR chaperone MESD (Mesoderm development candidate 2) (Mesoderm development protein) (Renal carcinoma antigen NY-REN-61)	26 kDa	0.14	34	18	15	4	6	2	7	1	3	4	7	-1.058079821	0% (0.50)
MCA3_HUMAN	Q5QPL9_HUMAN	RALY	Q5QPL9	RNA-binding protein Raly (Fragment)	25 kDa	0.19	33	18	15	4	4	5	6	4	3	3	5	-1.058079821	0% (0.50)
TI23B_HUMAN	F8VQX6_HUMAN	METTL7A	F8VQX6	Methyltransferase-like protein 7A (Fragment)	21 kDa	0.11	33	18	15	4	2	5	7	4	4	4	3	-1.058079821	0% (0.50)
A8K800_HUMAN (+4)	ASNA_HUMAN	ASNA1	O43681	ATPase ASNA1 (EC 3.6.-.-) (Arsenical pump-driving ATPase) (Arsenite-stimulated ATPase) (Transmembrane domain recognition complex 40 kDa ATPase subunit) (hARSA-I) (hASNA-I)	39 kDa	0.13	33	18	15	5	4	3	6	6	0	5	4	-1.058079821	0% (0.50)
B7Z587_HUMAN (+1)	B4DH58_HUMAN		B4DH58	cDNA FLJ56344, highly similar to Implantation-associated protein	41 kDa	0.18	33	18	15	7	6	3	3	1	7	3	5	-1.058079821	0% (0.50)

B2R4C1_HUMAN (+1)	PECA1_HUMAN	PECAM1	P16284	Platelet endothelial cell adhesion molecule (PECAM-1) (EndoCAM) (GPIIA') (PECA1) (CD antigen CD31)	83 kDa	0.18	33	18	15	3	7	5	3	0	4	7	4	-1.058079821	0% (0.50)
A8K4M4_HUMAN (+1)	COX41_HUMAN	COX4I1	P13073	Cytochrome c oxidase subunit 4 isoform 1, mitochondrial (Cytochrome c oxidase polypeptide IV) (Cytochrome c oxidase subunit IV isoform 1) (COX IV-1)	20 kDa	0.076	90	50	42	12	13	10	15	13	8	9	11	-1.058421637	0% (0.43)
K7ERC8_HUMAN	E7ENR4_HUMAN	HK1	E7ENR4	Hexokinase-1	106 kDa	0.21	160	87	73	16	26	24	22	10	32	19	13	-1.061651601	0% (0.39)
GOGA2_HUMAN	Q7Z7M4_HUMAN	SOD2	Q7Z7M4	Superoxide dismutase (EC 1.15.1.1) (Fragment)	24 kDa	0.09	252	137	115	34	42	45	16	7	37	24	48	-1.062313245	0% (0.35)
MAGD2_HUMAN	ENPL_HUMAN	HSP90B1	P14625	Endoplasmin (94 kDa glucose-regulated protein) (GRP-94) (Heat shock protein 90 kDa beta member 1) (Tumor rejection antigen 1) (gp96 homolog)	92 kDa	0.059	652	356	299	83	114	101	60	37	52	105	104	-1.063022806	0% (0.24)
H3BN55_HUMAN (+1)	TTL12_HUMAN	TTLL12	Q14166	Tubulin--tyrosine ligase-like protein 12	74 kDa	0.36	8	5	4	0	1	0	3	1	3	0	0	-1.063305248	0% (0.57)
WDR61_HUMAN	LAP2_HUMAN	ERBB2IP	Q96RT1	Protein LAP2 (Densin-180-like protein) (Erbb2-interacting protein) (Erbin)	158 kDa	0.15	9	5	4	3	0	2	0	1	0	2	1	-1.063305248	0% (0.57)
B2R802_HUMAN (+1)	CDC73_HUMAN	CDC73	Q6P1J9	Parafibromin (Cell division cycle protein 73 homolog) (Hyperparathyroidism 2 protein)	61 kDa	0.17	9	5	4	0	1	2	2	1	1	2	0	-1.063305248	0% (0.57)
IF4A2_HUMAN	B1ALA9_HUMAN	PRPS1	B1ALA9	Ribose-phosphate pyrophosphokinase 1	24 kDa	0.36	9	5	4	0	4	1	0	0	4	0	0	-1.063305248	0% (0.57)
K0020_HUMAN	A8K2N5_HUMAN		A8K2N5	Integrin beta	86 kDa	0.052	9	5	4	1	2	2	0	0	1	0	3	-1.063305248	0% (0.57)
B4E2W6_HUMAN (+1)	CATH_HUMAN	CTSH	P09668	Pro-cathepsin H [Cleaved into: Cathepsin H mini chain; Cathepsin H (EC 3.4.22.16); Cathepsin H heavy chain; Cathepsin H light chain]	37 kDa	0.045	9	5	4	0	2	3	0	0	0	3	1	-1.063305248	0% (0.57)
CO6A1_HUMAN	I2BPL_HUMAN	IRF2BPL	Q9H1B7	Interferon regulatory factor 2-binding protein-like (Enhanced at puberty protein 1)	83 kDa	0.071	9	5	4	0	2	2	1	1	0	3	1	-1.063305248	0% (0.57)
MYEF2_HUMAN	PLRKT_HUMAN	PLGRKT	Q9HBL7	Plasminogen receptor (KT) (Plg-R(KT))	17 kDa	0.075	9	5	4	3	1	0	1	0	2	0	2	-1.063305248	0% (0.57)
MX2_HUMAN	C9JZ87_HUMAN	TMEM106B	C9JZ87	Transmembrane protein 106B (Fragment)	12 kDa	0.24	9	5	4	5	0	0	0	0	1	1	2	-1.063305248	0% (0.57)
IDI1_HUMAN	TF3C4_HUMAN	GTF3C4	Q9UKN8	General transcription factor 3C polypeptide 4 (EC 2.3.1.48) (TF3C-delta) (Transcription factor IIIC 90 kDa subunit) (TFIIIC 90 kDa subunit) (TFIIIC90) (Transcription factor IIIC subunit delta)	92 kDa	0.093	9	5	4	0	0	3	2	2	0	1	0	-1.063305248	0% (0.57)
WDR43_HUMAN	C9JTZ6_HUMAN	NT5DC2	C9JTZ6	5'-nucleotidase domain-containing protein 2	61 kDa	0.034	9	5	4	0	2	3	0	2	0	2	0	-1.063305248	0% (0.57)
E9PH70_HUMAN (+1)	B3KW23_HUMAN		B3KW23	cDNA FLJ41961 fis, clone PUAEN2004083, highly similar to Nucleoporin p58/p45	59 kDa	0.048	9	5	4	0	0	2	3	0	0	3	1	-1.063305248	0% (0.57)
TM9S4_HUMAN	RT31_HUMAN	MRPS31	Q92665	28S ribosomal protein S31, mitochondrial (MRP-S31) (S31mt) (Imogen 38)	45 kDa	0.047	9	5	4	3	0	1	1	1	0	0	3	-1.063305248	0% (0.57)
CAB39_HUMAN	B2R4F3_HUMAN		B2R4F3	cDNA, FLJ92068, highly similar to Homo sapiens Rho GDP dissociation inhibitor (GDI) beta (ARHGDIB),mRNA	23 kDa	0.097	9	5	4	1	2	1	1	1	1	1	1	-1.063305248	0% (0.57)
B1PL87_HUMAN (+3)	B5MCC7_HUMAN	ACP1	B5MCC7	Low molecular weight phosphotyrosine protein phosphatase	12 kDa	0.22	10	5	4	0	2	1	2	1	2	1	0	-1.063305248	0% (0.57)

B4DE16_HUMAN (+2)	RM14_HUMAN	MRPL14	Q6P1L8	39S ribosomal protein L14, mitochondrial (L14mt) (MRP-L14) (39S ribosomal protein L32, mitochondrial) (L32mt) (MRP-L32)	16 kDa	0.082	9	5	4	2	2	1	0	2	0	0	2	-1.063305248	0% (0.57)
B7ZKL3_HUMAN	B4DFY5_HUMAN	MAP2K1	B4DFY5	Mitogen-activated protein kinase kinase 1, isoform CRA_d (cDNA FLJ58770, highly similar to Dual specificity mitogen-activated protein kinase kinase 1 (EC 2.7.12.2))	41 kDa	0.13	9	5	4	0	2	0	3	0	2	2	0	-1.063305248	0% (0.57)
PCKGC_HUMAN	B4DIB1_HUMAN		B4DIB1	cDNA FLJ55065, highly similar to Integral membrane protein 2C	25 kDa	0.25	9	5	4	1	1	3	0	4	0	0	0	-1.063305248	0% (0.57)
B4DDM6_HUMAN (+2)	COX20_HUMAN	COX20	Q5RI15	Cytochrome c oxidase protein 20 homolog	13 kDa	0.13	9	5	4	1	1	1	2	1	1	1	1	-1.063305248	0% (0.57)
RT25_HUMAN	LYPL1_HUMAN	LYPLAL1	Q5VWZ2	Lysophospholipase-like protein 1 (EC 3.1.2.-)	26 kDa	0.21	9	5	4	0	2	2	1	3	0	0	1	-1.063305248	0% (0.57)
Q59F44_HUMAN	E9PBR6_HUMAN	TMEM201	E9PBR6	Transmembrane protein 201	70 kDa	0.2	9	5	4	4	0	0	1	2	0	0	2	-1.063305248	0% (0.57)
A8MV58_HUMAN (+1)	CATD_HUMAN	CTSD	P07339	Cathepsin D (EC 3.4.23.5) [Cleaved into: Cathepsin D light chain; Cathepsin D heavy chain]	45 kDa	0.039	359	196	164	53	46	50	47	18	28	55	63	-1.066299358	0% (0.30)
A2RTY6_HUMAN (+3)	APOA1_HUMAN	APOA1	P02647	Apolipoprotein A-I (Apo-AI) (ApoA-I) (Apolipoprotein A1) [Cleaved into: Truncated apolipoprotein A-I (Apolipoprotein A-I(1-242))]	31 kDa	0.055	20	11	9	1	2	0	8	1	1	0	7	-1.067460246	0% (0.52)
SF01_HUMAN	HPRT_HUMAN	HPRT1	P00492	Hypoxanthine-guanine phosphoribosyltransferase (HGPRT) (HGPRTase) (EC 2.4.2.8)	25 kDa	0.045	20	11	9	0	1	4	6	5	2	2	0	-1.067460246	0% (0.52)
CACP_HUMAN	Q86XM2_HUMAN	ADD1	Q86XM2	ADD1 protein (Adducin 1 (Alpha)) (Alpha-adducin)	73 kDa	0.12	20	11	9	1	5	3	2	1	1	5	2	-1.067460246	0% (0.52)
MET7B_HUMAN	URP2_HUMAN	FERMT3	Q86UX7	Fermitin family homolog 3 (Kindlin-3) (MIG2-like protein) (Unc-112-related protein 2)	76 kDa	0.16	19	11	9	0	2	8	1	0	4	1	4	-1.067460246	0% (0.52)
F13A_HUMAN	A8K7S8_HUMAN		A8K7S8	cDNA FLJ75165, highly similar to Homo sapiens BCL2-antagonist/killer 1 (BAK1), mRNA	23 kDa	0.12	20	11	9	1	2	3	5	2	1	3	3	-1.067460246	0% (0.52)
IFIT2_HUMAN (+2)	MCA3_HUMAN	EEF1E1	O43324	Eukaryotic translation elongation factor 1 epsilon-1 (Aminoacyl tRNA synthetase complex-interacting multifunctional protein 3) (Elongation factor p18) (Multisynthase complex auxiliary component p18)	20 kDa	0.12	20	11	9	1	2	2	6	2	3	2	2	-1.067460246	0% (0.52)
PTPM1_HUMAN	NAMPT_HUMAN	NAMPT	P43490	Nicotinamide phosphoribosyltransferase (NAmPRTase) (Nampt) (EC 2.4.2.12) (Pre-B-cell colony-enhancing factor 1) (Pre-B cell-enhancing factor) (Visfatin)	56 kDa	0.27	108	60	50	3	21	24	12	7	19	16	9	-1.067502748	0% (0.40)
CYB5B_HUMAN (+5)	RL11_HUMAN	RPL11	P62913	60S ribosomal protein L11 (CLL-associated antigen KW-12)	20 kDa	0.089	31	17	14	4	4	4	5	5	4	2	3	-1.068894287	0% (0.49)
1433T_HUMAN	B2RBB2_HUMAN		B2RBB2	cDNA, FLJ95416, highly similar to Homo sapiens phosphatidylinositol glycan, class S (PIGS), mRNA	62 kDa	0.22	31	17	14	7	2	2	6	5	1	1	7	-1.068894287	0% (0.49)
B4DJV9_HUMAN (+2)	DEK_HUMAN	DEK	P35659	Protein DEK	43 kDa	0.095	42	23	19	2	6	7	8	5	5	5	4	-1.069622894	0% (0.47)
B4DRW3_HUMAN	MIRO2_HUMAN	RHOT2	Q8IXI1	Mitochondrial Rho GTPase 2 (MIRO-2) (hMiro-2) (EC 3.6.5.-) (Ras homolog gene family member T2)	68 kDa	0.091	42	23	19	8	6	6	3	3	2	8	6	-1.069622894	0% (0.47)

FABP5_HUMAN	RADI_HUMAN	RDX	P35241	Radixin	69 kDa	0.29	73	41	34	9	12	11	10	0	0	0	8	-1.070577966	0% (0.43)
STX8_HUMAN	RAP1B_HUMAN	RAP1B	P61224	Ras-related protein Rap-1b (GTP-binding protein smg p21B)	21 kDa	0.18	97	53	44	13	11	17	11	10	13	14	7	-1.07086903	0% (0.41)
B2R983_HUMAN (+1)	FACE1_HUMAN	ZMPSTE24	O75844	CAAX prenyl protease 1 homolog (EC 3.4.24.84) (Farnesylated proteins-converting enzyme 1) (FACE-1) (Prenyl protein-specific endoprotease 1) (Zinc metalloproteinase Ste24 homolog)	55 kDa	0.2	128	71	59	17	19	15	20	17	12	12	17	-1.071131372	0% (0.38)
LPCT4_HUMAN	B2RA56_HUMAN		B2RA56	cDNA, FLJ94705, highly similar to Homo sapiens nicalin (LOC56926), mRNA	62 kDa	0.12	125	70	58	32	9	10	21	7	15	14	22	-1.074135625	0% (0.38)
Q5LJA0_HUMAN (+1)	Q29849_HUMAN		Q29849	Lymphocyte antigen	40 kDa	0.12	106	58	48	17	0	0	0	0	9	0	19	-1.074583662	0% (0.39)
A8K6G9_HUMAN (+2)	A8K787_HUMAN		A8K787	cDNA FLJ75273, highly similar to Homo sapiens solute carrier family 25 (mitochondrial carrier; adenine nucleotide translocator), member 4, mRNA	33 kDa	0.032	338	182	151	49	43	36	56	35	44	38	35	-1.075210202	0% (0.28)
ARF6_HUMAN (+1)	A8K3A8_HUMAN		A8K3A8	cDNA FLJ75085, highly similar to Homo sapiens glutaminyl-tRNA synthetase (QARS), mRNA	88 kDa	0.18	62	34	28	6	9	9	10	6	9	8	4	-1.076421546	0% (0.43)
B2R9L0_HUMAN (+3)	Q96MH4_HUMAN		Q96MH4	cDNA FLJ32377 fis, clone SKMUS1000014, highly similar to Polyubiquitin 9	44 kDa	0.15	126	69	57	21	17	16	18	16	15	12	14	-1.077242981	0% (0.37)
AN32A_HUMAN (+3)	HNRPD_HUMAN	HNRNPD	Q14103	Heterogeneous nuclear ribonucleoprotein D0 (hnRNP D0) (AU-rich element RNA-binding protein 1)	38 kDa	0.25	126	69	57	9	17	22	21	14	10	25	8	-1.077242981	0% (0.37)
A8K964_HUMAN (+1)	B3KNK5_HUMAN		B3KNK5	cDNA FLJ14795 fis, clone NT2RP4001219, highly similar to Homo sapiens DnaJ (Hsp40) homolog, subfamily C, member 10 (DNAJC10), mRNA	69 kDa	0.077	40	22	18	9	2	6	5	3	3	2	10	-1.078789884	0% (0.46)
LSM1_HUMAN	DAD1_HUMAN	DAD1	P61803	Dolichyl-diphosphooligosaccharide--protein glycosyltransferase subunit DAD1 (Oligosaccharyl transferase subunit DAD1) (EC 2.4.99.18) (Defender against cell death 1) (DAD-1)	12 kDa	0.3	40	22	18	7	4	5	7	5	4	4	5	-1.078789884	0% (0.46)
Q9H9B7_HUMAN	H11_HUMAN	HIST1H1A	Q02539	Histone H1.1 (Histone H1a)	22 kDa	0.11	40	22	18	0	0	0	13	0	0	0	5	-1.078789884	0% (0.46)
E9PHY8_HUMAN (+1)	TPM1_HUMAN	TPM1	P09493	Tropomyosin alpha-1 chain (Alpha-tropomyosin) (Tropomyosin-1)	33 kDa	0.047	39	22	18	4	0	7	6	0	0	5	0	-1.078789884	0% (0.46)
FDFT_HUMAN	B4DY09_HUMAN		B4DY09	cDNA FLJ51660, highly similar to Interleukin enhancer-binding factor 2	39 kDa	0.2	210	115	95	21	33	26	36	26	9	28	34	-1.078884166	0% (0.32)
BRE_HUMAN (+1)	Q53G99_HUMAN		Q53G99	Beta actin variant (Fragment)	42 kDa	0.073	675	371	307	90	93	90	99	65	77	98	70	-1.078993616	0% (0.18)
SEH1_HUMAN	B4DJV2_HUMAN	CS	B4DJV2	Citrate synthase	50 kDa	0.064	228	126	104	28	36	29	32	45	16	20	23	-1.07999924	0% (0.31)
C9JL85_HUMAN	C9J9K3_HUMAN	RPSA	C9J9K3	40S ribosomal protein SA (Fragment)	30 kDa	0.52	180	97	80	15	24	28	30	21	24	22	15	-1.080152407	0% (0.34)
Q59FB9_HUMAN (+1)	FCL_HUMAN	TSTA3	Q13630	GDP-L-fucose synthase (EC 1.1.1.271) (GDP-4-keto-6-deoxy-D-mannose-3,5-epimerase-4-reductase) (Protein FX) (Red cell NADP(H)-binding protein) (Short-chain dehydrogenase/reductase family 4E member 1)	36 kDa	0.12	29	16	13	1	2	2	11	3	7	3	0	-1.08122638	0% (0.48)

SRRT_HUMAN	CLD3_HUMAN	CLDN3	O15551	Claudin-3 (Clostridium perfringens enterotoxin receptor 2) (CPE-R 2) (CPE-receptor 2) (Rat ventral prostate.1 protein homolog) (hRVP1)	23 kDa	0.026	29	16	13	5	2	2	7	6	1	3	3	-1.08122638	0% (0.48)
B5MCF9_HUMAN (+1)	D6RA47_HUMAN	MFSD10	D6RA47	Major facilitator superfamily domain-containing protein 10	39 kDa	0.089	29	16	13	8	4	2	2	1	3	4	5	-1.08122638	0% (0.48)
B7ZA00_HUMAN	MGST3_HUMAN	MGST3	O14880	Microsomal glutathione S-transferase 3 (Microsomal GST-3) (EC 2.5.1.18) (Microsomal GST-III)	17 kDa	0.089	71	39	32	14	8	8	9	8	9	7	8	-1.081250394	0% (0.41)
F5GZS0_HUMAN	A8K7N0_HUMAN		A8K7N0	cDNA FLJ75556, highly similar to Homo sapiens ribosomal protein L14, mRNA	24 kDa	0.056	60	33	27	8	7	9	9	9	8	6	4	-1.082908898	0% (0.42)
RFA1_HUMAN	M0QWZ7_HUMAN	SARS2	M0QWZ7	Serine--tRNA ligase, mitochondrial (Seryl-tRNA synthetase 2, isoform CRA_d)	58 kDa	0.12	59	33	27	11	8	5	9	9	4	8	6	-1.082908898	0% (0.42)
J3QKR5_HUMAN	ANXA5_HUMAN	ANXA5	P08758	Annexin A5 (Anchorin CII) (Annexin V) (Annexin-5) (Calphobindin I) (CBP-I) (Endonexin II) (Lipocortin V) (Placental anticoagulant protein 4) (PP4) (Placental anticoagulant protein I) (PAP-I) (Thromboplastin inhibitor) (Vascular anticoagulant-alpha) (VAC-alpha)	36 kDa	0.068	329	181	149	37	48	47	49	29	33	35	52	-1.083594218	0% (0.26)
RRS1_HUMAN	PP1A_HUMAN	PPP1CA	P62136	Serine/threonine-protein phosphatase PP1-alpha catalytic subunit (PP-1A) (EC 3.1.3.16)	38 kDa	0.11	110	61	50	11	17	12	20	11	13	14	13	-1.084945314	0% (0.37)
MA1B1_HUMAN	ACOT9_HUMAN	ACOT9	Q9Y305	Acyl-coenzyme A thioesterase 9, mitochondrial (Acyl-CoA thioesterase 9) (EC 3.1.2.-) (Acyl-CoA thioester hydrolase 9)	50 kDa	0.09	80	44	36	17	12	10	5	0	11	7	18	-1.085043784	0% (0.40)
B3KS62_HUMAN (+1)	RAB4B_HUMAN	RAB4B	P61018	Ras-related protein Rab-4B	24 kDa	0.053	49	27	22	6	7	7	7	4	5	5	8	-1.085283122	0% (0.44)
A8KA19_HUMAN (+1)	B4DMF5_HUMAN		B4DMF5	Glutamate dehydrogenase	57 kDa	0.1	471	258	212	73	61	60	66	56	66	32	61	-1.0861245	0% (0.21)
A4FTY4_HUMAN	NDUB4_HUMAN	NDUFB4	O95168	NADH dehydrogenase [ubiquinone] 1 beta subcomplex subunit 4 (Complex I-B15) (CI-B15) (NADH-ubiquinone oxidoreductase B15 subunit)	15 kDa	0.24	18	10	8	3	2	2	3	3	2	2	1	-1.086302913	0% (0.51)
ARPC5_HUMAN	CYB5B_HUMAN	CYB5B	O43169	Cytochrome b5 type B (Cytochrome b5 outer mitochondrial membrane isoform)	16 kDa	0.26	18	10	8	3	1	4	2	1	4	1	2	-1.086302913	0% (0.51)
STX4_HUMAN	B7ZKM8_HUMAN	SEC24B	B7ZKM8	Protein transport protein Sec24B (SEC24B protein)	140 kDa	0.13	19	10	8	2	2	3	3	2	1	3	2	-1.086302913	0% (0.51)
EMC7_HUMAN (+1)	B2R6E5_HUMAN		B2R6E5	NADPH--cytochrome P450 reductase (EC 1.6.2.4)	77 kDa	0.12	191	105	86	40	25	21	20	18	16	23	30	-1.087793651	0% (0.31)
PWP2_HUMAN	H2AY_HUMAN	H2AFY	O75367	Core histone macro-H2A.1 (Histone macroH2A1) (mH2A1) (Histone H2A.y) (H2A/y) (Medulloblastoma antigen MU-MB-50.205)	40 kDa	0.064	323	177	145	66	35	33	43	37	32	36	40	-1.088804626	0% (0.25)
PP1B_HUMAN	B2RAR6_HUMAN		B2RAR6	cDNA, FLJ95068, highly similar to Homo sapiens eukaryotic translation elongation factor 1 delta (guanine nucleotide exchange protein) (EEF1D), transcript variant 1, mRNA	71 kDa	0.31	38	21	17	1	6	5	9	6	4	3	4	-1.088961326	0% (0.45)
RM17_HUMAN	B1AVU8_HUMAN	PSAP	B1AVU8	Saposin-D	62 kDa	0.2	57	32	26	9	10	8	5	2	3	9	11	-1.089872286	0% (0.42)
B4DZJ7_HUMAN (+2)	DSG2_HUMAN	DSG2	Q14126	Desmoglein-2 (Cadherin family member 5) (HDGC)	122 kDa	0.057	58	32	26	7	9	11	6	8	1	7	10	-1.089872286	0% (0.42)

NLRX1_HUMAN	Q504R6_HUMAN	RAB13	Q504R6	RAB13 protein (Fragment)	27 kDa	0.034	60	32	26	12	9	8	4	4	6	7	10	-1.089872286	0% (0.42)
SPTC2_HUMAN	SF3B1_HUMAN	SF3B1	O75533	Splicing factor 3B subunit 1 (Pre-mRNA-splicing factor SF3b 155 kDa subunit) (SF3b155) (Spliceosome-associated protein 155) (SAP 155)	146 kDa	0.08	98	54	44	13	13	12	16	3	7	21	13	-1.090622573	0% (0.37)
B7Z532_HUMAN	ACSL3_HUMAN	ACSL3	O95573	Long-chain-fatty-acid--CoA ligase 3 (EC 6.2.1.3) (Long-chain acyl-CoA synthetase 3) (LACS 3)	80 kDa	0.46	117	65	53	21	19	17	8	16	9	16	12	-1.090817469	0% (0.35)
ES1_HUMAN	C9JFR7_HUMAN	CYCS	C9JFR7	Cytochrome c (Fragment)	11 kDa	0.18	48	26	21	6	8	6	7	3	4	6	8	-1.093917859	0% (0.43)
PCCA_HUMAN	G3V203_HUMAN	RPL18	G3V203	60S ribosomal protein L18	19 kDa	0.11	47	26	21	7	4	5	10	6	7	3	5	-1.093917859	0% (0.43)
Q30077_HUMAN (+1)	A8K4W2_HUMAN		A8K4W2	cDNA FLJ78635, highly similar to Homo sapiens ATP synthase, H+ transporting, mitochondrial F0 complex, subunit b, isoform 1 (ATP5F1), transcript variant 1, mRNA	29 kDa	0.25	137	75	61	27	12	8	28	13	17	15	16	-1.094138415	0% (0.33)
E9PKP7_HUMAN	J3KTE4_HUMAN	RPL19	J3KTE4	Ribosomal protein L19	23 kDa	0.11	27	15	12	3	4	4	5	2	3	3	4	-1.095419714	0% (0.47)
MMS19_HUMAN	AL4A1_HUMAN	ALDH4A1	P30038	Delta-1-pyrroline-5-carboxylate dehydrogenase, mitochondrial (P5C dehydrogenase) (EC 1.2.1.88) (Aldehyde dehydrogenase family 4 member A1) (L-glutamate gamma-semialdehyde dehydrogenase)	62 kDa	0.088	27	15	12	2	7	5	1	6	0	1	5	-1.095419714	0% (0.47)
INADL_HUMAN	B2R7B5_HUMAN		B2R7B5	cDNA, FLJ93365, highly similar to Homo sapiens KH domain containing, RNA binding, signal transduction associated 1 (KHDRBS1), mRNA	48 kDa	0.042	27	15	12	3	4	4	4	3	1	6	2	-1.095419714	0% (0.47)
B2R858_HUMAN (+1)	CY24B_HUMAN	CYBB	P04839	Cytochrome b-245 heavy chain (EC 1.-.-.-) (CGD91-phox) (Cytochrome b(558) subunit beta) (Cytochrome b558 subunit beta) (Heme-binding membrane glycoprotein gp91phox) (NADPH oxidase 2) (Neutrophil cytochrome b 91 kDa polypeptide) (Superoxide-generating NADPH oxidase heavy chain subunit) (gp91-1) (gp91-phox) (p22 phagocyte B-cytochrome)	65 kDa	0.07	125	69	56	15	27	18	10	0	19	17	21	-1.096075232	0% (0.34)
B2R4Q7_HUMAN (+2)	RL7A_HUMAN	RPL7A	P62424	60S ribosomal protein L7a (PLA-X polypeptide) (Surfeit locus protein 3)	30 kDa	0.24	103	58	47	16	14	14	16	11	12	8	15	-1.096870615	0% (0.35)
B2R9T9_HUMAN (+1)	COPB_HUMAN	COPB1	P53618	Coatomer subunit beta (Beta-coat protein) (Beta-COP)	107 kDa	0.091	104	58	47	10	12	18	18	9	22	12	4	-1.096870615	0% (0.35)
SMD2_HUMAN	PSD11_HUMAN	PSMD11	O00231	26S proteasome non-ATPase regulatory subunit 11 (26S proteasome regulatory subunit RPN6) (26S proteasome regulatory subunit S9) (26S proteasome regulatory subunit p44.5)	47 kDa	0.33	56	31	25	3	8	8	12	5	11	4	6	-1.097366113	0% (0.41)
CAZA2_HUMAN (+1)	GNA13_HUMAN	GNA13	Q14344	Guanine nucleotide-binding protein subunit alpha-13 (G alpha-13) (G-protein subunit alpha-13)	44 kDa	0.14	55	31	25	8	6	10	7	3	3	10	9	-1.097366113	0% (0.41)
AP1M1_HUMAN (+3)	RS25_HUMAN	RPS25	P62851	40S ribosomal protein S25	14 kDa	0.11	36	20	16	3	4	6	7	4	5	3	4	-1.100311905	0% (0.44)

LBR_HUMAN	B4DN31_HUMAN		B4DN31	cDNA FLJ55809	51 kDa	0.057	36	20	16	5	6	5	4	4	2	3	7	-1.100311905	0% (0.44)
IMP3_HUMAN	H0YHS6_HUMAN	YARS2	H0YHS6	Tyrosine--tRNA ligase, mitochondrial (Fragment)	32 kDa	0.34	74	41	33	14	12	7	8	10	4	6	13	-1.101851574	0% (0.38)
A8K8A4_HUMAN (+2)	ODO1_HUMAN	OGDH	Q02218	2-oxoglutarate dehydrogenase, mitochondrial (EC 1.2.4.2) (2-oxoglutarate dehydrogenase complex component E1) (OGDC-E1) (Alpha-ketoglutarate dehydrogenase)	116 kDa	0.11	179	99	80	37	27	19	17	27	10	23	21	-1.102168598	0% (0.29)
PP1R7_HUMAN	UTRO_HUMAN	UTRN	P46939	Utrophin (Dystrophin-related protein 1) (DRP-1)	394 kDa	0.081	7	4	3	1	2	0	1	1	0	2	0	-1.103337259	0% (0.56)
A7YA96_HUMAN (+1)	PAR14_HUMAN	PARP14	Q460N5	Poly [ADP-ribose] polymerase 14 (PARP-14) (EC 2.4.2.30) (ADP-ribosyltransferase diphtheria toxin-like 8) (ARTD8) (B aggressive lymphoma protein 2)	203 kDa	0.067	7	4	3	0	0	0	4	3	0	0	0	-1.103337259	0% (0.56)
PRKRA_HUMAN	E7EQN5_HUMAN	ARHGAP4	E7EQN5	Rho GTPase-activating protein 4	103 kDa	0.073	7	4	3	0	0	0	4	2	0	0	1	-1.103337259	0% (0.56)
PDC10_HUMAN	C9JC84_HUMAN	FGG	C9JC84	Fibrinogen gamma chain	52 kDa	0.17	7	4	3	1	0	0	3	0	0	0	3	-1.103337259	0% (0.56)
CY24A_HUMAN	DTX3L_HUMAN	DTX3L	Q8TDB6	E3 ubiquitin-protein ligase DTX3L (EC 6.3.2.-) (B-lymphoma- and BAL-associated protein) (Protein deltex-3-like) (Rhysin-2) (Rhysin2)	84 kDa	0.11	7	4	3	0	1	1	2	2	1	0	0	-1.103337259	0% (0.56)
GRB7_HUMAN	A2A2G4_HUMAN	ALG6	A2A2G4	Dolichyl pyrophosphate Man9GlcNAc2 alpha-1,3-glucosyltransferase	58 kDa	0.083	7	4	3	2	2	0	0	0	1	2	0	-1.103337259	0% (0.56)
B3KQN7_HUMAN (+2)	OGT1_HUMAN	OGT	O15294	UDP-N-acetylglucosamine--peptide N-acetylglucosaminyltransferase 110 kDa subunit (EC 2.4.1.255) (O-GlcNAc transferase subunit p110) (O-linked N-acetylglucosamine transferase 110 kDa subunit) (OGT)	117 kDa	0.035	7	4	3	0	2	2	0	0	1	2	0	-1.103337259	0% (0.56)
B4E1K0_HUMAN (+2)	PDE12_HUMAN	PDE12	Q6L8Q7	2',5'-phosphodiesterase 12 (2'-PDE) (2-PDE) (EC 3.1.4.-) (Mitochondrial deadenylase) (EC 3.1.13.4)	67 kDa	0.036	7	4	3	2	0	1	1	1	2	0	0	-1.103337259	0% (0.56)
S10AE_HUMAN	PIGO_HUMAN	PIGO	Q8TEQ8	GPI ethanolamine phosphate transferase 3 (EC 2.-.-.-) (Phosphatidylinositol-glycan biosynthesis class O protein) (PIG-O)	119 kDa	0.25	7	4	3	1	2	0	1	2	1	1	0	-1.103337259	0% (0.56)
B4DG22_HUMAN (+4)	POF1B_HUMAN	POF1B	Q8WVV4	Protein POF1B (Premature ovarian failure protein 1B)	68 kDa	0.083	7	4	3	4	0	0	0	3	0	0	0	-1.103337259	0% (0.56)
B4DY16_HUMAN (+2)	B7ZKY2_HUMAN	CASK	B7ZKY2	Calcium/calmodulin-dependent serine protein kinase (MAGUK family)	102 kDa	0.13	7	4	3	0	2	2	0	1	0	1	1	-1.103337259	0% (0.56)
DAZP1_HUMAN	A8K5R6_HUMAN		A8K5R6	Golgi SNAP receptor complex member 1	29 kDa	0.12	7	4	3	3	0	0	1	0	2	0	1	-1.103337259	0% (0.56)
B7Z4K8_HUMAN (+2)	B3KQ71_HUMAN		B3KQ71	cDNA FLJ32982 fis, clone THYMU1000002, highly similar to Golgi phosphoprotein 3	32 kDa	0.089	7	4	3	0	1	2	1	0	0	2	1	-1.103337259	0% (0.56)
D7RF68_HUMAN	SPX2_HUMAN	SLC37A2	Q8TED4	Sugar phosphate exchanger 2 (Solute carrier family 37 member 2)	54 kDa	0.055	7	4	3	1	2	1	0	0	0	2	1	-1.103337259	0% (0.56)
E9PK47_HUMAN (+1)	D39U1_HUMAN	SDR39U1	Q9NRG7	Epimerase family protein SDR39U1 (EC 1.1.1.-) (Short-chain dehydrogenase/reductase family 39U member 1)	35 kDa	0.12	7	4	3	2	0	1	1	2	0	1	0	-1.103337259	0% (0.56)
CMPK2_HUMAN	B7Z322_HUMAN		B7Z322	cDNA FLJ52146, highly similar to Grancalcin	22 kDa	0.2	7	4	3	0	1	2	1	0	3	0	0	-1.103337259	0% (0.56)

B4DKU3_HUMAN (+1)	F8WAB8_HUMAN	SLC25A26	F8WAB8	S-adenosylmethionine mitochondrial carrier protein	29 kDa	0.076	7	4	3	2	0	0	2	1	1	0	1	- 1.1033372 59	0% (0.56)
RL35A_HUMAN	F134A_HUMAN	FAM134A	Q8NC44	Protein FAM134A	58 kDa	0.26	7	4	3	3	0	0	1	1	1	1	0	- 1.1033372 59	0% (0.56)
B3KYA7_HUMAN	LAR4B_HUMAN	LARP4B	Q92615	La-related protein 4B (La ribonucleoprotein domain family member 4B) (La ribonucleoprotein domain family member 5) (La-related protein 5)	81 kDa	0.073	6	4	3	0	1	2	1	1	1	1	0	- 1.1033372 59	0% (0.56)
KC1A_HUMAN	SRP09_HUMAN	SRP9	P49458	Signal recognition particle 9 kDa protein (SRP9)	10 kDa	0.1	7	4	3	0	0	0	4	0	1	2	0	- 1.1033372 59	0% (0.56)
MYO1E_HUMAN	ANO10_HUMAN	ANO10	Q9NW15	Anoctamin-10 (Transmembrane protein 16K)	76 kDa	0.09	7	4	3	2	1	1	0	0	2	1	0	- 1.1033372 59	0% (0.56)
B4DIY7_HUMAN	J3QK90_HUMAN	NSFL1C	J3QK90	NSFL1 cofactor p47	41 kDa	0.097	7	4	3	0	2	2	0	0	1	0	2	- 1.1033372 59	0% (0.56)
TTL12_HUMAN	B4DRA5_HUMAN		B4DRA5	cDNA FLJ61346, highly similar to Protein transport protein Sec23B	84 kDa	0.056	45	25	20	4	4	8	9	4	8	4	4	- 1.1033651 49	0% (0.42)
THMS2_HUMAN	ATP5L_HUMAN	ATP5L	O75964	ATP synthase subunit g, mitochondrial (ATPase subunit g)	11 kDa	0.096	45	25	20	5	8	5	7	4	5	5	6	- 1.1033651 49	0% (0.42)
Q59E99_HUMAN (+1)	F5GWP8_HUMAN	JUP	F5GWP8	Junction plakoglobin	66 kDa	0.042	159	88	71	41	11	20	0	18	0	17	22	- 1.1034489 09	0% (0.30)
AT2B1_HUMAN	B4DP27_HUMAN		B4DP27	cDNA FLJ52153, highly similar to Transmembrane emp24 domain-containing protein 2	19 kDa	0.093	54	30	24	10	6	4	10	6	6	5	7	- 1.1054534 01	0% (0.40)
A8K2X4_HUMAN (+3)	6PGD_HUMAN	PGD	P52209	6-phosphogluconate dehydrogenase, decarboxylating (EC 1.1.1.44)	53 kDa	0.09	54	30	24	2	9	10	9	3	9	4	8	- 1.1054534 01	0% (0.40)
SPB9_HUMAN	LRC59_HUMAN	LRRC59	Q96AG4	Leucine-rich repeat-containing protein 59 (Ribosome-binding protein p34) (p34)	35 kDa	0.22	157	87	70	24	24	20	20	16	16	20	18	- 1.1064005 14	0% (0.29)
TLR2_HUMAN	SYDC_HUMAN	DARS	P14868	Aspartate--tRNA ligase, cytoplasmic (EC 6.1.1.12) (Aspartyl-tRNA synthetase) (AspRS) (Cell proliferation-inducing gene 40 protein)	57 kDa	0.098	63	35	28	2	10	12	12	9	17	4	0	- 1.1069725 56	0% (0.39)
A6GV77_HUMAN (+1)	Q6FHK9_HUMAN	PSME2	Q6FHK9	PSME2 protein	27 kDa	0.088	63	35	28	2	11	9	13	5	11	9	3	- 1.1069725 56	0% (0.39)
CPSF3_HUMAN (+2)	ODPA_HUMAN	PDHA1	P08559	Pyruvate dehydrogenase E1 component subunit alpha, somatic form, mitochondrial (EC 1.2.4.1) (PDHE1-A type I)	43 kDa	0.079	73	40	32	8	10	11	11	14	6	7	7	- 1.1081280 02	0% (0.37)
SMCA1_HUMAN	A8K1D2_HUMAN		A8K1D2	cDNA FLJ76601, highly similar to Homo sapiens LIM and SH3 protein 1 (LASP1), mRNA	30 kDa	0.099	16	9	7	1	3	4	1	3	3	0	1	- 1.1097131 66	0% (0.50)
SC5A3_HUMAN	LRC47_HUMAN	LRRC47	Q8N1G4	Leucine-rich repeat-containing protein 47	63 kDa	0.081	16	9	7	0	1	2	6	2	5	0	0	- 1.1097131 66	0% (0.50)
Q86YH3_HUMAN	WDR61_HUMAN	WDR61	Q9GZS3	WD repeat-containing protein 61 (Meiotic recombination REC14 protein homolog) (SKI8 homolog) (Ski8)	34 kDa	0.075	16	9	7	0	3	3	3	2	5	0	0	- 1.1097131 66	0% (0.50)
TRIPC_HUMAN	SPTC2_HUMAN	SPTLC2	O15270	Serine palmitoyltransferase 2 (EC 2.3.1.50) (Long chain base biosynthesis protein 2) (LCB 2) (Long chain base biosynthesis protein 2a) (LCB2a) (Serine-palmitoyl-CoA transferase 2) (SPT 2)	63 kDa	0.019	16	9	7	4	3	0	2	2	0	4	1	- 1.1097131 66	0% (0.50)
CDCP1_HUMAN	IMP3_HUMAN	IMP3	Q9NV31	U3 small nucleolar ribonucleoprotein protein IMP3 (U3 snoRNP protein IMP3) (BRMS2)	22 kDa	0.1	16	9	7	1	1	2	5	0	2	2	3	- 1.1097131 66	0% (0.50)
C9J837_HUMAN (+2)	RS23_HUMAN	RPS23	P62266	40S ribosomal protein S23	16 kDa	0.066	16	9	7	2	2	2	3	2	2	1	2	- 1.1097131 66	0% (0.50)

J3QRN6_HUMAN (+1)	IQGA2_HUMAN	IQGAP2	Q13576	Ras GTPase-activating-like protein IQGAP2	181 kDa	0.085	15	9	7	1	3	5	0	0	2	4	0	-1.109713166	0% (0.50)
A4QN18_HUMAN (+3)	D3DNA1_HUMAN	ITGB5	D3DNA1	Integrin beta	77 kDa	0.017	16	9	7	2	4	2	1	0	0	4	0	-1.109713166	0% (0.50)
Q4LE60_HUMAN	B3KTM6_HUMAN	RPL5	B3KTM6	Ribosomal protein L5, isoform CRA_b (cDNA FLJ38496 fis, clone FELIV1000137, highly similar to 60S RIBOSOMAL PROTEIN L5)	28 kDa	0.074	126	70	56	14	19	14	23	9	19	17	11	-1.111692022	0% (0.31)
AN32E_HUMAN (+1)	A8K9T8_HUMAN		A8K9T8	cDNA FLJ76106, highly similar to Homo sapiens neurolysin (metallopeptidase M3 family) (NLN), mRNA	81 kDa	0.17	24	14	11	4	2	5	3	4	0	2	5	-1.111930103	0% (0.46)
CYBP_HUMAN (+1)	B3GQE6_HUMAN	DDX27	B3GQE6	DEAD box polypeptide 27	87 kDa	0.13	25	14	11	3	5	3	3	5	0	1	5	-1.111930103	0% (0.46)
B3KRR1_HUMAN (+2)	ABCF1_HUMAN	ABCF1	Q8NE71	ATP-binding cassette sub-family F member 1 (ATP-binding cassette 50) (TNF-alpha-stimulated ABC protein)	96 kDa	0.071	25	14	11	1	5	3	5	3	2	5	1	-1.111930103	0% (0.46)
Q3B7X1_HUMAN (+1)	Q9HBK7_HUMAN	RHBDL7	Q9HBK7	NPD007	47 kDa	0.11	25	14	11	4	5	3	3	1	3	3	4	-1.111930103	0% (0.46)
H0Y858_HUMAN	Q3KQZ2_HUMAN	SYNGR2	Q3KQZ2	Synaptogyrin	30 kDa	0.02	25	14	11	4	3	4	3	0	3	3	5	-1.111930103	0% (0.46)
RDH10_HUMAN	RTCB_HUMAN	RTCB	Q9Y3I0	tRNA-splicing ligase RtcB homolog (EC 6.5.1.3)	55 kDa	0.31	34	19	15	4	5	4	6	4	7	3	2	-1.11305931	0% (0.43)
FADS2_HUMAN	PARP9_HUMAN	PARP9	Q8IXQ6	Poly [ADP-ribose] polymerase 9 (PARP-9) (EC 2.4.2.30) (ADP-ribosyltransferase diphtheria toxin-like 9) (ARTD9) (B aggressive lymphoma protein)	96 kDa	0.14	34	19	15	4	5	4	6	4	5	3	3	-1.11305931	0% (0.43)
B4DL39_HUMAN (+2)	COR1B_HUMAN	CORO1B	Q9BR76	Coronin-1B (Coronin-2)	54 kDa	0.063	34	19	15	3	5	7	4	1	9	2	3	-1.11305931	0% (0.43)
CIB1_HUMAN	HEXB_HUMAN	HEXB	P07686	Beta-hexosaminidase subunit beta (EC 3.2.1.52) (Beta-N-acetylhexosaminidase subunit beta) (Hexosaminidase subunit B) (Cervical cancer proto-oncogene 7 protein) (HCC-7) (N-acetyl-beta-glucosaminidase subunit beta) [Cleaved into: Beta-hexosaminidase subunit beta chain B; Beta-hexosaminidase subunit beta chain A]	63 kDa	0.37	42	24	19	5	8	7	4	1	2	6	11	-1.113745368	0% (0.41)
MTOR_HUMAN	Q6NZ55_HUMAN	RPL13	Q6NZ55	60S ribosomal protein L13	24 kDa	0.01	43	24	19	7	4	7	6	5	6	4	4	-1.113745368	0% (0.41)
B4DH53_HUMAN	MDHC_HUMAN	MDH1	P40925	Malate dehydrogenase, cytoplasmic (EC 1.1.1.37) (Cytosolic malate dehydrogenase) (Diiodophenylpyruvate reductase) (EC 1.1.1.96)	36 kDa	0.06	43	24	19	1	6	6	11	8	2	5	4	-1.113745368	0% (0.41)
B4DF41_HUMAN (+2)	PABP2_HUMAN	PABPN1	Q86U42	Polyadenylate-binding protein 2 (PABP-2) (Poly(A)-binding protein 2) (Nuclear poly(A)-binding protein 1) (Poly(A)-binding protein II) (PABII) (Polyadenylate-binding nuclear protein 1)	33 kDa	0.085	43	24	19	5	8	6	5	6	3	6	4	-1.113745368	0% (0.41)
A8K7T0_HUMAN (+1)	H3BV16_HUMAN	NDUFB10	H3BV16	NADH dehydrogenase [ubiquinone] 1 beta subcomplex subunit 10 (Fragment)	17 kDa	0.14	42	24	19	8	4	4	8	5	3	5	7	-1.113745368	0% (0.41)
PYRD_HUMAN	B3KSH1_HUMAN	EIF3F	B3KSH1	Eukaryotic translation initiation factor 3 subunit F (eIF3f) (Eukaryotic translation initiation factor 3 subunit 5) (eIF-3-epsilon) (eIF3 p47)	39 kDa	0.15	42	24	19	1	8	8	7	7	5	5	2	-1.113745368	0% (0.41)

D6REA1_HUMAN	PP1B_HUMAN	PPP1CB	P62140	Serine/threonine-protein phosphatase PP1-beta catalytic subunit (PP-1B) (PPP1CD) (EC 3.1.3.16) (EC 3.1.3.53)	37 kDa	0.083	98	54	43	9	14	11	20	11	9	13	11	-1.115285464	0% (0.33)
NDUA5_HUMAN	CTNA1_HUMAN	CTNNA1	P35221	Catenin alpha-1 (Alpha E-catenin) (Cadherin-associated protein) (Renal carcinoma antigen NY-REN-13)	100 kDa	0.35	160	88	70	27	18	26	17	26	4	20	21	-1.118952023	0% (0.27)
UK114_HUMAN	A8K4Z4_HUMAN		A8K4Z4	cDNA FLJ75549, highly similar to Homo sapiens ribosomal protein, large, P0 (RPLP0), transcript variant 1, mRNA	34 kDa	0.31	139	78	62	16	19	18	25	19	16	12	14	-1.119234318	0% (0.28)
HEBP1_HUMAN	A8K916_HUMAN		A8K916	cDNA FLJ78481, highly similar to Homo sapiens adaptor-related protein complex 2, beta 1 subunit, mRNA	106 kDa	0.25	96	53	42	16	15	12	10	14	5	16	8	-1.120421238	0% (0.32)
SNX5_HUMAN	UFL1_HUMAN	UFL1	O94874	E3 UFM1-protein ligase 1 (EC 6.3.2.-) (LZAP-binding protein)	90 kDa	0.072	87	48	38	13	11	10	14	9	7	10	11	-1.12080836	0% (0.33)
B3KSE0_HUMAN (+3)	PRDX1_HUMAN	PRDX1	Q06830	Peroxiredoxin-1 (EC 1.11.1.15) (Natural killer cell-enhancing factor A) (NKEF-A) (Proliferation-associated gene protein) (PAG) (Thioredoxin peroxidase 2) (Thioredoxin-dependent peroxide reductase 2)	22 kDa	0.12	182	102	81	14	30	37	22	22	28	17	14	-1.121377098	0% (0.25)
B2R6P4_HUMAN (+1)	ABHDA_HUMAN	ABHD10	Q9NUJ1	Mycophenolic acid acyl-glucuronide esterase, mitochondrial (EC 3.1.1.93) (Alpha/beta hydrolase domain-containing protein 10) (Abhydrolase domain-containing protein 10)	34 kDa	0.14	68	38	30	9	11	8	10	12	5	5	8	-1.121885669	0% (0.36)
NDUA4_HUMAN	AT131_HUMAN	ATP13A1	Q9HD20	Probable cation-transporting ATPase 13A1 (EC 3.6.3.-)	133 kDa	0.27	68	38	30	19	4	4	11	4	8	10	8	-1.121885669	0% (0.36)
QPCTL_HUMAN	PGH1_HUMAN	PTGS1	P23219	Prostaglandin G/H synthase 1 (EC 1.14.99.1) (Cyclooxygenase-1) (COX-1) (Prostaglandin H2 synthase 1) (PGH synthase 1) (PGHS-1) (PHS 1) (Prostaglandin-endoperoxide synthase 1)	69 kDa	0.071	68	38	30	8	2	3	25	6	5	2	17	-1.121885669	0% (0.36)
GNL1_HUMAN (+1)	DJB11_HUMAN	DNAJB11	Q9UBS4	DnaJ homolog subfamily B member 11 (APOBEC1-binding protein 2) (ABBP-2) (DnaJ protein homolog 9) (ER-associated DNAJ) (ER-associated Hsp40 co-chaperone) (Endoplasmic reticulum DNA J domain-containing protein 3) (ER-resident protein ERdj3) (ERdj3) (ERj3p) (HEDJ) (Human DnaJ protein 9) (hDj-9) (PWP1-interacting protein 4)	41 kDa	0.066	93	52	41	13	12	11	16	8	11	12	11	-1.125800062	0% (0.32)
A8K885_HUMAN (+1)	OST48_HUMAN	DDOST	P39656	Dolichyl-diphosphooligosaccharide--protein glycosyltransferase 48 kDa subunit (DDOST 48 kDa subunit) (Oligosaccharyl transferase 48 kDa subunit) (EC 2.4.99.18)	51 kDa	0.14	188	105	83	37	22	14	33	15	27	17	24	-1.126576817	0% (0.23)
ABHEB_HUMAN (+2)	B3KQB4_HUMAN		B3KQB4	cDNA FLJ90131 fis, clone HEMBB1000447, highly similar to PRA1 family protein 3	22 kDa	0.18	35	18	14	6	4	3	4	3	4	3	3	-1.127478325	0% (0.42)
SPHM_HUMAN	FAH2A_HUMAN	FAHD2A	Q96GK7	Fumarylacetoacetate hydrolase domain-containing protein 2A (EC 3.-.-.-)	35 kDa	0.068	32	18	14	4	4	10	0	5	5	3	1	-1.127478325	0% (0.42)
FA98A_HUMAN (+1)	PRS4_HUMAN	PSMC1	P62191	26S protease regulatory subunit 4 (P26s4) (26S proteasome AAA-ATPase subunit RPT2) (Proteasome 26S subunit ATPase 1)	49 kDa	0.096	32	18	14	0	5	6	7	1	9	4	0	-1.127478325	0% (0.42)

MYO1G_HUMAN	TNAP2_HUMAN	TNFAIP2	Q03169	Tumor necrosis factor alpha-induced protein 2 (TNF alpha-induced protein 2) (Primary response gene B94 protein)	73 kDa	0.06	32	18	14	1	5	8	4	1	8	4	1	-1.127478325	0% (0.42)
Q5HYL6_HUMAN	RAB12_HUMAN	RAB12	Q6IQ22	Ras-related protein Rab-12	27 kDa	0.2	32	18	14	4	5	4	0	0	0	4	4	-1.127478325	0% (0.42)
SIAS_HUMAN	B2R7M1_HUMAN		B2R7M1	cDNA, FLJ93507, highly similar to Homo sapiens ATPase, H+ transporting, lysosomal 38kDa, V0 subunit d isoform 1 (ATP6V0D1), mRNA	40 kDa	0.14	66	37	29	14	9	8	7	1	10	8	10	-1.129446565	0% (0.35)
PDIA5_HUMAN	RASN_HUMAN	NRAS	P01111	GTPase NRas (Transforming protein N-Ras)	21 kDa	0.062	23	13	10	1	6	3	3	5	1	4	1	-1.131375428	0% (0.45)
B2RTQ5_HUMAN (+2)	FCGRN_HUMAN	FCGRT	P55899	IgG receptor FcRn large subunit p51 (FcRn) (IgG Fc fragment receptor transporter alpha chain) (Neonatal Fc receptor)	40 kDa	0.023	22	13	10	2	3	2	6	0	4	1	5	-1.131375428	0% (0.45)
TOR4A_HUMAN	B4DLB8_HUMAN		B4DLB8	cDNA FLJ52205, highly similar to Beta-1,4-galactosyltransferase 1 (EC 2.4.1.-)	40 kDa	0.12	23	13	10	4	4	3	2	1	2	3	4	-1.131375428	0% (0.45)
CRKL_HUMAN	GNA11_HUMAN	GNA11	P29992	Guanine nucleotide-binding protein subunit alpha-11 (G alpha-11) (G-protein subunit alpha-11) (Guanine nucleotide-binding protein G(y) subunit alpha)	42 kDa	0.14	23	13	10	8	1	2	2	3	1	3	3	-1.131375428	0% (0.45)
A8K3W7_HUMAN (+1)	FUBP3_HUMAN	FUBP3	Q96I24	Far upstream element-binding protein 3 (FUSE-binding protein 3)	62 kDa	0.16	23	13	10	2	0	4	0	0	0	6	0	-1.131375428	0% (0.45)
A8K2Q7_HUMAN (+1)	B9EJA8_HUMAN	MRC1L1	B9EJA8	Mannose receptor, C type 1-like 1	166 kDa	0.13	91	51	40	5	22	24	0	0	3	16	21	-1.131439609	0% (0.31)
Q53TD0_HUMAN (+1)	B2R6D7_HUMAN		B2R6D7	cDNA, FLJ92904, highly similar to Homo sapiens casein kinase 2, alpha 1 polypeptide (CSNK2A1), mRNA	45 kDa	0.043	81	46	36	5	13	11	16	10	8	6	12	-1.13303065	0% (0.32)
ACOT8_HUMAN (+1)	Q59GY2_HUMAN		Q59GY2	Ribosomal protein L4 variant (Fragment)	49 kDa	0.078	165	93	73	26	24	22	22	18	17	26	13	-1.133919388	0% (0.24)
B3KQ18_HUMAN	FXR1_HUMAN	FXR1	P51114	Fragile X mental retardation syndrome-related protein 1 (hFXR1p)	70 kDa	0.049	64	36	28	6	9	11	10	9	8	6	5	-1.137524351	0% (0.34)
FMNL_HUMAN	CS010_HUMAN	C19orf10	Q969H8	UPF0556 protein C19orf10 (Interleukin-25) (IL-25) (Stromal cell-derived growth factor SF20)	19 kDa	0.049	39	22	17	6	5	5	6	2	3	3	9	-1.137918031	0% (0.39)
Q5VWC4_HUMAN	B5MBZ0_HUMAN	EML4	B5MBZ0	Echinoderm microtubule-associated protein-like 4	110 kDa	0.092	15	8	6	1	2	3	2	3	1	2	1	-1.139581041	0% (0.48)
A8K935_HUMAN (+3)	RT25_HUMAN	MRPS25	P82663	28S ribosomal protein S25, mitochondrial (MRP-S25) (S25mt)	20 kDa	0.027	14	8	6	3	2	1	2	2	1	1	2	-1.139581041	0% (0.48)
A8K139_HUMAN (+3)	SC5A3_HUMAN	SLC5A3	P53794	Sodium/myo-inositol cotransporter (Na(+)/myo-inositol cotransporter) (Sodium/myo-inositol transporter 1) (SMIT1) (Solute carrier family 5 member 3)	80 kDa	0.078	14	8	6	5	3	0	0	6	0	0	0	-1.139581041	0% (0.48)
TF3C3_HUMAN	G3V1C3_HUMAN	API5	G3V1C3	Apoptosis inhibitor 5	58 kDa	0.068	14	8	6	0	2	1	5	0	0	6	0	-1.139581041	0% (0.48)
B9A018_HUMAN (+1)	LYSC_HUMAN	LYZ	P61626	Lysozyme C (EC 3.2.1.17) (1,4-beta-N-acetylmuramidase C)	17 kDa	0.14	14	8	6	2	2	2	2	0	2	2	2	-1.139581041	0% (0.48)
A8K5B0_HUMAN (+1)	ENPP4_HUMAN	ENPP4	Q9Y6X5	Bis(5'-adenosyl)-triphosphatase ENPP4 (EC 3.6.1.29) (AP3A hydrolase) (AP3Aase) (Ectonucleotide pyrophosphatase/phosphodiesterase family member 4) (E-NPP 4) (NPP-4)	52 kDa	0.25	14	8	6	4	2	1	1	1	0	3	2	-1.139581041	0% (0.48)
ATAD2_HUMAN	KRR1_HUMAN	KRR1	Q13601	KRR1 small subunit processome component homolog (HIV-1 Rev-binding	44 kDa	0.07	14	8	6	0	2	2	4	1	2	1	2	-1.139581041	0% (0.48)

				protein 2) (KRR-R motif-containing protein 1) (Rev-interacting protein 1) (Rip-1)														
NOL11_HUMAN	B4DH46_HUMAN		B4DH46	cDNA FLJ60519, highly similar to WD repeat protein 68	19 kDa	0.056	14	8	6	0	1	1	6	2	2	1	1	-1.139581041	0% (0.48)
RM21_HUMAN	TM41A_HUMAN	TMEM41A	Q96HV5	Transmembrane protein 41A	30 kDa	0.14	14	8	6	3	1	1	2	1	1	2	2	-1.139581041	0% (0.48)
Q6FHJ5_HUMAN (+1)	B7Z418_HUMAN	ILK	B7Z418	Integrin-linked protein kinase (cDNA FLJ50979, moderately similar to Integrin-linked protein kinase (EC 2.7.11.1))	44 kDa	0.092	14	8	6	0	2	4	2	0	2	2	2	-1.139581041	0% (0.48)
F5GZD0_HUMAN	A5PLM9_HUMAN	CTSL1	A5PLM9	Cathepsin L1	38 kDa	0.056	14	8	6	0	3	3	2	0	3	2	1	-1.139581041	0% (0.48)
NCOA5_HUMAN	ANX11_HUMAN	ANXA11	P50995	Annexin A11 (56 kDa autoantigen) (Annexin XI) (Annexin-11) (Calcyclin-associated annexin 50) (CAP-50)	54 kDa	0.079	189	105	82	31	24	24	28	15	20	19	31	-1.140125691	0% (0.21)
LAP2_HUMAN (+1)	RS3_HUMAN	RPS3	P23396	40S ribosomal protein S3	27 kDa	0.023	128	72	56	17	20	15	21	19	17	12	8	-1.142926806	0% (0.25)
G3V1C3_HUMAN	NDUS1_HUMAN	NDUFS1	P28331	NADH-ubiquinone oxidoreductase 75 kDa subunit, mitochondrial (EC 1.6.5.3) (EC 1.6.99.3) (Complex I-75kD) (CI-75kD)	79 kDa	0.17	127	72	56	28	20	14	10	18	5	15	17	-1.142926806	0% (0.25)
B2R6P1_HUMAN (+4)	S10A4_HUMAN	S100A4	P26447	Protein S100-A4 (Calvasculin) (Metastasin) (Placental calcium-binding protein) (Protein Mts1) (S100 calcium-binding protein A4)	12 kDa	0.067	30	17	13	6	2	4	5	3	4	2	4	-1.14392087	0% (0.41)
F8VSD4_HUMAN (+3)	B2R5V2_HUMAN		B2R5V2	Succinyl-CoA:3-ketoacid-coenzyme A transferase (EC 2.8.3.5)	56 kDa	0.21	30	17	13	3	9	6	0	10	0	3	0	-1.14392087	0% (0.41)
G3XAB3_HUMAN (+1)	B4E2S7_HUMAN	LAMP2	B4E2S7	Lysosome-associated membrane glycoprotein 2 (cDNA FLJ58780, highly similar to Homo sapiens lysosomal-associated membrane protein 2 (LAMP2), transcript variant LAMP2B, mRNA)	40 kDa	0.031	30	17	13	5	6	3	3	1	2	5	5	-1.14392087	0% (0.41)
B8QFA1_HUMAN (+2)	Q96DV6_HUMAN	RPS6	Q96DV6	40S ribosomal protein S6	29 kDa	0.053	46	26	20	7	7	7	5	7	5	6	2	-1.145412828	0% (0.37)
B7Z700_HUMAN (+1)	B2RAQ8_HUMAN		B2RAQ8	cDNA, FLJ95058, highly similar to Homo sapiens carnitine palmitoyltransferase 1A (liver) (CPT1A),nuclear gene encoding mitochondrial protein, mRNA	88 kDa	0.063	133	75	58	22	16	17	20	15	9	20	14	-1.149587478	0% (0.24)
PLMN_HUMAN	B3KNN7_HUMAN		B3KNN7	cDNA FLJ30049 fis, clone ADRGL1000033, highly similar to 26S proteasome non-ATPase regulatory subunit 3	57 kDa	0.075	85	48	37	2	13	15	18	7	21	5	5	-1.150127092	0% (0.29)
A8K984_HUMAN (+3)	A8K4H1_HUMAN		A8K4H1	cDNA FLJ78268, highly similar to Homo sapiens fusion (involved in t(12;16) in malignant liposarcoma), transcript variant 1, mRNA	53 kDa	0.036	70	39	30	11	14	10	4	8	3	7	13	-1.150483532	0% (0.32)
ES8L1_HUMAN	RALA_HUMAN	RALA	P11233	Ras-related protein Ral-A	24 kDa	0.14	69	39	30	13	13	6	7	6	9	6	9	-1.150483532	0% (0.32)
TIM14_HUMAN	B2RD27_HUMAN		B2RD27	cDNA, FLJ96428, highly similar to Homo sapiens proteasome (prosome, macropain) 26S subunit, non-ATPase, 7 (Mov34 homolog) (PSMD7), mRNA	37 kDa	0.36	53	30	23	3	9	6	12	3	13	5	2	-1.151055671	0% (0.35)
ATAD1_HUMAN	BZW1_HUMAN	BZW1	Q7L1Q6	Basic leucine zipper and W2 domain-containing protein 1 (Protein Orf)	48 kDa	0.1	37	21	16	1	8	9	3	3	8	5	1	-1.152106095	0% (0.38)
CKLF6_HUMAN	PRS6B_HUMAN	PSMC4	P43686	26S protease regulatory subunit 6B (26S proteasome AAA-ATPase subunit RPT3)	47 kDa	0.06	37	21	16	3	5	7	6	5	5	4	2	-1.152106095	0% (0.38)

				(MB67-interacting protein) (MIP224) (Proteasome 26S subunit ATPase 4) (Tat-binding protein 7) (TBP-7)															
RS23_HUMAN	A8KAQ5_HUMAN		A8KAQ5	cDNA FLJ77404, highly similar to Homo sapiens small nuclear ribonucleoprotein 70kDa polypeptide (RNP antigen) (SNRP70), transcript variant 1, mRNA	51 kDa	0.16	22	12	9	1	5	4	2	3	2	3	1	-1.154614695	0% (0.44)
NFU1_HUMAN	CNOT1_HUMAN	CNOT1	A5YKK6	CCR4-NOT transcription complex subunit 1 (CCR4-associated factor 1) (Negative regulator of transcription subunit 1 homolog) (NOT1H) (hNOT1)	267 kDa	0.15	21	12	9	1	5	6	0	3	2	3	1	-1.154614695	0% (0.44)
A2A3U5_HUMAN (+1)	PDXK_HUMAN	PDXK	O00764	Pyridoxal kinase (EC 2.7.1.35) (Pyridoxine kinase)	35 kDa	0.13	21	12	9	1	5	3	3	2	1	5	1	-1.154614695	0% (0.44)
H7BXZ6_HUMAN (+1)	COPZ1_HUMAN	COPZ1	P61923	Coatomer subunit zeta-1 (Zeta-1-coat protein) (Zeta-1 COP)	20 kDa	0.07	21	12	9	0	4	6	2	0	6	1	2	-1.154614695	0% (0.44)
SNAG_HUMAN	PM14_HUMAN	SF3B14	Q9Y3B4	Pre-mRNA branch site protein p14 (SF3b 14 kDa subunit)	15 kDa	0.13	21	12	9	2	2	4	4	0	1	3	5	-1.154614695	0% (0.44)
ACAD8_HUMAN	PEF1_HUMAN	PEF1	Q9UBV8	Peflin (PEF protein with a long N-terminal hydrophobic domain) (Penta-EF hand domain-containing protein 1)	30 kDa	0.13	21	12	9	4	3	2	3	2	3	2	2	-1.154614695	0% (0.44)
A8K588_HUMAN (+1)	USMG5_HUMAN	USMG5	Q96IX5	Up-regulated during skeletal muscle growth protein 5 (Diabetes-associated protein in insulin-sensitive tissues) (HCV F-transactivated protein 2)	6 kDa	0.12	21	12	9	3	2	2	5	2	2	2	3	-1.154614695	0% (0.44)
VATG1_HUMAN	TMM43_HUMAN	TMEM43	Q9BTV4	Transmembrane protein 43 (Protein LUMA)	45 kDa	0.31	60	34	26	13	5	5	11	2	12	1	12	-1.155458216	0% (0.33)
DBLOH_HUMAN (+1)	RAB5B_HUMAN	RAB5B	P61020	Ras-related protein Rab-5B	24 kDa	0.14	61	34	26	9	9	9	8	5	8	5	8	-1.155458216	0% (0.33)
CSK2B_HUMAN (+2)	AK1A1_HUMAN	AKR1A1	P14550	Alcohol dehydrogenase [NADP(+)] (EC 1.1.1.2) (Aldehyde reductase) (Aldo-keto reductase family 1 member A1)	37 kDa	0.14	46	25	19	4	4	4	13	2	6	3	7	-1.157868977	0% (0.36)
B0QYK0_HUMAN (+3)	RBBP4_HUMAN	RBBP4	Q09028	Histone-binding protein RBBP4 (Chromatin assembly factor 1 subunit C) (CAF-1 subunit C) (Chromatin assembly factor I p48 subunit) (CAF-I 48 kDa subunit) (CAF-I p48) (Nucleosome-remodeling factor subunit RBAP48) (Retinoblastoma-binding protein 4) (RBBP-4) (Retinoblastoma-binding protein p48)	48 kDa	0.047	44	25	19	7	7	7	4	4	1	9	5	-1.157868977	0% (0.36)
CDC73_HUMAN	IDHP_HUMAN	IDH2	P48735	Isocitrate dehydrogenase [NADP], mitochondrial (IDH) (EC 1.1.1.42) (ICD-M) (IDP) (NADP(+)-specific ICDH) (Oxalosuccinate decarboxylase)	51 kDa	0.034	536	304	234	73	66	63	104	49	60	64	61	-1.159566458	0% (0.052)
A8KA56_HUMAN (+2)	ALDH2_HUMAN	ALDH2	P05091	Aldehyde dehydrogenase, mitochondrial (EC 1.2.1.3) (ALDH class 2) (ALDH-E2) (ALDHI)	56 kDa	0.083	219	124	95	27	34	39	24	6	32	26	32	-1.162545306	0% (0.15)
B4DHN5_HUMAN (+3)	F120A_HUMAN	FAM120A	Q9NZB2	Constitutive coactivator of PPAR-gamma-like protein 1 (Oxidative stress-associated Src activator) (Protein FAM120A)	122 kDa	0.14	28	16	12	4	6	5	1	2	3	4	3	-1.162845101	0% (0.40)
COASY_HUMAN	MCAT_HUMAN	SLC25A20	O43772	Mitochondrial carnitine/acylcarnitine carrier protein (Carnitine/acylcarnitine translocase) (CAC) (Solute carrier family 25 member 20)	33 kDa	0.071	28	16	12	5	0	1	10	1	6	2	3	-1.162845101	0% (0.40)

B4DJK9_HUMAN (+1)	SYJ2B_HUMAN	SYNJ2BP	P57105	Synaptojanin-2-binding protein (Mitochondrial outer membrane protein 25)	16 kDa	0.18	28	16	12	4	4	4	4	3	4	4	1	-1.162845101	0% (0.40)
B1AHD1_HUMAN (+1)	TLN1_HUMAN	TLN1	Q9Y490	Talin-1	270 kDa	0.27	150	85	65	4	33	28	18	12	13	19	21	-1.162989621	0% (0.20)
GDPD3_HUMAN	B4E380_HUMAN		B4E380	Histone H3	13 kDa	0.22	79	46	35	12	10	8	16	11	10	5	8	-1.164303425	0% (0.28)
RFC1_HUMAN	J3KRX5_HUMAN	RPL17	J3KRX5	60S ribosomal protein L17 (Fragment)	20 kDa	0.02	81	46	35	12	12	9	14	10	6	8	11	-1.164303425	0% (0.28)
J3KMX1_HUMAN (+1)	HSDL2_HUMAN	HSDL2	Q6YN16	Hydroxysteroid dehydrogenase-like protein 2 (EC 1.-.-.-)	45 kDa	0.039	57	33	25	8	12	5	8	11	5	2	6	-1.165449784	0% (0.32)
Q53EL1_HUMAN (+1)	K2013_HUMAN	KIAA2013	Q8IYS2	Uncharacterized protein KIAA2013	69 kDa	0.065	5	3	2	0	0	1	2	1	0	0	1	-1.168003509	0% (0.55)
B4DQJ1_HUMAN (+1)	VPS18_HUMAN	VPS18	Q9P253	Vacuolar protein sorting-associated protein 18 homolog (hVPS18)	110 kDa	0.078	5	3	2	1	0	1	1	0	2	0	0	-1.168003509	0% (0.55)
NPL4_HUMAN	B4DWG1_HUMAN		B4DWG1	cDNA FLJ55643, highly similar to SEC23-interacting protein	90 kDa	0.048	5	3	2	2	1	0	0	0	2	0	0	-1.168003509	0% (0.55)
COX5B_HUMAN	PHF2_HUMAN	PHF2	O75151	Lysine-specific demethylase PHF2 (EC 1.14.11.-) (GRC5) (PHD finger protein 2)	121 kDa	0.19	5	3	2	0	1	0	2	0	1	1	0	-1.168003509	0% (0.55)
A8K005_HUMAN (+2)	GLNA_HUMAN	GLUL	P15104	Glutamine synthetase (GS) (EC 6.3.1.2) (Glutamate decarboxylase) (EC 4.1.1.15) (Glutamate--ammonia ligase)	42 kDa	0.2	5	3	2	0	1	0	2	0	3	0	0	-1.168003509	0% (0.55)
TCRG1_HUMAN	B3KUB9_HUMAN	SWAP70	B3KUB9	SWAP-70 protein, isoform CRA_b (cDNA FLJ39540 fis, clone PUAEN2008314, highly similar to Switch-associated protein 70)	62 kDa	0.018	4	3	2	1	0	1	0	1	1	0	0	-1.168003509	0% (0.55)
DDX58_HUMAN	Q0P5W4_HUMAN	RCE1	Q0P5W4	RCE1 protein (Fragment)	35 kDa	0.091	5	3	2	0	1	0	2	0	2	0	0	-1.168003509	0% (0.55)
TR112_HUMAN	CUL3_HUMAN	CUL3	Q13618	Cullin-3 (CUL-3)	89 kDa	0.22	5	3	2	0	0	3	0	1	0	1	0	-1.168003509	0% (0.55)
EPHA2_HUMAN	UB2L6_HUMAN	UBE2L6	O14933	Ubiquitin/ISG15-conjugating enzyme E2 L6 (EC 6.3.2.19) (Retinoic acid-induced gene B protein) (RIG-B) (UbcH8) (Ubiquitin carrier protein L6) (Ubiquitin-protein ligase L6)	18 kDa	0.06	5	3	2	0	0	0	3	0	2	0	0	-1.168003509	0% (0.55)
HBD_HUMAN	B4E381_HUMAN		B4E381	cDNA FLJ58568	46 kDa	0.71	5	3	2	2	1	0	0	0	0	1	1	-1.168003509	0% (0.55)
B2R6P3_HUMAN (+2)	B2RNR6_HUMAN	ZFR	B2RNR6	Zinc finger RNA binding protein	117 kDa	0.062	5	3	2	0	1	2	0	0	0	2	0	-1.168003509	0% (0.55)
AT2A3_HUMAN	PTGR1_HUMAN	PTGR1	Q14914	Prostaglandin reductase 1 (PRG-1) (EC 1.3.1.-) (15-oxoprostaglandin 13-reductase) (EC 1.3.1.48) (NADP-dependent leukotriene B4 12-hydroxydehydrogenase) (EC 1.3.1.74)	36 kDa	0.14	5	3	2	0	0	1	2	1	1	0	0	-1.168003509	0% (0.55)
B4DSK8_HUMAN (+2)	GALT7_HUMAN	GALNT7	Q86SF2	N-acetylgalactosaminyltransferase 7 (EC 2.4.1.-) (Polypeptide GalNAc transferase 7) (GalNAc-T7) (pp-GaNTase 7) (Protein-UDP acetylgalactosaminyltransferase 7) (UDP-GalNAc:polypeptide N-acetylgalactosaminyltransferase 7)	75 kDa	0.098	5	3	2	2	0	0	1	0	2	0	0	-1.168003509	0% (0.55)
ITPA_HUMAN	G5E994_HUMAN	GPR107	G5E994	G protein-coupled receptor 107, isoform CRA_c (Protein GPR107)	64 kDa	0.17	5	3	2	1	0	0	2	0	1	1	0	-1.168003509	0% (0.55)
IDE_HUMAN	CLCA_HUMAN	CLTA	P09496	Clathrin light chain A (Lca)	27 kDa	0.047	5	3	2	0	1	2	0	1	0	1	0	-1.168003509	0% (0.55)

A8K3C5_HUMAN (+2)	GAS6_HUMAN	GAS6	Q14393	Growth arrest-specific protein 6 (GAS-6) (AXL receptor tyrosine kinase ligand)	80 kDa	0.058	5	3	2	3	0	0	0	0	0	0	2	-1.168003509	0% (0.55)
A8K3Q3_HUMAN (+1)	PLP2_HUMAN	PLP2	Q04941	Proteolipid protein 2 (Differentiation-dependent protein A4) (Intestinal membrane A4 protein)	17 kDa	0.11	6	3	2	0	2	1	0	1	2	1	0	-1.168003509	0% (0.55)
B4DT03_HUMAN (+3)	F8WBK5_HUMAN	MRPL40	F8WBK5	39S ribosomal protein L40, mitochondrial	20 kDa	0.11	5	3	2	0	0	1	2	1	0	0	1	-1.168003509	0% (0.55)
GPAA1_HUMAN	A8K6B4_HUMAN		A8K6B4	cDNA FLJ77723	24 kDa	0.063	5	3	2	2	0	0	1	0	0	2	0	-1.168003509	0% (0.55)
E5RJR5_HUMAN (+3)	GRAM4_HUMAN	GRAMD4	Q6IC98	GRAM domain-containing protein 4 (Death-inducing protein)	66 kDa	0.086	5	3	2	3	0	0	0	0	0	0	2	-1.168003509	0% (0.55)
K7EIN2_HUMAN	A6NEM5_HUMAN	PIGK	A6NEM5	GPI-anchor transamidase	38 kDa	0.21	5	3	2	1	0	0	2	1	0	0	1	-1.168003509	0% (0.55)
FND3B_HUMAN	FKBP2_HUMAN	FKBP2	P26885	Peptidyl-prolyl cis-trans isomerase FKBP2 (PPIase FKBP2) (EC 5.2.1.8) (13 kDa FK506-binding protein) (13 kDa FKBP) (FKBP-13) (FK506-binding protein 2) (FKBP-2) (Immunophilin FKBP13) (Rotamase)	16 kDa	0.045	35	20	15	4	5	7	4	3	5	3	4	-1.168040212	0% (0.37)
B4DWH7_HUMAN (+2)	ARF4_HUMAN	ARF4	P18085	ADP-ribosylation factor 4	21 kDa	0.28	65	37	28	8	7	9	13	8	11	5	4	-1.168076932	0% (0.30)
J3KMZ9_HUMAN	B2R6K4_HUMAN		B2R6K4	cDNA, FLJ92996, highly similar to Homo sapiens guanine nucleotide binding protein (G protein), beta polypeptide 1 (GNB1), mRNA	37 kDa	0.025	65	37	28	13	8	9	7	9	4	6	9	-1.168076932	0% (0.30)
E7EX90_HUMAN	A6NN80_HUMAN	ANXA6	A6NN80	Annexin	75 kDa	0.031	183	105	80	21	35	32	17	6	25	23	24	-1.168223971	0% (0.17)
NU214_HUMAN	PCKGM_HUMAN	PCK2	Q16822	Phosphoenolpyruvate carboxykinase [GTP], mitochondrial (PEPCK-M) (EC 4.1.1.32) (Phosphoenolpyruvate carboxylase)	71 kDa	0.033	161	92	70	15	32	33	12	18	12	14	27	-1.169161285	0% (0.18)
RL8_HUMAN	CAN1_HUMAN	CAPN1	P07384	Calpain-1 catalytic subunit (EC 3.4.22.52) (Calcium-activated neutral proteinase 1) (CANP 1) (Calpain mu-type) (Calpain-1 large subunit) (Cell proliferation-inducing gene 30 protein) (Micromolar-calpain) (muCANP)	82 kDa	0.1	101	58	44	18	10	12	18	11	7	16	10	-1.169641825	0% (0.24)
5NT3A_HUMAN	GBLP_HUMAN	GNB2L1	P63244	Guanine nucleotide-binding protein subunit beta-2-like 1 (Cell proliferation-inducing gene 21 protein) (Guanine nucleotide-binding protein subunit beta-like protein 12.3) (Human lung cancer oncogene 7 protein) (HLC-7) (Receptor for activated C kinase) (Receptor of activated protein kinase C 1) (RACK1) [Cleaved into: Guanine nucleotide-binding protein subunit beta-2-like 1, N-terminally processed]	35 kDa	0.21	101	58	44	7	19	14	19	15	11	10	8	-1.169641825	0% (0.24)
B4DHQ3_HUMAN (+1)	Q5NKV8_HUMAN	ICAM1	Q5NKV8	Intercellular adhesion molecule 1	58 kDa	0.13	72	41	31	7	14	15	5	6	6	7	12	-1.170217136	0% (0.29)
A0JP11_HUMAN (+1)	AT1B1_HUMAN	ATP1B1	P05026	Sodium/potassium-transporting ATPase subunit beta-1 (Sodium/potassium-dependent ATPase subunit beta-1)	35 kDa	0.052	42	24	18	11	5	4	4	3	3	6	6	-1.171619128	0% (0.35)
ZN292_HUMAN	B4DKM6_HUMAN		B4DKM6	cDNA FLJ54305, highly similar to Serum paraoxonase/arylesterase 2 (EC 3.1.1.2)	40 kDa	0.0084	42	24	18	7	5	5	8	3	5	3	7	-1.171619128	0% (0.35)

B4DP99_HUMAN (+3)	NCBP1_HUMAN	NCBP1	Q09161	Nuclear cap-binding protein subunit 1 (80 kDa nuclear cap-binding protein) (CBP80) (NCBP 80 kDa subunit)	92 kDa	0.093	49	28	21	2	11	11	4	9	2	7	3	-1.174235591	0% (0.33)
WDR75_HUMAN	F8W1A4_HUMAN	AK2	F8W1A4	Adenylate kinase 2, mitochondrial	26 kDa	0.12	100	57	43	11	13	18	15	10	6	14	13	-1.175889305	0% (0.24)
B2RCG8_HUMAN (+2)	ERP44_HUMAN	ERP44	Q9BS26	Endoplasmic reticulum resident protein 44 (ER protein 44) (ERp44) (Thioredoxin domain-containing protein 4)	47 kDa	0.08	159	90	68	20	31	22	16	12	14	16	24	-1.177131235	0% (0.17)
SE1L1_HUMAN	B7Z1R5_HUMAN	ATP6V1A	B7Z1R5	V-type proton ATPase catalytic subunit A (cDNA FLJ51804, highly similar to Vacuolar ATP synthase catalytic subunit A, ubiquitous isoform (EC 3.6.3.14))	65 kDa	0.078	114	65	49	17	18	23	7	5	8	19	17	-1.177716522	0% (0.22)
B4DII5_HUMAN (+4)	LMNB1_HUMAN	LMNB1	P20700	Lamin-B1	66 kDa	0.16	237	135	102	34	41	33	29	33	6	35	29	-1.178954457	0% (0.12)
B3KM74_HUMAN (+4)	CCAR1_HUMAN	CCAR1	Q8IX12	Cell division cycle and apoptosis regulator protein 1 (Cell cycle and apoptosis regulatory protein 1) (CARP-1) (Death inducer with SAP domain)	133 kDa	0.13	11	7	5	3	1	2	2	1	0	1	3	-1.179006196	0% (0.47)
DHRS4_HUMAN	LSM1_HUMAN	LSM1	O15116	U6 snRNA-associated Sm-like protein LSm1 (Cancer-associated Sm-like) (Small nuclear ribonuclear CaSm)	15 kDa	0.14	12	7	5	0	3	3	1	2	3	0	0	-1.179006196	0% (0.47)
RBM14_HUMAN	SEH1_HUMAN	SEH1L	Q96EE3	Nucleoporin SEH1 (Nup107-160 subcomplex subunit SEH1) (SEC13-like protein)	40 kDa	0.096	11	7	5	1	3	3	1	2	1	2	0	-1.179006196	0% (0.47)
B3KPZ2_HUMAN (+1)	SNX5_HUMAN	SNX5	Q9Y5X3	Sorting nexin-5	47 kDa	0.11	13	7	5	0	2	2	4	1	2	1	1	-1.179006196	0% (0.47)
Q53FV3_HUMAN	Q53TD0_HUMAN	SP100	Q53TD0	Putative uncharacterized protein SP100 (Fragment)	97 kDa	0.11	12	7	5	2	1	1	3	1	0	2	2	-1.179006196	0% (0.47)
CBPM_HUMAN	B4DHS3_HUMAN		B4DHS3	cDNA FLJ57984, highly similar to Nucleolysin TIAR	20 kDa	0.038	12	7	5	0	2	3	2	0	1	2	2	-1.179006196	0% (0.47)
S27A4_HUMAN	A8K894_HUMAN		A8K894	cDNA FLJ77927	60 kDa	0.11	12	7	5	0	2	0	5	0	1	4	0	-1.179006196	0% (0.47)
SAHH3_HUMAN	NUD19_HUMAN	NUDT19	A8MXV4	Nucleoside diphosphate-linked moiety X motif 19, mitochondrial (Nudix motif 19) (EC 3.6.1.-)	42 kDa	0.08	12	7	5	4	1	2	0	3	1	1	0	-1.179006196	0% (0.47)
I7EQF7_HUMAN	S10AG_HUMAN	S100A16	Q96FQ6	Protein S100-A16 (Aging-associated gene 13 protein) (Protein S100-F) (S100 calcium-binding protein A16)	12 kDa	0.46	12	7	5	2	2	1	2	2	1	1	1	-1.179006196	0% (0.47)
H0Y2S9_HUMAN	Q96B24_HUMAN	ITM2B	Q96B24	Integral membrane protein 2B	30 kDa	0.015	12	7	5	1	4	1	1	3	1	0	0	-1.179006196	0% (0.47)
IFIT3_HUMAN	SYFM_HUMAN	FARS2	O95363	Phenylalanine--tRNA ligase, mitochondrial (EC 6.1.1.20) (Phenylalanyl-tRNA synthetase) (PheRS)	52 kDa	0.19	12	7	5	0	2	2	3	3	0	0	2	-1.179006196	0% (0.47)
ATG9A_HUMAN (+1)	MGN2_HUMAN	MAGOHB	Q96A72	Protein mago nashi homolog 2	17 kDa	0.048	71	40	30	7	9	10	14	10	7	8	5	-1.17908213	0% (0.28)
B3KNB4_HUMAN	B4E0H8_HUMAN		B4E0H8	cDNA FLJ60385, highly similar to Integrin alpha-3	115 kDa	0.17	70	40	30	16	10	11	3	10	0	9	12	-1.17908213	0% (0.28)
1433B_HUMAN (+1)	C9JZR2_HUMAN	CTNND1	C9JZR2	Catenin delta-1	105 kDa	0.26	128	73	55	21	16	13	23	18	5	19	13	-1.179158128	0% (0.20)
LYSC_HUMAN	ERP29_HUMAN	ERP29	P30040	Endoplasmic reticulum resident protein 29 (ERp29) (Endoplasmic reticulum resident protein 28) (ERp28) (Endoplasmic reticulum resident protein 31) (ERp31)	29 kDa	0.16	154	89	67	21	21	23	26	11	16	15	25	-1.181291313	0% (0.17)
WDR5_HUMAN	ARP3_HUMAN	ACTR3	P61158	Actin-related protein 3 (Actin-like protein 3)	47 kDa	0.12	98	56	42	13	14	15	14	14	10	11	7	-1.182425622	0% (0.23)

SNX3_HUMAN	3HIDH_HUMAN	HIBADH	P31937	3-hydroxyisobutyrate dehydrogenase, mitochondrial (HIBADH) (EC 1.1.1.31)	35 kDa	0.19	177	101	76	28	25	22	26	21	17	16	23	-1.182464308	0% (0.15)
A8K4R1_HUMAN (+2)	CSTF3_HUMAN	CSTF3	Q12996	Cleavage stimulation factor subunit 3 (CF-1 77 kDa subunit) (Cleavage stimulation factor 77 kDa subunit) (CSTF 77 kDa subunit) (CstF-77)	83 kDa	0.076	19	11	8	1	4	4	2	2	0	3	3	-1.182878371	0% (0.42)
ANAG_HUMAN	G0XQ39_HUMAN	STIM1	G0XQ39	STIM1L	89 kDa	0.067	18	11	8	5	3	3	1	0	2	2	4	-1.182878371	0% (0.42)
SNTB2_HUMAN	ARHL2_HUMAN	ADPRHL2	Q9NX46	Poly(ADP-ribose) glycohydrolase ARH3 (EC 3.2.1.143) (ADP-ribosylhydrolase 3) ([Protein ADP-ribosylarginine] hydrolase-like protein 2)	39 kDa	0.041	19	11	8	2	2	5	2	5	0	2	1	-1.182878371	0% (0.42)
NFKB1_HUMAN	B2L13_HUMAN	BCL2L13	Q9BXK5	Bcl-2-like protein 13 (Bcl2-L-13) (Bcl-rambo) (Protein Mil1)	53 kDa	0.031	19	11	8	5	2	2	2	5	1	2	0	-1.182878371	0% (0.42)
PPP6_HUMAN	Q05BS6_HUMAN	UNC93B1	Q05BS6	UNC93B1 protein (Fragment)	57 kDa	0.11	19	11	8	4	1	1	5	1	2	2	3	-1.182878371	0% (0.42)
FUCO2_HUMAN	J3QQX2_HUMAN	ARHGDIA	J3QQX2	Rho GDP-dissociation inhibitor 1	26 kDa	0.1	19	11	8	1	4	4	2	3	3	1	1	-1.182878371	0% (0.42)
A6XND9_HUMAN (+3)	B1PL87_HUMAN		B1PL87	Bid (Fragment)	15 kDa	0.23	19	11	8	1	3	4	3	2	5	1	0	-1.182878371	0% (0.42)
LGAT1_HUMAN	F5GZY0_HUMAN	APLP2	F5GZY0	Amyloid-like protein 2	76 kDa	0.15	26	15	11	7	6	3	0	3	1	3	4	-1.184858778	0% (0.39)
PA1B3_HUMAN	SYCM_HUMAN	CARS2	Q9HA77	Probable cysteine--tRNA ligase, mitochondrial (EC 6.1.1.16) (Cysteinyl-tRNA synthetase) (CysRS)	62 kDa	0.15	26	15	11	7	4	4	0	5	1	0	5	-1.184858778	0% (0.39)
PTN12_HUMAN	NAGK_HUMAN	NAGK	Q9UJ70	N-acetyl-D-glucosamine kinase (N-acetylglucosamine kinase) (EC 2.7.1.59) (GlcNAc kinase)	37 kDa	0.058	26	15	11	3	0	1	11	0	8	1	3	-1.184858778	0% (0.39)
C9J2G3_HUMAN (+2)	CYTB_HUMAN	CSTB	P04080	Cystatin-B (CPI-B) (Liver thiol proteinase inhibitor) (Stefin-B)	11 kDa	0.16	26	15	11	4	3	5	3	1	3	3	4	-1.184858778	0% (0.39)
B4E0B1_HUMAN (+1)	B3KMX0_HUMAN		B3KMX0	cDNA FLJ12837 fis, clone NT2RP2003228, highly similar to DNA replication licensing factor MCM4	97 kDa	0.079	33	19	14	0	10	8	1	8	1	5	0	-1.18606387	0% (0.36)
BCS1_HUMAN (+1)	ALG5_HUMAN	ALG5	Q9Y673	Dolichyl-phosphate beta-glucosyltransferase (DolP-glucosyltransferase) (EC 2.4.1.117) (Asparagine-linked glycosylation protein 5 homolog)	37 kDa	0.22	32	19	14	10	1	6	2	4	1	1	8	-1.18606387	0% (0.36)
D6W5X3_HUMAN (+3)	RAD21_HUMAN	RAD21	O60216	Double-strand-break repair protein rad21 homolog (hHR21) (Nuclear matrix protein 1) (NXP-1) (SCC1 homolog)	72 kDa	0.098	33	19	14	5	7	4	3	6	0	8	0	-1.18606387	0% (0.36)
NIF3L_HUMAN (+1)	B4E1R7_HUMAN		B4E1R7	cDNA FLJ58224, highly similar to Calpain-2 catalytic subunit (EC 3.4.22.53)	61 kDa	0.14	40	23	17	8	8	6	1	3	9	3	2	-1.186875995	0% (0.34)
NMT1_HUMAN (+1)	RECQ1_HUMAN	RECQL	P46063	ATP-dependent DNA helicase Q1 (EC 3.6.4.12) (DNA helicase, RecQ-like type 1) (RecQ1) (DNA-dependent ATPase Q1) (RecQ protein-like 1)	73 kDa	0.11	47	27	20	0	11	9	7	8	10	2	0	-1.187461587	0% (0.32)
D7PBN3_HUMAN	D3DV26_HUMAN	S100A10	D3DV26	S100 calcium binding protein A10 (Annexin II ligand, calpactin I, light polypeptide (P11)), isoform CRA_b (Fragment)	22 kDa	0.039	54	31	23	14	5	6	6	3	6	5	9	-1.18790472	0% (0.30)
PLIN3_HUMAN	B4E1U9_HUMAN		B4E1U9	cDNA FLJ54776, highly similar to Cell division control protein 42 homolog	27 kDa	0.15	61	35	26	10	7	3	15	8	6	6	6	-1.188252446	0% (0.29)
S15A3_HUMAN	B7Z7A8_HUMAN		B7Z7A8	cDNA FLJ50722, highly similar to Surfeit locus protein 4	29 kDa	0.041	75	43	32	11	15	7	10	3	10	10	9	-1.188764972	0% (0.26)

IQGA2_HUMAN	VINC_HUMAN	VCL	P18206	Vinculin (Metavinculin) (MV)	124 kDa	0.052	81	47	35	10	17	11	10	10	1	18	5	-1.188960051	0% (0.25)
THOC1_HUMAN	RAP1A_HUMAN	RAP1A	P62834	Ras-related protein Rap-1A (C21KG) (G-22K) (GTP-binding protein smg p21A) (Ras-related protein Krev-1)	21 kDa	0.075	82	47	35	12	10	13	11	7	10	11	7	-1.188960051	0% (0.25)
Q5JW30_HUMAN	A8K781_HUMAN		A8K781	cDNA FLJ75299, highly similar to Xenopus laevis proteasome (prosome, macropain) 26S subunit, ATPase 3, mRNA	47 kDa	0.075	88	51	38	6	15	13	17	11	12	11	4	-1.189126812	0% (0.24)
OGDHL_HUMAN	MX1_HUMAN	MX1	P20591	Interferon-induced GTP-binding protein Mx1 (Interferon-induced protein p78) (IFI-78K) (Interferon-regulated resistance GTP-binding protein MxA) (Myxoma resistance protein 1) (Myxovirus resistance protein 1) [Cleaved into: Interferon-induced GTP-binding protein Mx1, N-terminally processed]	76 kDa	0.16	95	54	40	0	6	10	38	2	18	15	7	-1.196448795	0% (0.22)
B7ZW51_HUMAN (+8)	LMNA_HUMAN	LMNA	P02545	Prelamin-A/C [Cleaved into: Lamin-A/C (70 kDa lamin) (Renal carcinoma antigen NY-REN-32)]	74 kDa	0.15	605	346	258	117	81	78	76	85	31	63	81	-1.197231023	95% (0.017)
PAR14_HUMAN	THIK_HUMAN	ACAA1	P09110	3-ketoacyl-CoA thiolase, peroxisomal (EC 2.3.1.16) (Acetyl-CoA acyltransferase) (Beta-ketothiolase) (Peroxisomal 3-oxoacyl-CoA thiolase)	44 kDa	0.035	73	42	31	15	7	8	12	8	11	7	5	-1.197929982	0% (0.25)
B7ZKT7_HUMAN (+1)	M0R0F0_HUMAN	RPS5	M0R0F0	40S ribosomal protein S5 (Fragment)	22 kDa	0.035	70	38	28	8	9	7	15	10	9	4	5	-1.198630298	0% (0.26)
E7ET40_HUMAN (+2)	RL12_HUMAN	RPL12	P30050	60S ribosomal protein L12	18 kDa	0.14	66	38	28	7	8	10	13	8	7	7	6	-1.198630298	0% (0.26)
J3K000_HUMAN (+1)	Q32Q62_HUMAN	RSL1D1	Q32Q62	RSL1D1 protein (Fragment)	52 kDa	0.053	45	26	19	4	5	6	11	4	4	4	7	-1.201993719	0% (0.31)
PSB2_HUMAN	B2R806_HUMAN	EIF3E	B2R806	Eukaryotic translation initiation factor 3 subunit E (eIF3e) (Eukaryotic translation initiation factor 3 subunit 6) (eIF-3 p48)	52 kDa	0.26	38	22	16	0	8	7	7	3	9	3	1	-1.203901615	0% (0.33)
ENPP4_HUMAN	J3KQU9_HUMAN	AP1G1	J3KQU9	AP-1 complex subunit gamma-1	92 kDa	0.075	38	22	16	5	7	6	4	7	1	6	2	-1.203901615	0% (0.33)
CPSF2_HUMAN	EFTS_HUMAN	TSFM	P43897	Elongation factor Ts, mitochondrial (EF-Ts) (EF-TsMt)	35 kDa	0.052	38	22	16	4	6	7	5	7	2	3	4	-1.203901615	0% (0.33)
KRR1_HUMAN	A8K2H6_HUMAN		A8K2H6	Lipase	45 kDa	0.092	37	22	16	5	5	7	4	2	0	6	8	-1.203901615	0% (0.33)
GUAA_HUMAN (+1)	VATC1_HUMAN	ATP6V1C1	P21283	V-type proton ATPase subunit C 1 (V-ATPase subunit C 1) (Vacuolar proton pump subunit C 1)	44 kDa	0.098	31	18	13	2	5	4	8	2	4	4	3	-1.206616971	0% (0.35)
B4DHS3_HUMAN	A8K8A4_HUMAN		A8K8A4	cDNA FLJ77640, highly similar to Homo sapiens copine II (CPNE2), mRNA	61 kDa	0.17	32	18	13	6	5	4	3	0	2	5	4	-1.206616971	0% (0.35)
B3KUY2_HUMAN (+2)	B4DZK8_HUMAN		B4DZK8	cDNA FLJ61192, highly similar to Erythrocyte band 7 integral membrane protein	31 kDa	0.16	64	37	27	9	10	8	10	2	6	7	12	-1.209442192	0% (0.26)
MRP4_HUMAN	PRP6_HUMAN	PRPF6	O94906	Pre-mRNA-processing factor 6 (Androgen receptor N-terminal domain-transactivating protein 1) (ANT-1) (PRP6 homolog) (U5 snRNP-associated 102 kDa protein) (U5-102 kDa protein)	107 kDa	0.032	23	14	10	4	4	3	3	0	1	6	3	-1.210785753	0% (0.37)
NDUS5_HUMAN	R4GN98_HUMAN	S100A6	R4GN98	Protein S100-A6 (Fragment)	10 kDa	0.42	27	14	10	3	3	4	4	3	4	1	2	-1.210785753	0% (0.37)
PARP4_HUMAN	SPB9_HUMAN	SERPINB9	P50453	Serpin B9 (Cytoplasmic antiproteinase 3) (CAP-3)	42 kDa	0.026	24	14	10	1	4	6	3	0	5	2	0	-1.210785753	0% (0.37)

				(CAP3) (Peptidase inhibitor 9) (PI-9)															
SRP68_HUMAN	H3BT71_HUMAN	RBMX	H3BT71	RNA-binding motif protein, X chromosome, N-terminally processed	32 kDa	0.091	57	33	24	8	7	9	9	7	2	6	9	-1.211623646	0% (0.27)
Q8N355_HUMAN	B2R8J0_HUMAN		B2R8J0	cDNA, FLJ93923, highly similar to Homo sapiens UDP-N-acetyl-alpha-D-galactosamine:polypeptide N-acetylgalactosaminyltransferase 3 (GalNAc-T3) (GALNT3), mRNA	73 kDa	0.15	56	33	24	10	8	9	6	6	7	4	7	-1.211623646	0% (0.27)
F5GXA0_HUMAN	OGDHL_HUMAN	OGDHL	Q9ULD0	2-oxoglutarate dehydrogenase-like, mitochondrial (EC 1.2.4.-) (2-oxoglutarate dehydrogenase complex component E1-like) (OGDC-E1-like) (Alpha-ketoglutarate dehydrogenase-like)	114 kDa	0.07	57	33	24	16	0	5	0	11	0	7	4	-1.211623646	0% (0.27)
B4DXV9_HUMAN (+1)	GPX1_HUMAN	GPX1	P07203	Glutathione peroxidase 1 (GPx-1) (GSHPx-1) (EC 1.11.1.9) (Cellular glutathione peroxidase)	22 kDa	0.2	90	52	38	13	13	16	10	3	13	8	14	-1.2119008	0% (0.21)
IF4G3_HUMAN (+1)	CD47_HUMAN	CD47	Q08722	Leukocyte surface antigen CD47 (Antigenic surface determinant protein OA3) (Integrin-associated protein) (IAP) (Protein MER6) (CD antigen CD47)	35 kDa	0.03	17	10	7	3	2	3	3	1	2	2	2	-1.217993515	0% (0.41)
B2R5U7_HUMAN (+1)	SC61G_HUMAN	SEC61G	P60059	Protein transport protein Sec61 subunit gamma	8 kDa	0.043	20	10	7	3	2	0	6	1	3	0	3	-1.217993515	0% (0.41)
HELZ2_HUMAN	FABD_HUMAN	MCAT	Q8IVS2	Malonyl-CoA-acyl carrier protein transacylase, mitochondrial (MCT) (EC 2.3.1.39) (Mitochondrial malonyl CoA:ACP acyltransferase) (Mitochondrial malonyltransferase) ([Acyl-carrier-protein] malonyltransferase)	43 kDa	0.015	17	10	7	6	0	4	1	1	1	1	4	-1.217993515	0% (0.41)
K6PF_HUMAN	STX8_HUMAN	STX8	Q9UNK0	Syntaxin-8	27 kDa	0.064	17	10	7	4	1	4	1	0	1	2	4	-1.217993515	0% (0.41)
S35B2_HUMAN	VATG1_HUMAN	ATP6V1G1	O75348	V-type proton ATPase subunit G 1 (V-ATPase subunit G 1) (V-ATPase 13 kDa subunit 1) (Vacuolar proton pump subunit G 1) (Vacuolar proton pump subunit M16)	14 kDa	0.086	17	10	7	1	2	6	1	0	2	2	3	-1.217993515	0% (0.41)
B1ALA9_HUMAN (+1)	RS18_HUMAN	RPS18	P62269	40S ribosomal protein S18 (Ke-3) (Ke3)	18 kDa	0.18	43	25	18	4	4	9	10	6	5	4	3	-1.21803554	0% (0.30)
PRG2_HUMAN	E7ESI6_HUMAN	TXNRD1	E7ESI6	Thioredoxin reductase 1, cytoplasmic	64 kDa	0.18	43	25	18	5	7	7	6	4	4	9	1	-1.21803554	0% (0.30)
CTBP1_HUMAN (+1)	I6RUN0_HUMAN	HLA-A	I6RUN0	MHC class I antigen (Fragment)	21 kDa	0.055	88	51	37	0	0	11	21	0	8	13	12	-1.220232656	0% (0.20)
DPP2_HUMAN	ACTN4_HUMAN	ACTN4	O43707	Alpha-actinin-4 (F-actin cross-linking protein) (Non-muscle alpha-actinin 4)	105 kDa	0.069	431	249	182	51	61	65	73	56	18	65	43	-1.220443144	95% (0.024)
B4DH46_HUMAN (+2)	TERA_HUMAN	VCP	P55072	Transitional endoplasmic reticulum ATPase (TER ATPase) (EC 3.6.4.6) (15S Mg(2+)-ATPase p97 subunit) (Valosin-containing protein) (VCP)	89 kDa	0.19	314	181	132	29	55	70	28	30	30	43	31	-1.22213831	95% (0.046)
Q5SWX3_HUMAN	ACTN1_HUMAN	ACTN1	P12814	Alpha-actinin-1 (Alpha-actinin cytoskeletal isoform) (F-actin cross-linking protein) (Non-muscle alpha-actinin-1)	103 kDa	0.062	234	136	99	26	35	43	32	34	15	29	21	-1.223215309	0% (0.073)
PCNA_HUMAN (+1)	S10AB_HUMAN	S100A11	P31949	Protein S100-A11 (Calgizzarin) (Metastatic lymph node gene 70 protein) (MLN 70) (Protein S100-C) (S100 calcium-binding protein A11) [Cleaved into: Protein	12 kDa	0.13	113	65	47	18	16	15	18	11	12	8	16	-1.226569098	0% (0.16)

				S100-A11, N-terminally processed]															
B3KWE3_HUMAN (+14)	Q53H34_HUMAN		Q53H34	Ribosomal protein L13a variant (Fragment)	24 kDa	0.038	31	17	12	4	5	3	5	4	5	1	2	-1.23027222	0% (0.34)
B3KX82_HUMAN	ARF1_HUMAN	ARF1	P84077	ADP-ribosylation factor 1	21 kDa	0.037	78	46	33	11	8	12	15	10	7	8	8	-1.232327415	0% (0.20)
PFD2_HUMAN	RM21_HUMAN	MRPL21	Q7Z2W9	39S ribosomal protein L21, mitochondrial (L21mt) (MRP-L21)	23 kDa	0.17	10	6	4	4	1	0	1	1	1	0	2	-1.233449936	0% (0.45)
CDK1_HUMAN (+1)	A8K3C5_HUMAN		A8K3C5	cDNA FLJ77715, highly similar to Homo sapiens cisplatin resistance-associated overexpressed protein(LUC7A), mRNA	51 kDa	0.13	10	6	4	1	2	2	1	1	0	2	1	-1.233449936	0% (0.45)
I3VM54_HUMAN (+1)	DHRS4_HUMAN	DHRS4	Q9BTZ2	Dehydrogenase/reductase SDR family member 4 (EC 1.1.1.184) (NADPH-dependent carbonyl reductase/NADP-retinol dehydrogenase) (CR) (PHCR) (NADPH-dependent retinol dehydrogenase/reductase) (NRDR) (humNRDR) (Peroxisomal short-chain alcohol dehydrogenase) (PSCD) (SCAD-SRL) (Short-chain dehydrogenase/reductase family member 4)	30 kDa	0.021	10	6	4	3	0	1	2	1	1	2	1	-1.233449936	0% (0.45)
ANM5_HUMAN (+1)	PLBL2_HUMAN	PLBD2	Q8NHP8	Putative phospholipase B-like 2 (EC 3.1.1.-) (76 kDa protein) (p76) (LAMA-like protein 2) (Lamina ancestor homolog 2) (Phospholipase B domain-containing protein 2) [Cleaved into: Putative phospholipase B-like 2 32 kDa form; Putative phospholipase B-like 2 45 kDa form]	65 kDa	0.044	10	6	4	1	4	1	0	0	0	3	1	-1.233449936	0% (0.45)
THOC4_HUMAN	SRP14_HUMAN	SRP14	P37108	Signal recognition particle 14 kDa protein (SRP14) (18 kDa Alu RNA-binding protein)	15 kDa	0.093	10	6	4	1	0	1	4	1	1	1	1	-1.233449936	0% (0.45)
MSLN_HUMAN	TMM70_HUMAN	TMEM70	Q9BUB7	Transmembrane protein 70, mitochondrial	29 kDa	0.1	10	6	4	1	1	1	3	1	1	1	1	-1.233449936	0% (0.45)
XPP3_HUMAN	B2RBH6_HUMAN		B2RBH6	cDNA, FLJ95513, highly similar to Homo sapiens cyclin fold protein 1 (CFP1), mRNA	39 kDa	0.097	10	6	4	3	1	0	2	0	0	3	1	-1.233449936	0% (0.45)
A8K2N5_HUMAN (+4)	B7Z792_HUMAN		B7Z792	cDNA FLJ53932, highly similar to NADH-ubiquinone oxidoreductase 49 kDa subunit, mitochondrial (EC 1.6.5.3)	46 kDa	0.058	41	24	17	13	4	4	3	4	1	2	10	-1.235835218	0% (0.29)
F8J390_HUMAN (+1)	VATL_HUMAN	ATP6V0C	P27449	V-type proton ATPase 16 kDa proteolipid subunit (V-ATPase 16 kDa proteolipid subunit) (Vacuolar proton pump 16 kDa proteolipid subunit)	16 kDa	0.062	41	24	17	10	7	3	4	0	6	6	5	-1.235835218	0% (0.29)
NIPBL_HUMAN (+1)	ARC1B_HUMAN	ARPC1B	O15143	Actin-related protein 2/3 complex subunit 1B (Arp2/3 complex 41 kDa subunit) (p41-ARC)	41 kDa	0.0086	41	24	17	4	6	8	7	3	5	5	3	-1.235835218	0% (0.29)
PURA2_HUMAN	EIF3A_HUMAN	EIF3A	Q14152	Eukaryotic translation initiation factor 3 subunit A (eIF3a) (Eukaryotic translation initiation factor 3 subunit 10) (eIF-3-theta) (eIF3 p167) (eIF3 p180) (eIF3 p185)	167 kDa	0.14	72	42	30	8	14	11	9	4	13	8	5	-1.236281532	0% (0.21)
2ABA_HUMAN	E9PH29_HUMAN	PRDX3	E9PH29	Thioredoxin-dependent peroxide reductase, mitochondrial	26 kDa	0.092	165	96	69	28	27	18	24	19	18	14	18	-1.236751185	0% (0.10)
B4DZS8_HUMAN (+1)	ACADV_HUMAN	ACADVL	P49748	Very long-chain specific acyl-CoA dehydrogenase, mitochondrial (VLCAD) (EC 1.3.8.9)	70 kDa	0.066	289	168	121	79	38	29	22	28	25	26	45	-1.237074891	95% (0.043)
B2RE59_HUMAN (+2)	ABD12_HUMAN	ABHD12	Q8N2K0	Monoacylglycerol lipase ABHD12 (EC 3.1.1.23) (2-arachidonoylglycerol	45 kDa	0.07	83	49	35	11	14	13	12	8	3	7	17	-1.238275207	0% (0.19)

				hydrolase) (Abhydrolase domain-containing protein 12)															
RM41_HUMAN	A8K2H4_HUMAN		A8K2H4	cDNA FLJ78235	38 kDa	0.23	127	74	53	20	19	22	14	5	7	16	26	-1.239147396	0% (0.13)
L0R6Q1_HUMAN	Q53EM5_HUMAN		Q53EM5	Transketolase variant (Fragment)	68 kDa	0.19	64	38	27	6	9	8	15	3	17	4	3	-1.241077548	0% (0.22)
TMM65_HUMAN	CLIC5_HUMAN	CLIC5	Q9NZA1	Chloride intracellular channel protein 5	47 kDa	0.13	22	13	9	1	1	1	10	4	4	1	0	-1.241771383	0% (0.36)
TM41A_HUMAN	NDUAB_HUMAN	NDUFA11	Q86Y39	NADH dehydrogenase [ubiquinone] 1 alpha subcomplex subunit 11 (Complex I-B14.7) (CI-B14.7) (NADH-ubiquinone oxidoreductase subunit B14.7)	15 kDa	0.083	22	13	9	3	4	3	2	4	1	3	1	-1.241771383	0% (0.36)
TMED5_HUMAN	B4DWH7_HUMAN		B4DWH7	cDNA FLJ57632, highly similar to Vacuolar ATP synthase subunit B, kidney isoform (EC 3.6.3.14)	54 kDa	0.17	120	70	50	0	20	16	21	13	0	18	0	-1.241948597	0% (0.14)
SNP23_HUMAN	EBP_HUMAN	EBP	Q15125	3-beta-hydroxysteroid-Delta(8),Delta(7)-isomerase (EC 5.3.3.5) (Cholestenol Delta-isomerase) (Delta(8)-Delta(7) sterol isomerase) (D8-D7 sterol isomerase) (Emopamil-binding protein)	26 kDa	0.15	36	20	14	7	3	2	8	5	3	4	2	-1.24465092	0% (0.30)
CLCC1_HUMAN	E9PAV3_HUMAN	NACA	E9PAV3	Nascent polypeptide-associated complex subunit alpha	205 kDa	0.04	34	20	14	2	7	5	6	3	4	4	3	-1.24465092	0% (0.30)
E5KSX8_HUMAN (+2)	RPR1B_HUMAN	RPRD1B	Q9NQG5	Regulation of nuclear pre-mRNA domain-containing protein 1B (Cell cycle-related and expression-elevated protein in tumor)	37 kDa	0.19	34	20	14	0	6	3	11	1	5	6	2	-1.24465092	0% (0.30)
O19707_HUMAN	SPRY4_HUMAN	SPRYD4	Q8WW59	SPRY domain-containing protein 4	23 kDa	0.33	34	20	14	4	4	3	9	7	2	1	4	-1.24465092	0% (0.30)
B7Z418_HUMAN (+1)	MYOF_HUMAN	MYOF	Q9NZM1	Myoferlin (Fer-1-like protein 3)	235 kDa	0.12	492	287	205	167	56	47	20	32	42	46	83	-1.249198591	95% (0.0090)
PPAP_HUMAN (+1)	ANXA4_HUMAN	ANXA4	P09525	Annexin A4 (35-beta calcimedin) (Annexin IV) (Annexin-4) (Carbohydrate-binding protein p33/p41) (Chromobindin-4) (Endonexin I) (Lipocortin IV) (P32.5) (PP4-X) (Placental anticoagulant protein II) (PAP-II) (Protein II)	36 kDa	0.17	184	107	76	52	19	15	21	17	24	13	23	-1.251947601	0% (0.076)
DGAT1_HUMAN (+1)	A8K3B0_HUMAN		A8K3B0	cDNA FLJ77877, highly similar to Human ENO2 neuron specific (gamma) enolase	47 kDa	0.082	63	37	26	0	8	7	19	9	6	9	0	-1.253843434	0% (0.21)
THOC6_HUMAN	B2R9S4_HUMAN		B2R9S4	cDNA, FLJ94534, highly similar to Homo sapiens capping protein (actin filament), gelsolin-like(CAPG), mRNA	38 kDa	0.073	27	16	11	4	3	3	6	1	2	3	5	-1.257789327	0% (0.32)
Q53G62_HUMAN (+1)	Q96K24_HUMAN		Q96K24	cDNA FLJ14836 fis, clone OVARC1001702	13 kDa	0.19	26	16	11	4	3	4	4	0	3	4	4	-1.257789327	0% (0.32)
B4DP77_HUMAN (+1)	RS10_HUMAN	RPS10	P46783	40S ribosomal protein S10	19 kDa	0.24	55	33	23	5	8	10	9	7	11	4	2	-1.261605661	0% (0.22)
GNL3_HUMAN	B2R4G1_HUMAN		B2R4G1	cDNA, FLJ92076, Homo sapiens likely ortholog of mouse hypoxia induced gene 1(HIG1), mRNA	10 kDa	0.11	17	9	6	4	1	1	3	0	2	2	2	-1.262795217	0% (0.39)
LSM4_HUMAN	RU2B_HUMAN	SNRPB2	P08579	U2 small nuclear ribonucleoprotein B'' (U2 snRNP B'')	25 kDa	0.12	15	9	6	1	2	3	3	0	0	2	4	-1.262795217	0% (0.39)
DHPR_HUMAN	PRKRA_HUMAN	PRKRA	O75569	Interferon-inducible double-stranded RNA-dependent protein kinase activator A (PKR-associated protein X) (PKR-associating protein X) (Protein activator of the	34 kDa	0.25	14	9	6	0	4	2	2	2	1	2	1	-1.262795217	0% (0.39)

				interferon-induced protein kinase) (Protein kinase, interferon-inducible double-stranded RNA-dependent activator)															
A1L3A9_HUMAN (+2)	KC1A_HUMAN	CSNK1A1	P48729	Casein kinase I isoform alpha (CKI-alpha) (EC 2.7.11.1) (CK1)	39 kDa	0.022	15	9	6	4	1	2	2	2	2	0	2	-1.262795217	0% (0.39)
B4DKK2_HUMAN (+2)	ACOT8_HUMAN	ACOT8	O14734	Acyl-coenzyme A thioesterase 8 (Acyl-CoA thioesterase 8) (EC 3.1.2.27) (Choloyl-coenzyme A thioesterase) (HIV-Nef-associated acyl-CoA thioesterase) (PTE-2) (Peroxisomal acyl-coenzyme A thioester hydrolase 1) (PTE-1) (Peroxisomal long-chain acyl-CoA thioesterase 1) (Thioesterase II) (hACTE-III) (hACTEIII) (hTE)	36 kDa	0.27	15	9	6	3	2	2	2	3	0	1	2	-1.262795217	0% (0.39)
A8KAQ3_HUMAN (+1)	DGAT1_HUMAN	DGAT1	O75907	Diacylglycerol O-acyltransferase 1 (EC 2.3.1.20) (ACAT-related gene product 1) (Acyl-CoA retinol O-fatty-acyltransferase) (ARAT) (Retinol O-fatty-acyltransferase) (EC 2.3.1.76) (Diglyceride acyltransferase)	55 kDa	0.064	14	9	6	2	3	0	4	2	0	3	1	-1.262795217	0% (0.39)
1433F_HUMAN	ARL8B_HUMAN	ARL8B	Q9NVJ2	ADP-ribosylation factor-like protein 8B (ADP-ribosylation factor-like protein 10C) (Novel small G protein indispensable for equal chromosome segregation 1)	22 kDa	0.19	84	50	35	18	12	12	8	3	12	8	13	-1.262933736	0% (0.16)
B4DK32_HUMAN (+1)	A8K132_HUMAN		A8K132	cDNA FLJ75476, highly similar to Homo sapiens glutaminase (GLS), mRNA	74 kDa	0.067	84	50	35	34	9	8	0	10	0	5	20	-1.262933736	0% (0.16)
NUDC1_HUMAN	J3KTL2_HUMAN	SRSF1	J3KTL2	Serine/arginine-rich-splicing factor 1 (Splicing factor, arginine/serine-rich 1 (Splicing factor 2, alternate splicing factor), isoform CRA_g)	28 kDa	0.11	73	43	30	6	11	8	18	7	3	12	8	-1.264882336	0% (0.18)
B4DQ14_HUMAN (+2)	CP27A_HUMAN	CYP27A1	Q02318	Sterol 26-hydroxylase, mitochondrial (EC 1.14.13.15) (5-beta-cholestane-3-alpha,7-alpha,12-alpha-triol 27-hydroxylase) (Cytochrome P-450C27/25) (Cytochrome P450 27) (Sterol 27-hydroxylase) (Vitamin D(3) 25-hydroxylase)	60 kDa	0.1	119	70	49	24	18	25	3	0	4	7	38	-1.266682226	0% (0.12)
J3QT16_HUMAN	RL15_HUMAN	RPL15	P61313	60S ribosomal protein L15	24 kDa	0.042	90	53	37	12	13	9	19	9	11	7	9	-1.266972703	0% (0.15)
B9EK46_HUMAN (+1)	FUBP2_HUMAN	KHSRP	Q92945	Far upstream element-binding protein 2 (FUSE-binding protein 2) (KH type-splicing regulatory protein) (KSRP) (p75)	73 kDa	0.022	32	19	13	2	6	8	3	2	1	8	2	-1.269314684	0% (0.29)
EI2BG_HUMAN	GHC1_HUMAN	SLC25A22	Q9H936	Mitochondrial glutamate carrier 1 (GC-1) (Glutamate/H(+) symporter 1) (Solute carrier family 25 member 22)	34 kDa	0.1	30	19	13	3	3	3	9	3	2	4	3	-1.269314684	0% (0.29)
CAH1_HUMAN (+1)	OXSR1_HUMAN	OXSR1	O95747	Serine/threonine-protein kinase OSR1 (EC 2.7.11.1) (Oxidative stress-responsive 1 protein)	58 kDa	0.26	32	19	13	2	4	10	3	6	4	1	2	-1.269314684	0% (0.29)
NTPCR_HUMAN (+1)	1A69_HUMAN	HLA-A	P10316	HLA class I histocompatibility antigen, A-69 alpha chain (Aw-69) (HLA class I histocompatibility antigen, A-28 alpha chain) (MHC class I antigen A*69)	41 kDa	0.29	124	73	51	10	18	18	28	6	11	17	16	-1.269501705	0% (0.11)
ABCF3_HUMAN (+1)	NDUV2_HUMAN	NDUFV2	P19404	NADH dehydrogenase [ubiquinone] flavoprotein 2, mitochondrial (EC 1.6.5.3) (EC 1.6.99.3) (NADH-ubiquinone oxidoreductase 24 kDa subunit)	27 kDa	0.038	49	29	20	11	8	4	6	5	4	4	8	-1.271562349	0% (0.23)

A2BDK6_HUMAN (+1)	IF2B3_HUMAN	IGF2BP3	O00425	Insulin-like growth factor 2 mRNA-binding protein 3 (IGF2 mRNA-binding protein 3) (IMP-3) (IGF-II mRNA-binding protein 3) (KH domain-containing protein overexpressed in cancer) (hKOC) (VICKZ family member 3)	64 kDa	0.015	100	59	41	4	15	12	29	21	5	14	1	-1.273907203	0% (0.13)
NQO1_HUMAN	Q9TP39_HUMAN	HLA-A	Q9TP39	MHC class I antigen (Fragment)	21 kDa	0.17	88	52	36	7	0	11	21	0	8	0	12	-1.277006057	0% (0.15)
DOCK7_HUMAN	PX11B_HUMAN	PEX11B	O96011	Peroxisomal membrane protein 11B (Peroxin-11B) (Peroxisomal biogenesis factor 11B) (Protein PEX11 homolog beta) (PEX11-beta)	28 kDa	0.024	36	22	15	6	4	2	10	3	7	0	5	-1.278006256	0% (0.26)
E7EQN5_HUMAN (+2)	A8MQ02_HUMAN	MLLT4	A8MQ02	Afadin	207 kDa	0.044	19	12	8	6	3	2	1	7	0	0	0	-1.279456311	0% (0.34)
B7Z920_HUMAN (+1)	IPO9_HUMAN	IPO9	Q96P70	Importin-9 (Imp9) (Ran-binding protein 9) (RanBP9)	116 kDa	0.035	19	12	8	0	4	5	3	2	5	1	0	-1.279456311	0% (0.34)
B3KN79_HUMAN	A8K4I8_HUMAN		A8K4I8	cDNA FLJ78131, highly similar to Homo sapiens nipsnap homolog 1 (C. elegans) (NIPSNAP1), mRNA	33 kDa	0.086	59	35	24	8	5	4	18	9	7	3	6	-1.282408358	0% (0.20)
TMLH_HUMAN	HEM6_HUMAN	CPOX	P36551	Coproporphyrinogen-III oxidase, mitochondrial (COX) (Coprogen oxidase) (Coproporphyrinogenase) (EC 1.3.3.3)	50 kDa	0.15	59	35	24	10	11	7	7	6	2	7	8	-1.282408358	0% (0.20)
G8JLD3_HUMAN (+1)	A8K5N5_HUMAN		A8K5N5	cDNA FLJ78142, highly similar to Homo sapiens transglutaminase 1 (K polypeptide epidermal type I, protein-glutamine-gamma-glutamyltransferase), mRNA	90 kDa	0.026	81	48	33	16	10	7	16	25	0	4	3	-1.284522448	0% (0.15)
A8K5K5_HUMAN (+4)	B4DYX8_HUMAN		B4DYX8	cDNA FLJ56179, highly similar to Pyruvate dehydrogenase (lipoamide)-phosphatase 1 (EC 3.1.3.43)	67 kDa	0.078	42	25	17	4	9	8	5	12	2	1	3	-1.284795699	0% (0.24)
KPCA_HUMAN (+1)	B2RTQ5_HUMAN	LRRC16A	B2RTQ5	LRRC16A protein	151 kDa	0.07	3	2	1	0	0	1	1	1	0	0	0	-1.290149649	0% (0.54)
B3KMR6_HUMAN (+1)	J3KMZ9_HUMAN	LDLR	J3KMZ9	Low-density lipoprotein receptor (Fragment)	105 kDa	0.078	3	2	1	0	1	1	0	1	0	0	0	-1.290149649	0% (0.54)
B4DSD7_HUMAN	RL8_HUMAN	RPL8	P62917	60S ribosomal protein L8	28 kDa	0.048	3	2	1	0	0	1	1	0	0	0	1	-1.290149649	0% (0.54)
B4DED6_HUMAN (+2)	HELZ2_HUMAN	HELZ2	Q9BYK8	Helicase with zinc finger domain 2 (ATP-dependent helicase PRIC285) (Helicase with zinc finger 2, transcriptional coactivator) (PPAR-alpha-interacting complex protein 285) (PPAR-gamma DNA-binding domain-interacting protein 1) (PDIP1) (PPAR-gamma DBD-interacting protein 1) (Peroxisomal proliferator-activated receptor A-interacting complex 285 kDa protein) (EC 3.6.4.-)	295 kDa	0.076	3	2	1	0	1	0	1	0	1	0	0	-1.290149649	0% (0.54)
Q53FA5_HUMAN	B4DST3_HUMAN		B4DST3	cDNA FLJ56487, highly similar to Methionine synthase (EC 2.1.1.13)	124 kDa	0.26	3	2	1	0	0	2	0	1	0	0	0	-1.290149649	0% (0.54)
ADA17_HUMAN	E7EVA0_HUMAN	MAP4	E7EVA0	Microtubule-associated protein	245 kDa	0.032	4	2	1	1	0	2	0	1	0	0	0	-1.290149649	0% (0.54)
NAGAB_HUMAN	HIP1_HUMAN	HIP1	O00291	Huntingtin-interacting protein 1 (HIP-1) (Huntingtin-interacting protein I) (HIP-I)	116 kDa	0.13	3	2	1	0	0	2	0	0	0	1	0	-1.290149649	0% (0.54)
B4DTS6_HUMAN (+2)	SERP3_HUMAN	SERPINE3	A8MV23	Serpin E3	47 kDa	0.057	3	2	1	0	1	0	1	0	1	0	0	-1.290149649	0% (0.54)

INO1_HUMAN	B7Z8W3_HUMAN		B7Z8W3	cDNA FLJ53272, highly similar to Homo sapiens LIM domain 7 (LMO7), mRNA	145 kDa	0.1	2	2	1	2	0	0	0	1	0	0	0	-1.290149649	0% (0.54)
B2RAG5_HUMAN (+1)	NASP_HUMAN	NASP	P49321	Nuclear autoantigenic sperm protein (NASP)	85 kDa	0.084	3	2	1	0	1	1	0	1	0	0	0	-1.290149649	0% (0.54)
CCD51_HUMAN	EPS8_HUMAN	EPS8	Q12929	Epidermal growth factor receptor kinase substrate 8	92 kDa	0.11	2	2	1	2	0	0	0	0	1	0	0	-1.290149649	0% (0.54)
TRFM_HUMAN	ARI1A_HUMAN	ARID1A	O14497	AT-rich interactive domain-containing protein 1A (ARID domain-containing protein 1A) (B120) (BRG1-associated factor 250) (BAF250) (BRG1-associated factor 250a) (BAF250A) (Osa homolog 1) (hOSA1) (SWI-like protein) (SWI/SNF complex protein p270) (SWI/SNF-related, matrix-associated, actin-dependent regulator of chromatin subfamily F member 1) (hELD)	242 kDa	0.089	3	2	1	1	1	0	0	0	0	1	0	-1.290149649	0% (0.54)
GBF1_HUMAN (+2)	A6NHH5_HUMAN	UPK3B	A6NHH5	Uroplakin-3b	30 kDa	0.03	4	2	1	2	0	0	0	1	0	0	0	-1.290149649	0% (0.54)
B4DST3_HUMAN (+3)	UIF_HUMAN	FYTTD1	Q96QD9	UAP56-interacting factor (Forty-two-three domain-containing protein 1) (Protein 40-2-3)	36 kDa	0.038	3	2	1	0	1	0	1	0	0	0	1	-1.290149649	0% (0.54)
D6R952_HUMAN (+2)	B4DL79_HUMAN	KIF20A	B4DL79	Kinesin-like protein KIF20A (cDNA FLJ55710, highly similar to Kinesin family member 20A)	98 kDa	0.083	2	2	1	0	0	1	0	1	0	0	0	-1.290149649	0% (0.54)
DHB7_HUMAN (+1)	SNTB1_HUMAN	SNTB1	Q13884	Beta-1-syntrophin (59 kDa dystrophin-associated protein A1 basic component 1) (DAPA1B) (BSYN2) (Syntrophin-2) (Tax interaction protein 43) (TIP-43)	58 kDa	0.11	4	2	1	0	0	0	2	0	0	0	1	-1.290149649	0% (0.54)
ARSA_HUMAN (+1)	SIA4A_HUMAN	ST3GAL1	Q11201	CMP-N-acetylneuraminate-beta-galactosamide-alpha-2,3-sialyltransferase 1 (Alpha 2,3-ST 1) (Beta-galactoside alpha-2,3-sialyltransferase 1) (EC 2.4.99.4) (Gal-NAc6S) (Gal-beta-1,3-GalNAc-alpha-2,3-sialyltransferase) (SIATFL) (ST3Gal I) (ST3GalI) (ST3GalA.1) (ST3O) (Sialyltransferase 4A) (SIAT4-A)	39 kDa	0.061	3	2	1	1	0	1	0	0	0	0	0	-1.290149649	0% (0.54)
F5H013_HUMAN (+2)	PAG15_HUMAN	PLA2G15	Q8NCC3	Group XV phospholipase A2 (EC 2.3.1.-) (1-O-acylceramide synthase) (ACS) (LCAT-like lysophospholipase) (LLPL) (Lysophospholipase 3) (Lysosomal phospholipase A2) (LPLA2)	47 kDa	0.29	3	2	1	0	1	1	0	0	0	1	0	-1.290149649	0% (0.54)
A5PLM9_HUMAN (+2)	VP33B_HUMAN	VPS33B	Q9H267	Vacuolar protein sorting-associated protein 33B (hVPS33B)	71 kDa	0.12	3	2	1	0	0	1	1	0	1	0	0	-1.290149649	0% (0.54)
B7Z4S4_HUMAN (+2)	PR38B_HUMAN	PRPF38B	Q5VTL8	Pre-mRNA-splicing factor 38B (Sarcoma antigen NY-SAR-27)	64 kDa	0.14	3	2	1	0	0	0	2	0	1	0	0	-1.290149649	0% (0.54)
ATPF2_HUMAN	A8KAL2_HUMAN		A8KAL2	cDNA FLJ76981, highly similar to Homo sapiens golgi autoantigen, golgin subfamily a, 5 (GOLGA5), mRNA	83 kDa	0.12	3	2	1	0	1	1	0	0	0	1	0	-1.290149649	0% (0.54)
Q5JWT2_HUMAN	PLXB1_HUMAN	PLXNB1	O43157	Plexin-B1 (Semaphorin receptor SEP)	232 kDa	0.026	3	2	1	1	0	0	1	1	0	0	0	-1.290149649	0% (0.54)
RTN1_HUMAN	Q53F88_HUMAN		Q53F88	General transcription factor IIE, polypeptide 1 (Alpha subunit, 56kD) variant (Fragment)	40 kDa	0.061	4	2	1	0	0	2	0	0	0	0	1	-1.290149649	0% (0.54)

NNMT_HUMAN	B7ZLX5_HUMAN	PTPRG	B7ZLX5	PTPRG protein	159 kDa	0.1	2	2	1	0	0	1	0	1	0	0	0	- 1.2901496 49	0% (0.54)
B4DG57_HUMAN (+1)	A8K2W6_HUMAN		A8K2W6	cDNA FLJ76248, highly similar to Homo sapiens phospholipase A2, group VII (platelet-activating factor acetylhydrolase, plasma) (PLA2G7), mRNA	50 kDa	0.018	3	2	1	0	2	0	0	0	0	0	1	- 1.2901496 49	0% (0.54)
CATH_HUMAN (+1)	A8MXZ4_HUMAN	GPRC5C	A8MXZ4	G-protein-coupled receptor family C group 5 member C (Fragment)	50 kDa	0.14	3	2	1	2	0	0	0	0	1	0	0	- 1.2901496 49	0% (0.54)
E9PGN7_HUMAN	CP1A1_HUMAN	CYP1A1	P04798	Cytochrome P450 1A1 (EC 1.14.14.1) (CYPIA1) (Cytochrome P450 form 6) (Cytochrome P450-C) (Cytochrome P450-P1)	58 kDa	0.099	3	2	1	0	0	1	1	1	0	0	0	- 1.2901496 49	0% (0.54)
H0Y6T6_HUMAN	G8JLK1_HUMAN	USE1	G8JLK1	Vesicle transport protein USE1	29 kDa	0.18	3	2	1	2	0	0	0	0	0	0	1	- 1.2901496 49	0% (0.54)
FA83H_HUMAN (+1)	R4GN49_HUMAN	S100A2	R4GN49	Protein S100-A2	7 kDa	0.031	3	2	1	2	0	0	0	1	0	0	0	- 1.2901496 49	0% (0.54)
C9JC84_HUMAN (+1)	MRT4_HUMAN	MRTO4	Q9UKD2	mRNA turnover protein 4 homolog	28 kDa	0.085	3	2	1	0	0	0	2	1	0	0	0	- 1.2901496 49	0% (0.54)
B3KMS0_HUMAN (+2)	B7Z3I9_HUMAN	ALAD	B7Z3I9	Delta-aminolevulinic acid dehydratase (EC 4.2.1.24)	35 kDa	0.065	3	2	1	0	0	0	2	0	1	0	0	- 1.2901496 49	0% (0.54)
TMED1_HUMAN	B3KTL0_HUMAN		B3KTL0	cDNA FLJ38437 fis, clone FEBRA2016572, highly similar to Membralin	48 kDa	0.097	3	2	1	2	0	0	0	0	0	1	0	- 1.2901496 49	0% (0.54)
B4DXJ1_HUMAN (+2)	CTBP2_HUMAN	CTBP2	P56545	C-terminal-binding protein 2 (CtBP2)	49 kDa	0.071	3	2	1	0	0	1	1	0	0	1	0	- 1.2901496 49	0% (0.54)
OSTC_HUMAN	VAMP8_HUMAN	VAMP8	Q9BV40	Vesicle-associated membrane protein 8 (VAMP-8) (Endobrevin) (EDB)	11 kDa	0.14	3	2	1	1	0	1	0	0	0	0	1	- 1.2901496 49	0% (0.54)
LTOR2_HUMAN	CAMP_HUMAN	CAMP	P49913	Cathelicidin antimicrobial peptide (18 kDa cationic antimicrobial protein) (CAP-18) (hCAP-18) [Cleaved into: Antibacterial protein FALL-39 (FALL-39 peptide antibiotic); Antibacterial protein LL-37]	19 kDa	0.29	3	2	1	1	0	0	1	0	1	0	0	- 1.2901496 49	0% (0.54)
RHOG_HUMAN	GGCT_HUMAN	GGCT	O75223	Gamma-glutamylcyclotransferase (EC 2.3.2.4) (Cytochrome c-releasing factor 21)	21 kDa	0.13	3	2	1	0	0	0	2	0	1	0	0	- 1.2901496 49	0% (0.54)
J3QRU1_HUMAN	NAT10_HUMAN	NAT10	Q9H0A0	N-acetyltransferase 10 (EC 2.3.1.-)	116 kDa	0.11	25	15	10	0	2	4	9	1	2	8	0	- 1.2901981 19	0% (0.31)
I2BPL_HUMAN	CBX5_HUMAN	CBX5	P45973	Chromobox protein homolog 5 (Antigen p25) (Heterochromatin protein 1 homolog alpha) (HP1 alpha)	22 kDa	0.055	25	15	10	0	6	1	6	4	0	4	0	- 1.2901981 19	0% (0.31)
BGH3_HUMAN (+2)	ALDR_HUMAN	AKR1B1	P15121	Aldose reductase (AR) (EC 1.1.1.21) (Aldehyde reductase) (Aldo-keto reductase family 1 member B1)	36 kDa	0.059	25	15	10	0	5	6	0	0	0	0	7	- 1.2901981 19	0% (0.31)
SRS11_HUMAN	B7Z4B8_HUMAN	HNRNPUL1	B7Z4B8	Heterogeneous nuclear ribonucleoprotein U-like protein 1 (cDNA FLJ56481, highly similar to Heterogeneous nuclear ribonucleoprotein U-like protein 1)	86 kDa	0.052	47	28	19	6	6	5	11	7	2	6	4	- 1.2902466 06	0% (0.22)
NDK3_HUMAN (+1)	GNAS1_HUMAN	GNAS	Q5JWF2	Guanine nucleotide-binding protein G(s) subunit alpha isoforms XLas (Adenylate cyclase-stimulating G alpha protein) (Extra large alphas protein) (XLalphas)	111 kDa	0.25	69	41	28	12	9	11	9	5	6	9	8	- 1.2902951 09	0% (0.17)
J3KN32_HUMAN (+1)	MFN2_HUMAN	MFN2	O95140	Mitofusin-2 (EC 3.6.5.-) (Transmembrane GTPase MFN2)	86 kDa	0.091	30	18	12	10	3	2	3	1	2	3	6	- 1.2977010 73	0% (0.28)
PLRKT_HUMAN	FINC_HUMAN	FN1	P02751	Fibronectin (FN) (Cold-insoluble globulin) (CIG)	263 kDa	0.21	138	82	56	62	0	0	0	0	12	15	26	- 1.2991248 33	0% (0.074)

				[Cleaved into: Anastellin; Ugl-Y1; Ugl-Y2; Ugl-Y3]															
G45IP_HUMAN	A2AB90_HUMAN	TAPBP	A2AB90	Tapasin	49 kDa	0.16	35	21	14	1	7	5	9	0	2	7	5	-1.303239476	0% (0.25)
RMND1_HUMAN	CATA_HUMAN	CAT	P04040	Catalase (EC 1.11.1.6)	60 kDa	0.071	67	40	27	9	8	11	13	3	9	6	9	-1.304350701	0% (0.16)
PLBL2_HUMAN	F8W930_HUMAN	IGF2BP2	F8W930	Insulin-like growth factor 2 mRNA-binding protein 2	67 kDa	0.1	135	81	55	6	27	31	19	32	5	15	2	-1.306339864	0% (0.071)
B3KRC6_HUMAN (+1)	A8K883_HUMAN		A8K883	cDNA FLJ77590, highly similar to Homo sapiens leucine rich repeat containing 1, mRNA	59 kDa	0.11	40	24	16	11	2	5	6	6	0	6	2	-1.307496651	0% (0.23)
H0Y8R1_HUMAN	ARF5_HUMAN	ARF5	P84085	ADP-ribosylation factor 5	21 kDa	0.046	40	24	16	0	0	0	10	0	4	0	0	-1.307496651	0% (0.23)
B4DDF4_HUMAN (+3)	ERAP1_HUMAN	ERAP1	Q9NZ08	Endoplasmic reticulum aminopeptidase 1 (EC 3.4.11.-) (ARTS-1) (Adipocyte-derived leucine aminopeptidase) (A-LAP) (Aminopeptidase PILS) (Puromycin-insensitive leucyl-specific aminopeptidase) (PILS-AP) (Type 1 tumor necrosis factor receptor shedding aminopeptidase regulator)	107 kDa	0.11	119	71	48	17	16	29	8	3	4	21	20	-1.310576351	0% (0.083)
A8K894_HUMAN (+4)	Q5VU59_HUMAN	TPM3	Q5VU59	Uncharacterized protein	27 kDa	0.089	83	49	33	4	12	17	16	6	7	12	8	-1.310620972	0% (0.13)
B4DZA5_HUMAN	FAHD1_HUMAN	FAHD1	Q6P587	Acylpyruvase FAHD1, mitochondrial (EC 3.7.1.5) (Fumarylacetoacetate hydrolase domain-containing protein 1) (YisK-like protein)	25 kDa	0.033	46	27	18	5	7	4	11	4	3	4	7	-1.310871942	0% (0.21)
PCP_HUMAN	DDRGK_HUMAN	DDRGK1	Q96HY6	DDRGK domain-containing protein 1	36 kDa	0.071	45	27	18	6	4	4	13	5	1	5	7	-1.310871942	0% (0.21)
TXD17_HUMAN	A4QN18_HUMAN	SEC16A	A4QN18	SEC16A protein (Fragment)	211 kDa	0.38	8	5	3	2	1	1	1	1	1	1	0	-1.313513614	0% (0.43)
H1X_HUMAN	SNTB2_HUMAN	SNTB2	Q13425	Beta-2-syntrophin (59 kDa dystrophin-associated protein A1 basic component 2) (Syntrophin-3) (SNT3) (Syntrophin-like) (SNTL)	58 kDa	0.23	8	5	3	1	3	1	0	1	0	1	1	-1.313513614	0% (0.43)
MAAI_HUMAN	ACS2L_HUMAN	ACSS1	Q9NUB1	Acetyl-coenzyme A synthetase 2-like, mitochondrial (EC 6.2.1.1) (Acetate--CoA ligase 2) (Acetyl-CoA synthetase 2) (AceCS2) (Acyl-CoA synthetase short-chain family member 1)	75 kDa	0.13	7	5	3	0	2	1	1	3	0	0	0	-1.313513614	0% (0.43)
GSTM2_HUMAN (+1)	SCFD2_HUMAN	SCFD2	Q8WU76	Sec1 family domain-containing protein 2 (Syntaxin-binding protein 1-like 1)	75 kDa	0.21	8	5	3	0	1	1	3	2	1	0	0	-1.313513614	0% (0.43)
FIBB_HUMAN (+1)	ELMD2_HUMAN	ELMOD2	Q8IZ81	ELMO domain-containing protein 2	35 kDa	0.18	8	5	3	4	0	0	1	1	1	1	0	-1.313513614	0% (0.43)
RRP1B_HUMAN	E7EWV1_HUMAN	PIGG	E7EWV1	GPI ethanolamine phosphate transferase 2	99 kDa	0.028	8	5	3	4	1	0	0	1	0	2	0	-1.313513614	0% (0.43)
A8K5X9_HUMAN (+1)	A8K5L5_HUMAN		A8K5L5	cDNA FLJ75689, highly similar to Homo sapiens poliovirus receptor-related 2 (herpesvirus entry mediator B) (PVRL2), mRNA	51 kDa	0.026	8	5	3	0	3	1	1	0	0	2	1	-1.313513614	0% (0.43)
A8K9U6_HUMAN (+1)	B2R7V4_HUMAN		B2R7V4	cDNA, FLJ93619, highly similar to Homo sapiens PRP4 pre-mRNA processing factor 4 homolog (yeast) (PRPF4), mRNA	58 kDa	0.052	8	5	3	0	2	0	3	0	1	2	0	-1.313513614	0% (0.43)
DHSO_HUMAN	AG10A_HUMAN	ALG10	Q5BKT4	Dol-P-Glc:Glc(2)Man(9)GlcNAc(2)-PP-Dol alpha-1,2-glucosyltransferase (EC	56 kDa	0.13	9	5	3	1	1	1	2	1	0	1	1	-1.313513614	0% (0.43)

				2.4.1.256) (Alpha-1,2-glucosyltransferase ALG10-A) (Alpha-2-glucosyltransferase ALG10-A) (Asparagine-linked glycosylation protein 10 homolog A)															
DHRS1_HUMAN	CAH2_HUMAN	CA2	P00918	Carbonic anhydrase 2 (EC 4.2.1.1) (Carbonate dehydratase II) (Carbonic anhydrase C) (CAC) (Carbonic anhydrase II) (CA-II)	29 kDa	0.13	8	5	3	2	1	0	2	0	0	0	3	-1.313513614	0% (0.43)
F6M9T7_HUMAN (+1)	A8K321_HUMAN		A8K321	cDNA FLJ78524, highly similar to Homo sapiens SMILE protein (SMILE), mRNA	102 kDa	0.11	8	5	3	5	0	0	0	0	0	1	2	-1.313513614	0% (0.43)
COIL_HUMAN	SCRN1_HUMAN	SCRN1	Q12765	Secernin-1	46 kDa	0.049	8	5	3	0	2	0	3	0	3	0	0	-1.313513614	0% (0.43)
DTX3L_HUMAN	B3KM57_HUMAN		B3KM57	cDNA FLJ10347 fis, clone NT2RM2001035, highly similar to CCR4-NOT transcription complex subunit 7	33 kDa	0.043	8	5	3	1	2	1	1	1	0	1	1	-1.313513614	0% (0.43)
NUD19_HUMAN	B4DYY5_HUMAN		B4DYY5	cDNA FLJ55671, highly similar to Ubiquitin-associated protein 2-like	114 kDa	0.18	8	5	3	1	2	1	1	1	1	0	1	-1.313513614	0% (0.43)
RFIP1_HUMAN	DNLZ_HUMAN	DNLZ	Q5SXM8	DNL-type zinc finger protein (Hsp70-escort protein 1) (HEP1) (mtHsp70-escort protein)	19 kDa	0.018	7	5	3	0	2	2	1	1	0	1	1	-1.313513614	0% (0.43)
TEX2_HUMAN	TBK1_HUMAN	TBK1	Q9UHD2	Serine/threonine-protein kinase TBK1 (EC 2.7.11.1) (NF-kappa-B-activating kinase) (T2K) (TANK-binding kinase 1)	84 kDa	0.024	8	5	3	0	3	2	0	1	1	1	0	-1.313513614	0% (0.43)
EI2BE_HUMAN	B4DNC0_HUMAN		B4DNC0	cDNA FLJ61141, highly similar to Ras-related protein Rab-34	31 kDa	0.031	8	5	3	3	0	0	2	0	2	0	1	-1.313513614	0% (0.43)
PSB8_HUMAN (+1)	Q5U0F7_HUMAN		Q5U0F7	Phosphatidic acid phosphatase type 2B	35 kDa	0.13	8	5	3	0	3	2	0	0	3	0	0	-1.313513614	0% (0.43)
SRP72_HUMAN	C9IZG4_HUMAN	CUTA	C9IZG4	Protein CutA	14 kDa	0.11	8	5	3	0	1	1	3	1	0	1	1	-1.313513614	0% (0.43)
A2A2G4_HUMAN (+1)	F8W9Y0_HUMAN	STX3	F8W9Y0	Syntaxin-3	30 kDa	0.049	8	5	3	3	0	1	1	0	0	1	2	-1.313513614	0% (0.43)
CLP1L_HUMAN (+1)	B5MCX3_HUMAN	37500	B5MCX3	Septin-2	37 kDa	0.084	50	30	20	1	11	10	8	3	8	3	6	-1.313614352	0% (0.20)
A8K337_HUMAN (+1)	Q53GW1_HUMAN		Q53GW1	Vesicle transport-related protein isoform a variant (Fragment)	72 kDa	0.088	92	55	37	18	15	17	6	16	6	8	7	-1.313715154	0% (0.11)
DUT_HUMAN (+1)	MCU_HUMAN	MCU	Q8NE86	Calcium uniporter protein, mitochondrial (Coiled-coil domain-containing protein 109A)	40 kDa	0.16	97	58	39	26	8	10	14	8	13	9	8	-1.315033579	0% (0.10)
ADRO_HUMAN (+2)	D3DUZ3_HUMAN	IFI16	D3DUZ3	Interferon, gamma-inducible protein 16, isoform CRA_a (Interferon, gamma-inducible protein 16, isoform CRA_b)	82 kDa	0.14	58	36	24	2	6	8	20	3	5	12	4	-1.317802078	0% (0.17)
B7Z747_HUMAN	Q53G61_HUMAN		Q53G61	Small nuclear ribonucleoprotein polypeptide A' variant (Fragment)	28 kDa	0.22	65	39	26	7	9	10	13	6	5	6	9	-1.319437795	0% (0.15)
S10AG_HUMAN	B4E1L5_HUMAN		B4E1L5	cDNA FLJ51601, highly similar to Interferon-induced guanylate-binding protein 1	64 kDa	0.22	12	8	5	0	0	0	8	2	0	2	1	-1.321933024	0% (0.37)
MGST2_HUMAN	B2R858_HUMAN		B2R858	cDNA, FLJ93750, Homo sapiens DEAD (Asp-Glu-Ala-Asp) box polypeptide 6 (DDX6), mRNA	53 kDa	0.18	13	8	5	1	2	3	2	1	2	1	1	-1.321933024	0% (0.37)
Q96B24_HUMAN	A8KA56_HUMAN		A8KA56	cDNA FLJ77678, highly similar to Homo sapiens DEAD (Asp-Glu-Ala-Asp) box polypeptide 23, mRNA	96 kDa	0.17	13	8	5	1	3	2	2	0	1	4	0	-1.321933024	0% (0.37)
NOL9_HUMAN	A8K3Q3_HUMAN		A8K3Q3	cDNA FLJ78230	48 kDa	0.06	13	8	5	1	4	1	2	0	2	2	1	-1.321933024	0% (0.37)

ARL2_HUMAN	5NT3A_HUMAN	NT5C3A	Q9H0P0	Cytosolic 5'-nucleotidase 3A (EC 3.1.3.5) (Cytosolic 5'-nucleotidase 3) (Cytosolic 5'-nucleotidase III) (cN-III) (Pyrimidine 5'-nucleotidase 1) (P5'N-1) (P5N-1) (PN-I) (Uridine 5'-monophosphate hydrolase 1) (p36)	38 kDa	0.22	13	8	5	1	0	0	7	0	4	1	0	-1.321933024	0% (0.37)
B2RAN1_HUMAN (+4)	SE1L1_HUMAN	SEL1L	Q9UBV2	Protein sel-1 homolog 1 (Suppressor of lin-12-like protein 1) (Sel-1L)	89 kDa	0.087	13	8	5	3	2	2	1	1	1	2	1	-1.321933024	0% (0.37)
ACDSB_HUMAN (+1)	L0R6Q1_HUMAN	SLC35A4	L0R6Q1	Alternative protein SLC35A4	11 kDa	0.076	14	8	5	2	1	4	1	2	1	2	1	-1.321933024	0% (0.37)
A4D198_HUMAN	A4D198_HUMAN	LOC392647	A4D198	Similar to mKIAA0038 protein	33 kDa	0.092	13	8	5	0	5	3	0	2	1	2	0	-1.321933024	0% (0.37)
K2013_HUMAN	A8K644_HUMAN	SFRS4	A8K644	Splicing factor, arginine/serine-rich 4, isoform CRA_b (cDNA FLJ76859, highly similar to Homo sapiens splicing factor, arginine/serine-rich 4 (SFRS4), mRNA)	57 kDa	0.047	13	8	5	0	3	4	0	0	0	3	0	-1.321933024	0% (0.37)
LARP7_HUMAN	B2R888_HUMAN		B2R888	Monocyte differentiation antigen CD14 (Myeloid cell-specific leucine-rich glycoprotein)	40 kDa	0.057	147	88	59	17	24	28	19	0	22	22	15	-1.323451605	0% (0.054)
RT05_HUMAN	RAB2A_HUMAN	RAB2A	P61019	Ras-related protein Rab-2A	24 kDa	0.1	212	128	86	35	23	33	36	19	22	26	19	-1.323660653	95% (0.025)
KPRA_HUMAN	B4DSE2_HUMAN		B4DSE2	cDNA FLJ57277, highly similar to Tripeptidyl-peptidase 1 (EC 3.4.14.9)	42 kDa	0.07	85	51	34	6	15	19	11	1	2	15	16	-1.324139413	0% (0.12)
B9EIS5_HUMAN (+3)	STRN_HUMAN	STRN	O43815	Striatin	86 kDa	0.01	18	11	7	2	3	3	3	3	2	0	2	-1.326276647	0% (0.32)
RBM10_HUMAN	B4DJV9_HUMAN		B4DJV9	cDNA FLJ60607, highly similar to Acyl-protein thioesterase 1 (EC 3.1.2.-)	28 kDa	0.031	18	11	7	1	3	2	5	2	2	2	1	-1.326276647	0% (0.32)
PARN_HUMAN	ARF6_HUMAN	ARF6	P62330	ADP-ribosylation factor 6	20 kDa	0.039	18	11	7	0	2	2	7	1	4	2	0	-1.326276647	0% (0.32)
E7EVA0_HUMAN	SSRP1_HUMAN	SSRP1	Q08945	FACT complex subunit SSRP1 (Chromatin-specific transcription elongation factor 80 kDa subunit) (Facilitates chromatin transcription complex 80 kDa subunit) (FACT 80 kDa subunit) (FACTp80) (Facilitates chromatin transcription complex subunit SSRP1) (Recombination signal sequence recognition protein 1) (Structure-specific recognition protein 1) (hSSRP1) (T160)	81 kDa	0.017	102	60	40	12	16	14	18	24	4	6	6	-1.326482211	0% (0.094)
A8K2S7_HUMAN (+1)	B2R5M8_HUMAN		B2R5M8	Isocitrate dehydrogenase [NADP] (EC 1.1.1.42)	47 kDa	0.12	100	60	40	9	17	17	19	3	20	10	7	-1.326482211	0% (0.094)
WDR3_HUMAN	PRDX4_HUMAN	PRDX4	Q13162	Peroxiredoxin-4 (EC 1.11.1.15) (Antioxidant enzyme AOE372) (AOE37-2) (Peroxiredoxin IV) (Prx-IV) (Thioredoxin peroxidase AO372) (Thioredoxin-dependent peroxide reductase A0372)	31 kDa	0.04	100	60	40	11	20	16	12	4	13	10	13	-1.326482211	0% (0.094)
UFSP2_HUMAN	RTN4_HUMAN	RTN4	Q9NQC3	Reticulon-4 (Foocen) (Neurite outgrowth inhibitor) (Nogo protein) (Neuroendocrine-specific protein) (NSP) (Neuroendocrine-specific protein C homolog) (RTN-x) (Reticulon-5)	130 kDa	0.12	115	69	46	39	10	12	10	6	16	7	17	-1.328240013	0% (0.076)
CD2A1_HUMAN (+1)	COX6C_HUMAN	COX6C	P09669	Cytochrome c oxidase subunit 6C (Cytochrome c oxidase polypeptide VIc)	9 kDa	0.26	23	14	9	2	2	2	8	4	4	2	1	-1.328930311	0% (0.29)

H11_HUMAN	E7EVQ6_HUMAN	SQLE	E7EVQ6	Squalene monooxygenase	53 kDa	0.21	23	14	9	0	6	5	3	6	1	2	0	-1.328930311	0% (0.29)
DECR2_HUMAN (+1)	B4DLN7_HUMAN		B4DLN7	cDNA FLJ59248, highly similar to Homo sapiens cytochrome P450, family 20, subfamily A, polypeptide 1, transcript variant 1, mRNA	53 kDa	0.11	23	14	9	5	5	1	3	0	3	2	4	-1.328930311	0% (0.29)
B3KXX5_HUMAN (+2)	A8K6H9_HUMAN		A8K6H9	cDNA FLJ75876, highly similar to Homo sapiens solute carrier family 38, member 2 (SLC38A2), mRNA	56 kDa	0.021	23	14	9	4	5	5	0	6	0	3	0	-1.328930311	0% (0.29)
B7ZKM8_HUMAN (+1)	TOP1_HUMAN	TOP1	P11387	DNA topoisomerase 1 (EC 5.99.1.2) (DNA topoisomerase I)	91 kDa	0.03	27	17	11	2	4	5	6	4	4	1	2	-1.330721751	0% (0.26)
TM245_HUMAN	RM13_HUMAN	MRPL13	Q9BYD1	39S ribosomal protein L13, mitochondrial (L13mt) (MRP-L13)	21 kDa	0.04	28	17	11	5	3	4	5	2	1	4	4	-1.330721751	0% (0.26)
B7WP27_HUMAN (+1)	J3QLR8_HUMAN	MRPS23	J3QLR8	28S ribosomal protein S23, mitochondrial	18 kDa	0.026	28	17	11	7	3	3	4	5	0	2	4	-1.330721751	0% (0.26)
SRP14_HUMAN	HXK3_HUMAN	HK3	P52790	Hexokinase-3 (EC 2.7.1.1) (Hexokinase type III) (HK III)	99 kDa	0.27	28	17	11	0	4	10	2	0	3	3	5	-1.330721751	0% (0.26)
B4DLE7_HUMAN (+2)	MA2A1_HUMAN	MAN2A1	Q16706	Alpha-mannosidase 2 (EC 3.2.1.114) (Golgi alpha-mannosidase II) (AMan II) (Man II) (Mannosidase alpha class 2A member 1) (Mannosyl-oligosaccharide 1,3-1,6-alpha-mannosidase)	131 kDa	0.097	33	20	13	6	7	4	3	0	3	5	5	-1.332014008	0% (0.24)
B3KY30_HUMAN (+3)	MOT4_HUMAN	SLC16A3	O15427	Monocarboxylate transporter 4 (MCT 4) (Solute carrier family 16 member 3)	49 kDa	0.14	33	20	13	3	8	5	4	2	2	3	6	-1.332014008	0% (0.24)
Q59G24_HUMAN (+1)	B7Z5Z2_HUMAN	RRAS2	B7Z5Z2	Ras-related protein R-Ras2 (cDNA FLJ61162, highly similar to Ras-related protein R-Ras2)	24 kDa	0.25	33	20	13	11	4	4	0	2	0	6	4	-1.332014008	0% (0.24)
B5BU32_HUMAN (+5)	RL36_HUMAN	RPL36	Q9Y3U8	60S ribosomal protein L36	12 kDa	0.24	38	23	15	6	5	3	9	4	4	3	4	-1.332991398	0% (0.22)
ECHD1_HUMAN	CNPY2_HUMAN	CNPY2	Q9Y2B0	Protein canopy homolog 2 (MIR-interacting saposin-like protein) (Putative secreted protein Zsig9) (Transmembrane protein 4)	21 kDa	0.098	63	38	25	6	7	14	11	4	10	6	5	-1.335674277	0% (0.15)
B7ZLZ2_HUMAN (+1)	B4DW81_HUMAN		B4DW81	cDNA FLJ58863, highly similar to Protein NipSnap3A	26 kDa	0.06	67	41	27	5	13	14	9	10	6	3	9	-1.335988497	0% (0.14)
VPS16_HUMAN	H2A2B_HUMAN	HIST2H2AB	Q8IUE6	Histone H2A type 2-B	14 kDa	0.052	136	82	54	14	0	24	28	20	0	7	17	-1.346190735	0% (0.050)
B4DQW0_HUMAN (+1)	DRA_HUMAN	HLA-DRA	P01903	HLA class II histocompatibility antigen, DR alpha chain (MHC class II antigen DRA)	29 kDa	0.15	111	67	44	13	20	17	17	8	7	11	18	-1.347464861	0% (0.070)
CBX5_HUMAN	B4DRS6_HUMAN		B4DRS6	cDNA FLJ58980, highly similar to Sideroflexin-3	36 kDa	0.36	82	49	32	16	8	9	16	1	8	6	17	-1.350057572	0% (0.10)
B7Z5X7_HUMAN	CLIC1_HUMAN	CLIC1	O00299	Chloride intracellular channel protein 1 (Chloride channel ABP) (Nuclear chloride ion channel 27) (NCC27) (Regulatory nuclear chloride ion channel protein) (hRNCC)	27 kDa	0.12	72	43	28	6	11	8	18	4	11	9	4	-1.351408911	0% (0.12)
RUFY1_HUMAN	VATB2_HUMAN	ATP6V1B2	P21281	V-type proton ATPase subunit B, brain isoform (V-ATPase subunit B 2) (Endomembrane proton pump 58 kDa subunit) (HO57) (Vacuolar proton pump subunit B 2)	57 kDa	0.031	164	99	65	19	29	30	22	9	11	24	21	-1.352008198	95% (0.033)
A8MQB8_HUMAN	A8K3M9_HUMAN		A8K3M9	cDNA FLJ76387, highly similar to Homo sapiens splicing factor, arginine/serine-rich 9 (SFRS9), mRNA	26 kDa	0.07	47	28	18	5	5	6	11	6	2	5	5	-1.357291933	0% (0.18)
GCP60_HUMAN	B4DLV7_HUMAN		B4DLV7	cDNA FLJ60299, highly similar to Rab GDP dissociation inhibitor beta	51 kDa	0.045	104	63	41	12	17	18	16	13	6	13	10	-1.358551826	0% (0.071)

68MP_HUMAN (+1)	GNAI3_HUMAN	GNAI3	P08754	Guanine nucleotide-binding protein G(k) subunit alpha (G(i) alpha-3)	41 kDa	0.33	103	63	41	18	16	17	12	9	7	12	12	- 1.3585518 26	0% (0.071)
ACS2L_HUMA N (+1)	ZNT7_HUMAN	SLC30A7	Q8NEW 0	Zinc transporter 7 (ZnT-7) (Solute carrier family 30 member 7) (Znt-like transporter 2)	42 kDa	0.073	41	25	16	10	5	3	7	7	4	3	2	- 1.3592961 66	0% (0.19)
MCTS1_HUMA N	ITPR2_HUMAN	ITPR2	Q14571	Inositol 1,4,5-trisphosphate receptor type 2 (IP3 receptor isoform 2) (IP3R 2) (InsP3R2) (Type 2 inositol 1,4,5-trisphosphate receptor) (Type 2 InsP3 receptor)	308 kDa	0.2	41	25	16	12	6	6	1	1	2	6	7	- 1.3592961 66	0% (0.19)
OGT1_HUMAN	C9JV86_HUMA N	CD63	C9JV86	CD63 antigen	25 kDa	0.046	36	22	14	6	7	8	1	2	5	3	4	- 1.3618295 38	0% (0.21)
C9JZ87_HUMA N (+2)	BASI_HUMAN	BSG	P35613	Basigin (5F7) (Collagenase stimulatory factor) (Extracellular matrix metalloproteinase inducer) (EMMPRIN) (Leukocyte activation antigen M6) (OK blood group antigen) (Tumor cell-derived collagenase stimulatory factor) (TCSF) (CD antigen CD147)	42 kDa	0.28	36	22	14	5	7	8	2	4	3	5	2	- 1.3618295 38	0% (0.21)
D6RGZ2_HUMA N (+2)	RS26_HUMAN	RPS26	P62854	40S ribosomal protein S26	13 kDa	0.18	36	22	14	6	5	4	7	5	5	2	3	- 1.3618295 38	0% (0.21)
UBP10_HUMAN	DESP_HUMAN	DSP	P15924	Desmoplakin (DP) (250/210 kDa paraneoplastic pemphigus antigen)	332 kDa	0.049	176	106	69	26	29	28	24	25	17	14	17	- 1.3640990 16	95% (0.025)
5NTC_HUMAN (+2)	A8YXX5_HUM AN	PIG60	A8YXX5	Cell proliferation-inducing protein 60 (cDNA FLJ12721 fis, clone NT2RP1001310, highly similar to Mitochondrial carrier homolog 1)	38 kDa	0.075	31	19	12	7	4	3	5	5	2	3	2	- 1.3651316 59	0% (0.23)
PNKD_HUMAN	F8RZC1_HUMA N	ND4	F8RZC1	NADH-ubiquinone oxidoreductase chain 4 (EC 1.6.5.3)	52 kDa	0.11	31	19	12	8	4	3	4	5	2	3	2	- 1.3651316 59	0% (0.23)
A8K5C2_HUMA N (+1)	H0YF29_HUMA N	C8orf82	H0YF29	UPF0598 protein C8orf82 (Fragment)	29 kDa	0.083	31	19	12	3	6	4	6	4	3	2	3	- 1.3651316 59	0% (0.23)
RDH11_HUMA N	B2R5B3_HUMA N		B2R5B3	Histone H2A	14 kDa	0.17	553	334	218	66	71	91	107	78	47	34	61	- 1.3673105 01	95% (0.0001 9)
B7ZKT9_HUMA N	B4DTE8_HUMA N	PLSCR1	B4DTE8	Phospholipid scramblase 1 (cDNA FLJ57588, highly similar to Phospholipid scramblase 1)	27 kDa	0.055	27	16	10	7	5	3	3	5	1	2	3	- 1.3696125 27	0% (0.25)
CB054_HUMAN	VIGLN_HUMA N	HDLBP	Q00341	Vigilin (High density lipoprotein-binding protein) (HDL-binding protein)	141 kDa	0.11	26	16	10	0	5	11	1	0	1	9	0	- 1.3696125 27	0% (0.25)
MYO5C_HUMA N	ADA10_HUMA N	ADAM10	O14672	Disintegrin and metalloproteinase domain-containing protein 10 (ADAM 10) (EC 3.4.24.81) (CDw156) (Kuzbanian protein homolog) (Mammalian disintegrin-metalloprotease) (CD antigen CD156c)	84 kDa	0.038	26	16	10	8	3	4	1	3	0	4	3	- 1.3696125 27	0% (0.25)
PXMP2_HUMA N	PSMD8_HUMA N	PSMD8	P48556	26S proteasome non-ATPase regulatory subunit 8 (26S proteasome regulatory subunit RPN12) (26S proteasome regulatory subunit S14) (p31)	40 kDa	0.14	26	16	10	2	3	3	8	2	5	2	1	- 1.3696125 27	0% (0.25)
TPPC3_HUMAN	MYO1B_HUMA N	MYO1B	O43795	Unconventional myosin-Ib (MYH-1c) (Myosin I alpha) (MMI-alpha) (MMIa)	132 kDa	0.12	25	16	10	0	6	9	0	0	0	6	4	- 1.3696125 27	0% (0.25)
NELFB_HUMA N	A8K2Y2_HUMA N		A8K2Y2	cDNA FLJ78120, highly similar to Homo sapiens eukaryotic translation initiation factor 2, subunit 3 gamma, 52kDa (EIF2S3), mRNA	51 kDa	0.043	26	16	10	2	4	6	4	2	8	0	0	- 1.3696125 27	0% (0.25)
B7Z1C5_HUMA N (+1)	A8K0F7_HUMA N		A8K0F7	cDNA FLJ76587, highly similar to Homo sapiens vitamin K epoxide reductase	20 kDa	0.074	26	16	10	5	4	3	4	2	3	2	3	- 1.3696125 27	0% (0.25)

				complex, subunit 1-like 1 (VKORC1L1), mRNA														
B1AJY5_HUMAN (+2)	RM39_HUMAN	MRPL39	Q9NYK5	39S ribosomal protein L39, mitochondrial (L39mt) (MRP-L39) (39S ribosomal protein L5, mitochondrial) (L5mt) (MRP-L5)	39 kDa	0.097	25	16	10	7	5	4	0	4	1	3	2	-1.369612527	0% (0.25)
B4DMJ9_HUMAN	Q9BWC7_HUMAN	COMT	Q9BWC7	COMT protein	20 kDa	0.18	58	36	23	11	9	6	10	6	6	6	5	-1.372164177	0% (0.13)
H0UI80_HUMAN (+1)	B2R6N9_HUMAN		B2R6N9	cDNA, FLJ93042, highly similar to Homo sapiens signal sequence receptor, alpha (translocon-associated protein alpha) (SSR1), mRNA	32 kDa	0.083	59	36	23	10	9	5	12	7	6	4	6	-1.372164177	0% (0.13)
KPCD_HUMAN	F6IQV5_HUMAN	HLA-A	F6IQV5	MHC class I antigen (Fragment)	38 kDa	0.065	87	53	34	0	0	12	22	0	0	0	13	-1.374859527	0% (0.082)
PSA2_HUMAN	M4QNU4_HUMAN	HLA-A	M4QNU4	MHC class I antigen (Fragment)	32 kDa	0.18	87	53	34	0	0	12	22	3	0	0	0	-1.374859527	0% (0.082)
B3GA3_HUMAN (+1)	H0Y368_HUMAN	DPM1	H0Y368	Dolichol-phosphate mannosyltransferase (Fragment)	33 kDa	0.099	54	33	21	13	5	6	9	5	5	4	7	-1.375047988	0% (0.14)
Q32Q10_HUMAN (+1)	ISG15_HUMAN	ISG15	P05161	Ubiquitin-like protein ISG15 (Interferon-induced 15 kDa protein) (Interferon-induced 17 kDa protein) (IP17) (Ubiquitin cross-reactive protein) (hUCRP)	18 kDa	0.093	20	13	8	0	0	0	13	3	4	2	0	-1.376036734	0% (0.27)
MBD2_HUMAN	H3BV80_HUMAN	RNPS1	H3BV80	RNA-binding protein with serine-rich domain 1	25 kDa	0.068	21	13	8	4	4	2	3	2	2	1	3	-1.376036734	0% (0.27)
SCFD2_HUMAN	SURF1_HUMAN	SURF1	Q15526	Surfeit locus protein 1	33 kDa	0.045	21	13	8	4	2	1	6	2	1	2	3	-1.376036734	0% (0.27)
B7Z5Z2_HUMAN (+1)	CPT2_HUMAN	CPT2	P23786	Carnitine O-palmitoyltransferase 2, mitochondrial (EC 2.3.1.21) (Carnitine palmitoyltransferase II) (CPT II)	74 kDa	0.26	81	50	32	13	19	10	9	12	4	7	8	-1.376942092	0% (0.088)
COQ9_HUMAN	SAC1_HUMAN	SACM1L	Q9NTJ5	Phosphatidylinositide phosphatase SAC1 (EC 3.1.3.-) (Suppressor of actin mutations 1-like protein)	67 kDa	0.14	51	30	19	19	3	6	2	2	5	5	6	-1.378504031	0% (0.15)
NPA1P_HUMAN	VAT1_HUMAN	VAT1	Q99536	Synaptic vesicle membrane protein VAT-1 homolog (EC 1.-.-.-)	42 kDa	0.012	77	47	30	16	10	15	6	5	7	5	13	-1.379292905	0% (0.094)
TF3C4_HUMAN	M0R210_HUMAN	RPS16	M0R210	40S ribosomal protein S16 (Ribosomal protein S16, isoform CRA_a)	14 kDa	0.032	76	47	30	10	12	12	13	13	10	3	4	-1.379292905	0% (0.094)
VPS18_HUMAN	ERO1A_HUMAN	ERO1L	Q96HE7	ERO1-like protein alpha (ERO1-L) (ERO1-L-alpha) (EC 1.8.4.-) (Endoplasmic oxidoreductin-1-like protein) (Oxidoreductin-1-L-alpha)	54 kDa	0.033	182	112	72	29	35	31	17	6	29	13	25	-1.381466221	95% (0.018)
A8K8J9_HUMAN	ARPC2_HUMAN	ARPC2	O15144	Actin-related protein 2/3 complex subunit 2 (Arp2/3 complex 34 kDa subunit) (p34-ARC)	34 kDa	0.11	67	41	26	8	9	13	12	8	10	3	5	-1.385035529	0% (0.11)
EXOSX_HUMAN	B3KU62_HUMAN		B3KU62	cDNA FLJ39243 fis, clone OCBBF2008283, highly similar to Protein NDRG1	39 kDa	0.035	67	41	26	5	11	13	12	9	2	12	3	-1.385035529	0% (0.11)
C9JTZ6_HUMAN (+2)	F5H1S9_HUMAN	PUS1	F5H1S9	tRNA pseudouridine synthase (EC 5.4.99.-)	42 kDa	0.075	16	10	6	2	3	3	2	2	0	1	3	-1.386012559	0% (0.30)
TM192_HUMAN	ACSF3_HUMAN	ACSF3	Q4G176	Acyl-CoA synthetase family member 3, mitochondrial (EC 6.2.1.-)	64 kDa	0.15	16	10	6	5	2	2	1	2	0	0	4	-1.386012559	0% (0.30)
D3DNQ1_HUMAN (+1)	IFIT3_HUMAN	IFIT3	O14879	Interferon-induced protein with tetratricopeptide repeats 3 (IFIT-3) (CIG49) (ISG-60) (Interferon-induced 60 kDa protein) (IFI-60K) (Interferon-induced protein with tetratricopeptide repeats 4) (IFIT-4) (Retinoic acid-induced gene G protein) (P60) (RIG-G)	56 kDa	0.075	16	10	6	0	0	0	10	0	6	0	0	-1.386012559	0% (0.30)

EI2BA_HUMAN	H0YKW5_HUMAN	C15orf48	H0YKW5	Normal mucosa of esophagus-specific gene 1 protein (Fragment)	15 kDa	0.13	40	24	15	9	8	4	5	0	4	5	6	-1.387977953	0% (0.18)
PSA1_HUMAN	B4DM33_HUMAN		B4DM33	cDNA FLJ52068, highly similar to Microtubule-associated protein RP/EB family member 1	27 kDa	0.13	40	24	15	0	11	10	3	6	2	4	4	-1.387977953	0% (0.18)
B3KRM8_HUMAN (+4)	H4_HUMAN	HIST1H4A	P62805	Histone H4	11 kDa	0.083	786	484	311	107	101	107	175	110	72	60	70	-1.39046238	95% (< 0.00010)
B4DZP4_HUMAN (+1)	AP2A2_HUMAN	AP2A2	O94973	AP-2 complex subunit alpha-2 (100 kDa coated vesicle protein C) (Adapter-related protein complex 2 subunit alpha-2) (Adaptor protein complex AP-2 subunit alpha-2) (Alpha-adaptin C) (Alpha2-adaptin) (Clathrin assembly protein complex 2 alpha-C large chain) (Huntingtin yeast partner J) (Huntingtin-interacting protein 9) (HIP-9) (Huntingtin-interacting protein J) (Plasma membrane adaptor HA2/AP2 adaptin alpha C subunit)	104 kDa	0.14	104	63	40	14	20	15	13	6	11	13	9	-1.391506443	0% (0.057)
CAYP1_HUMAN (+3)	BLVRB_HUMAN	BLVRB	P30043	Flavin reductase (NADPH) (FR) (EC 1.5.1.30) (Biliverdin reductase B) (BVR-B) (EC 1.3.1.24) (Biliverdin-IX beta-reductase) (Green heme-binding protein) (GHBP) (NADPH-dependent diaphorase) (NADPH-flavin reductase) (FLR)	22 kDa	0.32	34	21	13	0	4	4	13	1	6	4	2	-1.394714944	0% (0.20)
HIP1_HUMAN	A4_HUMAN	APP	P05067	Amyloid beta A4 protein (ABPP) (APPI) (APP) (Alzheimer disease amyloid protein) (Cerebral vascular amyloid peptide) (CVAP) (PreA4) (Protease nexin-II) (PN-II) [Cleaved into: N-APP; Soluble APP-alpha (S-APP-alpha); Soluble APP-beta (S-APP-beta); C99; Beta-amyloid protein 42 (Beta-APP42); Beta-amyloid protein 40 (Beta-APP40); C83; P3(42); P3(40); C80; Gamma-secretase C-terminal fragment 59 (Amyloid intracellular domain 59) (AICD-59) (AID(59)) (Gamma-CTF(59)); Gamma-secretase C-terminal fragment 57 (Amyloid intracellular domain 57) (AICD-57) (AID(57)) (Gamma-CTF(57)); Gamma-secretase C-terminal fragment 50 (Amyloid intracellular domain 50) (AICD-50) (AID(50)) (Gamma-CTF(50)); C31]	87 kDa	0.039	34	21	13	7	8	6	0	4	0	6	3	-1.394714944	0% (0.20)
E9PSH2_HUMAN	A8K4Q9_HUMAN		A8K4Q9	cDNA FLJ77931	103 kDa	0.11	34	21	13	12	3	3	0	0	4	4	5	-1.394714944	0% (0.20)
UBE2O_HUMAN	D3DPU2_HUMAN	CAP1	D3DPU2	Adenylyl cyclase-associated protein	52 kDa	0.029	79	46	29	10	11	16	8	9	6	8	6	-1.395360121	0% (0.089)
B3KUH3_HUMAN (+2)	B7ZLE5_HUMAN	FN1	B7ZLE5	FN1 protein	247 kDa	0.058	134	82	52	62	2	7	11	4	10	14	25	-1.396792104	95% (0.033)
RM24_HUMAN	MRP1_HUMAN	ABCC1	P33527	Multidrug resistance-associated protein 1 (ATP-binding cassette sub-family C member 1) (Leukotriene C(4) transporter) (LTC4 transporter)	172 kDa	0.18	52	32	20	12	6	12	2	3	2	6	9	-1.397721599	0% (0.13)
IF6_HUMAN	LAP2A_HUMAN	TMPO	P42166	Lamina-associated polypeptide 2, isoform alpha (Thymopoietin isoform alpha)	75 kDa	0.16	52	32	20	4	9	12	7	5	0	12	3	-1.397721599	0% (0.13)

				(TP alpha) (Thymopoietin-related peptide isoform alpha) (TPRP isoform alpha) [Cleaved into: Thymopoietin (TP) (Splenin); Thymopentin (TP5)]															
ELMD2_HUMA N	VAPB_HUMAN	VAPB	O95292	Vesicle-associated membrane protein-associated protein B/C (VAMP-B/VAMP-C) (VAMP-associated protein B/C) (VAP-B/VAP-C)	27 kDa	0.19	52	32	20	11	8	7	6	7	3	6	4	- 1.3977215 99	0% (0.13)
B2R6A3_HUMA N	AP1B1_HUMAN	AP1B1	Q10567	AP-1 complex subunit beta-1 (Adapter-related protein complex 1 subunit beta-1) (Adaptor protein complex AP-1 subunit beta-1) (Beta-1-adaptin) (Beta-adaptin 1) (Clathrin assembly protein complex 1 beta large chain) (Golgi adaptor HA1/AP1 adaptin beta subunit)	105 kDa	0.087	88	54	34	12	18	16	8	10	4	13	7	- 1.4002205 61	0% (0.069)
SYFM_HUMAN	A6GV77_HUMA N	FACL5	A6GV77	Fatty acid coenzyme A ligase 5 (EC 6.2.1.3)	73 kDa	0.08	11	7	4	3	0	0	5	0	1	1	2	- 1.4035989 96	0% (0.34)
CLPX_HUMAN	HEBP1_HUMA N	HEBP1	Q9NRV9	Heme-binding protein 1 (p22HBP)	21 kDa	0.079	12	7	4	1	2	4	1	1	2	0	2	- 1.4035989 96	0% (0.34)
PDE12_HUMAN	S27A4_HUMAN	SLC27A4	Q6P1M0	Long-chain fatty acid transport protein 4 (FATP-4) (Fatty acid transport protein 4) (EC 6.2.1.-) (Solute carrier family 27 member 4)	72 kDa	0.084	11	7	4	4	0	1	3	0	2	2	0	- 1.4035989 96	0% (0.34)
B4E1A5_HUMA N	A8K4R1_HUMA N		A8K4R1	cDNA FLJ77420, highly similar to Homo sapiens cancer susceptibility candidate 4 (CASC4), transcript variant 2, mRNA	43 kDa	0.043	11	7	4	1	1	2	3	0	0	1	2	- 1.4035989 96	0% (0.34)
E7EWV1_HUM AN (+2)	B3KWE3_HUM AN		B3KWE 3	cDNA FLJ42859 fis, clone BRHIP2009414, highly similar to Bax inhibitor 1	27 kDa	0.057	12	7	4	3	2	1	2	0	2	1	1	- 1.4035989 96	0% (0.34)
J3KR97_HUMA N (+1)	PPAP_HUMAN	ACPP	P15309	Prostatic acid phosphatase (PAP) (EC 3.1.3.2) (5'-nucleotidase) (5'-NT) (EC 3.1.3.5) (Ecto-5'-nucleotidase) (Thiamine monophosphatase) (TMPase) [Cleaved into: PAPf39]	45 kDa	0.059	11	7	4	1	2	3	1	3	0	0	1	- 1.4035989 96	0% (0.34)
B3KW23_HUM AN (+4)	NNMT_HUMAN	NNMT	P40261	Nicotinamide N-methyltransferase (EC 2.1.1.1)	30 kDa	0.076	11	7	4	0	4	3	0	2	1	1	0	- 1.4035989 96	0% (0.34)
ACAP2_HUMA N	RFIP1_HUMAN	RAB11FIP1	Q6WKZ 4	Rab11 family-interacting protein 1 (Rab11-FIP1) (Rab-coupling protein)	137 kDa	0.033	11	7	4	3	2	2	0	0	1	1	2	- 1.4035989 96	0% (0.34)
EDC4_HUMAN	B4DQW0_HUM AN		B4DQW 0	cDNA FLJ51530, highly similar to COMM domain-containing protein 9	19 kDa	0.036	11	7	4	1	3	0	3	0	3	0	1	- 1.4035989 96	0% (0.34)
GDE_HUMAN	TPPC3_HUMAN	TRAPPC3	O43617	Trafficking protein particle complex subunit 3 (BET3 homolog)	20 kDa	0.037	11	7	4	3	3	1	0	1	1	1	1	- 1.4035989 96	0% (0.34)
CENPF_HUMA N	KPCD_HUMAN	PRKCD	Q05655	Protein kinase C delta type (EC 2.7.11.13) (Tyrosine-protein kinase PRKCD) (EC 2.7.10.2) (nPKC-delta) [Cleaved into: Protein kinase C delta type regulatory subunit; Protein kinase C delta type catalytic subunit (Sphingosine-dependent protein kinase-1) (SDK1)]	78 kDa	0.007 8	11	7	4	1	1	2	3	0	4	0	0	- 1.4035989 96	0% (0.34)
COIA1_HUMAN (+2)	ERD22_HUMAN	KDELR2	P33947	ER lumen protein retaining receptor 2 (ERD2-like protein 1) (ELP-1) (KDEL endoplasmic reticulum protein retention receptor 2) (KDEL receptor 2)	24 kDa	0.025	10	7	4	2	1	1	3	0	2	0	1	- 1.4035989 96	0% (0.34)
ALDR_HUMAN	NT5D3_HUMA N	NT5DC3	Q86UY8	5'-nucleotidase domain-containing protein 3 (EC 3.1.3.-) (GRP94-neighboring nucleotidase)	63 kDa	0.16	11	7	4	5	1	1	0	0	0	2	2	- 1.4035989 96	0% (0.34)

HMGB2_HUMAN (+1)	B4DNZ1_HUMAN		B4DNZ1	cDNA FLJ53633, highly similar to Transmembrane BAX inhibitor motif-containing protein 1	28 kDa	0.33	11	7	4	4	1	2	0	0	0	1	3	-1.403598996	0% (0.34)
PB1_HUMAN	ARPC3_HUMAN	ARPC3	O15145	Actin-related protein 2/3 complex subunit 3 (Arp2/3 complex 21 kDa subunit) (p21-ARC)	21 kDa	0.014	47	29	18	5	6	9	9	7	4	4	3	-1.403713117	0% (0.14)
B4DE65_HUMAN	Q6LEU0_HUMAN	STX12	Q6LEU0	STX12 protein (Fragment)	31 kDa	0.12	47	29	18	11	6	5	7	2	2	3	11	-1.403713117	0% (0.14)
TMM70_HUMAN	E7ES33_HUMAN	39326	E7ES33	Septin-7	51 kDa	0.081	47	29	18	5	7	10	6	3	5	0	6	-1.403713117	0% (0.14)
GNA11_HUMAN (+1)	GBB2_HUMAN	GNB2	P62879	Guanine nucleotide-binding protein G(I)/G(S)/G(T) subunit beta-2 (G protein subunit beta-2) (Transducin beta chain 2)	37 kDa	0.16	86	51	32	17	13	12	9	12	4	7	9	-1.403827302	0% (0.074)
E9PKU7_HUMAN	TM9S2_HUMAN	TM9SF2	Q99805	Transmembrane 9 superfamily member 2 (p76)	76 kDa	0.34	60	37	23	12	9	7	9	6	6	8	3	-1.40901891	0% (0.11)
B4DKR0_HUMAN (+3)	I7GSS2_HUMAN	HLA-C	I7GSS2	MHC class I antigen (Fragment)	32 kDa	0.085	117	72	45	13	19	14	25	5	8	14	17	-1.414982084	95% (0.037)
B3KNQ2_HUMAN (+2)	A8K6V6_HUMAN		A8K6V6	cDNA FLJ75883, highly similar to Homo sapiens glucosamine (N-acetyl)-6-sulfatase (Sanfilippo disease IIID) (GNS), mRNA	62 kDa	0.2	22	15	9	4	6	3	1	2	1	4	2	-1.416091479	0% (0.23)
ERD22_HUMAN	LTOR1_HUMAN	LAMTOR1	Q6IAA8	Ragulator complex protein LAMTOR1 (Late endosomal/lysosomal adaptor and MAPK and MTOR activator 1) (Lipid raft adaptor protein p18) (Protein associated with DRMs and endosomes) (p27Kip1-releasing factor from RhoA) (p27RF-Rho)	18 kDa	0.13	24	15	9	7	2	3	4	0	2	2	5	-1.416091479	0% (0.23)
PIGO_HUMAN	TMED4_HUMAN	TMED4	Q7Z7H5	Transmembrane emp24 domain-containing protein 4 (Endoplasmic reticulum stress-response protein 25) (ERS25) (GMP25iso) (Putative NF-kappa-B-activating protein 156) (p24 family protein alpha-3) (p24alpha3)	26 kDa	0.026	24	15	9	4	4	2	5	2	3	2	2	-1.416091479	0% (0.23)
EMC8_HUMAN (+1)	B4E3I3_HUMAN		B4E3I3	cDNA FLJ59614, highly similar to Ubiquitin fusion degradation protein 1 homolog	35 kDa	0.2	23	15	9	0	4	4	5	2	3	2	2	-1.416091479	0% (0.23)
B5MDE0_HUMAN (+1)	MVP_HUMAN	MVP	Q14764	Major vault protein (MVP) (Lung resistance-related protein)	99 kDa	0.098	189	115	72	35	31	35	17	11	22	18	21	-1.41811609	95% (0.011)
A8K6Q8_HUMAN	Q5HYI5_HUMAN	DKFZp313C1541	Q5HYI5	Putative uncharacterized protein DKFZp313C1541	23 kDa	0.41	113	69	43	18	14	0	21	0	11	0	0	-1.418351428	95% (0.040)
NT5D3_HUMAN	SPTN2_HUMAN	SPTBN2	O15020	Spectrin beta chain, non-erythrocytic 2 (Beta-III spectrin) (Spinocerebellar ataxia 5 protein)	271 kDa	0.11	37	23	14	10	3	4	6	7	2	5	0	-1.420421106	0% (0.17)
D3DNA1_HUMAN (+1)	SPB6_HUMAN	SERPINB6	P35237	Serpin B6 (Cytoplasmic antiproteinase) (CAP) (Peptidase inhibitor 6) (PI-6) (Placental thrombin inhibitor)	43 kDa	0.075	36	23	14	10	8	5	0	2	4	3	5	-1.420421106	0% (0.17)
B2R4V4_HUMAN (+1)	F6IQP1_HUMAN	HLA-C	F6IQP1	MHC class I antigen (Fragment)	39 kDa	0.19	107	66	41	0	19	12	22	0	7	0	0	-1.422041007	95% (0.042)
B7Z2D6_HUMAN (+1)	B0UXB6_HUMAN	ABHD16A	B0UXB6	Abhydrolase domain-containing protein 16A (HLA-B associated transcript 5, isoform CRA_b)	68 kDa	0.014	63	39	24	12	5	5	18	1	3	10	10	-1.4239887	0% (0.096)
SNF5_HUMAN	B7Z3E5_HUMAN		B7Z3E5	cDNA FLJ53573, highly similar to Myosin Ic	119 kDa	0.15	127	79	49	27	17	16	19	8	6	16	19	-1.426849327	95% (0.028)
NTF2_HUMAN	TACD2_HUMAN	TACSTD2	P09758	Tumor-associated calcium signal transducer 2 (Cell surface glycoprotein Trop-2) (Membrane component chromosome 1 surface marker	36 kDa	0.24	97	60	37	21	12	14	13	17	3	4	13	-1.430581799	95% (0.048)

				1) (Pancreatic carcinoma marker protein GA733-1)														
SAFB2_HUMAN	RRP5_HUMAN	PDCD11	Q14690	Protein RRP5 homolog (NF-kappa-B-binding protein) (NFBP) (Programmed cell death protein 11)	209 kDa	0.065	32	20	12	1	5	7	7	6	2	3	1	-1.432563978	0% (0.18)
I7B0X5_HUMAN	B4DVA7_HUMAN		B4DVA7	Beta-hexosaminidase (EC 3.2.1.52)	62 kDa	0.41	32	20	12	2	5	9	5	1	1	7	3	-1.432563978	0% (0.18)
E41L5_HUMAN	J3QRS3_HUMAN	MYL12A	J3QRS3	Myosin regulatory light chain 12A (Myosin regulatory light chain MRCL3, isoform CRA_b)	20 kDa	0.064	32	20	12	4	7	4	5	2	4	3	3	-1.432563978	0% (0.18)
E5KNQ5_HUMAN	B2ZDQ1_HUMAN	NGAL	B2ZDQ1	Neutrophil gelatinase-associated lipocalin	23 kDa	0.059	168	105	65	25	24	30	28	6	22	13	24	-1.433037004	95% (0.012)
B4E1G6_HUMAN (+1)	Q53HG7_HUMAN		Q53HG7	Cortactin isoform a variant (Fragment)	62 kDa	0.13	19	12	7	4	1	4	3	1	4	0	2	-1.434562562	0% (0.25)
A8K644_HUMAN (+1)	B7Z7N1_HUMAN		B7Z7N1	cDNA FLJ50915, highly similar to NADH dehydrogenase (ubiquinone) 1 beta subcomplex subunit 9 (EC 1.6.5.3)	21 kDa	0.071	21	12	7	3	1	1	7	3	1	1	1	-1.434562562	0% (0.25)
SYAM_HUMAN	D7RF68_HUMAN		D7RF68	AGTRAP-BRAF fusion protein	66 kDa	0.049	18	12	7	5	4	3	0	0	2	0	4	-1.434562562	0% (0.25)
FPPS_HUMAN	A8K935_HUMAN		A8K935	cDNA FLJ75184, highly similar to Homo sapiens K-Cl cotransporter KCC3 mRNA	128 kDa	0.13	5	4	2	2	1	1	0	0	0	0	2	-1.442846402	0% (0.40)
Q29849_HUMAN	F8VSD4_HUMAN	UBE2N	F8VSD4	Ubiquitin-conjugating enzyme E2 N	12 kDa	0.25	6	4	2	0	0	3	1	0	0	1	0	-1.442846402	0% (0.40)
POF1B_HUMAN	A8K5K5_HUMAN	EIF3G	A8K5K5	Eukaryotic translation initiation factor 3 subunit G (eIF3g) (Eukaryotic translation initiation factor 3 RNA-binding subunit) (Eukaryotic translation initiation factor 3 subunit 4) (eIF-3-delta) (eIF3 p42) (eIF3 p44)	36 kDa	0.065	6	4	2	1	1	2	1	1	0	1	0	-1.442846402	0% (0.40)
CBP_HUMAN	PNKD_HUMAN	PNKD	Q8N490	Probable hydrolase PNKD (EC 3.-.-.-) (Myofibrillogenesis regulator 1) (MR-1) (Paroxysmal nonkinesiogenic dyskinesia protein) (Trans-activated by hepatitis C virus core protein 2)	43 kDa	0.014	6	4	2	2	0	0	2	0	2	0	0	-1.442846402	0% (0.40)
CO9_HUMAN	A8K8J9_HUMAN	DCTN2	A8K8J9	Dynactin 2 (P50), isoform CRA_b (cDNA FLJ31120 fis, clone IMR322000730, highly similar to Dynactin subunit 2) (cDNA FLJ77785)	34 kDa	0.084	6	4	2	0	2	2	0	0	1	0	1	-1.442846402	0% (0.40)
B7ZB83_HUMAN	SNF5_HUMAN	SMARCB1	Q12824	SWI/SNF-related matrix-associated actin-dependent regulator of chromatin subfamily B member 1 (BRG1-associated factor 47) (BAF47) (Integrase interactor 1 protein) (SNF5 homolog) (hSNF5)	44 kDa	0.098	5	4	2	2	0	0	2	0	0	1	1	-1.442846402	0% (0.40)
CO6A3_HUMAN	B7Z410_HUMAN		B7Z410	cDNA FLJ50791, highly similar to Nitrilase homolog 1 (EC 3.5.-.-)	34 kDa	0.01	6	4	2	0	1	0	3	2	0	0	0	-1.442846402	0% (0.40)
B4DHE8_HUMAN (+1)	J3KPT4_HUMAN	TRABD	J3KPT4	TraB domain-containing protein	43 kDa	0.15	6	4	2	3	0	1	0	0	0	0	2	-1.442846402	0% (0.40)
ODBB_HUMAN	STX6_HUMAN	STX6	O43752	Syntaxin-6	29 kDa	0.1	6	4	2	2	1	1	0	0	0	2	0	-1.442846402	0% (0.40)
ACPH_HUMAN (+1)	ODR4_HUMAN	ODR4	Q5SWX8	Protein odr-4 homolog (hODR-4) (LAG1-interacting protein) (Transactivated by transforming growth factor beta protein 1)	51 kDa	0.057	6	4	2	4	0	0	0	0	0	0	2	-1.442846402	0% (0.40)
B4DNZ1_HUMAN (+9)	Q53HE3_HUMAN		Q53HE3	Breast carcinoma amplified sequence 2 variant (Fragment)	26 kDa	0.06	6	4	2	0	0	0	4	0	0	2	0	-1.442846402	0% (0.40)

RT31_HUMAN	ERD21_HUMAN	KDELR1	P24390	ER lumen protein retaining receptor 1 (KDEL endoplasmic reticulum protein retention receptor 1) (KDEL receptor 1) (Putative MAPK-activating protein PM23)	25 kDa	0.13	6	4	2	1	1	0	2	0	2	0	0	-1.442846402	0% (0.40)
A8K5L5_HUMAN (+3)	VPS29_HUMAN	VPS29	Q9UBQ0	Vacuolar protein sorting-associated protein 29 (hVPS29) (EC 3.1.3.3) (PEP11 homolog) (Vesicle protein sorting 29)	21 kDa	0.075	6	4	2	0	2	1	1	0	2	0	0	-1.442846402	0% (0.40)
G3XAI2_HUMAN (+1)	DERL1_HUMAN	DERL1	Q9BUN8	Derlin-1 (Degradation in endoplasmic reticulum protein 1) (DERtrin-1) (Der1-like protein 1)	29 kDa	0.018	43	25	15	6	9	4	6	4	4	3	4	-1.442965922	0% (0.15)
B4DWG1_HUMAN (+2)	B5BU25_HUMAN	U2AF2	B5BU25	U2 small nuclear RNA auxiliary factor 2 isoform b	53 kDa	0.062	40	25	15	4	7	7	7	5	4	6	1	-1.442965922	0% (0.15)
GTPBA_HUMAN	L7QJN0_HUMAN	HLA-B	L7QJN0	MHC class I antigen (Fragment)	32 kDa	0.12	74	46	28	0	15	0	11	0	0	0	12	-1.443085507	0% (0.069)
NCF1B_HUMAN (+2)	E9PC28_HUMAN	PTPRC	E9PC28	Receptor-type tyrosine-protein phosphatase C	142 kDa	0.087	61	38	23	3	11	19	5	0	2	15	6	-1.445874591	0% (0.090)
B4DUC8_HUMAN (+3)	RAB43_HUMAN	RAB43	Q86YS6	Ras-related protein Rab-43 (Ras-related protein Rab-41)	23 kDa	0.12	61	38	23	9	9	10	10	5	7	5	6	-1.445874591	0% (0.090)
LTOR4_HUMAN	RL10A_HUMAN	RPL10A	P62906	60S ribosomal protein L10a (CSA-19) (Neural precursor cell expressed developmentally down-regulated protein 6) (NEDD-6)	25 kDa	0.28	103	64	39	17	13	16	19	14	10	7	8	-1.448313266	95% (0.037)
B3KSR7_HUMAN (+1)	MARH5_HUMAN	38412	Q9NX47	E3 ubiquitin-protein ligase MARCH5 (EC 6.3.2.-) (Membrane-associated RING finger protein 5) (Membrane-associated RING-CH protein V) (MARCH-V) (Mitochondrial ubiquitin ligase) (MITOL) (RING finger protein 153)	31 kDa	0.07	27	17	10	8	3	1	5	5	2	0	3	-1.449028975	0% (0.20)
B4DGX2_HUMAN (+1)	GNAQ_HUMAN	GNAQ	P50148	Guanine nucleotide-binding protein G(q) subunit alpha (Guanine nucleotide-binding protein alpha-q)	42 kDa	0.11	27	17	10	9	3	3	2	3	0	4	3	-1.449028975	0% (0.20)
NUCG_HUMAN	DCMC_HUMAN	MLYCD	O95822	Malonyl-CoA decarboxylase, mitochondrial (MCD) (EC 4.1.1.9)	55 kDa	0.18	27	17	10	7	7	3	0	5	0	2	3	-1.449028975	0% (0.20)
A8K693_HUMAN	PDIP2_HUMAN	POLDIP2	Q9Y2S7	Polymerase delta-interacting protein 2 (38 kDa DNA polymerase delta interaction protein) (p38)	42 kDa	0.13	27	17	10	4	3	4	6	4	0	3	3	-1.449028975	0% (0.20)
LARP1_HUMAN	H0Y9V9_HUMAN	RPL9	H0Y9V9	60S ribosomal protein L9 (Fragment)	22 kDa	0.026	48	30	18	7	8	6	9	4	7	2	5	-1.450135494	0% (0.12)
B4DG11_HUMAN (+1)	Q5JQ44_HUMAN	DKFZp547A0616	Q5JQ44	Putative uncharacterized protein DKFZp547A0616 (Fragment)	20 kDa	0.064	56	35	21	10	9	7	8	4	9	5	3	-1.455380173	0% (0.096)
E7ET76_HUMAN	GSLG1_HUMAN	GLG1	Q92896	Golgi apparatus protein 1 (CFR-1) (Cysteine-rich fibroblast growth factor receptor) (E-selectin ligand 1) (ESL-1) (Golgi sialoglycoprotein MG-160)	135 kDa	0.086	35	22	13	7	9	4	3	3	2	5	3	-1.457417492	0% (0.16)
PERE_HUMAN	B4DG42_HUMAN		B4DG42	cDNA FLJ53753, highly similar to Myeloid-associated differentiation marker	31 kDa	0.05	34	22	13	9	4	4	5	2	4	4	3	-1.457417492	0% (0.16)
GIMA1_HUMAN	DDX1_HUMAN	DDX1	Q92499	ATP-dependent RNA helicase DDX1 (EC 3.6.4.13) (DEAD box protein 1) (DEAD box protein retinoblastoma) (DBP-RB)	82 kDa	0.082	35	22	13	4	4	4	10	2	6	5	0	-1.457417492	0% (0.16)
B2R4F3_HUMAN (+3)	F5GYR8_HUMAN	USO1	F5GYR8	General vesicular transport factor p115	109 kDa	0.13	35	22	13	2	8	6	6	4	6	3	0	-1.457417492	0% (0.16)
QCR9_HUMAN	B4DT31_HUMAN	FUBP1	B4DT31	Far upstream element-binding protein 1 (cDNA FLJ53425, highly similar to Far upstream element-binding protein 1)	70 kDa	0.38	35	22	13	2	7	7	6	1	1	9	2	-1.457417492	0% (0.16)

EHD1_HUMAN	B4DT35_HUMAN	NUP54	B4DT35	Nucleoporin p54 (cDNA FLJ52746, highly similar to Nucleoporin p54)	51 kDa	0.18	35	22	13	4	5	7	6	2	5	3	3	-1.457417492	0% (0.16)
NCF2_HUMAN	TIDC1_HUMAN	TIMMDC1	Q9NPL8	Translocase of inner mitochondrial membrane domain-containing protein 1 (Protein M5-14)	32 kDa	0.074	35	22	13	6	7	5	4	6	2	1	4	-1.457417492	0% (0.16)
GAR1_HUMAN	Q6PK82_HUMAN	AP3D1	Q6PK82	AP3D1 protein (Fragment)	99 kDa	0.074	14	9	5	4	1	3	2	1	1	2	1	-1.464863524	0% (0.28)
A8K8E1_HUMAN (+1)	B4DGM3_HUMAN	SMARCE1	B4DGM3	SWI/SNF-related matrix-associated actin-dependent regulator of chromatin subfamily E member 1 (cDNA FLJ55202, highly similar to SWI/SNF-related matrix-associatedactin-dependent regulator of chromatin subfamily E member 1)	45 kDa	0.072	14	9	5	6	1	2	0	0	0	2	3	-1.464863524	0% (0.28)
PHF2_HUMAN	B7Z954_HUMAN		B7Z954	cDNA FLJ61560, highly similar to Tight junction protein ZO-2	125 kDa	0.02	14	9	5	2	1	2	4	3	1	1	0	-1.464863524	0% (0.28)
B1AUU8_HUMAN (+2)	B7Z587_HUMAN		B7Z587	cDNA FLJ51273, highly similar to Transmembrane protein 11	19 kDa	0.062	13	9	5	3	0	2	3	1	2	0	2	-1.464863524	0% (0.28)
B2R7V4_HUMAN (+1)	MA1B1_HUMAN	MAN1B1	Q9UKM7	Endoplasmic reticulum mannosyl-oligosaccharide 1,2-alpha-mannosidase (EC 3.2.1.113) (ER alpha-1,2-mannosidase) (ER mannosidase 1) (ERMan1) (Man9GlcNAc2-specific-processing alpha-mannosidase) (Mannosidase alpha class 1B member 1)	80 kDa	0.075	14	9	5	0	2	3	4	2	0	3	0	-1.464863524	0% (0.28)
COX15_HUMAN	Q3B7X1_HUMAN	TMX3	Q3B7X1	TMX3 protein (Fragment)	49 kDa	0.098	14	9	5	6	3	0	0	0	1	1	4	-1.464863524	0% (0.28)
RRF2M_HUMAN	LGAT1_HUMAN	LPGAT1	Q92604	Acyl-CoA:lysophosphatidylglycerol acyltransferase 1 (EC 2.3.1.-)	43 kDa	0.06	14	9	5	7	1	1	0	0	1	1	3	-1.464863524	0% (0.28)
TMM68_HUMAN	SNP23_HUMAN	SNAP23	O00161	Synaptosomal-associated protein 23 (SNAP-23) (Vesicle-membrane fusion protein SNAP-23)	23 kDa	0.13	14	9	5	3	1	2	3	1	0	2	2	-1.464863524	0% (0.28)
B4DMB5_HUMAN (+1)	DHB7_HUMAN	HSD17B7	P56937	3-keto-steroid reductase (EC 1.1.1.270) (17-beta-hydroxysteroid dehydrogenase 7) (17-beta-HSD 7) (Estradiol 17-beta-dehydrogenase 7) (EC 1.1.1.62)	38 kDa	0.11	14	9	5	2	2	2	3	3	0	1	1	-1.464863524	0% (0.28)
ACPM_HUMAN	B2RBG3_HUMAN		B2RBG3	cDNA, FLJ95493, highly similar to Homo sapiens fucosidase, alpha-L- 1, tissue (FUCA1), mRNA	53 kDa	0.15	51	32	19	9	6	7	10	3	3	6	8	-1.466765993	0% (0.10)
TRPM4_HUMAN	D0UU70_HUMAN	MECP2	D0UU70	Methyl CpG binding protein 2 isoform 1	52 kDa	0.068	22	14	8	5	3	3	3	1	2	1	4	-1.472619638	0% (0.21)
HTRA2_HUMAN	PAFA2_HUMAN	PAFAH2	Q99487	Platelet-activating factor acetylhydrolase 2, cytoplasmic (EC 3.1.1.47) (Serine-dependent phospholipase A2) (SD-PLA2) (hSD-PLA2)	44 kDa	0.059	22	14	8	11	0	1	2	1	0	1	6	-1.472619638	0% (0.21)
B7Z5W1_HUMAN (+3)	IMPA3_HUMAN	IMPAD1	Q9NX62	Inositol monophosphatase 3 (IMP 3) (IMPase 3) (EC 3.1.3.25) (EC 3.1.3.7) (Golgi 3-prime phosphoadenosine 5-prime phosphate 3-prime phosphatase) (Golgi-resident PAP phosphatase) (gPAPP) (Inositol monophosphatase domain-containing protein 1) (Inositol-1(or 4)-monophosphatase 3) (Myo-inositol monophosphatase A3)	39 kDa	0.076	22	14	8	5	5	3	1	3	3	1	1	-1.472619638	0% (0.21)
SELT_HUMAN	SAP18_HUMAN	SAP18	O00422	Histone deacetylase complex subunit SAP18 (18 kDa Sin3-associated polypeptide) (2HOR0202) (Cell growth-	18 kDa	0.15	22	14	8	4	2	1	7	3	3	1	1	-1.472619638	0% (0.21)

				inhibiting gene 38 protein) (Sin3-associated polypeptide p18)															
B4DR61_HUMAN (+1)	J3KRX2_HUMAN	SLC39A11	J3KRX2	Zinc transporter ZIP11 (Fragment)	21 kDa	0.081	22	14	8	5	4	2	3	0	1	3	4	-1.472619638	0% (0.21)
AG10A_HUMAN (+1)	J9JID7_HUMAN	LMNB2	J9JID7	Lamin B2, isoform CRA_a (Lamin-B2)	70 kDa	0.038	90	57	34	30	10	9	8	8	0	11	15	-1.476307579	95% (0.039)
B7Z6W8_HUMAN (+4)	A4D2P0_HUMAN	RAC1	A4D2P0	Ras-related C3 botulinum toxin substrate 1 (Rho family, small GTP binding protein Rac1) (Ras-related C3 botulinum toxin substrate 1 (Rho family, small GTP binding protein Rac1), isoform CRA_e) (cDNA FLJ77333, highly similar to Homo sapiens ras-related C3 botulinum toxin substrate 1 (rho family, small GTP binding protein Rac1) (RAC1), transcript variant Rac1b, mRNA)	23 kDa	0.11	30	19	11	6	4	4	6	2	3	3	3	-1.476592221	0% (0.17)
DJB12_HUMAN (+1)	B2RCX0_HUMAN		B2RCX0	cDNA, FLJ96345, Homo sapiens SET translocation (myeloid leukemia-associated) (SET),mRNA	32 kDa	0.067	30	19	11	0	6	4	9	2	2	2	5	-1.476592221	0% (0.17)
B5MCC7_HUMAN (+3)	B7Z1V4_HUMAN		B7Z1V4	cDNA FLJ56034, highly similar to 4-aminobutyrate aminotransferase, mitochondrial (EC 2.6.1.19)	58 kDa	0.19	30	19	11	3	7	8	1	1	2	3	5	-1.476592221	0% (0.17)
A8K7A4_HUMAN (+1)	B4DJQ8_HUMAN		B4DJQ8	cDNA FLJ55694, highly similar to Dipeptidyl-peptidase 1 (EC 3.4.14.1)	50 kDa	0.077	38	24	14	1	7	9	7	1	2	6	5	-1.47901418	0% (0.14)
NDUB3_HUMAN	B4DSN3_HUMAN		B4DSN3	cDNA FLJ60345, highly similar to Protein transport protein Sec24C	107 kDa	0.29	38	24	14	3	6	5	11	1	6	7	0	-1.47901418	0% (0.14)
A6XND5_HUMAN (+3)	B7Z992_HUMAN		B7Z992	cDNA FLJ53698, highly similar to Gelsolin	79 kDa	0.085	188	119	71	26	36	34	21	11	15	21	23	-1.487312719	95% (0.0043)
H0YNA7_HUMAN (+1)	F8W914_HUMAN	RTN4	F8W914	Reticulon	37 kDa	0.15	137	86	51	44	16	15	13	7	18	0	19	-1.492020798	95% (0.013)
SHLB1_HUMAN	RN213_HUMAN	RNF213	Q63HN8	E3 ubiquitin-protein ligase RNF213 (EC 6.3.2.-) (ALK lymphoma oligomerization partner on chromosome 17) (Mysterin) (RING finger protein 213)	591 kDa	0.074	97	61	36	7	11	10	34	10	21	1	3	-1.493010819	95% (0.031)
B7Z787_HUMAN (+2)	A8K4W0_HUMAN	RPS3A	A8K4W0	40S ribosomal protein S3a	30 kDa	0.067	97	61	36	13	13	12	24	11	15	7	4	-1.493010819	95% (0.031)
Q6ZQN2_HUMAN	B4DE59_HUMAN		B4DE59	cDNA FLJ60424, highly similar to Junction plakoglobin	63 kDa	0.33	525	329	196	141	47	51	91	53	48	40	55	-1.497523368	95% (< 0.00010)
DNJC5_HUMAN	SQRD_HUMAN	SQRDL	Q9Y6N5	Sulfide:quinone oxidoreductase, mitochondrial (EC 1.-.-.-)	50 kDa	0.16	267	169	100	61	33	41	35	7	24	29	39	-1.502952522	95% (0.00065)
CELF1_HUMAN (+1)	RS20_HUMAN	RPS20	P60866	40S ribosomal protein S20	13 kDa	0.08	25	16	9	3	4	5	4	3	3	2	1	-1.503254887	0% (0.18)
NIBAN_HUMAN	B2R6X6_HUMAN		B2R6X6	Peptidyl-prolyl cis-trans isomerase (EC 5.2.1.8)	22 kDa	0.043	17	11	6	2	5	3	1	0	0	4	2	-1.509233069	0% (0.23)
RM14_HUMAN	B4DDM6_HUMAN	BUB3	B4DDM6	Mitotic checkpoint protein BUB3 (cDNA FLJ57899, highly similar to Mitotic checkpoint protein BUB3)	28 kDa	0.13	17	11	6	1	3	3	4	1	2	2	1	-1.509233069	0% (0.23)
B4DS83_HUMAN	AN32E_HUMAN	ANP32E	Q9BTT0	Acidic leucine-rich nuclear phosphoprotein 32 family member E (LANP-like protein) (LANP-L)	31 kDa	0.049	17	11	6	0	1	2	8	1	4	1	0	-1.509233069	0% (0.23)
B5BU81_HUMAN (+2)	GRPE1_HUMAN	GRPEL1	Q9HAV7	GrpE protein homolog 1, mitochondrial (HMGE) (Mt-GrpE#1)	24 kDa	0.15	52	33	19	3	10	14	6	6	4	1	8	-1.510898675	0% (0.083)
AHSA1_HUMAN (+1)	APMAP_HUMAN	APMAP	Q9HDC9	Adipocyte plasma membrane-associated protein (Protein BSCv)	46 kDa	0.12	89	55	32	13	13	15	15	5	6	5	17	-1.511375057	95% (0.034)

SPS1_HUMAN	B3KVN0_HUMAN		B3KVN0	cDNA FLJ16785 fis, clone NT2RI2015342, highly similar to Solute carrier family 2, facilitated glucose transporter member 1	46 kDa	0.14	79	50	29	10	12	17	11	11	8	5	5	-1.513563675	95% (0.041)
EFHD1_HUMAN	B3KY04_HUMAN		B3KY04	cDNA FLJ46506 fis, clone THYMU3030752, highly similar to BTB/POZ domain-containing protein KCTD12	36 kDa	0.17	36	23	13	1	11	8	3	1	6	3	3	-1.520121651	0% (0.12)
B4DFY5_HUMAN (+1)	LYRIC_HUMAN	MTDH	Q86UE4	Protein LYRIC (3D3/LYRIC) (Astrocyte elevated gene-1 protein) (AEG-1) (Lysine-rich CEACAM1 co-isolated protein) (Metadherin) (Metastasis adhesion protein)	64 kDa	0.075	35	23	13	7	7	3	5	2	6	2	3	-1.520121651	0% (0.12)
CAH2_HUMAN	B4DDB9_HUMAN		B4DDB9	cDNA FLJ56339, highly similar to Signal peptidase complex subunit 2 (EC 3.4.-.-)	25 kDa	0.19	36	23	13	8	3	3	7	1	3	4	6	-1.520121651	0% (0.12)
Q5J8M4_HUMAN	A8K4K9_HUMAN		A8K4K9	cDNA FLJ76169	35 kDa	0.12	36	23	13	6	5	3	10	3	6	1	3	-1.520121651	0% (0.12)
PAXB1_HUMAN	ACO13_HUMAN	ACOT13	Q9NPJ3	Acyl-coenzyme A thioesterase 13 (Acyl-CoA thioesterase 13) (EC 3.1.2.-) (Thioesterase superfamily member 2) [Cleaved into: Acyl-coenzyme A thioesterase 13, N-terminally processed]	15 kDa	0.031	37	23	13	4	8	7	5	6	3	2	2	-1.520121651	0% (0.12)
B7Z966_HUMAN (+1)	LDHA_HUMAN	LDHA	P00338	L-lactate dehydrogenase A chain (LDH-A) (EC 1.1.1.27) (Cell proliferation-inducing gene 19 protein) (LDH muscle subunit) (LDH-M) (Renal carcinoma antigen NY-REN-59)	37 kDa	0.083	201	127	74	24	34	34	36	22	10	21	21	-1.523112125	95% (0.0021)
Q58EY4_HUMAN (+1)	F5GZD0_HUMAN	NPTN	F5GZD0	Neuroplastin	44 kDa	0.06	9	6	3	3	1	0	2	1	0	0	2	-1.523695369	0% (0.31)
B7Z410_HUMAN	B7Z920_HUMAN		B7Z920	cDNA FLJ61714, highly similar to Tripeptidyl-peptidase 2 (EC 3.4.14.10) (Fragment)	118 kDa	0.13	10	6	3	0	2	4	0	0	0	3	0	-1.523695369	0% (0.31)
A8K321_HUMAN (+2)	B7Z4S4_HUMAN		B7Z4S4	cDNA FLJ52567, highly similar to Renin receptor	36 kDa	0.093	9	6	3	1	2	1	2	0	0	0	3	-1.523695369	0% (0.31)
FA5_HUMAN	SRS11_HUMAN	SRSF11	Q05519	Serine/arginine-rich splicing factor 11 (Arginine-rich 54 kDa nuclear protein) (p54) (Splicing factor, arginine/serine-rich 11)	54 kDa	0.017	9	6	3	0	3	1	2	1	0	2	0	-1.523695369	0% (0.31)
ATG7_HUMAN (+1)	NDK3_HUMAN	NME3	Q13232	Nucleoside diphosphate kinase 3 (NDK 3) (NDP kinase 3) (EC 2.7.4.6) (DR-nm23) (Nucleoside diphosphate kinase C) (NDPKC) (nm23-H3)	19 kDa	0.088	9	6	3	3	0	1	2	1	0	2	0	-1.523695369	0% (0.31)
P3C2A_HUMAN	A8K337_HUMAN		A8K337	cDNA FLJ77862, highly similar to Homo sapiens catechol-O-methyltransferase domain containing 1 (COMTD1), mRNA	29 kDa	0.018	10	6	3	1	0	0	5	2	0	2	0	-1.523695369	0% (0.31)
E9PHY5_HUMAN (+1)	KPRA_HUMAN	PRPSAP1	Q14558	Phosphoribosyl pyrophosphate synthase-associated protein 1 (PRPP synthase-associated protein 1) (39 kDa phosphoribosypyrophosphate synthase-associated protein) (PAP39)	39 kDa	0.045	9	6	3	0	4	2	0	1	0	2	0	-1.523695369	0% (0.31)
NFKB2_HUMAN	EMC8_HUMAN	EMC8	O43402	ER membrane protein complex subunit 8 (Neighbor of COX4) (Protein FAM158B)	24 kDa	0.054	9	6	3	1	4	1	0	0	1	0	2	-1.523695369	0% (0.31)
CP4FB_HUMAN	NCF1B_HUMAN	NCF1B	A6NI72	Putative neutrophil cytosol factor 1B (NCF-1B) (Putative SH3 and PX domain-containing protein 1B)	45 kDa	0.09	9	6	3	0	3	3	0	0	1	0	2	-1.523695369	0% (0.31)
PGH2_HUMAN	NUCG_HUMAN	ENDOG	Q14249	Endonuclease G, mitochondrial (Endo G) (EC 3.1.30.-)	33 kDa	0.12	9	6	3	6	0	0	0	1	0	2	0	-1.523695369	0% (0.31)

E9PPN3_HUMAN (+1)	B7Z6W8_HUMAN		B7Z6W8	P2X purinoceptor	40 kDa	0.039	9	6	3	2	1	2	1	0	0	1	2	-1.523695369	0% (0.31)
A8K5B6_HUMAN (+2)	H0YNA7_HUMAN	SPPL2A	H0YNA7	Signal peptide peptidase-like 2A (Fragment)	17 kDa	0.052	9	6	3	1	2	2	1	0	0	3	0	-1.523695369	0% (0.31)
B2RBH6_HUMAN (+1)	B7Z966_HUMAN		B7Z966	cDNA FLJ58410, highly similar to Solute carrier family 2, facilitated glucosetransporter member 3	47 kDa	0.13	9	6	3	0	3	3	0	0	1	0	2	-1.523695369	0% (0.31)
CTR2_HUMAN	B3KWP3_HUMAN		B3KWP3	cDNA FLJ43503 fis, clone PEBLM2006215, highly similar to Mitochondrial folate transporter/carrier	34 kDa	0.062	8	6	3	1	1	0	4	1	1	0	1	-1.523695369	0% (0.31)
G3V2F7_HUMAN	B4DX69_HUMAN		B4DX69	cDNA FLJ55158, highly similar to Thioredoxin, mitochondrial	22 kDa	0.092	9	6	3	0	2	2	2	3	0	0	0	-1.523695369	0% (0.31)
B7ZKY2_HUMAN (+3)	NRAM1_HUMAN	SLC11A1	P49279	Natural resistance-associated macrophage protein 1 (NRAMP 1) (Solute carrier family 11 member 1)	60 kDa	0.043	9	6	3	0	3	3	0	0	0	0	3	-1.523695369	0% (0.31)
A8K8K1_HUMAN (+1)	B2RB23_HUMAN		B2RB23	cDNA, FLJ95265, highly similar to Homo sapiens acetyl-Coenzyme A acyltransferase 2 (mitochondrial 3-oxoacyl-Coenzyme A thiolase) (ACAA2), nuclear gene encoding mitochondrial protein, mRNA	42 kDa	0.093	101	64	37	21	14	19	10	6	7	8	16	-1.524085934	95% (0.022)
B7Z8X5_HUMAN (+2)	GNAI2_HUMAN	GNAI2	P04899	Guanine nucleotide-binding protein G(i) subunit alpha-2 (Adenylate cyclase-inhibiting G alpha protein)	40 kDa	0.043	176	112	65	29	32	31	20	10	13	21	21	-1.527586422	95% (0.0034)
GLCE_HUMAN (+1)	H0YL69_HUMAN	PSMA4	H0YL69	Proteasome subunit alpha type (EC 3.4.25.1) (Fragment)	26 kDa	0.066	28	18	10	1	6	3	8	1	2	5	2	-1.528447464	0% (0.16)
MTR1_HUMAN	H7BXZ6_HUMAN	RHOT1	H7BXZ6	Mitochondrial Rho GTPase (EC 3.6.5.-)	68 kDa	0.038	28	18	10	7	5	3	3	3	0	4	2	-1.528447464	0% (0.16)
B4DIB1_HUMAN (+3)	ANXA1_HUMAN	ANXA1	P04083	Annexin A1 (Annexin I) (Annexin-1) (Calpactin II) (Calpactin-2) (Chromobindin-9) (Lipocortin I) (Phospholipase A2 inhibitory protein) (p35)	39 kDa	0.14	436	273	159	71	63	60	78	51	32	33	44	-1.530433446	95% (< 0.00010)
PORED_HUMAN	ARP2_HUMAN	ACTR2	P61160	Actin-related protein 2 (Actin-like protein 2)	45 kDa	0.072	140	90	52	20	24	27	20	16	15	11	11	-1.531175916	95% (0.0076)
RBM28_HUMAN	VPP3_HUMAN	TCIRG1	Q13488	V-type proton ATPase 116 kDa subunit a isoform 3 (V-ATPase 116 kDa isoform a3) (Osteoclastic proton pump 116 kDa subunit) (OC-116 kDa) (OC116) (T-cell immune regulator 1) (T-cell immune response cDNA7 protein) (TIRC7) (Vacuolar proton translocating ATPase 116 kDa subunit a isoform 3)	93 kDa	0.042	95	61	35	23	14	18	6	0	2	9	24	-1.534219405	95% (0.023)
D3DNT9_HUMAN (+1)	Q59H06_HUMAN		Q59H06	Transporter 2, ATP-binding cassette, sub-family B isoform 1 variant (Fragment)	78 kDa	0.045	76	49	28	8	12	10	20	0	6	11	11	-1.534769173	95% (0.038)
GOGA3_HUMAN	G3V5Z7_HUMAN	PSMA6	G3V5Z7	Proteasome subunit alpha type (EC 3.4.25.1)	28 kDa	0.031	39	25	14	1	8	6	10	4	2	5	3	-1.53760876	0% (0.11)
GLNA_HUMAN	B2R6U8_HUMAN		B2R6U8	cDNA, FLJ93125, highly similar to Homo sapiens cleavage and polyadenylation specific factor 5, 25 kDa(CPSF5), mRNA	26 kDa	0.088	39	25	14	2	6	8	10	1	2	9	2	-1.53760876	0% (0.11)
B2RAU5_HUMAN (+1)	SYNM_HUMAN	NARS2	Q96I59	Probable asparagine--tRNA ligase, mitochondrial (EC 6.1.1.22) (Asparaginyl-tRNA synthetase) (AsnRS)	54 kDa	0.097	20	13	7	3	5	4	1	4	0	1	2	-1.54285126	0% (0.20)
B3KMQ6_HUMAN	VP26A_HUMAN	VPS26A	O75436	Vacuolar protein sorting-associated protein 26A (Vesicle protein sorting 26A) (hVPS26)	38 kDa	0.031	20	13	7	2	4	4	3	1	6	1	0	-1.54285126	0% (0.20)

SCEL_HUMAN	B7ZKM0_HUMAN	SART3	B7ZKM0	SART3 protein (Squamous cell carcinoma antigen recognized by T-cells 3)	106 kDa	0.039	20	13	7	3	5	2	3	0	2	4	1	-1.54285126	0% (0.20)
C2D1A_HUMAN	COR1A_HUMAN	CORO1A	P31146	Coronin-1A (Coronin-like protein A) (Clipin-A) (Coronin-like protein p57) (Tryptophan aspartate-containing coat protein) (TACO)	51 kDa	0.032	20	13	7	0	4	5	4	0	2	3	2	-1.54285126	0% (0.20)
GP110_HUMAN	CB047_HUMAN	C2orf47	Q8WWC4	Uncharacterized protein C2orf47, mitochondrial	33 kDa	0.03	31	20	11	5	4	3	7	1	5	3	2	-1.549530269	0% (0.13)
IPRI_HUMAN	DYSF_HUMAN	DYSF	O75923	Dysferlin (Dystrophy-associated fer-1-like protein) (Fer-1-like protein 1)	237 kDa	0.097	31	20	11	6	4	9	1	0	1	1	8	-1.549530269	0% (0.13)
B4DKC8_HUMAN	Q6ZS74_HUMAN	RALB	Q6ZS74	Ras-related protein Ral-B (V-ral simian leukemia viral oncogene homolog B (Ras related GTP binding protein), isoform CRA_b) (cDNA FLJ45773 fis, clone NETRP2003448, highly similar to Ras-related protein RAL-B)	26 kDa	0.033	75	48	27	19	9	9	11	3	9	3	12	-1.557475849	95% (0.035)
D6RE83_HUMAN (+1)	P4HA1_HUMAN	P4HA1	P13674	Prolyl 4-hydroxylase subunit alpha-1 (4-PH alpha-1) (EC 1.14.11.2) (Procollagen-proline,2-oxoglutarate-4-dioxygenase subunit alpha-1)	61 kDa	0.2	171	109	62	16	37	40	16	6	22	22	12	-1.557663076	95% (0.0027)
NDUB7_HUMAN	I7EQF7_HUMAN	HLA-A	I7EQF7	MHC class I antigen (Fragment)	32 kDa	0.18	97	62	35	0	16	15	25	0	0	15	0	-1.558885543	95% (0.019)
B3KUB9_HUMAN (+2)	SGPL1_HUMAN	SGPL1	O95470	Sphingosine-1-phosphate lyase 1 (S1PL) (SP-lyase 1) (SPL 1) (hSPL) (EC 4.1.2.27) (Sphingosine-1-phosphate aldolase)	64 kDa	0.065	76	50	28	25	3	7	14	1	3	12	12	-1.565331967	95% (0.030)
A8K5R6_HUMAN (+2)	CP2S1_HUMAN	CYP2S1	Q96SQ9	Cytochrome P450 2S1 (EC 1.14.14.1) (CYPIIS1)	56 kDa	0.18	45	29	16	17	6	5	1	3	4	1	8	-1.566507541	0% (0.080)
B4DRE6_HUMAN (+2)	Q53GA0_HUMAN		Q53GA0	Tumor protein D52-like 2 isoform e variant (Fragment)	22 kDa	0.057	23	15	8	1	5	8	1	0	0	2	6	-1.569205025	0% (0.17)
SYRM_HUMAN	F8W6G1_HUMAN	NRBP1	F8W6G1	Nuclear receptor-binding protein	61 kDa	0.042	23	15	8	1	7	5	2	3	3	0	2	-1.569205025	0% (0.17)
ATS1_HUMAN (+2)	S12A7_HUMAN	SLC12A7	Q9Y666	Solute carrier family 12 member 7 (Electroneutral potassium-chloride cotransporter 4) (K-Cl cotransporter 4)	119 kDa	0.026	12	8	4	8	0	0	0	1	0	2	1	-1.573752429	0% (0.26)
Q6AHZ7_HUMAN (+2)	F8VUJ3_HUMAN	POC1B-GALNT4	F8VUJ3	Protein POC1B-GALNT4	66 kDa	0.02	12	8	4	1	2	4	1	0	2	1	1	-1.573752429	0% (0.26)
SCRN1_HUMAN	B3KRR1_HUMAN		B3KRR1	cDNA FLJ34725 fis, clone MESAN2005958, highly similar to RNA-binding protein Luc7-like 2	40 kDa	0.063	12	8	4	0	2	2	3	0	2	0	2	-1.573752429	0% (0.26)
ABC3G_HUMAN (+3)	CRKL_HUMAN	CRKL	P46109	Crk-like protein	34 kDa	0.1	12	8	4	0	2	6	0	1	3	0	0	-1.573752429	0% (0.26)
CADH6_HUMAN	EPHA2_HUMAN	EPHA2	P29317	Ephrin type-A receptor 2 (EC 2.7.10.1) (Epithelial cell kinase) (Tyrosine-protein kinase receptor ECK)	108 kDa	0.042	12	8	4	6	1	1	0	4	0	0	0	-1.573752429	0% (0.26)
Q4LE48_HUMAN (+2)	GPAA1_HUMAN	GPAA1	O43292	Glycosylphosphatidylinositol anchor attachment 1 protein (GPI anchor attachment protein 1) (GAA1 protein homolog) (hGAA1)	68 kDa	0.055	12	8	4	2	2	0	4	1	0	2	1	-1.573752429	0% (0.26)
ATP9A_HUMAN (+1)	A6XND9_HUMAN		A6XND9	Beta-2-microglobulin	12 kDa	0.061	12	8	4	1	2	1	4	0	0	1	3	-1.573752429	0% (0.26)
COX20_HUMAN	Q8N355_HUMAN	IGL@	Q8N355	IGL@ protein	25 kDa	0.22	12	8	4	0	4	2	2	0	0	2	2	-1.573752429	0% (0.26)
B3KM57_HUMAN (+1)	TMM65_HUMAN	TMEM65	Q6PI78	Transmembrane protein 65	25 kDa	0.084	12	8	4	1	2	1	4	1	1	1	1	-1.573752429	0% (0.26)

B4DYY5_HUMAN (+3)	DHPR_HUMAN	QDPR	P09417	Dihydropteridine reductase (EC 1.5.1.34) (HDHPR) (Quinoid dihydropteridine reductase)	26 kDa	0.024	12	8	4	1	3	3	1	0	4	0	0	-1.573752429	0% (0.26)
EF1B_HUMAN	NUDC1_HUMAN	NUDCD1	Q96RS6	NudC domain-containing protein 1 (Chronic myelogenous leukemia tumor antigen 66) (Tumor antigen CML66)	67 kDa	0.12	12	8	4	0	2	2	4	0	4	0	0	-1.573752429	0% (0.26)
Q53GC2_HUMAN (+1)	B3KMR6_HUMAN		B3KMR6	cDNA FLJ12440 fis, clone NT2RM1000131, highly similar to Homo sapiens transmembrane protein 63A (TMEM63A), mRNA	92 kDa	0.24	12	8	4	7	0	1	0	1	1	1	1	-1.573752429	0% (0.26)
B4DVL2_HUMAN (+2)	B4DZA5_HUMAN		B4DZA5	Anoctamin	108 kDa	0.11	12	8	4	3	3	2	0	0	0	0	4	-1.573752429	0% (0.26)
LTOR5_HUMAN	E5KLL9_HUMAN		E5KLL9	Mitochondrial dynamin-like 120 kDa protein	118 kDa	0.37	117	75	42	26	20	14	15	15	6	15	9	-1.575231157	95% (0.0095)
B4DWC4_HUMAN (+2)	Q7Z3K9_HUMAN	DKFZp781L0540	Q7Z3K9	Putative uncharacterized protein DKFZp781L0540 (Fragment)	90 kDa	0.2	37	24	13	5	7	5	6	5	0	5	4	-1.582827421	0% (0.098)
DNLZ_HUMAN	SCRIB_HUMAN	SCRIB	Q14160	Protein scribble homolog (Scribble) (hScrib) (Protein LAP4)	175 kDa	0.051	36	24	13	8	4	4	7	7	2	3	1	-1.582827421	0% (0.098)
H9NTY4_HUMAN (+1)	RS11_HUMAN	RPS11	P62280	40S ribosomal protein S11	18 kDa	0.082	51	33	18	8	8	7	11	7	4	5	2	-1.589409787	0% (0.060)
B3KWP3_HUMAN (+2)	HS105_HUMAN	HSPH1	Q92598	Heat shock protein 105 kDa (Antigen NY-CO-25) (Heat shock 110 kDa protein)	97 kDa	0.1	104	67	37	6	21	22	18	13	13	8	3	-1.594220348	95% (0.012)
Q5S9Q5_HUMAN	PSB3_HUMAN	PSMB3	P49720	Proteasome subunit beta type-3 (EC 3.4.25.1) (Proteasome chain 13) (Proteasome component C10-II) (Proteasome theta chain)	23 kDa	0.075	40	26	14	4	5	8	9	1	4	7	2	-1.596204846	0% (0.084)
LYPL1_HUMAN	Q8N7G1_HUMAN		Q8N7G1	Purine nucleoside phosphorylase (EC 2.4.2.1) (Inosine-guanosine phosphorylase)	33 kDa	0.11	40	26	14	1	7	8	10	5	0	5	4	-1.596204846	0% (0.084)
B3KRS5_HUMAN (+2)	B4DRT4_HUMAN		B4DRT4	cDNA FLJ51535, highly similar to Phosphatidylethanolamine-binding protein 1	17 kDa	0.16	40	26	14	4	3	6	13	5	3	5	1	-1.596204846	0% (0.084)
IMA4_HUMAN (+1)	B4DV28_HUMAN		B4DV28	cDNA FLJ54170, highly similar to Cytosolic nonspecific dipeptidase	52 kDa	0.086	95	60	33	14	15	13	19	6	11	10	6	-1.59774902	95% (0.016)
B3KQ71_HUMAN (+4)	J3KP14_HUMAN	VPS13D	J3KP14	Vacuolar protein sorting-associated protein 13D	492 kDa	0.11	2	1	0	0	1	1	0	0	0	0	0	-1.607727417	0% (0.53)
SPX2_HUMAN	MISP_HUMAN	MISP	Q8IVT2	Mitotic interactor and substrate of PLK1 (Mitotic spindle positioning protein)	75 kDa	0.054	1	1	0	2	0	0	0	0	0	0	0	-1.607727417	0% (0.53)
Q8WY67_HUMAN (+1)	F263_HUMAN	PFKFB3	Q16875	6-phosphofructo-2-kinase/fructose-2,6-bisphosphatase 3 (6PF-2-K/Fru-2,6-P2ase 3) (PFK/FBPase 3) (6PF-2-K/Fru-2,6-P2ase brain/placenta-type isozyme) (Renal carcinoma antigen NY-REN-56) (iPFK-2) [Includes: 6-phosphofructo-2-kinase (EC 2.7.1.105); Fructose-2,6-bisphosphatase (EC 3.1.3.46)]	60 kDa	0.17	1	1	0	0	0	0	2	0	0	0	0	-1.607727417	0% (0.53)
CSN5_HUMAN	B2RDI6_HUMAN		B2RDI6	cDNA, FLJ96628, highly similar to Homo sapiens mitogen-activated protein kinase kinase kinase 11(MAP3K11), mRNA	93 kDa	0.11	2	1	0	1	0	1	0	0	0	0	0	-1.607727417	0% (0.53)
TBK1_HUMAN	A2VDI4_HUMAN	EFR3A	A2VDI4	EFR3A protein (Fragment)	76 kDa	0.064	2	1	0	1	1	0	0	0	0	0	0	-1.607727417	0% (0.53)
F5H5M9_HUMAN (+2)	EIF1_HUMAN	EIF1	P41567	Eukaryotic translation initiation factor 1 (eIF1) (A121) (Protein translation factor SUI1 homolog) (Sui1iso1)	13 kDa	0.02	2	1	0	0	0	0	1	0	0	0	0	-1.607727417	0% (0.53)

E9PS41_HUMAN (+1)	B2RDG1_HUMAN		B2RDG1	cDNA, FLJ96593	59 kDa	0.088	1	1	0	2	0	0	0	0	0	0	0	-1.607727417	0% (0.53)
B4DX69_HUMAN (+2)	C9JFX4_HUMAN	SIGIRR	C9JFX4	Single Ig IL-1-related receptor	55 kDa	0.081	1	1	0	2	0	0	0	0	0	0	0	-1.607727417	0% (0.53)
E9PHV4_HUMAN (+1)	Q59F44_HUMAN		Q59F44	Cytochrome b-5 isoform 1 variant (Fragment)	15 kDa	0.28	15	10	5	2	3	3	2	0	3	1	1	-1.607797698	0% (0.21)
B3KSG7_HUMAN (+2)	Q5LJA0_HUMAN	TROVE2	Q5LJA0	60 kDa SS-A/Ro ribonucleoprotein (TROVE domain family, member 2, isoform CRA_e)	59 kDa	0.042	15	10	5	1	2	3	5	1	3	1	0	-1.607797698	0% (0.21)
MECR_HUMAN	SMD2_HUMAN	SNRPD2	P62316	Small nuclear ribonucleoprotein Sm D2 (Sm-D2) (snRNP core protein D2)	14 kDa	0.067	15	10	5	1	2	1	6	1	1	2	1	-1.607797698	0% (0.21)
HSP72_HUMAN	A8K5B0_HUMAN		A8K5B0	cDNA FLJ75682, highly similar to Homo sapiens armadillo repeat containing, X-linked 3 (ARMCX3), transcript variant 1, mRNA	43 kDa	0.23	15	10	5	10	0	0	0	0	0	3	2	-1.607797698	0% (0.21)
SERP3_HUMAN	ETHE1_HUMAN	ETHE1	O95571	Persulfide dioxygenase ETHE1, mitochondrial (EC 1.13.11.18) (Ethylmalonic encephalopathy protein 1) (Hepatoma subtracted clone one protein) (Sulfur dioxygenase ETHE1)	28 kDa	0.047	29	19	10	6	4	6	4	1	2	3	4	-1.607867995	0% (0.12)
DHRSX_HUMAN	C9JVN9_HUMAN	L2HGDH	C9JVN9	L-2-hydroxyglutarate dehydrogenase, mitochondrial	48 kDa	0.16	31	19	10	5	5	5	4	8	2	1	1	-1.607867995	0% (0.12)
GATA_HUMAN	SORL_HUMAN	SORL1	Q92673	Sortilin-related receptor (Low-density lipoprotein receptor relative with 11 ligand-binding repeats) (LDLR relative with 11 ligand-binding repeats) (LR11) (SorLA-1) (Sorting protein-related receptor containing LDLR class A repeats) (SorLA)	248 kDa	0.12	29	19	10	12	2	5	0	3	1	4	2	-1.607867995	0% (0.12)
Q5LJA9_HUMAN	RRAS_HUMAN	RRAS	P10301	Ras-related protein R-Ras (p23)	23 kDa	0.098	43	28	15	9	7	6	6	3	3	6	4	-1.607938308	0% (0.073)
NRAM1_HUMAN	HUWE1_HUMAN	HUWE1	Q7Z6Z7	E3 ubiquitin-protein ligase HUWE1 (EC 6.3.2.-) (ARF-binding protein 1) (ARF-BP1) (HECT, UBA and WWE domain-containing protein 1) (Homologous to E6AP carboxyl terminus homologous protein 9) (HectH9) (Large structure of UREB1) (LASU1) (Mcl-1 ubiquitin ligase E3) (Mule) (Upstream regulatory element-binding protein 1) (URE-B1) (URE-binding protein 1)	482 kDa	0.051	74	48	26	0	16	15	15	5	15	6	1	-1.614654161	95% (0.025)
TB182_HUMAN	NPC1_HUMAN	NPC1	O15118	Niemann-Pick C1 protein	142 kDa	0.018	46	30	16	17	7	6	0	2	1	2	11	-1.618313714	0% (0.063)
NUDT9_HUMAN (+1)	HTAI2_HUMAN	HTATIP2	Q9BUP3	Oxidoreductase HTATIP2 (EC 1.1.1.-) (30 kDa HIV-1 TAT-interacting protein) (HIV-1 TAT-interactive protein 2)	27 kDa	0.074	32	21	11	6	6	5	4	2	3	2	4	-1.622470191	0% (0.10)
SPG21_HUMAN	Q53FN0_HUMAN		Q53FN0	Splicing factor, arginine/serine-rich 2 variant (Fragment)	26 kDa	0.14	32	21	11	2	8	5	6	2	1	4	4	-1.622470191	0% (0.10)
B2R514_HUMAN (+2)	Q6ZQN2_HUMAN		Q6ZQN2	cDNA FLJ46846 fis, clone UTERU3004635, moderately similar to Neuroblast differentiation associated protein AHNAK (Fragment)	181 kDa	0.15	228	147	80	50	45	35	19	34	0	0	22	-1.630897269	95% (0.00014)
ACOC_HUMAN	RAD50_HUMAN	RAD50	Q92878	DNA repair protein RAD50 (hRAD50) (EC 3.6.-.-)	154 kDa	0.028	18	12	6	1	1	5	5	1	0	3	2	-1.632456745	0% (0.18)
B4DMR3_HUMAN (+1)	VAC14_HUMAN	VAC14	Q08AM6	Protein VAC14 homolog (Tax1-binding protein 2)	88 kDa	0.14	19	12	6	2	5	5	0	0	4	0	2	-1.632456745	0% (0.18)

B7Z8W3_HUMAN (+1)	Q6LEU8_HUMAN	SPINT2	Q6LEU8	SPINT2 protein (Fragment)	28 kDa	0.024	18	12	6	1	2	3	6	1	0	3	2	-1.632456745	0% (0.18)
AK1C1_HUMAN	GALE_HUMAN	GALE	Q14376	UDP-glucose 4-epimerase (EC 5.1.3.2) (Galactowaldenase) (UDP-galactose 4-epimerase)	38 kDa	0.14	18	12	6	1	0	0	11	2	4	0	0	-1.632456745	0% (0.18)
GLE1_HUMAN	B2R983_HUMAN		B2R983	cDNA, FLJ94267, highly similar to Homo sapiens glutathione S-transferase omega 1 (GSTO1), mRNA	28 kDa	0.063	18	12	6	2	5	2	3	0	4	1	2	-1.632456745	0% (0.18)
G3V207_HUMAN (+1)	PYRD_HUMAN	DHODH	Q02127	Dihydroorotate dehydrogenase (quinone), mitochondrial (DHOdehase) (EC 1.3.5.2) (Dihydroorotate oxidase)	43 kDa	0.12	18	12	6	3	4	3	2	2	1	1	2	-1.632456745	0% (0.18)
H7C417_HUMAN	J3KT73_HUMAN	RPL38	J3KT73	60S ribosomal protein L38	8 kDa	0.074	35	23	12	4	4	4	11	3	4	1	4	-1.634871334	0% (0.088)
FKB1A_HUMAN	Q53HQ0_HUMAN		Q53HQ0	Flotillin 1 variant (Fragment)	47 kDa	0.25	109	71	38	31	12	11	17	4	11	11	12	-1.644717867	95% (0.0066)
D39U1_HUMAN	D3DUE6_HUMAN	N-PAC	D3DUE6	Cytokine-like nuclear factor n-pac, isoform CRA_c	61 kDa	0.11	21	14	7	2	4	2	6	0	0	5	2	-1.651142741	0% (0.15)
PEX16_HUMAN	E7EQT4_HUMAN	ACIN1	E7EQT4	Apoptotic chromatin condensation inducer in the nucleus	147 kDa	0.068	21	14	7	5	3	2	4	2	2	1	2	-1.651142741	0% (0.15)
B4DNC0_HUMAN (+1)	TMOD1_HUMAN	TMOD1	P28289	Tropomodulin-1 (Erythrocyte tropomodulin) (E-Tmod)	41 kDa	0.15	21	14	7	3	7	5	0	4	0	2	1	-1.651142741	0% (0.15)
E9PBR6_HUMAN (+1)	LTOR3_HUMAN	LAMTOR3	Q9UHA4	Ragulator complex protein LAMTOR3 (Late endosomal/lysosomal adaptor and MAPK and MTOR activator 3) (MEK-binding partner 1) (Mp1) (Mitogen-activated protein kinase kinase 1-interacting protein 1) (Mitogen-activated protein kinase scaffold protein 1)	14 kDa	0.044	21	14	7	3	7	2	2	1	1	1	4	-1.651142741	0% (0.15)
RET1_HUMAN	B3KWW5_HUMAN		B3KWW5	Galectin	36 kDa	0.37	21	14	7	4	2	2	6	0	0	2	5	-1.651142741	0% (0.15)
ANFY1_HUMAN	ALG11_HUMAN	ALG11	Q2TAA5	GDP-Man:Man(3)GlcNAc(2)-PP-Dol alpha-1,2-mannosyltransferase (EC 2.4.1.131) (Asparagine-linked glycosylation protein 11 homolog) (Glycolipid 2-alpha-mannosyltransferase)	56 kDa	0.015	21	14	7	6	5	2	1	1	2	2	2	-1.651142741	0% (0.15)
A8K9V9_HUMAN (+1)	B2R7T8_HUMAN		B2R7T8	cDNA, FLJ93598, highly similar to Homo sapiens capping protein (actin filament) muscle Z-line, beta (CAPZB), mRNA	31 kDa	0.1	42	27	14	3	5	9	10	10	2	2	0	-1.654802439	0% (0.066)
Q0P5W4_HUMAN	ANXA2_HUMAN	ANXA2	P07355	Annexin A2 (Annexin II) (Annexin-2) (Calpactin I heavy chain) (Calpactin-1 heavy chain) (Chromobindin-8) (Lipocortin II) (Placental anticoagulant protein IV) (PAP-IV) (Protein I) (p36)	39 kDa	0.082	819	534	288	248	97	89	101	67	50	78	94	-1.657313175	95% (< 0.00010)
ATG3_HUMAN	H2AW_HUMAN	H2AFY2	Q9P0M6	Core histone macro-H2A.2 (Histone macroH2A2) (mH2A2)	40 kDa	0.12	103	68	36	32	9	9	19	9	7	10	10	-1.661049089	95% (0.0068)
B4E0E0_HUMAN (+1)	B3KMB8_HUMAN		B3KMB8	cDNA FLJ10654 fis, clone NT2RP2005901, highly similar to FAST kinase domains-containing protein 2	81 kDa	0.045	24	16	8	3	7	3	3	1	4	4	0	-1.665792894	0% (0.13)
INT3_HUMAN	PIGT_HUMAN	PIGT	Q969N2	GPI transamidase component PIG-T (Phosphatidylinositol-glycan biosynthesis class T protein)	66 kDa	0.031	24	16	8	4	4	3	5	3	0	3	2	-1.665792894	0% (0.13)
ABCA1_HUMAN (+2)	PPA5_HUMAN	ACP5	P13686	Tartrate-resistant acid phosphatase type 5 (TR-AP) (EC 3.1.3.2) (Tartrate-resistant acid ATPase) (TrATPase) (Type 5 acid phosphatase)	37 kDa	0.01	24	16	8	1	6	7	2	0	2	0	6	-1.665792894	0% (0.13)

ARHG2_HUMAN	RL35_HUMAN	RPL35	P42766	60S ribosomal protein L35	15 kDa	0.023	24	16	8	3	4	3	6	2	2	2	2	-1.665792894	0% (0.13)
2B1G_HUMAN	A8MXB9_HUMAN	SUMF2	A8MXB9	Sulfatase-modifying factor 2	34 kDa	0.26	24	16	8	2	3	5	6	1	2	3	2	-1.665792894	0% (0.13)
Q8WVC2_HUMAN (+2)	TRA2A_HUMAN	TRA2A	Q13595	Transformer-2 protein homolog alpha (TRA-2 alpha) (TRA2-alpha) (Transformer-2 protein homolog A)	33 kDa	0.41	24	16	8	4	3	4	5	2	3	2	1	-1.665792894	0% (0.13)
Q5U0F7_HUMAN	B4DZ88_HUMAN	KTN1	B4DZ88	KTN1 protein (cDNA FLJ61494, highly similar to Kinectin)	153 kDa	0.077	46	31	16	4	8	14	5	4	0	12	0	-1.670121219	95% (0.049)
RPRD2_HUMAN	E7EW20_HUMAN	MYO6	E7EW20	Unconventional myosin-VI	149 kDa	0.037	49	33	17	17	5	6	4	2	5	6	4	-1.676524856	95% (0.043)
SUSD2_HUMAN	PSA3_HUMAN	PSMA3	P25788	Proteasome subunit alpha type-3 (EC 3.4.25.1) (Macropain subunit C8) (Multicatalytic endopeptidase complex subunit C8) (Proteasome component C8)	28 kDa	0.064	29	18	9	1	4	5	8	3	1	4	1	-1.677588425	0% (0.11)
PPIG_HUMAN	TCRG1_HUMAN	TCERG1	O14776	Transcription elongation regulator 1 (TATA box-binding protein-associated factor 2S) (Transcription factor CA150)	124 kDa	0.052	4	3	1	0	1	0	2	0	0	1	0	-1.687140448	0% (0.36)
ANR22_HUMAN	A8K5X9_HUMAN		A8K5X9	cDNA FLJ77989, highly similar to Homo sapiens RNA binding motif protein 19, mRNA (cDNA, FLJ95445, highly similar to Homo sapiens RNA binding motif protein 19 (RBM19), mRNA)	107 kDa	0.15	4	3	1	0	0	0	3	0	0	1	0	-1.687140448	0% (0.36)
MDC1_HUMAN	B4E1A5_HUMAN		B4E1A5	cDNA FLJ59652, highly similar to Thyroid hormone receptor-associated protein complex 100 kDa component	100 kDa	0.02	4	3	1	1	1	1	0	0	0	0	1	-1.687140448	0% (0.36)
DOCK2_HUMAN	COIA1_HUMAN	COL18A1	P39060	Collagen alpha-1(XVIII) chain [Cleaved into: Endostatin]	178 kDa	0.015	4	3	1	2	1	0	0	0	1	0	0	-1.687140448	0% (0.36)
A8K245_HUMAN (+1)	B4DGX2_HUMAN	PIP4K2A	B4DGX2	Phosphatidylinositol 5-phosphate 4-kinase type-2 alpha (cDNA FLJ53867, highly similar to Phosphatidylinositol-4-phosphate 5-kinase type-2 alpha (EC 2.7.1.68))	40 kDa	0.081	4	3	1	0	2	0	1	0	0	0	1	-1.687140448	0% (0.36)
C9JEJ2_HUMAN (+1)	P3C2A_HUMAN	PIK3C2A	O00443	Phosphatidylinositol 4-phosphate 3-kinase C2 domain-containing subunit alpha (PI3K-C2-alpha) (PtdIns-3-kinase C2 subunit alpha) (EC 2.7.1.154) (Phosphoinositide 3-kinase-C2-alpha)	191 kDa	0.13	5	3	1	0	1	2	0	0	0	1	0	-1.687140448	0% (0.36)
F5H7V7_HUMAN (+1)	B2RAU5_HUMAN		B2RAU5	Sorting nexin	67 kDa	0.16	4	3	1	2	0	1	0	0	1	0	0	-1.687140448	0% (0.36)
ACSA_HUMAN (+3)	DHRSX_HUMAN	DHRSX	Q8N5I4	Dehydrogenase/reductase SDR family member on chromosome X (EC 1.1.-.-) (DHRSXY)	36 kDa	0.07	4	3	1	3	0	0	0	0	0	0	1	-1.687140448	0% (0.36)
A8K6U0_HUMAN (+2)	ANFY1_HUMAN	ANKFY1	Q9P2R3	Ankyrin repeat and FYVE domain-containing protein 1 (Ankyrin repeats hooked to a zinc finger motif)	128 kDa	0.056	5	3	1	0	1	1	1	0	1	0	0	-1.687140448	0% (0.36)
HPDL_HUMAN	RIC8A_HUMAN	RIC8A	Q9NPQ8	Synembryn-A (Protein Ric-8A)	60 kDa	0.049	4	3	1	0	1	0	2	0	1	0	0	-1.687140448	0% (0.36)
B7Z2R7_HUMAN	Q12915_HUMAN	Ibd1	Q12915	Ibd1 protein (Fragment)	22 kDa	0.082	3	3	1	0	0	1	1	0	1	0	0	-1.687140448	0% (0.36)
ADDG_HUMAN (+1)	FARP1_HUMAN	FARP1	Q9Y4F1	FERM, RhoGEF and pleckstrin domain-containing protein 1 (Chondrocyte-derived ezrin-like protein) (Pleckstrin homology domain-containing family C member	119 kDa	0.042	4	3	1	0	2	1	0	0	0	1	0	-1.687140448	0% (0.36)

				2) (PH domain-containing family C member 2)															
CUL3_HUMAN (+1)	PI42C_HUMAN	PIP4K2C	Q8TBX8	Phosphatidylinositol 5-phosphate 4-kinase type-2 gamma (EC 2.7.1.149) (Phosphatidylinositol 5-phosphate 4-kinase type II gamma) (PI(5)P 4-kinase type II gamma) (PIP4KII-gamma)	47 kDa	0.049	4	3	1	0	2	0	1	0	1	0	0	-1.687140448	0% (0.36)
6PGL_HUMAN	CP4X1_HUMAN	CYP4X1	Q8N118	Cytochrome P450 4X1 (EC 1.14.14.1) (CYPIVX1)	59 kDa	0.17	4	3	1	0	3	0	0	0	1	0	0	-1.687140448	0% (0.36)
MTER1_HUMAN	AGM1_HUMAN	PGM3	O95394	Phosphoacetylglucosamine mutase (PAGM) (EC 5.4.2.3) (Acetylglucosamine phosphomutase) (N-acetylglucosamine-phosphate mutase) (Phosphoglucomutase-3) (PGM 3)	60 kDa	0.053	5	3	1	1	1	0	2	0	1	0	0	-1.687140448	0% (0.36)
SNX27_HUMAN	LRC8C_HUMAN	LRRC8C	Q8TDW0	Leucine-rich repeat-containing protein 8C (Factor for adipocyte differentiation 158)	92 kDa	0.041	4	3	1	0	2	1	0	0	0	1	0	-1.687140448	0% (0.36)
ECSIT_HUMAN	J3QRV5_HUMAN	LLGL2	J3QRV5	Lethal(2) giant larvae protein homolog 2	113 kDa	0.074	4	3	1	1	0	0	2	0	1	0	0	-1.687140448	0% (0.36)
ATAD5_HUMAN	CC127_HUMAN	CCDC127	Q96BQ5	Coiled-coil domain-containing protein 127	31 kDa	0.014	4	3	1	2	0	0	1	0	0	1	0	-1.687140448	0% (0.36)
E9PIN3_HUMAN	MAN1_HUMAN	LEMD3	Q9Y2U8	Inner nuclear membrane protein Man1 (LEM domain-containing protein 3)	100 kDa	0.068	4	3	1	2	0	1	0	0	0	1	0	-1.687140448	0% (0.36)
SYNE3_HUMAN	B2RAW1_HUMAN		B2RAW1	cDNA, FLJ95155, highly similar to Homo sapiens Bruton agammaglobulinemia tyrosine kinase (BTK), mRNA	76 kDa	0.027	4	3	1	0	1	1	1	0	1	0	0	-1.687140448	0% (0.36)
B2RDR4_HUMAN	GNPI1_HUMAN	GNPDA1	P46926	Glucosamine-6-phosphate isomerase 1 (EC 3.5.99.6) (Glucosamine-6-phosphate deaminase 1) (GNPDA 1) (GlcN6P deaminase 1) (Oscillin)	33 kDa	0.12	4	3	1	0	2	1	0	1	0	0	0	-1.687140448	0% (0.36)
FUBP3_HUMAN	F8W8C2_HUMAN	VEZT	F8W8C2	Vezatin (Fragment)	82 kDa	0.093	4	3	1	3	0	0	0	0	0	1	0	-1.687140448	0% (0.36)
AT8B1_HUMAN	TRM6_HUMAN	TRMT6	Q9UJA5	tRNA (adenine(58)-N(1))-methyltransferase non-catalytic subunit TRM6 (tRNA(m1A58)-methyltransferase subunit TRM6) (tRNA(m1A58)MTase subunit TRM6)	56 kDa	0.026	4	3	1	0	0	0	3	1	0	0	0	-1.687140448	0% (0.36)
B7Z5H3_HUMAN	B4DFR2_HUMAN	DYNLRB1	B4DFR2	Dynein light chain roadblock-type 1 (cDNA FLJ59194, moderately similar to Dynein light chain 2A, cytoplasmic)	13 kDa	0.16	4	3	1	0	0	2	1	0	1	0	0	-1.687140448	0% (0.36)
SH3L1_HUMAN	EDC3_HUMAN	EDC3	Q96F86	Enhancer of mRNA-decapping protein 3 (LSM16 homolog) (YjeF N-terminal domain-containing protein 2) (YjeF_N2) (hYjeF_N2) (YjeF domain-containing protein 1)	56 kDa	0.26	4	3	1	1	2	0	1	0	1	0	0	-1.687140448	0% (0.36)
COQ6_HUMAN	SYPM_HUMAN	PARS2	Q7L3T8	Probable proline--tRNA ligase, mitochondrial (EC 6.1.1.15) (Prolyl-tRNA synthetase) (ProRS)	53 kDa	0.079	4	3	1	0	1	0	2	1	0	0	0	-1.687140448	0% (0.36)
B7Z7F0_HUMAN	GDPD1_HUMAN	GDPD1	Q8N9F7	Glycerophosphodiester phosphodiesterase domain-containing protein 1 (EC 3.1.-.-) (Glycerophosphodiester phosphodiesterase 4)	36 kDa	0.063	4	3	1	2	0	0	1	0	1	0	0	-1.687140448	0% (0.36)
G5E9A6_HUMAN (+2)	PRRC1_HUMAN	PRRC1	Q96M27	Protein PRRC1 (Proline-rich and coiled-coil-containing protein 1)	47 kDa	0.055	4	3	1	0	0	2	1	0	0	1	0	-1.687140448	0% (0.36)
ARL3_HUMAN	B4DDH9_HUMAN		B4DDH9	cDNA FLJ53820, highly similar to U4/U6.U5 tri-snRNP-associated protein 1	73 kDa	0.2	4	3	1	0	0	1	2	0	0	1	0	-1.687140448	0% (0.36)

CDR2L_HUMAN	C9JNZ0_HUMAN	SEC22A	C9JNZ0	Vesicle-trafficking protein SEC22a (Fragment)	33 kDa	0.054	4	3	1	2	1	0	0	0	0	0	1	-1.687140448	0% (0.36)
TBD2A_HUMAN	A8K6D2_HUMAN		A8K6D2	cDNA FLJ76620, highly similar to Homo sapiens C2f protein (C2F), mRNA	27 kDa	0.029	4	3	1	0	0	0	3	0	1	0	0	-1.687140448	0% (0.36)
SLMAP_HUMAN	CRK_HUMAN	CRK	P46108	Adapter molecule crk (Proto-oncogene c-Crk) (p38)	34 kDa	0.053	4	3	1	0	0	0	3	0	1	0	0	-1.687140448	0% (0.36)
AUHM_HUMAN	PMVK_HUMAN	PMVK	Q15126	Phosphomevalonate kinase (PMKase) (hPMK) (EC 2.7.4.2)	22 kDa	0.062	4	3	1	0	0	0	3	0	1	0	0	-1.687140448	0% (0.36)
A8K9D2_HUMAN (+1)	SIAT1_HUMAN	ST6GAL1	P15907	Beta-galactoside alpha-2,6-sialyltransferase 1 (Alpha 2,6-ST 1) (EC 2.4.99.1) (B-cell antigen CD75) (CMP-N-acetylneuraminate-beta-galactosamide-alpha-2,6-sialyltransferase 1) (ST6Gal I) (ST6GalI) (Sialyltransferase 1)	47 kDa	0.12	4	3	1	3	0	0	0	0	0	1	0	-1.687140448	0% (0.36)
C9IZG4_HUMAN (+1)	I3L3B4_HUMAN		I3L3B4	Uncharacterized protein (Fragment)	11 kDa	0.21	4	3	1	0	0	0	3	0	0	1	0	-1.687140448	0% (0.36)
AP1S1_HUMAN (+1)	A8K3E4_HUMAN		A8K3E4	cDNA FLJ78367, highly similar to Homo sapiens fibrinogen, A alpha polypeptide (FGA), transcriptvariant alpha, mRNA	70 kDa	0.1	4	3	1	1	0	0	2	0	0	0	1	-1.687140448	0% (0.36)
RT15_HUMAN	M0QXN5_HUMAN	NUP62	M0QXN5	Nuclear pore glycoprotein p62	46 kDa	0.082	4	3	1	1	0	0	2	0	0	1	0	-1.687140448	0% (0.36)
C9JAF7_HUMAN (+1)	THIO_HUMAN	TXN	P10599	Thioredoxin (Trx) (ATL-derived factor) (ADF) (Surface-associated sulphydryl protein) (SASP)	12 kDa	0.16	30	20	10	2	6	7	6	4	2	2	2	-1.687290567	0% (0.092)
J3KN55_HUMAN (+1)	Q53GD1_HUMAN		Q53GD1	Guanine nucleotide-binding protein subunit gamma (Fragment)	8 kDa	0.16	30	20	10	5	4	4	7	1	3	3	3	-1.687290567	0% (0.092)
B4DPG9_HUMAN (+2)	PSME1_HUMAN	PSME1	Q06323	Proteasome activator complex subunit 1 (11S regulator complex subunit alpha) (REG-alpha) (Activator of multicatalytic protease subunit 1) (Interferon gamma up-regulated I-5111 protein) (IGUP I-5111) (Proteasome activator 28 subunit alpha) (PA28a) (PA28alpha)	29 kDa	0.06	35	24	12	0	5	8	11	3	3	4	2	-1.702310586	0% (0.068)
PLCC_HUMAN	F5H569_HUMAN	ATP6V0A1	F5H569	V-type proton ATPase 116 kDa subunit a isoform 1	96 kDa	0.061	36	24	12	12	4	7	1	1	3	0	8	-1.702310586	0% (0.068)
F210A_HUMAN (+1)	A1L172_HUMAN	ACOT1	A1L172	Acyl-CoA thioesterase 1	46 kDa	0.1	36	24	12	3	13	6	2	1	1	6	4	-1.702310586	0% (0.068)
ITB8_HUMAN (+1)	B2R5U1_HUMAN		B2R5U1	cDNA, FLJ92620, highly similar to Homo sapiens staphylococcal nuclease domain containing 1 (SND1),mRNA	100 kDa	0.073	103	68	35	7	23	20	18	0	14	18	4	-1.706895698	95% (0.0048)
ABHD6_HUMAN	D6RER5_HUMAN	40787	D6RER5	Septin-11	50 kDa	0.089	41	26	13	4	6	12	3	2	3	4	4	-1.708243798	0% (0.058)
F5GYJ5_HUMAN (+1)	B3KWV6_HUMAN		B3KWV6	cDNA FLJ43948 fis, clone TESTI4014924, highly similar to Homo sapiens cytoplasmic FMR1 interacting protein 1 (CYFIP1), transcript variant 1, mRNA	146 kDa	0.091	75	49	25	9	15	20	6	5	7	7	6	-1.710245194	95% (0.014)
B4DP38_HUMAN (+1)	B9EK46_HUMAN	CGN	B9EK46	Cingulin	137 kDa	0.079	6	5	2	2	1	0	2	2	0	0	0	-1.717696357	0% (0.28)
Q86X36_HUMAN	B3KN79_HUMAN		B3KN79	cDNA FLJ13894 fis, clone THYRO1001671, highly similar to 59 kDa 2'-5'-oligoadenylate synthetase-like protein	59 kDa	0.03	7	5	2	0	0	0	5	0	2	0	0	-1.717696357	0% (0.28)
H7C525_HUMAN	H0Y8R1_HUMAN	GRSF1	H0Y8R1	G-rich sequence factor 1 (Fragment)	47 kDa	0.17	8	5	2	1	3	1	0	1	0	3	0	-1.717696357	0% (0.28)

B9A062_HUMAN (+1)	ACAP2_HUMAN	ACAP2	Q15057	Arf-GAP with coiled-coil, ANK repeat and PH domain-containing protein 2 (Centaurin-beta-2) (Cnt-b2)	88 kDa	0.16	7	5	2	0	1	1	3	0	2	0	0	-1.717696357	0% (0.28)
F6IQP1_HUMAN (+1)	B5MDE0_HUMAN	RFT1	B5MDE0	Protein RFT1 homolog (RFT1 homolog (S. cerevisiae), isoform CRA_b)	56 kDa	0.32	7	5	2	1	0	1	3	0	1	0	1	-1.717696357	0% (0.28)
WDFY1_HUMAN	B4DUC8_HUMAN	MTAP	B4DUC8	S-methyl-5'-thioadenosine phosphorylase (cDNA FLJ59758, highly similar to S-methyl-5-thioadenosine phosphorylase (EC 2.4.2.28))	33 kDa	0.071	7	5	2	0	0	2	4	1	1	0	0	-1.717696357	0% (0.28)
BAX_HUMAN	NCF2_HUMAN	NCF2	P19878	Neutrophil cytosol factor 2 (NCF-2) (67 kDa neutrophil oxidase factor) (NADPH oxidase activator 2) (Neutrophil NADPH oxidase factor 2) (p67-phox)	60 kDa	0.26	7	5	2	0	2	2	1	0	1	1	0	-1.717696357	0% (0.28)
B7Z322_HUMAN (+3)	GAR1_HUMAN	GAR1	Q9NY12	H/ACA ribonucleoprotein complex subunit 1 (Nucleolar protein family A member 1) (snoRNP protein GAR1)	22 kDa	0.096	7	5	2	1	0	1	3	0	0	1	1	-1.717696357	0% (0.28)
J3KPT4_HUMAN (+2)	E9PE17_HUMAN	MRPS17	E9PE17	28S ribosomal protein S17, mitochondrial (Fragment)	14 kDa	0.066	7	5	2	1	1	1	2	2	0	0	0	-1.717696357	0% (0.28)
NUDC_HUMAN	E9PGT3_HUMAN	RPS6KA1	E9PGT3	Ribosomal protein S6 kinase (EC 2.7.11.1)	81 kDa	0.082	7	5	2	0	2	2	0	0	0	0	0	-1.717696357	0% (0.28)
CHERP_HUMAN (+1)	C163A_HUMAN	CD163	Q86VB7	Scavenger receptor cysteine-rich type 1 protein M130 (Hemoglobin scavenger receptor) (CD antigen CD163) [Cleaved into: Soluble CD163 (sCD163)]	125 kDa	0.032	48	32	16	6	12	6	8	0	3	9	5	-1.721930055	95% (0.038)
GATM_HUMAN	NEUA_HUMAN	CMAS	Q8NFW8	N-acylneuraminate cytidylyltransferase (EC 2.7.7.43) (CMP-N-acetylneuraminic acid synthase) (CMP-NeuNAc synthase)	48 kDa	0.064	48	32	16	6	10	10	6	0	7	8	1	-1.721930055	95% (0.038)
M0QX35_HUMAN	HBA_HUMAN	HBA1;	P69905	Hemoglobin subunit alpha (Alpha-globin) (Hemoglobin alpha chain)	15 kDa	0.052	248	163	84	13	56	52	42	12	19	23	30	-1.722386844	95% (< 0.00010)
B4DVY1_HUMAN	FLNB_HUMAN	FLNB	O75369	Filamin-B (FLN-B) (ABP-278) (ABP-280 homolog) (Actin-binding-like protein) (Beta-filamin) (Filamin homolog 1) (Fh1) (Filamin-3) (Thyroid autoantigen) (Truncated actin-binding protein) (Truncated ABP)	278 kDa	0.1	265	175	90	42	55	63	17	31	17	28	14	-1.726813894	95% (< 0.00010)
B4DND4_HUMAN (+1)	I7B0X5_HUMAN	HLA-B	I7B0X5	MHC class I antigen (Fragment)	32 kDa	0.11	101	67	34	0	20	17	18	5	0	0	0	-1.729973366	95% (0.0043)
B3KS61_HUMAN (+1)	MYO1G_HUMAN	MYO1G	B0I1T2	Unconventional myosin-Ig [Cleaved into: Minor histocompatibility antigen HA-2 (mHag HA-2)]	116 kDa	0.23	10	7	3	0	3	3	1	0	0	2	1	-1.733882526	0% (0.22)
AK1BA_HUMAN	E5RJR5_HUMAN	SKP1	E5RJR5	S-phase kinase-associated protein 1	19 kDa	0.18	10	7	3	1	2	1	3	2	0	2	0	-1.733882526	0% (0.22)
D3DQ48_HUMAN (+3)	B4DP77_HUMAN		B4DP77	cDNA FLJ57413, highly similar to Mitochondrial 28S ribosomal protein S10	19 kDa	0.083	11	7	3	3	1	0	3	2	1	0	1	-1.733882526	0% (0.22)
TPM1_HUMAN	A8K2S7_HUMAN		A8K2S7	cDNA FLJ77865	39 kDa	0.17	10	7	3	7	0	0	0	1	0	2	0	-1.733882526	0% (0.22)
B4DEA3_HUMAN (+3)	UFSP2_HUMAN	UFSP2	Q9NUQ7	Ufm1-specific protease 2 (UfSP2) (EC 3.4.22.-)	53 kDa	0.06	10	7	3	6	0	0	1	0	0	0	3	-1.733882526	0% (0.22)
B2RCJ6_HUMAN (+1)	B7Z1C5_HUMAN		B7Z1C5	cDNA FLJ51785, highly similar to Glutathione synthetase (EC 6.3.2.3)	45 kDa	0.02	10	7	3	0	2	4	1	1	1	1	0	-1.733882526	0% (0.22)
UB2L6_HUMAN	B2R4V4_HUMAN		B2R4V4	cDNA, FLJ92232, highly similar to Homo sapiens barrier to autointegration factor 1 (BANF1), mRNA	10 kDa	0.1	10	7	3	1	1	1	4	0	3	0	0	-1.733882526	0% (0.22)

41_HUMAN (+1)	ACPH_HUMAN	APEH	P13798	Acylamino-acid-releasing enzyme (AARE) (EC 3.4.19.1) (Acyl-peptide hydrolase) (APH) (Acylaminoacyl-peptidase) (Oxidized protein hydrolase) (OPH)	81 kDa	0.053	10	7	3	0	2	3	2	2	0	1	0	-1.733882526	0% (0.22)
H0Y3C5_HUMAN (+1)	RPR1A_HUMAN	RPRD1A	Q96P16	Regulation of nuclear pre-mRNA domain-containing protein 1A (Cyclin-dependent kinase inhibitor 2B-related protein) (p15INK4B-related protein)	36 kDa	0.14	10	7	3	0	2	0	0	0	0	3	0	-1.733882526	0% (0.22)
RAI14_HUMAN	PLSL_HUMAN	LCP1	P13796	Plastin-2 (L-plastin) (LC64P) (Lymphocyte cytosolic protein 1) (LCP-1)	70 kDa	0.023	168	112	57	12	46	46	7	1	7	27	21	-1.737583817	95% (0.00030)
E9PIM6_HUMAN (+3)	RCC1_HUMAN	RCC1	P18754	Regulator of chromosome condensation (Cell cycle regulatory protein) (Chromosome condensation protein 1)	45 kDa	0.25	69	46	23	15	6	5	20	11	3	7	2	-1.740754151	95% (0.014)
B2R6G5_HUMAN (+1)	CYBP_HUMAN	CACYBP	Q9HB71	Calcyclin-binding protein (CacyBP) (hCacyBP) (S100A6-binding protein) (Siah-interacting protein)	26 kDa	0.044	13	9	4	0	2	2	4	2	2	0	0	-1.743910235	0% (0.18)
GNAI1_HUMAN	Q5VWC4_HUMAN	PSMD4	Q5VWC4	26S proteasome non-ATPase regulatory subunit 4 (Proteasome (Prosome, macropain) 26S subunit, non-ATPase, 4, isoform CRA_b)	41 kDa	0.3	13	9	4	0	3	3	3	1	1	2	0	-1.743910235	0% (0.18)
E9PDY6_HUMAN (+1)	B3KPZ2_HUMAN		B3KPZ2	cDNA FLJ32487 fis, clone SKNSH1000002, highly similar to Prostaglandin E synthase 2 (EC 5.3.99.3)	42 kDa	0.012	13	9	4	5	1	1	2	0	1	2	1	-1.743910235	0% (0.18)
A8K3R2_HUMAN (+2)	PTN12_HUMAN	PTPN12	Q05209	Tyrosine-protein phosphatase non-receptor type 12 (EC 3.1.3.48) (PTP-PEST) (Protein-tyrosine phosphatase G1) (PTPG1)	88 kDa	0.054	13	9	4	1	5	3	0	0	2	1	1	-1.743910235	0% (0.18)
J3KNH7_HUMAN (+1)	PSB8_HUMAN	PSMB8	P28062	Proteasome subunit beta type-8 (EC 3.4.25.1) (Low molecular mass protein 7) (Macropain subunit C13) (Multicatalytic endopeptidase complex subunit C13) (Proteasome component C13) (Proteasome subunit beta-5i) (Really interesting new gene 10 protein)	30 kDa	0.049	12	9	4	0	4	1	4	0	0	3	1	-1.743910235	0% (0.18)
AFAP1_HUMAN	LEG1_HUMAN	LGALS1	P09382	Galectin-1 (Gal-1) (14 kDa laminin-binding protein) (HLBP14) (14 kDa lectin) (Beta-galactoside-binding lectin L-14-I) (Galaptin) (HBL) (HPL) (Lactose-binding lectin 1) (Lectin galactoside-binding soluble 1) (Putative MAPK-activating protein PM12) (S-Lac lectin 1)	15 kDa	0.034	75	50	25	7	15	22	6	2	9	7	7	-1.744302349	95% (0.011)
B4DYN8_HUMAN (+1)	B4DIT7_HUMAN	TGM2	B4DIT7	Protein-glutamine gamma-glutamyltransferase 2 (cDNA FLJ58187, highly similar to Protein-glutamine gamma-glutamyltransferase 2(EC 2.3.2.13))	69 kDa	0.13	209	140	71	38	41	37	24	8	18	16	30	-1.747523533	95% (< 0.00010)
F8WAB8_HUMAN	A6NG51_HUMAN	SPTAN1	A6NG51	Spectrin alpha chain, non-erythrocytic 1	285 kDa	0.11	227	150	76	49	36	36	29	18	6	32	20	-1.750225294	95% (< 0.00010)
B2R7N6_HUMAN (+2)	GRB2_HUMAN	GRB2	P62993	Growth factor receptor-bound protein 2 (Adapter protein GRB2) (Protein Ash) (SH2/SH3 adapter GRB2)	25 kDa	0.053	16	11	5	0	3	3	4	0	2	1	2	-1.750735545	0% (0.15)
NADC_HUMAN	KDEL2_HUMAN	KDELC2	Q7Z4H8	KDEL motif-containing protein 2	59 kDa	0.088	16	11	5	2	5	2	3	0	0	3	2	-1.750735545	0% (0.15)
B4E381_HUMAN (+3)	SIAS_HUMAN	NANS	Q9NR45	Sialic acid synthase (N-acetylneuraminate synthase) (EC 2.5.1.56) (N-acetylneuraminate-9-phosphate synthase) (EC 2.5.1.57) (N-acetylneuraminic acid	40 kDa	0.047	16	11	5	0	6	2	3	2	3	0	0	-1.750735545	0% (0.15)

				phosphate synthase) (N-acetylneuraminic acid synthase)															
A6NJZ9_HUMAN (+2)	DBLOH_HUMAN	DIABLO	Q9NR28	Diablo homolog, mitochondrial (Direct IAP-binding protein with low pI) (Second mitochondria-derived activator of caspase) (Smac)	27 kDa	0.056	16	11	5	2	5	2	2	2	0	1	2	-1.750735545	0% (0.15)
D6W4Z6_HUMAN (+1)	SYLM_HUMAN	LARS2	Q15031	Probable leucine--tRNA ligase, mitochondrial (EC 6.1.1.4) (Leucyl-tRNA synthetase) (LeuRS)	102 kDa	0.098	19	13	6	5	2	4	2	4	0	0	2	-1.755683587	0% (0.13)
DCD_HUMAN	B2R5N4_HUMAN		B2R5N4	cDNA, FLJ92544, highly similar to Homo sapiens lectin, mannose-binding 2-like (LMAN2L), mRNA	40 kDa	0.33	19	13	6	4	4	1	4	0	2	0	4	-1.755683587	0% (0.13)
KAD1_HUMAN (+2)	AP1M2_HUMAN	AP1M2	Q9Y6Q5	AP-1 complex subunit mu-2 (AP-mu chain family member mu1B) (Adaptor protein complex AP-1 subunit mu-2) (Adaptor-related protein complex 1 subunit mu-2) (Clathrin assembly protein complex 1 mu-2 medium chain 2) (Golgi adaptor HA1/AP1 adaptin mu-2 subunit) (Mu-adaptin 2) (Mu1B-adaptin)	48 kDa	0.2	19	13	6	8	1	2	2	2	1	1	2	-1.755683587	0% (0.13)
B4DPS5_HUMAN (+3)	Q53HP9_HUMAN		Q53HP9	Chromosome 14 open reading frame 159 variant (Fragment)	44 kDa	0.11	19	13	6	6	1	1	5	3	0	3	0	-1.755683587	0% (0.13)
F5GYG5_HUMAN (+1)	CBPD_HUMAN	CPD	O75976	Carboxypeptidase D (EC 3.4.17.22) (Metallocarboxypeptidase D) (gp180)	153 kDa	0.18	22	15	7	10	3	2	0	1	0	2	4	-1.759437005	0% (0.11)
LONP2_HUMAN	B5BU72_HUMAN	PICALM	B5BU72	Phosphatidylinositol-binding clathrin assembly protein isoform 2	66 kDa	0.04	22	15	7	0	6	9	1	2	0	3	2	-1.759437005	0% (0.11)
RAB12_HUMAN	U2AF1_HUMAN	U2AF1	Q01081	Splicing factor U2AF 35 kDa subunit (U2 auxiliary factor 35 kDa subunit) (U2 small nuclear RNA auxiliary factor 1) (U2 snRNP auxiliary factor small subunit)	28 kDa	0.13	21	15	7	1	3	4	7	2	1	2	2	-1.759437005	0% (0.11)
B2RAH7_HUMAN (+2)	B7Z4K8_HUMAN		B7Z4K8	cDNA FLJ52869, highly similar to Homo sapiens basic leucine zipper and W2 domains 2 (BZW2), mRNA	45 kDa	0.07	22	15	7	0	5	6	4	0	0	4	1	-1.759437005	0% (0.11)
RIC8A_HUMAN	B0YJ32_HUMAN	LAMA3	B0YJ32	Laminin alpha-3 chain variant 1	367 kDa	0.072	28	19	9	9	5	5	0	4	0	0	5	-1.764758554	0% (0.081)
NOG2_HUMAN	THEM6_HUMAN	THEM6	Q8WUY1	Protein THEM6 (Mesenchymal stem cell protein DSCD75) (Thioesterase superfamily member 6)	24 kDa	0.049	28	19	9	3	4	3	9	3	1	4	2	-1.764758554	0% (0.081)
Q12915_HUMAN	CTL1_HUMAN	SLC44A1	Q8WWI5	Choline transporter-like protein 1 (CDw92) (Solute carrier family 44 member 1) (CD antigen CD92)	73 kDa	0.13	28	19	9	3	8	7	1	2	1	4	2	-1.764758554	0% (0.081)
SLTM_HUMAN	EFHD2_HUMAN	EFHD2	Q96C19	EF-hand domain-containing protein D2 (Swiprosin-1)	27 kDa	0.019	31	21	10	1	6	11	4	0	4	3	3	-1.76671518	0% (0.069)
NASP_HUMAN (+1)	B7Z7G9_HUMAN		B7Z7G9	cDNA FLJ56170, highly similar to Glucosylceramidase (EC 3.2.1.45)	55 kDa	0.057	33	23	11	11	7	5	0	3	0	4	4	-1.768355659	0% (0.060)
RL7L_HUMAN	A8KA84_HUMAN		A8KA84	cDNA FLJ78682, highly similar to Homo sapiens 2'-5'-oligoadenylate synthetase 3, 100kDa (OAS3), mRNA	121 kDa	0.16	38	25	12	0	1	3	21	1	6	5	0	-1.769751571	0% (0.051)
NOC4L_HUMAN	QOR_HUMAN	CRYZ	Q08257	Quinone oxidoreductase (EC 1.6.5.5) (NADPH:quinone reductase) (Zeta-crystallin)	35 kDa	0.045	37	25	12	10	3	2	10	4	2	1	5	-1.769751571	0% (0.051)
GLRX3_HUMAN	Q6DEN2_HUMAN	DPYSL3	Q6DEN2	DPYSL3 protein	74 kDa	0.081	40	27	13	0	12	6	9	0	6	0	7	-1.770954405	95% (0.044)
RASM_HUMAN	B4E2A6_HUMAN		B4E2A6	cDNA FLJ55508, highly similar to Sad1/unc-84-like protein 2	84 kDa	0.13	52	35	17	22	4	4	5	2	1	4	10	-1.774469733	95% (0.025)

ITCH_HUMAN	AHNK_HUMAN	AHNAK	Q09666	Neuroblast differentiation-associated protein AHNAK (Desmoyokin)	629 kDa	0.05	663	442	222	173	122	101	51	88	14	58	63	-1.777770945	95% (< 0.00010)
STRP1_HUMAN	G5E972_HUMAN	TMPO	G5E972	Thymopentin (Thymopoietin, isoform CRA_d)	46 kDa	0.024	88	59	29	12	15	20	12	11	2	6	10	-1.779566127	95% (0.0050)
B3KTA3_HUMAN (+3)	H2AZ_HUMAN	H2AFZ	P0C0S5	Histone H2A.Z (H2A/z)	14 kDa	0.055	255	169	84	34	35	38	63	31	16	10	28	-1.785443008	95% (< 0.00010)
RTN2_HUMAN	MAOM_HUMAN	ME2	P23368	NAD-dependent malic enzyme, mitochondrial (NAD-ME) (EC 1.1.1.38) (Malic enzyme 2)	65 kDa	0.048	224	151	74	19	55	54	23	14	21	11	28	-1.808697574	95% (< 0.00010)
Q53G41_HUMAN	B3KXC3_HUMAN		B3KXC3	Ferritin	21 kDa	0.061	55	38	18	4	15	17	3	3	2	6	8	-1.821557478	95% (0.017)
B2RC75_HUMAN	H10_HUMAN	H1F0	P07305	Histone H1.0 (Histone H1') (Histone H1(0)) [Cleaved into: Histone H1.0, N-terminally processed]	21 kDa	0.075	41	28	13	4	6	8	10	2	4	2	5	-1.833666623	95% (0.034)
NUP50_HUMAN	TPBG_HUMAN	TPBG	Q13641	Trophoblast glycoprotein (5T4 oncofetal antigen) (5T4 oncofetal trophoblast glycoprotein) (5T4 oncotrophoblast glycoprotein) (M6P1)	46 kDa	0.09	41	28	13	5	12	11	0	1	2	2	8	-1.833666623	95% (0.034)
PKD2_HUMAN	R4RWU8_HUMAN	HLA-C	R4RWU8	MHC class I antigen (Fragment)	32 kDa	0.024	99	67	32	11	17	13	25	0	0	10	12	-1.834084704	95% (0.0020)
CUL2_HUMAN (+1)	SPTB2_HUMAN	SPTBN1	Q01082	Spectrin beta chain, non-erythrocytic 1 (Beta-II spectrin) (Fodrin beta chain) (Spectrin, non-erythroid beta chain 1)	275 kDa	0.017	146	100	48	28	25	35	12	19	2	16	11	-1.837306018	95% (0.00021)
F134A_HUMAN	LAMB3_HUMAN	LAMB3	Q13751	Laminin subunit beta-3 (Epiligrin subunit bata) (Kalinin B1 chain) (Kalinin subunit beta) (Laminin B1k chain) (Laminin-5 subunit beta) (Nicein subunit beta)	130 kDa	0.063	35	24	11	9	9	6	0	0	0	0	11	-1.841301206	95% (0.045)
B2R7C2_HUMAN (+1)	A7BI36_HUMAN	RRBP1	A7BI36	p180/ribosome receptor	166 kDa	0.028	80	55	26	9	25	17	5	4	0	18	5	-1.844314123	95% (0.0044)
CSDE1_HUMAN (+3)	B3KTJ9_HUMAN		B3KTJ9	cDNA FLJ38393 fis, clone FEBRA2007212	103 kDa	0.049	32	22	10	6	7	5	4	1	2	3	4	-1.846141835	0% (0.052)
FARP1_HUMAN	B3KUF6_HUMAN		B3KUF6	cDNA FLJ39748 fis, clone SMINT2017436, highly similar to Homo sapiens haloacid dehalogenase-like hydrolase domain containing 3 (HDHD3), mRNA	28 kDa	0.028	33	22	10	5	5	4	8	2	4	2	2	-1.846141835	0% (0.052)
Q9TP39_HUMAN	ARL1_HUMAN	ARL1	P40616	ADP-ribosylation factor-like protein 1	20 kDa	0.54	29	20	9	7	4	4	5	1	4	3	1	-1.851930923	0% (0.060)
CORO7_HUMAN	CISD1_HUMAN	CISD1	Q9NZ45	CDGSH iron-sulfur domain-containing protein 1 (MitoNEET)	12 kDa	0.039	29	20	9	7	4	2	7	1	3	5	0	-1.851930923	0% (0.060)
C9JZG9_HUMAN (+3)	RL23A_HUMAN	RPL23A	P62750	60S ribosomal protein L23a	18 kDa	0.29	26	18	8	3	3	4	8	2	3	1	2	-1.858976079	0% (0.070)
M0QYZ2_HUMAN	VASP_HUMAN	VASP	P50552	Vasodilator-stimulated phosphoprotein (VASP)	40 kDa	0.2	25	18	8	1	5	10	2	1	3	2	2	-1.858976079	0% (0.070)
B4DU97_HUMAN	RAB9A_HUMAN	RAB9A	P51151	Ras-related protein Rab-9A	23 kDa	0.18	26	18	8	5	5	3	5	1	0	3	4	-1.858976079	0% (0.070)
B2RNR6_HUMAN (+1)	AP1M1_HUMAN	AP1M1	Q9BXS5	AP-1 complex subunit mu-1 (AP-mu chain family member mu1A) (Adapter-related protein complex 1 subunit mu-1) (Adaptor protein complex AP-1 subunit mu-1) (Clathrin assembly protein complex 1 mu-1 medium chain 1) (Clathrin coat assembly protein AP47) (Clathrin coat-associated protein AP47) (Golgi adaptor HA1/AP1	49 kDa	0.036	26	18	8	3	6	6	3	2	1	3	2	-1.858976079	0% (0.070)

				adaptin mu-1 subunit) (Mu-adaptin 1) (Mu1A-adaptin)															
I6RUN0_HUMAN	LOX5_HUMAN	ALOX5	P09917	Arachidonate 5-lipoxygenase (5-LO) (5-lipoxygenase) (EC 1.13.11.34)	78 kDa	0.54	63	43	20	7	8	11	18	0	1	5	14	-1.860388767	95% (0.0097)
A6H8W6_HUMAN (+4)	B5BU01_HUMAN	EIF2S2	B5BU01	Eukaryotic translation initiation factor 2 beta	38 kDa	0.014	23	16	7	0	5	5	6	2	1	2	2	-1.867734053	0% (0.081)
CAND2_HUMAN	TOIP1_HUMAN	TOR1AIP1	Q5JTV8	Torsin-1A-interacting protein 1 (Lamin-associated protein 1B) (LAP1B)	66 kDa	0.024	85	58	27	20	9	12	17	3	9	3	13	-1.873955511	95% (0.0030)
EPS8_HUMAN	GNAI1_HUMAN	GNAI1	P63096	Guanine nucleotide-binding protein G(i) subunit alpha-1 (Adenylate cyclase-inhibiting G alpha protein)	40 kDa	0.019	84	58	27	17	0	15	0	6	0	7	9	-1.873955511	95% (0.0030)
FAAH1_HUMAN	PSA5_HUMAN	PSMA5	P28066	Proteasome subunit alpha type-5 (EC 3.4.25.1) (Macropain zeta chain) (Multicatalytic endopeptidase complex zeta chain) (Proteasome zeta chain)	26 kDa	0.041	20	14	6	0	5	3	6	2	4	1	0	-1.878913597	0% (0.095)
B2RBE6_HUMAN (+2)	LAMB2_HUMAN	LAMB2	P55268	Laminin subunit beta-2 (Laminin B1s chain) (Laminin-11 subunit beta) (Laminin-14 subunit beta) (Laminin-15 subunit beta) (Laminin-3 subunit beta) (Laminin-4 subunit beta) (Laminin-7 subunit beta) (Laminin-9 subunit beta) (S-laminin subunit beta) (S-LAM beta)	196 kDa	0.028	20	14	6	4	3	6	1	6	0	0	0	-1.878913597	0% (0.095)
Q53F93_HUMAN	IVD_HUMAN	IVD	P26440	Isovaleryl-CoA dehydrogenase, mitochondrial (IVD) (EC 1.3.8.4)	46 kDa	0.071	20	14	6	7	2	2	3	2	2	1	1	-1.878913597	0% (0.095)
IPO8_HUMAN	B2RDW0_HUMAN		B2RDW0	cDNA, FLJ96792, highly similar to Homo sapiens calmodulin 2 (phosphorylase kinase, delta) (CALM2), mRNA	17 kDa	0.023	48	33	15	6	6	13	8	3	2	4	6	-1.882920558	95% (0.019)
ABCF2_HUMAN (+1)	NIBL1_HUMAN	FAM129B	Q96TA1	Niban-like protein 1 (Meg-3) (Melanoma invasion by ERK) (MINERVA) (Protein FAM129B)	84 kDa	0.069	45	31	14	4	9	6	11	1	5	6	2	-1.889207871	95% (0.022)
I6NXG5_HUMAN	SEPT9_HUMAN	40057	Q9UHD8	Septin-9 (MLL septin-like fusion protein MSF-A) (MLL septin-like fusion protein) (Ovarian/Breast septin) (Ov/Br septin) (Septin D1)	65 kDa	0.53	45	31	14	4	10	12	5	0	4	6	4	-1.889207871	95% (0.022)
ACD10_HUMAN	D3DR31_HUMAN	IFIT1	D3DR31	Interferon-induced protein with tetratricopeptide repeats 1 (Interferon-induced protein with tetratricopeptide repeats 1, isoform CRA_b)	52 kDa	0.033	45	31	14	0	0	0	31	0	10	4	0	-1.889207871	95% (0.022)
A8KA74_HUMAN (+2)	B3KS98_HUMAN	EIF3H	B3KS98	Eukaryotic translation initiation factor 3 subunit H (eIF3h) (Eukaryotic translation initiation factor 3 subunit 3) (eIF-3 gamma) (eIF3 p40 subunit)	42 kDa	0.016	17	12	5	0	7	1	4	1	4	0	0	-1.893677065	0% (0.11)
SBDS_HUMAN	B2R5I8_HUMAN		B2R5I8	cDNA, FLJ92490, highly similar to Homo sapiens RAB32, member RAS oncogene family (RAB32), mRNA	25 kDa	0.14	17	12	5	6	3	1	2	0	1	1	3	-1.893677065	0% (0.11)
ZNT1_HUMAN	K7ERC8_HUMAN	KDSR	K7ERC8	3-ketodihydrosphingosine reductase (Fragment)	23 kDa	0.057	17	12	5	6	2	3	1	0	3	0	2	-1.893677065	0% (0.11)
H2A2B_HUMAN	STX4_HUMAN	STX4	Q12846	Syntaxin-4 (Renal carcinoma antigen NY-REN-31)	34 kDa	0.3	16	12	5	3	1	3	5	1	1	0	3	-1.893677065	0% (0.11)
DYL2_HUMAN	DNJC3_HUMAN	DNAJC3	Q13217	DnaJ homolog subfamily C member 3 (Endoplasmic reticulum DNA J domain-containing protein 6) (ER-resident protein ERdj6) (ERdj6) (Interferon-induced, double-stranded RNA-activated protein kinase	58 kDa	0.38	38	27	12	5	11	7	4	1	2	3	6	-1.904638743	95% (0.029)

				inhibitor) (Protein kinase inhibitor of 58 kDa) (Protein kinase inhibitor p58)															
F8W9Y0_HUMAN (+2)	Q6GMX0_HUMAN		Q6GMX0	Uncharacterized protein	26 kDa	0.11	61	42	19	2	16	13	12	5	0	6	8	-1.908143878	95% (0.0084)
B3KR61_HUMAN (+1)	BIG2_HUMAN	ARFGEF2	Q9Y6D5	Brefeldin A-inhibited guanine nucleotide-exchange protein 2 (Brefeldin A-inhibited GEP 2) (ADP-ribosylation factor guanine nucleotide-exchange factor 2)	202 kDa	0.12	14	10	4	2	5	1	2	1	2	1	0	-1.914072413	0% (0.13)
EMD_HUMAN	THMS2_HUMAN	THEMIS2	Q5TEJ8	Protein THEMIS2 (Induced by contact to basement membrane 1 protein) (Protein ICB-1) (Thymocyte-expressed molecule involved in selection protein 2)	72 kDa	0.11	14	10	4	2	3	3	2	0	1	3	0	-1.914072413	0% (0.13)
CX6B1_HUMAN	TOR4A_HUMAN	TOR4A	Q9NXH8	Torsin-4A (Torsin family 4 member A)	47 kDa	0.27	14	10	4	7	2	1	0	0	0	0	4	-1.914072413	0% (0.13)
TOR2A_HUMAN	FRIL_HUMAN	FTL	P02792	Ferritin light chain (Ferritin L subunit)	20 kDa	0.12	118	83	38	8	29	32	15	2	7	12	16	-1.918185005	95% (0.00033)
TIM22_HUMAN	PLXB2_HUMAN	PLXNB2	O15031	Plexin-B2 (MM1)	205 kDa	0.16	55	38	17	21	7	6	4	6	1	9	2	-1.921396491	95% (0.011)
DGLB_HUMAN (+1)	RENT1_HUMAN	UPF1	Q92900	Regulator of nonsense transcripts 1 (EC 3.6.4.-) (ATP-dependent helicase RENT1) (Nonsense mRNA reducing factor 1) (NORF1) (Up-frameshift suppressor 1 homolog) (hUpf1)	124 kDa	0.046	74	51	23	5	16	18	12	11	8	4	0	-1.925084678	95% (0.0038)
PPCS_HUMAN	Q53GX6_HUMAN		Q53GX6	Nucleobindin 1 variant (Fragment)	54 kDa	0.051	52	36	16	12	8	12	4	2	0	5	9	-1.929178719	95% (0.012)
SFXN2_HUMAN	Q6N097_HUMAN	DKFZp686H20196	Q6N097	Putative uncharacterized protein DKFZp686H20196	53 kDa	0.13	49	34	15	2	13	11	8	1	2	5	8	-1.93792125	95% (0.014)
A2ACR1_HUMAN (+2)	SYNE2_HUMAN	SYNE2	Q8WXH0	Nesprin-2 (Nuclear envelope spectrin repeat protein 2) (Nucleus and actin connecting element protein) (Protein NUANCE) (Synaptic nuclear envelope protein 2) (Syne-2)	796 kDa	0.14	30	21	9	17	3	1	0	2	0	7	0	-1.939105534	95% (0.044)
CLYBL_HUMAN	F8W7U8_HUMAN	MRE11A	F8W7U8	Double-strand break repair protein MRE11A	81 kDa	0.074	11	8	3	1	2	2	3	1	0	2	0	-1.944075084	0% (0.16)
ASGL1_HUMAN	D3DWV9_HUMAN	hCG_2004980	D3DWV9	HCG2004980, isoform CRA_g	37 kDa	0.14	11	8	3	5	0	0	4	1	0	0	2	-1.944075084	0% (0.16)
B7Z3U1_HUMAN (+2)	CAB39_HUMAN	CAB39	Q9Y376	Calcium-binding protein 39 (MO25alpha) (Protein Mo25)	40 kDa	0.063	11	8	3	2	2	2	3	1	1	1	0	-1.944075084	0% (0.16)
NDUBB_HUMAN (+1)	B1AHD1_HUMAN	NHP2L1	B1AHD1	NHP2-like protein 1	15 kDa	0.18	11	8	3	0	1	1	6	0	1	1	1	-1.944075084	0% (0.16)
STX6_HUMAN	DDX58_HUMAN	DDX58	O95786	Probable ATP-dependent RNA helicase DDX58 (EC 3.6.4.13) (DEAD box protein 58) (RIG-I-like receptor 1) (RLR-1) (Retinoic acid-inducible gene 1 protein) (RIG-1) (Retinoic acid-inducible gene I protein) (RIG-I)	107 kDa	0.047	11	8	3	0	0	0	8	1	1	1	0	-1.944075084	0% (0.16)
PI42C_HUMAN	THOC4_HUMAN	ALYREF	Q86V81	THO complex subunit 4 (Tho4) (Ally of AML-1 and LEF-1) (Aly/REF export factor) (Transcriptional coactivator Aly/REF) (bZIP-enhancing factor BEF)	27 kDa	0.074	11	8	3	0	3	2	3	0	0	1	2	-1.944075084	0% (0.16)
F6IQY8_HUMAN	Q53G62_HUMAN		Q53G62	Mitochondrial ribosomal protein S28 variant (Fragment)	21 kDa	0.28	11	8	3	4	1	1	2	1	0	0	2	-1.944075084	0% (0.16)
A8KAJ7_HUMAN (+2)	CAH1_HUMAN	CA1	P00915	Carbonic anhydrase 1 (EC 4.2.1.1) (Carbonate dehydratase I) (Carbonic	29 kDa	0.055	11	8	3	0	2	2	4	0	0	0	3	-1.944075084	0% (0.16)

				anhydrase B) (CAB) (Carbonic anhydrase I) (CA-I)															
B4DEX8_HUMAN (+1)	RHOG_HUMAN	RHOG	P84095	Rho-related GTP-binding protein RhoG	21 kDa	0.12	11	8	3	1	1	1	5	0	1	1	1	-1.944075084	0% (0.16)
DNJC9_HUMAN	TOR2A_HUMAN	TOR2A	Q5JU69	Torsin-2A (Torsin family 2 member A) (Torsin-related protein 1)	36 kDa	0.11	11	8	3	0	2	1	3	0	1	2	0	-1.944075084	0% (0.16)
ADNP_HUMAN (+2)	Q53EW8_HUMAN		Q53EW8	Thiosulfate sulfurtransferase variant (Fragment)	33 kDa	0.026	43	30	13	12	8	6	4	7	3	0	3	-1.959095896	95% (0.019)
B4DDT6_HUMAN (+2)	LEMD2_HUMAN	LEMD2	Q8NC56	LEM domain-containing protein 2 (hLEM2)	57 kDa	0.15	60	41	18	11	6	5	19	4	6	3	5	-1.960860416	95% (0.0072)
AURKB_HUMAN (+4)	B7XGC2_HUMAN	HLA-A	B7XGC2	MHC class I antigen (Fragment)	32 kDa	0.078	75	52	23	5	11	13	23	3	5	8	7	-1.961953627	95% (0.0028)
CP4X1_HUMAN	HBD_HUMAN	HBD	P02042	Hemoglobin subunit delta (Delta-globin) (Hemoglobin delta chain)	16 kDa	0.047	343	235	106	0	76	82	67	0	0	26	0	-1.970422561	95% (< 0.00010)
A8K4P8_HUMAN (+1)	B2R6F5_HUMAN		B2R6F5	cDNA, FLJ92928, highly similar to Homo sapiens retinitis pigmentosa 2 (X-linked recessive) (RP2), mRNA	40 kDa	0.016	24	17	7	7	4	5	1	0	1	2	4	-1.976033884	0% (0.059)
NEK9_HUMAN	A8K878_HUMAN		A8K878	cDNA FLJ77177, highly similar to Homo sapiens arginine-rich, mutated in early stage tumors (ARMET), mRNA	21 kDa	0.027	24	17	7	4	4	7	2	0	5	0	2	-1.976033884	0% (0.059)
RADI_HUMAN	NLRX1_HUMAN	NLRX1	Q86UT6	NLR family member X1 (Caterpiller protein 11.3) (CLR11.3) (Nucleotide-binding oligomerization domain protein 26) (Nucleotide-binding oligomerization domain protein 5) (Nucleotide-binding oligomerization domain protein 9)	108 kDa	0.15	8	6	2	2	2	1	1	1	1	0	0	-1.992553376	0% (0.19)
C9JS50_HUMAN (+1)	J3QT16_HUMAN	PTK2	J3QT16	Focal adhesion kinase 1	121 kDa	0.11	8	6	2	0	2	4	0	1	0	1	0	-1.992553376	0% (0.19)
B0QZ43_HUMAN (+3)	B4DDF4_HUMAN	CNN2	B4DDF4	Calponin-2 (cDNA FLJ52469, highly similar to Calponin-2)	33 kDa	0.2	8	6	2	2	2	2	0	0	0	0	2	-1.992553376	0% (0.19)
RGPA2_HUMAN	ADRO_HUMAN	FDXR	P22570	NADPH:adrenodoxin oxidoreductase, mitochondrial (AR) (Adrenodoxin reductase) (EC 1.18.1.6) (Ferredoxin--NADP(+) reductase) (Ferredoxin reductase)	54 kDa	0.012	8	6	2	2	0	0	4	0	2	0	0	-1.992553376	0% (0.19)
E7EU94_HUMAN	Q32Q10_HUMAN	RSU1	Q32Q10	RSU1 protein (Fragment)	31 kDa	0.1	8	6	2	0	2	4	0	0	1	1	1	-1.992553376	0% (0.19)
A7E2E5_HUMAN (+1)	MBD2_HUMAN	MBD2	Q9UBB5	Methyl-CpG-binding domain protein 2 (Demethylase) (DMTase) (Methyl-CpG-binding protein MBD2)	43 kDa	0.022	8	6	2	1	2	1	2	0	0	0	2	-1.992553376	0% (0.19)
E9PDC5_HUMAN (+2)	B4DE65_HUMAN		B4DE65	cDNA FLJ57705, highly similar to Acid sphingomyelinase-like phosphodiesterase 3b (EC 3.1.4.-)	43 kDa	0.04	8	6	2	6	0	0	0	2	0	0	0	-1.992553376	0% (0.19)
A8K6A6_HUMAN (+2)	A8K8K1_HUMAN		A8K8K1	cDNA FLJ76936, highly similar to Homo sapiens RNA terminal phosphate cyclase domain 1 (RTCD1), mRNA	39 kDa	0.042	8	6	2	0	2	0	4	1	1	0	0	-1.992553376	0% (0.19)
B7Z7E4_HUMAN	B7Z8X5_HUMAN		B7Z8X5	cDNA FLJ61541, highly similar to Homo sapiens PDZ and LIM domain 5 (PDLIM5), transcript variant 2, mRNA	53 kDa	0.088	8	6	2	2	3	1	0	0	0	2	0	-1.992553376	0% (0.19)
AOFA_HUMAN (+2)	NDUB7_HUMAN	NDUFB7	P17568	NADH dehydrogenase [ubiquinone] 1 beta subcomplex subunit 7 (Cell adhesion protein SQM1) (Complex I-B18) (CI-B18) (NADH-ubiquinone oxidoreductase B18 subunit)	16 kDa	0.046	8	6	2	2	1	2	1	1	0	1	0	-1.992553376	0% (0.19)

Q53GD8_HUMAN	B4DRE6_HUMAN	SPATS2L	B4DRE6	SPATS2-like protein (cDNA FLJ59743, weakly similar to Mus musculus spermatogenesis associated, serine-rich 2 (Spats2), mRNA)	63 kDa	0.11	8	6	2	0	3	2	1	0	0	2	0	-1.992553376	0% (0.19)
GSTM4_HUMAN	B4DVL2_HUMAN	NAAA	B4DVL2	N-acylethanolamine-hydrolyzing acid amidase (cDNA FLJ54891, highly similar to N-acylethanolamine-hydrolyzing acid amidase (EC 3.5.1.-))	28 kDa	0.26	8	6	2	1	2	2	1	0	0	1	1	-1.992553376	0% (0.19)
A4D212_HUMAN (+1)	FKB1A_HUMAN	FKBP1A	P62942	Peptidyl-prolyl cis-trans isomerase FKBP1A (PPIase FKBP1A) (EC 5.2.1.8) (12 kDa FK506-binding protein) (12 kDa FKBP) (FKBP-12) (Calstabin-1) (FK506-binding protein 1A) (FKBP-1A) (Immunophilin FKBP12) (Rotamase)	12 kDa	0.01	8	6	2	0	1	1	4	1	0	1	0	-1.992553376	0% (0.19)
Q59GW6_HUMAN	RET1_HUMAN	RBP1	P09455	Retinol-binding protein 1 (Cellular retinol-binding protein) (CRBP) (Cellular retinol-binding protein I) (CRBP-I)	16 kDa	0.11	8	6	2	1	1	0	4	0	2	0	0	-1.992553376	0% (0.19)
IGHG2_HUMAN (+1)	41_HUMAN	EPB41	P11171	Protein 4.1 (P4.1) (4.1R) (Band 4.1) (EPB4.1)	97 kDa	0.23	8	6	2	0	3	1	0	1	0	0	0	-1.992553376	0% (0.19)
Q6QNY7_HUMAN	B3KR61_HUMAN	NDUFAF5	B3KR61	HCG1811060, isoform CRA_c (NADH dehydrogenase [ubiquinone] 1 alpha subcomplex assembly factor 5) (cDNA FLJ33741 fis, clone BRAWH2018875, weakly similar to Protein At1g22800)	30 kDa	0.055	8	6	2	1	1	1	3	2	0	0	0	-1.992553376	0% (0.19)
LAR4B_HUMAN	B4DDP6_HUMAN	DBNL	B4DDP6	Drebrin-like protein (cDNA FLJ52531, highly similar to Drebrin-like protein)	43 kDa	0.031	21	15	6	3	2	5	5	3	2	0	1	-2.002146774	0% (0.068)
PTGR1_HUMAN	A8KAH7_HUMAN		A8KAH7	cDNA FLJ75444, highly similar to Homo sapiens protein kinase, cAMP-dependent, regulatory, type II, alpha (PRKAR2A), mRNA	46 kDa	0.067	21	15	6	2	5	3	5	2	3	1	0	-2.002146774	0% (0.068)
ENOPH_HUMAN (+1)	BCAT1_HUMAN	BCAT1	P54687	Branched-chain-amino-acid aminotransferase, cytosolic (BCAT(c)) (EC 2.6.1.42) (Protein ECA39)	43 kDa	0.092	21	15	6	0	9	6	0	0	5	1	0	-2.002146774	0% (0.068)
F210B_HUMAN	JAGN1_HUMAN	JAGN1	Q8N5M9	Protein jagunal homolog 1	21 kDa	0.15	21	15	6	7	3	1	4	2	4	0	0	-2.002146774	0% (0.068)
B4DQQ5_HUMAN	FLOT2_HUMAN	FLOT2	Q14254	Flotillin-2 (Epidermal surface antigen) (ESA) (Membrane component chromosome 17 surface marker 1)	47 kDa	0.047	71	49	21	23	10	8	10	2	9	3	7	-2.017821141	95% (0.0027)
RPP30_HUMAN	H6A2E0_HUMAN	HLA-B	H6A2E0	MHC class I antigen (Fragment)	21 kDa	0.21	54	38	16	0	9	0	15	0	0	7	0	-2.032811043	95% (0.0068)
AGM1_HUMAN (+1)	IF2A_HUMAN	EIF2S1	P05198	Eukaryotic translation initiation factor 2 subunit 1 (Eukaryotic translation initiation factor 2 subunit alpha) (eIF-2-alpha) (eIF-2A) (eIF-2alpha)	36 kDa	0.033	18	13	5	0	7	6	0	3	2	0	0	-2.036622259	0% (0.079)
E7EQ01_HUMAN	RETST_HUMAN	RETSAT	Q6NUM9	All-trans-retinol 13,14-reductase (EC 1.3.99.23) (All-trans-13,14-dihydroretinol saturase) (RetSat) (PPAR-alpha-regulated and starvation-induced gene protein)	67 kDa	0.31	18	13	5	9	1	1	2	0	1	1	3	-2.036622259	0% (0.079)
AATC_HUMAN (+1)	A8K4M4_HUMAN		A8K4M4	cDNA FLJ75329, highly similar to Homo sapiens LAG1 longevity assurance homolog 2 (S. cerevisiae), transcript variant 2, mRNA	45 kDa	0.061	18	13	5	4	3	3	3	0	1	2	2	-2.036622259	0% (0.079)
A0AV58_HUMAN	Q30077_HUMAN	HLA-DQB1	Q30077	MHC class II HLA-DQ-beta-1 (Fragment)	26 kDa	0.045	17	13	5	0	5	6	1	0	0	0	5	-2.036622259	0% (0.079)

ARI1A_HUMAN	DYN2_HUMAN	DNM2	P50570	Dynamin-2 (EC 3.6.5.5)	98 kDa	0.018	28	20	8	0	1	9	9	5	3	1	0	-2.052169195	95% (0.037)
VANG1_HUMAN	A0S1I7_HUMAN	COX1	A0S1I7	Cytochrome c oxidase subunit 1 (EC 1.9.3.1) (Fragment)	57 kDa	0.097	27	20	8	8	4	5	2	2	2	1	3	-2.052169195	95% (0.037)
GALT7_HUMAN	OCAD2_HUMAN	OCIAD2	Q56VL3	OCIA domain-containing protein 2 (Ovarian carcinoma immunoreactive antigen-like protein)	17 kDa	0.04	38	27	11	14	7	3	3	2	4	1	4	-2.060149096	95% (0.018)
LRC8C_HUMAN	Q6FGL0_HUMAN	LGALS3	Q6FGL0	Galectin (Fragment)	26 kDa	0.059	48	34	14	10	6	3	15	2	5	4	3	-2.065027763	95% (0.0088)
Q6NUR1_HUMAN	B2R7U4_HUMAN		B2R7U4	cDNA, FLJ93605, highly similar to Homo sapiens heme oxygenase (decycling) 1 (HMOX1), mRNA	33 kDa	0.026	58	41	17	4	13	18	5	0	2	9	6	-2.068334581	95% (0.0044)
A8K070_HUMAN (+2)	B2R4M6_HUMAN		B2R4M6	cDNA, FLJ92148, highly similar to Homo sapiens S100 calcium binding protein A9 (calgranulin B) (S100A9), mRNA	13 kDa	0.049	99	69	29	3	22	32	13	1	4	12	11	-2.075196619	95% (0.00031)
J3QRV5_HUMAN (+1)	B3KS62_HUMAN		B3KS62	cDNA FLJ35573 fis, clone SPLEN2005927, highly similar to Tripartite motif-containing protein 4	54 kDa	0.039	2	4	1	1	0	1	0	0	1	0	0	-2.084141449	0% (0.23)
B4DM85_HUMAN (+1)	PDIA5_HUMAN	PDIA5	Q14554	Protein disulfide-isomerase A5 (EC 5.3.4.1) (Protein disulfide isomerase-related protein)	60 kDa	0.059	5	4	1	3	1	0	0	1	0	0	0	-2.084141449	0% (0.23)
LSG1_HUMAN	F5GXA0_HUMAN	SUMF1	F5GXA0	Sulfatase-modifying factor 1	47 kDa	0.029	5	4	1	1	0	3	0	0	0	0	1	-2.084141449	0% (0.23)
A6NHH5_HUMAN	LTOR4_HUMAN	LAMTOR4	Q0VGL1	Ragulator complex protein LAMTOR4 (Late endosomal/lysosomal adaptor and MAPK and MTOR activator 4) [Cleaved into: Ragulator complex protein LAMTOR4, N-terminally processed]	11 kDa	0.12	6	4	1	0	2	0	2	0	0	1	0	-2.084141449	0% (0.23)
AT11A_HUMAN (+3)	ABC3G_HUMAN	APOBEC3G	Q9HC16	DNA dC->dU-editing enzyme APOBEC-3G (EC 3.5.4.-) (APOBEC-related cytidine deaminase) (APOBEC-related protein) (ARCD) (APOBEC-related protein 9) (ARP-9) (CEM-15) (CEM15) (Deoxycytidine deaminase) (A3G)	46 kDa	0.022	5	4	1	0	3	1	0	1	0	0	0	-2.084141449	0% (0.23)
RENT2_HUMAN	SPG21_HUMAN	SPG21	Q9NZD8	Maspardin (Acid cluster protein 33) (Spastic paraplegia 21 autosomal recessive Mast syndrome protein) (Spastic paraplegia 21 protein)	35 kDa	0.024	5	4	1	1	3	0	0	0	0	0	1	-2.084141449	0% (0.23)
Q9Y3E8_HUMAN	ACOC_HUMAN	ACO1	P21399	Cytoplasmic aconitate hydratase (Aconitase) (EC 4.2.1.3) (Citrate hydro-lyase) (Ferritin repressor protein) (Iron regulatory protein 1) (IRP1) (Iron-responsive element-binding protein 1) (IRE-BP 1)	98 kDa	0.05	5	4	1	0	3	1	0	1	0	0	0	-2.084141449	0% (0.23)
B2R6H7_HUMAN (+2)	Q8WVC2_HUMAN	RPS21	Q8WVC2	40S ribosomal protein S21	9 kDa	0.035	5	4	1	0	2	2	0	1	0	0	0	-2.084141449	0% (0.23)
B4DIH5_HUMAN (+3)	SUSD2_HUMAN	SUSD2	Q9UGT4	Sushi domain-containing protein 2	90 kDa	0.095	5	4	1	0	2	2	0	0	0	1	0	-2.084141449	0% (0.23)
B3KQQ0_HUMAN (+1)	B7Z2R7_HUMAN		B7Z2R7	Acyl-CoA-binding domain-containing protein 5	59 kDa	0.075	5	4	1	3	1	0	1	0	0	0	1	-2.084141449	0% (0.23)
MISP_HUMAN	ADDG_HUMAN	ADD3	Q9UEY8	Gamma-adducin (Adducin-like protein 70)	79 kDa	0.016	5	4	1	0	1	3	0	0	0	1	0	-2.084141449	0% (0.23)
B3KM36_HUMAN (+2)	PPCS_HUMAN	PPCS	Q9HAB8	Phosphopantothenate--cysteine ligase (EC 6.3.2.5) (Phosphopantothenoylcysteine synthetase) (PPC synthetase)	34 kDa	0.081	5	4	1	0	1	1	2	0	1	0	0	-2.084141449	0% (0.23)

SSFA2_HUMAN	E9PLS9_HUMAN	TM7SF2	E9PLS9	Delta(14)-sterol reductase (Fragment)	30 kDa	0.017	5	4	1	2	0	0	2	0	0	0	1	-2.084141449	0% (0.23)
B4DEF8_HUMAN (+1)	A6PVN5_HUMAN	PPP2R4	A6PVN5	Serine/threonine-protein phosphatase 2A activator	37 kDa	0.079	5	4	1	0	0	0	4	0	1	0	0	-2.084141449	0% (0.23)
RM16_HUMAN	D3DSU3_HUMAN	KIF13B	D3DSU3	Kinesin family member 13B, isoform CRA_a	175 kDa	0.14	5	4	1	1	1	2	0	0	0	0	0	-2.084141449	0% (0.23)
CLCA_HUMAN (+1)	DHB8_HUMAN	HSD17B8	Q92506	Estradiol 17-beta-dehydrogenase 8 (EC 1.1.1.62) (17-beta-hydroxysteroid dehydrogenase 8) (17-beta-HSD 8) (3-oxoacyl-[acyl-carrier-protein] reductase) (EC 1.1.1.-) (Protein Ke6) (Ke-6) (Really interesting new gene 2 protein) (Testosterone 17-beta-dehydrogenase 8) (EC 1.1.1.239)	27 kDa	0.069	5	4	1	1	1	0	2	1	0	0	0	-2.084141449	0% (0.23)
NADE_HUMAN	TIM13_HUMAN	TIMM13	Q9Y5L4	Mitochondrial import inner membrane translocase subunit Tim13	11 kDa	0.044	5	4	1	0	0	1	3	0	0	1	0	-2.084141449	0% (0.23)
RCL1_HUMAN	B3GN3_HUMAN	B3GNT3	Q9Y2A9	UDP-GlcNAc:betaGal beta-1,3-N-acetylglucosaminyltransferase 3 (BGnT-3) (Beta-1,3-Gn-T3) (Beta-1,3-N-acetylglucosaminyltransferase 3) (Beta3Gn-T3) (EC 2.4.1.-) (Beta-1,3-galactosyltransferase 8) (Beta-1,3-GalTase 8) (Beta3Gal-T8) (Beta3GalT8) (b3Gal-T8) (Beta-3-Gx-T8) (Core 1 extending beta-1,3-N-acetylglucosaminyltransferase) (Core1-beta3GlcNAcT) (Transmembrane protein 3) (UDP-Gal:beta-GlcNAc beta-1,3-galactosyltransferase 8) (UDP-galactose:beta-N-acetylglucosamine beta-1,3-galactosyltransferase 8)	43 kDa	0.1	5	4	1	3	0	0	1	1	0	0	0	-2.084141449	0% (0.23)
Q96AD0_HUMAN (+1)	AL1A1_HUMAN	ALDH1A1	P00352	Retinal dehydrogenase 1 (RALDH 1) (RalDH1) (EC 1.2.1.36) (ALDH-E1) (ALHDII) (Aldehyde dehydrogenase family 1 member A1) (Aldehyde dehydrogenase, cytosolic)	55 kDa	0.12	5	4	1	0	2	2	0	1	0	0	0	-2.084141449	0% (0.23)
UIF_HUMAN	WFS1_HUMAN	WFS1	O76024	Wolframin	100 kDa	0.094	15	11	4	5	5	1	0	0	3	0	0	-2.084238965	0% (0.091)
SRP09_HUMAN	B2R802_HUMAN		B2R802	cDNA, FLJ93681, highly similar to Homo sapiens small nuclear ribonucleoprotein polypeptide A (SNRPA), mRNA	31 kDa	0.35	26	18	7	1	8	7	3	0	0	3	4	-2.084336498	95% (0.042)
B4DL79_HUMAN (+1)	B2R673_HUMAN		B2R673	cDNA, FLJ92818, highly similar to Homo sapiens pyruvate dehydrogenase complex, component X (PDHX), mRNA	54 kDa	0.025	35	25	10	9	7	3	5	5	2	2	1	-2.084434049	95% (0.020)
B3KQV7_HUMAN	ITB4_HUMAN	ITGB4	P16144	Integrin beta-4 (GP150) (CD antigen CD104)	202 kDa	0.045	55	39	16	30	4	4	1	2	0	2	12	-2.084629203	95% (0.0050)
B4DJ54_HUMAN (+1)	FA49B_HUMAN	FAM49B	Q9NUQ9	Protein FAM49B (L1)	37 kDa	0.094	31	23	9	1	11	5	6	0	4	4	1	-2.113461476	95% (0.023)
FA50A_HUMAN	C8C504_HUMAN	HBB	C8C504	Beta-globin	16 kDa	0.08	515	360	151	22	113	124	105	25	35	37	55	-2.124486189	95% (< 0.00010)
STAT2_HUMAN	PCAT2_HUMAN	LPCAT2	Q7L5N7	Lysophosphatidylcholine acyltransferase 2 (LPC acyltransferase 2) (LPCAT-2) (LysoPC acyltransferase 2) (EC 2.3.1.23) (1-acylglycerol-3-phosphate O-acyltransferase 11) (1-AGP acyltransferase 11) (1-AGPAT 11) (EC	60 kDa	0.047	12	9	3	5	2	2	0	0	0	1	2	-2.154273044	0% (0.11)

				2.3.1.51) (1-acylglycerophosphocholine O-acyltransferase) (1-alkylglycerophosphocholine O-acetyltransferase) (EC 2.3.1.67) (Acetyl-CoA:lyso-platelet-activating factor acetyltransferase) (Acetyl-CoA:lyso-PAF acetyltransferase) (Lyso-PAF acetyltransferase) (LysoPAFAT) (Acyltransferase-like 1) (Lysophosphatidic acid acyltransferase alpha) (LPAAT-alpha)															
GAS6_HUMAN (+1)	B4DIY7_HUMAN	TAMM41	B4DIY7	Mitochondrial translocator assembly and maintenance protein 41 homolog (cDNA FLJ60579)	51 kDa	0.028	13	9	3	4	2	2	1	2	1	0	0	-2.154273044	0% (0.11)
CATG_HUMAN	A8K3W7_HUMAN		A8K3W7	cDNA FLJ76271, highly similar to Homo sapiens protein O-fucosyltransferase 2 (POFUT2), transcript variant 3, mRNA	50 kDa	0.086	12	9	3	2	4	3	0	3	0	0	0	-2.154273044	0% (0.11)
B4DI03_HUMAN (+2)	A2A3U5_HUMAN	hCG_20471	A2A3U5	HCG20471, isoform CRA_d (cDNA FLJ76396, highly similar to Homo sapiens opioid receptor, sigma 1 (OPRS1), transcript variant 2, mRNA)	21 kDa	0.18	11	9	3	1	2	4	2	1	1	1	0	-2.154273044	0% (0.11)
A8K5G0_HUMAN (+2)	D6W5X3_HUMAN	SLC12A9	D6W5X3	Solute carrier family 12 (Potassium/chloride transporters), member 9, isoform CRA_d	69 kDa	0.015	12	9	3	3	2	3	1	0	0	0	3	-2.154273044	0% (0.11)
B7Z4C3_HUMAN (+2)	B4DKR0_HUMAN	STX5	B4DKR0	Syntaxin 5A, isoform CRA_c (Syntaxin-5) (cDNA FLJ57518, highly similar to Syntaxin-5)	29 kDa	0.052	12	9	3	2	3	2	2	1	0	1	1	-2.154273044	0% (0.11)
E7ESY4_HUMAN (+1)	FAM3C_HUMAN	FAM3C	Q92520	Protein FAM3C (Interleukin-like EMT inducer)	25 kDa	0.064	70	50	20	18	10	8	14	4	5	5	6	-2.15488146	95% (0.0012)
DDX54_HUMAN	DHRS7_HUMAN	DHRS7	Q9Y394	Dehydrogenase/reductase SDR family member 7 (EC 1.1.-.-) (Retinal short-chain dehydrogenase/reductase 4) (retSDR4)	38 kDa	0.039	53	38	15	18	8	7	5	0	5	3	7	-2.157938155	95% (0.0041)
B7ZMD7_HUMAN	B2RBL3_HUMAN		B2RBL3	cDNA, FLJ95575, highly similar to Homo sapiens endothelial cell growth factor 1 (platelet-derived) (ECGF1), mRNA	50 kDa	0.13	53	38	15	0	12	8	18	0	4	8	3	-2.157938155	95% (0.0041)
PKHG3_HUMAN	LRC8A_HUMAN	LRRC8A	Q8IWT6	Leucine-rich repeat-containing protein 8A	94 kDa	0.027	19	14	5	3	5	5	1	1	0	1	3	-2.179571127	0% (0.055)
B4DN34_HUMAN (+2)	B4E2S3_HUMAN		B4E2S3	cDNA FLJ56561	87 kDa	0.19	19	14	5	1	4	6	3	0	1	3	1	-2.179571127	0% (0.055)
ANO10_HUMAN	CAPR1_HUMAN	CAPRIN1	Q14444	Caprin-1 (Cell cycle-associated protein 1) (Cytoplasmic activation- and proliferation-associated protein 1) (GPI-anchored membrane protein 1) (GPI-anchored protein p137) (GPI-p137) (p137GPI) (Membrane component chromosome 11 surface marker 1) (RNA granule protein 105)	78 kDa	0.036	19	14	5	2	6	5	1	1	0	2	2	-2.179571127	0% (0.055)
AIFM2_HUMAN	MCM6_HUMAN	MCM6	Q14566	DNA replication licensing factor MCM6 (EC 3.6.4.12) (p105MCM)	93 kDa	0.072	26	19	7	0	9	11	0	5	1	1	0	-2.192641896	95% (0.030)
E9PE17_HUMAN (+3)	CHP1_HUMAN	CHP1	Q99653	Calcineurin B homologous protein 1 (Calcineurin B-like protein) (Calcium-binding protein CHP) (Calcium-binding protein p22) (EF-hand calcium-binding domain-containing protein p22)	22 kDa	0.14	33	24	9	12	3	4	5	1	3	1	3	-2.200642808	95% (0.016)

Q4G1C4_HUMAN (+1)	B4E3A8_HUMAN		B4E3A8	cDNA FLJ53963, highly similar to Leukocyte elastase inhibitor	39 kDa	0.066	64	47	18	15	7	12	13	1	4	3	10	-2.239498527	95% (0.0011)
PLP2_HUMAN	Q4AEJ3_HUMAN	hfzo1	Q4AEJ3	Mitochondrial transmembrane GTPase Fzo-1	84 kDa	0.18	23	17	6	7	4	4	2	3	1	1	2	-2.248622629	95% (0.033)
ARP5L_HUMAN (+1)	ATP5I_HUMAN	ATP5I	P56385	ATP synthase subunit e, mitochondrial (ATPase subunit e)	8 kDa	0.16	23	17	6	3	2	4	8	0	2	3	1	-2.248622629	95% (0.033)
CBX1_HUMAN (+1)	BPHL_HUMAN	BPHL	Q86WA6	Valacyclovir hydrolase (VACVase) (Valacyclovirase) (EC 3.1.-.-) (Biphenyl hydrolase-like protein) (Biphenyl hydrolase-related protein) (Bph-rp) (Breast epithelial mucin-associated antigen) (MCNAA)	33 kDa	0.24	23	17	6	3	1	5	8	0	0	1	5	-2.248622629	95% (0.033)
B4DXB6_HUMAN (+2)	A9CQZ8_HUMAN	TJP1	A9CQZ8	Tight junction protein ZO-1 (Fragment)	197 kDa	0.066	16	12	4	3	4	3	2	3	0	1	0	-2.254409889	0% (0.062)
OSBP1_HUMAN	S2540_HUMAN	SLC25A40	Q8TBP6	Solute carrier family 25 member 40 (Mitochondrial carrier family protein)	38 kDa	0.036	16	12	4	1	4	2	5	0	0	1	3	-2.254409889	0% (0.062)
A6NFN2_HUMAN (+7)	PDC10_HUMAN	PDCD10	Q9BUL8	Programmed cell death protein 10 (Cerebral cavernous malformations 3 protein) (TF-1 cell apoptosis-related protein 15)	25 kDa	0.053	16	12	4	1	4	1	7	2	1	0	1	-2.254409889	0% (0.062)
VSIG4_HUMAN	CMPK2_HUMAN	CMPK2	Q5EBM0	UMP-CMP kinase 2, mitochondrial (EC 2.7.4.14) (Nucleoside-diphosphate kinase) (EC 2.7.4.6)	49 kDa	0.068	16	12	4	0	1	1	10	0	4	0	0	-2.254409889	0% (0.062)
C9JLU1_HUMAN (+1)	WDR5_HUMAN	WDR5	P61964	WD repeat-containing protein 5 (BMP2-induced 3-kb gene protein)	37 kDa	0.26	16	12	4	1	4	3	4	1	0	1	2	-2.254409889	0% (0.062)
RPB7_HUMAN	E9PHY8_HUMAN	MROH1	E9PHY8	Maestro heat-like repeat-containing protein family member 1	180 kDa	0.17	9	7	2	5	0	0	2	1	0	1	0	-2.267417458	0% (0.12)
J3QK90_HUMAN (+3)	H0Y858_HUMAN		H0Y858	Uncharacterized protein (Fragment)	134 kDa	0.1	9	7	2	0	2	1	4	0	2	0	0	-2.267417458	0% (0.12)
B4DT01_HUMAN (+1)	B4DHN5_HUMAN	SDCBP	B4DHN5	Syntenin-1 (cDNA FLJ55055, moderately similar to Syntenin-1)	26 kDa	0.075	10	7	2	1	2	2	2	0	1	0	1	-2.267417458	0% (0.12)
FINC_HUMAN	Q53EL1_HUMAN		Q53EL1	Protein KIAA0196 variant (Fragment)	135 kDa	0.19	9	7	2	1	5	0	1	0	2	0	1	-2.267417458	0% (0.12)
M0QYR6_HUMAN (+2)	E5KSX8_HUMAN		E5KSX8	Mitochondrial transcription factor A	29 kDa	0.13	8	7	2	3	1	1	2	0	1	0	1	-2.267417458	0% (0.12)
CC127_HUMAN	B2RAN1_HUMAN		B2RAN1	cDNA, FLJ95012, highly similar to Homo sapiens UDP-glucose pyrophosphorylase 2 (UGP2), mRNA	57 kDa	0.1	9	7	2	0	1	1	5	1	1	0	0	-2.267417458	0% (0.12)
PSB5_HUMAN	AHSA1_HUMAN	AHSA1	O95433	Activator of 90 kDa heat shock protein ATPase homolog 1 (AHA1) (p38)	38 kDa	0.099	9	7	2	0	3	0	4	1	0	1	0	-2.267417458	0% (0.12)
MAN1_HUMAN	LTOR5_HUMAN	LAMTOR5	O43504	Ragulator complex protein LAMTOR5 (Hepatitis B virus X-interacting protein) (HBV X-interacting protein) (HBX-interacting protein) (Late endosomal/lysosomal adaptor and MAPK and MTOR activator 5)	10 kDa	0.027	9	7	2	3	2	2	1	0	0	1	1	-2.267417458	0% (0.12)
G9LJ35_HUMAN	F5H7V7_HUMAN	NDUFA8	F5H7V7	NADH dehydrogenase [ubiquinone] 1 alpha subcomplex subunit 8	15 kDa	0.043	9	7	2	3	1	1	2	0	1	1	0	-2.267417458	0% (0.12)
E9PLS9_HUMAN (+1)	AP1S1_HUMAN	AP1S1	P61966	AP-1 complex subunit sigma-1A (Adapter-related protein complex 1 subunit sigma-1A) (Adaptor protein complex AP-1 subunit sigma-1A) (Clathrin assembly protein complex 1 sigma-1A small chain) (Clathrin coat assembly protein AP19) (Golgi adaptor HA1/AP1 adaptin sigma-1A subunit) (HA1 19 kDa subunit)	19 kDa	0.11	9	7	2	2	2	2	1	1	1	0	0	-2.267417458	0% (0.12)

				(Sigma 1a subunit of AP-1 clathrin) (Sigma-adaptin 1A) (Sigma1A-adaptin)															
RRAGA_HUMAN	BAX_HUMAN	BAX	Q07812	Apoptosis regulator BAX (Bcl-2-like protein 4) (Bcl2-L-4)	21 kDa	0.083	9	7	2	2	0	0	5	0	1	1	0	-2.267417458	0% (0.12)
SNTB1_HUMAN	B4DND4_HUMAN		B4DND4	cDNA FLJ50588, highly similar to Gamma-glutamyltransferase 5 (EC 2.3.2.2)	54 kDa	0.08	9	7	2	0	4	3	0	0	0	2	0	-2.267417458	0% (0.12)
B2R5R5_HUMAN (+4)	E9PIM6_HUMAN	THY1	E9PIM6	Thy-1 membrane glycoprotein (Fragment)	17 kDa	0.086	9	7	2	7	0	0	0	0	0	0	2	-2.267417458	0% (0.12)
SIA4A_HUMAN	B3AT_HUMAN	SLC4A1	P02730	Band 3 anion transport protein (Anion exchange protein 1) (AE 1) (Anion exchanger 1) (Solute carrier family 4 member 1) (CD antigen CD233)	102 kDa	0.076	87	63	24	3	27	26	7	5	2	9	8	-2.273776595	95% (0.00016)
HD_HUMAN	E7EVP7_HUMAN	ITPR1	E7EVP7	Inositol 1,4,5-trisphosphate receptor type 1	312 kDa	0.0083	34	25	9	14	4	7	0	1	0	1	7	-2.287826382	95% (0.012)
B3KNI8_HUMAN	GOGA4_HUMAN	GOLGA4	Q13439	Golgin subfamily A member 4 (256 kDa golgin) (Golgin-245) (Protein 72.1) (Trans-Golgi p230)	261 kDa	0.053	2	2	0	0	1	1	0	0	0	0	0	-2.322302773	0% (0.28)
B4DVV7_HUMAN (+1)	DOCK2_HUMAN	DOCK2	Q92608	Dedicator of cytokinesis protein 2	212 kDa	0.04	2	2	0	0	1	1	0	0	0	0	0	-2.322302773	0% (0.28)
B5TY32_HUMAN (+2)	CDR2L_HUMAN	CDR2L	Q86X02	Cerebellar degeneration-related protein 2-like (Paraneoplastic 62 kDa antigen)	53 kDa	0.09	2	2	0	0	0	2	0	0	0	0	0	-2.322302773	0% (0.28)
ODR4_HUMAN	RAI14_HUMAN	RAI14	Q9P0K7	Ankycorbin (Ankyrin repeat and coiled-coil structure-containing protein) (Novel retinal pigment epithelial cell protein) (Retinoic acid-induced protein 14)	110 kDa	0.086	2	2	0	1	0	1	0	0	0	0	0	-2.322302773	0% (0.28)
RB27B_HUMAN	AFAP1_HUMAN	AFAP1	Q8N556	Actin filament-associated protein 1 (110 kDa actin filament-associated protein) (AFAP-110)	81 kDa	0.27	2	2	0	0	0	2	0	0	0	0	0	-2.322302773	0% (0.28)
A8K4T2_HUMAN (+1)	PKD2_HUMAN	PKD2	Q13563	Polycystin-2 (Autosomal dominant polycystic kidney disease type II protein) (Polycystic kidney disease 2 protein) (Polycystwin) (R48321)	110 kDa	0.074	2	2	0	2	0	0	0	0	0	0	0	-2.322302773	0% (0.28)
Q53HE3_HUMAN (+1)	A6H8W6_HUMAN	SIPA1L1	A6H8W6	SIPA1L1 protein	200 kDa	0.12	2	2	0	0	1	1	0	0	0	0	0	-2.322302773	0% (0.28)
B2R6E2_HUMAN (+2)	RGPA2_HUMAN	RALGAPA2	Q2PPJ7	Ral GTPase-activating protein subunit alpha-2 (250 kDa substrate of Akt) (AS250) (p220)	211 kDa	0.1	2	2	0	2	0	0	0	0	0	0	0	-2.322302773	0% (0.28)
B2R9X3_HUMAN (+1)	SSFA2_HUMAN	SSFA2	P28290	Sperm-specific antigen 2 (Cleavage signal-1 protein) (CS-1) (Ki-ras-induced actin-interacting protein)	138 kDa	0.067	2	2	0	2	0	0	0	0	0	0	0	-2.322302773	0% (0.28)
E9PKF6_HUMAN (+3)	B4DI03_HUMAN	SEC11L3	B4DI03	SEC11-like 3 (S. cerevisiae), isoform CRA_a (cDNA FLJ57851, highly similar to Microsomal signal peptidase 21 kDa subunit (EC 3.4.-.-))	17 kDa	0.045	2	2	0	2	1	0	0	0	0	0	0	-2.322302773	0% (0.28)
E7ESL0_HUMAN (+2)	B5TY32_HUMAN	MAPK14	B5TY32	Mitogen-activated protein kinase 14 isoform 4	29 kDa	0.11	3	2	0	0	1	0	2	0	0	0	0	-2.322302773	0% (0.28)
B4DZK0_HUMAN	J3KNA0_HUMAN	OXA1L	J3KNA0	Mitochondrial inner membrane protein OXA1L	55 kDa	0.086	2	2	0	0	0	0	2	0	0	0	0	-2.322302773	0% (0.28)
A8KAM9_HUMAN (+2)	VAV2_HUMAN	VAV2	P52735	Guanine nucleotide exchange factor VAV2 (VAV-2)	101 kDa	0.096	2	2	0	0	1	1	0	0	0	0	0	-2.322302773	0% (0.28)
OSBL3_HUMAN	B7Z1V3_HUMAN		B7Z1V3	cDNA FLJ54733, highly similar to General transcription factor 3C polypeptide 5	46 kDa	0.065	2	2	0	0	0	0	2	0	0	0	0	-2.322302773	0% (0.28)

IF44L_HUMAN	F5H2X3_HUMAN	NRP2	F5H2X3	Neuropilin-2	102 kDa	0.11	2	2	0	0	1	1	0	0	0	0	0	-2.322302773	0% (0.28)
B7Z7Y3_HUMAN (+1)	TIG1_HUMAN	RARRES1	P49788	Retinoic acid receptor responder protein 1 (RAR-responsive protein TIG1) (Tazarotene-induced gene 1 protein)	33 kDa	0.06	2	2	0	0	2	0	0	0	0	0	0	-2.322302773	0% (0.28)
B2R880_HUMAN (+1)	NCKPL_HUMAN	NCKAP1L	P55160	Nck-associated protein 1-like (Hematopoietic protein 1) (Membrane-associated protein HEM-1)	128 kDa	0.083	2	2	0	0	2	0	0	0	0	0	0	-2.322302773	0% (0.28)
CI142_HUMAN	GPAT4_HUMAN	AGPAT6	Q86UL3	Glycerol-3-phosphate acyltransferase 4 (GPAT4) (EC 2.3.1.15) (1-acylglycerol-3-phosphate O-acyltransferase 6) (1-AGP acyltransferase 6) (1-AGPAT 6) (Acyl-CoA:glycerol-3-phosphate acyltransferase 4) (Lysophosphatidic acid acyltransferase zeta) (LPAAT-zeta)	52 kDa	0.15	2	2	0	2	0	0	0	0	0	0	0	-2.322302773	0% (0.28)
B2RAW1_HUMAN (+1)	G3V3Y1_HUMAN		G3V3Y1	Uncharacterized protein	32 kDa	0.018	2	2	0	0	0	0	2	0	0	0	0	-2.322302773	0% (0.28)
BYST_HUMAN	E9PHI6_HUMAN	DYNC1LI1	E9PHI6	Cytoplasmic dynein 1 light intermediate chain 1	43 kDa	0.053	2	2	0	0	0	1	1	0	0	0	0	-2.322302773	0% (0.28)
SRPK2_HUMAN	PAR6B_HUMAN	PARD6B	Q9BYG5	Partitioning defective 6 homolog beta (PAR-6 beta) (PAR-6B)	41 kDa	0.044	2	2	0	0	0	1	1	0	0	0	0	-2.322302773	0% (0.28)
E9PN70_HUMAN (+1)	E7EQU1_HUMAN	HMGB3	E7EQU1	High mobility group protein B3 (Fragment)	22 kDa	0.14	2	2	0	1	0	0	1	0	0	0	0	-2.322302773	0% (0.28)
GNPI1_HUMAN	ETUD1_HUMAN	EFTUD1	Q7Z2Z2	Elongation factor Tu GTP-binding domain-containing protein 1 (Elongation factor-like 1) (Protein FAM42A)	125 kDa	0.14	2	2	0	0	0	0	2	0	0	0	0	-2.322302773	0% (0.28)
B1ALI9_HUMAN	CBL_HUMAN	CBL	P22681	E3 ubiquitin-protein ligase CBL (EC 6.3.2.-) (Casitas B-lineage lymphoma proto-oncogene) (Proto-oncogene c-Cbl) (RING finger protein 55) (Signal transduction protein CBL)	100 kDa	0.089	2	2	0	0	1	2	0	0	0	0	0	-2.322302773	0% (0.28)
B4E2I4_HUMAN (+1)	B7Z6C9_HUMAN		B7Z6C9	cDNA FLJ61658, highly similar to Transmembrane 9 superfamily protein member 1	92 kDa	0.05	2	2	0	0	1	1	0	0	0	0	0	-2.322302773	0% (0.28)
IKIP_HUMAN	ATLA1_HUMAN	ATL1	Q8WXF7	Atlastin-1 (EC 3.6.5.-) (Brain-specific GTP-binding protein) (GTP-binding protein 3) (GBP-3) (hGBP3) (Guanine nucleotide-binding protein 3) (Spastic paraplegia 3 protein A)	64 kDa	0.071	2	2	0	2	0	0	0	0	0	0	0	-2.322302773	0% (0.28)
ADCK3_HUMAN (+4)	A8K2S1_HUMAN		A8K2S1	cDNA FLJ75258, highly similar to Homo sapiens phospholipase A2, group IVA (cytosolic, calcium-dependent) (PLA2G4A), mRNA	85 kDa	0.04	2	2	0	0	2	0	0	0	0	0	0	-2.322302773	0% (0.28)
PAG15_HUMAN	SSH3_HUMAN	SSH3	Q8TE77	Protein phosphatase Slingshot homolog 3 (EC 3.1.3.16) (EC 3.1.3.48) (SSH-like protein 3) (SSH-3L) (hSSH-3L)	73 kDa	0.061	2	2	0	0	0	0	2	0	0	0	0	-2.322302773	0% (0.28)
VP33B_HUMAN	D6REB4_HUMAN	PAIP1	D6REB4	Polyadenylate-binding protein-interacting protein 1 (Uncharacterized protein)	40 kDa	0.044	2	2	0	0	1	1	0	0	0	0	0	-2.322302773	0% (0.28)
GHDC_HUMAN (+1)	G3ST2_HUMAN	GAL3ST2	Q9H3Q3	Galactose-3-O-sulfotransferase 2 (Gal3ST-2) (EC 2.8.2.-) (Beta-galactose-3-O-sulfotransferase 2) (Gal-beta-1, 3-GalNAc 3'-sulfotransferase 2) (Glycoprotein beta-Gal 3'-sulfotransferase 2)	46 kDa	0.043	2	2	0	1	0	1	0	0	0	0	0	-2.322302773	0% (0.28)
A6NIR4_HUMAN (+3)	FGFP1_HUMAN	FGFBP1	Q14512	Fibroblast growth factor-binding protein 1 (FGF-BP) (FGF-BP1) (FGF-binding protein 1) (FGFBP-1) (17 kDa	26 kDa	0.048	2	2	0	0	1	1	0	0	0	0	0	-2.322302773	0% (0.28)

				heparin-binding growth factor-binding protein) (17 kDa HBGF-binding protein) (HBp17)															
F8W8C2_HUMAN	BSPRY_HUMAN	BSPRY	Q5W0U4	B box and SPRY domain-containing protein	44 kDa	0.033	2	2	0	2	0	0	0	0	0	0	0	-2.322302773	0% (0.28)
PR38B_HUMAN	B2RAH2_HUMAN		B2RAH2	Sodium/hydrogen exchanger	91 kDa	0.046	2	2	0	2	0	0	0	0	0	0	0	-2.322302773	0% (0.28)
B2RAY1_HUMAN (+1)	A5D8Z7_HUMAN	SIGLEC1	A5D8Z7	SIGLEC1 protein (Fragment)	180 kDa	0.072	2	2	0	0	0	1	0	0	0	0	0	-2.322302773	0% (0.28)
TRM6_HUMAN	VPS25_HUMAN	VPS25	Q9BRG1	Vacuolar protein-sorting-associated protein 25 (hVps25) (Dermal papilla-derived protein 9) (ELL-associated protein of 20 kDa) (ESCRT-II complex subunit VPS25)	21 kDa	0.054	2	2	0	0	0	0	2	0	0	0	0	-2.322302773	0% (0.28)
A6PVN5_HUMAN (+1)	G3V145_HUMAN	SDR16C5	G3V145	Epidermal retinol dehydrogenase 2 (Retinal short chain dehydrogenase reductase isoform 1, isoform CRA_c)	35 kDa	0.07	2	2	0	2	0	0	0	0	0	0	0	-2.322302773	0% (0.28)
H0YBJ4_HUMAN (+2)	MZB1_HUMAN	MZB1	Q8WU39	Marginal zone B- and B1-cell-specific protein (Mesenteric estrogen-dependent adipose 7) (MEDA-7) (Plasma cell-induced resident endoplasmic reticulum protein) (Plasma cell-induced resident ER protein) (pERp1) (Proapoptotic caspase adapter protein)	21 kDa	0.036	2	2	0	0	1	1	0	0	0	0	0	-2.322302773	0% (0.28)
F208A_HUMAN	F8WCD0_HUMAN	RNF149	F8WCD0	E3 ubiquitin-protein ligase RNF149	43 kDa	0.009	2	2	0	0	1	0	1	0	0	0	0	-2.322302773	0% (0.28)
Q59ET0_HUMAN	B2RE38_HUMAN		B2RE38	cDNA, FLJ96906, highly similar to Homo sapiens solute carrier family 17 (anion/sugar transporter), member 5 (SLC17A5), mRNA	55 kDa	0.036	2	2	0	2	0	0	0	0	0	0	0	-2.322302773	0% (0.28)
F263_HUMAN	C9K0K7_HUMAN	PRPSAP2	C9K0K7	Phosphoribosyl pyrophosphate synthase-associated protein 2 (Fragment)	27 kDa	0.017	2	2	0	0	1	1	0	0	0	0	0	-2.322302773	0% (0.28)
B4DRD3_HUMAN (+2)	CDS1_HUMAN	CDS1	Q92903	Phosphatidate cytidylyltransferase 1 (EC 2.7.7.41) (CDP-DAG synthase 1) (CDP-DG synthase 1) (CDP-diacylglycerol synthase 1) (CDS 1) (CDP-diglyceride pyrophosphorylase 1) (CDP-diglyceride synthase 1) (CTP:phosphatidate cytidylyltransferase 1)	53 kDa	0.048	2	2	0	2	0	0	0	0	0	0	0	-2.322302773	0% (0.28)
TM260_HUMAN	TP4A2_HUMAN	PTP4A2	Q12974	Protein tyrosine phosphatase type IVA 2 (EC 3.1.3.48) (HU-PP-1) (OV-1) (PTP(CAAXII)) (Protein-tyrosine phosphatase 4a2) (Protein-tyrosine phosphatase of regenerating liver 2) (PRL-2)	19 kDa	0.045	2	2	0	1	1	0	0	0	0	0	0	-2.322302773	0% (0.28)
J3KNA0_HUMAN (+2)	B4DKQ5_HUMAN		B4DKQ5	cDNA FLJ54710, highly similar to Target of Myb protein 1	55 kDa	0.044	21	15	5	7	3	5	0	0	1	1	3	-2.322523669	95% (0.038)
PTPRK_HUMAN	E3SWK8_HUMAN	HLA-C	E3SWK8	MHC class I antigen (Fragment)	21 kDa	0.019	38	28	10	0	8	0	12	0	0	4	0	-2.322744638	95% (0.0073)
FN3K_HUMAN	CD82_HUMAN	CD82	P27701	CD82 antigen (C33 antigen) (IA4) (Inducible membrane protein R2) (Metastasis suppressor Kangai-1) (Suppressor of tumorigenicity 6 protein) (Tetraspanin-27) (Tspan-27) (CD antigen CD82)	30 kDa	0.1	60	44	16	13	12	15	4	5	1	5	5	-2.343739988	95% (0.00097)
B3KUJ0_HUMAN (+2)	B3KX23_HUMAN		B3KX23	cDNA FLJ44516 fis, clone UTERU3002600, highly similar to Homo sapiens	73 kDa	0.073	13	10	3	1	4	2	3	0	0	0	3	-2.364476406	0% (0.070)

				DEAD (Asp-Glu-Ala-Asp) box polypeptide 42 (DDX42), transcript variant 2, mRNA														
A8MVT4_HUMAN (+1)	OAS2_HUMAN	OAS2	P29728	2'-5'-oligoadenylate synthase 2 ((2-5')oligo(A) synthase 2) (2-5A synthase 2) (EC 2.7.7.84) (p69 OAS / p71 OAS) (p69OAS / p71OAS)	82 kDa	0.14	28	21	7	0	2	2	17	0	7	0	0	-2.409261043	95% (0.014)
GEMI4_HUMAN (+4)	DPYL2_HUMAN	DPYSL2	Q16555	Dihydropyrimidinase-related protein 2 (DRP-2) (Collapsin response mediator protein 2) (CRMP-2) (N2A3) (Unc-33-like phosphoprotein 2) (ULIP-2)	62 kDa	0.014	28	21	7	2	11	8	0	0	2	2	0	-2.409261043	95% (0.014)
UB2J1_HUMAN	TOR1B_HUMAN	TOR1B	O14657	Torsin-1B (Torsin family 1 member B)	38 kDa	0.063	17	13	4	0	2	2	9	0	1	0	3	-2.424585187	95% (0.042)
B4DFR2_HUMAN	ABHEB_HUMAN	ABHD14B	Q96IU4	Alpha/beta hydrolase domain-containing protein 14B (Abhydrolase domain-containing protein 14B) (EC 3.-.-.-) (CCG1-interacting factor B)	22 kDa	0.23	17	13	4	2	3	3	5	0	3	1	0	-2.424585187	95% (0.042)
B7Z4A7_HUMAN (+3)	AP2M1_HUMAN	AP2M1	Q96CW1	AP-2 complex subunit mu (AP-2 mu chain) (Adapter-related protein complex 2 subunit mu) (Adaptin-mu2) (Adaptor protein complex AP-2 subunit mu) (Clathrin assembly protein complex 2 mu medium chain) (Clathrin coat assembly protein AP50) (Clathrin coat-associated protein AP50) (HA2 50 kDa subunit) (Plasma membrane adaptor AP-2 50 kDa protein)	50 kDa	0.065	42	32	11	10	9	5	9	3	1	3	4	-2.424933084	95% (0.0032)
B2R6Q2_HUMAN (+2)	PLMN_HUMAN	PLG	P00747	Plasminogen (EC 3.4.21.7) [Cleaved into: Plasmin heavy chain A; Activation peptide; Angiostatin; Plasmin heavy chain A, short form; Plasmin light chain B]	91 kDa	0.04	6	5	1	0	1	0	4	1	0	0	0	-2.481152651	0% (0.14)
A8K559_HUMAN (+1)	Q5JW30_HUMAN	STAU1	Q5JW30	Double-stranded RNA-binding protein Staufen homolog 1	55 kDa	0.064	6	5	1	0	3	2	0	0	0	1	0	-2.481152651	0% (0.14)
PLAP_HUMAN	A8MQB8_HUMAN	FMR1	A8MQB8	Fragile X mental retardation protein 1	66 kDa	0.082	6	5	1	1	1	0	3	1	0	0	0	-2.481152651	0% (0.14)
ADHX_HUMAN (+2)	68MP_HUMAN	MP68	P56378	6.8 kDa mitochondrial proteolipid	7 kDa	0.11	6	5	1	2	1	1	1	0	1	0	0	-2.481152651	0% (0.14)
Q52NV4_HUMAN (+1)	G3XAI2_HUMAN	LAMB1	G3XAI2	Laminin subunit beta-1 (Laminin, beta 1, isoform CRA_a)	200 kDa	0.12	6	5	1	1	2	2	0	0	0	1	0	-2.481152651	0% (0.14)
AURKA_HUMAN (+2)	B3KSR7_HUMAN		B3KSR7	cDNA FLJ36831 fis, clone ASTRO2010615, highly similar to Calpain-5 (EC 3.4.22.-)	64 kDa	0.11	6	5	1	4	1	0	0	0	1	0	0	-2.481152651	0% (0.14)
F6IQV5_HUMAN	A8K693_HUMAN		A8K693	cDNA FLJ75713, highly similar to Homo sapiens kynurenine 3-monooxygenase (kynurenine 3-hydroxylase) (KMO), mRNA	56 kDa	0.37	6	5	1	0	4	2	0	0	0	0	1	-2.481152651	0% (0.14)
M4QNU4_HUMAN	F5H5M9_HUMAN	TNIK	F5H5M9	TRAF2 and NCK-interacting protein kinase	150 kDa	0.46	6	5	1	2	1	1	0	0	0	0	1	-2.481152651	0% (0.14)
B4DZX1_HUMAN (+1)	H7C417_HUMAN		H7C417	Uncharacterized protein (Fragment)	37 kDa	0.023	6	5	1	1	1	1	2	1	0	0	0	-2.481152651	0% (0.14)
DPOD1_HUMAN (+2)	B4DVY1_HUMAN	EIF3D	B4DVY1	Eukaryotic translation initiation factor 3 subunit D (eIF3d) (Eukaryotic translation initiation factor 3 subunit 7) (eIF-3-zeta)	58 kDa	0.022	6	5	1	0	0	3	2	0	1	0	0	-2.481152651	0% (0.14)
B2R745_HUMAN	RASM_HUMAN	MRAS	O14807	Ras-related protein M-Ras (Ras-related protein R-Ras3)	24 kDa	0.051	6	5	1	0	2	2	0	0	0	0	2	-2.481152651	0% (0.14)

SYUG_HUMAN	M0QYZ2_HUMAN	AP2S1	M0QYZ2	AP-2 complex subunit sigma	19 kDa	0.12	6	5	1	0	0	2	3	0	0	1	0	-2.481152651	0% (0.14)
KIF1A_HUMAN	A2ACR1_HUMAN	PSMB9	A2ACR1	Proteasome subunit beta type (EC 3.4.25.1)	21 kDa	0.012	6	5	1	0	1	2	2	0	0	1	0	-2.481152651	0% (0.14)
A8K8C2_HUMAN (+2)	F210B_HUMAN	FAM210B	Q96KR6	Protein FAM210B	20 kDa	0.055	6	5	1	2	2	0	1	0	1	0	0	-2.481152651	0% (0.14)
RAB7B_HUMAN	RPP30_HUMAN	RPP30	P78346	Ribonuclease P protein subunit p30 (RNaseP protein p30) (EC 3.1.26.5) (RNase P subunit 2)	29 kDa	0.2	7	5	1	0	1	1	4	0	0	1	0	-2.481152651	0% (0.14)
Q658W7_HUMAN	Q4G1C4_HUMAN	ZADH2	Q4G1C4	ZADH2 protein (Fragment)	39 kDa	0.037	6	5	1	1	1	1	2	1	0	0	0	-2.481152651	0% (0.14)
IGHM_HUMAN (+1)	RPB7_HUMAN	POLR2G	P62487	DNA-directed RNA polymerase II subunit RPB7 (RNA polymerase II subunit B7) (DNA-directed RNA polymerase II subunit G) (RNA polymerase II 19 kDa subunit) (RPB19)	19 kDa	0.066	6	5	1	0	3	1	1	0	0	1	0	-2.481152651	0% (0.14)
H3BRY5_HUMAN (+1)	B2R6E2_HUMAN		B2R6E2	cDNA, FLJ92910, highly similar to Homo sapiens programmed cell death 4 (neoplastic transformation inhibitor) (PDCD4), transcript variant 1, mRNA	52 kDa	0.14	6	5	1	0	0	0	5	1	0	0	0	-2.481152651	0% (0.14)
D6RDJ3_HUMAN (+3)	RASL1_HUMAN	RASAL1	O95294	RasGAP-activating-like protein 1	90 kDa	0.23	25	19	6	19	0	0	0	0	0	0	6	-2.495111155	95% (0.016)
B3KUB8_HUMAN (+7)	B4DGM9_HUMAN	TOR1A	B4DGM9	Torsin family 1, member A (Torsin A), isoform CRA_a (cDNA FLJ56343, highly similar to Torsin A)	41 kDa	0.055	44	33	11	8	8	4	14	0	4	3	4	-2.497895508	95% (0.0022)
ISOC1_HUMAN	5NTD_HUMAN	NT5E	P21589	5'-nucleotidase (5'-NT) (EC 3.1.3.5) (Ecto-5'-nucleotidase) (CD antigen CD73)	63 kDa	0.13	29	22	7	7	9	7	0	0	3	2	2	-2.517574792	95% (0.0098)
F8WBK5_HUMAN (+1)	S10A8_HUMAN	S100A8	P05109	Protein S100-A8 (Calgranulin-A) (Calprotectin L1L subunit) (Cystic fibrosis antigen) (CFAG) (Leukocyte L1 complex light chain) (Migration inhibitory factor-related protein 8) (MRP-8) (p8) (S100 calcium-binding protein A8) (Urinary stone protein band A) [Cleaved into: Protein S100-A8, N-terminally processed]	11 kDa	0.15	71	53	18	1	22	20	10	0	5	5	8	-2.518179627	95% (0.00014)
ERD21_HUMAN	ITPR3_HUMAN	ITPR3	Q14573	Inositol 1,4,5-trisphosphate receptor type 3 (IP3 receptor isoform 3) (IP3R 3) (InsP3R3) (Type 3 inositol 1,4,5-trisphosphate receptor) (Type 3 InsP3 receptor)	304 kDa	0.1	185	138	48	85	21	29	5	6	0	32	10	-2.528098693	95% (< 0.00010)
VPS29_HUMAN	A8K964_HUMAN		A8K964	cDNA FLJ75071, highly similar to Homo sapiens pinin, desmosome associated protein (PNN), mRNA	82 kDa	0.2	10	8	2	1	1	3	3	0	0	1	1	-2.542288604	0% (0.078)
B3KN09_HUMAN (+1)	FIBB_HUMAN	FGB	P02675	Fibrinogen beta chain [Cleaved into: Fibrinopeptide B; Fibrinogen beta chain]	56 kDa	0.076	10	8	2	0	0	0	8	0	0	1	1	-2.542288604	0% (0.078)
EDC3_HUMAN	PSA2_HUMAN	PSMA2	P25787	Proteasome subunit alpha type-2 (EC 3.4.25.1) (Macropain subunit C3) (Multicatalytic endopeptidase complex subunit C3) (Proteasome component C3)	26 kDa	0.061	10	8	2	0	2	2	4	0	1	2	0	-2.542288604	0% (0.078)
B2R8N1_HUMAN (+2)	NCKP1_HUMAN	NCKAP1	Q9Y2A7	Nck-associated protein 1 (NAP 1) (Membrane-associated protein HEM-2) (p125Nap1)	129 kDa	0.14	37	28	9	11	5	6	5	2	3	1	2	-2.549390548	95% (0.0038)
A8K6B4_HUMAN (+3)	B2R4C1_HUMAN		B2R4C1	cDNA, FLJ92036, highly similar to Homo sapiens ribosomal protein L31 (RPL31), mRNA	14 kDa	0.12	14	11	3	2	3	2	4	1	1	0	1	-2.57468517	95% (0.046)
GBRL2_HUMAN (+1)	AN32A_HUMAN	ANP32A	P39687	Acidic leucine-rich nuclear phosphoprotein 32 family member A (Acidic nuclear	29 kDa	0.2	14	11	3	1	3	1	7	1	0	2	0	-2.57468517	95% (0.046)

				phosphoprotein pp32) (Leucine-rich acidic nuclear protein) (LANP) (Mapmodulin) (Potent heat-stable protein phosphatase 2A inhibitor I1PP2A) (Putative HLA-DR-associated protein I) (PHAPI)														
SYPM_HUMAN	ARL8A_HUMAN	ARL8A	Q96BM9	ADP-ribosylation factor-like protein 8A (ADP-ribosylation factor-like protein 10B) (Novel small G protein indispensable for equal chromosome segregation 2)	21 kDa	0.061	53	40	13	14	10	8	8	0	4	3	6	-2.586338998	95% (0.00063)
A8KAL2_HUMAN (+2)	A8K2I7_HUMAN		A8K2I7	cDNA FLJ76072, highly similar to Homo sapiens GIPC PDZ domain containing family, member 1 (GIPC1), transcript variant 1, mRNA	36 kDa	0.041	18	14	4	4	1	1	8	2	0	0	2	-2.594764859	95% (0.028)
B4DZ28_HUMAN (+1)	EXOS2_HUMAN	EXOSC2	Q13868	Exosome complex component RRP4 (Exosome component 2) (Ribosomal RNA-processing protein 4)	33 kDa	0.061	18	14	4	0	4	4	6	1	1	2	0	-2.594764859	95% (0.028)
GDPD1_HUMAN	AGO2_HUMAN	AGO2	Q9UKV8	Protein argonaute-2 (Argonaute2) (hAgo2) (EC 3.1.26.n2) (Argonaute RISC catalytic component 2) (Eukaryotic translation initiation factor 2C 2) (eIF-2C 2) (eIF2C 2) (PAZ Piwi domain protein) (PPD) (Protein slicer)	97 kDa	0.1	18	14	4	0	6	8	1	1	0	2	1	-2.594764859	95% (0.028)
PLXB1_HUMAN	EHD1_HUMAN	EHD1	Q9H4M9	EH domain-containing protein 1 (PAST homolog 1) (hPAST1) (Testilin)	61 kDa	0.02	18	14	4	5	4	1	4	0	2	2	0	-2.594764859	95% (0.028)
APOBR_HUMAN (+1)	Q0PNF2_HUMAN		Q0PNF2	FEX1	275 kDa	0.023	22	17	5	0	7	9	0	0	0	1	4	-2.608439774	95% (0.017)
E3SWK8_HUMAN	GSHR_HUMAN	GSR	P00390	Glutathione reductase, mitochondrial (GR) (GRase) (EC 1.8.1.7)	56 kDa	0.37	22	17	5	2	7	7	1	1	0	2	2	-2.608439774	95% (0.017)
Q53R32_HUMAN (+1)	STX7_HUMAN	STX7	O15400	Syntaxin-7	30 kDa	0.18	26	20	6	9	4	4	3	0	0	2	4	-2.618360169	95% (0.011)
Q53F88_HUMAN (+1)	HPCL1_HUMAN	HPCAL1	P37235	Hippocalcin-like protein 1 (Calcium-binding protein BDR-1) (HLP2) (Visinin-like protein 3) (VILIP-3)	22 kDa	0.078	30	23	7	5	7	9	2	0	1	3	3	-2.625891325	95% (0.0066)
FAKD5_HUMAN	HMGB1_HUMAN	HMGB1	P09429	High mobility group protein B1 (High mobility group protein 1) (HMG-1)	25 kDa	0.034	38	29	9	8	5	5	11	2	1	1	5	-2.636583085	95% (0.0026)
B7ZLX5_HUMAN (+1)	A8K274_HUMAN		A8K274	cDNA FLJ78227, highly similar to Homo sapiens pituitary tumor-transforming 1 interacting protein (PTTG1IP), mRNA	20 kDa	0.0071	46	35	11	7	9	10	9	6	2	3	0	-2.643825983	95% (0.0011)
TM38B_HUMAN	ITA2_HUMAN	ITGA2	P17301	Integrin alpha-2 (CD49 antigen-like family member B) (Collagen receptor) (Platelet membrane glycoprotein Ia) (GPIa) (VLA-2 subunit alpha) (CD antigen CD49b)	129 kDa	0.089	31	24	7	10	7	7	0	0	0	0	7	-2.734210642	95% (0.0045)
MSPD2_HUMAN (+1)	BAIP2_HUMAN	BAIAP2	Q9UQB8	Brain-specific angiogenesis inhibitor 1-associated protein 2 (BAI-associated protein 2) (BAI1-associated protein 2) (Protein BAP2) (Fas ligand-associated factor 3) (FLAF3) (Insulin receptor substrate p53/p58) (IRS-58) (IRSp53/58) (Insulin receptor substrate protein of 53 kDa) (IRSp53) (Insulin receptor substrate p53)	61 kDa	0.039	27	21	6	8	4	4	5	1	0	3	2	-2.741612351	95% (0.0071)
CC115_HUMAN	TMX4_HUMAN	TMX4	Q9H1E5	Thioredoxin-related transmembrane protein 4 (Thioredoxin domain-containing protein 13)	39 kDa	0.14	27	21	6	8	3	5	5	0	1	2	3	-2.741612351	95% (0.0071)

C9K0W8_HUMAN	B7Z4A1_HUMAN		B7Z4A1	cDNA FLJ50798, weakly similar to Ubiquinol-cytochrome c reductase complex ubiquinone-binding protein QP-C (EC 1.10.2.2)	8 kDa	0.18	19	15	4	2	4	3	6	1	1	0	2	-2.764948904	95% (0.018)
GRAM4_HUMAN	EFHD1_HUMAN	EFHD1	Q9BUP0	EF-hand domain-containing protein D1 (EF-hand domain-containing protein 1) (Swiprosin-2)	27 kDa	0.078	19	15	4	0	6	7	2	0	2	1	1	-2.764948904	95% (0.018)
FA10_HUMAN	J3QKR5_HUMAN	CDK11B	J3QKR5	Cyclin-dependent kinase 11B	90 kDa	0.041	15	12	3	0	3	4	5	1	0	1	1	-2.784899336	95% (0.030)
A8K2W6_HUMAN (+1)	GNL1_HUMAN	GNL1	P36915	Guanine nucleotide-binding protein-like 1 (GTP-binding protein HSR1)	69 kDa	0.068	15	12	3	0	1	2	9	1	1	1	0	-2.784899336	95% (0.030)
HEAT2_HUMAN	PLIN3_HUMAN	PLIN3	O60664	Perilipin-3 (47 kDa mannose 6-phosphate receptor-binding protein) (47 kDa MPR-binding protein) (Cargo selection protein TIP47) (Mannose-6-phosphate receptor-binding protein 1) (Placental protein 17) (PP17)	47 kDa	0.035	15	12	3	8	1	1	2	0	0	0	3	-2.784899336	95% (0.030)
C109B_HUMAN	B4DG22_HUMAN	RPS6KA3	B4DG22	Ribosomal protein S6 kinase alpha-3 (cDNA FLJ56618, highly similar to Ribosomal protein S6 kinase alpha-3 (EC 2.7.11.1))	81 kDa	0.074	11	9	2	0	4	4	1	1	1	0	0	-2.817166813	95% (0.050)
RABP1_HUMAN	ATAD2_HUMAN	ATAD2	Q6PL18	ATPase family AAA domain-containing protein 2 (EC 3.6.1.3) (AAA nuclear coregulator cancer-associated protein) (ANCCA)	159 kDa	0.18	11	9	2	0	4	5	0	0	0	2	0	-2.817166813	95% (0.050)
RS28_HUMAN	CCD51_HUMAN	CCDC51	Q96ER9	Coiled-coil domain-containing protein 51	46 kDa	0.3	11	9	2	1	0	0	8	0	0	0	2	-2.817166813	95% (0.050)
MRP3_HUMAN (+1)	H1X_HUMAN	H1FX	Q92522	Histone H1x	22 kDa	0.02	11	9	2	0	2	3	4	0	0	0	2	-2.817166813	95% (0.050)
SPRY7_HUMAN	STING_HUMAN	TMEM173	Q86WV6	Stimulator of interferon genes protein (hSTING) (Endoplasmic reticulum interferon stimulator) (ERIS) (Mediator of IRF3 activation) (hMITA) (Transmembrane protein 173)	42 kDa	0.17	32	25	7	8	5	1	11	0	2	2	3	-2.842532744	95% (0.0030)
B7Z7N6_HUMAN (+1)	B8QFA1_HUMAN	PKP2	B8QFA1	Plakophilin-2 transcript variant 2b	97 kDa	0.072	7	6	1	2	1	0	3	0	1	0	0	-2.878174056	0% (0.084)
DDX56_HUMAN (+2)	NFKB1_HUMAN	NFKB1	P19838	Nuclear factor NF-kappa-B p105 subunit (DNA-binding factor KBF1) (EBP-1) (Nuclear factor of kappa light polypeptide gene enhancer in B-cells 1) [Cleaved into: Nuclear factor NF-kappa-B p50 subunit]	105 kDa	0.037	7	6	1	2	1	2	1	0	0	0	1	-2.878174056	0% (0.084)
PLPL4_HUMAN	B4E0E0_HUMAN		B4E0E0	cDNA FLJ54898, highly similar to Homo sapiens transmembrane protein 41B (TMEM41B), mRNA	33 kDa	0.26	8	6	1	3	1	2	0	0	0	1	0	-2.878174056	0% (0.084)
B3KNF4_HUMAN (+2)	G5E9A6_HUMAN	USP11	G5E9A6	Ubiquitin carboxyl-terminal hydrolase (EC 3.4.19.12)	105 kDa	0.056	7	6	1	0	0	0	6	0	0	1	0	-2.878174056	0% (0.084)
MRRP3_HUMAN	WDFY1_HUMAN	WDFY1	Q8IWB7	WD repeat and FYVE domain-containing protein 1 (FENS-1) (Phosphoinositide-binding protein 1) (WD40- and FYVE domain-containing protein 1) (Zinc finger FYVE domain-containing protein 17)	46 kDa	0.039	7	6	1	2	1	3	0	0	1	0	0	-2.878174056	0% (0.084)
CRP_HUMAN	AK1BA_HUMAN	AKR1B10	O60218	Aldo-keto reductase family 1 member B10 (EC 1.1.1.-) (ARL-1) (Aldose reductase-like) (Aldose reductase-related protein) (ARP) (hARP) (Small intestine reductase) (SI reductase)	36 kDa	0.11	8	6	1	0	2	4	0	0	1	0	0	-2.878174056	0% (0.084)

COQ3_HUMAN	KAD1_HUMAN	AK1	P00568	Adenylate kinase isoenzyme 1 (AK 1) (EC 2.7.4.3) (EC 2.7.4.6) (ATP-AMP transphosphorylase 1) (ATP:AMP phosphotransferase) (Adenylate monophosphate kinase) (Myokinase)	22 kDa	0.089	7	6	1	0	1	1	4	0	1	0	0	-2.878174056	0% (0.084)
B4DKH6_HUMAN (+1)	ERLN2_HUMAN	ERLIN2	O94905	Erlin-2 (Endoplasmic reticulum lipid raft-associated protein 2) (Stomatin-prohibitin-flotillin-HflC/K domain-containing protein 2) (SPFH domain-containing protein 2)	38 kDa	0.072	79	61	18	19	13	14	15	1	5	4	8	-2.889821313	95% (< 0.00010)
RHOF_HUMAN	HSDL1_HUMAN	HSDL1	Q3SXM5	Inactive hydroxysteroid dehydrogenase-like protein 1	37 kDa	0.095	21	16	4	9	2	4	1	1	0	1	2	-2.935137323	95% (0.012)
MAVS_HUMAN	SCRB2_HUMAN	SCARB2	Q14108	Lysosome membrane protein 2 (85 kDa lysosomal membrane sialoglycoprotein) (LGP85) (CD36 antigen-like 2) (Lysosome membrane protein II) (LIMP II) (Scavenger receptor class B member 2) (CD antigen CD36)	54 kDa	0.048	33	26	7	9	7	6	4	0	1	4	3	-2.95085763	95% (0.0020)
QSOX2_HUMAN	H3BN55_HUMAN	RAB27A	H3BN55	Ras-related protein Rab-27A (Fragment)	21 kDa	0.039	16	13	3	10	0	2	1	0	0	2	1	-2.995118905	95% (0.019)
PRRC1_HUMAN	B4E2W6_HUMAN		B4E2W6	cDNA FLJ58409, highly similar to Tight junction protein ZO-3	97 kDa	0.065	16	13	3	6	2	0	5	0	3	0	0	-2.995118905	95% (0.019)
B4DGF8_HUMAN (+2)	CO6A1_HUMAN	COL6A1	P12109	Collagen alpha-1(VI) chain	109 kDa	0.043	16	13	3	1	7	5	0	0	0	0	3	-2.995118905	95% (0.019)
WDR12_HUMAN	EPIPL_HUMAN	EPPK1	P58107	Epiplakin (450 kDa epidermal antigen)	556 kDa	0.052	169	134	39	53	21	31	31	22	3	12	2	-3.004763848	95% (< 0.00010)
G9JXB8_HUMAN (+1)	GOGA3_HUMAN	GOLGA3	Q08378	Golgin subfamily A member 3 (Golgi complex-associated protein of 170 kDa) (GCP170) (Golgin-160)	167 kDa	0.026	3	3	0	0	2	1	0	0	0	0	0	-3.036896491	0% (0.15)
A8K5N3_HUMAN (+1)	SCEL_HUMAN	SCEL	O95171	Sciellin	78 kDa	0.042	3	3	0	1	0	0	2	0	0	0	0	-3.036896491	0% (0.15)
CHM2A_HUMAN	AT8B1_HUMAN	ATP8B1	O43520	Probable phospholipid-transporting ATPase IC (EC 3.6.3.1) (ATPase class I type 8B member 1) (Familial intrahepatic cholestasis type 1)	144 kDa	0.11	3	3	0	2	1	0	0	0	0	0	0	-3.036896491	0% (0.15)
A8MXZ4_HUMAN	TBD2A_HUMAN	TBC1D2	Q9BYX2	TBC1 domain family member 2A (Armus) (Prostate antigen recognized and identified by SEREX 1) (PARIS-1)	105 kDa	0.048	3	3	0	2	1	0	0	0	0	0	0	-3.036896491	0% (0.15)
TSR1_HUMAN	B4DEA3_HUMAN		B4DEA3	cDNA FLJ56531, highly similar to UV excision repair protein RAD23 homolog B	42 kDa	0.039	3	3	0	0	2	1	0	0	0	0	0	-3.036896491	0% (0.15)
GMPPB_HUMAN	Q53G41_HUMAN		Q53G41	Tripartite motif protein TRIM29 isoform alpha variant (Fragment)	66 kDa	0.12	3	3	0	2	0	1	0	0	0	0	0	-3.036896491	0% (0.15)
RM20_HUMAN	B2RBE6_HUMAN		B2RBE6	cDNA, FLJ95470, highly similar to Homo sapiens homolog of yeast Sec5 (SEC5), mRNA	104 kDa	0.14	3	3	0	1	1	1	0	0	0	0	0	-3.036896491	0% (0.15)
ANPRA_HUMAN	ACD10_HUMAN	ACAD10	Q6JQN1	Acyl-CoA dehydrogenase family member 10 (ACAD-10) (EC 1.3.99.-)	119 kDa	0.031	3	3	0	3	0	0	0	0	0	0	0	-3.036896491	0% (0.15)
B3KUH0_HUMAN (+1)	Q6QNY7_HUMAN	GNE	Q6QNY7	UDP-N-acetylglucosamine-2-epimerase / N-acetylmannosamine kinase	79 kDa	0.049	3	3	0	0	0	3	0	0	0	0	0	-3.036896491	0% (0.15)
PTX3_HUMAN	PKHG3_HUMAN	PLEKHG3	A1L390	Pleckstrin homology domain-containing family G member 3 (PH domain-containing family G member 3)	134 kDa	0.1	3	3	0	3	0	0	0	0	0	0	0	-3.036896491	0% (0.15)
VPS39_HUMAN	H0YBJ4_HUMAN	MATN2	H0YBJ4	Matrilin-2 (Fragment)	81 kDa	0.027	3	3	0	0	2	1	0	0	0	0	0	-3.036896491	0% (0.15)

PTPRJ_HUMAN	UB2J1_HUMAN	UBE2J1	Q9Y385	Ubiquitin-conjugating enzyme E2 J1 (EC 6.3.2.19) (Non-canonical ubiquitin-conjugating enzyme 1) (NCUBE-1) (Yeast ubiquitin-conjugating enzyme UBC6 homolog E) (HsUBC6e)	35 kDa	0.023	3	3	0	0	1	1	1	0	0	1	0	-3.036896491	0% (0.15)
LRP10_HUMAN (+2)	SYUG_HUMAN	SNCG	O76070	Gamma-synuclein (Breast cancer-specific gene 1 protein) (Persyn) (Synoretin) (SR)	13 kDa	0.056	3	3	0	0	0	0	3	0	0	0	0	-3.036896491	0% (0.15)
Q5HYI5_HUMAN (+1)	IGHM_HUMAN	IGHM	P01871	Ig mu chain C region	49 kDa	0.5	3	3	0	0	0	0	3	0	0	0	0	-3.036896491	0% (0.15)
C9J8U2_HUMAN (+3)	RS28_HUMAN	RPS28	P62857	40S ribosomal protein S28	8 kDa	0.051	3	3	0	0	1	2	0	0	0	0	0	-3.036896491	0% (0.15)
E9PJ81_HUMAN (+2)	B3KNF4_HUMAN		B3KNF4	cDNA FLJ14514 fis, clone NT2RM1000742, highly similar to Prominin-1	96 kDa	0.12	3	3	0	3	0	0	0	0	0	0	0	-3.036896491	0% (0.15)
CP1A1_HUMAN (+2)	RHOF_HUMAN	RHOF	Q9HBH0	Rho-related GTP-binding protein RhoF (Rho family GTPase Rif) (Rho in filopodia)	24 kDa	0.055	3	3	0	3	0	0	0	0	0	0	0	-3.036896491	0% (0.15)
B4DDH9_HUMAN (+1)	PTPRJ_HUMAN	PTPRJ	Q12913	Receptor-type tyrosine-protein phosphatase eta (Protein-tyrosine phosphatase eta) (R-PTP-eta) (EC 3.1.3.48) (Density-enhanced phosphatase 1) (DEP-1) (HPTP eta) (Protein-tyrosine phosphatase receptor type J) (R-PTP-J) (CD antigen CD148)	146 kDa	0.042	3	3	0	0	1	2	0	0	0	0	0	-3.036896491	0% (0.15)
NO66_HUMAN	LRP10_HUMAN	LRP10	Q7Z4F1	Low-density lipoprotein receptor-related protein 10 (LRP-10)	76 kDa	0.042	3	3	0	1	0	2	0	0	0	0	0	-3.036896491	0% (0.15)
TIGAR_HUMAN	B2RDH6_HUMAN		B2RDH6	cDNA, FLJ96613, highly similar to Homo sapiens vacuolar protein sorting 33A (yeast) (VPS33A), mRNA	68 kDa	0.13	3	3	0	2	0	0	1	0	0	0	0	-3.036896491	0% (0.15)
NHRF2_HUMAN (+1)	Q8N572_HUMAN	WBSCR16	Q8N572	WBSCR16 protein	49 kDa	0.077	3	3	0	3	0	0	0	0	0	0	0	-3.036896491	0% (0.15)
B4DL80_HUMAN (+2)	TATD1_HUMAN	TATDN1	Q6P1N9	Putative deoxyribonuclease TATDN1 (EC 3.1.21.-) (Hepatocarcinoma high expression protein)	34 kDa	0.039	3	3	0	0	1	0	3	0	0	0	0	-3.036896491	0% (0.15)
B2RDH6_HUMAN (+1)	B0AZM0_HUMAN		B0AZM0	cDNA, FLJ79460, highly similar to Voltage-gated potassium channel subunitbeta-2	39 kDa	0.037	3	3	0	0	0	1	2	0	0	0	0	-3.036896491	0% (0.15)
D3DSU3_HUMAN (+1)	Q6ZT62_HUMAN	SH3BP1	Q6ZT62	SH3 domain-binding protein 1 (cDNA FLJ44925 fis, clone BRAMY3014613, highly similar to Homo sapiens SH3-domain binding protein 1 (SH3BP1))	66 kDa	0.022	3	3	0	0	1	2	0	0	0	0	0	-3.036896491	0% (0.15)
B2RDI6_HUMAN (+2)	A2A2Q9_HUMAN	AAR2	A2A2Q9	Protein AAR2 homolog	45 kDa	0.028	3	3	0	0	0	2	1	0	0	0	0	-3.036896491	0% (0.15)
VAV2_HUMAN	DEOC_HUMAN	DERA	Q9Y315	Putative deoxyribose-phosphate aldolase (DERA) (EC 4.1.2.4) (2-deoxy-D-ribose 5-phosphate aldolase) (Phosphodeoxyriboaldolase) (Deoxyriboaldolase)	35 kDa	0.031	3	3	0	1	0	0	2	0	0	0	0	-3.036896491	0% (0.15)
B7Z1V3_HUMAN (+1)	FOLR1_HUMAN	FOLR1	P15328	Folate receptor alpha (FR-alpha) (Adult folate-binding protein) (FBP) (Folate receptor 1) (Folate receptor, adult) (KB cells FBP) (Ovarian tumor-associated antigen MOv18)	30 kDa	0.063	3	3	0	0	0	0	3	0	0	0	0	-3.036896491	0% (0.15)
Q8N572_HUMAN	CARM1_HUMAN	CARM1	Q86X55	Histone-arginine methyltransferase CARM1 (EC 2.1.1.-) (EC 2.1.1.125) (Coactivator-associated arginine methyltransferase 1) (Protein arginine N-methyltransferase 4)	66 kDa	0.059	3	3	0	1	1	0	1	0	0	0	0	-3.036896491	0% (0.15)

A6NEM5_HUMAN (+1)	GOGA7_HUMAN	GOLGA7	Q7Z5G4	Golgin subfamily A member 7 (Golgi complex-associated protein of 16 kDa)	16 kDa	0.069	3	3	0	1	0	1	1	0	0	0	0	-3.036896491	0% (0.15)
DHB8_HUMAN	PAPD1_HUMAN	MTPAP	Q9NVV4	Poly(A) RNA polymerase, mitochondrial (PAP) (EC 2.7.7.19) (PAP-associated domain-containing protein 1) (Polynucleotide adenylyltransferase) (Terminal uridylyltransferase 1) (TUTase 1) (mtPAP)	66 kDa	0.1	3	3	0	1	0	0	2	0	0	0	0	-3.036896491	0% (0.15)
B4DFL3_HUMAN (+1)	DDAH2_HUMAN	DDAH2	O95865	N(G),N(G)-dimethylarginine dimethylaminohydrolase 2 (DDAH-2) (Dimethylarginine dimethylaminohydrolase 2) (EC 3.5.3.18) (DDAHII) (Dimethylargininase-2) (Protein G6a) (S-phase protein)	30 kDa	0.14	3	3	0	0	0	0	3	0	0	0	0	-3.036896491	0% (0.15)
B2R960_HUMAN (+3)	B4E2A3_HUMAN		B4E2A3	cDNA FLJ52834, moderately similar to Cold-inducible RNA-binding protein	16 kDa	0.062	3	3	0	0	0	0	3	0	0	0	0	-3.036896491	0% (0.15)
C9JNZ0_HUMAN (+2)	B2RAU6_HUMAN		B2RAU6	cDNA, FLJ95129, highly similar to Homo sapiens phosphatidylinositol glycan, class Q (PIGQ), transcript variant 2, mRNA	65 kDa	0.073	3	3	0	3	0	0	0	0	0	0	0	-3.036896491	0% (0.15)
TIM13_HUMAN	C9JSC3_HUMAN	NCKIPSD	C9JSC3	NCK-interacting protein with SH3 domain (Fragment)	42 kDa	0.26	3	3	0	0	1	1	1	0	0	0	0	-3.036896491	0% (0.15)
VATF_HUMAN	CD59_HUMAN	CD59	P13987	CD59 glycoprotein (1F5 antigen) (20 kDa homologous restriction factor) (HRF-20) (HRF20) (MAC-inhibitory protein) (MAC-IP) (MEM43 antigen) (Membrane attack complex inhibition factor) (MACIF) (Membrane inhibitor of reactive lysis) (MIRL) (Protectin) (CD antigen CD59)	14 kDa	0.24	3	3	0	1	1	0	1	0	0	0	0	-3.036896491	0% (0.15)
HSP7E_HUMAN	DOPP1_HUMAN	DOLPP1	Q86YN1	Dolichyldiphosphatase 1 (EC 3.6.1.43) (Dolichyl pyrophosphate phosphatase 1)	27 kDa	0.049	3	3	0	1	0	0	2	0	0	0	0	-3.036896491	0% (0.15)
B3GN3_HUMAN (+2)	A6XNE0_HUMAN		A6XNE0	Glycodelin	16 kDa	0.07	3	3	0	3	0	0	0	0	0	0	0	-3.036896491	0% (0.15)
G8JLK1_HUMAN	B2R7K1_HUMAN		B2R7K1	cDNA, FLJ93479, highly similar to Homo sapiens solute carrier family 1 (neuronal/epithelial high affinity glutamate transporter, system Xag), member 1 (SLC1A1), nuclear gene encoding mitochondrial protein, mRNA	57 kDa	0.089	3	3	0	3	0	0	0	0	0	0	0	-3.036896491	0% (0.15)
R4GN49_HUMAN (+1)	PLCE_HUMAN	AGPAT5	Q9NUQ2	1-acyl-sn-glycerol-3-phosphate acyltransferase epsilon (EC 2.3.1.51) (1-acylglycerol-3-phosphate O-acyltransferase 5) (1-AGP acyltransferase 5) (1-AGPAT 5) (Lysophosphatidic acid acyltransferase epsilon) (LPAAT-epsilon)	42 kDa	0.27	3	3	0	3	0	0	0	0	0	0	0	-3.036896491	0% (0.15)
H0YEE1_HUMAN (+1)	B7ZKL3_HUMAN	EPS8L2	B7ZKL3	EPS8L2 protein (Epidermal growth factor receptor kinase substrate 8-like protein 2)	82 kDa	0.13	12	10	2	6	1	2	1	2	0	0	0	-3.092052087	95% (0.031)
A8K6D2_HUMAN (+1)	BCS1_HUMAN	BCS1L	Q9Y276	Mitochondrial chaperone BCS1 (h-BCS1) (BCS1-like protein)	48 kDa	0.22	12	10	2	3	3	2	2	0	2	0	0	-3.092052087	95% (0.031)
CRK_HUMAN	B4DKK2_HUMAN	SRI	B4DKK2	Sorcin (cDNA FLJ60640, highly similar to Sorcin)	20 kDa	0.2	12	10	2	3	3	1	3	0	2	0	0	-3.092052087	95% (0.031)
PMVK_HUMAN	KCRU_HUMAN	CKMT1A	P12532	Creatine kinase U-type, mitochondrial (EC 2.7.3.2) (Acidic-type mitochondrial creatine kinase) (Mia-CK) (Ubiquitous mitochondrial creatine kinase) (U-MtCK)	47 kDa	0.11	39	31	8	13	5	3	11	2	0	6	0	-3.114908873	95% (0.00055)

SIAT1_HUMAN	DHRS3_HUMAN	DHRS3	O75911	Short-chain dehydrogenase/reductase 3 (EC 1.1.1.300) (DD83.1) (Retinal short-chain dehydrogenase/reductase 1) (retSDR1)	34 kDa	0.071	26	21	5	12	2	7	0	0	1	3	1	-3.180316077	95% (0.0031)
FA98B_HUMAN	H3BPE1_HUMAN	MACF1	H3BPE1	Microtubule-actin cross-linking factor 1, isoforms 1/2/3/5	857 kDa	0.13	54	43	11	9	11	19	5	4	0	7	0	-3.227622919	95% (< 0.00010)
I3L3B4_HUMAN	COTL1_HUMAN	COTL1	Q14019	Coactosin-like protein	16 kDa	0.23	7	7	1	0	2	5	0	0	0	1	0	-3.275205664	95% (0.050)
PTBP3_HUMAN	FA83H_HUMAN	FAM83H	Q6ZRV2	Protein FAM83H	127 kDa	0.063	8	7	1	5	0	0	2	1	0	0	0	-3.275205664	95% (0.050)
TATD1_HUMAN	VPS16_HUMAN	VPS16	Q9H269	Vacuolar protein sorting-associated protein 16 homolog (hVPS16)	95 kDa	0.13	8	7	1	2	2	1	2	0	1	0	0	-3.275205664	95% (0.050)
F5H2X3_HUMAN	A8K5C2_HUMAN		A8K5C2	cDNA FLJ75055	51 kDa	0.034	8	7	1	3	4	0	0	1	0	0	0	-3.275205664	95% (0.050)
HINT3_HUMAN	B1AUU8_HUMAN	EPS15	B1AUU8	Epidermal growth factor receptor substrate 15	84 kDa	0.14	8	7	1	1	4	2	0	1	0	0	0	-3.275205664	95% (0.050)
A8K7A1_HUMAN (+1)	COX15_HUMAN	COX15	Q7KZN9	Cytochrome c oxidase assembly protein COX15 homolog	46 kDa	0.043	8	7	1	3	0	0	4	0	1	0	0	-3.275205664	95% (0.050)
B2ZGL7_HUMAN (+4)	G3V2F7_HUMAN	TMEM189	G3V2F7	HCG2044781 (Protein TMEM189-UBE2V1) (Transmembrane protein 189)	42 kDa	0.13	8	7	1	1	1	3	2	1	0	0	0	-3.275205664	95% (0.050)
EXOS4_HUMAN	B3KSG7_HUMAN		B3KSG7	cDNA FLJ36171 fis, clone TESTI2026215, highly similar to Homo sapiens two pore segment channel 1 (TPCN1), mRNA	94 kDa	0.11	8	7	1	4	2	1	0	0	0	0	1	-3.275205664	95% (0.050)
B0AZM0_HUMAN (+6)	A8K9V9_HUMAN		A8K9V9	cDNA FLJ76064	35 kDa	0.084	8	7	1	4	0	2	1	0	0	1	0	-3.275205664	95% (0.050)
A2VDI4_HUMAN (+2)	ITB8_HUMAN	ITGB8	P26012	Integrin beta-8	86 kDa	0.043	8	7	1	3	1	1	2	0	0	1	0	-3.275205664	95% (0.050)
TIG1_HUMAN	D3DQ48_HUMAN	TMEM59	D3DQ48	Transmembrane protein 59, isoform CRA_e	36 kDa	0.071	8	7	1	0	4	3	0	1	0	0	0	-3.275205664	95% (0.050)
NCKPL_HUMAN	CX6B1_HUMAN	COX6B1	P14854	Cytochrome c oxidase subunit 6B1 (Cytochrome c oxidase subunit VIb isoform 1) (COX VIb-1)	10 kDa	0.023	8	7	1	1	1	2	3	1	0	0	0	-3.275205664	95% (0.050)
BRCC3_HUMAN (+2)	B4DWA0_HUMAN		B4DWA0	cDNA FLJ54188, moderately similar to High mobility group protein HMG-I/HMG-Y	34 kDa	0.076	13	11	2	2	3	1	5	2	0	0	0	-3.366944425	95% (0.019)
SPEE_HUMAN	CD109_HUMAN	CD109	Q6YHK3	CD109 antigen (150 kDa TGF-beta-1-binding protein) (C3 and PZP-like alpha-2-macroglobulin domain-containing protein 7) (Platelet-specific Gov antigen) (p180) (r150) (CD antigen CD109)	162 kDa	0.076	23	19	4	6	7	6	0	2	0	2	0	-3.445728826	95% (0.0031)
TPPC5_HUMAN	ANXA9_HUMAN	ANXA9	O76027	Annexin A9 (Annexin XXXI) (Annexin-31) (Annexin-9) (Pemphaxin)	38 kDa	0.11	29	23	5	13	0	1	10	5	0	0	0	-3.466276278	95% (0.0013)
B7Z377_HUMAN (+2)	S12A2_HUMAN	SLC12A2	P55011	Solute carrier family 12 member 2 (Basolateral Na-K-Cl symporter) (Bumetanide-sensitive sodium-(potassium)-chloride cotransporter 1)	131 kDa	0.19	38	31	7	1	14	17	0	5	0	1	1	-3.492523828	95% (0.00023)
MRT4_HUMAN	A4D2Q0_HUMAN	UNC84A	A4D2Q0	SUN domain-containing protein 1 (Unc-84 homolog A (C. elegans))	108 kDa	0.088	19	16	3	13	2	0	2	1	0	0	2	-3.625810028	95% (0.0047)
DDX60_HUMAN	APEX1_HUMAN	APEX1	P27695	DNA-(apurinic or apyrimidinic site) lyase (EC 3.1.-.-) (EC 4.2.99.18) (APEX nuclease) (APEN) (Apurinic-apyrimidinic endonuclease 1) (AP endonuclease 1) (APE-1) (REF-1) (Redox factor-1) [Cleaved into: DNA-(apurinic or apyrimidinic site) lyase, mitochondrial]	36 kDa	0.016	19	16	3	0	1	1	14	1	1	1	0	-3.625810028	95% (0.0047)

GTDC2_HUMAN	GDPD3_HUMAN	GDPD3	Q7L5L3	Glycerophosphodiester phosphodiesterase domain-containing protein 3 (EC 3.1.-.-)	37 kDa	0.074	14	12	2	1	1	0	10	1	0	1	0	-3.641843827	95% (0.012)
AL3B1_HUMAN (+1)	F8J390_HUMAN	PKP3	F8J390	Plakophilin 3b (Fragment)	89 kDa	0.11	9	8	1	4	0	2	2	1	0	0	0	-3.672247474	95% (0.029)
B7Z3I9_HUMAN (+2)	H0Y6T6_HUMAN	NRM	H0Y6T6	Nurim (Fragment)	30 kDa	0.096	9	8	1	2	0	0	6	0	0	0	1	-3.672247474	95% (0.029)
A16A1_HUMAN	DNJC5_HUMAN	DNAJC5	Q9H3Z4	DnaJ homolog subfamily C member 5 (Cysteine string protein) (CSP)	22 kDa	0.031	9	8	1	2	2	2	2	0	0	0	1	-3.672247474	95% (0.029)
A8K367_HUMAN (+3)	SPS1_HUMAN	SEPHS1	P49903	Selenide, water dikinase 1 (EC 2.7.9.3) (Selenium donor protein 1) (Selenophosphate synthase 1)	43 kDa	0.035	9	8	1	0	3	1	4	1	0	0	0	-3.672247474	95% (0.029)
Q7Z524_HUMAN	SRRM2_HUMAN	SRRM2	Q9UQ35	Serine/arginine repetitive matrix protein 2 (300 kDa nuclear matrix antigen) (Serine/arginine-rich splicing factor-related nuclear matrix protein of 300 kDa) (SR-related nuclear matrix protein of 300 kDa) (Ser/Arg-related nuclear matrix protein of 300 kDa) (Splicing coactivator subunit SRm300) (Tax-responsive enhancer element-binding protein 803) (TaxREB803)	300 kDa	0.053	4	4	0	0	2	2	0	0	0	0	0	-3.751508572	0% (0.078)
B2RBP3_HUMAN (+2)	B7ZKT7_HUMAN	FYCO1	B7ZKT7	FYVE and coiled-coil domain-containing protein 1 (Uncharacterized protein)	169 kDa	0.065	4	4	0	4	0	0	0	0	0	0	0	-3.751508572	0% (0.078)
GPAT4_HUMAN	LARP1_HUMAN	LARP1	Q6PKG0	La-related protein 1 (La ribonucleoprotein domain family member 1)	124 kDa	0.059	4	4	0	0	2	2	0	0	0	0	0	-3.751508572	0% (0.078)
Q6ZT62_HUMAN	SH3L1_HUMAN	SH3BGRL	O75368	SH3 domain-binding glutamic acid-rich-like protein	13 kDa	0.04	4	4	0	0	1	1	2	0	0	0	0	-3.751508572	0% (0.078)
G3V3Y1_HUMAN (+1)	AUHM_HUMAN	AUH	Q13825	Methylglutaconyl-CoA hydratase, mitochondrial (EC 4.2.1.18) (AU-specific RNA-binding enoyl-CoA hydratase) (AU-binding protein/enoyl-CoA hydratase)	36 kDa	0.091	4	4	0	4	0	0	0	0	0	0	0	-3.751508572	0% (0.078)
A8K6N3_HUMAN (+1)	Q53F93_HUMAN		Q53F93	Palmitoylated membrane protein 1 variant (Fragment)	52 kDa	0.043	4	4	0	0	2	2	0	0	0	0	0	-3.751508572	0% (0.078)
B7SBB1_HUMAN	SBDS_HUMAN	SBDS	Q9Y3A5	Ribosome maturation protein SBDS (Shwachman-Bodian-Diamond syndrome protein)	29 kDa	0.079	4	4	0	0	0	0	4	0	0	0	0	-3.751508572	0% (0.078)
GSH0_HUMAN	AT11A_HUMAN	ATP11A	P98196	Probable phospholipid-transporting ATPase IH (EC 3.6.3.1) (ATPase IS) (ATPase class VI type 11A)	130 kDa	0.21	4	4	0	1	2	1	0	0	0	0	0	-3.751508572	0% (0.078)
UBA7_HUMAN	B3KQQ0_HUMAN		B3KQQ0	cDNA PSEC0007 fis, clone NT2RM1000634, highly similar to FK506-binding protein 9 (EC 5.2.1.8)	63 kDa	0.031	4	4	0	0	0	3	1	0	0	0	0	-3.751508572	0% (0.078)
A8K3E4_HUMAN (+1)	CATG_HUMAN	CTSG	P08311	Cathepsin G (CG) (EC 3.4.21.20)	29 kDa	0.042	4	4	0	2	0	0	2	0	0	0	0	-3.751508572	0% (0.078)
M0QXN5_HUMAN (+1)	OSBL3_HUMAN	OSBPL3	Q9H4L5	Oxysterol-binding protein-related protein 3 (ORP-3) (OSBP-related protein 3)	101 kDa	0.063	4	4	0	3	0	1	0	0	0	0	0	-3.751508572	0% (0.078)
E7ETU7_HUMAN (+2)	IF44L_HUMAN	IFI44L	Q53G44	Interferon-induced protein 44-like	51 kDa	0.048	4	4	0	0	0	0	4	0	0	0	0	-3.751508572	0% (0.078)
B3KTL0_HUMAN (+1)	CI142_HUMAN	C9orf142	Q9BUH6	Uncharacterized protein C9orf142	22 kDa	0.062	4	4	0	0	0	0	4	0	0	0	0	-3.751508572	0% (0.078)
A2IDC6_HUMAN (+2)	B4DRD3_HUMAN		B4DRD3	cDNA FLJ58123, highly similar to Tissue-type plasminogen activator (EC 3.4.21.68)	58 kDa	0.087	4	4	0	4	0	0	0	0	0	0	0	-3.751508572	0% (0.078)
S35E1_HUMAN	TM260_HUMAN	TMEM260	Q9NX78	Transmembrane protein 260	80 kDa	0.088	4	4	0	4	0	0	0	0	0	0	0	-3.751508572	0% (0.078)

A2A2Q9_HUMAN (+1)	B2R745_HUMAN		B2R745	cDNA, FLJ93277, highly similar to Homo sapiens solute carrier family 30 (zinc transporter), member 9(SLC30A9), mRNA	63 kDa	0.078	4	4	0	4	0	0	0	0	0	0	0	-3.751508572	0% (0.078)
DEOC_HUMAN (+1)	H3BRY5_HUMAN	SULT1A1	H3BRY5	Sulfotransferase 1A1 (Fragment)	33 kDa	0.079	4	4	0	0	0	0	4	0	0	0	0	-3.751508572	0% (0.078)
A8K9V7_HUMAN (+12)	MRP3_HUMAN	ABCC3	O15438	Canalicular multispecific organic anion transporter 2 (ATP-binding cassette sub-family C member 3) (Multi-specific organic anion transporter D) (MOAT-D) (Multidrug resistance-associated protein 3)	169 kDa	0.037	4	4	0	0	3	1	0	0	0	0	0	-3.751508572	0% (0.078)
B3KY51_HUMAN (+1)	B4DKH6_HUMAN		B4DKH6	cDNA FLJ51234, highly similar to Bactericidal permeability-increasing protein	45 kDa	0.046	4	4	0	4	0	0	0	0	0	0	0	-3.751508572	0% (0.078)
FOLR1_HUMAN	HINT3_HUMAN	HINT3	Q9NQE9	Histidine triad nucleotide-binding protein 3 (HINT-3) (EC 3.-.-.-)	20 kDa	0.074	3	4	0	3	0	0	1	0	0	0	0	-3.751508572	0% (0.078)
H7C189_HUMAN (+3)	A16A1_HUMAN	ALDH16A1	Q8IZ83	Aldehyde dehydrogenase family 16 member A1	85 kDa	0.064	4	4	0	0	1	1	2	0	0	0	0	-3.751508572	0% (0.078)
H6A2E0_HUMAN	RRFM_HUMAN	MRRF	Q96E11	Ribosome-recycling factor, mitochondrial (RRF) (Ribosome-releasing factor, mitochondrial)	29 kDa	0.4	4	4	0	1	1	0	2	0	0	0	0	-3.751508572	0% (0.078)
AL1A1_HUMAN (+1)	MPZL2_HUMAN	MPZL2	O60487	Myelin protein zero-like protein 2 (Epithelial V-like antigen 1)	24 kDa	0.096	4	4	0	2	1	1	0	0	0	0	0	-3.751508572	0% (0.078)
ISCA2_HUMAN	UPP1_HUMAN	UPP1	Q16831	Uridine phosphorylase 1 (UPase 1) (UrdPase 1) (EC 2.4.2.3)	34 kDa	0.16	4	4	0	0	3	1	0	0	0	0	0	-3.751508572	0% (0.078)
L7QJN0_HUMAN	TALDO_HUMAN	TALDO1	P37837	Transaldolase (EC 2.2.1.2)	38 kDa	0.29	25	21	4	2	2	9	8	1	1	1	1	-3.786145033	95% (0.0012)
CARM1_HUMAN	A8K9B9_HUMAN		A8K9B9	cDNA FLJ77391, highly similar to Homo sapiens EH-domain containing 4 (EHD4), mRNA	61 kDa	0.038	20	17	3	4	5	0	8	0	3	0	0	-3.836051208	95% (0.0029)
J3QT22_HUMAN (+1)	Q59FB9_HUMAN		Q59FB9	Toll interacting protein variant (Fragment)	39 kDa	0.05	15	13	2	6	4	2	2	0	0	1	1	-3.916750294	95% (0.0070)
B5BUI8_HUMAN (+1)	Q5HYL6_HUMAN	DKFZp686E1899	Q5HYL6	Putative uncharacterized protein DKFZp686E1899	40 kDa	0.22	15	13	2	1	1	7	4	0	0	2	0	-3.916750294	95% (0.0070)
B4DDV1_HUMAN (+1)	B2R6P3_HUMAN		B2R6P3	cDNA, FLJ93047, highly similar to Homo sapiens matrix metallopeptidase 14 (membrane-inserted) (MMP14), mRNA	66 kDa	0.06	14	13	2	3	5	5	0	0	0	0	2	-3.916750294	95% (0.0070)
A8K3S0_HUMAN (+5)	A8K6K8_HUMAN		A8K6K8	cDNA FLJ77325	55 kDa	0.11	36	31	6	14	5	3	9	0	1	2	2	-3.974308426	95% (< 0.00010)
TRMB_HUMAN	E5KSE7_HUMAN		E5KSE7	Mitochondrial short-chain specific acyl-CoA dehydrogenase	44 kDa	0.094	21	18	3	5	5	3	5	0	1	0	1	-4.046297792	95% (0.0018)
ARF5_HUMAN (+1)	F8VXY3_HUMAN	OAS1	F8VXY3	2'-5'-oligoadenylate synthase 1	41 kDa	0.28	21	18	3	0	0	0	18	0	3	0	0	-4.046297792	95% (0.0018)
RRFM_HUMAN	ABCD1_HUMAN	ABCD1	P33897	ATP-binding cassette sub-family D member 1 (Adrenoleukodystrophy protein) (ALDP)	83 kDa	0.1	10	9	1	4	1	0	4	0	0	1	0	-4.069299488	95% (0.017)
GMPPA_HUMAN	NDUS5_HUMAN	NDUFS5	O43920	NADH dehydrogenase [ubiquinone] iron-sulfur protein 5 (Complex I-15 kDa) (CI-15 kDa) (NADH-ubiquinone oxidoreductase 15 kDa subunit)	13 kDa	0.064	10	9	1	3	2	2	2	0	0	1	0	-4.069299488	95% (0.017)
MPZL2_HUMAN	DHSO_HUMAN	SORD	Q00796	Sorbitol dehydrogenase (EC 1.1.1.14) (L-iditol 2-dehydrogenase)	38 kDa	0.1	10	9	1	0	0	0	9	0	0	1	0	-4.069299488	95% (0.017)
GOGA7_HUMAN	ECHD1_HUMAN	ECHDC1	Q9NTX5	Ethylmalonyl-CoA decarboxylase (EC 4.1.1.94) (Enoyl-CoA hydratase	34 kDa	0.18	10	9	1	3	2	1	3	0	0	1	0	-4.069299488	95% (0.017)

				domain-containing protein 1) (Methylmalonyl-CoA decarboxylase) (MMCD) (EC 4.1.1.41)														
PAPD1_HUMAN	PLEC_HUMAN	PLEC	Q15149	Plectin (PCN) (PLTN) (Hemidesmosomal protein 1) (HD1) (Plectin-1)	532 kDa	0.043	1357	1115	246	418	276	278	146	34	22	108	83	-4.076490048	95% (< 0.00010)
HEAT3_HUMAN	ANK1_HUMAN	ANK1	P16157	Ankyrin-1 (ANK-1) (Ankyrin-R) (Erythrocyte ankyrin)	206 kDa	0.035	33	28	5	0	12	16	0	0	0	5	0	-4.181241096	95% (0.00012)
NENF_HUMAN	CIB1_HUMAN	CIB1	Q99828	Calcium and integrin-binding protein 1 (CIB) (Calcium- and integrin-binding protein) (CIBP) (Calmyrin) (DNA-PKcs-interacting protein) (Kinase-interacting protein) (KIP) (SNK-interacting protein 2-28) (SIP2-28)	22 kDa	0.15	16	14	2	4	2	0	8	0	0	0	2	-4.191663827	95% (0.0042)
PAR6B_HUMAN	PPB1_HUMAN	ALPP	P05187	Alkaline phosphatase, placental type (EC 3.1.3.1) (Alkaline phosphatase Regan isozyme) (Placental alkaline phosphatase 1) (PLAP-1)	58 kDa	0.081	28	24	4	14	5	5	0	0	0	0	4	-4.296802157	95% (0.00028)
INT5_HUMAN	DOCK7_HUMAN	DOCK7	Q96N67	Dedicator of cytokinesis protein 7	243 kDa	0.03	5	5	0	1	0	3	1	0	0	0	0	-4.466139016	95% (0.042)
NCALD_HUMAN	PERE_HUMAN	EPX	P11678	Eosinophil peroxidase (EPO) (EC 1.11.1.7) [Cleaved into: Eosinophil peroxidase light chain; Eosinophil peroxidase heavy chain]	81 kDa	0.17	5	5	0	2	0	0	3	0	0	0	0	-4.466139016	95% (0.042)
E7EQU1_HUMAN (+3)	TB182_HUMAN	TNKS1BP1	Q9C0C2	182 kDa tankyrase-1-binding protein	182 kDa	0.15	5	5	0	2	1	2	0	0	0	0	0	-4.466139016	95% (0.042)
UPP1_HUMAN	SLMAP_HUMAN	SLMAP	Q14BN4	Sarcolemmal membrane-associated protein (Sarcolemmal-associated protein)	95 kDa	0.068	5	5	0	5	0	0	0	0	0	0	0	-4.466139016	95% (0.042)
B1AZU4_HUMAN (+1)	ITCH_HUMAN	ITCH	Q96J02	E3 ubiquitin-protein ligase Itchy homolog (Itch) (EC 6.3.2.-) (Atrophin-1-interacting protein 4) (AIP4) (NFE2-associated polypeptide 1) (NAPP1)	103 kDa	0.11	5	5	0	1	1	3	0	0	0	0	0	-4.466139016	95% (0.042)
HMCS1_HUMAN (+2)	RTN2_HUMAN	RTN2	O75298	Reticulon-2 (Neuroendocrine-specific protein-like 1) (NSP-like protein 1) (Neuroendocrine-specific protein-like I) (NSP-like protein I) (NSPLI)	59 kDa	0.046	5	5	0	3	0	0	2	0	0	0	0	-4.466139016	95% (0.042)
DDAH2_HUMAN	B7Z7E4_HUMAN		B7Z7E4	cDNA FLJ53675, highly similar to Homo sapiens LETM1 domain containing 1 (LETMD1), transcript variant 1, mRNA	31 kDa	0.18	4	5	0	2	0	0	3	0	0	0	0	-4.466139016	95% (0.042)
A8K0C9_HUMAN (+1)	Q53GD8_HUMAN		Q53GD8	Peptidyl-prolyl cis-trans isomerase (Fragment)	25 kDa	0.17	5	5	0	0	1	0	4	0	0	0	0	-4.466139016	95% (0.042)
PHS_HUMAN (+1)	Q9Y3E8_HUMAN	C17orf25	Q9Y3E8	CGI-150 protein	55 kDa	0.25	5	5	0	0	2	1	2	0	0	0	0	-4.466139016	95% (0.042)
B4E2A3_HUMAN	B7Z4C3_HUMAN		B7Z4C3	cDNA FLJ50805, highly similar to Erythrocyte membrane protein band 4.2	77 kDa	0.18	5	5	0	0	4	1	0	0	0	0	0	-4.466139016	95% (0.042)
B4DMK2_HUMAN	B7Z7Y3_HUMAN		B7Z7Y3	cDNA FLJ61618, highly similar to Discoidin, CUB and LCCL domain-containing protein 2	74 kDa	0.071	5	5	0	5	0	0	0	0	0	0	0	-4.466139016	95% (0.042)
B7Z2F7_HUMAN (+1)	B2R880_HUMAN		B2R880	cDNA, FLJ93778, highly similar to Homo sapiens CD38 antigen (p45) (CD38), mRNA	34 kDa	0.039	5	5	0	0	0	0	5	0	0	0	0	-4.466139016	95% (0.042)
ETUD1_HUMAN	B2RAY1_HUMAN		B2RAY1	cDNA, FLJ95184, highly similar to Homo sapiens signal transducing adaptor molecule (SH3 domain and ITAM motif) 1 (STAM), mRNA	59 kDa	0.026	5	5	0	1	1	3	0	0	0	0	0	-4.466139016	95% (0.042)
B2RAU6_HUMAN	OSB11_HUMAN	OSBPL11	Q9BXB4	Oxysterol-binding protein-related protein 11 (ORP-11) (OSBP-related protein 11)	84 kDa	0.048	5	5	0	1	1	2	1	0	0	0	0	-4.466139016	95% (0.042)

CASP6_HUMAN	C9K0W8_HUMAN	STX16	C9K0W8	Syntaxin-16 (Uncharacterized protein)	35 kDa	0.13	5	5	0	3	1	1	0	0	0	0	0	-4.466139016	95% (0.042)
Q56FN6_HUMAN	SPRY7_HUMAN	SPRYD7	Q5W111	SPRY domain-containing protein 7 (Chronic lymphocytic leukemia deletion region gene 6 protein) (CLL deletion region gene 6 protein)	22 kDa	0.24	5	5	0	4	1	0	0	0	0	0	0	-4.466139016	95% (0.042)
CTBP2_HUMAN (+2)	PLPL4_HUMAN	PNPLA4	P41247	Patatin-like phospholipase domain-containing protein 4 (EC 3.1.1.3) (Protein GS2)	28 kDa	0.029	5	5	0	4	0	1	0	0	0	0	0	-4.466139016	95% (0.042)
CBL_HUMAN	VATF_HUMAN	ATP6V1F	Q16864	V-type proton ATPase subunit F (V-ATPase subunit F) (V-ATPase 14 kDa subunit) (Vacuolar proton pump subunit F)	13 kDa	0.029	5	5	0	1	1	1	2	0	0	0	0	-4.466139016	95% (0.042)
A8K097_HUMAN (+1)	B2ZGL7_HUMAN	MGLL	B2ZGL7	Monoacylglycerol lipase isoform 2	30 kDa	0.058	5	5	0	4	1	0	0	0	0	0	0	-4.466139016	95% (0.042)
B7Z6C9_HUMAN (+1)	EXOS4_HUMAN	EXOSC4	Q9NPD3	Exosome complex component RRP41 (Exosome component 4) (Ribosomal RNA-processing protein 41) (p12A)	26 kDa	0.036	5	5	0	0	3	0	2	0	0	0	0	-4.466139016	95% (0.042)
MGME1_HUMAN	RB27B_HUMAN	RAB27B	O00194	Ras-related protein Rab-27B (C25KG)	25 kDa	0.087	11	10	1	7	1	2	0	0	0	1	0	-4.466361706	95% (0.0098)
XCT_HUMAN	PERM_HUMAN	MPO	P05164	Myeloperoxidase (MPO) (EC 1.11.2.2) [Cleaved into: Myeloperoxidase; 89 kDa myeloperoxidase; 84 kDa myeloperoxidase; Myeloperoxidase light chain; Myeloperoxidase heavy chain]	84 kDa	0.058	29	25	4	12	0	0	13	0	1	3	0	-4.467029948	95% (0.00017)
ATLA1_HUMAN (+2)	B2RMN7_HUMAN	SPTB	B2RMN7	Spectrin, beta, erythrocytic	247 kDa	0.041	18	16	2	0	6	10	0	1	0	0	0	-4.741512088	95% (0.0015)
PPA6_HUMAN	CACP_HUMAN	CRAT	P43155	Carnitine O-acetyltransferase (Carnitine acetylase) (EC 2.3.1.7) (Carnitine acetyltransferase) (CAT) (CrAT)	71 kDa	0.054	12	11	1	1	1	5	4	0	1	0	0	-4.863434128	95% (0.0056)
A8K2S1_HUMAN	ES8L1_HUMAN	EPS8L1	Q8TE68	Epidermal growth factor receptor kinase substrate 8-like protein 1 (EPS8-like protein 1) (Epidermal growth factor receptor pathway substrate 8-related protein 1) (EPS8-related protein 1)	80 kDa	0.036	12	11	1	11	0	0	1	0	0	0	2	-4.863434128	95% (0.0056)
MP2K3_HUMAN (+2)	TRFM_HUMAN	MFI2	P08582	Melanotransferrin (Melanoma-associated antigen p97) (CD antigen CD228)	80 kDa	0.092	12	11	1	6	3	2	0	0	0	1	0	-4.863434128	95% (0.0056)
ARMC9_HUMAN (+2)	A8K8G0_HUMAN	HDGF	A8K8G0	Hepatoma-derived growth factor (cDNA FLJ75113)	23 kDa	0.024	33	29	4	4	6	8	11	0	2	0	2	-5.147984872	95% (< 0.00010)
C9JSC3_HUMAN (+1)	CTBP1_HUMAN	CTBP1	Q13363	C-terminal-binding protein 1 (CtBP1) (EC 1.1.1.-)	48 kDa	0.063	6	6	0	0	1	3	2	0	0	0	0	-5.180787824	95% (0.022)
EIF1_HUMAN (+1)	A2BDK6_HUMAN	MAP1B	A2BDK6	Microtubule-associated protein 1B (Microtubule-associated protein 1B, isoform CRA_a)	257 kDa	0.29	6	6	0	2	1	3	0	0	0	0	0	-5.180787824	95% (0.022)
B4DMD3_HUMAN (+3)	B7ZKT9_HUMAN	KIAA1033	B7ZKT9	KIAA1033 protein	137 kDa	0.036	5	6	0	5	0	0	1	0	0	0	0	-5.180787824	95% (0.022)
SLAF8_HUMAN	E5KNQ5_HUMAN	TK2	E5KNQ5	Mitochondrial thymidine kinase 2 (Thymidine kinase 2, mitochondrial) (Thymidine kinase 2, mitochondrial, isoform CRA_e)	35 kDa	0.11	6	6	0	2	2	1	1	0	0	0	0	-5.180787824	95% (0.022)
GATC_HUMAN (+1)	E7ET76_HUMAN	GGT1	E7ET76	Gamma-glutamyltranspeptidase 1 light chain (Fragment)	50 kDa	0.19	6	6	0	3	1	2	0	0	0	0	0	-5.180787824	95% (0.022)
AAK1_HUMAN (+4)	GP110_HUMAN	GPR110	Q5T601	Probable G-protein coupled receptor 110 (G-protein coupled receptor KPG_012) (G-protein coupled receptor PGR19)	101 kDa	0.028	6	6	0	4	0	0	2	0	0	0	0	-5.180787824	95% (0.022)
E9PGT3_HUMAN (+2)	C9JS50_HUMAN	STEAP4	C9JS50	Metalloreductase STEAP4 (Uncharacterized protein)	39 kDa	0.062	6	6	0	0	2	1	3	0	0	0	0	-5.180787824	95% (0.022)

HID1_HUMAN	LAMC2_HUMAN	LAMC2	Q13753	Laminin subunit gamma-2 (Cell-scattering factor 140 kDa subunit) (CSF 140 kDa subunit) (Epiligrin subunit gamma) (Kalinin subunit gamma) (Kalinin/nicein/epiligrin 100 kDa subunit) (Ladsin 140 kDa subunit) (Laminin B2t chain) (Laminin-5 subunit gamma) (Large adhesive scatter factor 140 kDa subunit) (Nicein subunit gamma)	131 kDa	0.049	34	30	4	4	12	14	0	0	0	0	3	-5.318234544	95% (< 0.00010)
SSH3_HUMAN	IFIT2_HUMAN	IFIT2	P09913	Interferon-induced protein with tetratricopeptide repeats 2 (IFIT-2) (ISG-54 K) (Interferon-induced 54 kDa protein) (IFI-54K) (P54)	55 kDa	0.036	14	13	1	0	0	0	13	0	1	0	0	-5.657609586	95% (0.0018)
F127A_HUMAN	HMGB2_HUMAN	HMGB2	P26583	High mobility group protein B2 (High mobility group protein 2) (HMG-2)	24 kDa	0.23	14	13	1	0	1	3	7	1	0	0	0	-5.657609586	95% (0.0018)
VAMP8_HUMAN	B3KUB6_HUMAN		B3KUB6	cDNA FLJ39529 fis, clone PUAEN2004067, highly similar to Band 4.1-like protein 1	86 kDa	0.24	22	20	2	1	5	8	6	0	1	0	1	-5.841293406	95% (0.00017)
CAMP_HUMAN (+1)	DHRS1_HUMAN	DHRS1	Q96LJ7	Dehydrogenase/reductase SDR family member 1 (EC 1.1.-.-)	34 kDa	0.059	7	7	0	3	0	0	4	0	0	0	0	-5.895454998	95% (0.012)
GGCT_HUMAN	MYO5C_HUMAN	MYO5C	Q9NQX4	Unconventional myosin-Vc	203 kDa	0.11	7	7	0	7	0	0	0	0	0	0	0	-5.895454998	95% (0.012)
RB22A_HUMAN	IPRI_HUMAN	ITPRIP	Q8IWB1	Inositol 1,4,5-trisphosphate receptor-interacting protein (Protein DANGER)	62 kDa	0.26	7	7	0	6	0	0	1	0	0	0	0	-5.895454998	95% (0.012)
F8VWL0_HUMAN (+1)	ATP9A_HUMAN	ATP9A	O75110	Probable phospholipid-transporting ATPase IIA (EC 3.6.3.1) (ATPase class II type 9A)	119 kDa	0.073	7	7	0	7	0	0	0	0	0	0	0	-5.895454998	95% (0.012)
NDUB1_HUMAN	E9PDY6_HUMAN	MUC4	E9PDY6	Mucin-4 beta chain	542 kDa	0.16	7	7	0	6	1	0	0	0	0	0	0	-5.895454998	95% (0.012)
ARFRP_HUMAN (+2)	B4DYN8_HUMAN		B4DYN8	cDNA FLJ56519, highly similar to Interferon-induced protein 44	47 kDa	0.14	7	7	0	0	0	0	7	0	0	0	0	-5.895454998	95% (0.012)
C9J712_HUMAN (+1)	RRAGA_HUMAN	RRAGA	Q7L523	Ras-related GTP-binding protein A (Rag A) (RagA) (Adenovirus E3 14.7 kDa-interacting protein 1) (FIP-1)	37 kDa	0.23	7	7	0	1	1	3	2	0	0	0	0	-5.895454998	95% (0.012)
RPR1A_HUMAN	K7EKI8_HUMAN	PPL	K7EKI8	Periplakin	204 kDa	0.14	211	187	25	98	25	18	42	11	0	7	7	-6.418424993	95% (< 0.00010)
CD59_HUMAN (+2)	E7ET40_HUMAN	PLAU	E7ET40	Urokinase-type plasminogen activator chain B	47 kDa	0.19	8	8	0	8	0	0	0	0	0	0	0	-6.610140537	95% (0.0062)
DCAKD_HUMAN (+1)	DPP2_HUMAN	DPP7	Q9UHL4	Dipeptidyl peptidase 2 (EC 3.4.14.2) (Dipeptidyl aminopeptidase II) (Dipeptidyl peptidase 7) (Dipeptidyl peptidase II) (DPP II) (Quiescent cell proline dipeptidase)	54 kDa	0.11	8	8	0	3	3	0	2	0	0	0	0	-6.610140537	95% (0.0062)
D6REB4_HUMAN (+1)	DECR2_HUMAN	DECR2	Q9NUI1	Peroxisomal 2,4-dienoyl-CoA reductase (pDCR) (EC 1.3.1.34) (2,4-dienoyl-CoA reductase 2)	31 kDa	0.057	8	8	0	4	0	0	4	0	0	0	0	-6.610140537	95% (0.0062)
G3ST2_HUMAN	TM192_HUMAN	TMEM192	Q8IY95	Transmembrane protein 192	31 kDa	0.053	8	8	0	4	3	1	0	0	0	0	0	-6.610140537	95% (0.0062)
FGFP1_HUMAN	GIMA1_HUMAN	GIMAP1	Q8WWP7	GTPase IMAP family member 1 (Immunity-associated protein 1) (hIMAP1)	34 kDa	0.11	8	8	0	0	2	2	3	0	0	0	0	-6.610140537	95% (0.0062)
A6ND99_HUMAN (+2)	TRPM4_HUMAN	TRPM4	Q8TD43	Transient receptor potential cation channel subfamily M member 4 (hTRPM4) (Calcium-activated non-selective cation channel 1) (Long transient receptor potential channel 4) (LTrpC-4) (LTrpC4) (Melastatin-4)	134 kDa	0.065	8	8	0	8	0	0	0	0	0	0	0	-6.610140537	95% (0.0062)

B0S7V1_HUMAN (+6)	B4DMR3_HUMAN		B4DMR3	cDNA FLJ51896, highly similar to Glia-derived nexin	37 kDa	0.086	8	8	0	1	3	4	0	0	0	0	0	-6.610140537	95% (0.0062)
Q860I7_HUMAN	G3V207_HUMAN	TMCC3	G3V207	Transmembrane and coiled-coil domain family 3, isoform CRA_c (Transmembrane and coiled-coil domains protein 3)	50 kDa	0.31	8	8	0	8	0	0	0	0	0	0	0	-6.610140537	95% (0.0062)
DOPP1_HUMAN	ANR22_HUMAN	ANKRD22	Q5VYY1	Ankyrin repeat domain-containing protein 22	22 kDa	0.084	8	8	0	2	0	1	5	0	0	0	0	-6.610140537	95% (0.0062)
BSPRY_HUMAN	B2RC75_HUMAN		B2RC75	Transmembrane channel-like protein	79 kDa	0.062	8	8	0	3	0	0	5	0	0	0	0	-6.610140537	95% (0.0062)
PYRG2_HUMAN	B4DKB2_HUMAN	ECE1	B4DKB2	Endothelin-converting enzyme 1 (cDNA FLJ59212, highly similar to Endothelin-converting enzyme 1 (EC 3.4.24.71))	84 kDa	0.048	42	38	4	11	13	15	0	3	0	1	0	-6.680389479	95% (< 0.00010)
B2RAH2_HUMAN (+1)	GSTM4_HUMAN	GSTM4	Q03013	Glutathione S-transferase Mu 4 (EC 2.5.1.18) (GST class-mu 4) (GST-Mu2) (GSTM4-4)	26 kDa	0.017	10	9	0	0	2	0	7	0	0	0	0	-7.324844443	95% (0.0033)
A5D8Z7_HUMAN (+1)	NCALD_HUMAN	NCALD	P61601	Neurocalcin-delta	22 kDa	0.017	12	9	0	4	0	0	2	0	0	0	0	-7.324844443	95% (0.0033)
B2RDG1_HUMAN (+3)	INADL_HUMAN	INADL	Q8NI35	InaD-like protein (Inadl protein) (hINADL) (Pals1-associated tight junction protein) (Protein associated to tight junctions)	196 kDa	0.025	10	10	0	9	0	0	1	0	0	0	0	-8.039566716	95% (0.0017)
GRPE2_HUMAN (+1)	J3QRN6_HUMAN	MYO1D	J3QRN6	Unconventional myosin-Id	111 kDa	0.12	10	10	0	10	0	0	0	0	0	0	0	-8.039566716	95% (0.0017)
ELP1_HUMAN (+2)	PRG2_HUMAN	PRG2	P13727	Bone marrow proteoglycan (BMPG) (Proteoglycan 2) [Cleaved into: Eosinophil granule major basic protein (EMBP) (MBP) (Pregnancy-associated major basic protein)]	25 kDa	0.029	10	10	0	4	0	0	6	0	0	0	0	-8.039566716	95% (0.0017)
B4DRC8_HUMAN (+1)	B4DZS8_HUMAN		B4DZS8	Peptidyl-prolyl cis-trans isomerase (Fragment)	98 kDa	0.075	10	10	0	0	4	6	0	0	0	0	0	-8.039566716	95% (0.0017)
VPS25_HUMAN	CB054_HUMAN	C2orf54	Q08AI8	Uncharacterized protein C2orf54	50 kDa	0.13	10	10	0	3	0	0	7	0	0	0	0	-8.039566716	95% (0.0017)
G3V145_HUMAN (+1)	E9PSH2_HUMAN	CD55	E9PSH2	Complement decay-accelerating factor	41 kDa	0.082	10	10	0	2	4	4	0	0	0	0	0	-8.039566716	95% (0.0017)
A6XNE0_HUMAN (+6)	B7ZB83_HUMAN		B7ZB83	cDNA, FLJ79447, highly similar to Nucleosome assembly protein 1-like 4	44 kDa	0.16	13	10	0	0	5	5	0	0	0	0	0	-8.039566716	95% (0.0017)
B2R533_HUMAN (+7)	IGHG2_HUMAN	IGHG2	P01859	Ig gamma-2 chain C region	36 kDa	0.12	10	10	0	0	0	0	6	0	0	0	0	-8.039566716	95% (0.0017)
B2R7K1_HUMAN (+1)	PTGIS_HUMAN	PTGIS	Q16647	Prostacyclin synthase (EC 5.3.99.4) (Prostaglandin I2 synthase)	57 kDa	0.067	43	40	3	35	0	0	5	0	0	0	3	-8.673090159	95% (< 0.00010)
B3KRF9_HUMAN (+1)	PADI2_HUMAN	PADI2	Q9Y2J8	Protein-arginine deiminase type-2 (EC 3.5.3.15) (PAD-H19) (Peptidylarginine deiminase II) (Protein-arginine deiminase type II)	76 kDa	0.1	11	11	0	0	5	6	0	0	0	0	0	-8.754307357	95% (0.00091)
PLCE_HUMAN (+1)	F6M9T7_HUMAN	IDO1	F6M9T7	Indolamine 2,3 dioxygenase (EC 1.13.11.52)	45 kDa	0.08	11	11	0	0	6	5	0	0	0	0	0	-8.754307357	95% (0.00091)
G3V4W4_HUMAN (+1)	PGH2_HUMAN	PTGS2	P35354	Prostaglandin G/H synthase 2 (EC 1.14.99.1) (Cyclooxygenase-2) (COX-2) (PHS II) (Prostaglandin H2 synthase 2) (PGH synthase 2) (PGHS-2) (Prostaglandin-endoperoxide synthase 2)	69 kDa	0.18	13	11	0	2	6	3	0	0	0	0	0	-8.754307357	95% (0.00091)
PCH2_HUMAN	PTBP3_HUMAN	PTBP3	O95758	Polypyrimidine tract-binding protein 3 (Regulator of differentiation 1) (Rod1)	60 kDa	0.06	23	12	0	0	4	0	3	0	0	0	0	-9.469066366	95% (0.00048)
WDR82_HUMAN	A8K2T7_HUMAN		A8K2T7	cDNA FLJ76780, highly similar to Homo sapiens epidermal growth factor receptor (erythroblastic	134 kDa	0.08	37	35	2	10	12	12	1	1	0	1	0	-9.966480545	95% (< 0.00010)

				leukemia viral (v-erb-b) oncogene homolog, avian) (EGFR), transcript variant 1															
B8K1J4_HUMAN (+1)	A8K7T0_HUMAN		A8K7T0	Kynureninase (EC 3.7.1.3)	52 kDa	0.13	13	13	0	0	6	8	0	0	0	0	0	-10.18384374	95% (0.00026)
ALG9_HUMAN (+3)	LY75_HUMAN	LY75	O60449	Lymphocyte antigen 75 (Ly-75) (C-type lectin domain family 13 member B) (DEC-205) (gp200-MR6) (CD antigen CD205)	198 kDa	0.047	14	14	0	1	4	9	0	0	0	0	0	-10.89863949	95% (0.00014)
MZB1_HUMAN	MYO5B_HUMAN	MYO5B	Q9ULV0	Unconventional myosin-Vb	214 kDa	0.13	14	14	0	12	2	0	0	0	0	0	0	-10.89863949	95% (0.00014)
F8WCD0_HUMAN (+1)	F13A_HUMAN	F13A1	P00488	Coagulation factor XIII A chain (Coagulation factor XIIIa) (EC 2.3.2.13) (Protein-glutamine gamma-glutamyltransferase A chain) (Transglutaminase A chain)	83 kDa	0.055	14	14	0	0	4	10	0	0	0	0	0	-10.89863949	95% (0.00014)
OSBL2_HUMAN	CDCP1_HUMAN	CDCP1	Q9H5V8	CUB domain-containing protein 1 (Membrane glycoprotein gp140) (Subtractive immunization M plus HEp3-associated 135 kDa protein) (SIMA135) (Transmembrane and associated with src kinases) (CD antigen CD318)	93 kDa	0.073	14	14	0	1	6	7	0	0	0	0	0	-10.89863949	95% (0.00014)
B2RE38_HUMAN (+2)	AHNK2_HUMAN	AHNAK2	Q8IVF2	Protein AHNAK2	617 kDa	0.028	15	15	0	15	0	0	0	0	0	0	0	-11.61345361	95% (< 0.00010)
CDS1_HUMAN	B4DYU3_HUMAN		B4DYU3	cDNA FLJ57235, highly similar to Aldehyde dehydrogenase 1A3 (EC 1.2.1.5)	49 kDa	0.043	45	42	2	11	16	16	0	0	0	2	0	-11.8921123	95% (< 0.00010)
A5D8W8_HUMAN (+1)	SPTA1_HUMAN	SPTA1	P02549	Spectrin alpha chain, erythrocytic 1 (Erythroid alpha-spectrin)	280 kDa	0.042	16	16	0	0	5	11	0	0	0	0	0	-12.3282861	95% (< 0.00010)
B4E126_HUMAN (+3)	B7Z416_HUMAN	DHRS9	B7Z416	Dehydrogenase/reductase SDR family member 9 (cDNA FLJ55219, highly similar to Dehydrogenase/reductase SDR family member 9 (EC 1.1.-.-))	42 kDa	0.065	48	46	2	18	18	18	18	0	0	0	2	-12.9926289	95% (< 0.00010)
DUS23_HUMAN	H0Y3C5_HUMAN	HCK	H0Y3C5	Tyrosine-protein kinase HCK	60 kDa	0.13	28	19	0	2	2	2	2	0	0	0	0	-14.47289381	95% (< 0.00010)
F8VX04_HUMAN (+2)	B0QZ43_HUMAN	ERLIN1	B0QZ43	Erlin-1 (Fragment)	31 kDa	0.038	23	19	0	7	4	3	0	0	0	0	0	-14.47289381	95% (< 0.00010)
PR38A_HUMAN	MX2_HUMAN	MX2	P20592	Interferon-induced GTP-binding protein Mx2 (Interferon-regulated resistance GTP-binding protein MxB) (Myxovirus resistance protein 2) (p78-related protein)	82 kDa	0.09	39	28	0	0	0	3	22	0	0	0	0	-20.90770921	95% (< 0.00010)
TP4A2_HUMAN	I6NXG5_HUMAN	HLA-C	I6NXG5	MHC class I antigen (Fragment)	21 kDa	0.14	64	42	0	0	11	7	16	0	0	0	0	-30.92038153	95% (< 0.00010)
B3KTN5_HUMAN (+2)	B7ZLH8_HUMAN	EVPL	B7ZLH8	EVPL protein	234 kDa	0.067	51	52	0	33	2	0	16	0	0	0	0	-38.07449707	95% (< 0.00010)
C9JFX4_HUMAN (+2)	Q860I7_HUMAN	HLA-B	Q860I7	MHC class I antigen	40 kDa	0.022	116	75	0	0	20	0	25	0	0	0	0	-54.53594588	95% (< 0.00010)

[A]Obtained from UniProt (http://www.uniprot.org/) databases

[B]Spectral counts (SpC) in ALL datasets (CN and CR)

[C]Spectral counts (SpC) in CN datasets (combined)

[D]Spectral counts (SpC) in CR datasets (combined)

[E]Spectral count ratio (Rsc) between CR (combined) and CN (combined)

[F]Statistical significance (p-value) (Fisher's Exact Test)

Table S3. Proteins differentially expressed in CN and CR tumor cells.

Uniprot Acc[A]	Gene Symbol[A]	Protein Description[A]	CN_Combined[C]	CR_Combined[D]	CN_1	CN_2	CN_3	CN_4	CR_1	CR_2	CR_3	CR_4	RSC (CR / CN)[E]	p value[F]
P23378	GLDC	Glycine dehydrogenase [decarboxylating], mitochondrial (EC 1.4.4.2) (Glycine cleavage system P protein) (Glycine decarboxylase)	0	21	0	0	0	0	20	0	0	2	19.9	95% (< 0.00010)
P21266	GSTM3	Glutathione S-transferase Mu 3 (EC 2.5.1.18) (GST class-mu 3) (GSTM3-3) (hGSTM3-3)	0	21	0	0	0	0	3	14	4	0	19.9	95% (< 0.00010)
Q56FN6	HLA-DRB1	MHC class II antigen	0	16	0	0	0	0	0	9	0	0	15.5	95% (< 0.00010)
Q13085	ACACA	Acetyl-CoA carboxylase 1 (ACC1) (EC 6.4.1.2) (ACC-alpha) [Includes: Biotin carboxylase (EC 6.3.4.14)]	0	13	0	0	0	0	12	0	0	0	12.8	95% (< 0.00010)
Q99715	COL12A1	Collagen alpha-1(XII) chain	1	23	0	0	1	0	12	1	6	4	12.1	95% (< 0.00010)
Q52NV4	HRS	Histidyl-tRNA synthetase	0	12	0	0	0	0	6	0	2	0	11.9	95% (0.00012)
C9J837	RAB3GAP1	Rab3 GTPase-activating protein catalytic subunit	0	11	0	0	0	0	0	9	2	0	11.0	95% (0.00025)
P08243	ASNS	Asparagine synthetase [glutamine-hydrolyzing] (EC 6.3.5.4) (Cell cycle control protein TS11) (Glutamine-dependent asparagine synthetase)	1	20	0	0	1	0	9	10	1	0	10.6	95% (< 0.00010)
P42771	CDKN2A	Cyclin-dependent kinase inhibitor 2A, isoforms 1/2/3 (Cyclin-dependent kinase 4 inhibitor A) (CDK4I) (Multiple tumor suppressor 1) (MTS-1) (p16-INK4a) (p16-INK4) (p16INK4A)	0	9	0	0	0	0	3	6	0	0	9.2	95% (0.0011)
B4DF41	MSH6	DNA mismatch repair protein Msh6 (cDNA FLJ55677, highly similar to DNA mismatch repair protein MSH6)	0	9	0	0	0	0	8	0	1	0	9.2	95% (0.0011)
B3KMS0		Condensin complex subunit 1	0	9	0	0	0	0	8	0	1	0	9.2	95% (0.0011)
Q86YH3	EARS2	EARS2 protein	0	8	0	0	0	0	1	0	2	4	8.3	95% (0.0024)
D6RE83	UCHL1	Ubiquitin carboxyl-terminal hydrolase isozyme L1	0	8	0	0	0	0	0	5	3	0	8.3	95% (0.0024)

Q04828	AKR1C1	Aldo-keto reductase family 1 member C1 (EC 1.1.1.-) (20-alpha-hydroxysteroid dehydrogenase) (20-alpha-HSD) (EC 1.1.1.149) (Chlordecone reductase homolog HAKRC) (Dihydrodiol dehydrogenase 1/2) (DD1/DD2) (High-affinity hepatic bile acid-binding protein) (HBAB) (Indanol dehydrogenase) (EC 1.1.1.112) (Trans-1,2-dihydrobenzene-1,2-diol dehydrogenase) (EC 1.3.1.20)	0	7	0	0	0	0	0	6	1	0	7.4	95% (0.0051)
P55285	CDH6	Cadherin-6 (Kidney cadherin) (K-cadherin)	0	7	0	0	0	0	4	0	1	2	7.4	95% (0.0051)
P37268	FDFT1	Squalene synthase (SQS) (SS) (EC 2.5.1.21) (FPP:FPP farnesyltransferase) (Farnesyl-diphosphate farnesyltransferase)	1	12	1	0	0	0	9	1	2	0	6.6	95% (0.00087)
P36405	ARL3	ADP-ribosylation factor-like protein 3	0	6	0	0	0	0	1	4	1	0	6.5	95% (0.011)
B4DIH5	COPS2	COP9 signalosome complex subunit 2 (cDNA FLJ52928, highly similar to COP9 signalosome complex subunit 2)	0	6	0	0	0	0	3	2	1	0	6.5	95% (0.011)
D3DNT9	EPHB3	EPH receptor B3, isoform CRA_a	0	6	0	0	0	0	4	0	2	0	6.5	95% (0.011)
P21397	MAOA	Amine oxidase [flavin-containing] A (EC 1.4.3.4) (Monoamine oxidase type A) (MAO-A)	0	6	0	0	0	0	0	3	0	3	6.5	95% (0.011)
Q14669	TRIP12	E3 ubiquitin-protein ligase TRIP12 (EC 6.3.2.-) (E3 ubiquitin-protein ligase for Arf) (ULF) (Thyroid receptor-interacting protein 12) (TR-interacting protein 12) (TRIP-12)	0	6	0	0	0	0	2	0	4	0	6.5	95% (0.011)
A8K984		Structural maintenance of chromosomes protein	0	6	0	0	0	0	4	0	1	0	6.5	95% (0.011)
O60701	UGDH	UDP-glucose 6-dehydrogenase (UDP-Glc dehydrogenase) (UDP-GlcDH) (UDPGDH) (EC 1.1.1.22)	3	23	1	1	1	0	2	15	6	0	6.4	95% (< 0.00010)
Q96C36	PYCR2	Pyrroline-5-carboxylate reductase 2 (P5C reductase 2) (P5CR 2) (EC 1.5.1.2)	4	26	0	0	0	3	12	12	12	12	5.8	95% (< 0.00010)
Q9UKF6	CPSF3	Cleavage and polyadenylation specificity factor subunit 3 (EC 3.1.27.-) (Cleavage and polyadenylation specificity factor 73 kDa subunit) (CPSF 73 kDa subunit) (mRNA 3'-end-processing endonuclease CPSF-73)	1	10	0	0	1	0	1	0	7	2	5.6	95% (0.0034)

B4DHQ3		Phosphoserine aminotransferase (EC 2.6.1.52)	1	10	0	0	0	1	5	4	1	0	5.6	95% (0.0034)
P11766	ADH5	Alcohol dehydrogenase class-3 (EC 1.1.1.1) (Alcohol dehydrogenase 5) (Alcohol dehydrogenase class chi chain) (Alcohol dehydrogenase class-III) (Glutathione-dependent formaldehyde dehydrogenase) (FALDH) (FDH) (GSH-FDH) (EC 1.1.1.-) (S-(hydroxymethyl)glutathione dehydrogenase) (EC 1.1.1.284)	0	5	0	0	0	0	2	3	0	0	5.6	95% (0.023)
B7ZMD7	AMY1A	Amylase, alpha 1A (Salivary)	0	5	0	0	0	0	0	5	0	0	5.6	95% (0.023)
O14965	AURKA	Aurora kinase A (EC 2.7.11.1) (Aurora 2) (Aurora/IPL1-related kinase 1) (ARK-1) (Aurora-related kinase 1) (hARK1) (Breast tumor-amplified kinase) (Serine/threonine-protein kinase 15) (Serine/threonine-protein kinase 6) (Serine/threonine-protein kinase aurora-A)	0	5	0	0	0	0	0	3	1	0	5.6	95% (0.023)
Q96GD4	AURKB	Aurora kinase B (EC 2.7.11.1) (Aurora 1) (Aurora- and IPL1-like midbody-associated protein 1) (AIM-1) (Aurora/IPL1-related kinase 2) (ARK-2) (Aurora-related kinase 2) (STK-1) (Serine/threonine-protein kinase 12) (Serine/threonine-protein kinase 5) (Serine/threonine-protein kinase aurora-B)	0	5	0	0	0	0	1	0	4	0	5.6	95% (0.023)
O43708	GSTZ1	Maleylacetoacetate isomerase (MAAI) (EC 5.2.1.2) (GSTZ1-1) (Glutathione S-transferase zeta 1) (EC 2.5.1.18)	0	5	0	0	0	0	2	1	2	0	5.6	95% (0.023)
Q86Y56	HEATR2	HEAT repeat-containing protein 2	0	5	0	0	0	0	1	2	1	0	5.6	95% (0.023)
Q68E01	INTS3	Integrator complex subunit 3 (Int3) (SOSS complex subunit A) (Sensor of single-strand DNA complex subunit A) (SOSS-A) (Sensor of ssDNA subunit A)	0	5	0	0	0	0	1	1	2	1	5.6	95% (0.023)
Q15397	KIAA0020	Pumilio domain-containing protein KIAA0020 (HBV X-transactivated gene 5 protein) (HBV XAg-transactivated protein 5) (Minor histocompatibility antigen HA-8) (HLA-HA8)	0	5	0	0	0	0	2	2	1	0	5.6	95% (0.023)

E7ESY4	MTA1	Metastasis-associated protein MTA1	0	5	0	0	0	0	1	0	3	0	5.6	95% (0.023)
Q9Y263	PLAA	Phospholipase A-2-activating protein (PLA2P) (PLAP)	0	5	0	0	0	0	2	3	0	0	5.6	95% (0.023)
E9PKF6	PPP6R3	Serine/threonine-protein phosphatase 6 regulatory subunit 3	0	5	0	0	0	0	0	4	1	0	5.6	95% (0.023)
Q9C0C9	UBE2O	Ubiquitin-conjugating enzyme E2 O (EC 6.3.2.19) (Ubiquitin carrier protein O) (Ubiquitin-conjugating enzyme E2 of 230 kDa) (Ubiquitin-conjugating enzyme E2-230K) (Ubiquitin-protein ligase O)	0	5	0	0	0	0	0	4	1	0	5.6	95% (0.023)
Q59GW6		Acetyl-CoA acetyltransferase, cytosolic variant (Fragment)	0	5	0	0	0	0	5	0	0	0	5.6	95% (0.023)
Q8NFF5	FLAD1	FAD synthase (EC 2.7.7.2) (FAD pyrophosphorylase) (FMN adenylyltransferase) (Flavin adenine dinucleotide synthase) [Includes: Molybdenum cofactor biosynthesis protein-like region; FAD synthase region]	3	19	0	0	0	3	11	2	3	3	5.3	95% (0.00016)
Q9Y5Q9	GTF3C3	General transcription factor 3C polypeptide 3 (Transcription factor IIIC 102 kDa subunit) (TFIIIC 102 kDa subunit) (TFIIIC102) (Transcription factor IIIC subunit gamma) (TF3C-gamma)	1	9	0	0	0	1	4	0	3	2	5.1	95% (0.0065)
O15230	LAMA5	Laminin subunit alpha-5 (Laminin-10 subunit alpha) (Laminin-11 subunit alpha) (Laminin-15 subunit alpha)	1	9	1	0	0	0	3	1	3	2	5.1	95% (0.0065)
Q6UX53	METTL7B	Methyltransferase-like protein 7B (EC 2.1.1.-)	1	9	1	0	0	0	6	1	1	1	5.1	95% (0.0065)
P52569	SLC7A2	Low affinity cationic amino acid transporter 2 (CAT-2) (CAT2) (Solute carrier family 7 member 2)	1	9	1	0	0	0	1	3	2	3	5.1	95% (0.0065)
J3KR97	TBCD	Tubulin-specific chaperone D	1	9	0	1	0	0	1	7	1	0	5.1	95% (0.0065)
B4DMB5		CTP synthase (EC 6.3.4.2) (UTP--ammonia ligase)	1	9	0	0	0	1	5	5	0	0	5.1	95% (0.0065)
P50851	LRBA	Lipopolysaccharide-responsive and beige-like anchor protein (Beige-like protein) (CDC4-like protein)	3	18	3	0	0	0	5	9	3	2	5.1	95% (0.00029)
Q9NZJ6	COQ3	Hexaprenyldihydroxybenzoate methyltransferase, mitochondrial (EC 2.1.1.114) (2-polyprenyl-6-hydroxyphenol	0	4	0	0	0	0	4	0	0	0	4.7	95% (0.049)

		methylase) (EC 2.1.1.222) (3,4-dihydroxy-5-hexaprenylbenzoate methyltransferase) (DHHB methyltransferase) (DHHB-MT) (DHHB-MTase) (3-demethylubiquinone-10 3-methyltransferase) (EC 2.1.1.64) (Dihydroxyhexaprenylbenzoate methyltransferase)												
P29762	CRABP1	Cellular retinoic acid-binding protein 1 (Cellular retinoic acid-binding protein I) (CRABP-I)	0	4	0	0	0	0	0	4	0	0	4.7	95% (0.049)
P02741	CRP	C-reactive protein [Cleaved into: C-reactive protein(1-205)]	0	4	0	0	0	0	0	0	0	4	4.7	95% (0.049)
P48507	GCLM	Glutamate--cysteine ligase regulatory subunit (GCS light chain) (Gamma-ECS regulatory subunit) (Gamma-glutamylcysteine synthetase regulatory subunit) (Glutamate--cysteine ligase modifier subunit)	0	4	0	0	0	0	0	4	0	0	4.7	95% (0.049)
Q96IJ6	GMPPA	Mannose-1-phosphate guanyltransferase alpha (EC 2.7.7.13) (GDP-mannose pyrophosphorylase A) (GTP-mannose-1-phosphate guanylyltransferase alpha)	0	4	0	0	0	0	0	2	1	1	4.7	95% (0.049)
Q9BYC9	MRPL20	39S ribosomal protein L20, mitochondrial (L20mt) (MRP-L20)	0	4	0	0	0	0	1	1	0	2	4.7	95% (0.049)
Q9UKX7	NUP50	Nuclear pore complex protein Nup50 (50 kDa nucleoporin) (Nuclear pore-associated protein 60 kDa-like) (Nucleoporin Nup50)	0	4	0	0	0	0	2	0	2	0	4.7	95% (0.049)
E7EU94	PRPF31	U4/U6 small nuclear ribonucleoprotein Prp31 (Fragment)	0	4	0	0	0	0	2	2	0	0	4.7	95% (0.049)
P26022	PTX3	Pentraxin-related protein PTX3 (Pentaxin-related protein PTX3) (Tumor necrosis factor alpha-induced protein 5) (TNF alpha-induced protein 5) (Tumor necrosis factor-inducible gene 14 protein) (TSG-14)	0	4	0	0	0	0	1	0	3	0	4.7	95% (0.049)
Q96AH8	RAB7B	Ras-related protein Rab-7b	0	4	0	0	0	0	0	0	0	4	4.7	95% (0.049)
J3KNH7	SENP3	Sentrin-specific protease 3	0	4	0	0	0	0	2	0	2	0	4.7	95% (0.049)
Q96K37	SLC35E1	Solute carrier family 35 member E1	0	4	0	0	0	0	0	2	0	2	4.7	95% (0.049)
Q15599	SLC9A3R2	Na(+)/H(+) exchange regulatory cofactor NHE-RF2 (NHERF-2) (NHE3 kinase A regulatory protein E3KARP) (SRY-	0	4	0	0	0	0	2	0	1	1	4.7	95% (0.049)

		interacting protein 1) (SIP-1) (Sodium-hydrogen exchanger regulatory factor 2) (Solute carrier family 9 isoform A3 regulatory factor 2) (Tyrosine kinase activator protein 1) (TKA-1)											
A8KAM9		Peptidyl-prolyl cis-trans isomerase E (PPIase E) (EC 5.2.1.8)	0	4	0	0	0	0	2	0	2	0	4.7	95% (0.049)
Q96HN2	AHCYL2	Putative adenosylhomocysteinase 3 (AdoHcyase 3) (EC 3.3.1.1) (S-adenosyl-L-homocysteine hydrolase 3) (S-adenosylhomocysteine hydrolase-like protein 2)	1	8	0	0	0	1	2	2	4	0	4.6	95% (0.013)
O94766	B3GAT3	Galactosylgalactosylxylosylprotein 3-beta-glucuronosyltransferase 3 (EC 2.4.1.135) (Beta-1,3-glucuronyltransferase 3) (Glucuronosyltransferase I) (GlcAT-I) (UDP-GlcUA:Gal beta-1,3-Gal-R glucuronyltransferase) (GlcUAT-I)	1	8	0	0	0	1	2	1	2	3	4.6	95% (0.013)
O75208	COQ9	Ubiquinone biosynthesis protein COQ9, mitochondrial	1	8	1	0	0	0	6	0	2	0	4.6	95% (0.013)
P14324	FDPS	Farnesyl pyrophosphate synthase (FPP synthase) (FPS) (EC 2.5.1.10) ((2E,6E)-farnesyl diphosphate synthase) (Dimethylallyltranstransferase) (EC 2.5.1.1) (Farnesyl diphosphate synthase) (Geranyltranstransferase)	1	8	0	1	0	0	7	0	1	0	4.6	95% (0.013)
B4E1G6	GALK1	Galactokinase (Galactokinase 1) (cDNA FLJ56840, highly similar to Galactokinase (EC 2.7.1.6))	1	8	0	1	0	0	0	6	2	0	4.6	95% (0.013)
A0JP11	PIK3R4	Phosphoinositide-3-kinase, regulatory subunit 4	1	8	0	1	0	0	5	2	1	0	4.6	95% (0.013)
P63151	PPP2R2A	Serine/threonine-protein phosphatase 2A 55 kDa regulatory subunit B alpha isoform (PP2A subunit B isoform B55-alpha) (PP2A subunit B isoform PR55-alpha) (PP2A subunit B isoform R2-alpha) (PP2A subunit B isoform alpha)	1	8	1	0	0	0	3	4	2	0	4.6	95% (0.013)
P98175	RBM10	RNA-binding protein 10 (G patch domain-containing protein 9) (RNA-binding motif	1	8	0	0	0	1	1	0	4	3	4.6	95% (0.013)

		protein 10) (RNA-binding protein S1-1) (S1-1)											
E1NZA1	PRIC295	Peroxisome proliferator activated receptor interacting complex protein	21	90	4	2	2	13	40	38	12	2	4.6	95% (< 0.00010)
Q8IWA0	WDR75	WD repeat-containing protein 75	2	12	0	2	0	0	9	0	3	0	4.6	95% (0.0035)
P36222	CHI3L1	Chitinase-3-like protein 1 (39 kDa synovial protein) (Cartilage glycoprotein 39) (CGP-39) (GP-39) (hCGP-39) (YKL-40)	5	24	3	1	0	1	2	1	3	18	4.5	95% (< 0.00010)
Q8TD30	GPT2	Alanine aminotransferase 2 (ALT2) (EC 2.6.1.2) (Glutamate pyruvate transaminase 2) (GPT 2) (Glutamic--alanine transaminase 2) (Glutamic--pyruvic transaminase 2)	5	24	0	1	1	3	14	9	1	0	4.5	95% (< 0.00010)
Q92626	PXDN	Peroxidasin homolog (EC 1.11.1.7) (Melanoma-associated antigen MG50) (Vascular peroxidase 1) (p53-responsive gene 2 protein)	4	19	4	0	0	0	10	1	2	6	4.3	95% (0.00051)
P61221	ABCE1	ATP-binding cassette sub-family E member 1 (2'-5'-oligoadenylate-binding protein) (HuHP68) (RNase L inhibitor) (Ribonuclease 4 inhibitor) (RNS4I)	3	15	0	0	0	3	7	4	4	0	4.3	95% (0.0018)
Q8IZV5	RDH10	Retinol dehydrogenase 10 (EC 1.1.1.300)	2	11	1	0	1	0	0	9	1	1	4.2	95% (0.0064)
Q8NBL1	POGLUT1	Protein O-glucosyltransferase 1 (EC 2.4.1.-) (CAP10-like 46 kDa protein) (hCLP46) (KTEL motif-containing protein 1) (Myelodysplastic syndromes relative protein)	4	18	0	2	1	1	8	4	2	5	4.1	95% (0.00090)
Q92905	COPS5	COP9 signalosome complex subunit 5 (SGN5) (Signalosome subunit 5) (EC 3.4.-.-) (Jun activation domain-binding protein 1)	1	7	0	0	0	1	4	1	2	0	4.1	95% (0.024)
B7Z5H3	MAOB	Amine oxidase [flavin-containing] B (cDNA FLJ52418, highly similar to Amine oxidase (flavin-containing) B (EC 1.4.3.4))	1	7	0	0	0	1	0	7	0	0	4.1	95% (0.024)
Q9Y266	NUDC	Nuclear migration protein nudC (Nuclear distribution protein C homolog)	1	7	0	0	0	1	5	0	2	0	4.1	95% (0.024)
P00738	HP	Haptoglobin (Zonulin) [Cleaved into: Haptoglobin alpha chain; Haptoglobin beta chain]	3	14	0	2	0	1	0	2	5	6	4.0	95% (0.0032)

P02748	C9	Complement component C9 [Cleaved into: Complement component C9a; Complement component C9b]	2	10	0	0	0	2	0	0	0	10	3.9	95% (0.012)
Q9BSD7	NTPCR	Cancer-related nucleoside-triphosphatase (NTPase) (EC 3.6.1.15) (Nucleoside triphosphate phosphohydrolase)	2	10	2	0	0	0	1	2	0	7	3.9	95% (0.012)
Q14151	SAFB2	Scaffold attachment factor B2 (SAF-B2)	2	10	2	0	0	0	6	0	2	2	3.9	95% (0.012)
P11388	TOP2A	DNA topoisomerase 2-alpha (EC 5.99.1.3) (DNA topoisomerase II, alpha isozyme)	20	68	0	9	9	2	26	18	14	10	3.7	95% (< 0.00010)
Q5JTZ9	AARS2	Alanine--tRNA ligase, mitochondrial (EC 6.1.1.7) (Alanyl-tRNA synthetase) (AlaRS)	1	6	1	0	0	0	5	0	0	1	3.6	95% (0.045)
B4DQ14	EIF2A	Eukaryotic translation initiation factor 2A (cDNA FLJ58035, highly similar to Homo sapiens eukaryotic translation initiation factor (eIF) 2A (eIF2A), mRNA)	1	6	0	0	0	1	3	3	1	0	3.6	95% (0.045)
Q14232	EIF2B1	Translation initiation factor eIF-2B subunit alpha (eIF-2B GDP-GTP exchange factor subunit alpha)	1	6	0	0	0	1	3	3	0	0	3.6	95% (0.045)
Q96ND0	FAM210A	Protein FAM210A	1	6	0	0	1	0	1	2	2	1	3.6	95% (0.045)
P82675	MRPS5	28S ribosomal protein S5, mitochondrial (MRP-S5) (S5mt)	1	6	1	0	0	0	1	0	1	4	3.6	95% (0.045)
B4DU97	SLC25A17	Peroxisomal membrane protein PMP34 (cDNA FLJ57596, highly similar to Peroxisomal membrane protein PMP34)	1	6	0	1	0	0	2	4	0	0	3.6	95% (0.045)
Q8NCA5	FAM98A	Protein FAM98A	2	9	0	0	0	2	3	3	1	2	3.5	95% (0.021)
Q69YN4	KIAA1429	Protein virilizer homolog	2	9	0	1	1	0	2	0	7	0	3.5	95% (0.021)
Q96T76	MMS19	MMS19 nucleotide excision repair protein homolog (hMMS19) (MET18 homolog) (MMS19-like protein)	2	9	0	0	0	2	2	5	1	0	3.5	95% (0.021)
P05165	PCCA	Propionyl-CoA carboxylase alpha chain, mitochondrial (PCCase subunit alpha) (EC 6.4.1.3) (Propanoyl-CoA:carbon dioxide ligase subunit alpha)	2	9	0	0	1	1	3	0	1	5	3.5	95% (0.021)
P49588	AARS	Alanine--tRNA ligase, cytoplasmic (EC 6.1.1.7) (Alanyl-tRNA synthetase) (AlaRS) (Renal carcinoma antigen NY-REN-42)	11	37	0	2	4	5	15	18	6	0	3.5	95% (< 0.00010)
Q13131	PRKAA1	5'-AMP-activated protein kinase catalytic subunit alpha-1 (AMPK subunit alpha-1) (EC 2.7.11.1) (Acetyl-CoA carboxylase	3	12	0	2	1	0	5	5	2	0	3.5	95% (0.0098)

		kinase) (ACACA kinase) (EC 2.7.11.27) (Hydroxymethylglutaryl-CoA reductase kinase) (HMGCR kinase) (EC 2.7.11.31) (Tau-protein kinase PRKAA1) (EC 2.7.11.26)												
B9A018	USP39	U4/U6.U5 tri-snRNP-associated protein 2	3	12	1	0	2	0	0	0	11	1	3.5	95% (0.0098)
Q59FH1		Transformation/transcription domain-associated protein variant (Fragment)	3	12	1	0	0	2	2	0	9	1	3.5	95% (0.0098)
Q9H3U1	UNC45A	Protein unc-45 homolog A (Unc-45A) (GCUNC-45) (Smooth muscle cell-associated protein 1) (SMAP-1)	4	15	0	0	0	4	2	8	4	0	3.5	95% (0.0047)
E7EUY3	PCCB	Propionyl-CoA carboxylase beta chain, mitochondrial	9	29	1	3	2	3	17	1	3	9	3.3	95% (0.00023)
M1V485	SLC34A2-ROS1	Tyrosine-protein kinase receptor (EC 2.7.10.1)	13	40	4	4	2	3	8	1	14	18	3.2	95% (< 0.00010)
Q96KA5	CLPTM1L	Cleft lip and palate transmembrane protein 1-like protein (CLPTM1-like protein) (Cisplatin resistance-related protein 9) (CRR9p)	2	8	0	0	2	0	2	0	4	2	3.2	95% (0.037)
A7J992	PHIP	Pleckstrin homology domain-interacting protein variant 1	2	8	0	1	0	1	4	0	3	1	3.2	95% (0.037)
Q96T51	RUFY1	RUN and FYVE domain-containing protein 1 (FYVE-finger protein EIP1) (La-binding protein 1) (Rab4-interacting protein) (Zinc finger FYVE domain-containing protein 12)	2	8	1	0	0	1	3	2	3	0	3.2	95% (0.037)
B4DHJ3		DNA-directed RNA polymerase (EC 2.7.7.6)	6	19	0	2	1	3	2	2	12	3	3.1	95% (0.0031)
K7ELW0	PARK7	Protein DJ-1	5	16	1	1	2	1	5	5	3	3	3.1	95% (0.0065)
P50895	BCAM	Basal cell adhesion molecule (Auberger B antigen) (B-CAM cell surface glycoprotein) (F8/G253 antigen) (Lutheran antigen) (Lutheran blood group glycoprotein) (CD antigen CD239)	11	32	0	2	1	8	14	0	16	2	3.0	95% (0.00025)
Q6IAX9	SRPR	SRPR protein	17	48	0	5	6	6	18	12	15	4	3.0	95% (< 0.00010)
F8VPD4	CAD	CAD protein	10	29	3	2	2	3	17	7	4	1	3.0	95% (0.00049)
O00468	AGRN	Agrin [Cleaved into: Agrin N-terminal 110 kDa subunit; Agrin C-terminal 110 kDa	3	10	0	2	0	1	8	0	2	0	3.0	95% (0.029)

		subunit; Agrin C-terminal 90 kDa fragment (C90); Agrin C-terminal 22 kDa fragment (C22)]												
F5GZS0	DHX36	Probable ATP-dependent RNA helicase DHX36	3	10	0	1	2	0	5	2	2	1	3.0	95% (0.029)
B4DJK9		Perilipin	3	10	1	0	1	1	0	1	3	6	3.0	95% (0.029)
Q6NVW7	KPNA2	Importin subunit alpha	13	36	0	3	8	2	5	8	18	5	2.9	95% (0.00016)
P18583	SON	Protein SON (Bax antagonist selected in saccharomyces 1) (BASS1) (Negative regulatory element-binding protein) (NRE-binding protein) (Protein DBP-5) (SON3)	5	15	0	1	3	1	6	0	6	3	2.9	95% (0.011)
E7D7X9		Pyrroline-5-carboxylate reductase (EC 1.5.1.2)	5	15	0	2	2	1	5	6	4	0	2.9	95% (0.011)
Q53GS0		Nucleolar GTP-binding protein 1 (Fragment)	4	12	1	0	0	2	6	2	4	0	2.8	95% (0.023)
P22102	GART	Trifunctional purine biosynthetic protein adenosine-3 [Includes: Phosphoribosylamine--glycine ligase (EC 6.3.4.13) (Glycinamide ribonucleotide synthetase) (GARS) (Phosphoribosylglycinamide synthetase); Phosphoribosylformylglycinamidine cyclo-ligase (EC 6.3.3.1) (AIR synthase) (AIRS) (Phosphoribosyl-aminoimidazole synthetase); Phosphoribosylglycinamide formyltransferase (EC 2.1.2.2) (5'-phosphoribosylglycinamide transformylase) (GAR transformylase) (GART)]	8	22	0	1	1	6	9	9	4	0	2.8	95% (0.0032)
Q6IBU0	EIF5	EIF5 protein	5	14	0	2	2	1	3	7	4	0	2.7	95% (0.017)
A2RTY6	ITIH2	Inter-alpha (Globulin) inhibitor H2 (Inter-alpha (Globulin) inhibitor H2, isoform CRA_b) (cDNA FLJ75038, highly similar to Homo sapiens inter-alpha (globulin) inhibitor H2 (ITIH2), mRNA)	3	9	0	1	1	1	2	4	1	2	2.7	95% (0.049)
B4E1K0	KIF23	Kinesin-like protein KIF23 (cDNA FLJ58416, highly similar to Kinesin-like protein KIF23)	3	9	0	2	1	0	2	1	4	2	2.7	95% (0.049)

Q06AH7	TF	Transferrin	34	83	1	11	10	12	27	13	30	14	2.7	95% (< 0.00010)
Q13057	COASY	Bifunctional coenzyme A synthase (CoA synthase) (NBP) (POV-2) [Includes: Phosphopantetheine adenylyltransferase (EC 2.7.7.3) (Dephospho-CoA pyrophosphorylase) (Pantetheine-phosphate adenylyltransferase) (PPAT); Dephospho-CoA kinase (DPCK) (EC 2.7.1.24) (Dephosphocoenzyme A kinase) (DPCOAK)]	4	11	0	0	1	3	5	3	2	1	2.6	95% (0.037)
O95864	FADS2	Fatty acid desaturase 2 (EC 1.14.19.-) (Delta(6) fatty acid desaturase) (D6D) (Delta(6) desaturase) (Delta-6 desaturase)	4	11	0	2	2	0	2	0	9	0	2.6	95% (0.037)
J3KN16	KIAA0368	Proteasome-associated protein ECM29 homolog	4	11	0	0	0	4	3	4	4	0	2.6	95% (0.037)
E9PF74	SLC25A19	Mitochondrial thiamine pyrophosphate carrier	4	11	0	0	1	3	1	2	1	7	2.6	95% (0.037)
Q7L014	DDX46	Probable ATP-dependent RNA helicase DDX46 (EC 3.6.4.13) (DEAD box protein 46) (PRP5 homolog)	11	27	0	2	2	6	5	4	14	4	2.6	95% (0.0024)
P68104	EEF1A1	Elongation factor 1-alpha 1 (EF-1-alpha-1) (Elongation factor Tu) (EF-Tu) (Eukaryotic elongation factor 1 A-1) (eEF1A-1) (Leukocyte receptor cluster member 7)	96	222	13	28	24	32	26	137	40	22	2.6	95% (< 0.00010)
Q9H5Q4	TFB2M	Dimethyladenosine transferase 2, mitochondrial (EC 2.1.1.-) (Hepatitis C virus NS5A-transactivated protein 5) (HCV NS5A-transactivated protein 5) (Mitochondrial 12S rRNA dimethylase 2) (Mitochondrial transcription factor B2) (h-mtTFB) (h-mtTFB2) (hTFB2M) (mtTFB2) (S-adenosylmethionine-6-N', N'-adenosyl(rRNA) dimethyltransferase 2)	5	13	1	1	2	1	6	2	1	4	2.6	95% (0.028)
P01023	A2M	Alpha-2-macroglobulin (Alpha-2-M) (C3 and PZP-like alpha-2-macroglobulin domain-containing protein 5)	9	22	0	1	7	1	2	1	6	12	2.5	95% (0.0062)
Q0VGA5	SARS	SARS protein	9	22	0	0	1	8	7	9	6	0	2.5	95% (0.0062)
Q5TC12	ATPAF1	ATP synthase mitochondrial F1 complex assembly factor 1 (ATP11 homolog)	6	15	1	2	2	1	10	2	3	0	2.5	95% (0.021)

P14174	MIF	Macrophage migration inhibitory factor (MIF) (EC 5.3.2.1) (Glycosylation-inhibiting factor) (GIF) (L-dopachrome isomerase) (L-dopachrome tautomerase) (EC 5.3.3.12) (Phenylpyruvate tautomerase)	8	19	1	3	4	0	4	7	5	3	2.5	95% (0.012)
P07741	APRT	Adenine phosphoribosyltransferase (APRT) (EC 2.4.2.7)	14	32	1	3	5	5	9	15	4	4	2.4	95% (0.0017)
Q8NCN5	PDPR	Pyruvate dehydrogenase phosphatase regulatory subunit, mitochondrial (PDPr)	14	32	2	5	6	1	21	2	1	9	2.4	95% (0.0017)
Q9H0S4	DDX47	Probable ATP-dependent RNA helicase DDX47 (EC 3.6.4.13) (DEAD box protein 47)	9	21	0	1	5	3	4	7	6	4	2.4	95% (0.0095)
P45974	USP5	Ubiquitin carboxyl-terminal hydrolase 5 (EC 3.4.19.12) (Deubiquitinating enzyme 5) (Isopeptidase T) (Ubiquitin thioesterase 5) (Ubiquitin-specific-processing protease 5)	10	23	0	0	3	7	6	15	3	0	2.4	95% (0.0073)
Q8NI27	THOC2	THO complex subunit 2 (Tho2) (hTREX120)	11	25	1	2	4	3	7	1	12	5	2.4	95% (0.0056)
Q5W0H4	TPT1	Translationally-controlled tumor protein	11	25	2	4	3	1	8	8	6	3	2.4	95% (0.0056)
O14976	GAK	Cyclin-G-associated kinase (EC 2.7.11.1)	5	12	0	1	2	3	3	6	3	0	2.4	95% (0.044)
Q9Y4W2	LAS1L	Ribosomal biogenesis protein LAS1L (Protein LAS1 homolog)	5	12	2	0	0	3	6	2	0	5	2.4	95% (0.044)
A5PLK7	RCC2	RCC2 protein (Fragment)	6	14	0	1	1	4	8	4	2	0	2.4	95% (0.033)
Q6URC4		Diaphanous 1	7	16	0	3	3	1	7	2	5	1	2.3	95% (0.025)
Q53GX7		Threonyl-tRNA synthetase variant (Fragment)	19	41	0	6	5	8	15	13	9	4	2.3	95% (0.00071)
O15254	ACOX3	Peroxisomal acyl-coenzyme A oxidase 3 (EC 1.3.3.6) (Branched-chain acyl-CoA oxidase) (BRCACox) (Pristanoyl-CoA oxidase)	8	18	7	0	1	0	1	8	3	6	2.3	95% (0.019)
Q9UIG0	BAZ1B	Tyrosine-protein kinase BAZ1B (EC 2.7.10.2) (Bromodomain adjacent to zinc finger domain protein 1B) (Williams syndrome transcription factor) (Williams-Beuren syndrome chromosomal region 10 protein) (Williams-Beuren syndrome chromosomal region 9 protein) (hWALp2)	8	18	2	2	0	5	14	0	3	1	2.3	95% (0.019)

P61289	PSME3	Proteasome activator complex subunit 3 (11S regulator complex subunit gamma) (REG-gamma) (Activator of multicatalytic protease subunit 3) (Ki nuclear autoantigen) (Proteasome activator 28 subunit gamma) (PA28g) (PA28gamma)	8	18	0	1	1	6	4	6	8	1	2.3	95% (0.019)
P10909	CLU	Clusterin (Aging-associated gene 4 protein) (Apolipoprotein J) (Apo-J) (Complement cytolysis inhibitor) (CLI) (Complement-associated protein SP-40,40) (Ku70-binding protein 1) (NA1/NA2) (Testosterone-repressed prostate message 2) (TRPM-2) [Cleaved into: Clusterin beta chain (ApoJalpha) (Complement cytolysis inhibitor a chain); Clusterin alpha chain (ApoJbeta) (Complement cytolysis inhibitor b chain)]	9	20	3	1	4	1	1	8	3	7	2.3	95% (0.014)
Q969X5	ERGIC1	Endoplasmic reticulum-Golgi intermediate compartment protein 1 (ER-Golgi intermediate compartment 32 kDa protein) (ERGIC-32)	9	20	7	0	0	2	2	6	7	5	2.3	95% (0.014)
P18428	LBP	Lipopolysaccharide-binding protein (LBP)	9	20	0	2	1	6	0	0	4	15	2.3	95% (0.014)
Q4LE36	ACLY	ACLY variant protein (Fragment)	31	64	0	12	9	10	28	24	11	2	2.3	95% (< 0.00010)
Q96RP9	GFM1	Elongation factor G, mitochondrial (EF-Gmt) (Elongation factor G 1, mitochondrial) (mEF-G 1) (Elongation factor G1) (hEFG1)	33	68	4	12	10	8	37	6	10	15	2.3	95% (< 0.00010)
B5BTZ6	STAT3	Signal transducer and activator of transcription	20	41	3	7	3	7	12	21	9	0	2.2	95% (0.0012)
P54577	YARS	Tyrosine--tRNA ligase, cytoplasmic (EC 6.1.1.1) (Tyrosyl-tRNA synthetase) (TyrRS) [Cleaved into: Tyrosine--tRNA ligase, cytoplasmic, N-terminally processed]	9	19	0	3	3	3	7	8	3	1	2.2	95% (0.022)
P11172	UMPS	Uridine 5'-monophosphate synthase (UMP synthase) [Includes: Orotate phosphoribosyltransferase (OPRT) (OPRTase) (EC 2.4.2.10); Orotidine 5'-	8	17	0	2	5	1	12	2	3	1	2.2	95% (0.029)

		phosphate decarboxylase (ODC) (EC 4.1.1.23) (OMPdecase)]											
Q53FW8		TAP binding protein-like variant (Fragment)	7	15	2	0	0	6	0	11	0	5	2.2	95% (0.038)
Q53G19		Mitochondrial ribosomal protein L11 isoform a variant (Fragment)	7	15	3	3	0	1	4	0	5	6	2.2	95% (0.038)
P01024	C3	Complement C3 (C3 and PZP-like alpha-2-macroglobulin domain-containing protein 1) [Cleaved into: Complement C3 beta chain; Complement C3 alpha chain; C3a anaphylatoxin; Acylation stimulating protein (ASP) (C3adesArg); Complement C3b alpha' chain; Complement C3c alpha' chain fragment 1; Complement C3dg fragment; Complement C3g fragment; Complement C3d fragment; Complement C3f fragment; Complement C3c alpha' chain fragment 2]	54	104	18	11	11	15	5	14	38	47	2.1	95% (< 0.00010)
Q7L2H7	EIF3M	Eukaryotic translation initiation factor 3 subunit M (eIF3m) (Fetal lung protein B5) (hFL-B5) (PCI domain-containing protein 1)	11	22	0	5	1	5	6	10	4	2	2.1	95% (0.018)
Q9BW92	TARS2	Threonine--tRNA ligase, mitochondrial (EC 6.1.1.3) (Threonyl-tRNA synthetase) (ThrRS) (Threonyl-tRNA synthetase-like 1)	11	22	4	3	2	2	11	1	3	7	2.1	95% (0.018)
Q9BYD6	MRPL1	39S ribosomal protein L1, mitochondrial (L1mt) (MRP-L1)	9	18	5	1	3	0	8	3	3	4	2.1	95% (0.032)
J3KQ32	OLA1	Obg-like ATPase 1	9	18	0	2	2	5	3	6	4	5	2.1	95% (0.032)
Q02338	BDH1	D-beta-hydroxybutyrate dehydrogenase, mitochondrial (BDH) (EC 1.1.1.30) (3-hydroxybutyrate dehydrogenase)	17	33	7	1	0	8	13	12	7	1	2.1	95% (0.0054)
P49792	RANBP2	E3 SUMO-protein ligase RanBP2 (EC 6.3.2.-) (358 kDa nucleoporin) (Nuclear pore complex protein Nup358) (Nucleoporin Nup358) (Ran-binding protein 2) (RanBP2) (p270)	17	33	2	4	4	7	7	6	15	5	2.1	95% (0.0054)
Q8IY17	PNPLA6	Neuropathy target esterase (EC 3.1.1.5) (Patatin-like phospholipase domain-containing protein 6)	8	16	6	0	1	1	4	1	5	6	2.1	95% (0.042)

P52948	NUP98	Nuclear pore complex protein Nup98-Nup96 [Cleaved into: Nuclear pore complex protein Nup98 (98 kDa nucleoporin) (Nucleoporin Nup98) (Nup98); Nuclear pore complex protein Nup96 (96 kDa nucleoporin) (Nucleoporin Nup96) (Nup96)]	15	29	4	2	2	7	4	3	15	7	2.1	95% (0.0091)
P23526	AHCY	Adenosylhomocysteinase (AdoHcyase) (EC 3.3.1.1) (S-adenosyl-L-homocysteine hydrolase)	11	21	0	3	2	8	6	8	4	2	2.0	95% (0.027)
A8K3C3		T-complex protein 1 subunit delta	27	50	6	8	6	7	23	12	11	4	2.0	95% (0.0012)
Q13505	MTX1	Metaxin-1 (Mitochondrial outer membrane import complex protein 1)	10	19	3	0	0	7	6	4	5	4	2.0	95% (0.035)
B4DZ67	NUP107	Nuclear pore complex protein Nup107 (cDNA FLJ58739, highly similar to Nuclear pore complex protein Nup107)	10	19	3	3	1	3	3	2	10	4	2.0	95% (0.035)
Q96TC7	RMDN3	Regulator of microtubule dynamics protein 3 (RMD-3) (hRMD-3) (Cerebral protein 10) (Protein FAM82A2) (Protein FAM82C) (Protein tyrosine phosphatase-interacting protein 51) (TCPTP-interacting protein 51)	10	19	5	1	0	4	0	0	12	7	2.0	95% (0.035)
O15042	U2SURP	U2 snRNP-associated SURP motif-containing protein (140 kDa Ser/Arg-rich domain protein) (U2-associated protein SR140)	18	33	2	5	4	7	8	4	12	9	2.0	95% (0.0085)
F1T0J2	GOLGB1	Golgin subfamily B member 1	17	31	5	5	6	1	13	1	12	5	2.0	95% (0.011)
P0C0L5	C4B	Complement C4-B (Basic complement C4) (C3 and PZP-like alpha-2-macroglobulin domain-containing protein 3) [Cleaved into: Complement C4 beta chain; Complement C4-B alpha chain; C4a anaphylatoxin; C4b-B; C4d-B; Complement C4 gamma chain]	12	22	0	2	2	8	3	0	17	3	2.0	95% (0.029)
P02786	TFRC	Transferrin receptor protein 1 (TR) (TfR) (TfR1) (Trfr) (T9) (p90) (CD antigen CD71) [Cleaved into: Transferrin receptor protein 1, serum form (sTfR)]	117	205	29	34	37	20	59	36	47	65	2.0	95% (< 0.00010)
Q53EW8		Thiosulfate sulfurtransferase variant (Fragment)	30	13	12	8	6	4	7	3	0	3	-2.0	95% (0.019)

Q8NC56	LEMD2	LEM domain-containing protein 2 (hLEM2)	41	18	11	6	5	19	4	6	3	5	-2.0	95% (0.0072)
B7XGC2	HLA-A	MHC class I antigen (Fragment)	52	23	5	11	13	23	3	5	8	7	-2.0	95% (0.0028)
P02042	HBD	Hemoglobin subunit delta (Delta-globin) (Hemoglobin delta chain)	235	106	0	76	82	67	0	0	26	0	-2.0	95% (< 0.00010)
Q14254	FLOT2	Flotillin-2 (Epidermal surface antigen) (ESA) (Membrane component chromosome 17 surface marker 1)	49	21	23	10	8	10	2	9	3	7	-2.0	95% (0.0027)
H6A2E0	HLA-B	MHC class I antigen (Fragment)	38	16	0	9	0	15	0	0	7	0	-2.0	95% (0.0068)
A0S1I7	COX1	Cytochrome c oxidase subunit 1 (EC 1.9.3.1) (Fragment)	20	8	8	4	5	2	2	2	1	3	-2.1	95% (0.037)
P50570	DNM2	Dynamin-2 (EC 3.6.5.5)	20	8	0	1	9	9	5	3	1	0	-2.1	95% (0.037)
Q56VL3	OCIAD2	OCIA domain-containing protein 2 (Ovarian carcinoma immunoreactive antigen-like protein)	27	11	14	7	3	3	2	4	1	4	-2.1	95% (0.018)
Q6FGL0	LGALS3	Galectin (Fragment)	34	14	10	6	3	15	2	5	4	3	-2.1	95% (0.0088)
P16144	ITGB4	Integrin beta-4 (GP150) (CD antigen CD104)	39	16	30	4	4	1	2	0	2	12	-2.1	95% (0.0050)
Q9NUQ9	FAM49B	Protein FAM49B (L1)	23	9	1	11	5	6	0	4	4	1	-2.1	95% (0.023)
C8C504	HBB	Beta-globin	360	151	22	113	124	105	25	35	37	55	-2.1	95% (< 0.00010)
Q92520	FAM3C	Protein FAM3C (Interleukin-like EMT inducer)	50	20	18	10	8	14	4	5	5	6	-2.2	95% (0.0012)
Q9Y394	DHRS7	Dehydrogenase/reductase SDR family member 7 (EC 1.1.-.-) (Retinal short-chain dehydrogenase/reductase 4) (retSDR4)	38	15	18	8	7	5	0	5	3	7	-2.2	95% (0.0041)
Q14566	MCM6	DNA replication licensing factor MCM6 (EC 3.6.4.12) (p105MCM)	19	7	0	9	11	0	5	1	1	0	-2.2	95% (0.030)
Q99653	CHP1	Calcineurin B homologous protein 1 (Calcineurin B-like protein) (Calcium-binding protein CHP) (Calcium-binding protein p22) (EF-hand calcium-binding domain-containing protein p22)	24	9	12	3	4	5	1	3	1	3	-2.2	95% (0.016)
P56385	ATP5I	ATP synthase subunit e, mitochondrial (ATPase subunit e)	17	6	3	2	4	8	0	2	3	1	-2.2	95% (0.033)
Q86WA6	BPHL	Valacyclovir hydrolase (VACVase) (Valacyclovirase) (EC 3.1.-.-) (Biphenyl hydrolase-like protein) (Biphenyl hydrolase-related protein) (Bph-rp) (Breast	17	6	3	1	5	8	0	0	1	5	-2.2	95% (0.033)

		epithelial mucin-associated antigen) (MCNAA)											
Q4AEJ3	hfzo1	Mitochondrial transmembrane GTPase Fzo-1	17	6	7	4	4	2	3	1	1	2	-2.2	95% (0.033)
P02730	SLC4A1	Band 3 anion transport protein (Anion exchange protein 1) (AE 1) (Anion exchanger 1) (Solute carrier family 4 member 1) (CD antigen CD233)	63	24	3	27	26	7	5	2	9	8	-2.3	95% (0.00016)
E7EVP7	ITPR1	Inositol 1,4,5-trisphosphate receptor type 1	25	9	14	4	7	0	1	0	1	7	-2.3	95% (0.012)
E3SWK8	HLA-C	MHC class I antigen (Fragment)	28	10	0	8	0	12	0	0	4	0	-2.3	95% (0.0073)
P27701	CD82	CD82 antigen (C33 antigen) (IA4) (Inducible membrane protein R2) (Metastasis suppressor Kangai-1) (Suppressor of tumorigenicity 6 protein) (Tetraspanin-27) (Tspan-27) (CD antigen CD82)	44	16	13	12	15	4	5	1	5	5	-2.3	95% (0.00097)
Q16555	DPYSL2	Dihydropyrimidinase-related protein 2 (DRP-2) (Collapsin response mediator protein 2) (CRMP-2) (N2A3) (Unc-33-like phosphoprotein 2) (ULIP-2)	21	7	2	11	8	0	0	2	2	0	-2.4	95% (0.014)
P29728	OAS2	2'-5'-oligoadenylate synthase 2 ((2-5')oligo(A) synthase 2) (2-5A synthase 2) (EC 2.7.7.84) (p69 OAS / p71 OAS) (p69OAS / p71OAS)	21	7	0	2	2	17	0	7	0	0	-2.4	95% (0.014)
Q96IU4	ABHD14B	Alpha/beta hydrolase domain-containing protein 14B (Abhydrolase domain-containing protein 14B) (EC 3.-.-.-) (CCG1-interacting factor B)	13	4	2	3	3	5	0	3	1	0	-2.4	95% (0.042)
O14657	TOR1B	Torsin-1B (Torsin family 1 member B)	13	4	0	2	2	9	0	1	0	3	-2.4	95% (0.042)
Q96CW1	AP2M1	AP-2 complex subunit mu (AP-2 mu chain) (Adapter-related protein complex 2 subunit mu) (Adaptin-mu2) (Adaptor protein complex AP-2 subunit mu) (Clathrin assembly protein complex 2 mu medium chain) (Clathrin coat assembly protein AP50) (Clathrin coat-associated protein AP50) (HA2 50 kDa subunit) (Plasma membrane adaptor AP-2 50 kDa protein)	32	11	10	9	5	9	3	1	3	4	-2.4	95% (0.0032)
O95294	RASAL1	RasGAP-activating-like protein 1	19	6	19	0	0	0	0	0	0	6	-2.5	95% (0.016)

B4DGM9	TOR1A	Torsin family 1, member A (Torsin A), isoform CRA_a (cDNA FLJ56343, highly similar to Torsin A)	33	11	8	8	4	14	0	4	3	4	-2.5	95% (0.0022)
P21589	NT5E	5'-nucleotidase (5'-NT) (EC 3.1.3.5) (Ecto-5'-nucleotidase) (CD antigen CD73)	22	7	7	9	7	0	0	3	2	2	-2.5	95% (0.0098)
P05109	S100A8	Protein S100-A8 (Calgranulin-A) (Calprotectin L1L subunit) (Cystic fibrosis antigen) (CFAG) (Leukocyte L1 complex light chain) (Migration inhibitory factor-related protein 8) (MRP-8) (p8) (S100 calcium-binding protein A8) (Urinary stone protein band A) [Cleaved into: Protein S100-A8, N-terminally processed]	53	18	1	22	20	10	0	5	5	8	-2.5	95% (0.00014)
Q14573	ITPR3	Inositol 1,4,5-trisphosphate receptor type 3 (IP3 receptor isoform 3) (IP3R 3) (InsP3R3) (Type 3 inositol 1,4,5-trisphosphate receptor) (Type 3 InsP3 receptor)	138	48	85	21	29	5	6	0	32	10	-2.5	95% (< 0.00010)
Q9Y2A7	NCKAP1	Nck-associated protein 1 (NAP 1) (Membrane-associated protein HEM-2) (p125Nap1)	28	9	11	5	6	5	2	3	1	2	-2.5	95% (0.0038)
P39687	ANP32A	Acidic leucine-rich nuclear phosphoprotein 32 family member A (Acidic nuclear phosphoprotein pp32) (Leucine-rich acidic nuclear protein) (LANP) (Mapmodulin) (Potent heat-stable protein phosphatase 2A inhibitor I1PP2A) (Putative HLA-DR-associated protein I) (PHAPI)	11	3	1	3	1	7	1	0	2	0	-2.6	95% (0.046)
Q96BM9	ARL8A	ADP-ribosylation factor-like protein 8A (ADP-ribosylation factor-like protein 10B) (Novel small G protein indispensable for equal chromosome segregation 2)	40	13	14	10	8	8	0	4	3	6	-2.6	95% (0.00063)
Q9UKV8	AGO2	Protein argonaute-2 (Argonaute2) (hAgo2) (EC 3.1.26.n2) (Argonaute RISC catalytic component 2) (Eukaryotic translation initiation factor 2C 2) (eIF-2C 2) (eIF2C 2) (PAZ Piwi domain protein) (PPD) (Protein slicer)	14	4	0	6	8	1	1	0	2	1	-2.6	95% (0.028)
Q9H4M9	EHD1	EH domain-containing protein 1 (PAST homolog 1) (hPAST1) (Testilin)	14	4	5	4	1	4	0	2	2	0	-2.6	95% (0.028)

Q13868	EXOSC2	Exosome complex component RRP4 (Exosome component 2) (Ribosomal RNA-processing protein 4)	14	4	0	4	4	6	1	1	2	0	-2.6	95% (0.028)
P00390	GSR	Glutathione reductase, mitochondrial (GR) (GRase) (EC 1.8.1.7)	17	5	2	7	7	1	1	0	2	2	-2.6	95% (0.017)
Q0PNF2		FEX1	17	5	0	7	9	0	0	0	1	4	-2.6	95% (0.017)
O15400	STX7	Syntaxin-7	20	6	9	4	4	3	0	0	2	4	-2.6	95% (0.011)
P37235	HPCAL1	Hippocalcin-like protein 1 (Calcium-binding protein BDR-1) (HLP2) (Visinin-like protein 3) (VILIP-3)	23	7	5	7	9	2	0	1	3	3	-2.6	95% (0.0066)
P09429	HMGB1	High mobility group protein B1 (High mobility group protein 1) (HMG-1)	29	9	8	5	5	11	2	1	1	5	-2.6	95% (0.0026)
P17301	ITGA2	Integrin alpha-2 (CD49 antigen-like family member B) (Collagen receptor) (Platelet membrane glycoprotein Ia) (GPIa) (VLA-2 subunit alpha) (CD antigen CD49b)	24	7	10	7	7	0	0	0	0	7	-2.7	95% (0.0045)
Q9UQB8	BAIAP2	Brain-specific angiogenesis inhibitor 1-associated protein 2 (BAI-associated protein 2) (BAI1-associated protein 2) (Protein BAP2) (Fas ligand-associated factor 3) (FLAF3) (Insulin receptor substrate p53/p58) (IRS-58) (IRSp53/58) (Insulin receptor substrate protein of 53 kDa) (IRSp53) (Insulin receptor substrate p53)	21	6	8	4	4	5	1	0	3	2	-2.7	95% (0.0071)
Q9H1E5	TMX4	Thioredoxin-related transmembrane protein 4 (Thioredoxin domain-containing protein 13)	21	6	8	3	5	5	0	1	2	3	-2.7	95% (0.0071)
Q9BUP0	EFHD1	EF-hand domain-containing protein D1 (EF-hand domain-containing protein 1) (Swiprosin-2)	15	4	0	6	7	2	0	2	1	1	-2.8	95% (0.018)
J3QKR5	CDK11B	Cyclin-dependent kinase 11B	12	3	0	3	4	5	1	0	1	1	-2.8	95% (0.030)
P36915	GNL1	Guanine nucleotide-binding protein-like 1 (GTP-binding protein HSR1)	12	3	0	1	2	9	1	1	1	0	-2.8	95% (0.030)
O60664	PLIN3	Perilipin-3 (47 kDa mannose 6-phosphate receptor-binding protein) (47 kDa MPR-binding protein) (Cargo selection protein TIP47) (Mannose-6-phosphate receptor-binding protein 1) (Placental protein 17) (PP17)	12	3	8	1	1	2	0	0	0	3	-2.8	95% (0.030)

Q6PL18	ATAD2	ATPase family AAA domain-containing protein 2 (EC 3.6.1.3) (AAA nuclear coregulator cancer-associated protein) (ANCCA)	9	2	0	4	5	0	0	0	2	0	-2.8	95% (0.050)
Q96ER9	CCDC51	Coiled-coil domain-containing protein 51	9	2	1	0	0	8	0	0	0	2	-2.8	95% (0.050)
Q92522	H1FX	Histone H1x	9	2	0	2	3	4	0	0	0	2	-2.8	95% (0.050)
B4DG22	RPS6KA3	Ribosomal protein S6 kinase alpha-3 (cDNA FLJ56618, highly similar to Ribosomal protein S6 kinase alpha-3 (EC 2.7.11.1))	9	2	0	4	4	1	1	1	0	0	-2.8	95% (0.050)
Q86WV6	TMEM173	Stimulator of interferon genes protein (hSTING) (Endoplasmic reticulum interferon stimulator) (ERIS) (Mediator of IRF3 activation) (hMITA) (Transmembrane protein 173)	25	7	8	5	1	11	0	2	2	3	-2.8	95% (0.0030)
O94905	ERLIN2	Erlin-2 (Endoplasmic reticulum lipid raft-associated protein 2) (Stomatin-prohibitin-flotillin-HflC/K domain-containing protein 2) (SPFH domain-containing protein 2)	61	18	19	13	14	15	1	5	4	8	-2.9	95% (< 0.00010)
Q3SXM5	HSDL1	Inactive hydroxysteroid dehydrogenase-like protein 1	16	4	9	2	4	1	1	0	1	2	-2.9	95% (0.012)
Q14108	SCARB2	Lysosome membrane protein 2 (85 kDa lysosomal membrane sialoglycoprotein) (LGP85) (CD36 antigen-like 2) (Lysosome membrane protein II) (LIMP II) (Scavenger receptor class B member 2) (CD antigen CD36)	26	7	9	7	6	4	0	1	4	3	-3.0	95% (0.0020)
P12109	COL6A1	Collagen alpha-1(VI) chain	13	3	1	7	5	0	0	0	0	3	-3.0	95% (0.019)
H3BN55	RAB27A	Ras-related protein Rab-27A (Fragment)	13	3	10	0	2	1	0	0	2	1	-3.0	95% (0.019)
P58107	EPPK1	Epiplakin (450 kDa epidermal antigen)	134	39	53	21	31	31	22	3	12	2	-3.0	95% (< 0.00010)
Q9Y276	BCS1L	Mitochondrial chaperone BCS1 (h-BCS1) (BCS1-like protein)	10	2	3	3	2	2	0	2	0	0	-3.1	95% (0.031)
B7ZKL3	EPS8L2	EPS8L2 protein (Epidermal growth factor receptor kinase substrate 8-like protein 2)	10	2	6	1	2	1	2	0	0	0	-3.1	95% (0.031)
B4DKK2	SRI	Sorcin (cDNA FLJ60640, highly similar to Sorcin)	10	2	3	3	1	3	0	2	0	0	-3.1	95% (0.031)
P12532	CKMT1A	Creatine kinase U-type, mitochondrial (EC 2.7.3.2) (Acidic-type mitochondrial	31	8	13	5	3	11	2	0	6	0	-3.1	95% (0.00055)

		creatine kinase) (Mia-CK) (Ubiquitous mitochondrial creatine kinase) (U-MtCK)											
O75911	DHRS3	Short-chain dehydrogenase/reductase 3 (EC 1.1.1.300) (DD83.1) (Retinal short-chain dehydrogenase/reductase 1) (retSDR1)	21	5	12	2	7	0	0	1	3	1	-3.2	95% (0.0031)
H3BPE1	MACF1	Microtubule-actin cross-linking factor 1, isoforms 1/2/3/5	43	11	9	11	19	5	4	0	7	0	-3.2	95% (< 0.00010)
Q14019	COTL1	Coactosin-like protein	7	1	0	2	5	0	0	0	1	0	-3.3	95% (0.050)
Q7KZN9	COX15	Cytochrome c oxidase assembly protein COX15 homolog	7	1	3	0	0	4	0	1	0	0	-3.3	95% (0.050)
P14854	COX6B1	Cytochrome c oxidase subunit 6B1 (Cytochrome c oxidase subunit VIb isoform 1) (COX VIb-1)	7	1	1	1	2	3	1	0	0	0	-3.3	95% (0.050)
B1AUU8	EPS15	Epidermal growth factor receptor substrate 15	7	1	1	4	2	0	1	0	0	0	-3.3	95% (0.050)
Q6ZRV2	FAM83H	Protein FAM83H	7	1	5	0	0	2	1	0	0	0	-3.3	95% (0.050)
P26012	ITGB8	Integrin beta-8	7	1	3	1	1	2	0	0	1	0	-3.3	95% (0.050)
G3V2F7	TMEM189	HCG2044781 (Protein TMEM189-UBE2V1) (Transmembrane protein 189)	7	1	1	1	3	2	1	0	0	0	-3.3	95% (0.050)
D3DQ48	TMEM59	Transmembrane protein 59, isoform CRA_e	7	1	0	4	3	0	1	0	0	0	-3.3	95% (0.050)
Q9H269	VPS16	Vacuolar protein sorting-associated protein 16 homolog (hVPS16)	7	1	2	2	1	2	0	1	0	0	-3.3	95% (0.050)
Q6YHK3	CD109	CD109 antigen (150 kDa TGF-beta-1-binding protein) (C3 and PZP-like alpha-2-macroglobulin domain-containing protein 7) (Platelet-specific Gov antigen) (p180) (r150) (CD antigen CD109)	19	4	6	7	6	0	2	0	2	0	-3.4	95% (0.0031)
O76027	ANXA9	Annexin A9 (Annexin XXXI) (Annexin-31) (Annexin-9) (Pemphaxin)	23	5	13	0	1	10	5	0	0	0	-3.5	95% (0.0013)
P55011	SLC12A2	Solute carrier family 12 member 2 (Basolateral Na-K-Cl symporter) (Bumetanide-sensitive sodium-(potassium)-chloride cotransporter 1)	31	7	1	14	17	0	5	0	1	1	-3.5	95% (0.00023)
P27695	APEX1	DNA-(apurinic or apyrimidinic site) lyase (EC 3.1.-.-) (EC 4.2.99.18) (APEX nuclease) (APEN) (Apurinic-apyrimidinic endonuclease 1) (AP endonuclease 1) (APE-1) (REF-1) (Redox factor-1)	16	3	0	1	1	14	1	1	1	0	-3.6	95% (0.0047)

		[Cleaved into: DNA-(apurinic or apyrimidinic site) lyase, mitochondrial]												
A4D2Q0	UNC84A	SUN domain-containing protein 1 (Unc-84 homolog A (C. elegans))	16	3	13	2	0	2	1	0	0	2	-3.6	95% (0.0047)
Q7L5L3	GDPD3	Glycerophosphodiester phosphodiesterase domain-containing protein 3 (EC 3.1.-.-)	12	2	1	1	0	10	1	0	1	0	-3.6	95% (0.012)
Q9H3Z4	DNAJC5	DnaJ homolog subfamily C member 5 (Cysteine string protein) (CSP)	8	1	2	2	2	2	0	0	0	1	-3.7	95% (0.029)
H0Y6T6	NRM	Nurim (Fragment)	8	1	2	0	0	6	0	0	0	1	-3.7	95% (0.029)
F8J390	PKP3	Plakophilin 3b (Fragment)	8	1	4	0	2	2	1	0	0	0	-3.7	95% (0.029)
P49903	SEPHS1	Selenide, water dikinase 1 (EC 2.7.9.3) (Selenium donor protein 1) (Selenophosphate synthase 1)	8	1	0	3	1	4	1	0	0	0	-3.7	95% (0.029)
P37837	TALDO1	Transaldolase (EC 2.2.1.2)	21	4	2	2	9	8	1	1	1	1	-3.8	95% (0.0012)
Q5HYL6	DKFZp686E1899	Putative uncharacterized protein DKFZp686E1899	13	2	1	1	7	4	0	0	2	0	-3.9	95% (0.0070)
Q59FB9		Toll interacting protein variant (Fragment)	13	2	6	4	2	2	0	0	1	1	-3.9	95% (0.0070)
F8VXY3	OAS1	2'-5'-oligoadenylate synthase 1	18	3	0	0	0	18	0	3	0	0	-4.0	95% (0.0018)
E5KSE7		Mitochondrial short-chain specific acyl-CoA dehydrogenase	18	3	5	5	3	5	0	1	0	1	-4.0	95% (0.0018)
P33897	ABCD1	ATP-binding cassette sub-family D member 1 (Adrenoleukodystrophy protein) (ALDP)	9	1	4	1	0	4	0	0	1	0	-4.1	95% (0.017)
Q9NTX5	ECHDC1	Ethylmalonyl-CoA decarboxylase (EC 4.1.1.94) (Enoyl-CoA hydratase domain-containing protein 1) (Methylmalonyl-CoA decarboxylase) (MMCD) (EC 4.1.1.41)	9	1	3	2	1	3	0	0	1	0	-4.1	95% (0.017)
O43920	NDUFS5	NADH dehydrogenase [ubiquinone] iron-sulfur protein 5 (Complex I-15 kDa) (CI-15 kDa) (NADH-ubiquinone oxidoreductase 15 kDa subunit)	9	1	3	2	2	2	0	0	1	0	-4.1	95% (0.017)
Q00796	SORD	Sorbitol dehydrogenase (EC 1.1.1.14) (L-iditol 2-dehydrogenase)	9	1	0	0	0	9	0	0	1	0	-4.1	95% (0.017)
Q15149	PLEC	Plectin (PCN) (PLTN) (Hemidesmosomal protein 1) (HD1) (Plectin-1)	1115	246	418	276	278	146	34	22	108	83	-4.1	95% (< 0.00010)
P16157	ANK1	Ankyrin-1 (ANK-1) (Ankyrin-R) (Erythrocyte ankyrin)	28	5	0	12	16	0	0	0	5	0	-4.2	95% (0.00012)
Q99828	CIB1	Calcium and integrin-binding protein 1 (CIB) (Calcium- and integrin-binding	14	2	4	2	0	8	0	0	0	2	-4.2	95% (0.0042)

		protein) (CIBP) (Calmyrin) (DNA-PKcs-interacting protein) (Kinase-interacting protein) (KIP) (SNK-interacting protein 2-28) (SIP2-28)											
P05187	ALPP	Alkaline phosphatase, placental type (EC 3.1.3.1) (Alkaline phosphatase Regan isozyme) (Placental alkaline phosphatase 1) (PLAP-1)	24	4	14	5	5	0	0	0	0	4	-4.3	95% (0.00028)
Q16864	ATP6V1F	V-type proton ATPase subunit F (V-ATPase subunit F) (V-ATPase 14 kDa subunit) (Vacuolar proton pump subunit F)	5	0	1	1	1	2	0	0	0	0	-4.5	95% (0.042)
Q9Y3E8	C17orf25	CGI-150 protein	5	0	0	2	1	2	0	0	0	0	-4.5	95% (0.042)
Q96N67	DOCK7	Dedicator of cytokinesis protein 7	5	0	1	0	3	1	0	0	0	0	-4.5	95% (0.042)
P11678	EPX	Eosinophil peroxidase (EPO) (EC 1.11.1.7) [Cleaved into: Eosinophil peroxidase light chain; Eosinophil peroxidase heavy chain]	5	0	2	0	0	3	0	0	0	0	-4.5	95% (0.042)
Q9NPD3	EXOSC4	Exosome complex component RRP41 (Exosome component 4) (Ribosomal RNA-processing protein 41) (p12A)	5	0	0	3	0	2	0	0	0	0	-4.5	95% (0.042)
Q96J02	ITCH	E3 ubiquitin-protein ligase Itchy homolog (Itch) (EC 6.3.2.-) (Atrophin-1-interacting protein 4) (AIP4) (NFE2-associated polypeptide 1) (NAPP1)	5	0	1	1	3	0	0	0	0	0	-4.5	95% (0.042)
B2ZGL7	MGLL	Monoacylglycerol lipase isoform 2	5	0	4	1	0	0	0	0	0	0	-4.5	95% (0.042)
Q9BXB4	OSBPL11	Oxysterol-binding protein-related protein 11 (ORP-11) (OSBP-related protein 11)	5	0	1	1	2	1	0	0	0	0	-4.5	95% (0.042)
P41247	PNPLA4	Patatin-like phospholipase domain-containing protein 4 (EC 3.1.1.3) (Protein GS2)	5	0	4	0	1	0	0	0	0	0	-4.5	95% (0.042)
O75298	RTN2	Reticulon-2 (Neuroendocrine-specific protein-like 1) (NSP-like protein 1) (Neuroendocrine-specific protein-like I) (NSP-like protein I) (NSPLI)	5	0	3	0	0	2	0	0	0	0	-4.5	95% (0.042)
Q14BN4	SLMAP	Sarcolemmal membrane-associated protein (Sarcolemmal-associated protein)	5	0	5	0	0	0	0	0	0	0	-4.5	95% (0.042)
Q5W111	SPRYD7	SPRY domain-containing protein 7 (Chronic lymphocytic leukemia deletion region gene 6 protein) (CLL deletion region gene 6 protein)	5	0	4	1	0	0	0	0	0	0	-4.5	95% (0.042)
C9K0W8	STX16	Syntaxin-16 (Uncharacterized protein)	5	0	3	1	1	0	0	0	0	0	-4.5	95% (0.042)

Q9C0C2	TNKS1BP1	182 kDa tankyrase-1-binding protein	5	0	2	1	2	0	0	0	0	0	-4.5	95% (0.042)
Q53GD8		Peptidyl-prolyl cis-trans isomerase (Fragment)	5	0	0	1	0	4	0	0	0	0	-4.5	95% (0.042)
O00194	RAB27B	Ras-related protein Rab-27B (C25KG)	10	1	7	1	2	0	0	0	1	0	-4.5	95% (0.0098)
P05164	MPO	Myeloperoxidase (MPO) (EC 1.11.2.2) [Cleaved into: Myeloperoxidase; 89 kDa myeloperoxidase; 84 kDa myeloperoxidase; Myeloperoxidase light chain; Myeloperoxidase heavy chain]	25	4	12	0	0	13	0	1	3	0	-4.5	95% (0.00017)
B2RMN7	SPTB	Spectrin, beta, erythrocytic	16	2	0	6	10	0	1	0	0	0	-4.7	95% (0.0015)
P43155	CRAT	Carnitine O-acetyltransferase (Carnitine acetylase) (EC 2.3.1.7) (Carnitine acetyltransferase) (CAT) (CrAT)	11	1	1	1	5	4	0	1	0	0	-4.9	95% (0.0056)
Q8TE68	EPS8L1	Epidermal growth factor receptor kinase substrate 8-like protein 1 (EPS8-like protein 1) (Epidermal growth factor receptor pathway substrate 8-related protein 1) (EPS8-related protein 1)	11	1	11	0	0	1	0	0	0	2	-4.9	95% (0.0056)
P08582	MFI2	Melanotransferrin (Melanoma-associated antigen p97) (CD antigen CD228)	11	1	6	3	2	0	0	0	1	0	-4.9	95% (0.0056)
A8K8G0	HDGF	Hepatoma-derived growth factor (cDNA FLJ75113)	29	4	4	6	8	11	0	2	0	2	-5.1	95% (< 0.00010)
Q13363	CTBP1	C-terminal-binding protein 1 (CtBP1) (EC 1.1.1.-)	6	0	0	1	3	2	0	0	0	0	-5.2	95% (0.022)
E7ET76	GGT1	Gamma-glutamyltranspeptidase 1 light chain (Fragment)	6	0	3	1	2	0	0	0	0	0	-5.2	95% (0.022)
Q5T601	GPR110	Probable G-protein coupled receptor 110 (G-protein coupled receptor KPG_012) (G-protein coupled receptor PGR19)	6	0	4	0	0	2	0	0	0	0	-5.2	95% (0.022)
B7ZKT9	KIAA1033	KIAA1033 protein	6	0	5	0	0	1	0	0	0	0	-5.2	95% (0.022)
A2BDK6	MAP1B	Microtubule-associated protein 1B (Microtubule-associated protein 1B, isoform CRA_a)	6	0	2	1	3	0	0	0	0	0	-5.2	95% (0.022)
C9JS50	STEAP4	Metalloreductase STEAP4 (Uncharacterized protein)	6	0	0	2	1	3	0	0	0	0	-5.2	95% (0.022)
E5KNQ5	TK2	Mitochondrial thymidine kinase 2 (Thymidine kinase 2, mitochondrial) (Thymidine kinase 2, mitochondrial, isoform CRA_e)	6	0	2	2	1	1	0	0	0	0	-5.2	95% (0.022)

Q13753	LAMC2	Laminin subunit gamma-2 (Cell-scattering factor 140 kDa subunit) (CSF 140 kDa subunit) (Epiligrin subunit gamma) (Kalinin subunit gamma) (Kalinin/nicein/epiligrin 100 kDa subunit) (Ladsin 140 kDa subunit) (Laminin B2t chain) (Laminin-5 subunit gamma) (Large adhesive scatter factor 140 kDa subunit) (Nicein subunit gamma)	30	4	4	12	14	0	0	0	0	3	-5.3	95% (< 0.00010)
P26583	HMGB2	High mobility group protein B2 (High mobility group protein 2) (HMG-2)	13	1	0	1	3	7	1	0	0	0	-5.7	95% (0.0018)
P09913	IFIT2	Interferon-induced protein with tetratricopeptide repeats 2 (IFIT-2) (ISG-54 K) (Interferon-induced 54 kDa protein) (IFI-54K) (P54)	13	1	0	0	0	13	0	1	0	0	-5.7	95% (0.0018)
O75110	ATP9A	Probable phospholipid-transporting ATPase IIA (EC 3.6.3.1) (ATPase class II type 9A)	7	0	7	0	0	0	0	0	0	0	-5.9	95% (0.012)
Q96LJ7	DHRS1	Dehydrogenase/reductase SDR family member 1 (EC 1.1.-.-)	7	0	3	0	0	4	0	0	0	0	-5.9	95% (0.012)
Q8IWB1	ITPRIP	Inositol 1,4,5-trisphosphate receptor-interacting protein (Protein DANGER)	7	0	6	0	0	1	0	0	0	0	-5.9	95% (0.012)
E9PDY6	MUC4	Mucin-4 beta chain	7	0	6	1	0	0	0	0	0	0	-5.9	95% (0.012)
Q9NQX4	MYO5C	Unconventional myosin-Vc	7	0	7	0	0	0	0	0	0	0	-5.9	95% (0.012)
Q7L523	RRAGA	Ras-related GTP-binding protein A (Rag A) (RagA) (Adenovirus E3 14.7 kDa-interacting protein 1) (FIP-1)	7	0	1	1	3	2	0	0	0	0	-5.9	95% (0.012)
K7EKI8	PPL	Periplakin	187	25	98	25	18	42	11	0	7	7	-6.4	95% (< 0.00010)
Q5VYY1	ANKRD22	Ankyrin repeat domain-containing protein 22	8	0	2	0	1	5	0	0	0	0	-6.6	95% (0.0062)
Q9NUI1	DECR2	Peroxisomal 2,4-dienoyl-CoA reductase (pDCR) (EC 1.3.1.34) (2,4-dienoyl-CoA reductase 2)	8	0	4	0	0	4	0	0	0	0	-6.6	95% (0.0062)
Q9UHL4	DPP7	Dipeptidyl peptidase 2 (EC 3.4.14.2) (Dipeptidyl aminopeptidase II) (Dipeptidyl peptidase 7) (Dipeptidyl peptidase II) (DPP II) (Quiescent cell proline dipeptidase)	8	0	3	3	0	2	0	0	0	0	-6.6	95% (0.0062)
Q8WWP7	GIMAP1	GTPase IMAP family member 1 (Immunity-associated protein 1) (hIMAP1)	8	0	0	2	2	3	0	0	0	0	-6.6	95% (0.0062)

E7ET40	PLAU	Urokinase-type plasminogen activator chain B	8	0	8	0	0	0	0	0	0	0	-6.6	95% (0.0062)
G3V207	TMCC3	Transmembrane and coiled-coil domain family 3, isoform CRA_c (Transmembrane and coiled-coil domains protein 3)	8	0	8	0	0	0	0	0	0	0	-6.6	95% (0.0062)
Q8IY95	TMEM192	Transmembrane protein 192	8	0	4	3	1	0	0	0	0	0	-6.6	95% (0.0062)
Q8TD43	TRPM4	Transient receptor potential cation channel subfamily M member 4 (hTRPM4) (Calcium-activated non-selective cation channel 1) (Long transient receptor potential channel 4) (LTrpC-4) (LTrpC4) (Melastatin-4)	8	0	8	0	0	0	0	0	0	0	-6.6	95% (0.0062)
B2RC75		Transmembrane channel-like protein	8	0	3	0	0	5	0	0	0	0	-6.6	95% (0.0062)
B4DKB2	ECE1	Endothelin-converting enzyme 1 (cDNA FLJ59212, highly similar to Endothelin-converting enzyme 1 (EC 3.4.24.71))	38	4	11	13	15	0	3	0	1	0	-6.7	95% (< 0.00010)
Q03013	GSTM4	Glutathione S-transferase Mu 4 (EC 2.5.1.18) (GST class-mu 4) (GST-Mu2) (GSTM4-4)	9	0	0	2	0	7	0	0	0	0	-7.3	95% (0.0033)
P61601	NCALD	Neurocalcin-delta	9	0	4	0	0	2	0	0	0	0	-7.3	95% (0.0033)
Q08AI8	C2orf54	Uncharacterized protein C2orf54	10	0	3	0	0	7	0	0	0	0	-8.0	95% (0.0017)
E9PSH2	CD55	Complement decay-accelerating factor	10	0	2	4	4	0	0	0	0	0	-8.0	95% (0.0017)
P01859	IGHG2	Ig gamma-2 chain C region	10	0	0	0	0	6	0	0	0	0	-8.0	95% (0.0017)
Q8NI35	INADL	InaD-like protein (Inadl protein) (hINADL) (Pals1-associated tight junction protein) (Protein associated to tight junctions)	10	0	9	0	0	1	0	0	0	0	-8.0	95% (0.0017)
J3QRN6	MYO1D	Unconventional myosin-Id	10	0	10	0	0	0	0	0	0	0	-8.0	95% (0.0017)
P13727	PRG2	Bone marrow proteoglycan (BMPG) (Proteoglycan 2) [Cleaved into: Eosinophil granule major basic protein (EMBP) (MBP) (Pregnancy-associated major basic protein)]	10	0	4	0	0	6	0	0	0	0	-8.0	95% (0.0017)
B4DZS8		Peptidyl-prolyl cis-trans isomerase (Fragment)	10	0	0	4	6	0	0	0	0	0	-8.0	95% (0.0017)
Q16647	PTGIS	Prostacyclin synthase (EC 5.3.99.4) (Prostaglandin I2 synthase)	40	3	35	0	0	5	0	0	0	3	-8.7	95% (< 0.00010)
F6M9T7	IDO1	Indolamine 2,3 dioxygenase (EC 1.13.11.52)	11	0	0	6	5	0	0	0	0	0	-8.8	95% (0.00091)

Q9Y2J8	PADI2	Protein-arginine deiminase type-2 (EC 3.5.3.15) (PAD-H19) (Peptidylarginine deiminase II) (Protein-arginine deiminase type II)	11	0	0	5	6	0	0	0	0	0	-8.8	95% (0.00091)
P35354	PTGS2	Prostaglandin G/H synthase 2 (EC 1.14.99.1) (Cyclooxygenase-2) (COX-2) (PHS II) (Prostaglandin H2 synthase 2) (PGH synthase 2) (PGHS-2) (Prostaglandin-endoperoxide synthase 2)	11	0	2	6	3	0	0	0	0	0	-8.8	95% (0.00091)
O95758	PTBP3	Polypyrimidine tract-binding protein 3 (Regulator of differentiation 1) (Rod1)	12	0	0	4	0	3	0	0	0	0	-9.5	95% (0.00048)
A8K7T0		Kynureninase (EC 3.7.1.3)	13	0	0	6	8	0	0	0	0	0	-10.2	95% (0.00026)
Q9H5V8	CDCP1	CUB domain-containing protein 1 (Membrane glycoprotein gp140) (Subtractive immunization M plus HEp3-associated 135 kDa protein) (SIMA135) (Transmembrane and associated with src kinases) (CD antigen CD318)	14	0	1	6	7	0	0	0	0	0	-10.9	95% (0.00014)
P00488	F13A1	Coagulation factor XIII A chain (Coagulation factor XIIIa) (EC 2.3.2.13) (Protein-glutamine gamma-glutamyltransferase A chain) (Transglutaminase A chain)	14	0	0	4	10	0	0	0	0	0	-10.9	95% (0.00014)
O60449	LY75	Lymphocyte antigen 75 (Ly-75) (C-type lectin domain family 13 member B) (DEC-205) (gp200-MR6) (CD antigen CD205)	14	0	1	4	9	0	0	0	0	0	-10.9	95% (0.00014)
Q9ULV0	MYO5B	Unconventional myosin-Vb	14	0	12	2	0	0	0	0	0	0	-10.9	95% (0.00014)
Q8IVF2	AHNAK2	Protein AHNAK2	15	0	15	0	0	0	0	0	0	0	-11.6	95% (< 0.00010)
P02549	SPTA1	Spectrin alpha chain, erythrocytic 1 (Erythroid alpha-spectrin)	16	0	0	5	11	0	0	0	0	0	-12.3	95% (< 0.00010)
B7Z416	DHRS9	Dehydrogenase/reductase SDR family member 9 (cDNA FLJ55219, highly similar to Dehydrogenase/reductase SDR family member 9 (EC 1.1.-.-))	46	2	18	18	18	18	0	0	0	2	-13.0	95% (< 0.00010)
B0QZ43	ERLIN1	Erlin-1 (Fragment)	19	0	7	4	3	0	0	0	0	0	-14.5	95% (< 0.00010)

H0Y3C5	HCK	Tyrosine-protein kinase HCK	19	0	2	2	2	2	0	0	0	0	-14.5	95% (< 0.00010)
P20592	MX2	Interferon-induced GTP-binding protein Mx2 (Interferon-regulated resistance GTP-binding protein MxB) (Myxovirus resistance protein 2) (p78-related protein)	28	0	0	0	3	22	0	0	0	0	-20.9	95% (< 0.00010)
I6NXG5	HLA-C	MHC class I antigen (Fragment)	42	0	0	11	7	16	0	0	0	0	-30.9	95% (< 0.00010)
B7ZLH8	EVPL	EVPL protein	52	0	33	2	0	16	0	0	0	0	-38.1	95% (< 0.00010)
Q860I7	HLA-B	MHC class I antigen	75	0	0	20	0	25	0	0	0	0	-54.5	95% (< 0.00010)

[A]Obtained from UniProt (http://www.uniprot.org/) databases
[C]Spectral counts (SpC) in CN datasets (combined)
[D]Spectral counts (SpC) in CR datasets (combined)
[E]Spectral count ratio (Rsc) between CR (combined) and CN (combined)
[F]Statistical significance (p-value) (Fisher's Exact Test)

Table S4. Proteins identified as unique to CN samples.

Uniprot Acc[A]	Gene Symbol[A]	Protein Description[A]	CN_Combined[C]	CR_Combined[D]	CN_1	CN_2	CN_3	CN_4	CR_1	CR_2	CR_3	CR_4	RSC (CR / CN)[E]	p value[F]
Q860I7	HLA-B	MHC class I antigen	75	0	0	20	0	25	0	0	0	0	-54.5	95% (< 0.00010)
B7ZLH8	EVPL	EVPL protein	52	0	33	2	0	16	0	0	0	0	-38.1	95% (< 0.00010)
I6NXG5	HLA-C	MHC class I antigen (Fragment)	42	0	0	11	7	16	0	0	0	0	-30.9	95% (< 0.00010)
P20592	MX2	Interferon-induced GTP-binding protein Mx2 (Interferon-regulated resistance GTP-binding protein MxB) (Myxovirus resistance protein 2) (p78-related protein)	28	0	0	0	3	22	0	0	0	0	-20.9	95% (< 0.00010)
B0QZ43	ERLIN1	Erlin-1 (Fragment)	19	0	7	4	3	0	0	0	0	0	-14.5	95% (< 0.00010)
H0Y3C5	HCK	Tyrosine-protein kinase HCK	19	0	2	2	2	2	0	0	0	0	-14.5	95% (< 0.00010)
P02549	SPTA1	Spectrin alpha chain, erythrocytic 1 (Erythroid alpha-spectrin)	16	0	0	5	11	0	0	0	0	0	-12.3	95% (< 0.00010)
Q8IVF2	AHNAK2	Protein AHNAK2	15	0	15	0	0	0	0	0	0	0	-11.6	95% (< 0.00010)
Q9H5V8	CDCP1	CUB domain-containing protein 1 (Membrane glycoprotein gp140) (Subtractive immunization M plus HEp3-associated 135 kDa protein) (SIMA135) (Transmembrane and associated with src kinases) (CD antigen CD318)	14	0	1	6	7	0	0	0	0	0	-10.9	95% (0.00014)

P00488	F13A1	Coagulation factor XIII A chain (Coagulation factor XIIIa) (EC 2.3.2.13) (Protein-glutamine gamma-glutamyltransferase A chain) (Transglutaminase A chain)	14	0	0	4	10	0	0	0	0	0	-10.9	95% (0.00014)
O60449	LY75	Lymphocyte antigen 75 (Ly-75) (C-type lectin domain family 13 member B) (DEC-205) (gp200-MR6) (CD antigen CD205)	14	0	1	4	9	0	0	0	0	0	-10.9	95% (0.00014)
Q9ULV0	MYO5B	Unconventional myosin-Vb	14	0	12	2	0	0	0	0	0	0	-10.9	95% (0.00014)
A8K7T0		Kynureninase (EC 3.7.1.3)	13	0	0	6	8	0	0	0	0	0	-10.2	95% (0.00026)
O95758	PTBP3	Polypyrimidine tract-binding protein 3 (Regulator of differentiation 1) (Rod1)	12	0	0	4	0	3	0	0	0	0	-9.5	95% (0.00048)
F6M9T7	IDO1	Indolamine 2,3 dioxygenase (EC 1.13.11.52)	11	0	0	6	5	0	0	0	0	0	-8.8	95% (0.00091)
Q9Y2J8	PADI2	Protein-arginine deiminase type-2 (EC 3.5.3.15) (PAD-H19) (Peptidylarginine deiminase II) (Protein-arginine deiminase type II)	11	0	0	5	6	0	0	0	0	0	-8.8	95% (0.00091)
P35354	PTGS2	Prostaglandin G/H synthase 2 (EC 1.14.99.1) (Cyclooxygenase-2) (COX-2) (PHS II) (Prostaglandin H2 synthase 2) (PGH synthase 2) (PGHS-2)	11	0	2	6	3	0	0	0	0	0	-8.8	95% (0.00091)

		(Prostaglandin-endoperoxide synthase 2)												
Q08AI8	C2orf54	Uncharacterized protein C2orf54	10	0	3	0	0	7	0	0	0	0	-8.0	95% (0.0017)
E9PSH2	CD55	Complement decay-accelerating factor	10	0	2	4	4	0	0	0	0	0	-8.0	95% (0.0017)
P01859	IGHG2	Ig gamma-2 chain C region	10	0	0	0	0	6	0	0	0	0	-8.0	95% (0.0017)
Q8NI35	INADL	InaD-like protein (Inadl protein) (hINADL) (Pals1-associated tight junction protein) (Protein associated to tight junctions)	10	0	9	0	0	1	0	0	0	0	-8.0	95% (0.0017)
J3QRN6	MYO1D	Unconventional myosin-Id	10	0	10	0	0	0	0	0	0	0	-8.0	95% (0.0017)
P13727	PRG2	Bone marrow proteoglycan (BMPG) (Proteoglycan 2) [Cleaved into: Eosinophil granule major basic protein (EMBP) (MBP) (Pregnancy-associated major basic protein)]	10	0	4	0	0	6	0	0	0	0	-8.0	95% (0.0017)
B4DZS8		Peptidyl-prolyl cis-trans isomerase (Fragment)	10	0	0	4	6	0	0	0	0	0	-8.0	95% (0.0017)
Q03013	GSTM4	Glutathione S-transferase Mu 4 (EC 2.5.1.18) (GST class-mu 4) (GST-Mu2) (GSTM4-4)	9	0	0	2	0	7	0	0	0	0	-7.3	95% (0.0033)
P61601	NCALD	Neurocalcin-delta	9	0	4	0	0	2	0	0	0	0	-7.3	95% (0.0033)
Q5VYY1	ANKRD22	Ankyrin repeat domain-containing protein 22	8	0	2	0	1	5	0	0	0	0	-6.6	95% (0.0062)
Q9NUI1	DECR2	Peroxisomal 2,4-dienoyl-CoA reductase (pDCR) (EC 1.3.1.34) (2,4-dienoyl-CoA reductase 2)	8	0	4	0	0	4	0	0	0	0	-6.6	95% (0.0062)
Q9UHL4	DPP7	Dipeptidyl peptidase 2 (EC 3.4.14.2)	8	0	3	3	0	2	0	0	0	0	-6.6	95% (0.0062)

		(Dipeptidyl aminopeptidase II) (Dipeptidyl peptidase 7) (Dipeptidyl peptidase II) (DPP II) (Quiescent cell proline dipeptidase)												
Q8WWP7	GIMAP1	GTPase IMAP family member 1 (Immunity-associated protein 1) (hIMAP1)	8	0	0	2	2	3	0	0	0	0	-6.6	95% (0.0062)
E7ET40	PLAU	Urokinase-type plasminogen activator chain B	8	0	8	0	0	0	0	0	0	0	-6.6	95% (0.0062)
G3V207	TMCC3	Transmembrane and coiled-coil domain family 3, isoform CRA_c (Transmembrane and coiled-coil domains protein 3)	8	0	8	0	0	0	0	0	0	0	-6.6	95% (0.0062)
Q8IY95	TMEM192	Transmembrane protein 192	8	0	4	3	1	0	0	0	0	0	-6.6	95% (0.0062)
Q8TD43	TRPM4	Transient receptor potential cation channel subfamily M member 4 (hTRPM4) (Calcium-activated non-selective cation channel 1) (Long transient receptor potential channel 4) (LTrpC-4) (LTrpC4) (Melastatin-4)	8	0	8	0	0	0	0	0	0	0	-6.6	95% (0.0062)
B2RC75		Transmembrane channel-like protein	8	0	3	0	0	5	0	0	0	0	-6.6	95% (0.0062)
O75110	ATP9A	Probable phospholipid-transporting ATPase IIA (EC 3.6.3.1) (ATPase class II type 9A)	7	0	7	0	0	0	0	0	0	0	-5.9	95% (0.012)
Q96LJ7	DHRS1	Dehydrogenase/reductase SDR family member 1 (EC 1.1.-.-)	7	0	3	0	0	4	0	0	0	0	-5.9	95% (0.012)

Q8IWB1	ITPRIP	Inositol 1,4,5-trisphosphate receptor-interacting protein (Protein DANGER)	7	0	6	0	0	1	0	0	0	0	-5.9	95% (0.012)
E9PDY6	MUC4	Mucin-4 beta chain	7	0	6	1	0	0	0	0	0	0	-5.9	95% (0.012)
Q9NQX4	MYO5C	Unconventional myosin-Vc	7	0	7	0	0	0	0	0	0	0	-5.9	95% (0.012)
Q7L523	RRAGA	Ras-related GTP-binding protein A (Rag A) (RagA) (Adenovirus E3 14.7 kDa-interacting protein 1) (FIP-1)	7	0	1	1	3	2	0	0	0	0	-5.9	95% (0.012)
Q13363	CTBP1	C-terminal-binding protein 1 (CtBP1) (EC 1.1.1.-)	6	0	0	1	3	2	0	0	0	0	-5.2	95% (0.022)
E7ET76	GGT1	Gamma-glutamyltranspeptidase 1 light chain (Fragment)	6	0	3	1	2	0	0	0	0	0	-5.2	95% (0.022)
Q5T601	GPR110	Probable G-protein coupled receptor 110 (G-protein coupled receptor KPG_012) (G-protein coupled receptor PGR19)	6	0	4	0	0	2	0	0	0	0	-5.2	95% (0.022)
B7ZKT9	KIAA1033	KIAA1033 protein	6	0	5	0	0	1	0	0	0	0	-5.2	95% (0.022)
A2BDK6	MAP1B	Microtubule-associated protein 1B (Microtubule-associated protein 1B, isoform CRA_a)	6	0	2	1	3	0	0	0	0	0	-5.2	95% (0.022)
C9JS50	STEAP4	Metalloreductase STEAP4 (Uncharacterized protein)	6	0	0	2	1	3	0	0	0	0	-5.2	95% (0.022)
E5KNQ5	TK2	Mitochondrial thymidine kinase 2 (Thymidine kinase 2, mitochondrial) (Thymidine kinase 2, mitochondrial, isoform CRA_e)	6	0	2	2	1	1	0	0	0	0	-5.2	95% (0.022)
Q16864	ATP6V1F	V-type proton ATPase subunit F (V-ATPase	5	0	1	1	1	2	0	0	0	0	-4.5	95% (0.042)

		subunit F) (V-ATPase 14 kDa subunit) (Vacuolar proton pump subunit F)											
Q9Y3E8	C17orf25	CGI-150 protein	5	0	0	2	1	2	0	0	0	0	-4.5	95% (0.042)
Q96N67	DOCK7	Dedicator of cytokinesis protein 7	5	0	1	0	3	1	0	0	0	0	-4.5	95% (0.042)
P11678	EPX	Eosinophil peroxidase (EPO) (EC 1.11.1.7) [Cleaved into: Eosinophil peroxidase light chain; Eosinophil peroxidase heavy chain]	5	0	2	0	0	3	0	0	0	0	-4.5	95% (0.042)
Q9NPD3	EXOSC4	Exosome complex component RRP41 (Exosome component 4) (Ribosomal RNA-processing protein 41) (p12A)	5	0	0	3	0	2	0	0	0	0	-4.5	95% (0.042)
Q96J02	ITCH	E3 ubiquitin-protein ligase Itchy homolog (Itch) (EC 6.3.2.-) (Atrophin-1-interacting protein 4) (AIP4) (NFE2-associated polypeptide 1) (NAPP1)	5	0	1	1	3	0	0	0	0	0	-4.5	95% (0.042)
B2ZGL7	MGLL	Monoacylglycerol lipase isoform 2	5	0	4	1	0	0	0	0	0	0	-4.5	95% (0.042)
Q9BXB4	OSBPL11	Oxysterol-binding protein-related protein 11 (ORP-11) (OSBP-related protein 11)	5	0	1	1	2	1	0	0	0	0	-4.5	95% (0.042)
P41247	PNPLA4	Patatin-like phospholipase domain-containing protein 4 (EC 3.1.1.3) (Protein GS2)	5	0	4	0	1	0	0	0	0	0	-4.5	95% (0.042)
O75298	RTN2	Reticulon-2 (Neuroendocrine-specific protein-like 1) (NSP-like protein 1) (Neuroendocrine-specific	5	0	3	0	0	2	0	0	0	0	-4.5	95% (0.042)

		protein-like I) (NSP-like protein I) (NSPLI)											
Q14BN4	SLMAP	Sarcolemmal membrane-associated protein (Sarcolemmal-associated protein)	5	0	5	0	0	0	0	0	0	0	-4.5	95% (0.042)
Q5W111	SPRYD7	SPRY domain-containing protein 7 (Chronic lymphocytic leukemia deletion region gene 6 protein) (CLL deletion region gene 6 protein)	5	0	4	1	0	0	0	0	0	0	-4.5	95% (0.042)
C9K0W8	STX16	Syntaxin-16 (Uncharacterized protein)	5	0	3	1	1	0	0	0	0	0	-4.5	95% (0.042)
Q9C0C2	TNKS1BP1	182 kDa tankyrase-1-binding protein	5	0	2	1	2	0	0	0	0	0	-4.5	95% (0.042)
Q53GD8		Peptidyl-prolyl cis-trans isomerase (Fragment)	5	0	0	1	0	4	0	0	0	0	-4.5	95% (0.042)

[A]Obtained from UniProt (http://www.uniprot.org/) databases
[C]Spectral counts (SpC) in CN datasets (combined)
[D]Spectral counts (SpC) in CR datasets (combined)
[E]Spectral count ratio (Rsc) between CR (combined) and CN (combined)
[F]Statistical significance (p-value) (Fisher's Exact Test)

Table S5. Proteins identified as unique to CR samples.

Uniprot Acc[A]	Gene Symbol[A]	Protein Description[A]	CN_Combined[C]	CR_Combined[D]	CN_1	CN_2	CN_3	CN_4	CR_1	CR_2	CR_3	CR_4	RSC (CR / CN)[E]	p value[F]
P23378	GLDC	Glycine dehydrogenase [decarboxylating], mitochondrial (EC 1.4.4.2) (Glycine cleavage system P protein) (Glycine decarboxylase)	0	21	0	0	0	0	20	0	0	2	19.9	95% (< 0.00010)
P21266	GSTM3	Glutathione S-transferase Mu 3 (EC 2.5.1.18) (GST class-mu 3) (GSTM3-3) (hGSTM3-3)	0	21	0	0	0	0	3	14	4	0	19.9	95% (< 0.00010)
Q56FN6	HLA-DRB1	MHC class II antigen	0	16	0	0	0	0	0	9	0	0	15.5	95% (< 0.00010)
Q13085	ACACA	Acetyl-CoA carboxylase 1 (ACC1) (EC 6.4.1.2) (ACC-alpha) [Includes: Biotin carboxylase (EC 6.3.4.14)]	0	13	0	0	0	0	12	0	0	0	12.8	95% (< 0.00010)
Q52NV4	HRS	Histidyl-tRNA synthetase	0	12	0	0	0	0	6	0	2	0	11.9	95% (0.00012)
C9J837	RAB3GAP1	Rab3 GTPase-activating protein catalytic subunit	0	11	0	0	0	0	0	9	2	0	11.0	95% (0.00025)
P42771	CDKN2A	Cyclin-dependent kinase inhibitor 2A, isoforms 1/2/3 (Cyclin-dependent kinase 4 inhibitor A) (CDK4I) (Multiple tumor suppressor 1) (MTS-1) (p16-INK4a) (p16-INK4) (p16INK4A)	0	9	0	0	0	0	3	6	0	0	9.2	95% (0.0011)
B4DF41	MSH6	DNA mismatch repair protein Msh6 (cDNA FLJ55677, highly similar to DNA mismatch repair protein MSH6)	0	9	0	0	0	0	8	0	1	0	9.2	95% (0.0011)
B3KMS0		Condensin complex subunit 1	0	9	0	0	0	0	8	0	1	0	9.2	95% (0.0011)
Q86YH3	EARS2	EARS2 protein	0	8	0	0	0	0	1	0	2	4	8.3	95% (0.0024)
D6RE83	UCHL1	Ubiquitin carboxyl-terminal hydrolase isozyme L1	0	8	0	0	0	0	0	5	3	0	8.3	95% (0.0024)

Q04828	AKR1C1	Aldo-keto reductase family 1 member C1 (EC 1.1.1.-) (20-alpha-hydroxysteroid dehydrogenase) (20-alpha-HSD) (EC 1.1.1.149) (Chlordecone reductase homolog HAKRC) (Dihydrodiol dehydrogenase 1/2) (DD1/DD2) (High-affinity hepatic bile acid-binding protein) (HBAB) (Indanol dehydrogenase) (EC 1.1.1.112) (Trans-1,2-dihydrobenzene-1,2-diol dehydrogenase) (EC 1.3.1.20)	0	7	0	0	0	0	0	6	1	0	7.4	95% (0.0051)
P55285	CDH6	Cadherin-6 (Kidney cadherin) (K-cadherin)	0	7	0	0	0	0	4	0	1	2	7.4	95% (0.0051)
P36405	ARL3	ADP-ribosylation factor-like protein 3	0	6	0	0	0	0	1	4	1	0	6.5	95% (0.011)
B4DIH5	COPS2	COP9 signalosome complex subunit 2 (cDNA FLJ52928, highly similar to COP9 signalosome complex subunit 2)	0	6	0	0	0	0	3	2	1	0	6.5	95% (0.011)
D3DNT9	EPHB3	EPH receptor B3, isoform CRA_a	0	6	0	0	0	0	4	0	2	0	6.5	95% (0.011)
P21397	MAOA	Amine oxidase [flavin-containing] A (EC 1.4.3.4) (Monoamine oxidase type A) (MAO-A)	0	6	0	0	0	0	0	3	0	3	6.5	95% (0.011)
Q14669	TRIP12	E3 ubiquitin-protein ligase TRIP12 (EC 6.3.2.-) (E3 ubiquitin-protein ligase for Arf) (ULF) (Thyroid receptor-interacting protein 12) (TR-interacting protein 12) (TRIP-12)	0	6	0	0	0	0	2	0	4	0	6.5	95% (0.011)
A8K984		Structural maintenance of chromosomes protein	0	6	0	0	0	0	4	0	1	0	6.5	95% (0.011)

P11766	ADH5	Alcohol dehydrogenase class-3 (EC 1.1.1.1) (Alcohol dehydrogenase 5) (Alcohol dehydrogenase class chi chain) (Alcohol dehydrogenase class-III) (Glutathione-dependent formaldehyde dehydrogenase) (FALDH) (FDH) (GSH-FDH) (EC 1.1.1.-) (S-(hydroxymethyl)glutathione dehydrogenase) (EC 1.1.1.284)	0	5	0	0	0	0	2	3	0	0	5.6	95% (0.023)
B7ZMD7	AMY1A	Amylase, alpha 1A (Salivary)	0	5	0	0	0	0	0	5	0	0	5.6	95% (0.023)
O14965	AURKA	Aurora kinase A (EC 2.7.11.1) (Aurora 2) (Aurora/IPL1-related kinase 1) (ARK-1) (Aurora-related kinase 1) (hARK1) (Breast tumor-amplified kinase) (Serine/threonine-protein kinase 15) (Serine/threonine-protein kinase 6) (Serine/threonine-protein kinase aurora-A)	0	5	0	0	0	0	0	3	1	0	5.6	95% (0.023)
Q96GD4	AURKB	Aurora kinase B (EC 2.7.11.1) (Aurora 1) (Aurora- and IPL1-like midbody-associated protein 1) (AIM-1) (Aurora/IPL1-related kinase 2) (ARK-2) (Aurora-related kinase 2) (STK-1) (Serine/threonine-protein kinase 12) (Serine/threonine-protein kinase 5) (Serine/threonine-protein kinase aurora-B)	0	5	0	0	0	0	1	0	4	0	5.6	95% (0.023)
O43708	GSTZ1	Maleylacetoacetate isomerase (MAAI) (EC 5.2.1.2) (GSTZ1-1) (Glutathione S-transferase zeta 1) (EC 2.5.1.18)	0	5	0	0	0	0	2	1	2	0	5.6	95% (0.023)

Q86Y56	HEATR2	HEAT repeat-containing protein 2	0	5	0	0	0	0	1	2	1	0	5.6	95% (0.023)
Q68E01	INTS3	Integrator complex subunit 3 (Int3) (SOSS complex subunit A) (Sensor of single-strand DNA complex subunit A) (SOSS-A) (Sensor of ssDNA subunit A)	0	5	0	0	0	0	1	1	2	1	5.6	95% (0.023)
Q15397	KIAA0020	Pumilio domain-containing protein KIAA0020 (HBV X-transactivated gene 5 protein) (HBV XAg-transactivated protein 5) (Minor histocompatibility antigen HA-8) (HLA-HA8)	0	5	0	0	0	0	2	2	1	0	5.6	95% (0.023)
E7ESY4	MTA1	Metastasis-associated protein MTA1	0	5	0	0	0	0	1	0	3	0	5.6	95% (0.023)
Q9Y263	PLAA	Phospholipase A-2-activating protein (PLA2P) (PLAP)	0	5	0	0	0	0	2	3	0	0	5.6	95% (0.023)
E9PKF6	PPP6R3	Serine/threonine-protein phosphatase 6 regulatory subunit 3	0	5	0	0	0	0	0	4	1	0	5.6	95% (0.023)
Q9C0C9	UBE2O	Ubiquitin-conjugating enzyme E2 O (EC 6.3.2.19) (Ubiquitin carrier protein O) (Ubiquitin-conjugating enzyme E2 of 230 kDa) (Ubiquitin-conjugating enzyme E2-230K) (Ubiquitin-protein ligase O)	0	5	0	0	0	0	0	4	1	0	5.6	95% (0.023)
Q59GW6		Acetyl-CoA acetyltransferase, cytosolic variant (Fragment)	0	5	0	0	0	0	5	0	0	0	5.6	95% (0.023)
Q9NZJ6	COQ3	Hexaprenyldihydroxybenzoate methyltransferase, mitochondrial (EC 2.1.1.114) (2-polyprenyl-6-hydroxyphenol methylase) (EC 2.1.1.222) (3,4-dihydroxy-5-hexaprenylbenzoate methyltransferase) (DHHB methyltransferase) (DHHB-	0	4	0	0	0	0	4	0	0	0	4.7	95% (0.049)

		MT) (DHHB-MTase) (3-demethylubiquinone-10 3-methyltransferase) (EC 2.1.1.64) (Dihydroxyhexaprenylbenzoate methyltransferase)											
P29762	CRABP1	Cellular retinoic acid-binding protein 1 (Cellular retinoic acid-binding protein I) (CRABP-I)	0	4	0	0	0	0	0	4	0	0	4.7	95% (0.049)
P02741	CRP	C-reactive protein [Cleaved into: C-reactive protein(1-205)]	0	4	0	0	0	0	0	0	0	4	4.7	95% (0.049)
P48507	GCLM	Glutamate--cysteine ligase regulatory subunit (GCS light chain) (Gamma-ECS regulatory subunit) (Gamma-glutamylcysteine synthetase regulatory subunit) (Glutamate--cysteine ligase modifier subunit)	0	4	0	0	0	0	0	4	0	0	4.7	95% (0.049)
Q96IJ6	GMPPA	Mannose-1-phosphate guanyltransferase alpha (EC 2.7.7.13) (GDP-mannose pyrophosphorylase A) (GTP-mannose-1-phosphate guanylyltransferase alpha)	0	4	0	0	0	0	0	2	1	1	4.7	95% (0.049)
Q9BYC9	MRPL20	39S ribosomal protein L20, mitochondrial (L20mt) (MRP-L20)	0	4	0	0	0	0	1	1	0	2	4.7	95% (0.049)
Q9UKX7	NUP50	Nuclear pore complex protein Nup50 (50 kDa nucleoporin) (Nuclear pore-associated protein 60 kDa-like) (Nucleoporin Nup50)	0	4	0	0	0	0	2	0	2	0	4.7	95% (0.049)
E7EU94	PRPF31	U4/U6 small nuclear ribonucleoprotein Prp31 (Fragment)	0	4	0	0	0	0	2	2	0	0	4.7	95% (0.049)
P26022	PTX3	Pentraxin-related protein PTX3 (Pentaxin-related protein PTX3) (Tumor necrosis factor	0	4	0	0	0	0	1	0	3	0	4.7	95% (0.049)

		alpha-induced protein 5) (TNF alpha-induced protein 5) (Tumor necrosis factor-inducible gene 14 protein) (TSG-14)											
Q96AH8	RAB7B	Ras-related protein Rab-7b	0	4	0	0	0	0	0	0	0	4	4.7	95% (0.049)
J3KNH7	SENP3	Sentrin-specific protease 3	0	4	0	0	0	0	2	0	2	0	4.7	95% (0.049)
Q96K37	SLC35E1	Solute carrier family 35 member E1	0	4	0	0	0	0	0	2	0	2	4.7	95% (0.049)
Q15599	SLC9A3R2	Na(+)/H(+) exchange regulatory cofactor NHE-RF2 (NHERF-2) (NHE3 kinase A regulatory protein E3KARP) (SRY-interacting protein 1) (SIP-1) (Sodium-hydrogen exchanger regulatory factor 2) (Solute carrier family 9 isoform A3 regulatory factor 2) (Tyrosine kinase activator protein 1) (TKA-1)	0	4	0	0	0	0	2	0	1	1	4.7	95% (0.049)
A8KAM9		Peptidyl-prolyl cis-trans isomerase E (PPIase E) (EC 5.2.1.8)	0	4	0	0	0	0	2	0	2	0	4.7	95% (0.049)

[A]Obtained from UniProt (http://www.uniprot.org/) databases
[C]Spectral counts (SpC) in CN datasets (combined)
[D]Spectral counts (SpC) in CR datasets (combined)
[E]Spectral count ratio (Rsc) between CR (combined) and CN (combined)
[F]Statistical significance (p-value) (Fisher's Exact Test)

Table S6. Proteins which meet the selection criteria and are identified in both CN and CR samples.

Uniprot Acc[A]	Gene Symbol[A]	Protein Description[A]	CN_Combined[C]	CR_Combined[D]	CN_1	CN_2	CN_3	CN_4	CR_1	CR_2	CR_3	CR_4	RSC (CR / CN)[E]	p value[F]
Q99715	COL12A1	Collagen alpha-1(XII) chain	1	23	0	0	1	0	12	1	6	4	12.1	95% (< 0.00010)
P08243	ASNS	Asparagine synthetase [glutamine-hydrolyzing] (EC 6.3.5.4) (Cell cycle control protein TS11) (Glutamine-dependent asparagine synthetase)	1	20	0	0	1	0	9	10	1	0	10.6	95% (< 0.00010)
P37268	FDFT1	Squalene synthase (SQS) (SS) (EC 2.5.1.21) (FPP:FPP farnesyltransferase) (Farnesyl-diphosphate farnesyltransferase)	1	12	1	0	0	0	9	1	2	0	6.6	95% (0.00087)
O60701	UGDH	UDP-glucose 6-dehydrogenase (UDP-Glc dehydrogenase) (UDP-GlcDH) (UDPGDH) (EC 1.1.1.22)	3	23	1	1	1	0	2	15	6	0	6.4	95% (< 0.00010)
Q96C36	PYCR2	Pyrroline-5-carboxylate reductase 2 (P5C reductase 2) (P5CR 2) (EC 1.5.1.2)	4	26	0	0	0	3	12	12	12	12	5.8	95% (< 0.00010)
Q9UKF6	CPSF3	Cleavage and polyadenylation specificity factor subunit 3 (EC 3.1.27.-) (Cleavage and polyadenylation specificity factor 73 kDa subunit) (CPSF 73 kDa subunit) (mRNA 3'-end-processing endonuclease CPSF-73)	1	10	0	0	1	0	1	0	7	2	5.6	95% (0.0034)
B4DHQ3		Phosphoserine aminotransferase (EC 2.6.1.52)	1	10	0	0	0	1	5	4	1	0	5.6	95% (0.0034)
Q8NFF5	FLAD1	FAD synthase (EC 2.7.7.2) (FAD pyrophosphorylase) (FMN adenylyltransferase) (Flavin adenine dinucleotide synthase) [Includes: Molybdenum cofactor biosynthesis protein-like region; FAD synthase region]	3	19	0	0	0	3	11	2	3	3	5.3	95% (0.00016)
Q9Y5Q9	GTF3C3	General transcription factor 3C polypeptide 3 (Transcription factor	1	9	0	0	0	1	4	0	3	2	5.1	95% (0.0065)

		IIIC 102 kDa subunit) (TFIIIC 102 kDa subunit) (TFIIIC102) (Transcription factor IIIC subunit gamma) (TF3C-gamma)											
O15230	LAMA5	Laminin subunit alpha-5 (Laminin-10 subunit alpha) (Laminin-11 subunit alpha) (Laminin-15 subunit alpha)	1	9	1	0	0	0	3	1	3	2	5.1	95% (0.0065)
Q6UX53	METTL7B	Methyltransferase-like protein 7B (EC 2.1.1.-)	1	9	1	0	0	0	6	1	1	1	5.1	95% (0.0065)
P52569	SLC7A2	Low affinity cationic amino acid transporter 2 (CAT-2) (CAT2) (Solute carrier family 7 member 2)	1	9	1	0	0	0	1	3	2	3	5.1	95% (0.0065)
J3KR97	TBCD	Tubulin-specific chaperone D	1	9	0	1	0	0	1	7	1	0	5.1	95% (0.0065)
B4DMB5		CTP synthase (EC 6.3.4.2) (UTP--ammonia ligase)	1	9	0	0	0	1	5	5	0	0	5.1	95% (0.0065)
P50851	LRBA	Lipopolysaccharide-responsive and beige-like anchor protein (Beige-like protein) (CDC4-like protein)	3	18	3	0	0	0	5	9	3	2	5.1	95% (0.00029)
Q96HN2	AHCYL2	Putative adenosylhomocysteinase 3 (AdoHcyase 3) (EC 3.3.1.1) (S-adenosyl-L-homocysteine hydrolase 3) (S-adenosylhomocysteine hydrolase-like protein 2)	1	8	0	0	0	1	2	2	4	0	4.6	95% (0.013)
O94766	B3GAT3	Galactosylgalactosylxylosylprotein 3-beta-glucuronosyltransferase 3 (EC 2.4.1.135) (Beta-1,3-glucuronyltransferase 3) (Glucuronosyltransferase I) (GlcAT-I) (UDP-GlcUA:Gal beta-1,3-Gal-R glucuronyltransferase) (GlcUAT-I)	1	8	0	0	0	1	2	1	2	3	4.6	95% (0.013)
O75208	COQ9	Ubiquinone biosynthesis protein COQ9, mitochondrial	1	8	1	0	0	0	6	0	2	0	4.6	95% (0.013)
P14324	FDPS	Farnesyl pyrophosphate synthase (FPP synthase) (FPS) (EC 2.5.1.10) ((2E,6E)-farnesyl diphosphate synthase) (Dimethylallyltranstransferase) (EC	1	8	0	1	0	0	7	0	1	0	4.6	95% (0.013)

		2.5.1.1) (Farnesyl diphosphate synthase) (Geranyltranstransferase)											
B4E1G6	GALK1	Galactokinase (Galactokinase 1) (cDNA FLJ56840, highly similar to Galactokinase (EC 2.7.1.6))	1	8	0	1	0	0	0	6	2	0	4.6	95% (0.013)
A0JP11	PIK3R4	Phosphoinositide-3-kinase, regulatory subunit 4	1	8	0	1	0	0	5	2	1	0	4.6	95% (0.013)
P63151	PPP2R2A	Serine/threonine-protein phosphatase 2A 55 kDa regulatory subunit B alpha isoform (PP2A subunit B isoform B55-alpha) (PP2A subunit B isoform PR55-alpha) (PP2A subunit B isoform R2-alpha) (PP2A subunit B isoform alpha)	1	8	1	0	0	0	3	4	2	0	4.6	95% (0.013)
P98175	RBM10	RNA-binding protein 10 (G patch domain-containing protein 9) (RNA-binding motif protein 10) (RNA-binding protein S1-1) (S1-1)	1	8	0	0	0	1	1	0	4	3	4.6	95% (0.013)
E1NZA1	PRIC295	Peroxisome proliferator activated receptor interacting complex protein	21	90	4	2	2	13	40	38	12	2	4.6	95% (< 0.00010)
Q8IWA0	WDR75	WD repeat-containing protein 75	2	12	0	2	0	0	9	0	3	0	4.6	95% (0.0035)
P36222	CHI3L1	Chitinase-3-like protein 1 (39 kDa synovial protein) (Cartilage glycoprotein 39) (CGP-39) (GP-39) (hCGP-39) (YKL-40)	5	24	3	1	0	1	2	1	3	18	4.5	95% (< 0.00010)
Q8TD30	GPT2	Alanine aminotransferase 2 (ALT2) (EC 2.6.1.2) (Glutamate pyruvate transaminase 2) (GPT 2) (Glutamic--alanine transaminase 2) (Glutamic--pyruvic transaminase 2)	5	24	0	1	1	3	14	9	1	0	4.5	95% (< 0.00010)
Q92626	PXDN	Peroxidasin homolog (EC 1.11.1.7) (Melanoma-associated antigen MG50) (Vascular peroxidase 1) (p53-responsive gene 2 protein)	4	19	4	0	0	0	10	1	2	6	4.3	95% (0.00051)
P61221	ABCE1	ATP-binding cassette sub-family E member 1 (2'-5'-oligoadenylate-binding protein) (HuHP68) (RNase L inhibitor) (Ribonuclease 4 inhibitor) (RNS4I)	3	15	0	0	0	3	7	4	4	0	4.3	95% (0.0018)

Q8IZV5	RDH10	Retinol dehydrogenase 10 (EC 1.1.1.300)	2	11	1	0	1	0	0	9	1	1	4.2	95% (0.0064)
Q8NBL1	POGLUT1	Protein O-glucosyltransferase 1 (EC 2.4.1.-) (CAP10-like 46 kDa protein) (hCLP46) (KTEL motif-containing protein 1) (Myelodysplastic syndromes relative protein)	4	18	0	2	1	1	8	4	2	5	4.1	95% (0.00090)
Q92905	COPS5	COP9 signalosome complex subunit 5 (SGN5) (Signalosome subunit 5) (EC 3.4.-.-) (Jun activation domain-binding protein 1)	1	7	0	0	0	1	4	1	2	0	4.1	95% (0.024)
B7Z5H3	MAOB	Amine oxidase [flavin-containing] B (cDNA FLJ52418, highly similar to Amine oxidase (flavin-containing) B (EC 1.4.3.4))	1	7	0	0	0	1	0	7	0	0	4.1	95% (0.024)
Q9Y266	NUDC	Nuclear migration protein nudC (Nuclear distribution protein C homolog)	1	7	0	0	0	1	5	0	2	0	4.1	95% (0.024)
P00738	HP	Haptoglobin (Zonulin) [Cleaved into: Haptoglobin alpha chain; Haptoglobin beta chain]	3	14	0	2	0	1	0	2	5	6	4.0	95% (0.0032)
P02748	C9	Complement component C9 [Cleaved into: Complement component C9a; Complement component C9b]	2	10	0	0	0	2	0	0	0	10	3.9	95% (0.012)
Q9BSD7	NTPCR	Cancer-related nucleoside-triphosphatase (NTPase) (EC 3.6.1.15) (Nucleoside triphosphate phosphohydrolase)	2	10	2	0	0	0	1	2	0	7	3.9	95% (0.012)
Q14151	SAFB2	Scaffold attachment factor B2 (SAF-B2)	2	10	2	0	0	0	6	0	2	2	3.9	95% (0.012)
P11388	TOP2A	DNA topoisomerase 2-alpha (EC 5.99.1.3) (DNA topoisomerase II, alpha isozyme)	20	68	0	9	9	2	26	18	14	10	3.7	95% (< 0.00010)
Q5JTZ9	AARS2	Alanine--tRNA ligase, mitochondrial (EC 6.1.1.7) (Alanyl-tRNA synthetase) (AlaRS)	1	6	1	0	0	0	5	0	0	1	3.6	95% (0.045)
B4DQ14	EIF2A	Eukaryotic translation initiation factor 2A (cDNA FLJ58035, highly	1	6	0	0	0	1	3	3	1	0	3.6	95% (0.045)

		similar to Homo sapiens eukaryotic translation initiation factor (eIF) 2A (eIF2A), mRNA)											
Q14232	EIF2B1	Translation initiation factor eIF-2B subunit alpha (eIF-2B GDP-GTP exchange factor subunit alpha)	1	6	0	0	0	1	3	3	0	0	3.6	95% (0.045)
Q96ND0	FAM210A	Protein FAM210A	1	6	0	0	1	0	1	2	2	1	3.6	95% (0.045)
P82675	MRPS5	28S ribosomal protein S5, mitochondrial (MRP-S5) (S5mt)	1	6	1	0	0	0	1	0	1	4	3.6	95% (0.045)
B4DU97	SLC25A17	Peroxisomal membrane protein PMP34 (cDNA FLJ57596, highly similar to Peroxisomal membrane protein PMP34)	1	6	0	1	0	0	2	4	0	0	3.6	95% (0.045)
Q8NCA5	FAM98A	Protein FAM98A	2	9	0	0	0	2	3	3	1	2	3.5	95% (0.021)
Q69YN4	KIAA1429	Protein virilizer homolog	2	9	0	1	1	0	2	0	7	0	3.5	95% (0.021)
Q96T76	MMS19	MMS19 nucleotide excision repair protein homolog (hMMS19) (MET18 homolog) (MMS19-like protein)	2	9	0	0	0	2	2	5	1	0	3.5	95% (0.021)
P05165	PCCA	Propionyl-CoA carboxylase alpha chain, mitochondrial (PCCase subunit alpha) (EC 6.4.1.3) (Propanoyl-CoA:carbon dioxide ligase subunit alpha)	2	9	0	0	1	1	3	0	1	5	3.5	95% (0.021)
P49588	AARS	Alanine--tRNA ligase, cytoplasmic (EC 6.1.1.7) (Alanyl-tRNA synthetase) (AlaRS) (Renal carcinoma antigen NY-REN-42)	11	37	0	2	4	5	15	18	6	0	3.5	95% (< 0.00010)
Q13131	PRKAA1	5'-AMP-activated protein kinase catalytic subunit alpha-1 (AMPK subunit alpha-1) (EC 2.7.11.1) (Acetyl-CoA carboxylase kinase) (ACACA kinase) (EC 2.7.11.27) (Hydroxymethylglutaryl-CoA reductase kinase) (HMGCR kinase) (EC 2.7.11.31) (Tau-protein kinase PRKAA1) (EC 2.7.11.26)	3	12	0	2	1	0	5	5	2	0	3.5	95% (0.0098)
B9A018	USP39	U4/U6.U5 tri-snRNP-associated protein 2	3	12	1	0	2	0	0	0	11	1	3.5	95% (0.0098)

Q59FH1		Transformation/transcription domain-associated protein variant (Fragment)	3	12	1	0	0	2	2	0	9	1	3.5	95% (0.0098)
Q9H3U1	UNC45A	Protein unc-45 homolog A (Unc-45A) (GCUNC-45) (Smooth muscle cell-associated protein 1) (SMAP-1)	4	15	0	0	0	4	2	8	4	0	3.5	95% (0.0047)
E7EUY3	PCCB	Propionyl-CoA carboxylase beta chain, mitochondrial	9	29	1	3	2	3	17	1	3	9	3.3	95% (0.00023)
M1V485	SLC34A2-ROS1	Tyrosine-protein kinase receptor (EC 2.7.10.1)	13	40	4	4	2	3	8	1	14	18	3.2	95% (< 0.00010)
Q96KA5	CLPTM1L	Cleft lip and palate transmembrane protein 1-like protein (CLPTM1-like protein) (Cisplatin resistance-related protein 9) (CRR9p)	2	8	0	0	2	0	2	0	4	2	3.2	95% (0.037)
A7J992	PHIP	Pleckstrin homology domain-interacting protein variant 1	2	8	0	1	0	1	4	0	3	1	3.2	95% (0.037)
Q96T51	RUFY1	RUN and FYVE domain-containing protein 1 (FYVE-finger protein EIP1) (La-binding protein 1) (Rab4-interacting protein) (Zinc finger FYVE domain-containing protein 12)	2	8	1	0	0	1	3	2	3	0	3.2	95% (0.037)
B4DHJ3		DNA-directed RNA polymerase (EC 2.7.7.6)	6	19	0	2	1	3	2	2	12	3	3.1	95% (0.0031)
K7ELW0	PARK7	Protein DJ-1	5	16	1	1	2	1	5	5	3	3	3.1	95% (0.0065)
P50895	BCAM	Basal cell adhesion molecule (Auberger B antigen) (B-CAM cell surface glycoprotein) (F8/G253 antigen) (Lutheran antigen) (Lutheran blood group glycoprotein) (CD antigen CD239)	11	32	0	2	1	8	14	0	16	2	3.0	95% (0.00025)
Q6IAX9	SRPR	SRPR protein	17	48	0	5	6	6	18	12	15	4	3.0	95% (< 0.00010)
F8VPD4	CAD	CAD protein	10	29	3	2	2	3	17	7	4	1	3.0	95% (0.00049)
O00468	AGRN	Agrin [Cleaved into: Agrin N-terminal 110 kDa subunit; Agrin C-terminal 110 kDa subunit; Agrin C-terminal 90 kDa fragment (C90);	3	10	0	2	0	1	8	0	2	0	3.0	95% (0.029)

		Agrin C-terminal 22 kDa fragment (C22)]											
F5GZS0	DHX36	Probable ATP-dependent RNA helicase DHX36	3	10	0	1	2	0	5	2	2	1	3.0	95% (0.029)
B4DJK9		Perilipin	3	10	1	0	1	1	0	1	3	6	3.0	95% (0.029)
Q6NVW7	KPNA2	Importin subunit alpha	13	36	0	3	8	2	5	8	18	5	2.9	95% (0.00016)
P18583	SON	Protein SON (Bax antagonist selected in saccharomyces 1) (BASS1) (Negative regulatory element-binding protein) (NRE-binding protein) (Protein DBP-5) (SON3)	5	15	0	1	3	1	6	0	6	3	2.9	95% (0.011)
E7D7X9		Pyrroline-5-carboxylate reductase (EC 1.5.1.2)	5	15	0	2	2	1	5	6	4	0	2.9	95% (0.011)
Q53GS0		Nucleolar GTP-binding protein 1 (Fragment)	4	12	1	0	0	2	6	2	4	0	2.8	95% (0.023)
P22102	GART	Trifunctional purine biosynthetic protein adenosine-3 [Includes: Phosphoribosylamine--glycine ligase (EC 6.3.4.13) (Glycinamide ribonucleotide synthetase) (GARS) (Phosphoribosylglycinamide synthetase); Phosphoribosylformylglycinamidine cyclo-ligase (EC 6.3.3.1) (AIR synthase) (AIRS) (Phosphoribosyl-aminoimidazole synthetase); Phosphoribosylglycinamide formyltransferase (EC 2.1.2.2) (5'-phosphoribosylglycinamide transformylase) (GAR transformylase) (GART)]	8	22	0	1	1	6	9	9	4	0	2.8	95% (0.0032)
Q6IBU0	EIF5	EIF5 protein	5	14	0	2	2	1	3	7	4	0	2.7	95% (0.017)
A2RTY6	ITIH2	Inter-alpha (Globulin) inhibitor H2 (Inter-alpha (Globulin) inhibitor H2, isoform CRA_b) (cDNA FLJ75038, highly similar to Homo sapiens inter-alpha (globulin) inhibitor H2 (ITIH2), mRNA)	3	9	0	1	1	1	2	4	1	2	2.7	95% (0.049)

B4E1K0	KIF23	Kinesin-like protein KIF23 (cDNA FLJ58416, highly similar to Kinesin-like protein KIF23)	3	9	0	2	1	0	2	1	4	2	2.7	95% (0.049)
Q06AH7	TF	Transferrin	34	83	1	11	10	12	27	13	30	14	2.7	95% (< 0.00010)
Q13057	COASY	Bifunctional coenzyme A synthase (CoA synthase) (NBP) (POV-2) [Includes: Phosphopantetheine adenylyltransferase (EC 2.7.7.3) (Dephospho-CoA pyrophosphorylase) (Pantetheine-phosphate adenylyltransferase) (PPAT); Dephospho-CoA kinase (DPCK) (EC 2.7.1.24) (Dephosphocoenzyme A kinase) (DPCOAK)]	4	11	0	0	1	3	5	3	2	1	2.6	95% (0.037)
O95864	FADS2	Fatty acid desaturase 2 (EC 1.14.19.-) (Delta(6) fatty acid desaturase) (D6D) (Delta(6) desaturase) (Delta-6 desaturase)	4	11	0	2	2	0	2	0	9	0	2.6	95% (0.037)
J3KN16	KIAA0368	Proteasome-associated protein ECM29 homolog	4	11	0	0	0	4	3	4	4	0	2.6	95% (0.037)
E9PF74	SLC25A19	Mitochondrial thiamine pyrophosphate carrier	4	11	0	0	1	3	1	2	1	7	2.6	95% (0.037)
Q7L014	DDX46	Probable ATP-dependent RNA helicase DDX46 (EC 3.6.4.13) (DEAD box protein 46) (PRP5 homolog)	11	27	0	2	2	6	5	4	14	4	2.6	95% (0.0024)
P68104	EEF1A1	Elongation factor 1-alpha 1 (EF-1-alpha-1) (Elongation factor Tu) (EF-Tu) (Eukaryotic elongation factor 1 A-1) (eEF1A-1) (Leukocyte receptor cluster member 7)	96	222	13	28	24	32	26	137	40	22	2.6	95% (< 0.00010)
Q9H5Q4	TFB2M	Dimethyladenosine transferase 2, mitochondrial (EC 2.1.1.-) (Hepatitis C virus NS5A-transactivated protein 5) (HCV NS5A-transactivated protein 5) (Mitochondrial 12S rRNA dimethylase 2) (Mitochondrial	5	13	1	1	2	1	6	2	1	4	2.6	95% (0.028)

		transcription factor B2) (h-mtTFB) (h-mtTFB2) (hTFB2M) (mtTFB2) (S-adenosylmethionine-6-N', N'-adenosyl(rRNA) dimethyltransferase 2)												
P01023	A2M	Alpha-2-macroglobulin (Alpha-2-M) (C3 and PZP-like alpha-2-macroglobulin domain-containing protein 5)	9	22	0	1	7	1	2	1	6	12	2.5	95% (0.0062)
Q0VGA5	SARS	SARS protein	9	22	0	0	1	8	7	9	6	0	2.5	95% (0.0062)
Q5TC12	ATPAF1	ATP synthase mitochondrial F1 complex assembly factor 1 (ATP11 homolog)	6	15	1	2	2	1	10	2	3	0	2.5	95% (0.021)
P14174	MIF	Macrophage migration inhibitory factor (MIF) (EC 5.3.2.1) (Glycosylation-inhibiting factor) (GIF) (L-dopachrome isomerase) (L-dopachrome tautomerase) (EC 5.3.3.12) (Phenylpyruvate tautomerase)	8	19	1	3	4	0	4	7	5	3	2.5	95% (0.012)
P07741	APRT	Adenine phosphoribosyltransferase (APRT) (EC 2.4.2.7)	14	32	1	3	5	5	9	15	4	4	2.4	95% (0.0017)
Q8NCN5	PDPR	Pyruvate dehydrogenase phosphatase regulatory subunit, mitochondrial (PDPr)	14	32	2	5	6	1	21	2	1	9	2.4	95% (0.0017)
Q9H0S4	DDX47	Probable ATP-dependent RNA helicase DDX47 (EC 3.6.4.13) (DEAD box protein 47)	9	21	0	1	5	3	4	7	6	4	2.4	95% (0.0095)
P45974	USP5	Ubiquitin carboxyl-terminal hydrolase 5 (EC 3.4.19.12) (Deubiquitinating enzyme 5) (Isopeptidase T) (Ubiquitin thioesterase 5) (Ubiquitin-specific-processing protease 5)	10	23	0	0	3	7	6	15	3	0	2.4	95% (0.0073)
Q8NI27	THOC2	THO complex subunit 2 (Tho2) (hTREX120)	11	25	1	2	4	3	7	1	12	5	2.4	95% (0.0056)
Q5W0H4	TPT1	Translationally-controlled tumor protein	11	25	2	4	3	1	8	8	6	3	2.4	95% (0.0056)
O14976	GAK	Cyclin-G-associated kinase (EC 2.7.11.1)	5	12	0	1	2	3	3	6	3	0	2.4	95% (0.044)

Q9Y4W2	LAS1L	Ribosomal biogenesis protein LAS1L (Protein LAS1 homolog)	5	12	2	0	0	3	6	2	0	5	2.4	95% (0.044)
A5PLK7	RCC2	RCC2 protein (Fragment)	6	14	0	1	1	4	8	4	2	0	2.4	95% (0.033)
Q6URC4		Diaphanous 1	7	16	0	3	3	1	7	2	5	1	2.3	95% (0.025)
Q53GX7		Threonyl-tRNA synthetase variant (Fragment)	19	41	0	6	5	8	15	13	9	4	2.3	95% (0.00071)
O15254	ACOX3	Peroxisomal acyl-coenzyme A oxidase 3 (EC 1.3.3.6) (Branched-chain acyl-CoA oxidase) (BRCACox) (Pristanoyl-CoA oxidase)	8	18	7	0	1	0	1	8	3	6	2.3	95% (0.019)
Q9UIG0	BAZ1B	Tyrosine-protein kinase BAZ1B (EC 2.7.10.2) (Bromodomain adjacent to zinc finger domain protein 1B) (Williams syndrome transcription factor) (Williams-Beuren syndrome chromosomal region 10 protein) (Williams-Beuren syndrome chromosomal region 9 protein) (hWALp2)	8	18	2	2	0	5	14	0	3	1	2.3	95% (0.019)
P61289	PSME3	Proteasome activator complex subunit 3 (11S regulator complex subunit gamma) (REG-gamma) (Activator of multicatalytic protease subunit 3) (Ki nuclear autoantigen) (Proteasome activator 28 subunit gamma) (PA28g) (PA28gamma)	8	18	0	1	1	6	4	6	8	1	2.3	95% (0.019)
P10909	CLU	Clusterin (Aging-associated gene 4 protein) (Apolipoprotein J) (Apo-J) (Complement cytolysis inhibitor) (CLI) (Complement-associated protein SP-40,40) (Ku70-binding protein 1) (NA1/NA2) (Testosterone-repressed prostate message 2) (TRPM-2) [Cleaved into: Clusterin beta chain (ApoJalpha) (Complement cytolysis inhibitor a chain); Clusterin alpha chain (ApoJbeta) (Complement cytolysis inhibitor b chain)]	9	20	3	1	4	1	1	8	3	7	2.3	95% (0.014)

Q969X5	ERGIC1	Endoplasmic reticulum-Golgi intermediate compartment protein 1 (ER-Golgi intermediate compartment 32 kDa protein) (ERGIC-32)	9	20	7	0	0	2	2	6	7	5	2.3	95% (0.014)
P18428	LBP	Lipopolysaccharide-binding protein (LBP)	9	20	0	2	1	6	0	0	4	15	2.3	95% (0.014)
Q4LE36	ACLY	ACLY variant protein (Fragment)	31	64	0	12	9	10	28	24	11	2	2.3	95% (< 0.00010)
Q96RP9	GFM1	Elongation factor G, mitochondrial (EF-Gmt) (Elongation factor G 1, mitochondrial) (mEF-G 1) (Elongation factor G1) (hEFG1)	33	68	4	12	10	8	37	6	10	15	2.3	95% (< 0.00010)
B5BTZ6	STAT3	Signal transducer and activator of transcription	20	41	3	7	3	7	12	21	9	0	2.2	95% (0.0012)
P54577	YARS	Tyrosine--tRNA ligase, cytoplasmic (EC 6.1.1.1) (Tyrosyl-tRNA synthetase) (TyrRS) [Cleaved into: Tyrosine--tRNA ligase, cytoplasmic, N-terminally processed]	9	19	0	3	3	3	7	8	3	1	2.2	95% (0.022)
P11172	UMPS	Uridine 5'-monophosphate synthase (UMP synthase) [Includes: Orotate phosphoribosyltransferase (OPRT) (OPRTase) (EC 2.4.2.10); Orotidine 5'-phosphate decarboxylase (ODC) (EC 4.1.1.23) (OMPdecase)]	8	17	0	2	5	1	12	2	3	1	2.2	95% (0.029)
Q53FW8		TAP binding protein-like variant (Fragment)	7	15	2	0	0	6	0	11	0	5	2.2	95% (0.038)
Q53G19		Mitochondrial ribosomal protein L11 isoform a variant (Fragment)	7	15	3	3	0	1	4	0	5	6	2.2	95% (0.038)
P01024	C3	Complement C3 (C3 and PZP-like alpha-2-macroglobulin domain-containing protein 1) [Cleaved into: Complement C3 beta chain; Complement C3 alpha chain; C3a anaphylatoxin; Acylation stimulating protein (ASP) (C3adesArg); Complement C3b alpha' chain; Complement C3c	54	104	18	11	11	15	5	14	38	47	2.1	95% (< 0.00010)

		alpha' chain fragment 1; Complement C3dg fragment; Complement C3g fragment; Complement C3d fragment; Complement C3f fragment; Complement C3c alpha' chain fragment 2]												
Q7L2H7	EIF3M	Eukaryotic translation initiation factor 3 subunit M (eIF3m) (Fetal lung protein B5) (hFL-B5) (PCI domain-containing protein 1)	11	22	0	5	1	5	6	10	4	2	2.1	95% (0.018)
Q9BW92	TARS2	Threonine--tRNA ligase, mitochondrial (EC 6.1.1.3) (Threonyl-tRNA synthetase) (ThrRS) (Threonyl-tRNA synthetase-like 1)	11	22	4	3	2	2	11	1	3	7	2.1	95% (0.018)
Q9BYD6	MRPL1	39S ribosomal protein L1, mitochondrial (L1mt) (MRP-L1)	9	18	5	1	3	0	8	3	3	4	2.1	95% (0.032)
J3KQ32	OLA1	Obg-like ATPase 1	9	18	0	2	2	5	3	6	4	5	2.1	95% (0.032)
Q02338	BDH1	D-beta-hydroxybutyrate dehydrogenase, mitochondrial (BDH) (EC 1.1.1.30) (3-hydroxybutyrate dehydrogenase)	17	33	7	1	0	8	13	12	7	1	2.1	95% (0.0054)
P49792	RANBP2	E3 SUMO-protein ligase RanBP2 (EC 6.3.2.-) (358 kDa nucleoporin) (Nuclear pore complex protein Nup358) (Nucleoporin Nup358) (Ran-binding protein 2) (RanBP2) (p270)	17	33	2	4	4	7	7	6	15	5	2.1	95% (0.0054)
Q8IY17	PNPLA6	Neuropathy target esterase (EC 3.1.1.5) (Patatin-like phospholipase domain-containing protein 6)	8	16	6	0	1	1	4	1	5	6	2.1	95% (0.042)
P52948	NUP98	Nuclear pore complex protein Nup98-Nup96 [Cleaved into: Nuclear pore complex protein Nup98 (98 kDa nucleoporin) (Nucleoporin Nup98) (Nup98); Nuclear pore complex protein Nup96 (96 kDa nucleoporin) (Nucleoporin Nup96) (Nup96)]	15	29	4	2	2	7	4	3	15	7	2.1	95% (0.0091)

P23526	AHCY	Adenosylhomocysteinase (AdoHcyase) (EC 3.3.1.1) (S-adenosyl-L-homocysteine hydrolase)	11	21	0	3	2	8	6	8	4	2	2.0	95% (0.027)
A8K3C3		T-complex protein 1 subunit delta	27	50	6	8	6	7	23	12	11	4	2.0	95% (0.0012)
Q13505	MTX1	Metaxin-1 (Mitochondrial outer membrane import complex protein 1)	10	19	3	0	0	7	6	4	5	4	2.0	95% (0.035)
B4DZ67	NUP107	Nuclear pore complex protein Nup107 (cDNA FLJ58739, highly similar to Nuclear pore complex protein Nup107)	10	19	3	3	1	3	3	2	10	4	2.0	95% (0.035)
Q96TC7	RMDN3	Regulator of microtubule dynamics protein 3 (RMD-3) (hRMD-3) (Cerebral protein 10) (Protein FAM82A2) (Protein FAM82C) (Protein tyrosine phosphatase-interacting protein 51) (TCPTP-interacting protein 51)	10	19	5	1	0	4	0	0	12	7	2.0	95% (0.035)
O15042	U2SURP	U2 snRNP-associated SURP motif-containing protein (140 kDa Ser/Arg-rich domain protein) (U2-associated protein SR140)	18	33	2	5	4	7	8	4	12	9	2.0	95% (0.0085)
F1T0J2	GOLGB1	Golgin subfamily B member 1	17	31	5	5	6	1	13	1	12	5	2.0	95% (0.011)
P0C0L5	C4B	Complement C4-B (Basic complement C4) (C3 and PZP-like alpha-2-macroglobulin domain-containing protein 3) [Cleaved into: Complement C4 beta chain; Complement C4-B alpha chain; C4a anaphylatoxin; C4b-B; C4d-B; Complement C4 gamma chain]	12	22	0	2	2	8	3	0	17	3	2.0	95% (0.029)
P02786	TFRC	Transferrin receptor protein 1 (TR) (TfR) (TfR1) (Trfr) (T9) (p90) (CD antigen CD71) [Cleaved into: Transferrin receptor protein 1, serum form (sTfR)]	117	205	29	34	37	20	59	36	47	65	2.0	95% (< 0.00010)
Q53EW8		Thiosulfate sulfurtransferase variant (Fragment)	30	13	12	8	6	4	7	3	0	3	-2.0	95% (0.019)

Q8NC56	LEMD2	LEM domain-containing protein 2 (hLEM2)	41	18	11	6	5	19	4	6	3	5	-2.0	95% (0.0072)
B7XGC2	HLA-A	MHC class I antigen (Fragment)	52	23	5	11	13	23	3	5	8	7	-2.0	95% (0.0028)
P02042	HBD	Hemoglobin subunit delta (Delta-globin) (Hemoglobin delta chain)	235	106	0	76	82	67	0	0	26	0	-2.0	95% (< 0.00010)
Q14254	FLOT2	Flotillin-2 (Epidermal surface antigen) (ESA) (Membrane component chromosome 17 surface marker 1)	49	21	23	10	8	10	2	9	3	7	-2.0	95% (0.0027)
H6A2E0	HLA-B	MHC class I antigen (Fragment)	38	16	0	9	0	15	0	0	7	0	-2.0	95% (0.0068)
A0S1I7	COX1	Cytochrome c oxidase subunit 1 (EC 1.9.3.1) (Fragment)	20	8	8	4	5	2	2	2	1	3	-2.1	95% (0.037)
P50570	DNM2	Dynamin-2 (EC 3.6.5.5)	20	8	0	1	9	9	5	3	1	0	-2.1	95% (0.037)
Q56VL3	OCIAD2	OCIA domain-containing protein 2 (Ovarian carcinoma immunoreactive antigen-like protein)	27	11	14	7	3	3	2	4	1	4	-2.1	95% (0.018)
Q6FGL0	LGALS3	Galectin (Fragment)	34	14	10	6	3	15	2	5	4	3	-2.1	95% (0.0088)
P16144	ITGB4	Integrin beta-4 (GP150) (CD antigen CD104)	39	16	30	4	4	1	2	0	2	12	-2.1	95% (0.0050)
Q9NUQ9	FAM49B	Protein FAM49B (L1)	23	9	1	11	5	6	0	4	4	1	-2.1	95% (0.023)
C8C504	HBB	Beta-globin	360	151	22	113	124	105	25	35	37	55	-2.1	95% (< 0.00010)
Q92520	FAM3C	Protein FAM3C (Interleukin-like EMT inducer)	50	20	18	10	8	14	4	5	5	6	-2.2	95% (0.0012)
Q9Y394	DHRS7	Dehydrogenase/reductase SDR family member 7 (EC 1.1.-.-) (Retinal short-chain dehydrogenase/reductase 4) (retSDR4)	38	15	18	8	7	5	0	5	3	7	-2.2	95% (0.0041)
Q14566	MCM6	DNA replication licensing factor MCM6 (EC 3.6.4.12) (p105MCM)	19	7	0	9	11	0	5	1	1	0	-2.2	95% (0.030)
Q99653	CHP1	Calcineurin B homologous protein 1 (Calcineurin B-like protein) (Calcium-binding protein CHP) (Calcium-binding protein p22) (EF-hand calcium-binding domain-containing protein p22)	24	9	12	3	4	5	1	3	1	3	-2.2	95% (0.016)

P56385	ATP5I	ATP synthase subunit e, mitochondrial (ATPase subunit e)	17	6	3	2	4	8	0	2	3	1	-2.2	95% (0.033)
Q86WA6	BPHL	Valacyclovir hydrolase (VACVase) (Valacyclovirase) (EC 3.1.-.-) (Biphenyl hydrolase-like protein) (Biphenyl hydrolase-related protein) (Bph-rp) (Breast epithelial mucin-associated antigen) (MCNAA)	17	6	3	1	5	8	0	0	1	5	-2.2	95% (0.033)
Q4AEJ3	hfzo1	Mitochondrial transmembrane GTPase Fzo-1	17	6	7	4	4	2	3	1	1	2	-2.2	95% (0.033)
P02730	SLC4A1	Band 3 anion transport protein (Anion exchange protein 1) (AE 1) (Anion exchanger 1) (Solute carrier family 4 member 1) (CD antigen CD233)	63	24	3	27	26	7	5	2	9	8	-2.3	95% (0.00016)
E7EVP7	ITPR1	Inositol 1,4,5-trisphosphate receptor type 1	25	9	14	4	7	0	1	0	1	7	-2.3	95% (0.012)
E3SWK8	HLA-C	MHC class I antigen (Fragment)	28	10	0	8	0	12	0	0	4	0	-2.3	95% (0.0073)
P27701	CD82	CD82 antigen (C33 antigen) (IA4) (Inducible membrane protein R2) (Metastasis suppressor Kangai-1) (Suppressor of tumorigenicity 6 protein) (Tetraspanin-27) (Tspan-27) (CD antigen CD82)	44	16	13	12	15	4	5	1	5	5	-2.3	95% (0.00097)
Q16555	DPYSL2	Dihydropyrimidinase-related protein 2 (DRP-2) (Collapsin response mediator protein 2) (CRMP-2) (N2A3) (Unc-33-like phosphoprotein 2) (ULIP-2)	21	7	2	11	8	0	0	2	2	0	-2.4	95% (0.014)
P29728	OAS2	2'-5'-oligoadenylate synthase 2 ((2-5')oligo(A) synthase 2) (2-5A synthase 2) (EC 2.7.7.84) (p69 OAS / p71 OAS) (p69OAS / p71OAS)	21	7	0	2	2	17	0	7	0	0	-2.4	95% (0.014)
Q96IU4	ABHD14B	Alpha/beta hydrolase domain-containing protein 14B (Abhydrolase domain-containing protein 14B) (EC 3.-.-.-) (CCG1-interacting factor B)	13	4	2	3	3	5	0	3	1	0	-2.4	95% (0.042)
O14657	TOR1B	Torsin-1B (Torsin family 1 member B)	13	4	0	2	2	9	0	1	0	3	-2.4	95% (0.042)

Q96CW1	AP2M1	AP-2 complex subunit mu (AP-2 mu chain) (Adapter-related protein complex 2 subunit mu) (Adaptin-mu2) (Adaptor protein complex AP-2 subunit mu) (Clathrin assembly protein complex 2 mu medium chain) (Clathrin coat assembly protein AP50) (Clathrin coat-associated protein AP50) (HA2 50 kDa subunit) (Plasma membrane adaptor AP-2 50 kDa protein)	32	11	10	9	5	9	3	1	3	4	-2.4	95% (0.0032)
O95294	RASAL1	RasGAP-activating-like protein 1	19	6	19	0	0	0	0	0	0	6	-2.5	95% (0.016)
B4DGM9	TOR1A	Torsin family 1, member A (Torsin A), isoform CRA_a (cDNA FLJ56343, highly similar to Torsin A)	33	11	8	8	4	14	0	4	3	4	-2.5	95% (0.0022)
P21589	NT5E	5'-nucleotidase (5'-NT) (EC 3.1.3.5) (Ecto-5'-nucleotidase) (CD antigen CD73)	22	7	7	9	7	0	0	3	2	2	-2.5	95% (0.0098)
P05109	S100A8	Protein S100-A8 (Calgranulin-A) (Calprotectin L1L subunit) (Cystic fibrosis antigen) (CFAG) (Leukocyte L1 complex light chain) (Migration inhibitory factor-related protein 8) (MRP-8) (p8) (S100 calcium-binding protein A8) (Urinary stone protein band A) [Cleaved into: Protein S100-A8, N-terminally processed]	53	18	1	22	20	10	0	5	5	8	-2.5	95% (0.00014)
Q14573	ITPR3	Inositol 1,4,5-trisphosphate receptor type 3 (IP3 receptor isoform 3) (IP3R 3) (InsP3R3) (Type 3 inositol 1,4,5-trisphosphate receptor) (Type 3 InsP3 receptor)	138	48	85	21	29	5	6	0	32	10	-2.5	95% (< 0.00010)
Q9Y2A7	NCKAP1	Nck-associated protein 1 (NAP 1) (Membrane-associated protein HEM-2) (p125Nap1)	28	9	11	5	6	5	2	3	1	2	-2.5	95% (0.0038)
P39687	ANP32A	Acidic leucine-rich nuclear phosphoprotein 32 family member A (Acidic nuclear phosphoprotein	11	3	1	3	1	7	1	0	2	0	-2.6	95% (0.046)

		pp32) (Leucine-rich acidic nuclear protein) (LANP) (Mapmodulin) (Potent heat-stable protein phosphatase 2A inhibitor I1PP2A) (Putative HLA-DR-associated protein I) (PHAPI)											
Q96BM9	ARL8A	ADP-ribosylation factor-like protein 8A (ADP-ribosylation factor-like protein 10B) (Novel small G protein indispensable for equal chromosome segregation 2)	40	13	14	10	8	8	0	4	3	6	-2.6	95% (0.00063)
Q9UKV8	AGO2	Protein argonaute-2 (Argonaute2) (hAgo2) (EC 3.1.26.n2) (Argonaute RISC catalytic component 2) (Eukaryotic translation initiation factor 2C 2) (eIF-2C 2) (eIF2C 2) (PAZ Piwi domain protein) (PPD) (Protein slicer)	14	4	0	6	8	1	1	0	2	1	-2.6	95% (0.028)
Q9H4M9	EHD1	EH domain-containing protein 1 (PAST homolog 1) (hPAST1) (Testilin)	14	4	5	4	1	4	0	2	2	0	-2.6	95% (0.028)
Q13868	EXOSC2	Exosome complex component RRP4 (Exosome component 2) (Ribosomal RNA-processing protein 4)	14	4	0	4	4	6	1	1	2	0	-2.6	95% (0.028)
P00390	GSR	Glutathione reductase, mitochondrial (GR) (GRase) (EC 1.8.1.7)	17	5	2	7	7	1	1	0	2	2	-2.6	95% (0.017)
Q0PNF2		FEX1	17	5	0	7	9	0	0	0	1	4	-2.6	95% (0.017)
O15400	STX7	Syntaxin-7	20	6	9	4	4	3	0	0	2	4	-2.6	95% (0.011)
P37235	HPCAL1	Hippocalcin-like protein 1 (Calcium-binding protein BDR-1) (HLP2) (Visinin-like protein 3) (VILIP-3)	23	7	5	7	9	2	0	1	3	3	-2.6	95% (0.0066)
P09429	HMGB1	High mobility group protein B1 (High mobility group protein 1) (HMG-1)	29	9	8	5	5	11	2	1	1	5	-2.6	95% (0.0026)
P17301	ITGA2	Integrin alpha-2 (CD49 antigen-like family member B) (Collagen receptor) (Platelet membrane	24	7	10	7	7	0	0	0	0	7	-2.7	95% (0.0045)

		glycoprotein Ia) (GPIa) (VLA-2 subunit alpha) (CD antigen CD49b)												
Q9UQB8	BAIAP2	Brain-specific angiogenesis inhibitor 1-associated protein 2 (BAI-associated protein 2) (BAI1-associated protein 2) (Protein BAP2) (Fas ligand-associated factor 3) (FLAF3) (Insulin receptor substrate p53/p58) (IRS-58) (IRSp53/58) (Insulin receptor substrate protein of 53 kDa) (IRSp53) (Insulin receptor substrate p53)	21	6	8	4	4	5	1	0	3	2	-2.7	95% (0.0071)
Q9H1E5	TMX4	Thioredoxin-related transmembrane protein 4 (Thioredoxin domain-containing protein 13)	21	6	8	3	5	5	0	1	2	3	-2.7	95% (0.0071)
Q9BUP0	EFHD1	EF-hand domain-containing protein D1 (EF-hand domain-containing protein 1) (Swiprosin-2)	15	4	0	6	7	2	0	2	1	1	-2.8	95% (0.018)
J3QKR5	CDK11B	Cyclin-dependent kinase 11B	12	3	0	3	4	5	1	0	1	1	-2.8	95% (0.030)
P36915	GNL1	Guanine nucleotide-binding protein-like 1 (GTP-binding protein HSR1)	12	3	0	1	2	9	1	1	1	0	-2.8	95% (0.030)
O60664	PLIN3	Perilipin-3 (47 kDa mannose 6-phosphate receptor-binding protein) (47 kDa MPR-binding protein) (Cargo selection protein TIP47) (Mannose-6-phosphate receptor-binding protein 1) (Placental protein 17) (PP17)	12	3	8	1	1	2	0	0	0	3	-2.8	95% (0.030)
Q6PL18	ATAD2	ATPase family AAA domain-containing protein 2 (EC 3.6.1.3) (AAA nuclear coregulator cancer-associated protein) (ANCCA)	9	2	0	4	5	0	0	0	2	0	-2.8	95% (0.050)
Q96ER9	CCDC51	Coiled-coil domain-containing protein 51	9	2	1	0	0	8	0	0	0	2	-2.8	95% (0.050)
Q92522	H1FX	Histone H1x	9	2	0	2	3	4	0	0	0	2	-2.8	95% (0.050)
B4DG22	RPS6KA3	Ribosomal protein S6 kinase alpha-3 (cDNA FLJ56618, highly similar to Ribosomal protein S6 kinase alpha-3 (EC 2.7.11.1))	9	2	0	4	4	1	1	1	0	0	-2.8	95% (0.050)

Q86WV6	TMEM173	Stimulator of interferon genes protein (hSTING) (Endoplasmic reticulum interferon stimulator) (ERIS) (Mediator of IRF3 activation) (hMITA) (Transmembrane protein 173)	25	7	8	5	1	11	0	2	2	3	-2.8	95% (0.0030)
O94905	ERLIN2	Erlin-2 (Endoplasmic reticulum lipid raft-associated protein 2) (Stomatin-prohibitin-flotillin-HflC/K domain-containing protein 2) (SPFH domain-containing protein 2)	61	18	19	13	14	15	1	5	4	8	-2.9	95% (< 0.00010)
Q3SXM5	HSDL1	Inactive hydroxysteroid dehydrogenase-like protein 1	16	4	9	2	4	1	1	0	1	2	-2.9	95% (0.012)
Q14108	SCARB2	Lysosome membrane protein 2 (85 kDa lysosomal membrane sialoglycoprotein) (LGP85) (CD36 antigen-like 2) (Lysosome membrane protein II) (LIMP II) (Scavenger receptor class B member 2) (CD antigen CD36)	26	7	9	7	6	4	0	1	4	3	-3.0	95% (0.0020)
P12109	COL6A1	Collagen alpha-1(VI) chain	13	3	1	7	5	0	0	0	0	3	-3.0	95% (0.019)
H3BN55	RAB27A	Ras-related protein Rab-27A (Fragment)	13	3	10	0	2	1	0	0	2	1	-3.0	95% (0.019)
P58107	EPPK1	Epiplakin (450 kDa epidermal antigen)	134	39	53	21	31	31	22	3	12	2	-3.0	95% (< 0.00010)
Q9Y276	BCS1L	Mitochondrial chaperone BCS1 (h-BCS1) (BCS1-like protein)	10	2	3	3	2	2	0	2	0	0	-3.1	95% (0.031)
B7ZKL3	EPS8L2	EPS8L2 protein (Epidermal growth factor receptor kinase substrate 8-like protein 2)	10	2	6	1	2	1	2	0	0	0	-3.1	95% (0.031)
B4DKK2	SRI	Sorcin (cDNA FLJ60640, highly similar to Sorcin)	10	2	3	3	1	3	0	2	0	0	-3.1	95% (0.031)
P12532	CKMT1A	Creatine kinase U-type, mitochondrial (EC 2.7.3.2) (Acidic-type mitochondrial creatine kinase) (Mia-CK) (Ubiquitous mitochondrial creatine kinase) (U-MtCK)	31	8	13	5	3	11	2	0	6	0	-3.1	95% (0.00055)

O75911	DHRS3	Short-chain dehydrogenase/reductase 3 (EC 1.1.1.300) (DD83.1) (Retinal short-chain dehydrogenase/reductase 1) (retSDR1)	21	5	12	2	7	0	0	1	3	1	-3.2	95% (0.0031)
H3BPE1	MACF1	Microtubule-actin cross-linking factor 1, isoforms 1/2/3/5	43	11	9	11	19	5	4	0	7	0	-3.2	95% (< 0.00010)
Q14019	COTL1	Coactosin-like protein	7	1	0	2	5	0	0	0	1	0	-3.3	95% (0.050)
Q7KZN9	COX15	Cytochrome c oxidase assembly protein COX15 homolog	7	1	3	0	0	4	0	1	0	0	-3.3	95% (0.050)
P14854	COX6B1	Cytochrome c oxidase subunit 6B1 (Cytochrome c oxidase subunit VIb isoform 1) (COX VIb-1)	7	1	1	1	2	3	1	0	0	0	-3.3	95% (0.050)
B1AUU8	EPS15	Epidermal growth factor receptor substrate 15	7	1	1	4	2	0	1	0	0	0	-3.3	95% (0.050)
Q6ZRV2	FAM83H	Protein FAM83H	7	1	5	0	0	2	1	0	0	0	-3.3	95% (0.050)
P26012	ITGB8	Integrin beta-8	7	1	3	1	1	2	0	0	1	0	-3.3	95% (0.050)
G3V2F7	TMEM189	HCG2044781 (Protein TMEM189-UBE2V1) (Transmembrane protein 189)	7	1	1	1	3	2	1	0	0	0	-3.3	95% (0.050)
D3DQ48	TMEM59	Transmembrane protein 59, isoform CRA_e	7	1	0	4	3	0	1	0	0	0	-3.3	95% (0.050)
Q9H269	VPS16	Vacuolar protein sorting-associated protein 16 homolog (hVPS16)	7	1	2	2	1	2	0	1	0	0	-3.3	95% (0.050)
Q6YHK3	CD109	CD109 antigen (150 kDa TGF-beta-1-binding protein) (C3 and PZP-like alpha-2-macroglobulin domain-containing protein 7) (Platelet-specific Gov antigen) (p180) (r150) (CD antigen CD109)	19	4	6	7	6	0	2	0	2	0	-3.4	95% (0.0031)
O76027	ANXA9	Annexin A9 (Annexin XXXI) (Annexin-31) (Annexin-9) (Pemphaxin)	23	5	13	0	1	10	5	0	0	0	-3.5	95% (0.0013)
P55011	SLC12A2	Solute carrier family 12 member 2 (Basolateral Na-K-Cl symporter) (Bumetanide-sensitive sodium-(potassium)-chloride cotransporter 1)	31	7	1	14	17	0	5	0	1	1	-3.5	95% (0.00023)
P27695	APEX1	DNA-(apurinic or apyrimidinic site) lyase (EC 3.1.-.-) (EC 4.2.99.18)	16	3	0	1	1	14	1	1	1	0	-3.6	95% (0.0047)

		(APEX nuclease) (APEN) (Apurinic-apyrimidinic endonuclease 1) (AP endonuclease 1) (APE-1) (REF-1) (Redox factor-1) [Cleaved into: DNA-(apurinic or apyrimidinic site) lyase, mitochondrial]												
A4D2Q0	UNC84A	SUN domain-containing protein 1 (Unc-84 homolog A (C. elegans))	16	3	13	2	0	2	1	0	0	2	-3.6	95% (0.0047)
Q7L5L3	GDPD3	Glycerophosphodiester phosphodiesterase domain-containing protein 3 (EC 3.1.-.-)	12	2	1	1	0	10	1	0	1	0	-3.6	95% (0.012)
Q9H3Z4	DNAJC5	DnaJ homolog subfamily C member 5 (Cysteine string protein) (CSP)	8	1	2	2	2	2	0	0	0	1	-3.7	95% (0.029)
H0Y6T6	NRM	Nurim (Fragment)	8	1	2	0	0	6	0	0	0	1	-3.7	95% (0.029)
F8J390	PKP3	Plakophilin 3b (Fragment)	8	1	4	0	2	2	1	0	0	0	-3.7	95% (0.029)
P49903	SEPHS1	Selenide, water dikinase 1 (EC 2.7.9.3) (Selenium donor protein 1) (Selenophosphate synthase 1)	8	1	0	3	1	4	1	0	0	0	-3.7	95% (0.029)
P37837	TALDO1	Transaldolase (EC 2.2.1.2)	21	4	2	2	9	8	1	1	1	1	-3.8	95% (0.0012)
Q5HYL6	DKFZp686E1899	Putative uncharacterized protein DKFZp686E1899	13	2	1	1	7	4	0	0	2	0	-3.9	95% (0.0070)
Q59FB9		Toll interacting protein variant (Fragment)	13	2	6	4	2	2	0	0	1	1	-3.9	95% (0.0070)
F8VXY3	OAS1	2'-5'-oligoadenylate synthase 1	18	3	0	0	0	18	0	3	0	0	-4.0	95% (0.0018)
E5KSE7		Mitochondrial short-chain specific acyl-CoA dehydrogenase	18	3	5	5	3	5	0	1	0	1	-4.0	95% (0.0018)
P33897	ABCD1	ATP-binding cassette sub-family D member 1 (Adrenoleukodystrophy protein) (ALDP)	9	1	4	1	0	4	0	0	1	0	-4.1	95% (0.017)
Q9NTX5	ECHDC1	Ethylmalonyl-CoA decarboxylase (EC 4.1.1.94) (Enoyl-CoA hydratase domain-containing protein 1) (Methylmalonyl-CoA decarboxylase) (MMCD) (EC 4.1.1.41)	9	1	3	2	1	3	0	0	1	0	-4.1	95% (0.017)
O43920	NDUFS5	NADH dehydrogenase [ubiquinone] iron-sulfur protein 5 (Complex I-15 kDa) (CI-15 kDa) (NADH-	9	1	3	2	2	2	0	0	1	0	-4.1	95% (0.017)

		ubiquinone oxidoreductase 15 kDa subunit)												
Q00796	SORD	Sorbitol dehydrogenase (EC 1.1.1.14) (L-iditol 2-dehydrogenase)	9	1	0	0	0	9	0	0	1	0	-4.1	95% (0.017)
Q15149	PLEC	Plectin (PCN) (PLTN) (Hemidesmosomal protein 1) (HD1) (Plectin-1)	1115	246	418	276	278	146	34	22	108	83	-4.1	95% (< 0.00010)
P16157	ANK1	Ankyrin-1 (ANK-1) (Ankyrin-R) (Erythrocyte ankyrin)	28	5	0	12	16	0	0	0	5	0	-4.2	95% (0.00012)
Q99828	CIB1	Calcium and integrin-binding protein 1 (CIB) (Calcium- and integrin-binding protein) (CIBP) (Calmyrin) (DNA-PKcs-interacting protein) (Kinase-interacting protein) (KIP) (SNK-interacting protein 2-28) (SIP2-28)	14	2	4	2	0	8	0	0	0	2	-4.2	95% (0.0042)
P05187	ALPP	Alkaline phosphatase, placental type (EC 3.1.3.1) (Alkaline phosphatase Regan isozyme) (Placental alkaline phosphatase 1) (PLAP-1)	24	4	14	5	5	0	0	0	0	4	-4.3	95% (0.00028)
O00194	RAB27B	Ras-related protein Rab-27B (C25KG)	10	1	7	1	2	0	0	0	1	0	-4.5	95% (0.0098)
P05164	MPO	Myeloperoxidase (MPO) (EC 1.11.2.2) [Cleaved into: Myeloperoxidase; 89 kDa myeloperoxidase; 84 kDa myeloperoxidase; Myeloperoxidase light chain; Myeloperoxidase heavy chain]	25	4	12	0	0	13	0	1	3	0	-4.5	95% (0.00017)
B2RMN7	SPTB	Spectrin, beta, erythrocytic	16	2	0	6	10	0	1	0	0	0	-4.7	95% (0.0015)
Q8TE68	EPS8L1	Epidermal growth factor receptor kinase substrate 8-like protein 1 (EPS8-like protein 1) (Epidermal growth factor receptor pathway substrate 8-related protein 1) (EPS8-related protein 1)	11	1	11	0	0	1	0	0	0	2	-4.9	95% (0.0056)
P08582	MFI2	Melanotransferrin (Melanoma-associated antigen p97) (CD antigen CD228)	11	1	6	3	2	0	0	0	1	0	-4.9	95% (0.0056)

P43155	CRAT	Carnitine O-acetyltransferase (Carnitine acetylase) (EC 2.3.1.7) (Carnitine acetyltransferase) (CAT) (CrAT)	11	1	1	1	5	4	0	1	0	0	-4.9	95% (0.0056)
A8K8G0	HDGF	Hepatoma-derived growth factor (cDNA FLJ75113)	29	4	4	6	8	11	0	2	0	2	-5.1	95% (< 0.00010)
Q13753	LAMC2	Laminin subunit gamma-2 (Cell-scattering factor 140 kDa subunit) (CSF 140 kDa subunit) (Epiligrin subunit gamma) (Kalinin subunit gamma) (Kalinin/nicein/epiligrin 100 kDa subunit) (Ladsin 140 kDa subunit) (Laminin B2t chain) (Laminin-5 subunit gamma) (Large adhesive scatter factor 140 kDa subunit) (Nicein subunit gamma)	30	4	4	12	14	0	0	0	0	3	-5.3	95% (< 0.00010)
P26583	HMGB2	High mobility group protein B2 (High mobility group protein 2) (HMG-2)	13	1	0	1	3	7	1	0	0	0	-5.7	95% (0.0018)
P09913	IFIT2	Interferon-induced protein with tetratricopeptide repeats 2 (IFIT-2) (ISG-54 K) (Interferon-induced 54 kDa protein) (IFI-54K) (P54)	13	1	0	0	0	13	0	1	0	0	-5.7	95% (0.0018)
K7EKI8	PPL	Periplakin	187	25	98	25	18	42	11	0	7	7	-6.4	95% (< 0.00010)
B4DKB2	ECE1	Endothelin-converting enzyme 1 (cDNA FLJ59212, highly similar to Endothelin-converting enzyme 1 (EC 3.4.24.71))	38	4	11	13	15	0	3	0	1	0	-6.7	95% (< 0.00010)
Q16647	PTGIS	Prostacyclin synthase (EC 5.3.99.4) (Prostaglandin I2 synthase)	40	3	35	0	0	5	0	0	0	3	-8.7	95% (< 0.00010)
B7Z416	DHRS9	Dehydrogenase/reductase SDR family member 9 (cDNA FLJ55219, highly similar to Dehydrogenase/reductase SDR family member 9 (EC 1.1.-.-))	46	2	18	18	18	18	0	0	0	2	-13.0	95% (< 0.00010)

[A]Obtained from UniProt (http://www.uniprot.org/) databases
[C]Spectral counts (SpC) in CN datasets (combined)
[D]Spectral counts (SpC) in CR datasets (combined)
[E]Spectral count ratio (Rsc) between CR (combined) and CN (combined)
[F]Statistical significance (p-value) (Fisher's Exact Test)
